# A member of wheat class III peroxidase gene family, *TaPRX-2A,* enhanced the tolerance of salt stress

**DOI:** 10.1186/s12870-020-02602-1

**Published:** 2020-08-26

**Authors:** Peisen Su, Jun Yan, Wen Li, Liang Wang, Jinxiao Zhao, Xin Ma, Anfei Li, Hongwei Wang, Lingrang Kong

**Affiliations:** 1 State Key Laboratory of Crop Biology, Shandong Key Laboratory of Crop Biology, College of Agronomy, Shandong Agricultural University, Tai’an, 271018 People’s Republic of China; 2 College of Information Science and Engineering, Shandong Agricultural University, Tai’an, Shandong 271018 People’s Republic of China

**Keywords:** Wheat, Salinity stresses, Peroxidases, *TaPRX-2A*

## Abstract

**Background:**

Salt and drought are the main abiotic stresses that restrict the yield of crops. Peroxidases (PRXs) are involved in various abiotic stress responses. Furthermore, only few wheat PRXs have been characterized in the mechanism of the abiotic stress response.

**Results:**

In this study, a novel wheat peroxidase (PRX) gene named *TaPRX-2A,* a member of wheat class III PRX gene family, was cloned and its response to salt stress was characterized. Based on the identification and evolutionary analysis of class III PRXs in 12 plants, we proposed an evolutionary model for *TaPRX-2A*, suggesting that occurrence of some exon fusion events during evolution. We also detected the positive selection of PRX domain in 13 PRXs involving our evolutionary model, and found 2 or 6 positively selected sites during *TaPRX-2A* evolution. Quantitative reverse transcription–polymerase chain reaction (qRT–PCR) results showed that *TaPRX-2A* exhibited relatively higher expression levels in root tissue than those exhibited in leaf and stem tissues. *TaPRX-2A* expression was also induced by abiotic stresses and hormone treatments such as polyethylene glycol 6000, NaCl, hydrogen peroxide (H_2_O_2_), salicylic acid (SA), methyljasmonic acid (MeJA) and abscisic acid (ABA). Transgenic wheat plants with overexpression of *TaPRX-2A* showed higher tolerance to salt stress than wild-type (WT) plants. Confocal microscopy revealed that *TaPRX-2A*-eGFP was mainly localized in cell nuclei. Survival rate, relative water content, and shoot length were higher in *TaPRX-2A*-overexpressing wheat than in the WT wheat, whereas root length was not significantly different. The activities of superoxide dismutase (SOD), peroxidase (POD), and catalase (CAT) were enhanced in *TaPRX-2A*-overexpressing wheat compared with those in the WT wheat, resulting in the reduction of reactive oxygen species (ROS) accumulation and malondialdehyde (MDA) content. The expression levels of downstream stress-related genes showed that *RD22*, *TLP4*, *ABAI*, *GST22*, *FeSOD*, and *CAT* exhibited higher expressions in *TaPRX-2A*-overexpressing wheat than in WT under salt stress.

**Conclusions:**

The results show that *TaPRX-2A* plays a positive role in the response to salt stress by scavenging ROS and regulating stress-related genes.

## Background

Abiotic stresses such as high salinity and drought have profound negative impacts on plant development and biomass formation, resulting in significant reductions in crop yield worldwide [1]. To adapt to these abiotic stresses, plants have evolved complex mechanisms for physiological and biochemical mitigation of stress-associated damage, such as the release of reactive oxygen species (ROS) [2, 3]. Previous studies have shown that hydrogen peroxide (H_2_O_2_) pretreatment can improve the salt tolerance of wheat by modulating antioxidant enzyme activity, mineral uptake, and proline levels [4]. In the cells of higher plant, ROS exists in many forms, including H_2_O_2_, superoxide radicals (O_2_^.−^), and hydroxyl radicals (OH^−^). ROS are generated under abiotic conditions, and cause rapid cell damage by damaging membrane lipids, nucleic acids [5]. Plants have established a complex system to scavenge ROS for maintaining the steady-state level of ROS by activating the antioxidant system. In this case, the antioxidant system refers mainly to free-radical scavenging by several endogenous antioxidant enzymes, such as glutathione peroxidase (GPX), ascorbate peroxidase (APX), catalase (CAT), and superoxide dismutase (SOD) [6–8]. It has been reported that antioxidant enzymes (e.g.*,* APX, CAT) are also altered when plants experience salt stress [3, 9, 10]. In addition, PRXs have been reported to protect cells against ROS by catalyzing redox reactions [11].

PRXs exist in many species, such as microorganisms, animals, and plants [12–15]. These compounds are divided into three superfamilies based on different molecular structures and catalytic properties. The first superfamily includes animal enzymes, such as eosinophil PRX and GPX. The second superfamily is widely distributed in many species (bacteria, animals, fungi, plants, and yeast). The third superfamily is found in plants, bacteria, and fungi [14, 15]. According to differences in primary protein structure, PRXs are divided into three classes-class I PRXs found intracellularly, class II PRXs found extracellularly, and class III PRXs comprises large multigene families [16]. Class I PRXs play key roles in scavenging excess H_2_O_2_ [16–18], class II PRXs (found in fungi) are involved in the degradation of soil debris [16, 19], and class III PRXs are plant-specific [20]. More than 110 class III PRXs have been identified in allohexaploid wheat [21]. *Oryza sativa* comprises 138 class III PRXs [22]. Seventy-three sequences encode class III PRXs of *Arabidopsis thaliana,* and 119 class III PRXs have been identified in maize [12, 23]. *Populus trichocarpa* contains 93 class III PRXs [24].

Class III PRXs have various functions in plant development processes, including cell wall hardening, crosslinking of cell wall components, defense against pathogen infection, H_2_O_2_ removal, and wounding [12, 16, 25]. A large number of PRXs have been studied in *A. thaliana* and their functions have been demonstrated. For example, *AtPRX72* plays an important role in lignification [26], whereas *AtPRX33* and *AtPRX34* were identified to play a role in cell elongation [16]. Some studies have demonstrated that *AtPRX21, AtPRX62*, and *AtPRX71* are secreted as part of a response to wounding and fungal stresses [27, 28]. The *Gossypium hirsutum* gene *GhPOX1* may cause cotton fiber cell elongation through ROS production [29]. Some PRXs have been reported to play central roles in host plant defenses against necrotrophic or biotrophic pathogens by coordinating salicylic acid (SA), jasmonic acid (MeJA), and ethylene (ET) [20]. For example, *TaPRX111, TaPRX112*, and *TaPRX113* are involved in plant response to nematode infection in wheat [30]. In rice, the expression patterns of PRXs revealed important functional diversity, particularly in response to stresses [31]. The *Zea mays* PRXs *ZmPRX26*, *ZmPRX42*, *ZmPRX71*, *ZmPRX75*, and *ZmPRX78* are involved in the response to various abiotic stress conditions [12]. Furthermore, GPXs are members of the PRX family, and they have important functions in plants. The *A. thaliana* GPX gene, *AtGPX3,* acts as a general scavenger and signal transducer under drought stress and abscisic acid (ABA)-mediated signaling [32]. Six *GPXs* in *Cucumis sativus* were found to respond to ABA treatments and abiotic stress. Moreover, five rice GPXs were known to play roles in response against H_2_O_2_ and cold stress [33]. Several wheat PRXs have also been discovered to play a role in drought resistance, as revealed by a microarray experiment [34]. Two wheat GPXs, W69 and W106, have been shown to improve salt tolerance in transgenic *Arabidopsis* [7].

Wheat is an important commercial crop worldwide, but its yield is often restricted by abiotic stresses [35, 36]. The roles of some PRXs in tolerance against salinity stress have been reported; however, the molecular mechanisms of wheat PRXs underlying these responses remain to be fully understood. In this study, we cloned a PRX gene *TaPRX-2A* from wheat (*Triticum aestivum*) and investigated tolerance against salt stress conferred by *TaPRX-2A* in transgenic wheat. Evolutionary analysis revealed that some exon fusion events and positive selection might have occurred during *TaPRX-2A* evolution. Gene expression pattern analysis demonstrated that *TaPRX-2A* expression was upregulated by drought, salt, H_2_O_2_, and ABA treatments. Our results showed that *TaPRX-2A* improved the tolerance of wheat against salt by improving antioxidative stress ability and regulating stress-related genes. Our work will give the researchers with new insights into abiotic stress tolerance mechanisms in plants.

## Results

### Isolation and evolution of *TaPRX-2A*

To obtain further insights into the evolutionary conservation or divergence of genes among class III PRXs, we identified, classified, and described the gene structures of class III PRXs. PRXs of 12 plants (*T. aestivum*, *Triticum dicoccoides*, *Triticum urartu*, *Aegilops tauschii*, *Brachypodium distachyon*, *O. sativa*, *Z. mays*, *A. thaliana*, *Vitis vinifera*, *Selaginella moellendorffii*, *Physcomitrella patens*, *and Chlamydomonas reinhardtii*) were identified by HMMER 3.1 and Pfam 32.0 in batch mode with the PRX domain (peroxidase.hmm, PF00141.23) (Additional file 1: Table S1, and Additional file 2: Table S2). We excluded the atypical PRXs of these 12 plants that showed < 50% alignment with the PRX domain (Additional file 3: Table S3). The classification of these PRXs was based on two methods, HMMER3.1 scan and neighbour-joining (NJ) phylogenetic reconstruction (Additional file 2: Table S2 and Additional file 4: Figure S1). The exon-intron structures within the PRXs domain were also examined in the 12 plants (Additional file 5: Figure S2).

**Fig. 1:**
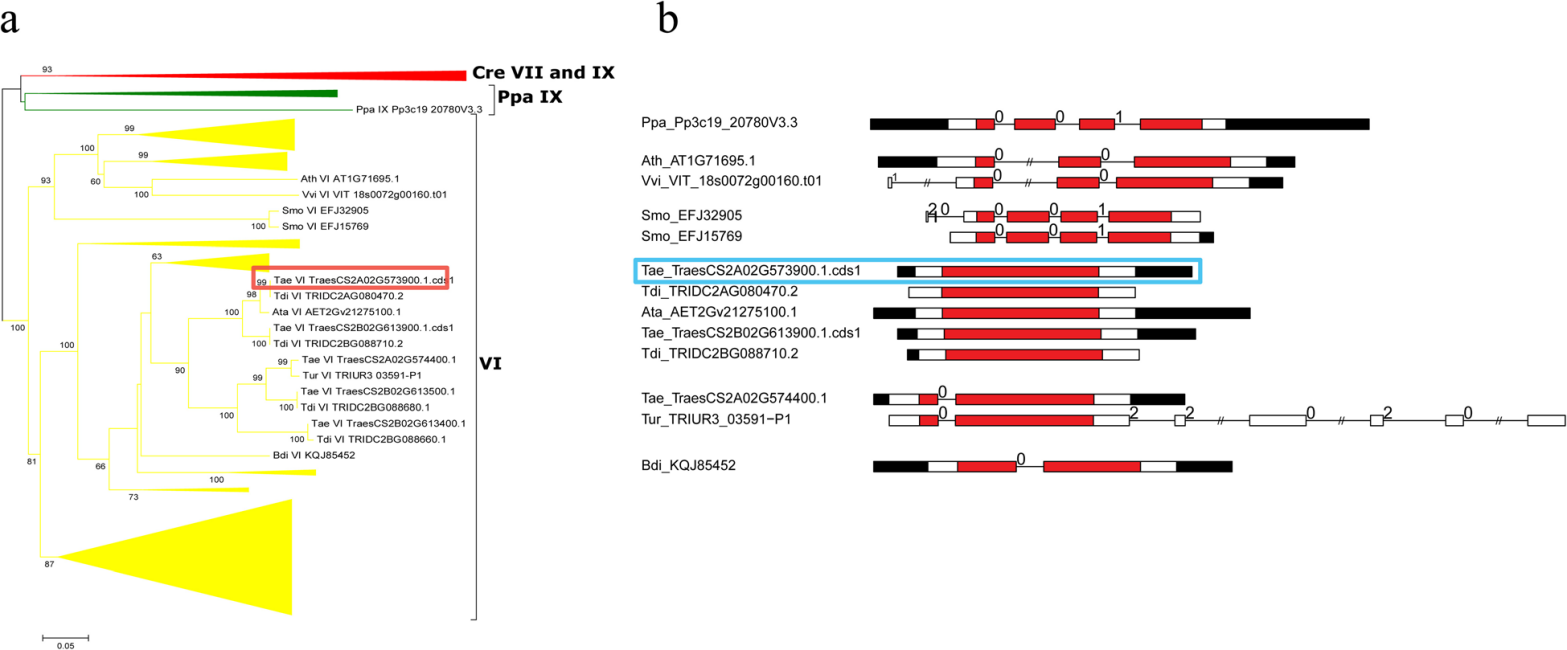
Phylogenetic tree and gene structures of *TaPRX-2A* and related PRXs in wheat *Ae. tauschii,* and other plants. **a** The neighbor-joining tree elaborated in this study. Amino acid sequences of the PRX domain were used to construct the neighbor-joining tree using the MEGA-CC 7.0 software with the p-distance model. Most sequences belong to subfamily VI of class III PRX and some branches are compressed. Detailed information is showed in Additional file 4: Figure S1. **b** The exon–intron structures of some PRXs examined in this study. Red boxes represent the PRX domain; white boxes represent other exon regions; black boxes represent untranslated regions (UTRs); lines represent the PRX introns; numbers 0, 1, and 2 represent the exon phases. The long introns are shortened by “//.” Our investigated PRX (TraesCS2A02G573900.1.cds1) in *T. aestivum* is circled by a red or cyan box

Among them, we cloned one member (named *TaPRX-2A*) of the PRXs obtained from the wheat cultivar “Sumai 3.” The predicted *TaPRX-2A* open reading frame (ORF) is 1026 bp, and the deduced *TaPRX-2A* protein comprises 342 amino acid residues. BLAST (basic local alignment search tool) results from the National Center for Biotechnology Information (NCBI) showed that a PRX gene (GenBank: AJ878510.1) in the *T. aestivum* cultivar “Cheyenne” contained the minimum E value. Our local BLAST against the identified PRXs of the 12 plants showed that the *T. aestivum* PRX TraesCS2A02G573900.1.cds1 from subfamily VI contained 100% sequence similarity with *TaPRX-2A.* To investigate the evolution of this clone, we reconstructed a small NJ phylogenetic tree only containing subfamily VI PRXs from these 12 plants and compared their structural features (Fig. 1a, b). As shown, the exon–intron structure of the *T. aestivum* clone (TraesCS2A02G573900.1.cds1) was a one-exon structure, whereas the other four wheat and *Ae. tauschii* homologous PRXs (Tdi_TRIDC2AG080470.2, Ata_AET2Gv21275100.1, Tae_TraesCS2B02G613900.1.cds1, and Tdi_TRIDC2BG088710.2) in this clade also had a one-exon structure, suggesting that this one-exon structure originated in these PRXs before the *Triticum–Aegilops* split (Fig. 1b).

**Fig. 2:**
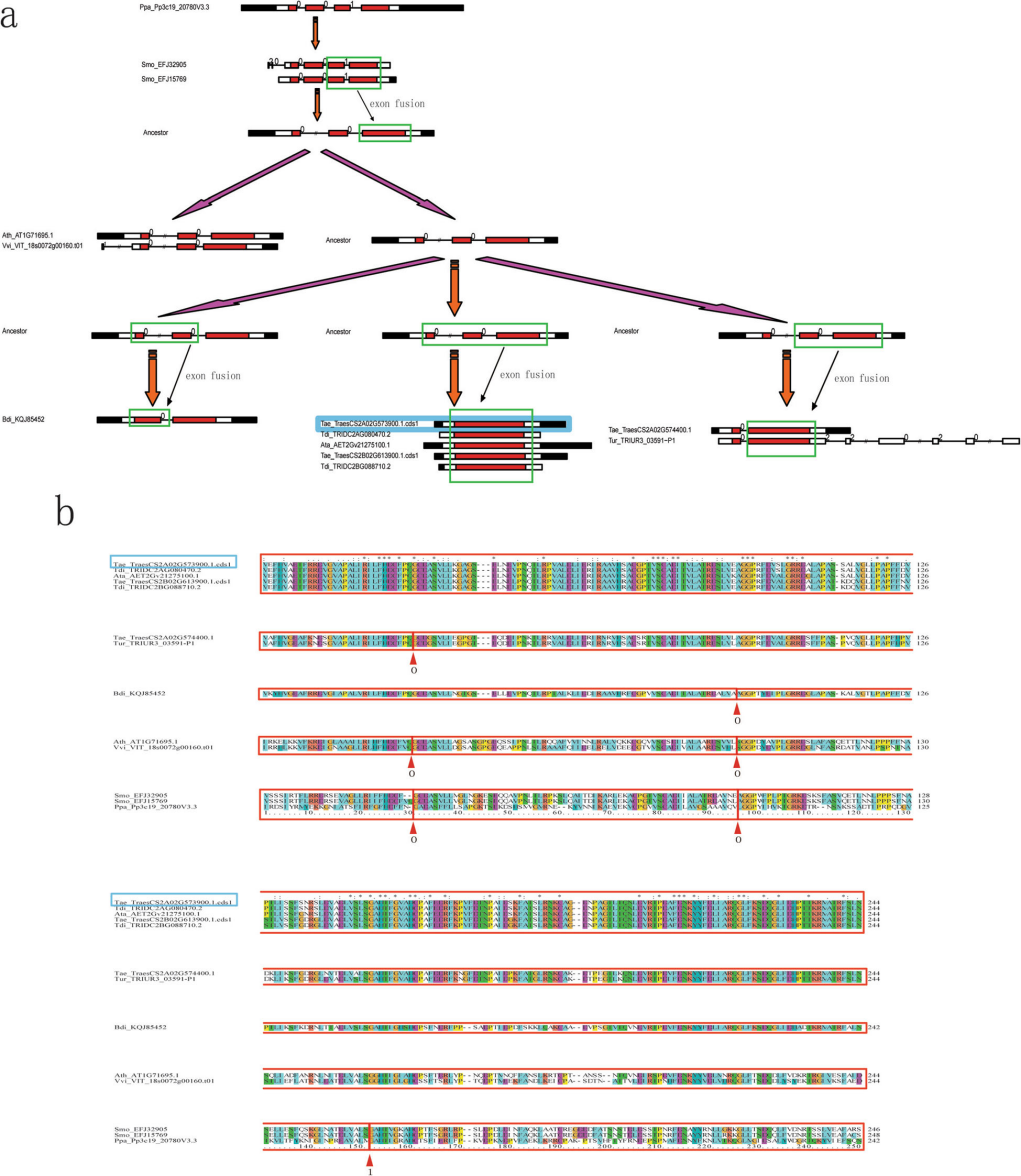
Evolutionary model of subfamily VI PRXs, including *TaPRX-2A*. **a** The exon fusion events of some subfamily VI PRXs in evolution. **b** The alignment of some subfamily VI PRXs. Exon phases are circled by red boxes and arrows. The PRX we investigated (TraesCS2A02G573900.1.cds1) in *T. aestivum* is circled by a cyan box

Based on the phylogenetic and exon-intron structure analysis (Additional file 5: Figure S2), we proposed an evolutionary model to determine the origin of *TaPRX*-2A (TraesCS2A02G573900.1.cds1), which was involved in the processes of exon fusion (Fig. 2a). This model suggests that two rounds of exon fusion events occurred during Angiosperm and Gramineae emergence. The first exon fusion event (four exons became three) occurred during Angiosperm emergence. An ancestral sequence resembling *P. patens* PRX (Pp3c19_20780V3.3) contained a conserved exon–intron structure within four exons and the “001” exon phases near the PRX domain. This four-exon structure within the “001” exon phase was retained in the ancestral sequences resembling two *S. moellendorffii* PRXs (Smo_EFJ32905 and Smo_EFJ15769). However, the exon–intron structures of PRXs in *A. thaliana* (Ath_AT1G71695.1)*, V. vinifera* (Vvi_VIT_18s0072g00160.t01), and *O. sativa* (Osa_Os04t0688200–01) changed into the three-exon structure within the “00” exon phases, suggesting the occurrence of an exon fusion event in the last two exons of the four-exon structures within the “001” exon phases before the monocot–eudicot evolutionary split. The second exon fusion event (three exons changed into two or one exon) occurred during Gramineae emergence. As shown in Fig. 2a, the first two exons in the three-exon structure within the “00” exon phases may have fused and changed into the two-exon structure within the “0” exon phase (*B. distachyon*, KQJ85452). Similarly, the last two exons could have fused (*T. aestivum,* TraesCS2A02G574400.1; *T. urartu,* TRIUR3_03591-P1) or all the three exons could have fused, thus merging into a single-exon structure (*Ae. tauschii,* Ata_AET2Gv21275100.1; *T. dicoccoides,* Tdi_TRIDC2AG080470.2, Tdi_TRIDC2BG088710.2; *T. aestivum,* TraesCS2A02G573900.1.cds1, Tae_TraesCS2B02G613900.1.cds1). The alignments of these PRXs within the breakpoints of exon fusion events supported our proposed evolutionary model (Fig. 2b).

To confirm these PRX sequences for the *TaPRX-2A* evolutionary model, we determined the cDNA-level evidences in RNA-sequencing (RNA-seq) data from seven plants (*P. patens*, *A. thaliana*, *V. vinifera*, *B. distachyon*, *Ae. tauschii*, *T. dicoccoides,* and *T. aestivum*) (Additional file 6: Table S4). We did not determine these evidences in *S. moellendorffii* and *T. urartu* because their GFF3 annotation files were just in scaffolds and not in chromosomes. The results showed that most PRX sequences (except VIT_18s0072g00160.t01 and TraesCS2A02G574400.1) from seven plants were detected in RNA-seq data (FPKM and coverage values in the “information” column of Additional file 6: Table S4), suggesting that the occurrence of exon fusion events during plant evolution.

We also detected positive selection of PRX domain sequences in *TaPRX-2A* and 12 other homologous PRXs using PAML 4.9 (Table 1). According to the likelihood ratio test of site-specific models, the M2a (selection) model was significantly higher than M1a (neutral) (degrees of freedom (df) = 2, 2ΔlnL = 68.4, *P* < 0.005), indicating that some amino acid sites underwent positive selection during evolution. The M7–M8 comparison (df = 2, 2ΔlnL = 7.47, *P* < 0.025) also supported the hypothesis of positive selection. These positively selected sites were identified using Naive Empirical Bayes and Bayes Empirical Bayes analyses (Additional file 7: Figure S3a,b). Two (95 E and 185 K, refer to sequence: Smo_EFJ32905) and six positively selected sites (95 E, 110 S, 117 Q, 135 E, 185 K, and 212 R) were found in the M2a and M8 models, respectively. Ancestral sequences in evolutionary nodes were also inferred by PAML 4.9 and MEGAX (Additional file 7: Figure S3).

**Table 1:** Detection of positive selection of *TaPRX-2A* and other 12 homologous PRX genes in plants

Models	np	Estimates of parameters	lnL	LRT pairs	df	2∆lnL	*P*
M0: one ratio	1	ω = 0.12224	− 5255.0007	M0 /M2	3	249.899394	< 0.005
M1a: neutral	2	p_0_ = 0.74332, (p_1_ = 0.25668), ω_0_ = 0.06658, (ω_1_ = 1.00)	− 5164.268639				
M2a: selection	4	p_0_ = 0.41411, p_1_ = 0.29296, (p_2_ = 0.29293), ω_0_ = 0.02081, (ω_1_ = 1.00), ω_2_ = 0.14800	− 5130.051003	M1/M2	2	68.435272	< 0.005
M7: beta	2	*p* = 0.53605, q = 2.52403	− 5119.765697	M7/M8	2	7.47398	< 0.025
M8: beta and ω	4	p_0_ = 0.66950, (p_1_ = 0.33050), *P* = 0.29052, q = 0.92947, ω = 0.09129	− 5116.028707				

Abbreviation, *np* Number of free parameters; *lnL* Log likelihood; *LRT* Likelihood ratio test; *df* Degrees of freedom; *2ΔlnL* Twice the log-likelihood difference of the models compared

### Expression patterns of *TaPRX-2A* in various tissues and stress treatments

**Fig. 3:**
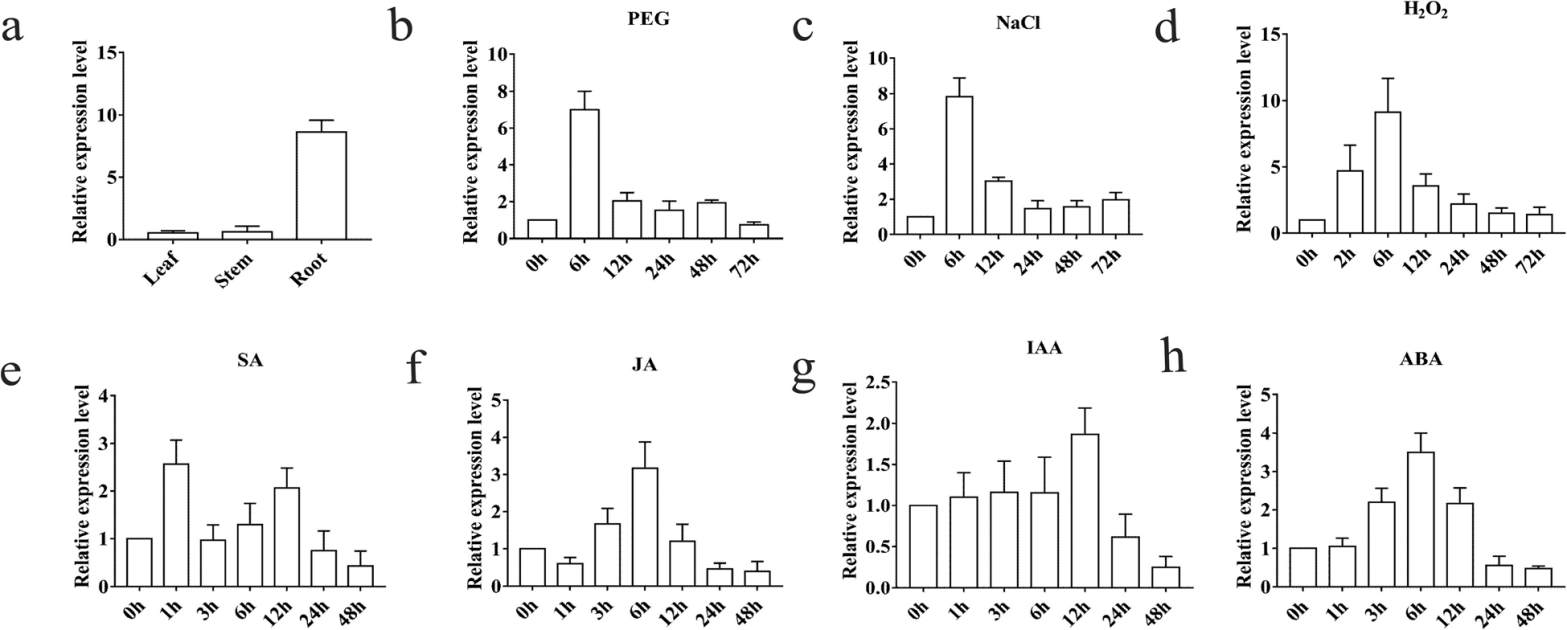
Transcript analysis of *TaPRX-2A*. **a** *TaPRX-2A* expression levels in the leaves, roots, and stems in normal conditions and under different stress treatments: (**b**) 20% (w/v) PEG 6000, (**c**) 200 mM NaCl, (**d**) 10 mM H_2_O_2_, (**e**) 2 mM SA, (**f**) 100 μM MeJA, (**g**) 100 μM IAA, and (**h**) 100 mM ABA. *18SrRNA* was used as an endogenous control. The gene relative expression was calculated by the cycle threshold (Ct) values using formula 2^–ΔΔCT^. Data are presented as mean ± standard deviation calculated from triplicates

To detect the expression patterns of *TaPRX-2A* in response to stress-related signaling*,* we performed qRT–PCR in different tissues (leaf, stem, and root) and with different stress treatments (PEG6000, NaCl, H_2_O_2_, SA, MeJA, IAA, and ABA). The results showed that *TaPRX-2A* was differentially expressed in the roots, stems, and leaves, with significantly higher expression levels in root tissues than in leaf and stem tissues (Fig. 3a). Then, we checked the expression patterns of *TaPRX-2A* by qRT–PCR in treatments of PEG 6000, NaCl, and H_2_O_2._ The results showed that the expressions of *TaPRX-2A* were induced by PEG 6000, NaCl, and H_2_O_2_ treatments and the expression levels reached a peak at 6 h after treatments (Fig. 3b, c, and d). We also examined the expression patterns of four phytohormones. As shown in Fig. 3e, *TaPRX-2A* exhibited approximately 2.5-fold upregulation at 1 h after SA treatment (Fig. 3e). Similarly, the expression levels of *TaPRX-2A* reached a peak at 6 h after JA and ABA treatments (Fig. 3f, h). However, the expression levels of *TaPRX-2A* remained relatively unchanged throughout 0–6 h after IAA treatment but exhibited an approximate 1.5-fold upregulation at 12 h (Fig. 3g). These results suggested the involvement of *TaPRX-2A* in various abiotic stress responses.

### Subcellular localization of the *TaPRX-2A* protein

To characterize the function of *TaPRX-2A*, the ORF of *TaPRX-2A* was fused to a pBIN35S-eGFP vector under the control of a CaMV 35S promoter (Additional file 8: Fig. S4a). The pBIN35S:eGFP empty vector control and the pBIN35S:*TaPRX-2A*:eGFP recombinant vector construct were transformed into tobacco leaf cells by *Agrobacterium* infiltration. We observed the epidermal cells of injected *N. benthamiana* leaves by confocal microscopy and found that *TaPRX-2A*:eGFP was mainly localized in the cell nuclei (Additional file 8: Fig. S4b_1_-d_2_). In addition, the pBIN35S-*TaPRX-2A*-eGFP and pBIN*35S*-eGFP vector were transformed into onion epidermal cells. Consistent with the localization results observed in tobacco epidermal cells, the *TaPRX-2A*:eGFP was also mainly localized in the nuclei of onion epidermal cells (Additional file 8: Fig. S4b_3_-d_4_). Moreover, the prediction of web server cNLS showed that five nuclear localization signal (NLS) sequences were present in *TaPRX-2A* (Additional file 9: Figure S5).

### *TaPRX-2A* enhanced salt tolerance in transgenic wheat

To further confirm the function of *TaPRX-2A* in responses against salt stress in plants, we transformed the wheat cultivar “KN199” with *TaPRX-2A* overexpression and constructed three independent transgenic lines (TaOE1, TaOE2, and TaOE3). The expression profile of *TaPRX-2A* was analyzed in *TaPRX-2A* transgenic lines through qRT–PCR. The results showed that the transgenic lines exhibited a higher expression level than wild-type (WT) plants (Additional file 10: Fig S6a). We subsequently measured the PRX activity in three independent transgenic lines and WT and found that the activity was higher in transgenic lines than in WT (Additional file 10: Fig. S6b). Taken together, we concluded that *TaPRX-2A* overexpression caused high PRX activity in transgenic lines.

**Fig. 4:**
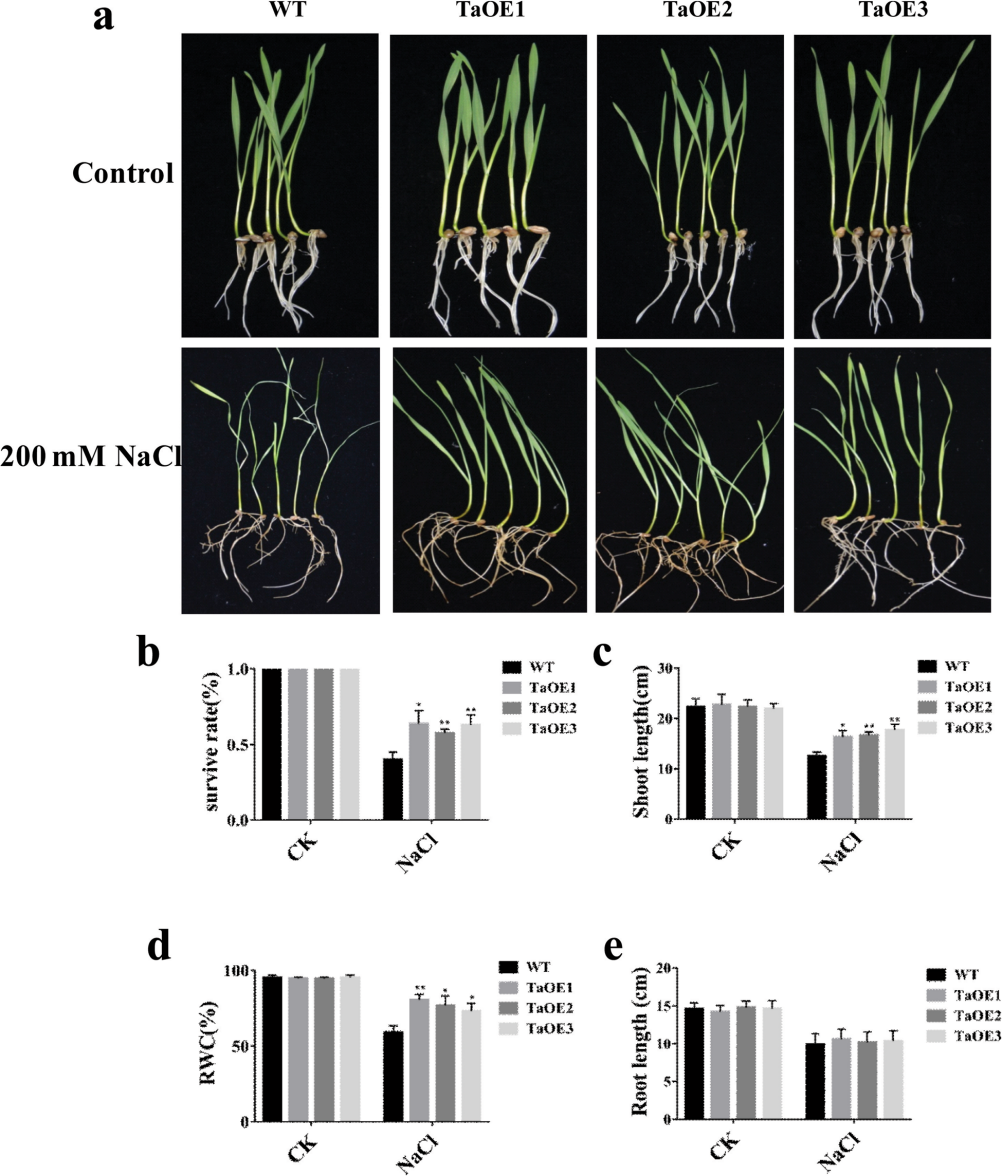
*TaPRX-2A* enhanced the salt tolerance of wheat. **a** Salt stress tolerance responses of *TaPRX-2A*-overexpressing transgenic and WT wheat (the cultivar “KN199”). (**b**) Survival rates, (**c**) shoot length, and (**d**) root length of *TaPRX-2A*-overexpressing and WT plants. (**e**) Relative water content of *TaPRX-2A*-overexpressing and WT plants. Data are presented as mean ± standard deviation calculated from triplicates. * and ** above each column indicate a significant difference compared with WT plants (*P < 0.05; **P < 0.01)

Then, we measured the phenotypic differences between transgenic lines (three independent lines (TaOE1, TaOE2, and TaOE3) and WT under salt stress conditions. Under non-stress conditions, no visibly phenotypic difference was observed between TaOE1–3 and WT. Under salt stress conditions, transgenic lines showed stronger growth compared with WT. In addition, the WT leaves turned yellow and wilted under salt stress, whereas the TaOE leaves still remained green (Fig. 4a). We also found that the survival rate of WT plants was only 40% after salt treatment, whereas the survival rates among TaOE1, TaOE2, and TaOE3 plants were 63.6, 57.6, and 63%, respectively (Fig. 4b). We then compared the shoot lengths, relative water content (RWC), and root lengths between WT and TaOE plants under salt treatment (Fig. 4c, d, and e). The results showed that transgenic lines exhibited longer shoot length and higher RWC than WT plants. However, no significant difference in root lengths was observed between WT and transgenic lines. Taken together, these results indicated that *TaPRX-2A* overexpression drastically enhanced salt tolerance in wheat.

**Fig. 5:**
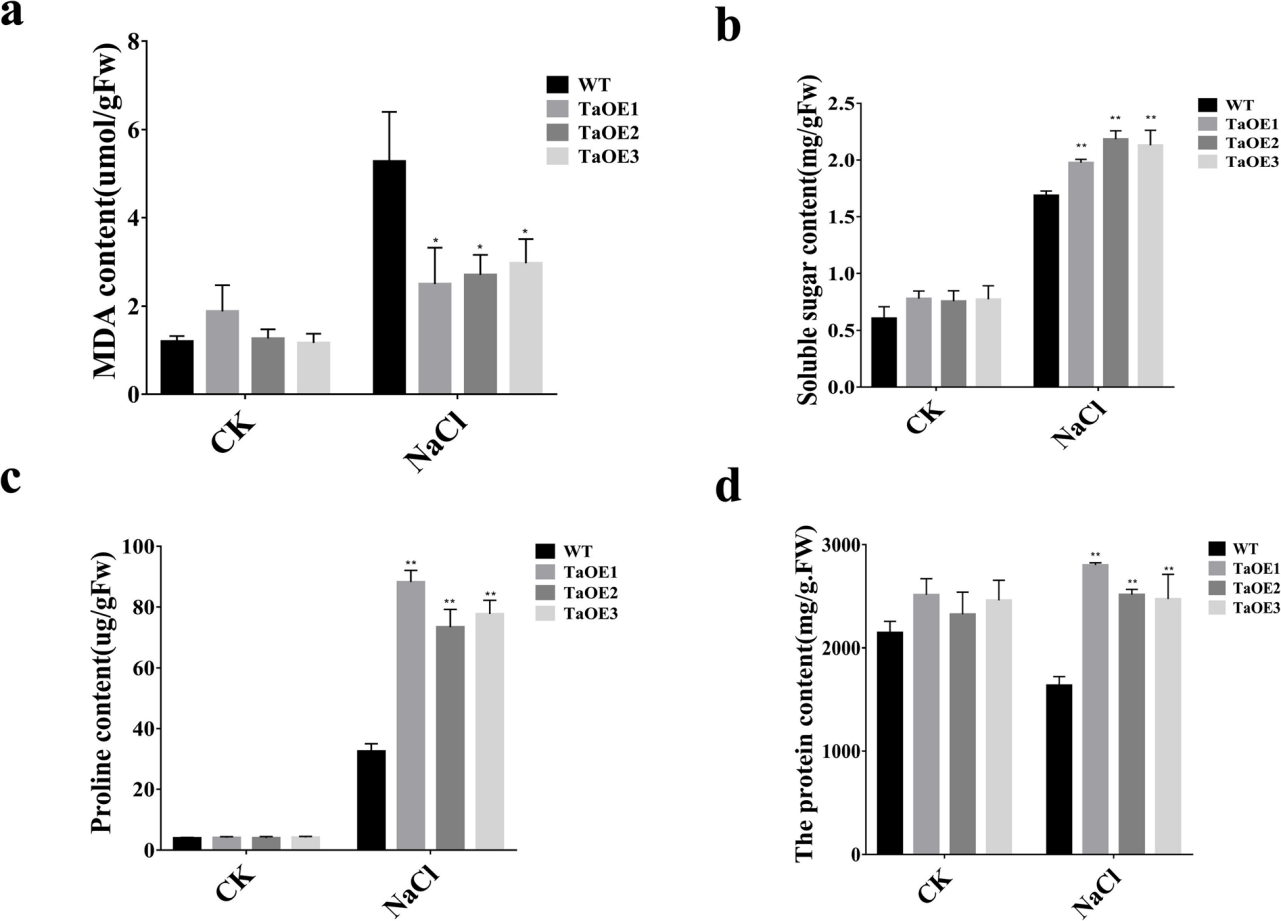
Physiological and biochemical indices between *TaPRX-2A*-overexpressing transgenic lines and WT plants. **a** MDA content, (**b**) soluble sugar content, (**c)** proline content, and (**d**) soluble protein content of *TaPRX-2A*-overexpressing and WT plants. Data are presented as mean ± standard deviation calculated from triplicates. * and ** above each column indicate a significant difference compared with WT plants (*P < 0.05; **P < 0.01)

To further explore mechanisms underlying *TaPRX-2A*-mediated response to salt stress, we measured physiological–biochemical indices between TaOE and WT plants under non-stress and salt stress conditions (Fig. 5a-d). Under salt treatment, TaOE lines contained significantly lower malondialdehyde (MDA) content than WT; they also contained higher soluble sugar, proline, and soluble protein contents. Moreover, the proline contents of transgenic lines were approximately 2-fold greater than those of the WT plants (Fig. 5c). These results suggested that overexpression of *TaPRX-2A* increased the contents of metabolites that were necessary for osmotic and oxidative stress tolerance in wheat cultivar “KN199”, thus resulting in improving tolerance to salt.

### *TaPRX-2A* regulates ROS scavenging and the expression of stress-related genes in transgenic wheat

**Fig. 6:**
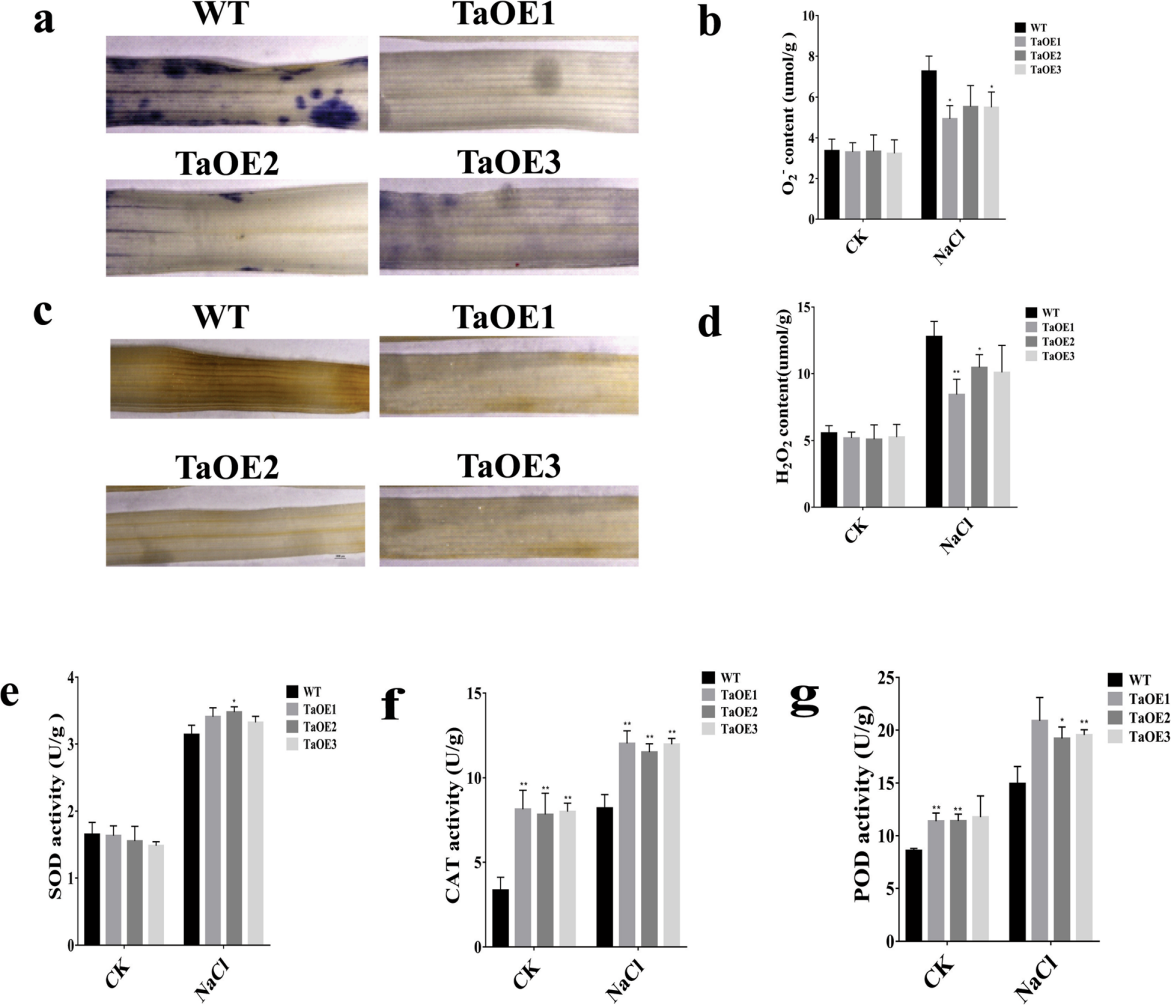
*TaPRX-2A* confers ROS-scavenging capacity by improving antioxidant enzymes activity. **a** Tissue localization of O_2_^−^ accumulation, (**b**) O_2_^−^ content, (**c)** tissue localization of H_2_O_2_ generation, (**d**) H_2_O_2_ content, (**e)** SOD activity, (**f**) CAT activity, and (**g**) POD activity of *TaPRX-2A*-overexpressing and WT plants. Data are presented as mean ± standard deviation calculated from triplicates. * and ** above each column indicate a significant difference compared with WT plants (*P < 0.05; **P < 0.01)

Previous studies have demonstrated that the tolerance to oxidative stress is associated with the physiological response of the plant to abiotic stresses [35, 37, 38]. Therefore, we examined the function of *TaPRX-2A* in reducing ROS levels in transgenic lines under salt stress. As major indicators of the ROS level, we assayed the accumulation of O_2_^−^ and H_2_O_2_ for comparison between TaOE and WT lines using nitroblue tetrazolium (NBT) and 3-diaminobenzidine (DAB) staining. Under salt treatment, we found that the levels of O_2_^−^ (stained blue with NBT) and H_2_O_2_ (stained brown by DAB) were significantly lower in transgenic lines than in WT plants (Fig. 6a-d). In addition, the activities of superoxide dismutase (SOD), peroxidase (POD), and catalase (CAT) antioxidant enzymes were higher in the transgenic plants than in the WT plants (Fig. 6e-g).

**Fig. 7:**
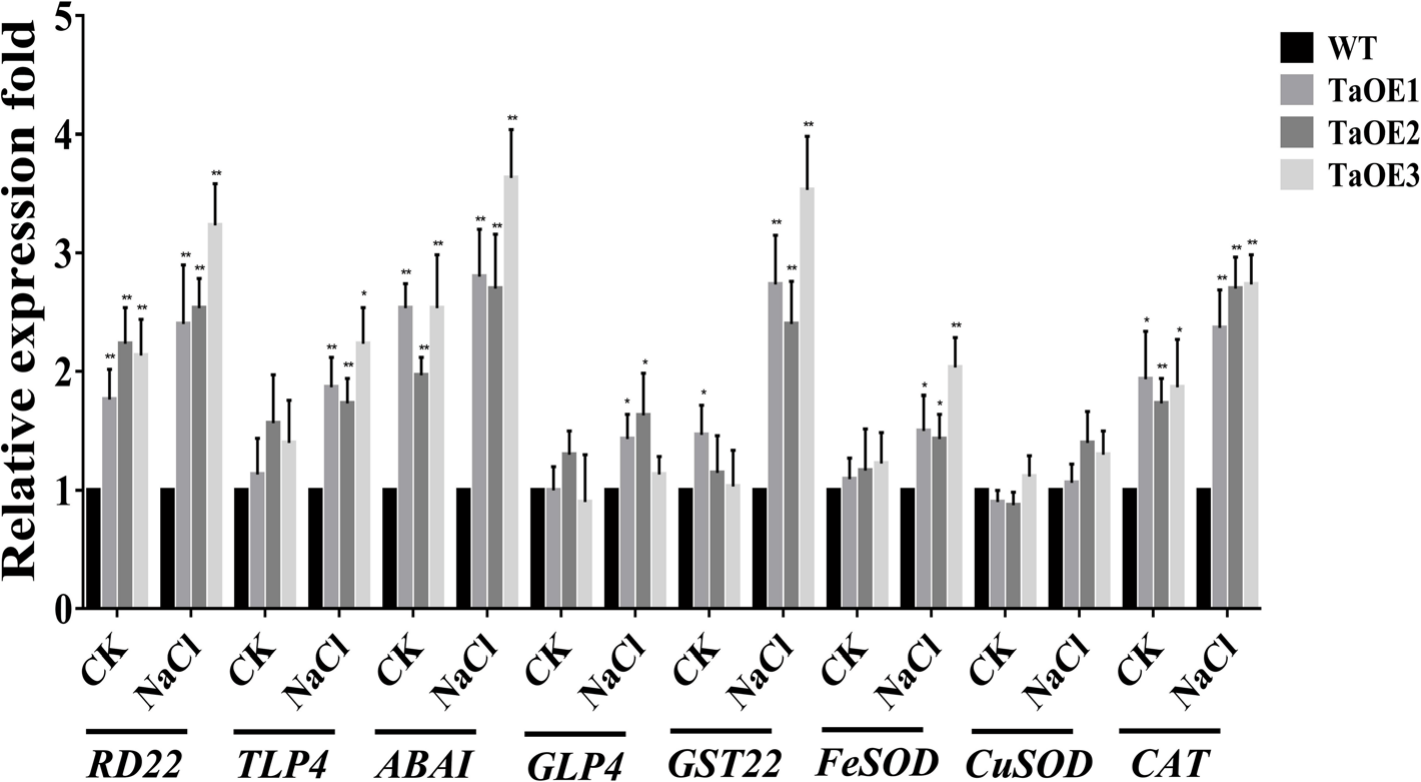
Analysis of the transcript profile of stress-related genes. Expression levels of stress-related genes in *TaPRX-2A*-overexpressing transgenic lines and WT plants under salt stress by qRT–PCR. The gene *18SrRNA* was as an endogenous control. Each treatment had three independent biological repeats. The gene relative expression was calculated by the cycle threshold (Ct) values using formula 2^–ΔΔCT^. The data are presented as mean ± standard deviation calculated from triplicates. * and ** above each column indicate a significant difference compared with WT plants (*P < 0.05; **P < 0.01)

To determine whether stress-responsive genes were associated with enhancing salt tolerance through *TaPRX-2A*, we determined the expression patterns of various stress-related genes in TaOE plants using qRT–PCR (Fig. 7). These stress-related genes (encoding dehydration-responsive protein, *RD22*; thaumatin-like protein, *TLP4*; ABA-inducing protein, *ABAI*; germin-like protein, *GLP4*; glutathione S-transferase, *GST22*; and the genes encoding the ROS-scavenging enzymes *FeSOD*, *CuSOD*, and *CAT*) were reported to be involved in the response to various abiotic stresses. Our results showed that the majority of these stress-related genes were more highly expressed in the TaOE lines than in the WT plants under salt stress, except *CuSOD* expression, which was not significantly different between the WT and transgenic lines. We also found that the expression of some stress-related genes, including *RD22*, *ABAI*, and *CAT*, was lower in the WT plants under non-stress conditions than in the transgenic plants*.* Taken together, these results suggested that *TaPRX-2A* overexpression may improve salt tolerance in wheat by enhancing the transcription levels of stress-responsive genes.

## Discussion

### Evolution of TaPRX-2A in *T. aestivum*

The objective of this study was to characterize the role of the wheat PRX gene *TaPRX-2A* in the plant’s response to salinity stress, in light of the severe reduction in crop yield associated with this form of abiotic stress [39]. Based on the classification of the NJ phylogenetic tree and the HMM scan, TraesCS2A02G573900.1.cds1 was found to belong to subfamily VI PRXs, which can be found in *S. moellendorffii* but not in *P. patens*, suggesting that subfamily VI PRXs have appeared in fern-resembling ancestors. Subfamily VI PRXs contain only one member in two investigated eudicots *A. thaliana* and *V. vinifera*, whereas subfamily VI PRXs contains various members of the investigated monocots (Additional file 5: Figure S2), suggesting that subfamily VI has experienced monocot-specific duplication events after the monocot–eudicot split.

Based on the analysis of exon–intron structures of the 12 plants investigated in this study, we proposed an evolutionary model involving two rounds of exon fusion events to infer the origin of *TaPRX*-2A (TraesCS2A02G573900.1.cds1) (Fig. 2). Among these exon fusion events, we focused on one of the second round of exon fusion events, wherein a three-exon structure changed into a one-exon structure before the *Triticum–Aegilops* split (formed into *TaPRX*-2A ancestor). The possible mechanism of this one-exon structure emergence might be “retroposition” (as a result of retrotransposition, the newly duplicated paralogs lack introns), which was reported in the origin of the gene *jingwei* in *Drosophila* species [40] and ATP synthase PGAM3 [41]. For instance, PGAM1 contains three introns, whereas PGAM3 has none. In plants, 69 retroposons and 1235 primary retrogenes were identified in *A. thaliana* and *O. sativa*, respectively [42, 43]. We also detected the positive selection among these 13 PRXs of evolutionary model by PAML4.9, and found 2 or 6 positively selected sites. It was also reported that 7 gene pairs among 24 retrogenes in *Oryza* species were identified to be under positive selection [44].

### *TaPRX-2A* enhanced the antioxidative stress ability and improved salt tolerance in wheat

In higher plants, class III PRXs comprise a large gene family, the members of which have been reported to participate in plant’s response to abiotic stresses [16]. For example, the class III PRX gene *OsPRX38* in *O. sativa* reportedly improved *Arabidopsis* arsenic (As) tolerance by activating the antioxidant system (SOD, PRX, and GST) and scavenging H_2_O_2_ [45]. In tobacco, it has been shown that the overexpression of the class III PRX gene *AtPrx64* in *A. thaliana* improved the plant’s aluminum (Al) tolerance by increasing root growth and scavenging the accumulation of Al and ROS [46]. In *A. thaliana,* overexpression of *AtPRX3* was shown to alleviate dehydration and increase salt tolerance. However, inhibition of *AtPRX3* expression decreased the tolerance to dehydration and salt [47]. Furthermore, the class III PRX genes *CrPrx1* and *CrPrx* in *Catharanthus roseus* have been reported to improve germination rate under salinity stress in *Nicotiana tabacum* [48]. Consistent with the results of these reports, our results also proved a positive regulator in the salt tolerance of wheat by *TaPRX-2A.*

Among the physiological issues that accompany abiotic stresses in plants, the excessive accumulation of ROS (particularly O_2_^−^ and H_2_O_2_) and high concentrations of ROS can damage cell membrane permeability and integrity as well as cell compartmentation [49–51]. The class III PRXs in plants can catalyze H_2_O_2_ reduction in the peroxidative cycle by transferring electrons from different donors [13, 16]. Previous studies have shown that class III PRXs can improve stress tolerance by regulating the ROS balance in plants. For example, *OsPRX38* improves *Arabidopsis* As tolerance by activating the antioxidant system and scavenging H_2_O_2_ [45]_._ *AtPrx64* improves the plant’s Al tolerance by scavenging ROS accumulation [46]. To maintain ROS balance through scavenging of free radicals, plants have evolved a complex antioxidative system to protect cells from damage [49, 51]. Notably, SOD, CAT, and PRX expression reportedly contributes to enhanced salt tolerance [9, 10]. In our study, we found that *TaPRX-2A* overexpression improved the antioxidant activity through CAT and PRX, thereby reducing ROS levels. In addition, the expression of antioxidant genes (*FeSOD*, *CuSOD*, and *CAT*) was altered in the *TaPRX-2A*-overexpressed transgenic plants compared with that observed in the WT plant, suggesting that *TaPRX-2A* regulates the expression of these genes, thereby affecting salt tolerance. Future research will explore the mechanisms through which *TaPRX-2A* regulates other antioxidant-encoding genes.

Interestingly, we found that *TaPRX-2A* was localized in the nucleus with NLSs. Some reports have shown that PRX genes such as *TaPRXs*, *AtGPX8*, and GPX in mammals and *LjGpx1* were located in the cell nuclei [52–56]. It was reported that a barely PRX possess a putative nuclear localization signal, which is located in the nucleus [57]. Previous studies have shown that ROS could cause DNA damage by activating endonucleases and damaging important biological macromolecules (such as nucleic acids) [5, 58, 59]. In *Arabidopsis, AtGPX8* is localized in the nucleus and can protect nuclear DNA from ROS damage by maintaining cellular redox homeostasis [53]. Based on the above evidence, we propose possible regulatory mechanisms: one explanation is that *TaPRX-2A* is expressly conserved for inhibiting ROS-mediated damage to genomic DNA in the nucleus and that other enzymes are responsible for scavenging ROS in or adjacent to organelles. Our second explanation is that *TaPRX-2A* is coexpressed with other ROS-scavenging enzymes and that its transcriptional upregulation leads to upregulation of enzymes modulating ROS levels outside the nucleus during exposure to salinity stress (Fig. 7).

### *TaPRX-2A’s* effects on salt tolerance via the ABA-dependent pathway

In plants, the ABA signaling pathway regulates various abiotic stress responses [60, 61]. For example, dehydrin and thaumatin-like proteins (*TLP*) genes, which are essential for tolerance to abiotic stresses, may be induced by ABA during stresses [62, 63]. In *Arabidopsis*, ABA reportedly mediates the transcriptional upregulation of the dehydration-responsive gene *RD22* [61]. Similar to *TaPRX-2A*, several class III PRX genes are reported to mediate tolerance to abiotic stresses via the ABA signaling pathway [64]. For example, the expression of *AtPRX3* in *A. thaliana* is induced by both salinity stress and exogenous ABA treatment [47]. Seven class III PRXs genes from *Tamarix hispida* are controlled by the ABA signaling pathway [65]. In our study, the expression of *TaPRX-2A* was highly upregulated by both NaCl and exogenous ABA treatments (Fig. 3). Moreover, we observed that the overexpression of *TaPRX-2A* in transgenic wheat led to the transcriptional upregulation of the stress-related genes *RD22*, *TLP4*, *ABAI*, *GLP4*, and *GST2*2 under salinity stress (Fig. 7). Our work demonstrated that *TaPRX-2A* enhances salt tolerance of wheat through activating the downstream stress-related genes and the ABA signaling pathway. Further study is warranted to explore the regulatory mechanisms through which *TaPRX-2A* regulates stress-related genes.

## Conclusions

In this study, we identified and characterized the role of the PRX gene *TaPRX-2A* in response to salinity stress in wheat. Phylogenetic analysis revealed the occurrence of some exon fusion events and positive selection during *TaPRX-2A* evolution. The overexpression of *TaPRX-2A* enhanced salt tolerance in transgenic wheat through activation of the ABA pathway and antioxidant enzymes, resulting in lower ROS accumulation and increased levels of osmotic metabolites. This work and its findings have strong future application value in the cultivation of salt-tolerant wheat varieties, which is especially relevant given the anticipated crop losses associated with the future impacts of climate change.

## Methods

### Isolation and cloning of *TaPRX-2A* and its transformation

Leaves of the harvest wheat cultivar “Sumai 3” were used to extract total RNA using TRIzol reagent (Transgen). cDNA was synthesized to amplify *TaPRX-2A.* The full*-*length cDNA sequence of *TaPRX-2A* (Genbank No. AJ878510.1) was obtained from NCBI (https://www.ncbi.nlm.nih.gov/), ligated into the PC186 vector, and transformed into “KN199” using particle gun-mediated gene transformation [66].

### Plant materials and abiotic treatments

Bread wheat (*T. aestivum* cultivars “KN199” and “Sumai 3”) seedlings were sourced from our laboratory (State Key Laboratory of Crop Biology, College of Agronomy, Shandong Agricultural University). The *TaPRX-2A*-overexpressing transgenic wheat lines and the WT “KN199” were grown at 20 °C–25 °C with a photoperiod of 16/8 h. When the plants grew to a period of one leaf and one heart, the transgenic plants and “KN199” were treated with 200 mM NaCl treatment. Concerning salt treatment, the control and transgenic seedlings were cultured in 200 mM NaCl solution for 10 days.

### Identification and classification of class III PRXs in wheat, *Ae. tauschii*, and other plants

The genomes and proteomes of 12 plants (*S. moellendorffii*, *Z. mays*, *B. distachyon*, *T. aestivum, Ae. tauschii*, *T. dicoccoides, V. vinifera, T. urartu, O. sativa*, *A. thaliana*, *P. patens* and *C. reinhardtii*) were downloaded from the Ensembl Plants 42 (http://plants.ensembl.org/) and analyzed. To identify PRXs, we scanned all the proteomes of the 12 plants in batch mode using our local server with Hmmer 3.1 (pfam profile PF00141.23, peroxidase.hmm, PRX domain). Then the pfam 32.0 website (http://pfam.xfam.org/) with an E value of 0.01 was used. Typical PRXs with a PRX domain covering > 50% alignment were retained and analyzed. Those covering < 50% of the PRX domain alignment were considered atypical PRXs and excluded from further analysis. PRX alignment of truncated sequences in the PRX domain was performed using ClustalW v2.0 [67]. We used the MEGA-CC 7.0 software to construct the NJ phylogenetic tree in our local server [68]. The PRX subfamily classification was performed using HMMER 3.1, and the models were generated based on maize PRX alignments [12].

The RNA-seq data of *P. patens* (SRR11434644, SRR11434645, and SRR11434646), *A. thaliana* (SRR11308184, SRR11308187, and SRR11308188), *V. vinifera* (SRR11249050, SRR11249059, and SRR11249060), *B. distachyon* (SRR10380965, SRR10380966, SRR10380967, and SRR10380968), *Ae. tauschii* (SRR9657462 and SRR9657463), and *T. dicoccoides* (SRR9657450 and SRR9657451) were downloaded from the NCBI SRA transcriptome database (https://www.ncbi.nlm.nih.gov/sra/). The RNA-seq data of *T. aestivum* (ERR1201797, ERR1201798, and ERR1201799) were downloaded from EBI ArrayExpress (https://www.ebi.ac.uk/arrayexpress/). Mapping of sample reads to the reference genome (the Ensembl Plants 42) was conducted using Hisat2 (version 2.2.0, https://daehwankimlab.github.io/hisat2/download/#version-hisat2-220). Conversion (sam to bam) and sorting were performed using Samtools (version 1.10, https://github.com/samtools/samtools/releases/). The transcripts were assembled using Stringtie (version 2.1.1, https://ccb.jhu.edu/software/stringtie/index.shtml).

### Domain and intron–exon structure diagram of PRXs

We used Perl and R scripts to generate PRXs intron–exon structures and domain diagrams for each of the 12 plants included in this study, based on the corresponding GFF file information from Ensembl Plants 42 (http://plants.ensembl.org/). The domain information of PRXs was batched from Pfam 32.0 (http://pfam.xfam.org/).

### Analysis of selective pressure

The truncated amino acid PRX domain sequences in *TaPRX-2A* and the other 12 homologous PRXs were aligned using Clustal X2. Based on the information of Pfam 32, the corresponding truncated cDNA of the PRX domain was generated from our Perl scripts. The codon alignment was generated by the web server PAL2NAL [69]. PAML 4.9 (CODEML) [70] and graphical interface PAMLX [71] were used to detect the selective pressure. Site-specific models M0 (one ratio), M1a (neutral), M2a (selection), M7 (beta), and M8 (beta & ω) were generated. Log likelihood (lnL) value of each model was calculated by CODEML. Models were compared using 2 Δ lnL = 2(lnL1 − lnL0) in accordance with the χ2 distribution with df. Ancestral sequences were inferred by CODEML (rst file of results) and MEGAX (using the ML method and JTT matrix-based model) [72].

### Expression pattern of *TaPRX-2A* in different abiotic stress treatments

The wheat leaf tissues of three-leaf stage seedlings were harvested at 0, 6, 12, 24, 48, and 72 h after 20% (w/v) PEG 6000 treatment and 200 mM NaCl treatment. The wheat leaf tissues were harvested at 0, 2, 6, 12, 24, 48, and 72 h after 10 mM H_2_O_2_ treatment. We harvested the wheat leaf tissues at 0, 1, 3, 6, 12, 24, and 48 h after treatments with 2 mM SA, 100 μM MeJA, 100 μM IAA, and 100 mM ABA. Total RNA of all harvested samples was extracted using TRIzol reagent (Invitrogen). First-strand cDNA synthesis and qRT–PCR were performed using the Roche LightCycler®480 system (Roche, Germany). The wheat gene *18SrRNA* was used as an endogenous control. Relative mRNA expressions were calculated using the 2^–ΔΔCT^ method. All qRT–PCR primers are summarized in Additional file 11: Table S5.

### Subcellular localization of the TaPRX-2A protein

According to the ORF of *TaPRX-2A*, we cloned this gene without a stop codon and constructed it into a pBIN*35S*-*eGFP* vector using the CaMV 35S promoter. Subsequently, pBIN*35S*-*TaPRX-2A*-*eGFP* and pBIN*35S*-*eGFP* (control) were transformed into *Agrobacterium* EHA105. The *Agrobacterium* EHA105 was resuspended in the suspension (10 mM MgCl_2_, 10 mM 4-morpholineethane-sulfonic acid hydrate at pH 5.6, 200 mM acetosyringone). The *Agrobacterium* suspension was adjusted to an optical density 600 value of 0.6, injected into tobacco leaves, and cultured for 3 days. The epidermal cells of the injected tobacco leaves were observed using a confocal microscope (Zeiss LSM880 Meta Confocal Microscope). In addition, we also transformed the pBIN*35S*-*TaPRX-2A*-*eGFP* and pBIN*35S*-*eGFP* vectors into onion epidermal cells through gene gun-mediated transformation [73]. The transformed epidermal cells were cultured in the dark at 28 °C for 8–12 h and observed using a confocal microscope. The NLS sequences of *TaPRX-2A* were predicted using the web server cNLS (http://nls-mapper.iab.keio.ac.jp/cgi-bin/NLS_Mapper_form.cgi) [74].

### Measurements of physiological–biochemical parameters

We collected the leaves of *TaPRX-2A*-overexpressed and “KN199” plants at 10 days after salt treatment. We used the formula to measure the leaf RWC: RWC = (FW − DW)/(TW − DW) × 100% where RWC is relative water content, FW is fresh weight, TW is turgid fresh weight, and DW is dry weight [75]. MDA content was measured using the thiobarbituric acid method [76]. Proline content was measured using the ninhydrin reaction method [77]. Soluble total sugars were determined by the anthrone method [78]. We used NBT and DAB staining to visualize O_2_^−^ and H_2_O_2_ levels, as reported previously [79, 80]. The SOD, CAT and PRX activity were detected using previously described methods [81–83].

## Supplementary information

**Additional file 1: Table S1.** The number of class III peroxidase gene family in 12 plants.

**Additional file 2: Table S2.** Subfamily classification of class III peroxidases in the investigated plant genomes.

**Additional file 3: Table S3.** List of atypical class III peroxidase in investigated plant genomes.

**Additional file 4: Figure S1.** Class III peroxidase phylogenetic tree. (a) Subfamily VI of class III peroxidases; (b) All subfamilies.

**Additional file 5: Figure S2.** Domain and exon-intron structure diagrams of class III peroxidase in *A. thaliana, V. vinifera, T. aestivum, P. patens, T. dicoccoides, T. urartu, Ae. tauschii, B. distachyon, C. reinhardtii, Z. mays, O. sativa* and *S. moellendorffii*. Filled boxes: red represents the PRX domain; white boxes represent the other exon regions; black boxes represent the untranslated regions (UTRs); lines represent the PRX introns; numbers 0, 1, and 2 represent the exon phases. The long introns are shortened by “//”.

**Additional file 6: Table S4.** The cDNA-level evidence performing by RNA-seq data from seven plants.

**Additional file 7: Figure S3.** Positively selected sites and inferred ancestral sequences. (a) by using M2a model of PAML. (b) by using M8 model of PAML. (c) by using MEGAX. Inferred positively selected sites were circled by red boxes in the alignment of 13 PRXs and 11 inferred ancestral sequences.

**Additional file 8: Figure S4.** Localization of *TaPRX-2A* was mainly in nucleus. (a) Vector construction diagrams of *pBIN35S:eGFP* and *pBIN35S:TaPRX-2A:eGFP*. (b_1_–d_2_) Subcellular localization of the *pBIN35S:TaPRX-2A:eGFP* fusion protein and *pBIN35S:eGFP* protein in tobacco epidermal cells. (b_3_–d_4_) Subcellular localization of the *pBIN35S:TaPRX-2A:eGFP* fusion protein and *pBIN35S:eGFP* protein in onion epidermal cells (b_1_–b_4_) Green fluorescent images; (c_1_–c_4_) Merged images of bright, green fluorescence; (d_1_–d_4_) Bright field images. Bars, 20 μm.

**Additional file 9: Figure S5.** The prediction of nuclear localization signals in *TaPRX-2A*.

**Additional file 10: Figure S6.** The expression profile and peroxidase activity measurement. (a) Expression analysis of *TaPRX-2A* in transgenic lines and WT by using *TaPRX-2A* gene. (b) The measurement of peroxidase activity in *TaPRX-2A* transgenic lines and WT. The gene *18SrRNA* was as an endogenous control. The gene relative expression was calculated by the cycle threshold (Ct) values using formula 2^–ΔΔCT^. The data are means ± SD calculated from three technical replicates. Asterisks, * and **, above each column indicate significant difference compared with WT plants (*P < 0.05; **P < 0.01).

**Additional file 11: Table S5.** Primers used for analysis.

## Data Availability

The genomes and proteomes of investigated plants are available in Ensembl Plants (http://plants.ensembl.org/). The accession numbers of investigated plants are *T. aestivum* (IWGSC), *Ae. tauschii* (Aet_v4.0), *A. thaliana* (TAIR10), *B. distachyon* (v3.0), *C. reinhardtii* (v5.5), *O. sativa* (IRGSP-1.0), *P. patens* (Phypa_V3), *S. moellendorffii* (v1.0), *T. dicoccoides* (WEWSeq_v.1.0), *T. urartu* (ASM34745v1), *V. vinifera* (12X), and *Z. mays* (B73_RefGen_v4). The nucleotid and amino acid sequence of T*aPRX-2A* is available at NCBI with accession number AJ878510.1 (https://www.ncbi.nlm.nih.gov/). The accession numbers of using RNA-seq data from NCBI SRA and EBI ArrayExpress were shown in Method section. The identification and exon-intron structures of PRXs in investigated plants are provided in supplementary files. The datasets used and/or analysed during the current study are available from the corresponding author on reasonable request.

## Abbreviations

WT: Wild type
PRXs: Peroxidases
SOD: Superoxide dismutase
POD: Peroxidase
CAT: Catalase
ROS: Reactive oxygen species
GPX: Glutathione peroxidase
APX: Ascorbate peroxidase
SA: Salicylic acid
MeJA: Methyljasmonic acid
IAA: Indole-3-acetic acid
ABA: Abscisic acid
RWC: Relative water content
NBT: Nitroblue tetrazolium
DAB: 3-Diaminobenzidine
GFP: Green fluorescent protein
qRT-PCR: Quantitative real-time PCR
PEG6000: Polyethylene glycol 6000

## References

[1] Mahajan S, Tuteja N (2005). Cold, salinity and drought stresses: an overview. Arch Biochem Biophys.

[2] Shinozaki K, Yamaguchi-Shinozaki K (2000). Molecular responses to dehydration and low temperature: differences and cross-talk between two stress signaling pathways. Curr Opin Plant Biol.

[3] Campo S, Baldrich P, Messeguer J, Lalanne E, Coca M, San SB (2014). Overexpression of a calcium-dependent protein kinase confers salt and drought tolerance in rice by preventing membrane lipid peroxidation. Plant Physiol.

[4] Abdel Latef AA, Kordrostam M, Zakir A, Zaki H, Saleh OM (2019). Eustress with H_2_O_2_ facilitates plant growth by improving tolerance to salt stress in two wheat cultivars. Plants.

[5] McCord JM (2000). The evolution of free radicals and oxidative stress. Am J Med.

[6] Roxas VP, Lodhi SA, Garrett DK, Mahan JR, Allen RD (2000). Stress tolerance in transgenic tobacco seedlings that overexpress glutathione S-transferase/glutathione peroxidase. Plant Cell Physiol..

[7] Zhai CZ, Zhao L, Yin LJ, Chen M, Wang QY, Li LC, Ma YZ (2013). Two wheat glutathione peroxidase genes whose products are located in chloroplasts improve salt and H_2_O_2_ tolerances in *Arabidopsis*. PLoS One.

[8] Mittler R (2002). Oxidative stress, antioxidants and stress tolerance. Trends Plant Sci.

[9] Abdel Latef AA, Mostofa MG, Rahman MM, Abdel Farid IB, Tran LSP (2019). Extracts from yeast and carrot roots enhance maize performance under seawater-induced salt stress by altering physio-biochemical characteristics of stressed plants. J Plant Growth Regul.

[10] Abdel Latef AA, Abu Alhmad MF, Kordrostami M, Abo-Baker AE, Zakir A. Inoculation with *Azospirillum lipoferum* or *Azotobacter chroococcum* reinforces maize growth by improving physiological activities under saline conditions. J Plant Growth Regul 2020. https://doi.org/10.1007/s00344-020-10065-9.

[11] Wood ZA, Schröder E, Robin Harris J, Poole LB. Structure, mechanism and regulation of peroxiredoxins. Trends Biochem Sci. 2003;28:32–40. 10.1016/s0968-0004(02)00003-8 12517450

[12] Wang Y, Wang Q, Zhao Y, Han G, Zhu S (2015). Systematic analysis of maize class III peroxidase gene family reveals a conserved subfamily involved in abiotic stress response. Gene.

[13] Hiraga S, Sasaki K, Ito H, Ohashi Y, Matsui H (2001). A large family of class III plant peroxidases. Plant Cell Physiol.

[14] Welinder KG (1992). Superfamily of plant, fungal and bacterial peroxidases. Curr Opin Struct Biol.

[15] Taurog A (1999). Molecular evolution of thyroid peroxidase. Biochimie.

[16] Cosio C, Dunand C (2009). Specific functions of individual class III peroxidase genes. J Exp Bot.

[17] Shigeoka S, Ishikawa T, Tamoi M, Miyagawa Y, Takeda T, Yabuta Y, Yoshimura K (2002). Regulation and function of ascorbate peroxidase isoenzymes. J Exp Bot.

[18] Erman JE, Vitello LB (2002). Yeast cytochrome c peroxidase: mechanistic studies via protein engineering. Biochim Biophys Acta Protein Struct Mol Enzymol.

[19] Martinez AT, Speranza M, Ruiz-Duenas FJ, Ferreira P, Camarero S, Guillen F, Martinez MJ, Gutierrez A, del Río JC (2005). Biodegradation of lignocellulosics: microbial, chemical, and enzymatic aspects of the fungal attack of lignin. Int Microbiol.

[20] Almagro L, Gómez LV, Belchi-Navarro S, Bru R, Ros Barceló A, Pedreno MA (2009). Class III peroxidases in plant defence reactions. J Exp Bot.

[21] Csiszár J, Gallé Á, Horváth E, Dancsó P, Gombos M, Váry Z, Tari I (2012). Different peroxidase activities and expression of abiotic stress-related peroxidases in apical root segments of wheat genotypes with different drought stress tolerance under osmotic stress. Plant Physiol Biochem.

[22] Passardi F, Longet D, Penel C, Dunand C (2004). The class III peroxidase multigenic family in rice and its evolution in land plants. Phytochemistry.

[23] Welinder KG, Justesen AF, Kjaersgard IV, Jensen RB, Rasmussen SK, Jespersen HM, Duroux L (2002). Structural diversity and transcription of class III peroxidases from *Arabidopsis thaliana*. Eur J Biochem.

[24] Ren LL, Liu YJ, Liu HJ, Qian TT, Qi LW, Wang XR, Zeng QY (2014). Subcellular relocalization and positive selection play key roles in the retention of duplicate genes of *Populus* class III peroxidase family. Plant Cell.

[25] De GL. Class III peroxidases and ascorbate metabolism in plants. Phytochem Rev 2004;3:195–205.

[26] Herrero J, Fernández-Pérez F, Yebra T, Novo-Uzal E, Pomar F, Pedreño MÁ, Cuello J, Guéra A, Esteban-Carrasco A, Zapata JM (2013). Bioinformatic and functional characterization of the basic peroxidase 72 from *Arabidopsis thaliana* involved in lignin biosynthesis. Planta.

[27] Cheong YH, Chang HS, Gupta R, Wang X, Zhu T, Luan S (2002). Transcriptional profiling reveals novel interactions between wounding, pathogen, abiotic stress, and hormonal responses in *Arabidopsis*. Plant Physiol.

[28] Chassot N, Nawrath C, Métraux JP (2007). Cuticular defects lead to full immunity to a major plant pathogen. Plant J.

[29] Mei WQ, Qin YM, Song WQ, Li J, Zhu YX (2009). Cotton *GhPOX1* encoding plant class III peroxidase may be responsible for the high level of reactive oxygen species production that is related to cotton fiber elongation. J Genet Genomics.

[30] Simonetti E, Veronico P, Melillo MT, Delibes Á, Andrés MF, López-Braña I (2009). Analysis of class III peroxidase genes expressed in roots of resistant and susceptible wheat lines infected by *Heterodera avenae*. Mol Plant-Microbe Interact.

[31] Shigeto M, Hironori K, Takehiro M, Kunisuke T. Induction of Rice Cytosolic Ascorbate Peroxidase mRNA by Oxidative Stress; the Involvement of Hydrogen Peroxide in Oxidative Stress Signalling. Plant Cell Physiol. 1999;40:417–422.

[32] Miao Y, Lv D, Wang P, Wang XC, Chen J, Miao C, Song CP (2006). An *Arabidopsis* glutathione peroxidase functions as both a redox transducer and a scavenger in abscisic acid and drought stress responses. Plant Cell.

[33] Zhou Y, Hu LF, Ye SF, Jiang LW, Liu SQ (2018). Genome-wide identification of glutathione peroxidase (GPX) gene family and their response to abiotic stress in *Cucumber*. 3. Biotech.

[34] Sečenji M, Lendvai Á, Miskolczi P, Kocsy G, Gallé Á, Szűcs A, Hoffmann B. Sa’rva’ri E, Schweizer P, stein N, Dudits D, Gyorgyey J. Differences in root functions during long-term drought adaptation: comparison of active gene sets of two wheat genotypes. Plant Biol. 2010;12:871–82. 10.1111/j.1438-8677.2009.00295.x 21040302

[35] Dong W, Wang MC, Xu F, Quan TY, Peng KQ, Xiao LT, Xia GM (2013). Wheat oxophytodienoate reductase gene *TaOPR1* confers salinity tolerance via enhancement of abscisic acid signaling and reactive oxygen species scavenging. Plant Physiol.

[36] Zhang M, Lv DW, Ge P, Bian YW, Chen GX, Zhu GR, Li XH, Yan YM (2014). Phosphoproteome analysis reveals new drought response and defense mechanisms of seedling leaves in bread wheat (*Triticum aestivum* L.). J. Proteomics.

[37] Hu LX, Li HY, Pang HC, Fu JM (2012). Responses of antioxidant gene, protein and enzymes to salinity stress in two genotypes of perennial ryegrass (*Lolium perenne*) differing in salt tolerance. J Plant Physiol.

[38] Mittler P, Vanderauwera S, Suzuki N, Miller G, Tognetti VB, Vandepoele K, Gollery M, Shulaev V, Breusegem FV (2011). ROS signaling: the new wave?. Trends Plant Sci.

[39] You J, Chan ZL (2015). ROS regulation during abiotic stress responses in crop plants. Front Plant Sci.

[40] Long M, Langley C (1993). Natural selection and the origin of jingwei, a chimeric processed functional gene in *Drosophila*. Science.

[41] Long MY, Deutsch M, Wang W, Betrán E, Brunet FG, Zhang JM (2003). Origin of new genes: evidence from experimental and computational analyses. Genetica..

[42] Zhang Y, Wu Y, Liu Y, Han B (2005). Computational identifcation of 69 retroposons in *Arabidopsis*. Plant Physiol.

[43] Wang W, Zheng HK, Fan CZ, Li J, Shi JJ, Cai ZQ, Zhang GJ, Liu DY, Zhang JG, Vang SR, Lu ZK, Wong G, Long MY, Wang J (2006). High rate of chimeric gene origination by retroposition in plant genomes. Plant Cell.

[44] Zhou YL, Zhang CJ (2019). Evolutionary patterns of chimeric retrogenes in *Oryza* species. Sci Rep.

[45] Maria K, Yogeshwar VD, Neelam G, Madhu T, Iffat ZA, Mehar HA, Debasis C (2019). *Oryza sativa* class III peroxidase (*Osprx38*) overexpression in *Arabidopsis thaliana* reduces arsenic accumulation due to apoplastic lignification. J Hazard Mater.

[46] Wu YS, Yang ZL, Xu HN HJY, Chen LM, Li KZ (2017). Overexpression of a peroxidase gene (*AtPrx64*) of *Arabidopsis thaliana* in tobacco improves plant’s tolerance to aluminum stress. Plant Mol Biol.

[47] Llorente F, Lopez-Cobollo RM, Catala R, Martinez-Zapater JM, Salinas J (2002). A novel cold-inducible gene from *Arabidopsis, RCI3*, encodes a peroxidase that constitutes a component for stress tolerance. Plant J.

[48] Kumar S, Jaggi M, Sinha AK (2012). Ectopic overexpression of vacuolar and apoplastic *Catharanthus roseus* peroxidases confers differential tolerance to salt and dehydration stress in transgenic tobacco. Protoplasma.

[49] Dat J, Vandenabeele S, Vranová E, Van MM, Inzé D, Van BF (2000). Dual action of the active oxygen species during plant stress responses. Cell Mol Life Sci.

[50] Baxter A, Mittler R, Suzuki NN (2013). ROS as key players in plant stress signaling. J Exp Bot.

[51] Gill SS, Tuteja N (2010). Reactive oxygen species and antioxidant machinery in abiotic stress tolerance in crop plants. Plant Physiol Biochem.

[52] Yan J, Su PS, Li W, Xiao GL, Zhao Y, Ma X, Wang HW, Nevo E, Kong LR (2019). Genome-wide and evolutionary analysis of the class III peroxidase gene family in wheat and *Aegilops tauschii* reveals that some members are involved in stress responses. BMC Genomics.

[53] Ahmed G, Tomoya O, Takanori M, Kazuya Y, Masahiro T, Shigeru S (2012). The involvement of *Arabidopsis* glutathione peroxidase 8 in the suppression of oxidative damage in the nucleus and cytosol. Plant Cell Physiol.

[54] Del Maestro R, McDonald W (1989). Subcellular localization of superoxide dismutases, glutathione peroxidase and catalase in developing rat cerebral cortex. Mech Ageing Dev.

[55] Rogers LK, Gupta S, Welty SE, Hansen TN, Smith CV (2002). Nuclear and nucleolar glutathione reductase, peroxidase, and transferase activities in livers of male and female Fischer-344 rats. Toxicol Sci.

[56] Matamoros MA, Saiz A, Peñuelas M, Bustos-Sanmamed P, Mulet JM, Barja MV, Rouhier N, Moore M, James EK, Dietz KJ, Becana M (2015). Function of glutathione peroxidases in legume root nodules. J Exp Bot.

[57] Stacy RA, Nordeng TW, Culianez-Macia FA, Aalen RB (1999). The dormancy-related peroxiredoxin anti-oxidant, PER1, is localized to the nucleus of barley embryo and aleurone cells. J Plant.

[58] Hagar H, Ueda N, Shah SV (1996). Role of reactive oxygen metabolites in DNA damage and cell death in chemical hypoxic injury to LLC-PK1 cells. Amer J Physiol.

[59] Chen SX, Schopfer P (1999). Hydroxyl radical production in physiological reactions: a novel function of peroxidase. Eur J Biochem.

[60] Chinnusamy V, Gong Z, Zhu JK (2008). Abscisic acid-mediated epigenetic processes in plant development and stress responses. J Integr Plant Biol.

[61] Shinozaki K, Yamaguchi-Shinozaki K, Seki M (2003). Regulatory network of gene expression in the drought and cold stress responses. Curr Opin Plant Biol.

[62] Seo PJ, Park CM (2016). MYB96-mediated abscisic acid signals induce pathogen resistance response by promoting salicylic acid biosynthesis in *Arabidopsis*. New Phytol.

[63] Jung YC, Lee HJ, Yum SS, Soh WY, Cho DY, Auh CK, Lee TK, Soh HC, Kim YS, Lee SC (2005). Drought-inducible-but ABA-independent-thaumatin-like protein from carrot (*Daucus carota L.*). Plant Cell Rep.

[64] Roberts E, Kolattukudy PE (1989). Molecular cloning, nucleotide sequence, and abscisic acid induction of a suberization-associated highly anionic peroxidase. Mol Gen Genom.

[65] Gao CQ, Wang YC, Liu GF, Wang C, Jiang J, Yang CP (2010). Cloning of ten peroxidase (POD) genes from *Tamarix Hispida* and characterization of their responses to abiotic stress. Plant Mol Biol Rep.

[66] Yao Q, Cong L, Chang JL, Li KX, Yang GX, He GY (2006). Low copy number gene transfer and stable expression in a commercial wheat cultivar via particle bombardment. J Exp Bot.

[67] Larkin MA, Blackshields G, Brown NP, Chenna R, McGettigan PA, McWilliam H, Valentin F, Wallace IM, Wilm A, Lopez R (2007). Clustal W and Clustal X version 2.0. Bioinformatics.

[68] Kumar S, Stecher G, Tamura K (2016). MEGA7: molecular evolutionary genetics analysis version 7.0 for bigger datasets. Mol. Biol. Evol..

[69] Suyama M, Torrents D, Bork P (2006). PAL2NAL: robust conversion of protein sequence alignments into the corresponding codon alignments. Nucleic Acids Res.

[70] Yang Z. PAML 4: a program package for phylogenetic analysis by maximum likelihood. Mol Biol Evol. 2007;24:1586–91. 10.1093/molbev/msm088 17483113

[71] Xu B, Zhang ZH. pamlX: a graphical user interface for PAML. Mol Biol Evol. 2013;30:2723–4. 10.1093/molbev/mst179 24105918

[72] Kumar S, Stecher G, Li M, Knyaz C, Tamura K. MEGA X: Molecular evolutionary genetics analysis across computing platforms. Mol Biol Evol. 2018;35:1547–9. 10.1093/molbev/msy096 PMC5967553 29722887

[73] Das P, Ito T, Wellmer F, Vernoux T, Dedieu A, Traas J, Meyerowitz EM. Floral stem cell termination involves the direct regulation of agamous by perianthia. Development. 2009;136:1605–11. 10.1242/dev.035436 19395638

[74] Kosugi S, Hasebe M, Tomita M, and Yanagawa H. Systematic identification of yeast cell cycle-dependent nucleocytoplasmic shuttling proteins by prediction of composite motifs. Proc Natl Acad Sci. 2009;106:10171–6. 10.1073/pnas.0900604106 PMC2695404 19520826

[75] Zhou SM, Sun XD, Yin SH, Kong XZ, Zhou S, Xu Y, Wang W. The role of the F-box gene TaFBA1 from wheat (*Triticum aestivum* L.) in drought tolerance. Plant Physiol. Biochem. 2014;84:213–23. 10.1016/j.plaphy.2014.09.017 25299612

[76] Heath R, Packer L. Photoperoxidation in isolated chloroplasts: I. Kinetics and stoichiometry of fatty acid peroxidation. Arch Biochem Biophys. 1968;125:189–98. 10.1016/0003-9861(68)90654-1 5655425

[77] Bates LS, Waldren PR, Teare ID. Rapid determination of free proline for water-stress studies. Plant Soil. 1973;39:205–7.

[78] Spiro RG. Analysis of sugars found in glycoprotein. Method Enzymol. 1966;8:3–26.

[79] Gay C, Collins J, Gebicki JM. Hydroperoxide assay with the ferric-xylenol orange complex. Anal. Biochem. 1999;273:149–55. 10.1006/abio.1999.4208 10469484

[80] Tian F, Gong J, Zhang J, Zhang M, Wang G, Li A, Wang W. Enhanced stability of thylakoid membrane proteins and antioxidant competence contribute to drought stress resistance in the tasg1 wheat stay-green mutant. J. Exp. Bot. 2013;64:1509–20. 10.1093/jxb/ert004 PMC3617820 23378376

[81] Dhindsa RA, Plumb-Dhindsa P, Thorpe TA. Leaf senescence: correlated with increased permeability and lipid peroxidation, and decreased levels of superoxide dismutase and catalase. J. Exp. Bot. 1981;126:93–101.

[82] Aebi H. Catalase in vitro. Methods Enzymol. 1984;105:121–6. 10.1016/s0076-6879(84)05016-3 6727660

[83] Chance B, Maehly A. Assay of catalases and peroxidases. Methods Enzymol. 1955;2:764–75. 10.1002/9780470110171.ch14 13193536

