# Supplementary figures and images for: A member of wheat class III peroxidase gene family, TaPRX-2A, enhanced the tolerance of salt stress
Source: BMC Plant Biol. 2020 Aug 26;20:392. doi: 10.1186/s12870-020-02602-1 (PMC7449071; doi:10.1186/s12870-020-02602-1)

a

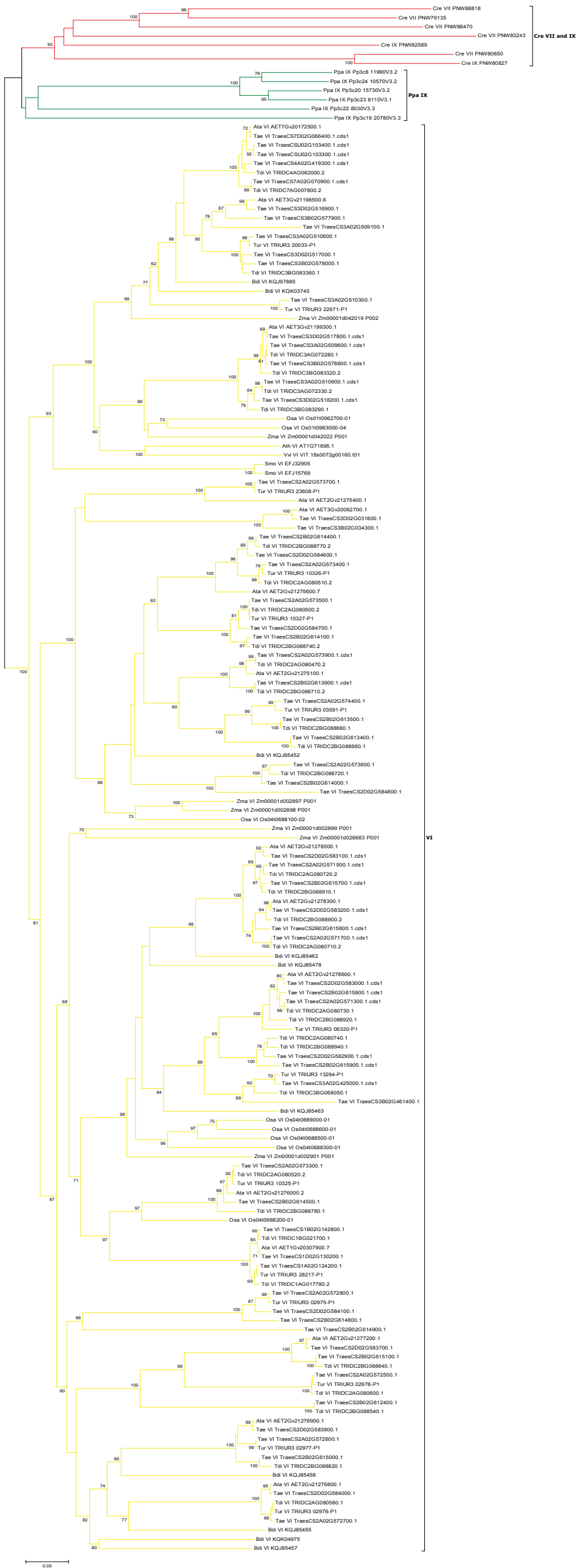

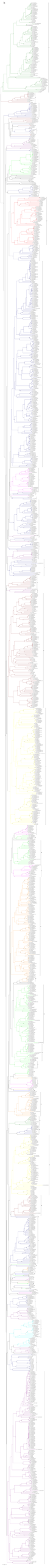

Supplement: Supplementary file 4 — Additional file 4: Figure S1. Class III peroxidase phylogenetic tree. (a) Subfamily VI of class III peroxidases; (b) All subfamilies. [file 12870_2020_2602_MOESM4_ESM.pdf]

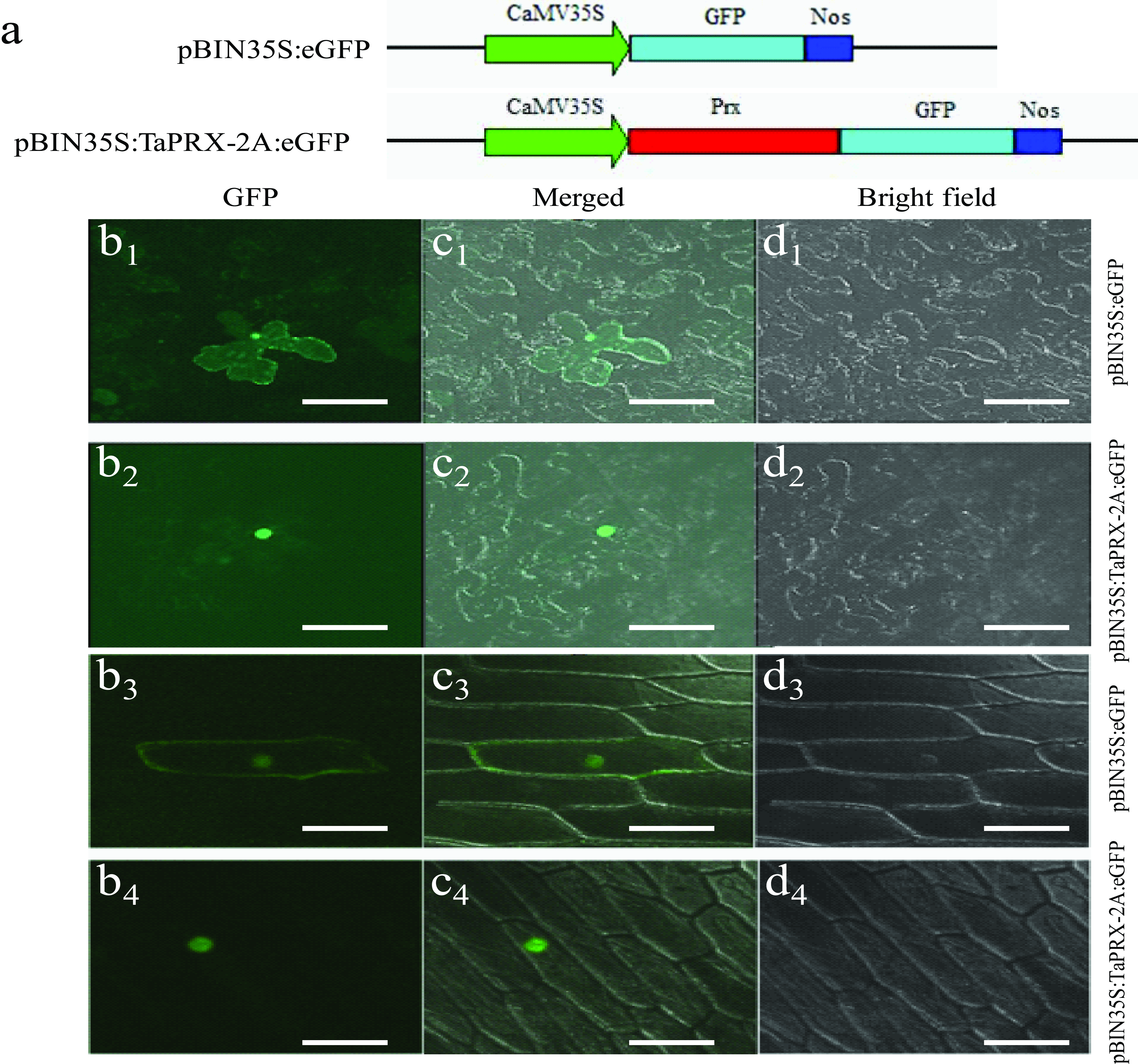

Supplement: Supplementary file 8 — Additional file 8: Figure S4. Localization of TaPRX-2A was mainly in nucleus. (a) Vector construction diagrams of pBIN35S:eGFP and pBIN35S:TaPRX-2A:eGFP. (b1–d2) Subcellular localization of the pBIN35S:TaPRX-2A:eGFP fusion protein and pBIN35S:eGFP protein in tobacco epidermal cells. (b3–d4) Subcellular localization of the pBIN35S:TaPRX-2A:eGFP fusion protein and pBIN35S:eGFP protein in onion epidermal cells (b1–b4) Green fluorescent images; (c1–c4) Merged images of bright, green fluorescence; (d1–d4) Bright field images. Bars, 20 μm. [file 12870_2020_2602_MOESM8_ESM.jpg]

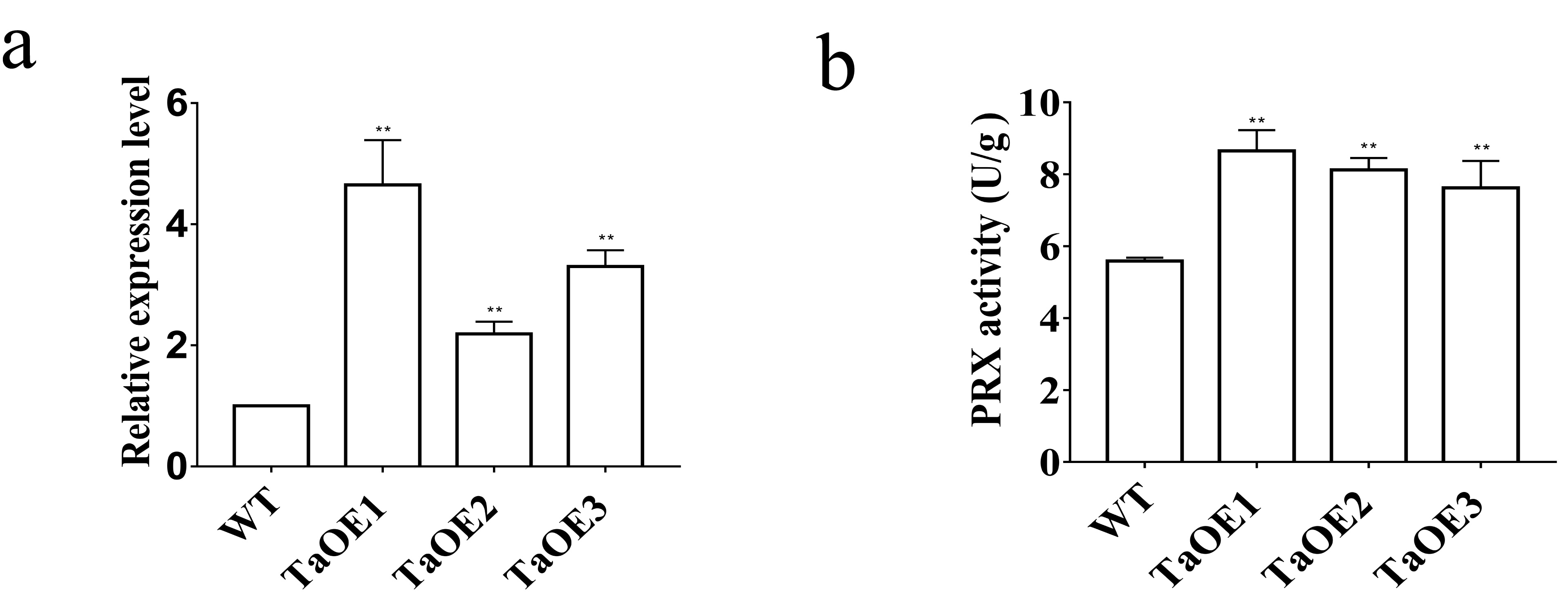

Supplement: Supplementary file 10 — Additional file 10: Figure S6. The expression profile and peroxidase activity measurement. (a) Expression analysis of TaPRX-2A in transgenic lines and WT by using TaPRX-2A gene. (b) The measurement of peroxidase activity in TaPRX-2A transgenic lines and WT. The gene 18SrRNA was as an endogenous control. The gene relative expression was calculated by the cycle threshold (Ct) values using formula 2–ΔΔCT. The data are means ± SD calculated from three technical replicates. Asterisks, * and **, above each column indicate significant difference compared with WT plants (*P < 0.05; **P < 0.01). [file 12870_2020_2602_MOESM10_ESM.jpg]
