# Supplementary material for: A member of wheat class III peroxidase gene family, TaPRX-2A, enhanced the tolerance of salt stress
Source: BMC Plant Biol. 2020 Aug 26;20:392. doi: 10.1186/s12870-020-02602-1 (PMC7449071; doi:10.1186/s12870-020-02602-1)

T.ae class III peroxidase I subfamily exon−intron and prx domain diagram (part 1)

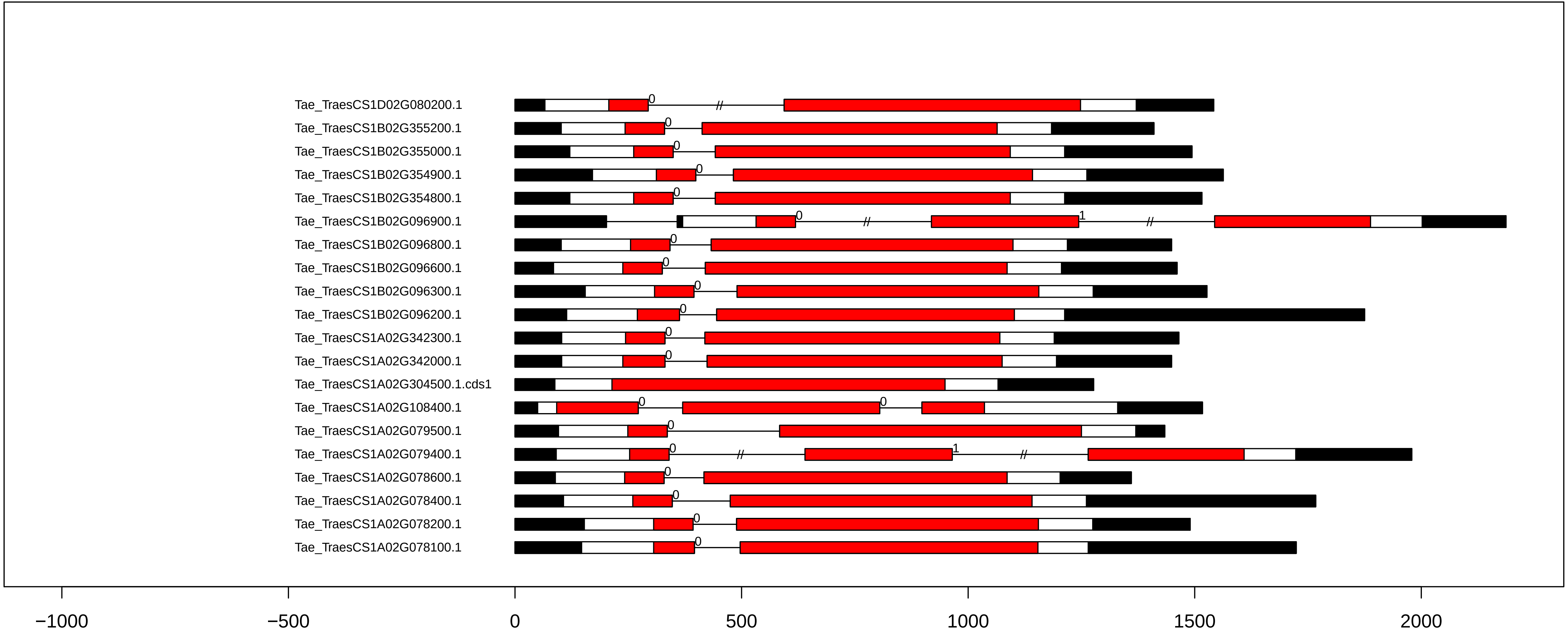

T.ae class III peroxidase I subfamily exon−intron and prx domain diagram (part 2)

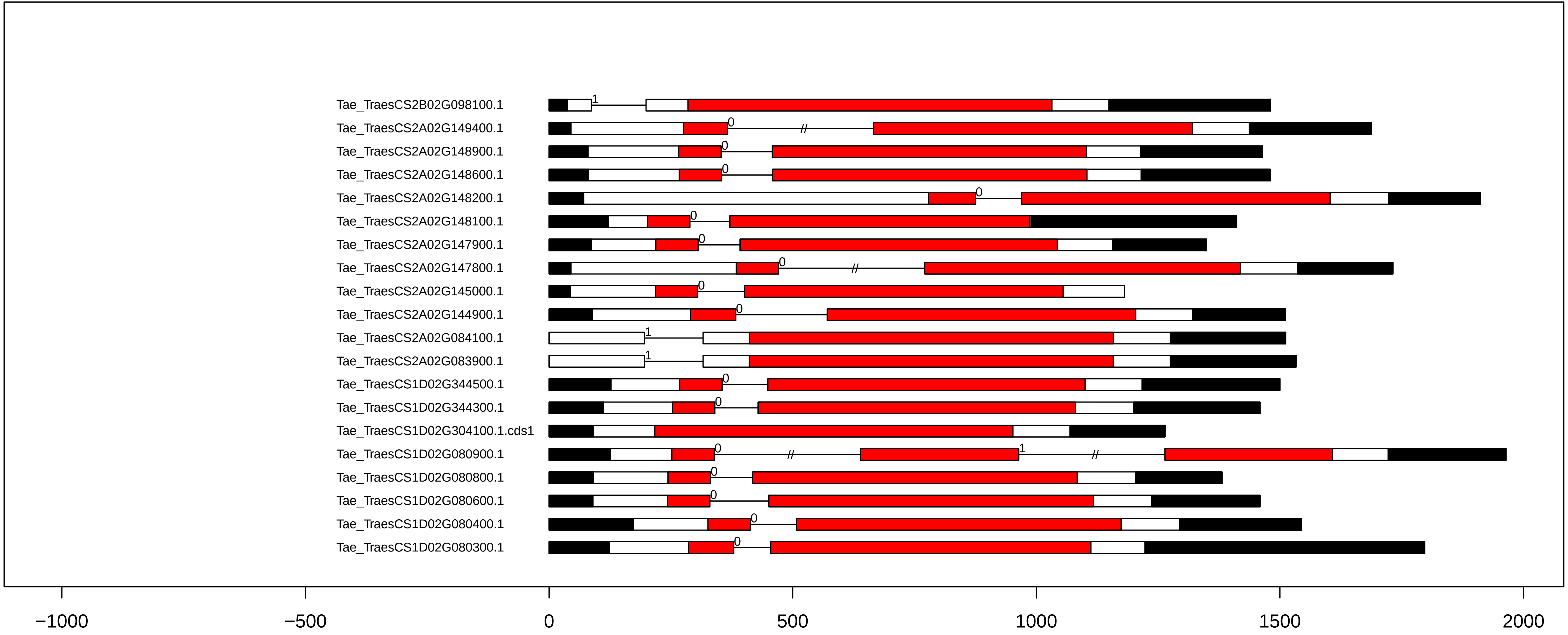

T.ae class III peroxidase I subfamily exon−intron and prx domain diagram (part 3)

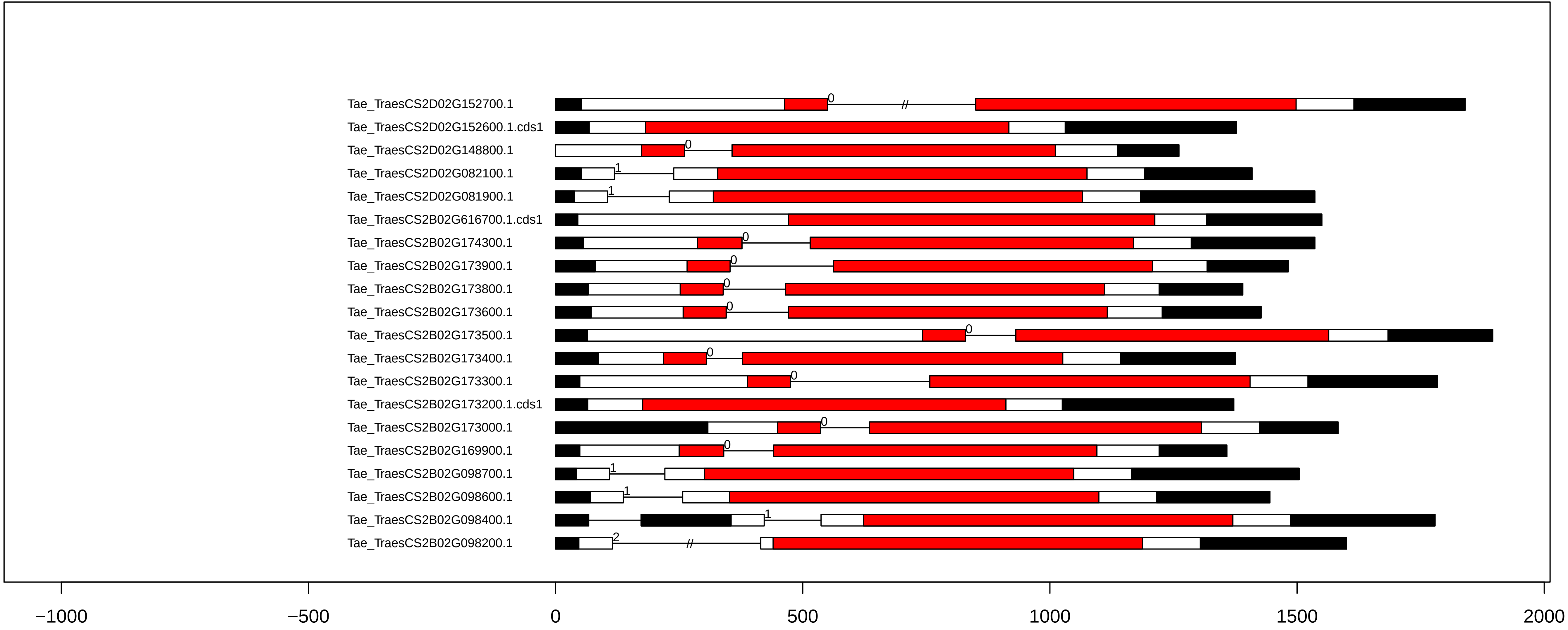

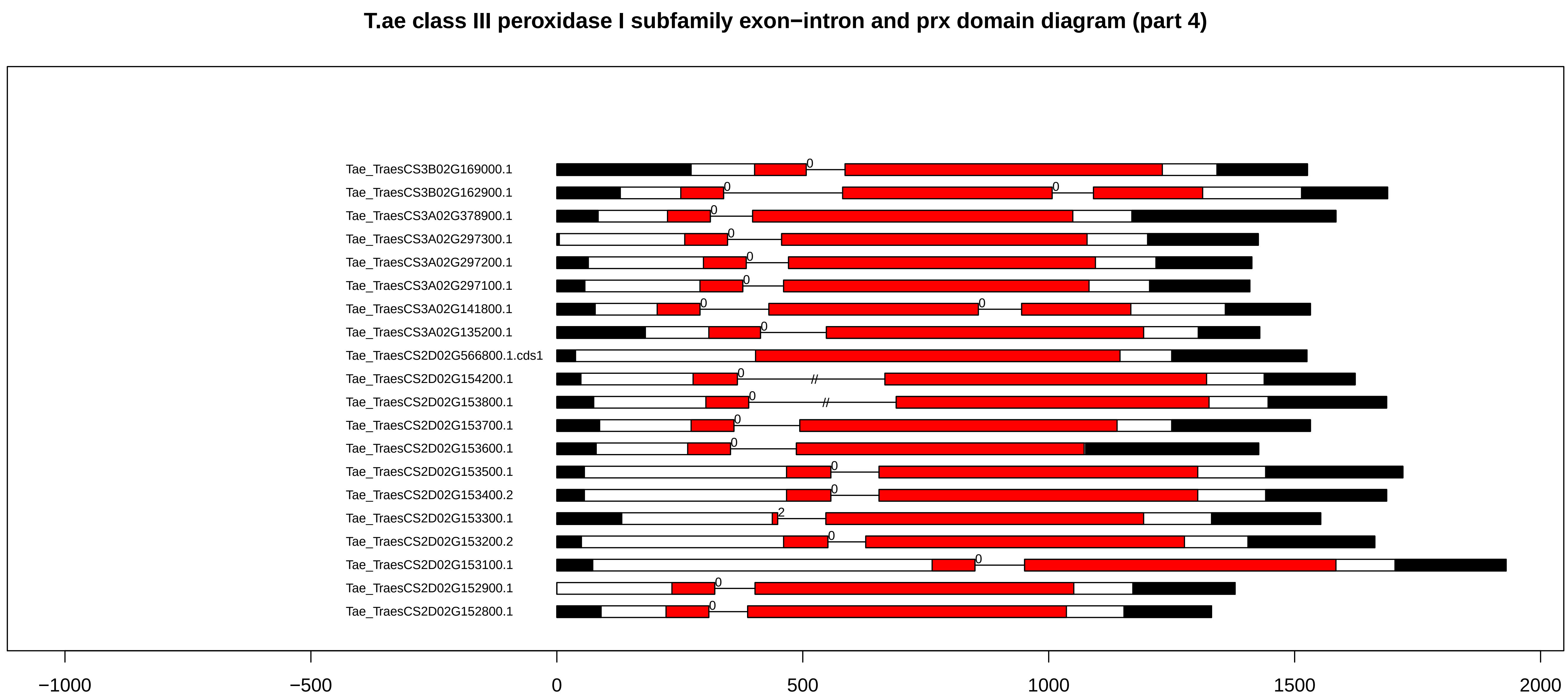

T.ae class III peroxidase I subfamily exon−intron and prx domain diagram (part 4)
Tae_TraesCS3B02G169000.1
Tae_TraesCS3B02G162900.1
Tae_TraesCS3A02G378900.1
Tae_TraesCS3A02G297300.1
Tae_TraesCS3A02G297200.1
Tae_TraesCS3A02G297100.1
Tae_TraesCS3A02G141800.1
Tae_TraesCS3A02G135200.1
Tae_TraesCS2D02G566800.1.cds1
Tae_TraesCS2D02G154200.1
Tae_TraesCS2D02G153800.1
Tae_TraesCS2D02G153700.1
Tae_TraesCS2D02G153600.1
Tae_TraesCS2D02G153500.1
Tae_TraesCS2D02G153400.2
Tae_TraesCS2D02G153300.1
Tae_TraesCS2D02G153200.2
Tae_TraesCS2D02G153100.1
Tae_TraesCS2D02G152900.1
Tae_TraesCS2D02G152800.1
−1000
−500
0
500
1000
1500
2000

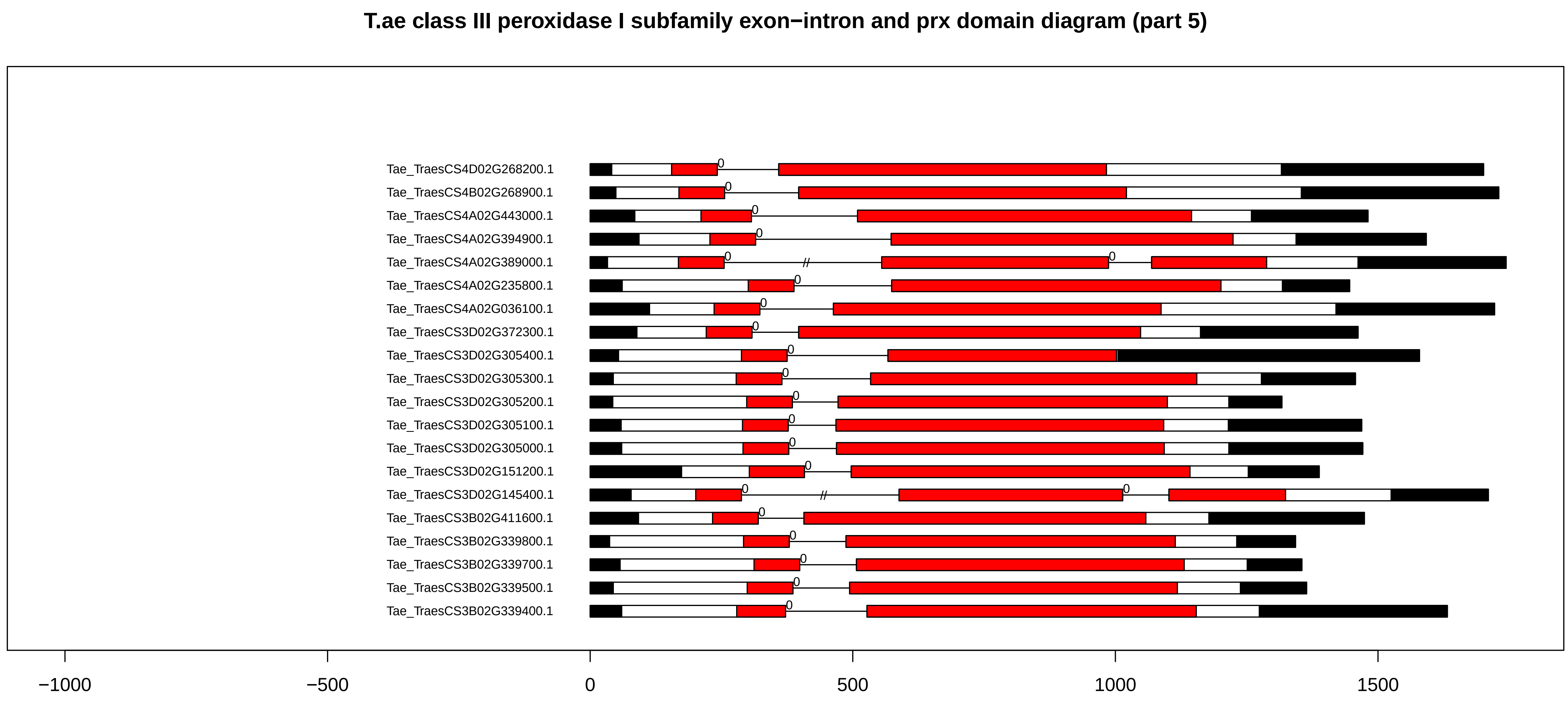

T.ae class III peroxidase I subfamily exon−intron and prx domain diagram (part 5)
Tae_TraesCS4D02G268200.1
Tae_TraesCS4B02G268900.1
Tae_TraesCS4A02G443000.1
Tae_TraesCS4A02G394900.1
Tae_TraesCS4A02G389000.1
Tae_TraesCS4A02G235800.1
Tae_TraesCS4A02G036100.1
Tae_TraesCS3D02G372300.1
Tae_TraesCS3D02G305400.1
Tae_TraesCS3D02G305300.1
Tae_TraesCS3D02G305200.1
Tae_TraesCS3D02G305100.1
Tae_TraesCS3D02G305000.1
Tae_TraesCS3D02G151200.1
Tae_TraesCS3D02G145400.1
Tae_TraesCS3B02G411600.1
Tae_TraesCS3B02G339800.1
Tae_TraesCS3B02G339700.1
Tae_TraesCS3B02G339500.1
Tae_TraesCS3B02G339400.1
−1000
−500
0
500
1000
1500

T.ae class III peroxidase I subfamily exon−intron and prx domain diagram (part 6)

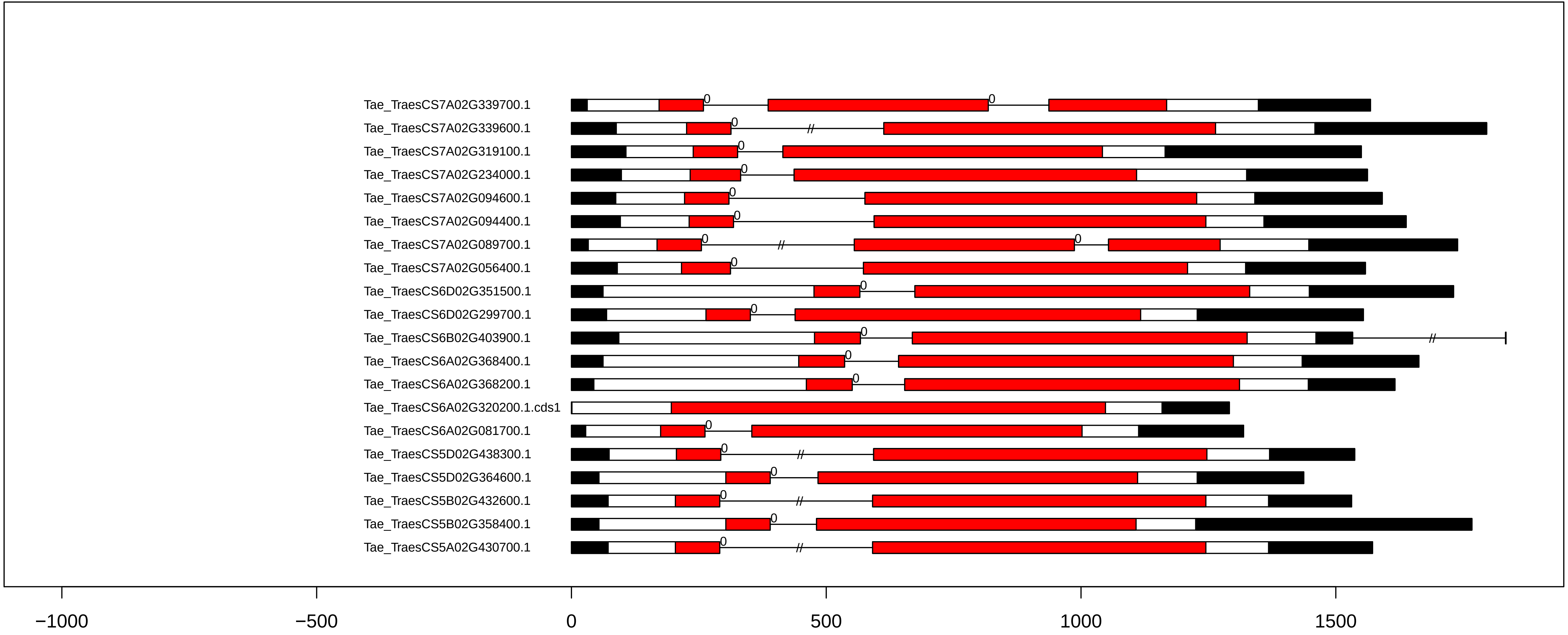

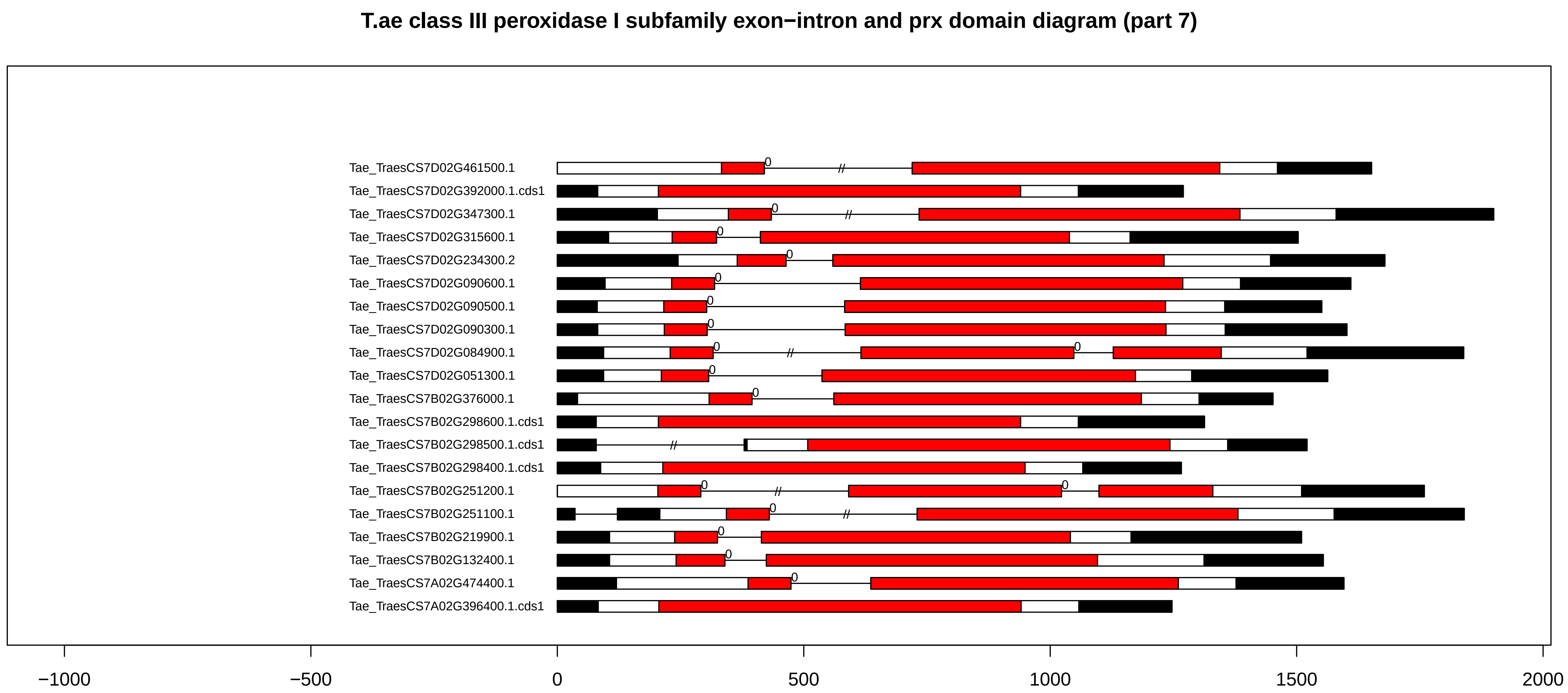
T.ae class III peroxidase I subfamily exon−intron and prx domain diagram (part 7)
Tae_TraesCS7D02G461500.1
Tae_TraesCS7D02G392000.1.cds1
Tae_TraesCS7D02G347300.1
Tae_TraesCS7D02G315600.1
Tae_TraesCS7D02G234300.2
Tae_TraesCS7D02G090600.1
Tae_TraesCS7D02G090500.1
Tae_TraesCS7D02G090300.1
Tae_TraesCS7D02G084900.1
Tae_TraesCS7D02G051300.1
Tae_TraesCS7B02G376000.1
Tae_TraesCS7B02G298600.1.cds1
Tae_TraesCS7B02G298500.1.cds1
Tae_TraesCS7B02G298400.1.cds1
Tae_TraesCS7B02G251200.1
Tae_TraesCS7B02G251100.1
Tae_TraesCS7B02G219900.1
Tae_TraesCS7B02G132400.1
Tae_TraesCS7A02G474400.1
Tae_TraesCS7A02G396400.1.cds1
−1000
−500
0
500
1000
1500
2000

T.ae class III peroxidase I subfamily exon−intron and prx domain diagram (part 8)

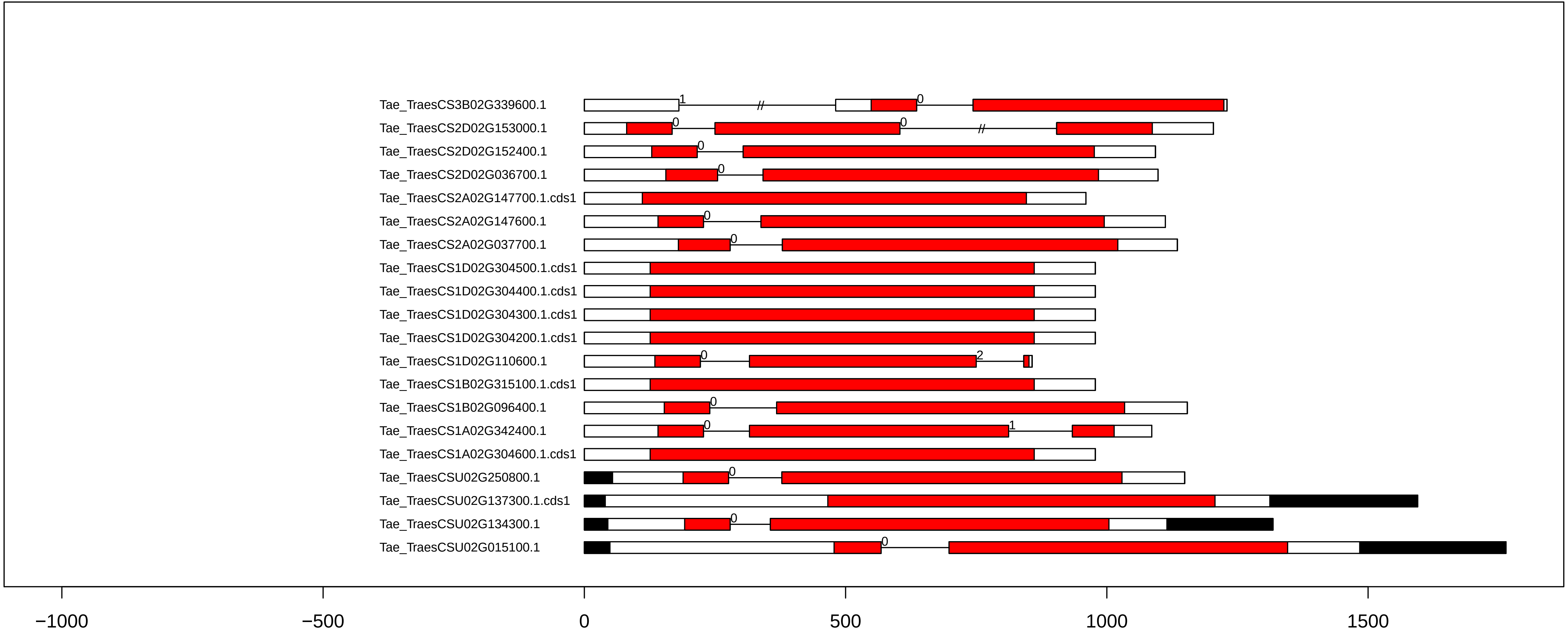

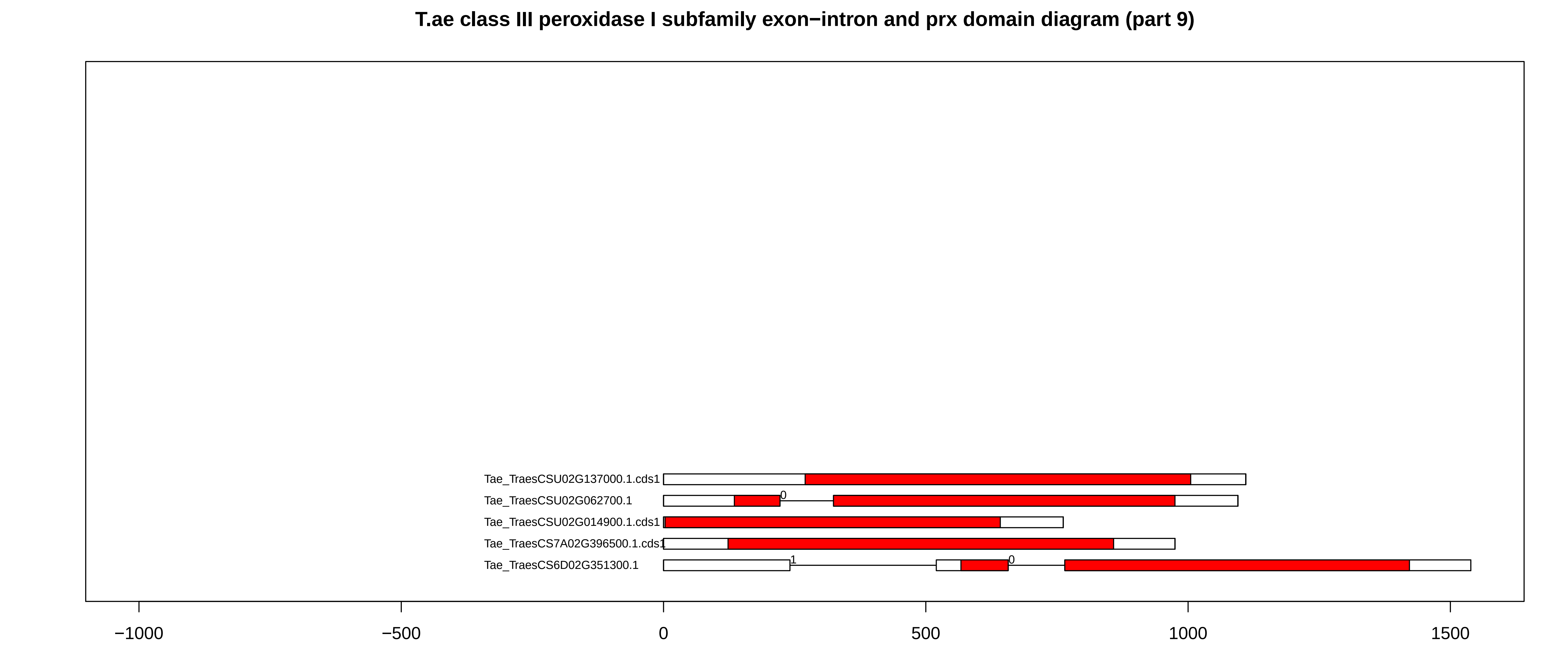
T.ae class III peroxidase I subfamily exon−intron and prx domain diagram (part 9)
Tae_TraesCSU02G137000.1.cds1
Tae_TraesCSU02G062700.1
0
Tae_TraesCSU02G014900.1.cds1
Tae_TraesCS7A02G396500.1.cds1
Tae_TraesCS6D02G351300.1
1
0
−1000
−500
0
500
1000
1500

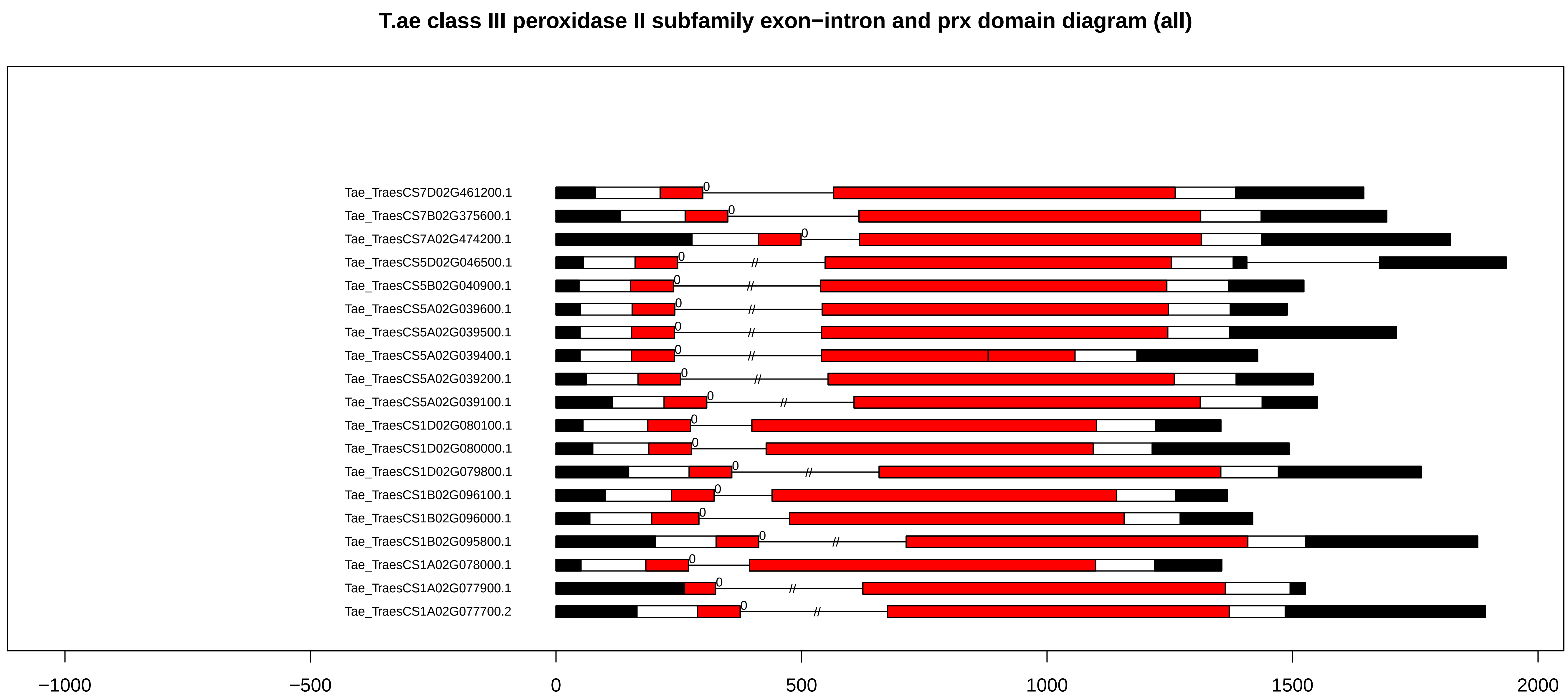
T.ae class III peroxidase II subfamily exon−intron and prx domain diagram (all)
Tae_TraesCS7D02G461200.1
Tae_TraesCS7B02G375600.1
Tae_TraesCS7A02G474200.1
Tae_TraesCS5D02G046500.1
Tae_TraesCS5B02G040900.1
Tae_TraesCS5A02G039600.1
Tae_TraesCS5A02G039500.1
Tae_TraesCS5A02G039400.1
Tae_TraesCS5A02G039200.1
Tae_TraesCS5A02G039100.1
Tae_TraesCS1D02G080100.1
Tae_TraesCS1D02G080000.1
Tae_TraesCS1D02G079800.1
Tae_TraesCS1B02G096100.1
Tae_TraesCS1B02G096000.1
Tae_TraesCS1B02G095800.1
Tae_TraesCS1A02G078000.1
Tae_TraesCS1A02G077900.1
Tae_TraesCS1A02G077700.2
−1000
−500
0
500
1000
1500
2000

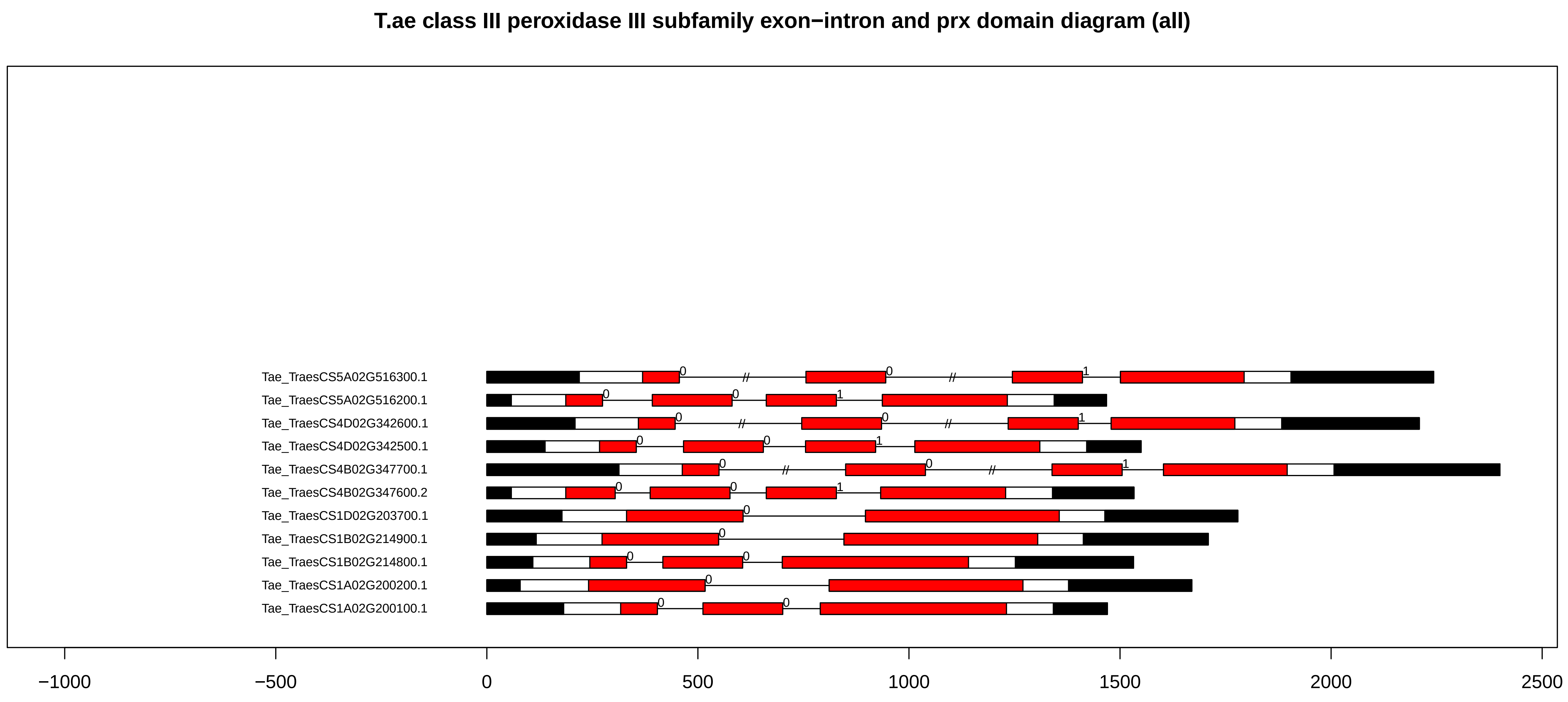

T.ae class III peroxidase III subfamily exon−intron and prx domain diagram (all)
Tae_TraesCS5A02G516300.1
Tae_TraesCS5A02G516200.1
Tae_TraesCS4D02G342600.1
Tae_TraesCS4D02G342500.1
Tae_TraesCS4B02G347700.1
Tae_TraesCS4B02G347600.2
Tae_TraesCS1D02G203700.1
Tae_TraesCS1B02G214900.1
Tae_TraesCS1B02G214800.1
Tae_TraesCS1A02G200200.1
Tae_TraesCS1A02G200100.1
−1000
−500
0
500
1000
1500
2000
2500

T.ae class III peroxidase V subfamily exon−intron and prx domain diagram (part 1)

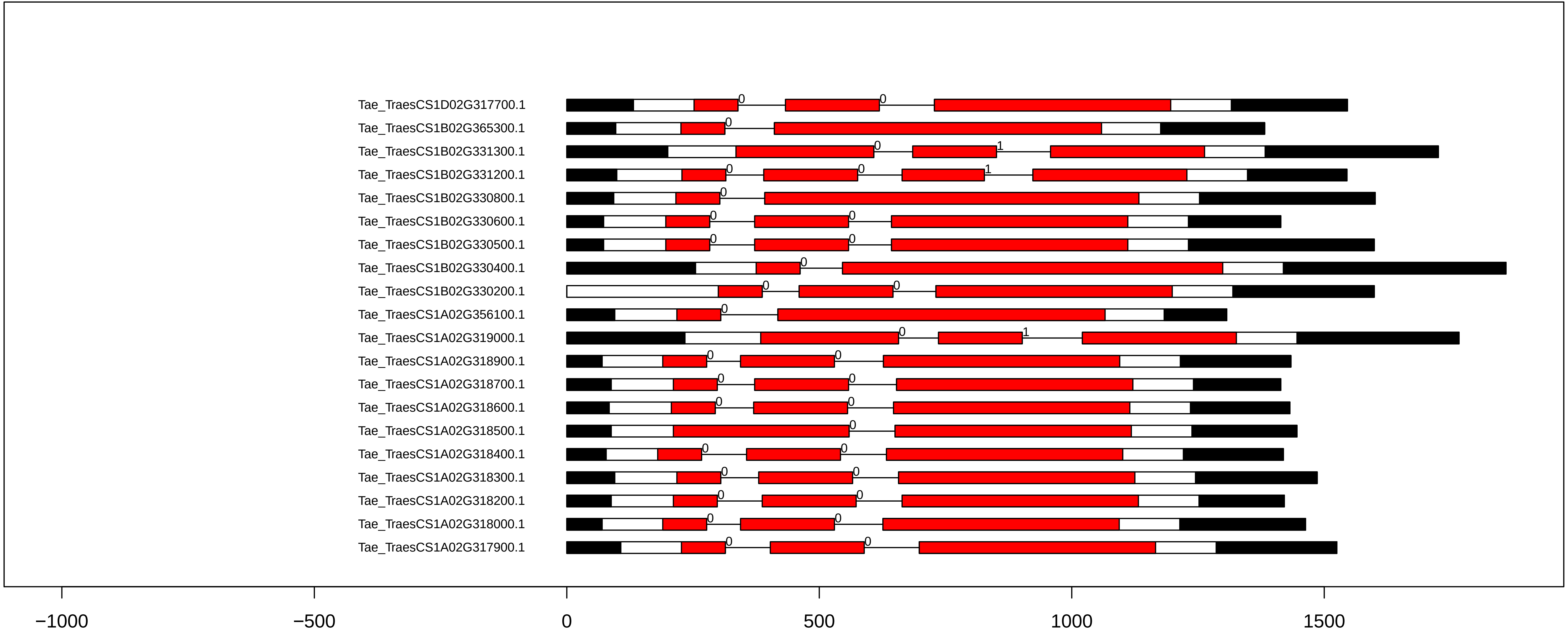

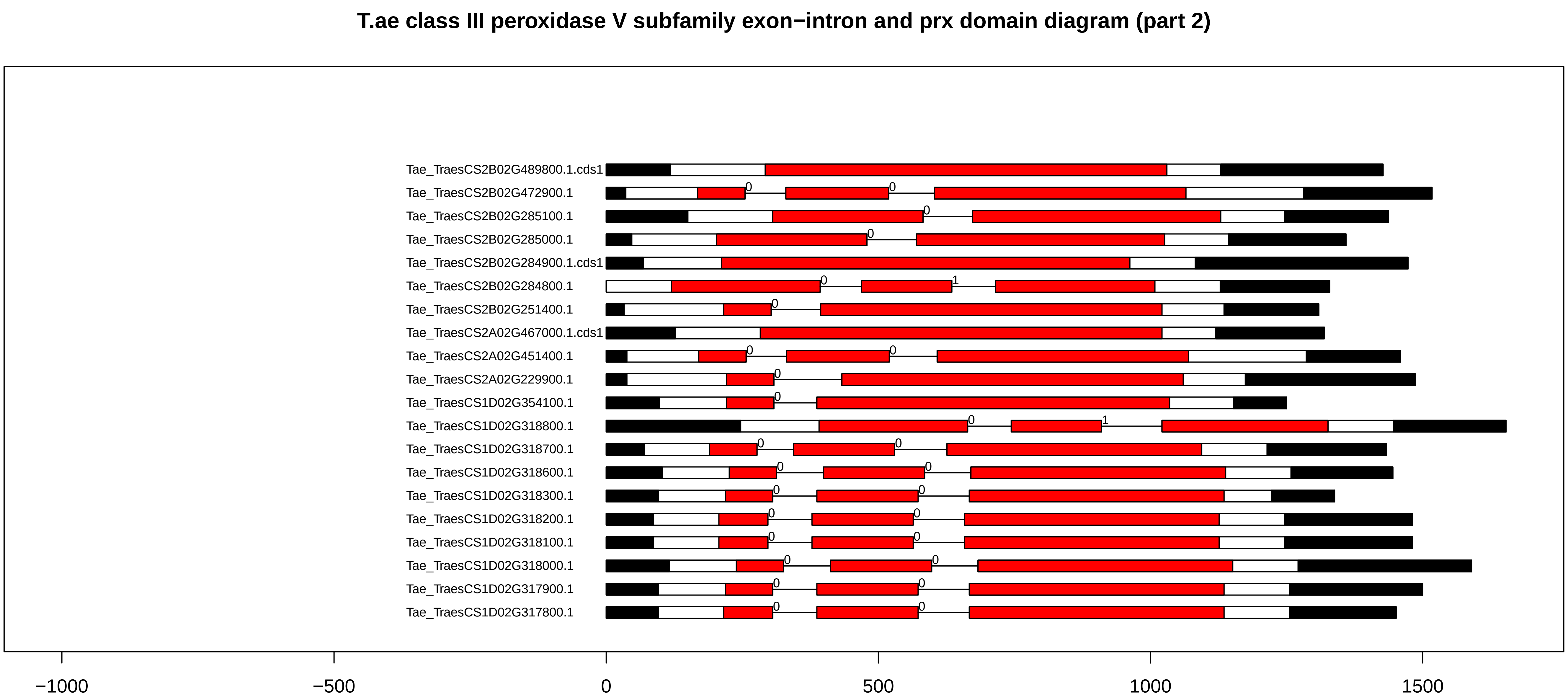
T.ae class III peroxidase V subfamily exon−intron and prx domain diagram (part 2)
Tae_TraesCS2B02G489800.1.cds1
Tae_TraesCS2B02G472900.1
Tae_TraesCS2B02G285100.1
Tae_TraesCS2B02G285000.1
Tae_TraesCS2B02G284900.1.cds1
Tae_TraesCS2B02G284800.1
Tae_TraesCS2B02G251400.1
Tae_TraesCS2A02G467000.1.cds1
Tae_TraesCS2A02G451400.1
Tae_TraesCS2A02G229900.1
Tae_TraesCS1D02G354100.1
Tae_TraesCS1D02G318800.1
Tae_TraesCS1D02G318700.1
Tae_TraesCS1D02G318600.1
Tae_TraesCS1D02G318300.1
Tae_TraesCS1D02G318200.1
Tae_TraesCS1D02G318100.1
Tae_TraesCS1D02G318000.1
Tae_TraesCS1D02G317900.1
Tae_TraesCS1D02G317800.1
−1000
−500
0
500
1000
1500

T.ae class III peroxidase V subfamily exon−intron and prx domain diagram (part 3)

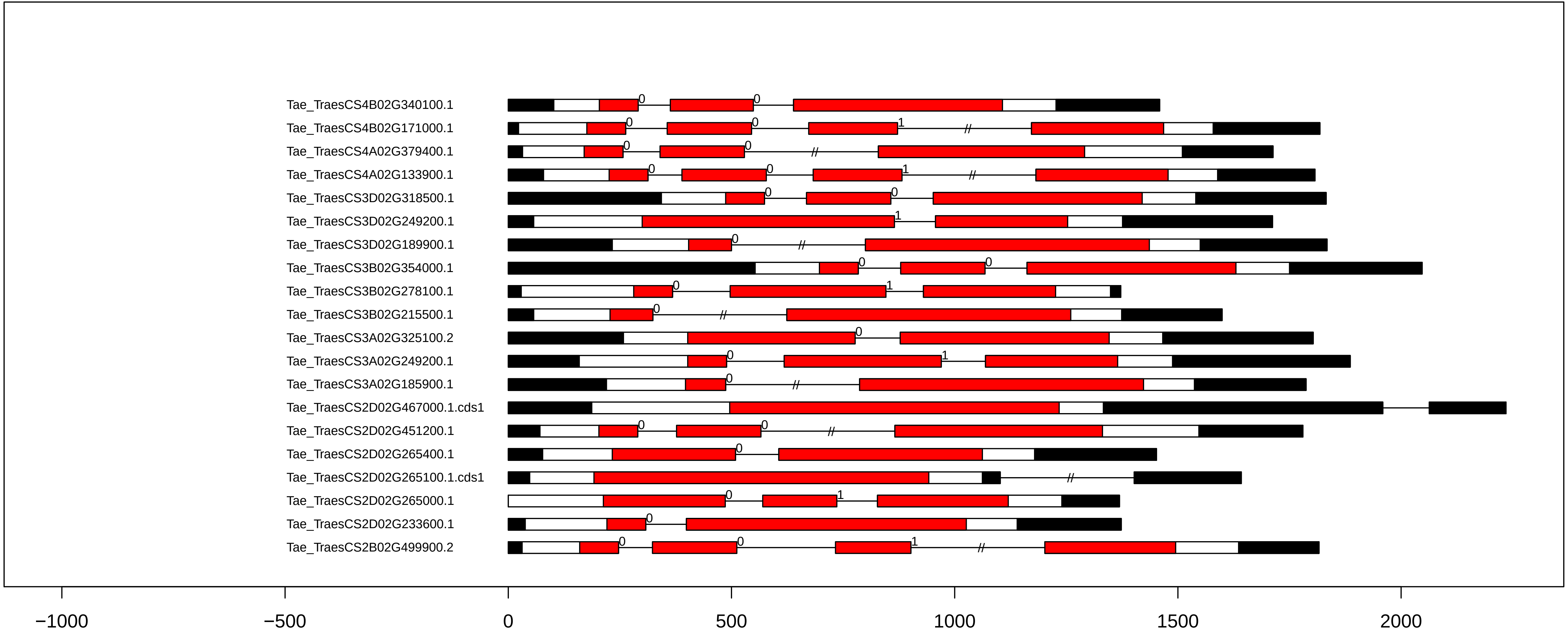

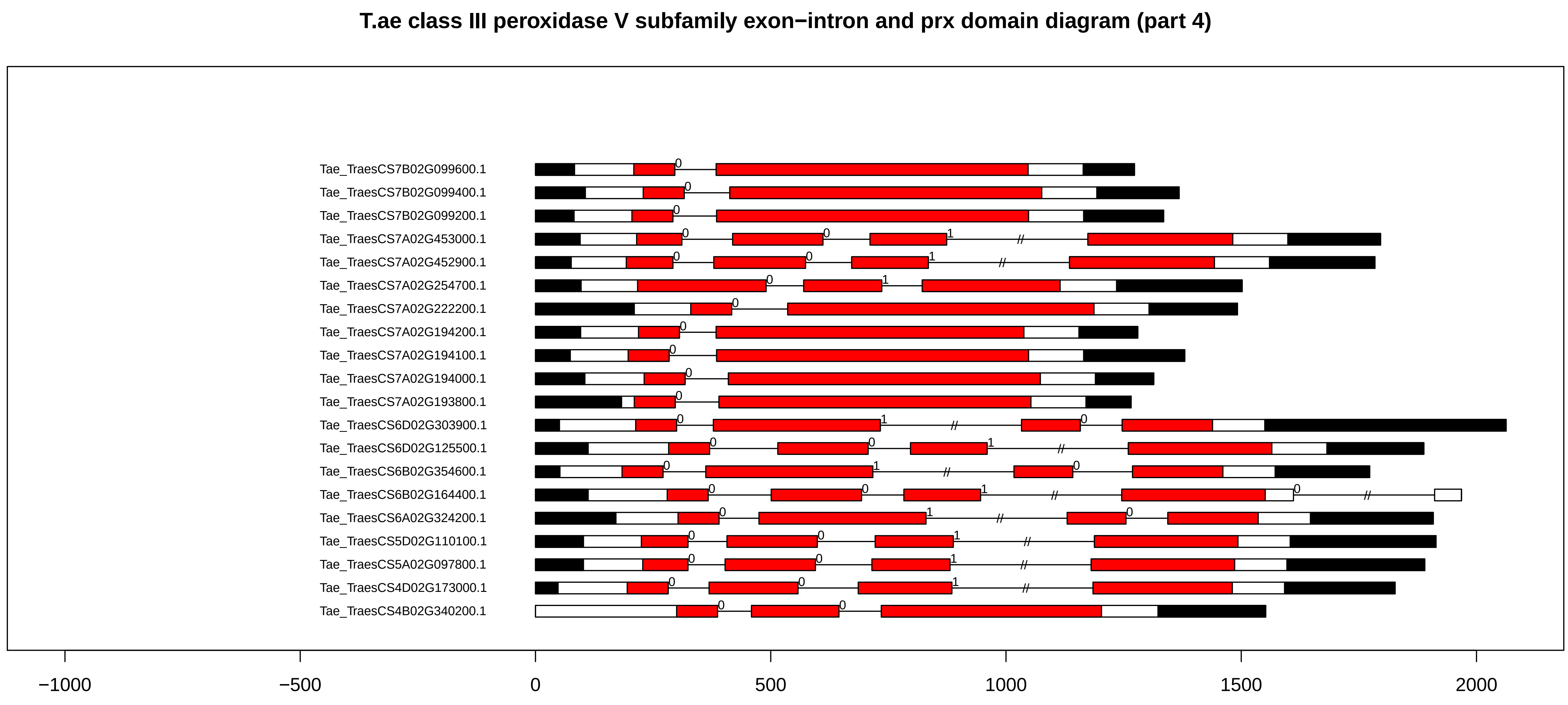
T.ae class III peroxidase V subfamily exon−intron and prx domain diagram (part 4)
Tae_TraesCS7B02G099600.1
Tae_TraesCS7B02G099400.1
Tae_TraesCS7B02G099200.1
Tae_TraesCS7A02G453000.1
Tae_TraesCS7A02G452900.1
Tae_TraesCS7A02G254700.1
Tae_TraesCS7A02G222200.1
Tae_TraesCS7A02G194200.1
Tae_TraesCS7A02G194100.1
Tae_TraesCS7A02G194000.1
Tae_TraesCS7A02G193800.1
Tae_TraesCS6D02G303900.1
Tae_TraesCS6D02G125500.1
Tae_TraesCS6B02G354600.1
Tae_TraesCS6B02G164400.1
Tae_TraesCS6A02G324200.1
Tae_TraesCS5D02G110100.1
Tae_TraesCS5A02G097800.1
Tae_TraesCS4D02G173000.1
Tae_TraesCS4B02G340200.1
−1000
−500
0
500
1000
1500
2000

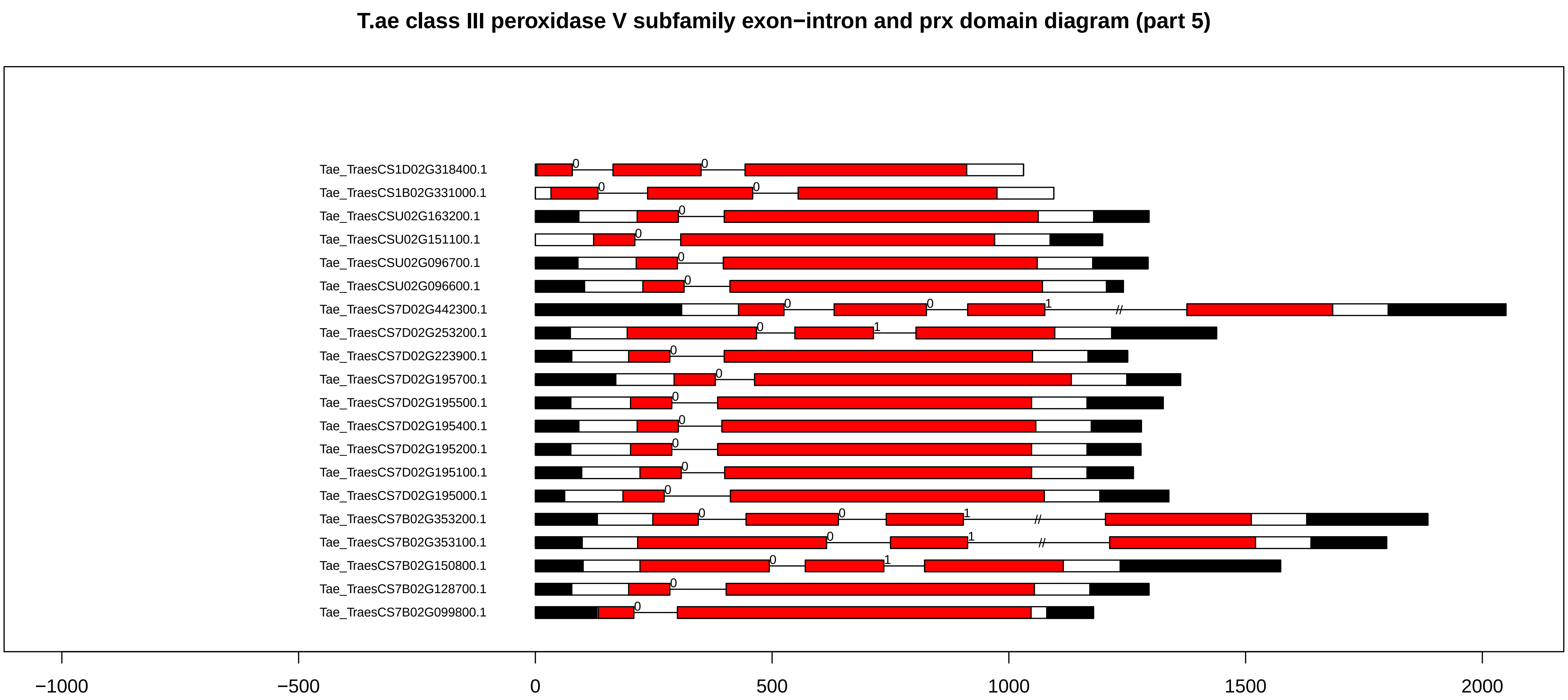

T.ae class III peroxidase V subfamily exon−intron and prx domain diagram (part 5)
Tae_TraesCS1D02G318400.1
Tae_TraesCS1B02G331000.1
Tae_TraesCSU02G163200.1
Tae_TraesCSU02G151100.1
Tae_TraesCSU02G096700.1
Tae_TraesCSU02G096600.1
Tae_TraesCS7D02G442300.1
Tae_TraesCS7D02G253200.1
Tae_TraesCS7D02G223900.1
Tae_TraesCS7D02G195700.1
Tae_TraesCS7D02G195500.1
Tae_TraesCS7D02G195400.1
Tae_TraesCS7D02G195200.1
Tae_TraesCS7D02G195100.1
Tae_TraesCS7D02G195000.1
Tae_TraesCS7B02G353200.1
Tae_TraesCS7B02G353100.1
Tae_TraesCS7B02G150800.1
Tae_TraesCS7B02G128700.1
Tae_TraesCS7B02G099800.1
−1000
−500
0
500
1000
1500
2000

T.ae class III peroxidase V subfamily exon−intron and prx domain diagram (part 6)

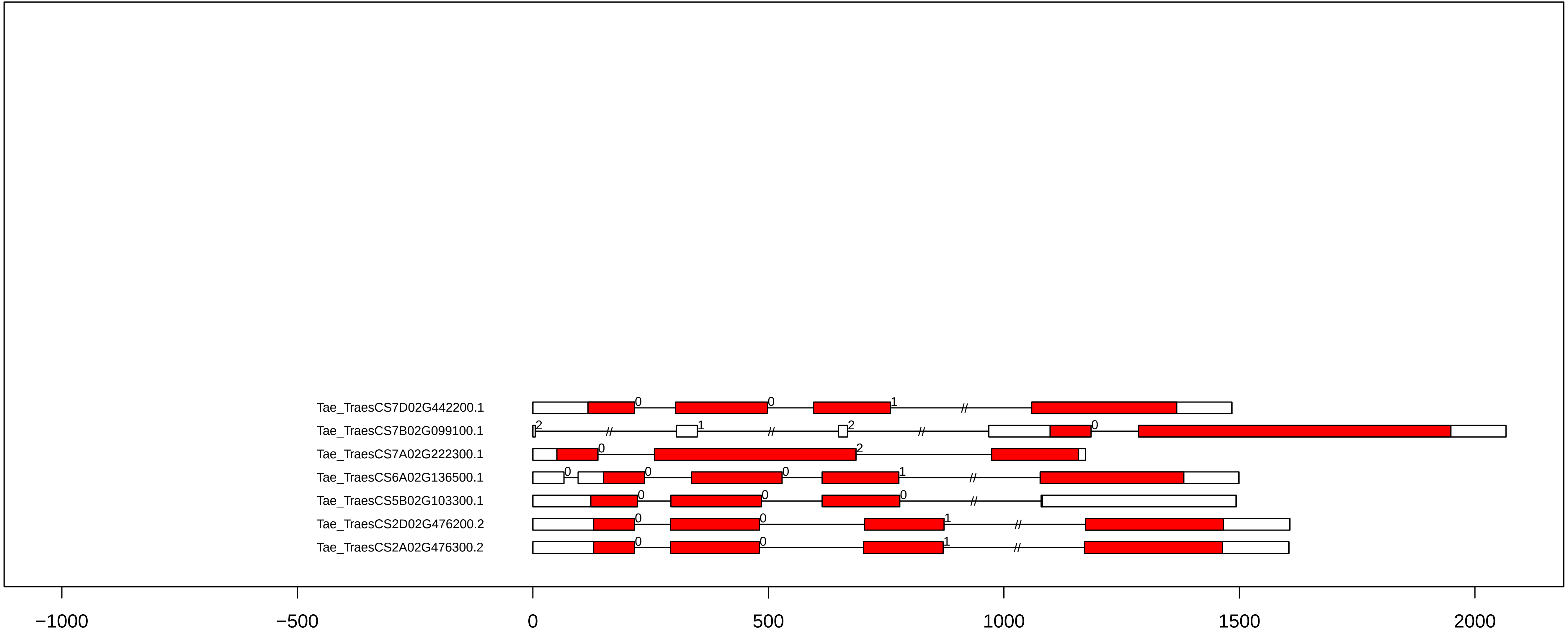

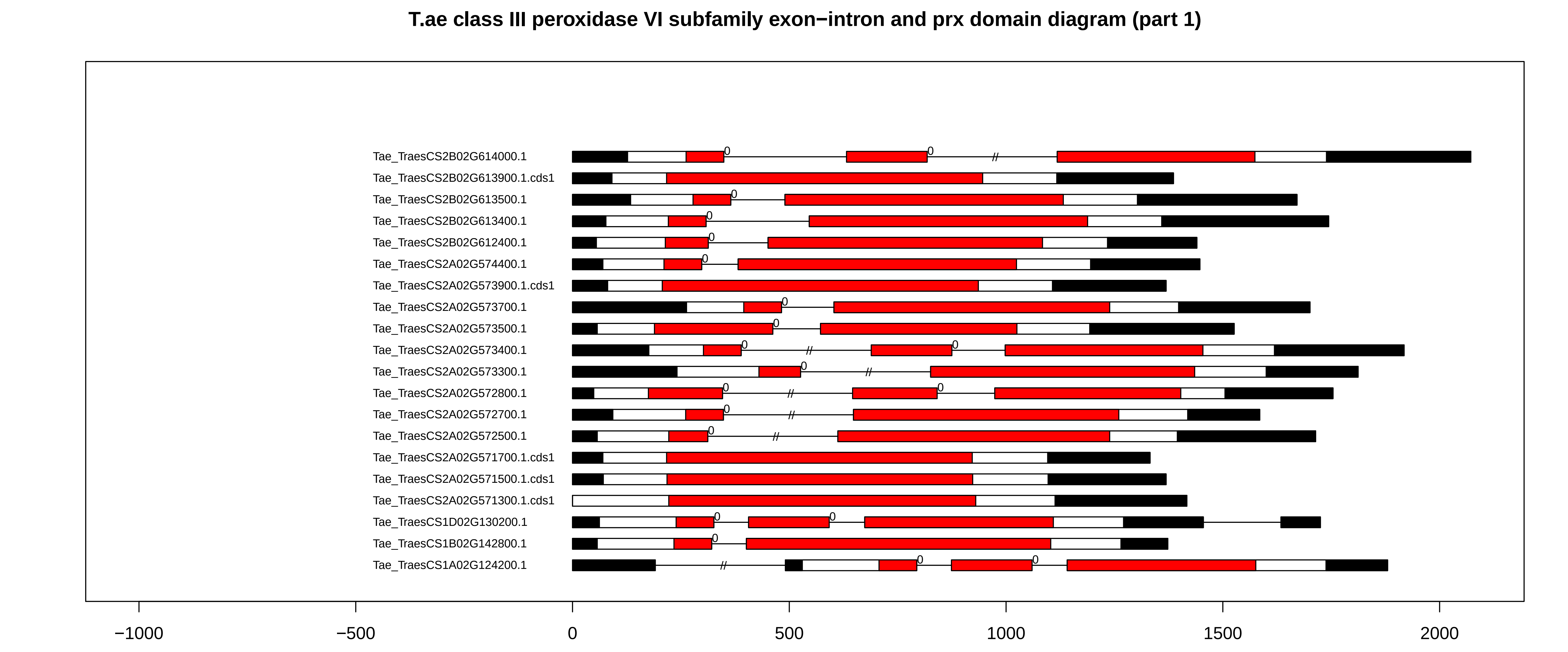
T.ae class III peroxidase VI subfamily exon−intron and prx domain diagram (part 1)
Tae_TraesCS2B02G614000.1
Tae_TraesCS2B02G613900.1.cds1
Tae_TraesCS2B02G613500.1
Tae_TraesCS2B02G613400.1
Tae_TraesCS2B02G612400.1
Tae_TraesCS2A02G574400.1
Tae_TraesCS2A02G573900.1.cds1
Tae_TraesCS2A02G573700.1
Tae_TraesCS2A02G573500.1
Tae_TraesCS2A02G573400.1
Tae_TraesCS2A02G573300.1
Tae_TraesCS2A02G572800.1
Tae_TraesCS2A02G572700.1
Tae_TraesCS2A02G572500.1
Tae_TraesCS2A02G571700.1.cds1
Tae_TraesCS2A02G571500.1.cds1
Tae_TraesCS2A02G571300.1.cds1
Tae_TraesCS1D02G130200.1
Tae_TraesCS1B02G142800.1
Tae_TraesCS1A02G124200.1
−1000
−500
0
500
1000
1500
2000

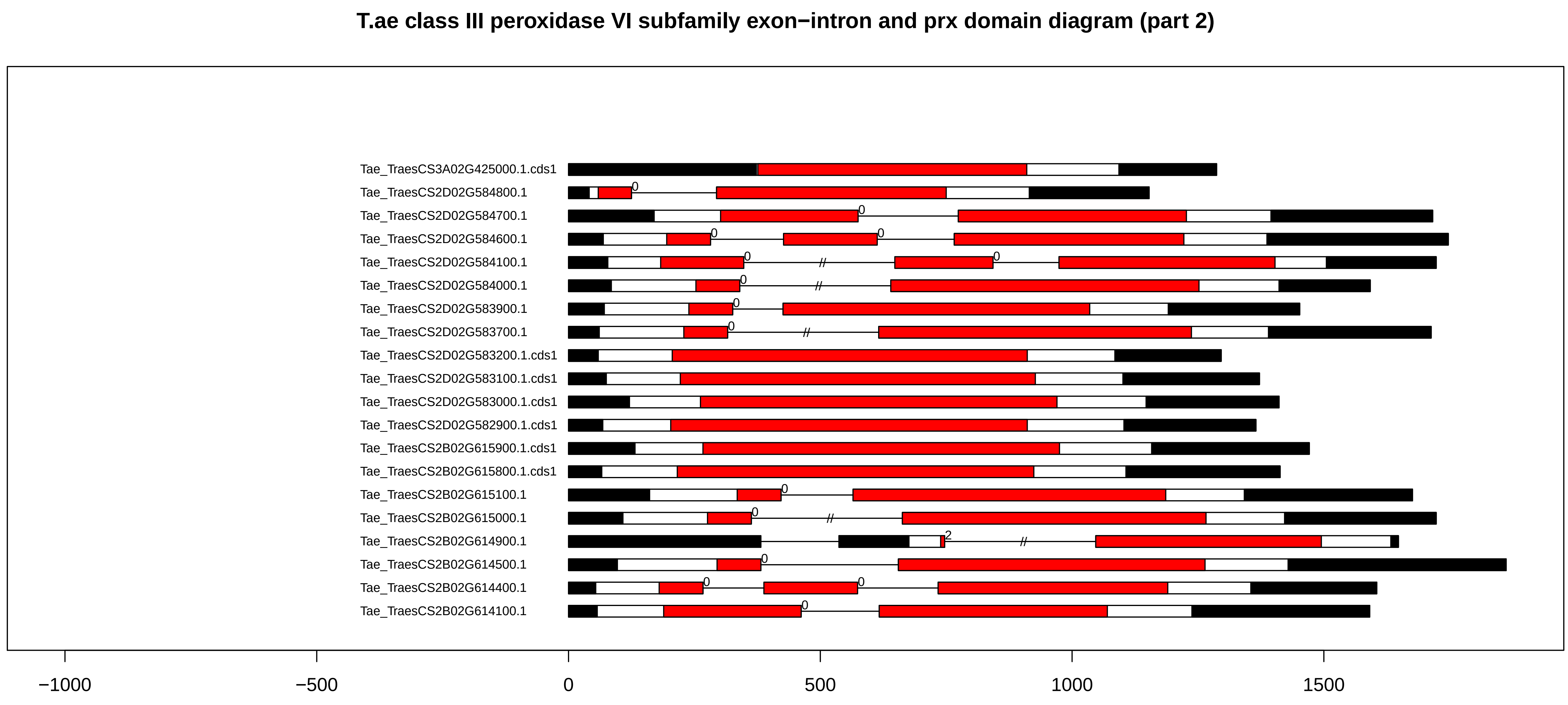
T.ae class III peroxidase VI subfamily exon−intron and prx domain diagram (part 2)
Tae_TraesCS3A02G425000.1.cds1
Tae_TraesCS2D02G584800.1
Tae_TraesCS2D02G584700.1
Tae_TraesCS2D02G584600.1
Tae_TraesCS2D02G584100.1
Tae_TraesCS2D02G584000.1
Tae_TraesCS2D02G583900.1
Tae_TraesCS2D02G583700.1
Tae_TraesCS2D02G583200.1.cds1
Tae_TraesCS2D02G583100.1.cds1
Tae_TraesCS2D02G583000.1.cds1
Tae_TraesCS2D02G582900.1.cds1
Tae_TraesCS2B02G615900.1.cds1
Tae_TraesCS2B02G615800.1.cds1
Tae_TraesCS2B02G615100.1
Tae_TraesCS2B02G615000.1
Tae_TraesCS2B02G614900.1
Tae_TraesCS2B02G614500.1
Tae_TraesCS2B02G614400.1
Tae_TraesCS2B02G614100.1
−1000
−500
0
500
1000
1500

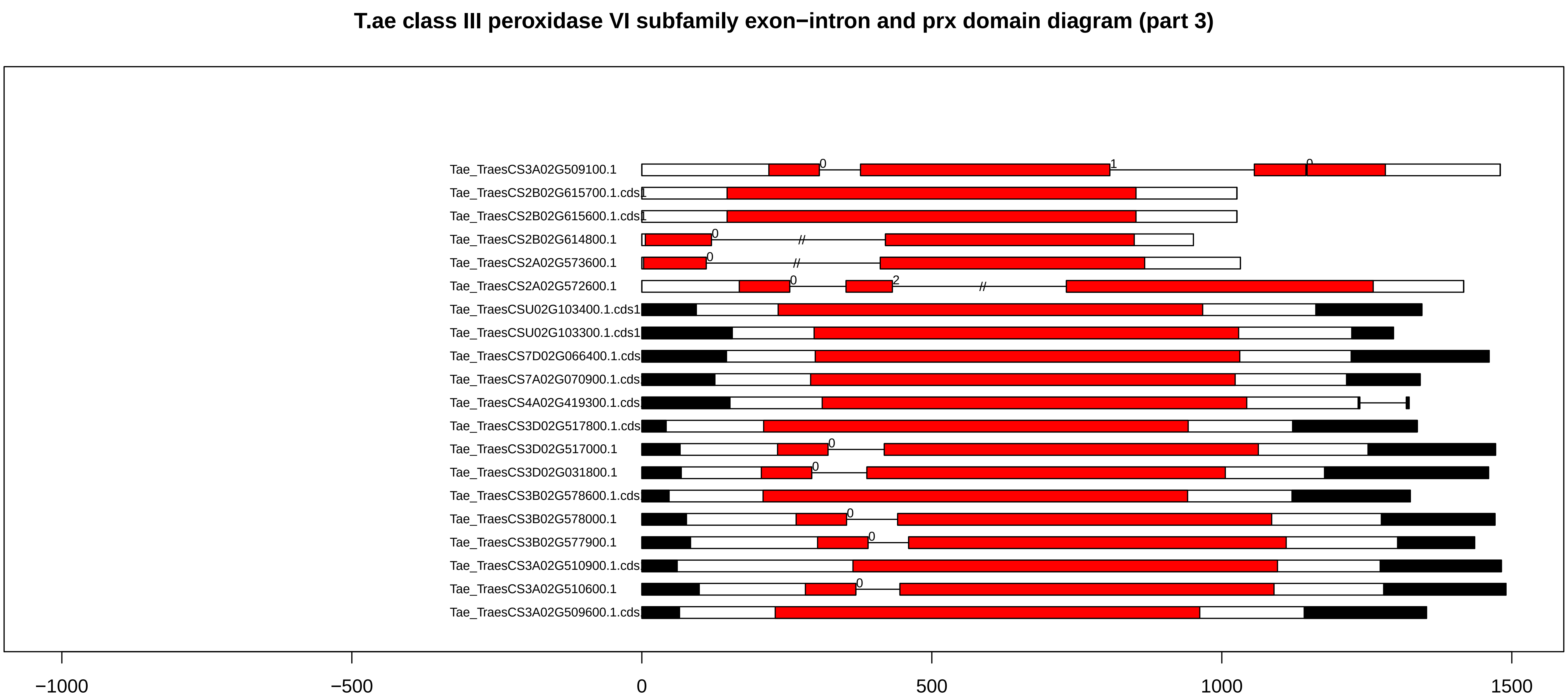

T.ae class III peroxidase VI subfamily exon−intron and prx domain diagram (part 3)
Tae_TraesCS3A02G509100.1
Tae_TraesCS2B02G615700.1.cds1
Tae_TraesCS2B02G615600.1.cds1
Tae_TraesCS2B02G614800.1
Tae_TraesCS2A02G573600.1
Tae_TraesCS2A02G572600.1
Tae_TraesCSU02G103400.1.cds1
Tae_TraesCSU02G103300.1.cds1
Tae_TraesCS7D02G066400.1.cds
Tae_TraesCS7A02G070900.1.cds
Tae_TraesCS4A02G419300.1.cds
Tae_TraesCS3D02G517800.1.cds
Tae_TraesCS3D02G517000.1
Tae_TraesCS3D02G031800.1
Tae_TraesCS3B02G578600.1.cds
Tae_TraesCS3B02G578000.1
Tae_TraesCS3B02G577900.1
Tae_TraesCS3A02G510900.1.cds
Tae_TraesCS3A02G510600.1
Tae_TraesCS3A02G509600.1.cds
−1000
−500
0
500
1000
1500

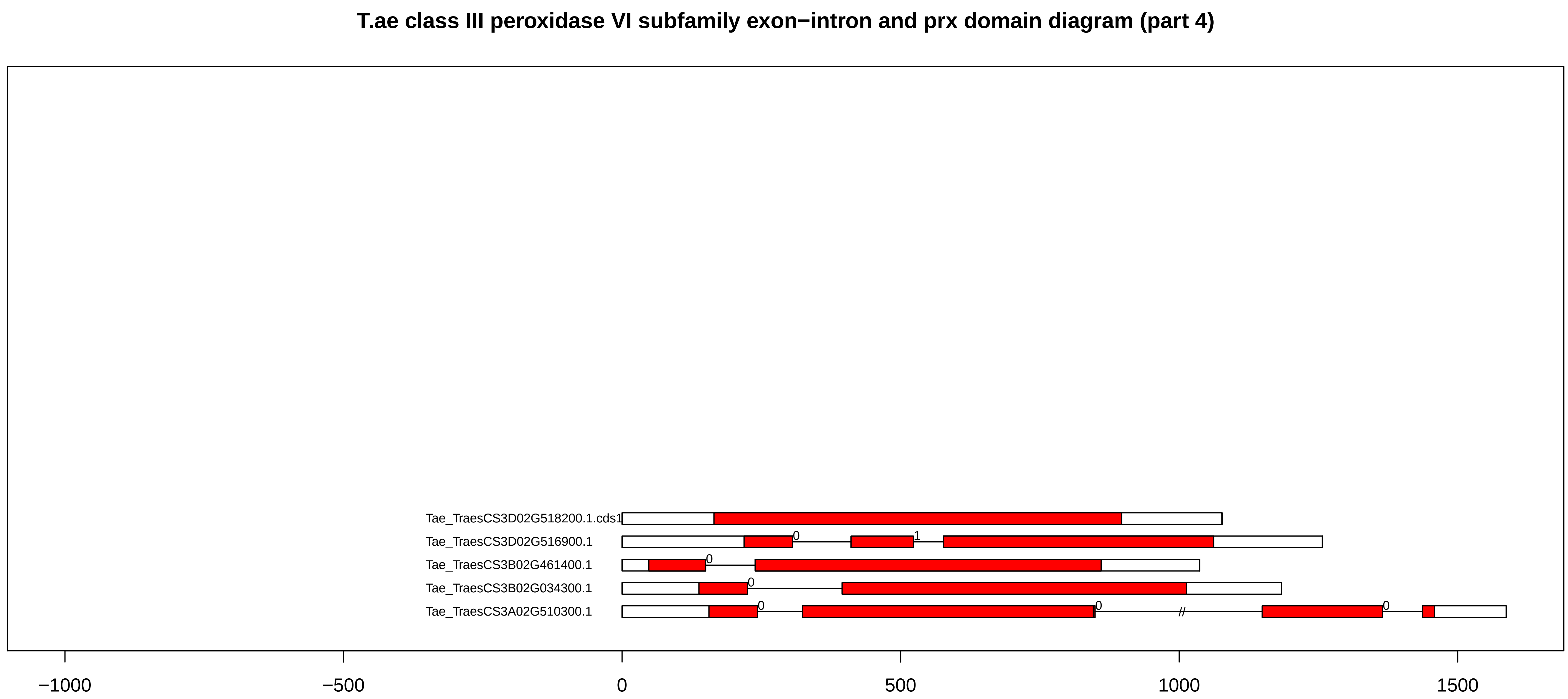
T.ae class III peroxidase VI subfamily exon−intron and prx domain diagram (part 4)
Tae_TraesCS3D02G518200.1.cds1
Tae_TraesCS3D02G516900.1
Tae_TraesCS3B02G461400.1
Tae_TraesCS3B02G034300.1
Tae_TraesCS3A02G510300.1
−1000
−500
0
500
1000
1500

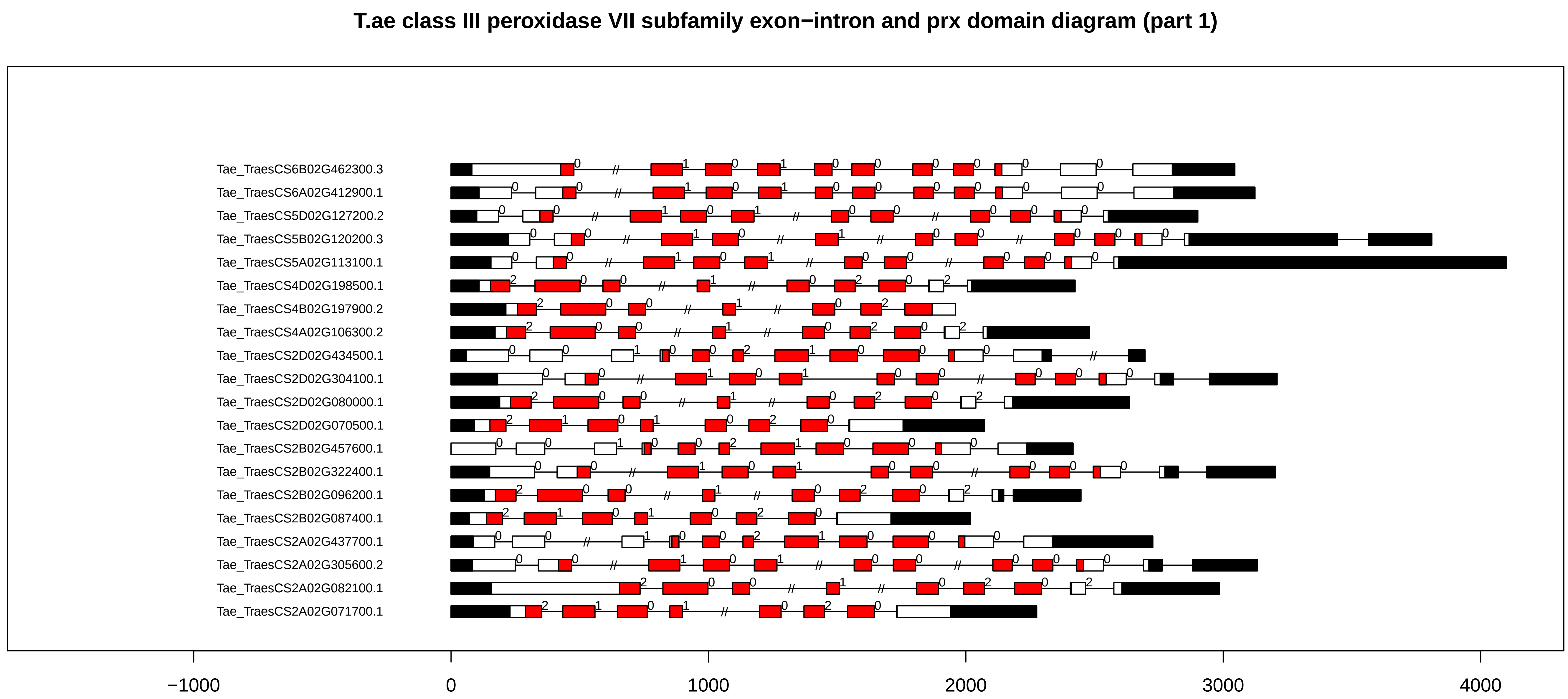
T.ae class III peroxidase VII subfamily exon−intron and prx domain diagram (part 1)
Tae_TraesCS6B02G462300.3
Tae_TraesCS6A02G412900.1
Tae_TraesCS5D02G127200.2
Tae_TraesCS5B02G120200.3
Tae_TraesCS5A02G113100.1
Tae_TraesCS4D02G198500.1
Tae_TraesCS4B02G197900.2
Tae_TraesCS4A02G106300.2
Tae_TraesCS2D02G434500.1
Tae_TraesCS2D02G304100.1
Tae_TraesCS2D02G080000.1
Tae_TraesCS2D02G070500.1
Tae_TraesCS2B02G457600.1
Tae_TraesCS2B02G322400.1
Tae_TraesCS2B02G096200.1
Tae_TraesCS2B02G087400.1
Tae_TraesCS2A02G437700.1
Tae_TraesCS2A02G305600.2
Tae_TraesCS2A02G082100.1
Tae_TraesCS2A02G071700.1
−1000
0
1000
2000
3000
4000

T.ae class III peroxidase VII subfamily exon−intron and prx domain diagram (part 2)

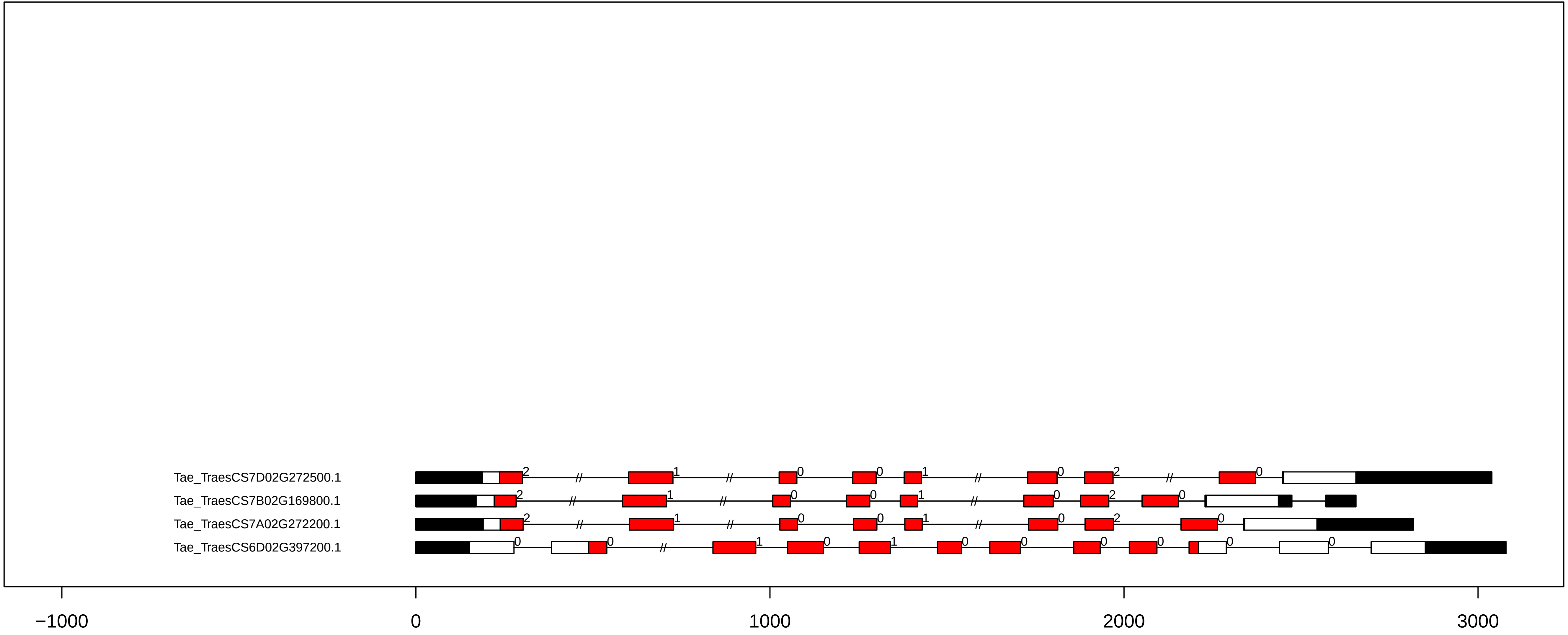

T.ae class III peroxidase IX subfamily exon−intron and prx domain diagram (part 1)

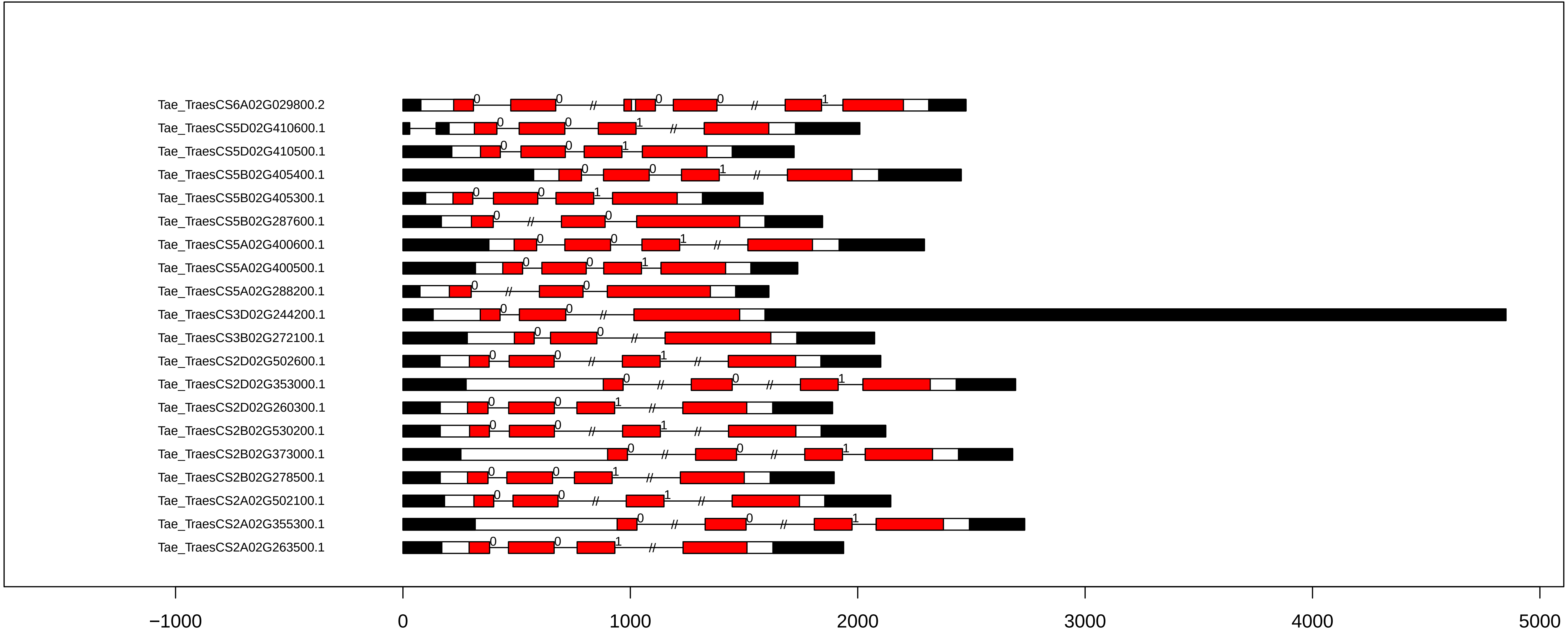

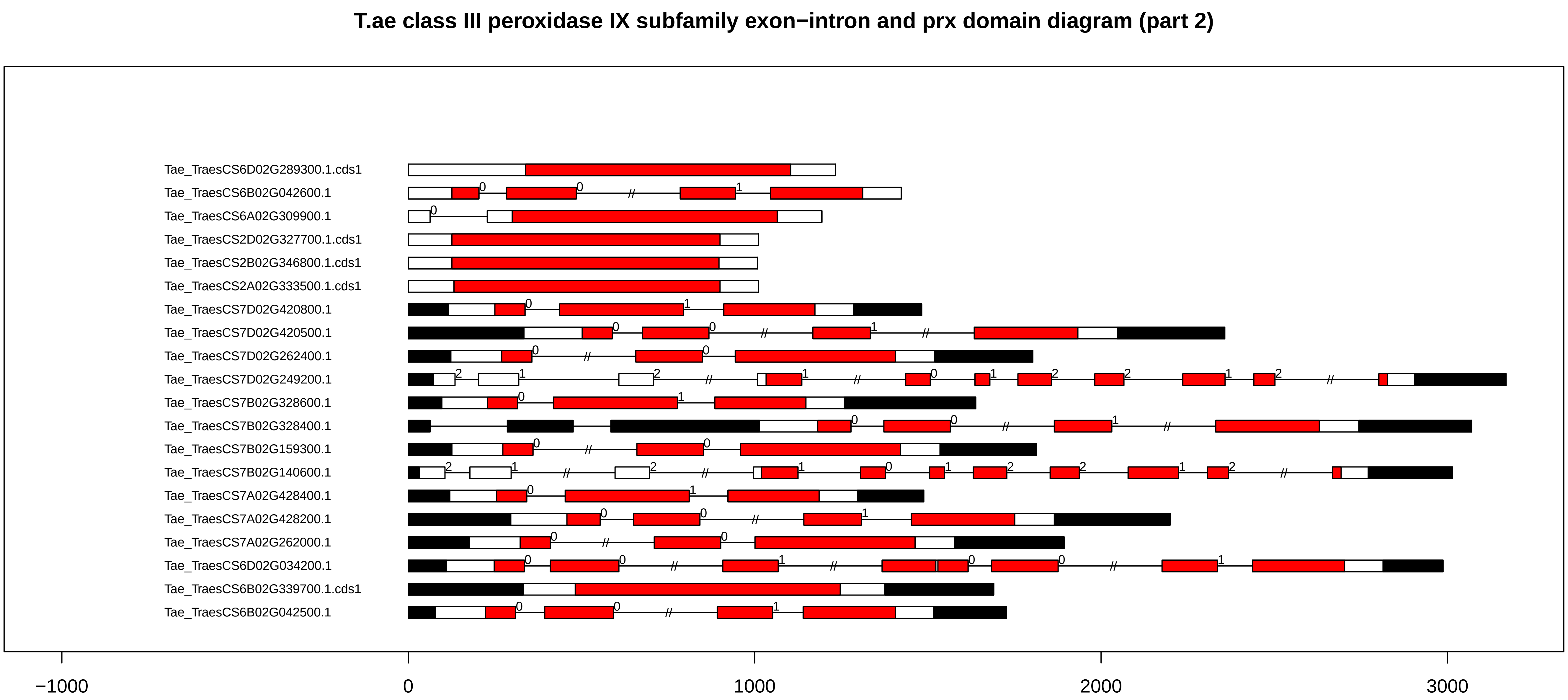
T.ae class III peroxidase IX subfamily exon−intron and prx domain diagram (part 2)
Tae_TraesCS6D02G289300.1.cds1
Tae_TraesCS6B02G042600.1
Tae_TraesCS6A02G309900.1
Tae_TraesCS2D02G327700.1.cds1
Tae_TraesCS2B02G346800.1.cds1
Tae_TraesCS2A02G333500.1.cds1
Tae_TraesCS7D02G420800.1
Tae_TraesCS7D02G420500.1
Tae_TraesCS7D02G262400.1
Tae_TraesCS7D02G249200.1
Tae_TraesCS7B02G328600.1
Tae_TraesCS7B02G328400.1
Tae_TraesCS7B02G159300.1
Tae_TraesCS7B02G140600.1
Tae_TraesCS7A02G428400.1
Tae_TraesCS7A02G428200.1
Tae_TraesCS7A02G262000.1
Tae_TraesCS6D02G034200.1
Tae_TraesCS6B02G339700.1.cds1
Tae_TraesCS6B02G042500.1
−1000
0
1000
2000
3000

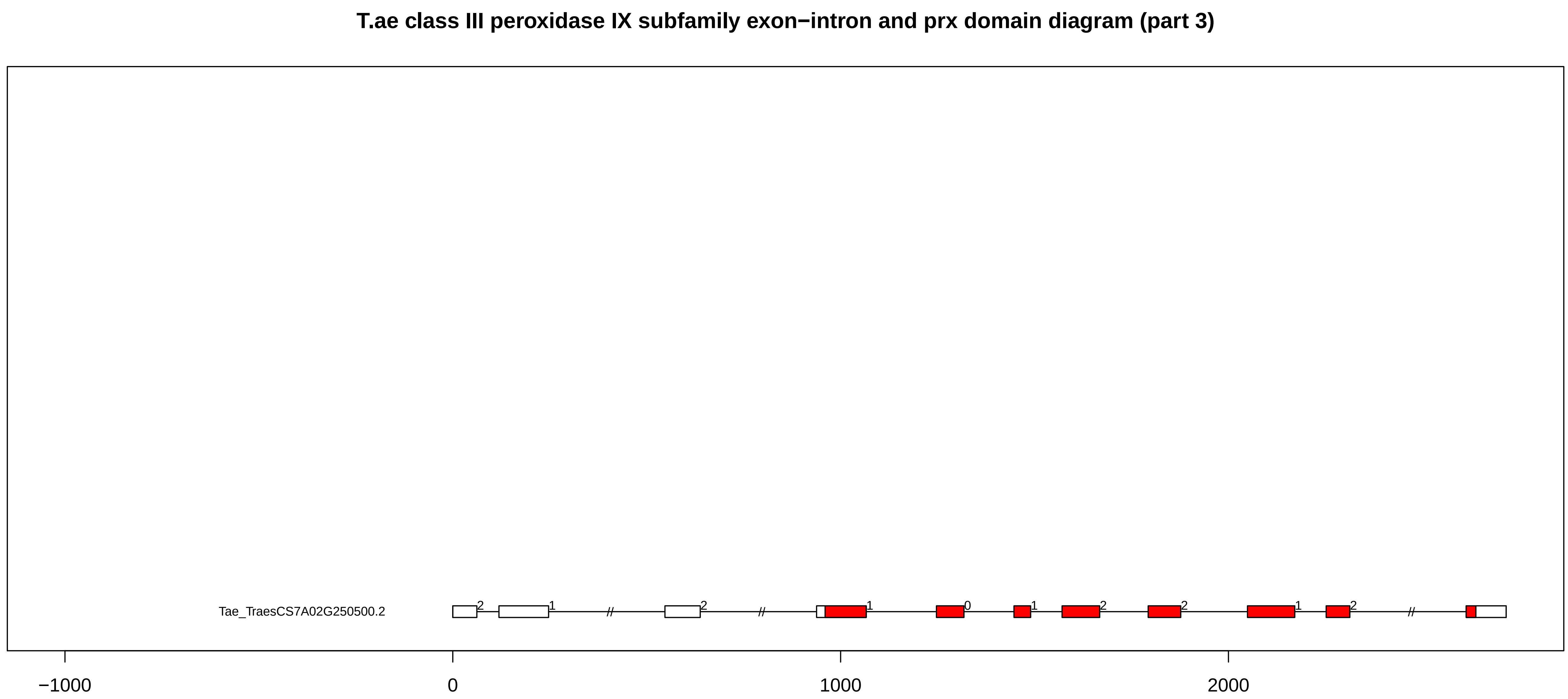
T.ae class III peroxidase IX subfamily exon−intron and prx domain diagram (part 3)
Tae_TraesCS7A02G250500.2
2
1
2
1
0
1
2
2
1
2
−1000
0
1000
2000

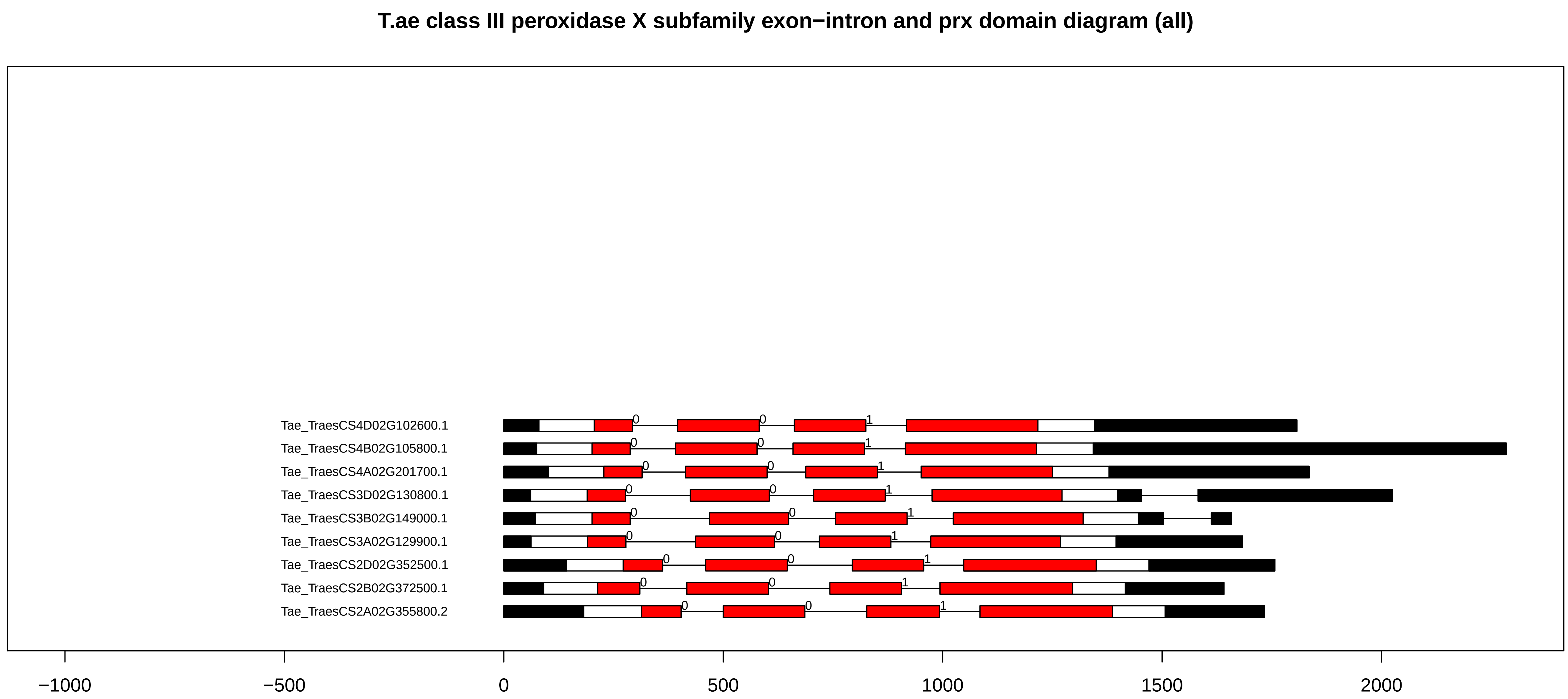
T.ae class III peroxidase X subfamily exon−intron and prx domain diagram (all)
Tae_TraesCS4D02G102600.1
Tae_TraesCS4B02G105800.1
Tae_TraesCS4A02G201700.1
Tae_TraesCS3D02G130800.1
Tae_TraesCS3B02G149000.1
Tae_TraesCS3A02G129900.1
Tae_TraesCS2D02G352500.1
Tae_TraesCS2B02G372500.1
Tae_TraesCS2A02G355800.2
−1000
−500
0
500
1000
1500
2000

T.ae class III peroxidase XI subfamily exon−intron and prx domain diagram (all)

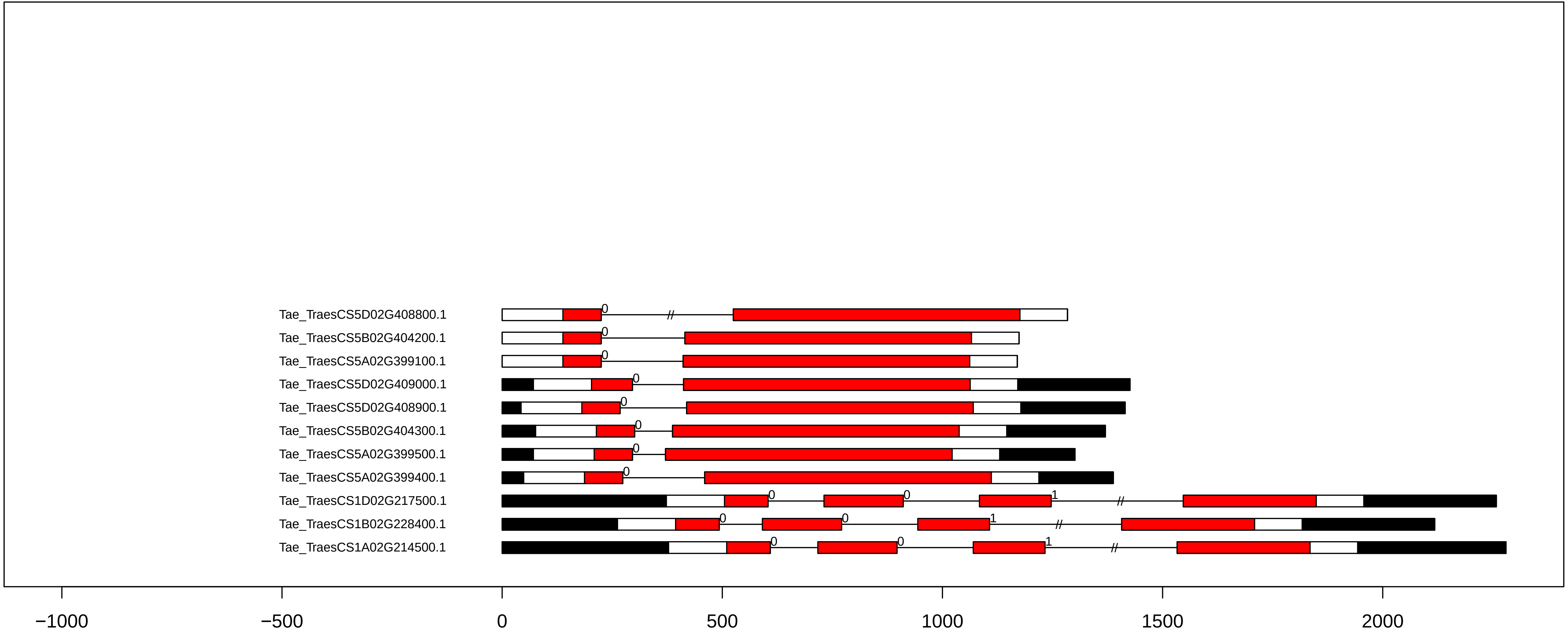

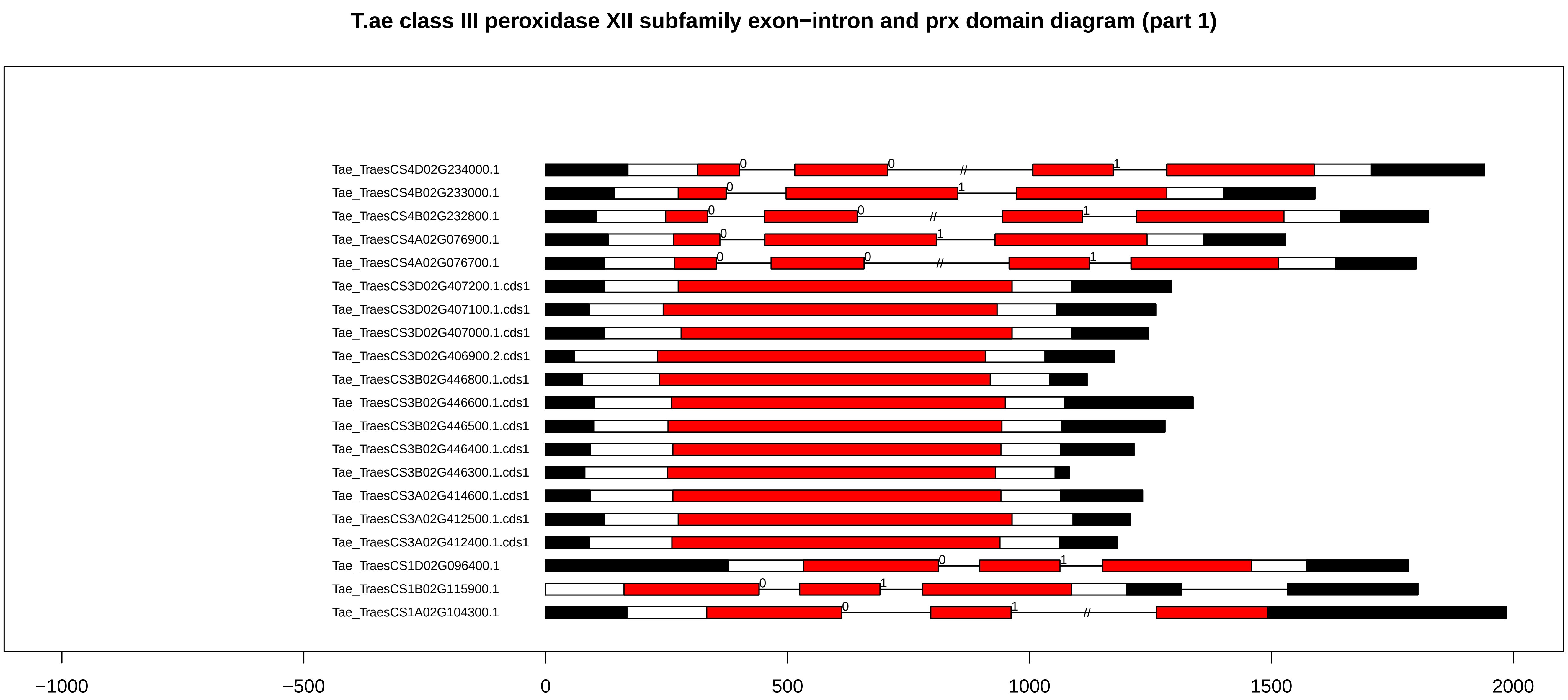

T.ae class III peroxidase XII subfamily exon−intron and prx domain diagram (part 1)
Tae_TraesCS4D02G234000.1
Tae_TraesCS4B02G233000.1
Tae_TraesCS4B02G232800.1
Tae_TraesCS4A02G076900.1
Tae_TraesCS4A02G076700.1
Tae_TraesCS3D02G407200.1.cds1
Tae_TraesCS3D02G407100.1.cds1
Tae_TraesCS3D02G407000.1.cds1
Tae_TraesCS3D02G406900.2.cds1
Tae_TraesCS3B02G446800.1.cds1
Tae_TraesCS3B02G446600.1.cds1
Tae_TraesCS3B02G446500.1.cds1
Tae_TraesCS3B02G446400.1.cds1
Tae_TraesCS3B02G446300.1.cds1
Tae_TraesCS3A02G414600.1.cds1
Tae_TraesCS3A02G412500.1.cds1
Tae_TraesCS3A02G412400.1.cds1
Tae_TraesCS1D02G096400.1
Tae_TraesCS1B02G115900.1
Tae_TraesCS1A02G104300.1
−1000
−500
0
500
1000
1500
2000

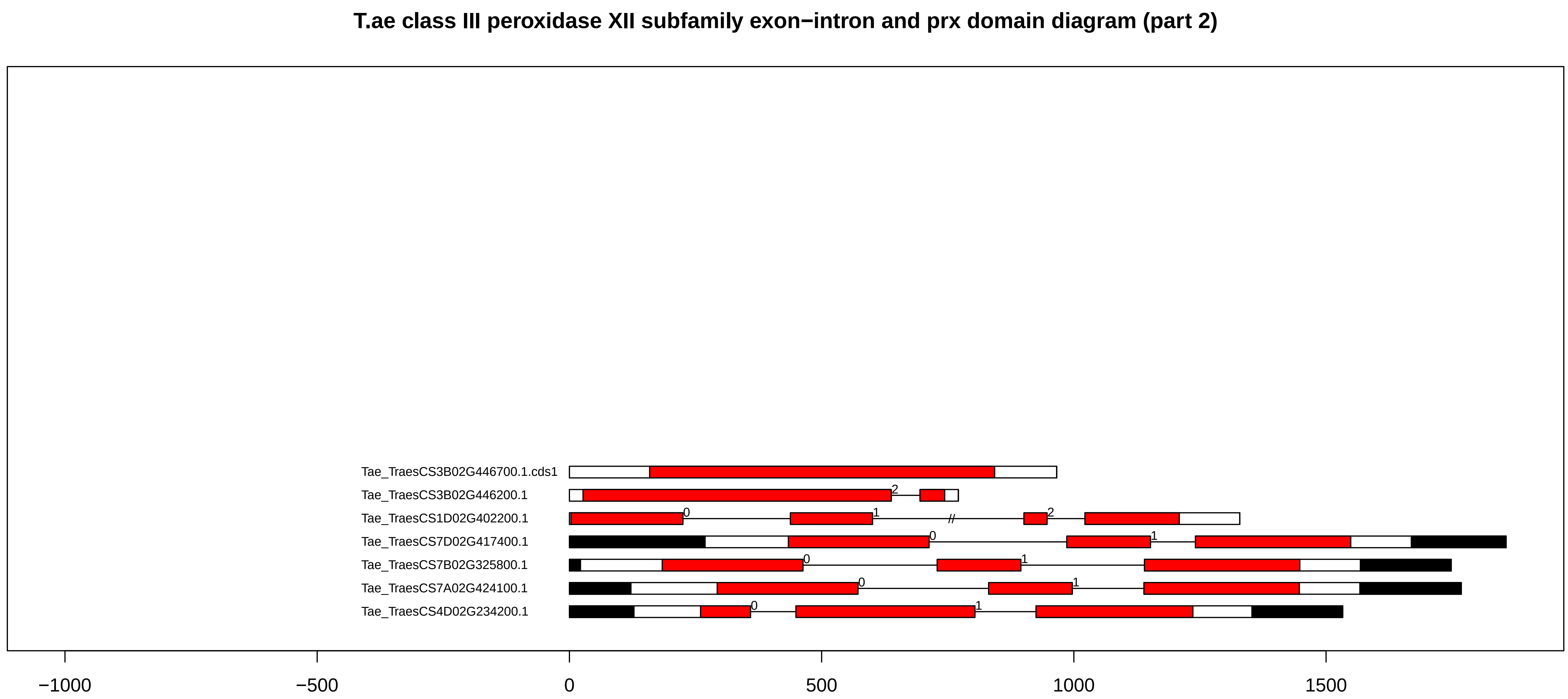
T.ae class III peroxidase XII subfamily exon−intron and prx domain diagram (part 2)
Tae_TraesCS3B02G446700.1.cds1
Tae_TraesCS3B02G446200.1
Tae_TraesCS1D02G402200.1
Tae_TraesCS7D02G417400.1
Tae_TraesCS7B02G325800.1
Tae_TraesCS7A02G424100.1
Tae_TraesCS4D02G234200.1
−1000
−500
0
500
1000
1500

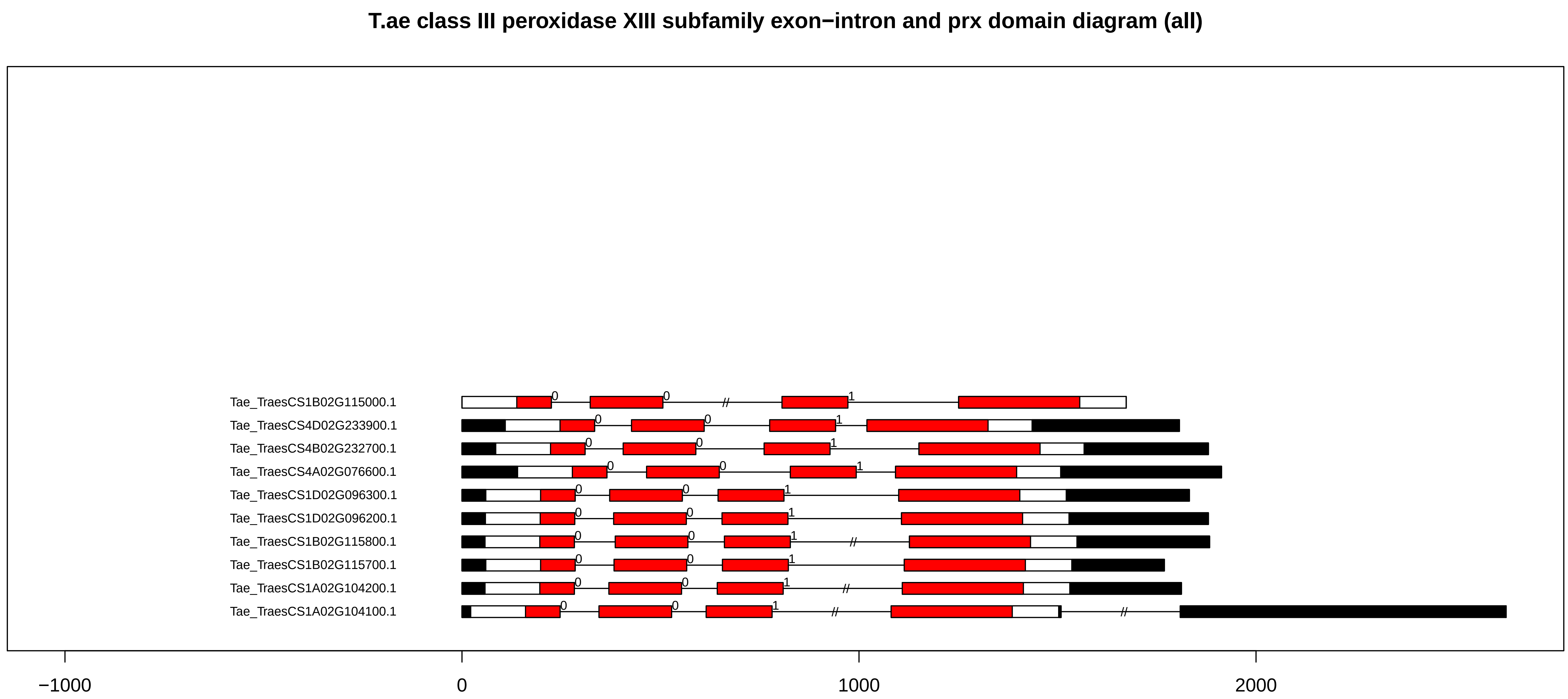
T.ae class III peroxidase XIII subfamily exon−intron and prx domain diagram (all)
Tae_TraesCS1B02G115000.1
Tae_TraesCS4D02G233900.1
Tae_TraesCS4B02G232700.1
Tae_TraesCS4A02G076600.1
Tae_TraesCS1D02G096300.1
Tae_TraesCS1D02G096200.1
Tae_TraesCS1B02G115800.1
Tae_TraesCS1B02G115700.1
Tae_TraesCS1A02G104200.1
Tae_TraesCS1A02G104100.1
−1000
0
1000
2000

T.ae class III peroxidase XIV subfamily exon−intron and prx domain diagram (part 1)

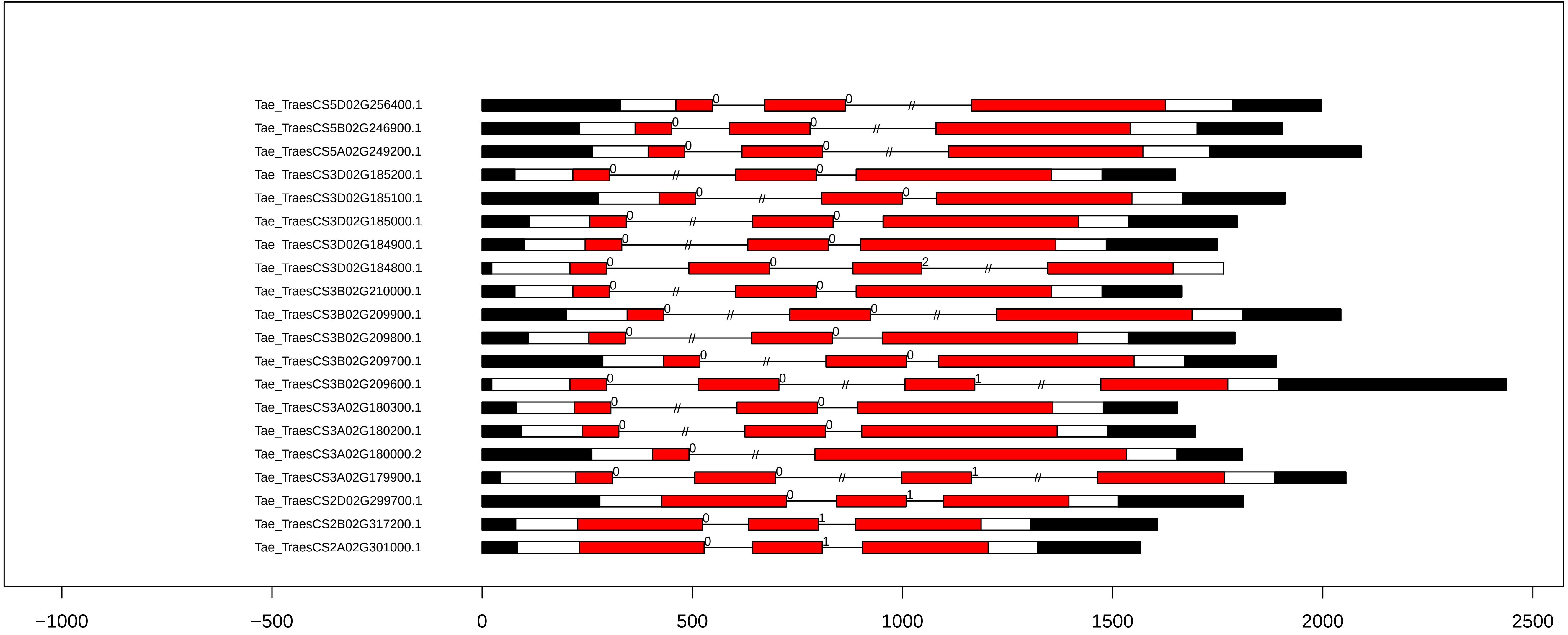

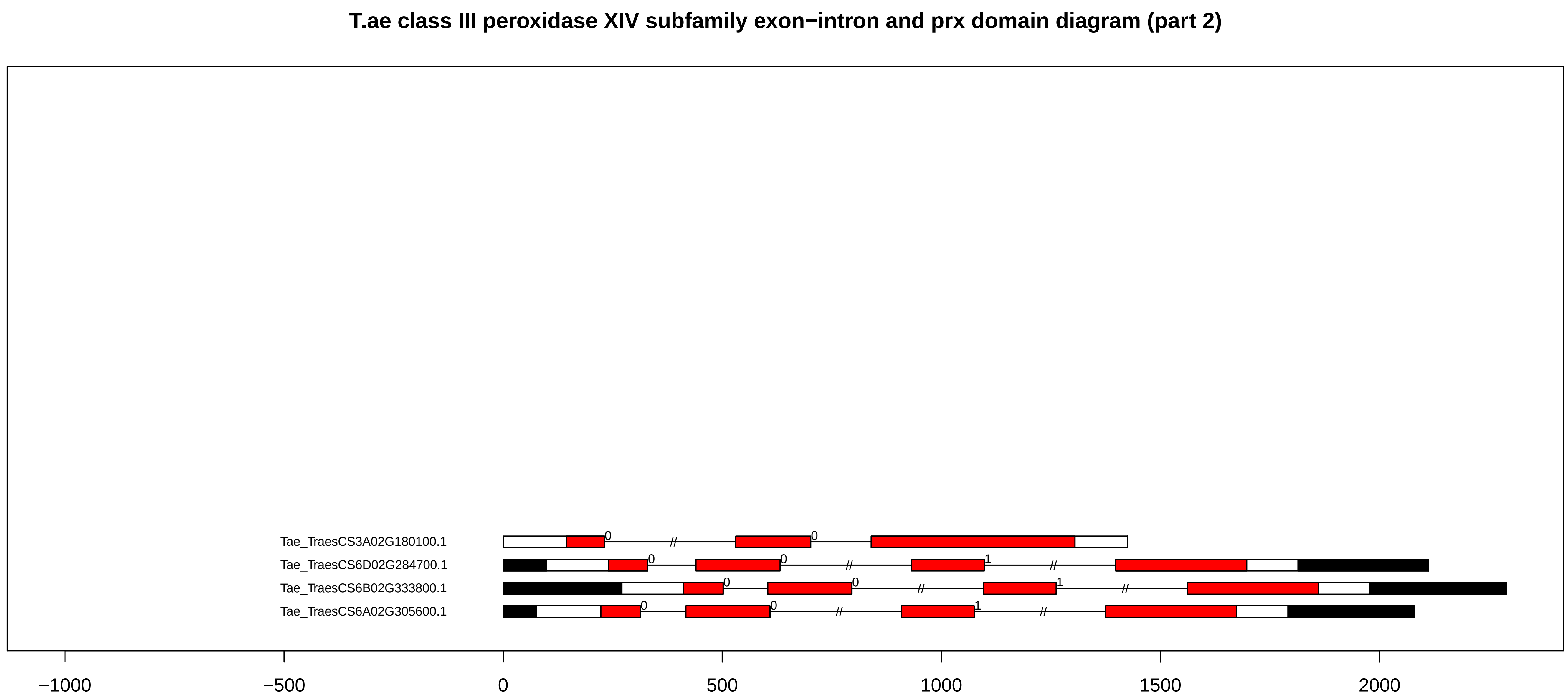
T.ae class III peroxidase XIV subfamily exon−intron and prx domain diagram (part 2)
Tae_TraesCS3A02G180100.1
Tae_TraesCS6D02G284700.1
Tae_TraesCS6B02G333800.1
Tae_TraesCS6A02G305600.1
−1000
−500
0
500
1000
1500
2000

T.ae class III peroxidase XV subfamily exon−intron and prx domain diagram (all)

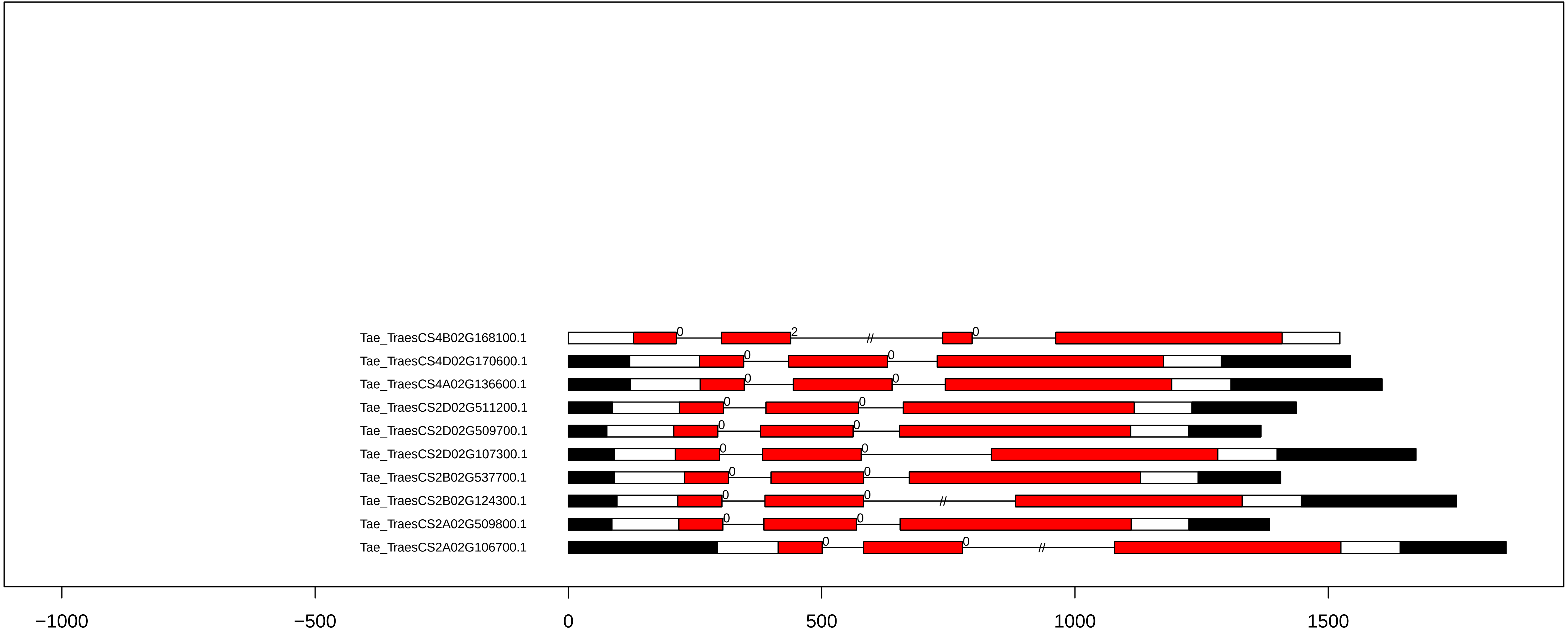

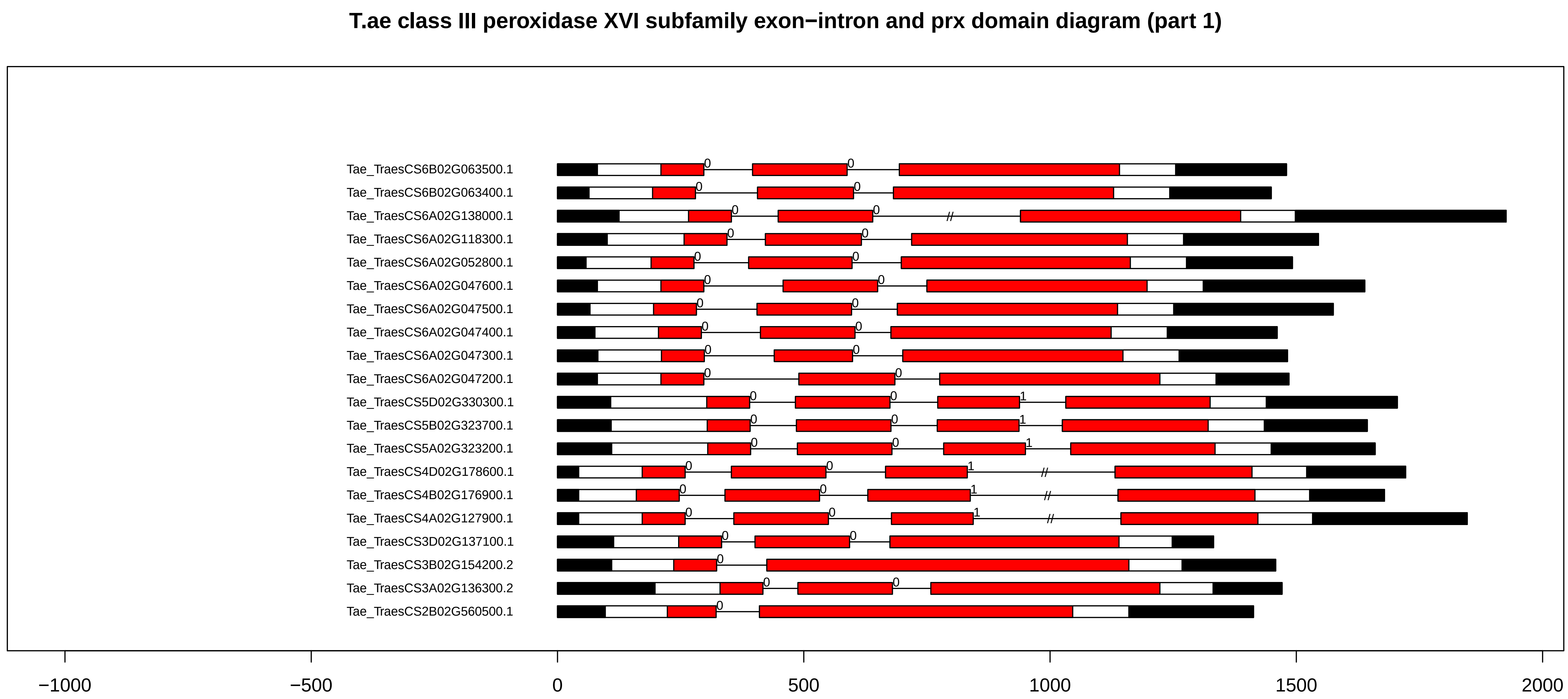

T.ae class III peroxidase XVI subfamily exon−intron and prx domain diagram (part 1)
Tae_TraesCS6B02G063500.1
Tae_TraesCS6B02G063400.1
Tae_TraesCS6A02G138000.1
Tae_TraesCS6A02G118300.1
Tae_TraesCS6A02G052800.1
Tae_TraesCS6A02G047600.1
Tae_TraesCS6A02G047500.1
Tae_TraesCS6A02G047400.1
Tae_TraesCS6A02G047300.1
Tae_TraesCS6A02G047200.1
Tae_TraesCS5D02G330300.1
Tae_TraesCS5B02G323700.1
Tae_TraesCS5A02G323200.1
Tae_TraesCS4D02G178600.1
Tae_TraesCS4B02G176900.1
Tae_TraesCS4A02G127900.1
Tae_TraesCS3D02G137100.1
Tae_TraesCS3B02G154200.2
Tae_TraesCS3A02G136300.2
Tae_TraesCS2B02G560500.1
−1000
−500
0
500
1000
1500
2000

T.ae class III peroxidase XVI subfamily exon−intron and prx domain diagram (part 2)

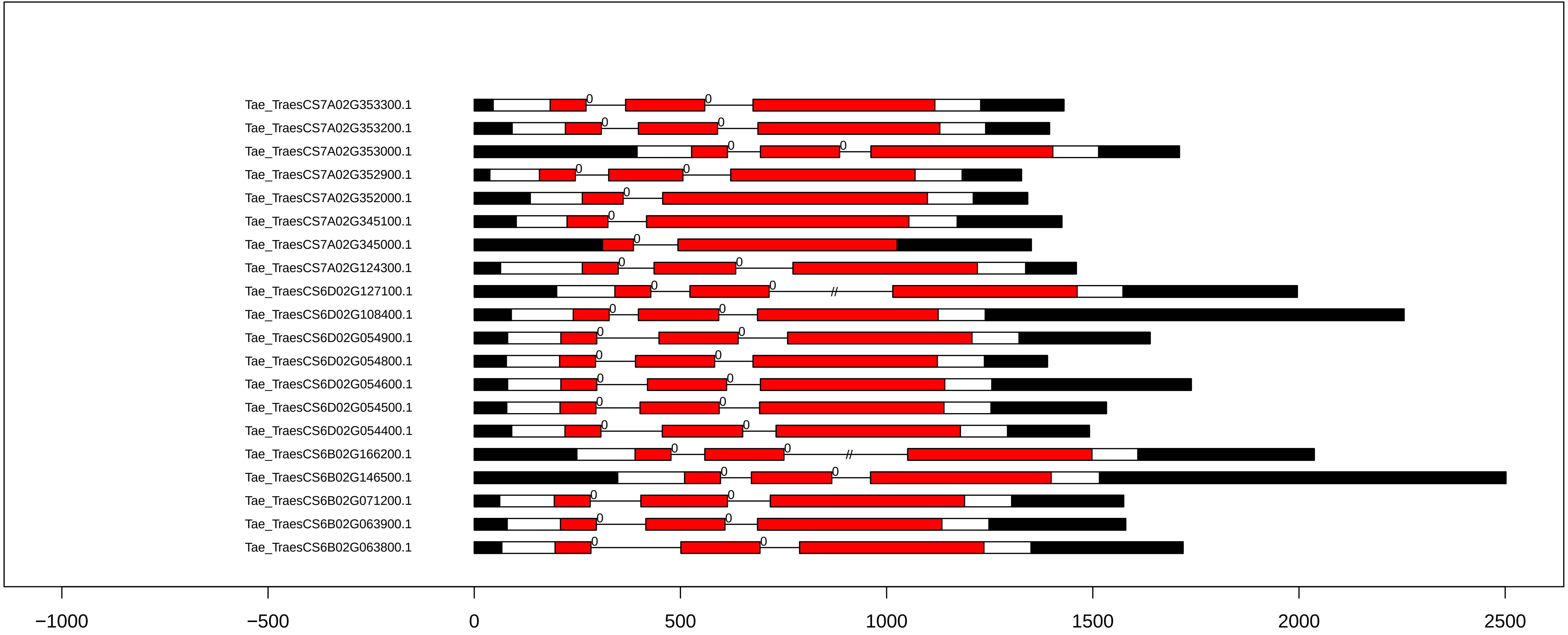

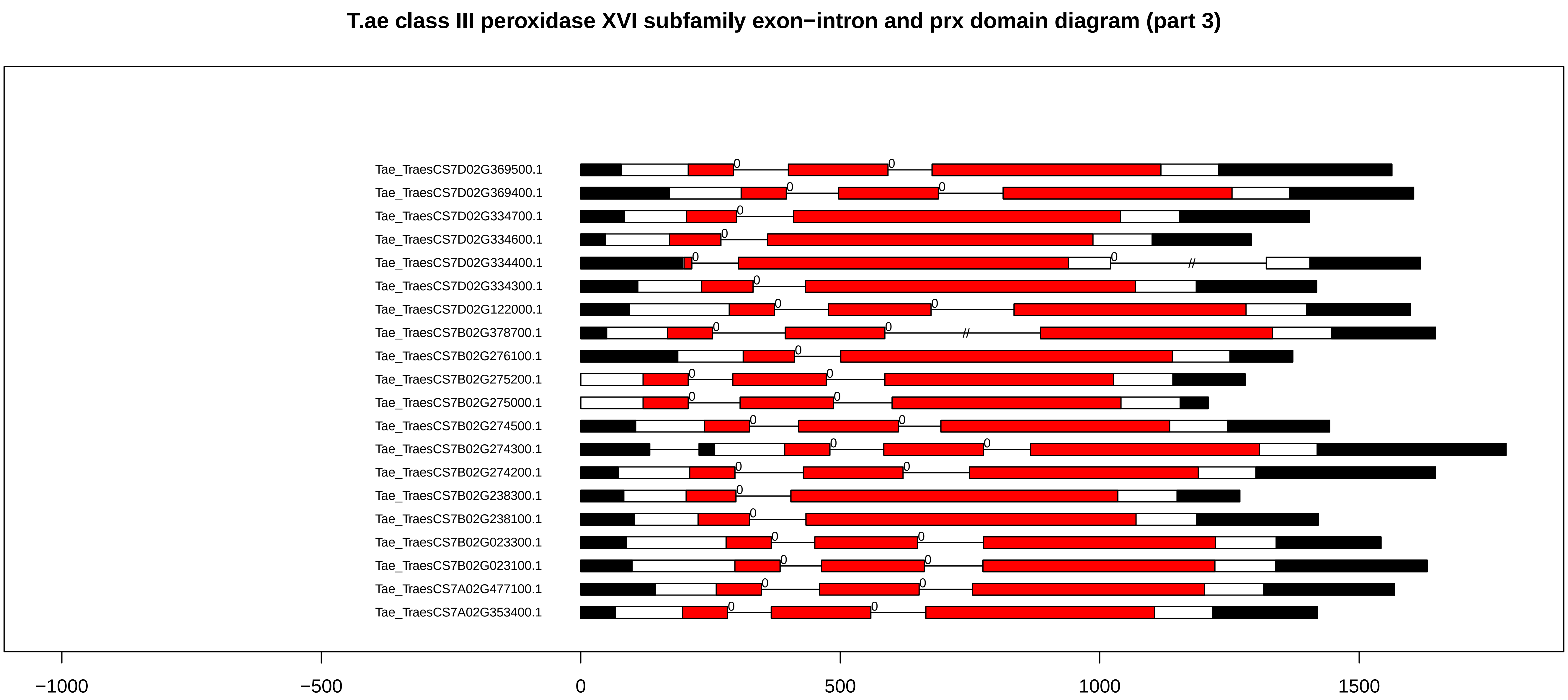

T.ae class III peroxidase XVI subfamily exon−intron and prx domain diagram (part 3)
Tae_TraesCS7D02G369500.1
Tae_TraesCS7D02G369400.1
Tae_TraesCS7D02G334700.1
Tae_TraesCS7D02G334600.1
Tae_TraesCS7D02G334400.1
Tae_TraesCS7D02G334300.1
Tae_TraesCS7D02G122000.1
Tae_TraesCS7B02G378700.1
Tae_TraesCS7B02G276100.1
Tae_TraesCS7B02G275200.1
Tae_TraesCS7B02G275000.1
Tae_TraesCS7B02G274500.1
Tae_TraesCS7B02G274300.1
Tae_TraesCS7B02G274200.1
Tae_TraesCS7B02G238300.1
Tae_TraesCS7B02G238100.1
Tae_TraesCS7B02G023300.1
Tae_TraesCS7B02G023100.1
Tae_TraesCS7A02G477100.1
Tae_TraesCS7A02G353400.1
−1000
−500
0
500
1000
1500

T.ae class III peroxidase XVI subfamily exon−intron and prx domain diagram (part 4)

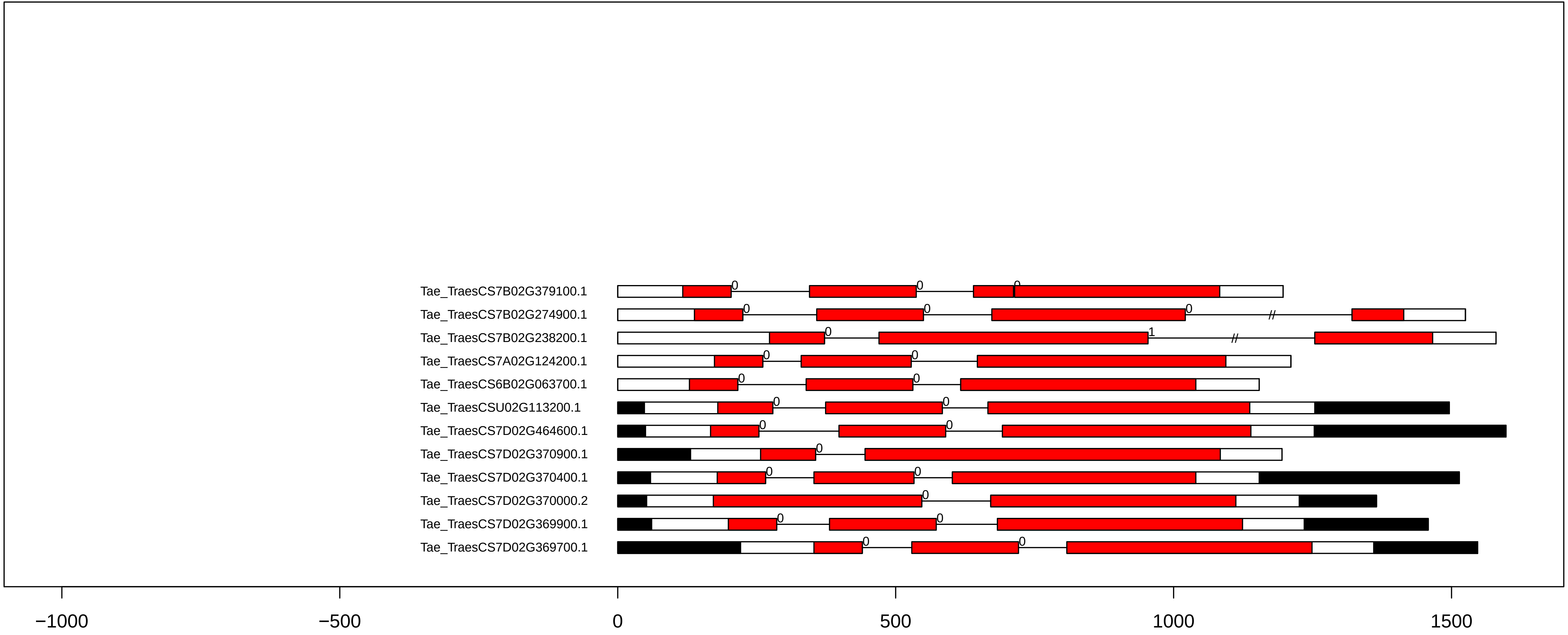

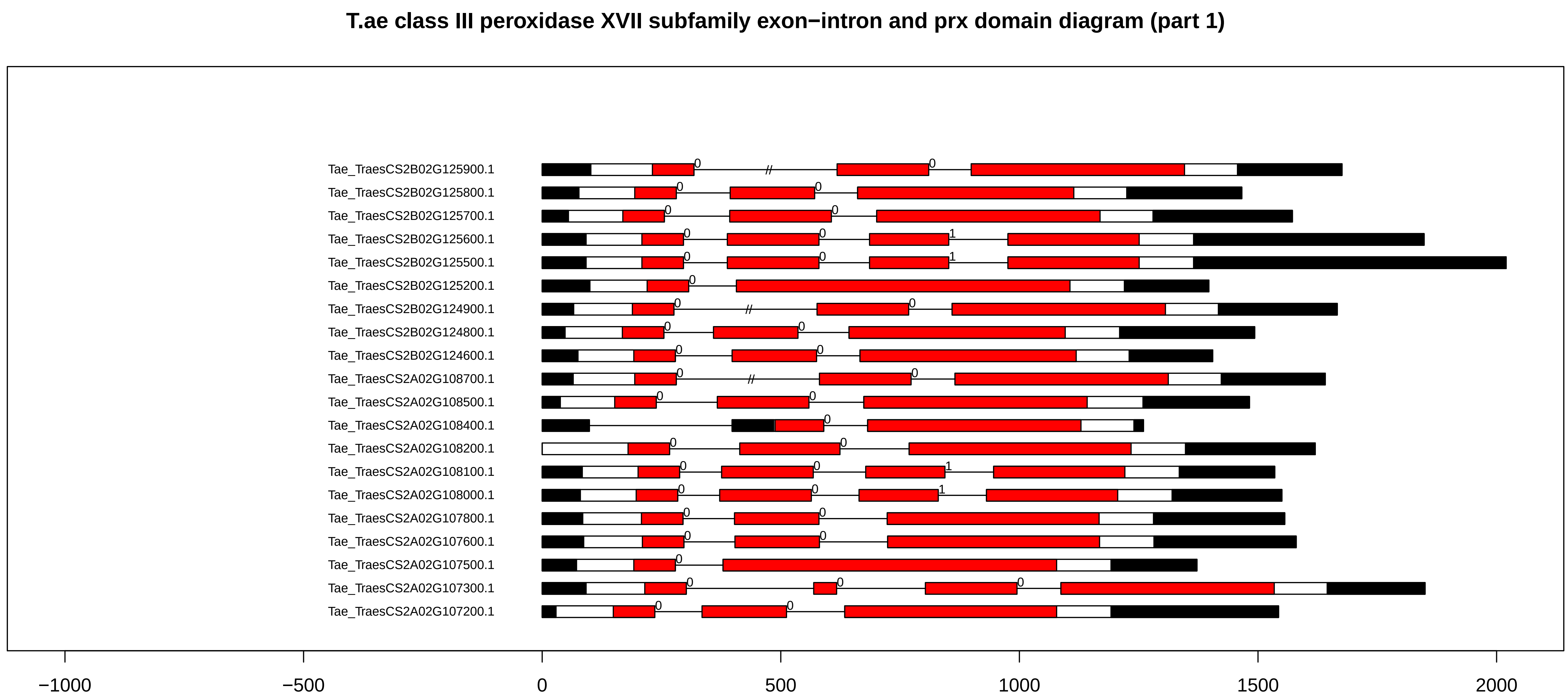
T.ae class III peroxidase XVII subfamily exon−intron and prx domain diagram (part 1)
Tae_TraesCS2B02G125900.1
Tae_TraesCS2B02G125800.1
Tae_TraesCS2B02G125700.1
Tae_TraesCS2B02G125600.1
Tae_TraesCS2B02G125500.1
Tae_TraesCS2B02G125200.1
Tae_TraesCS2B02G124900.1
Tae_TraesCS2B02G124800.1
Tae_TraesCS2B02G124600.1
Tae_TraesCS2A02G108700.1
Tae_TraesCS2A02G108500.1
Tae_TraesCS2A02G108400.1
Tae_TraesCS2A02G108200.1
Tae_TraesCS2A02G108100.1
Tae_TraesCS2A02G108000.1
Tae_TraesCS2A02G107800.1
Tae_TraesCS2A02G107600.1
Tae_TraesCS2A02G107500.1
Tae_TraesCS2A02G107300.1
Tae_TraesCS2A02G107200.1
−1000
−500
0
500
1000
1500
2000

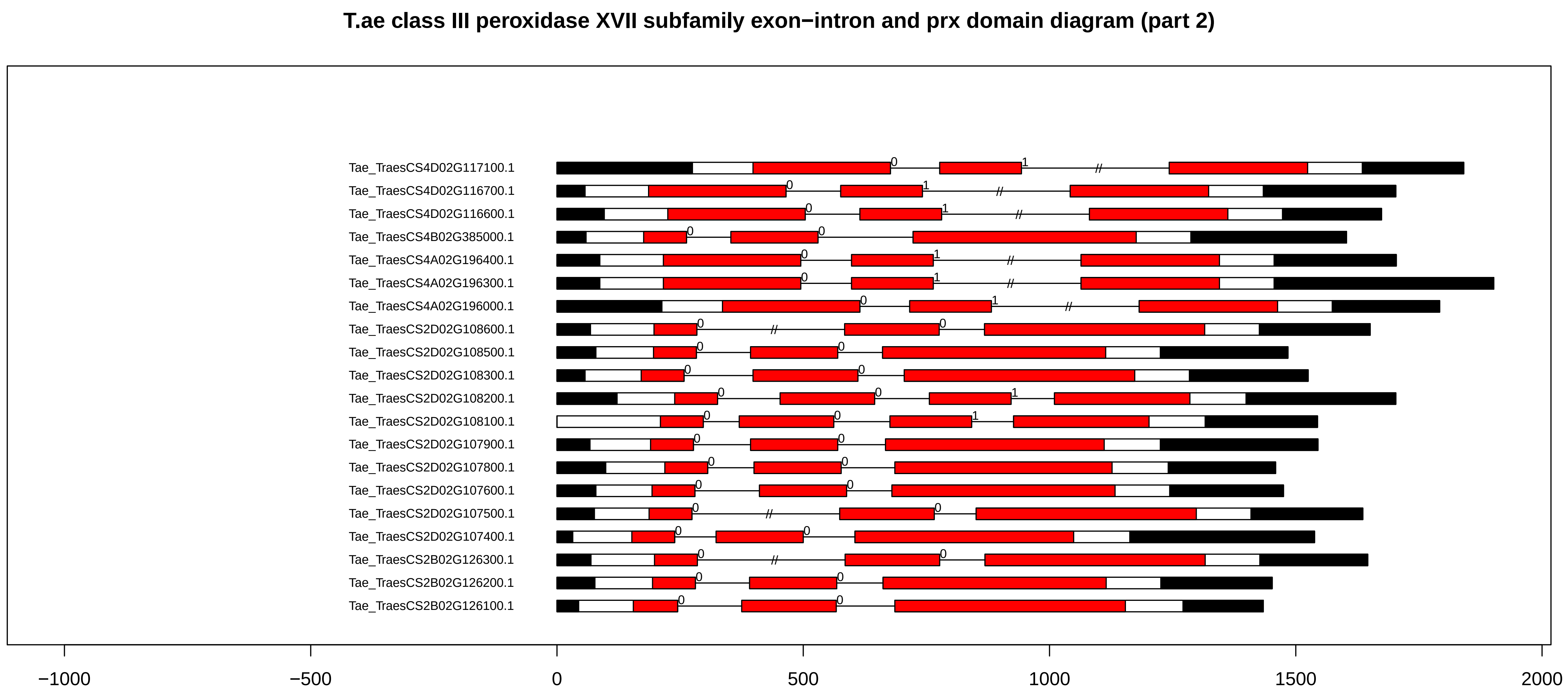
T.ae class III peroxidase XVII subfamily exon−intron and prx domain diagram (part 2)
Tae_TraesCS4D02G117100.1
Tae_TraesCS4D02G116700.1
Tae_TraesCS4D02G116600.1
Tae_TraesCS4B02G385000.1
Tae_TraesCS4A02G196400.1
Tae_TraesCS4A02G196300.1
Tae_TraesCS4A02G196000.1
Tae_TraesCS2D02G108600.1
Tae_TraesCS2D02G108500.1
Tae_TraesCS2D02G108300.1
Tae_TraesCS2D02G108200.1
Tae_TraesCS2D02G108100.1
Tae_TraesCS2D02G107900.1
Tae_TraesCS2D02G107800.1
Tae_TraesCS2D02G107600.1
Tae_TraesCS2D02G107500.1
Tae_TraesCS2D02G107400.1
Tae_TraesCS2B02G126300.1
Tae_TraesCS2B02G126200.1
Tae_TraesCS2B02G126100.1
−1000
−500
0
500
1000
1500
2000

T.ae class III peroxidase XVII subfamily exon−intron and prx domain diagram (part 3)

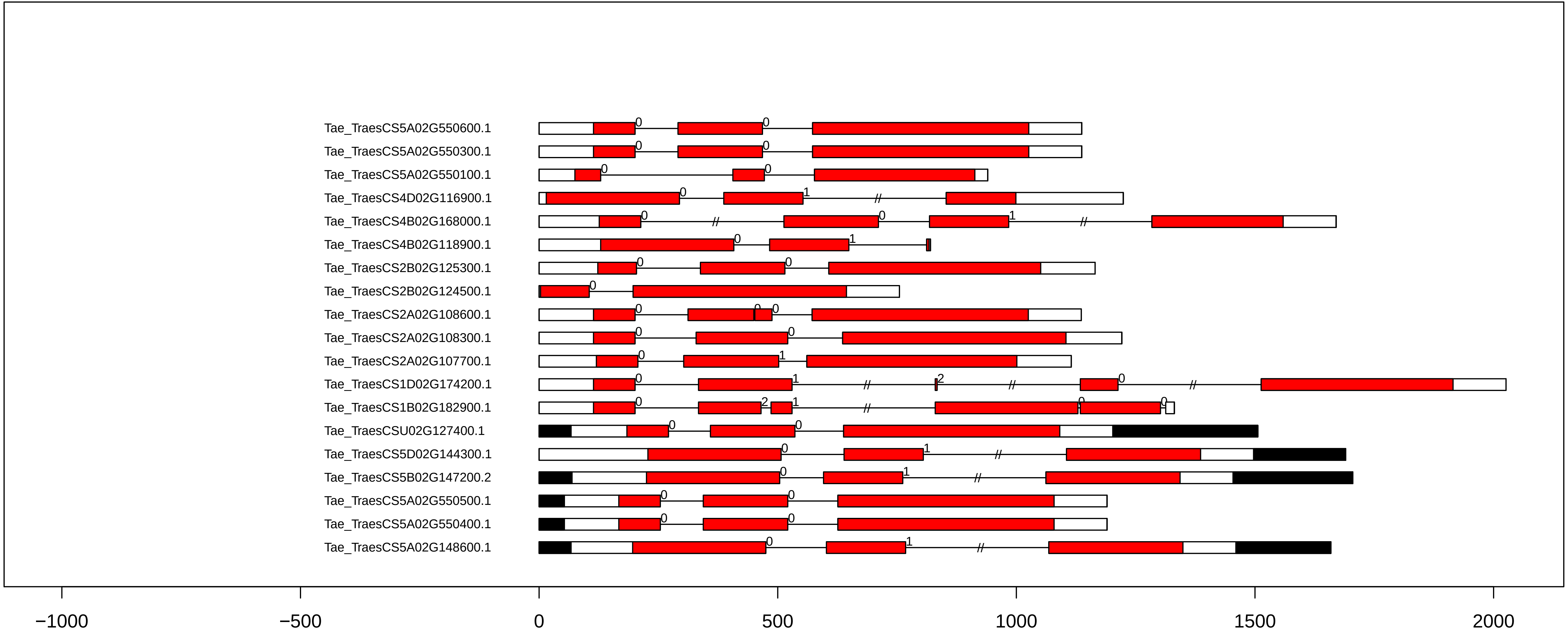

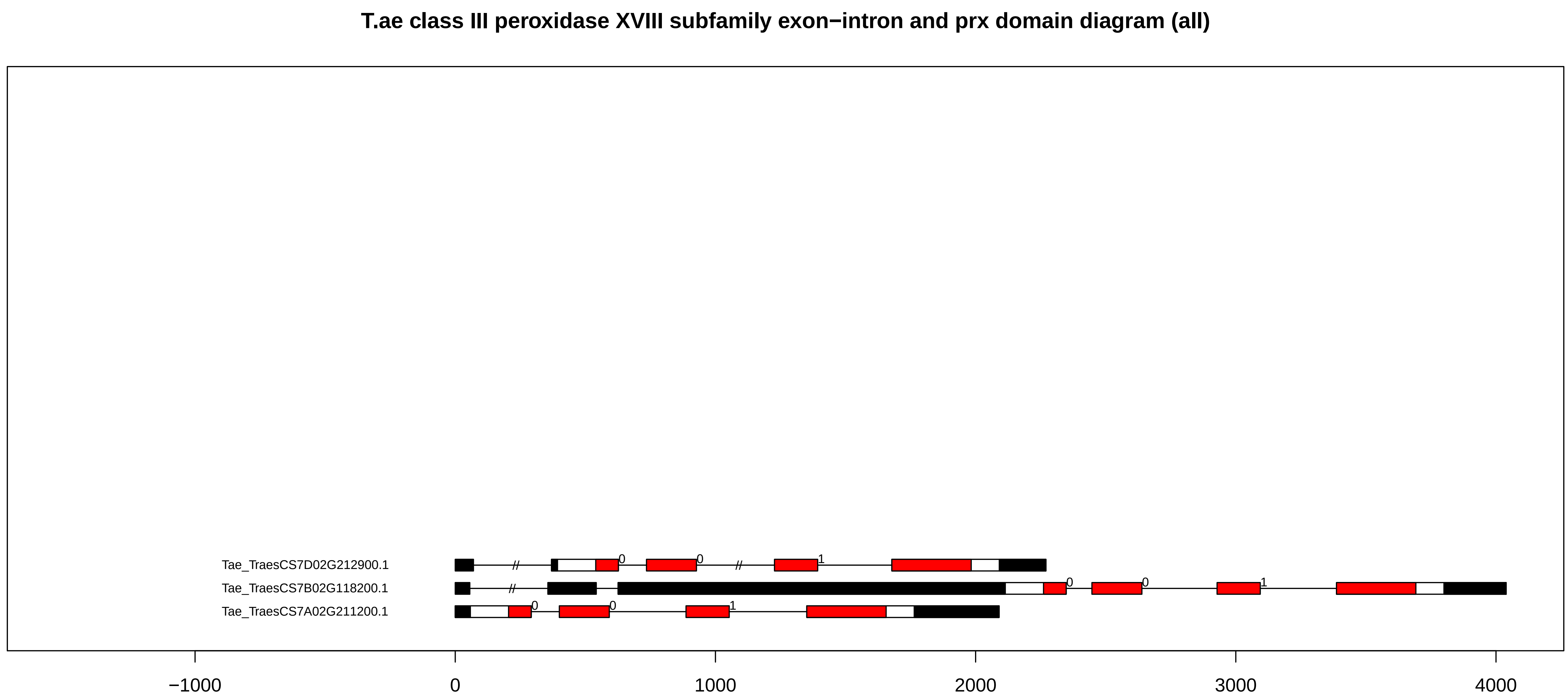
T.ae class III peroxidase XVIII subfamily exon−intron and prx domain diagram (all)
Tae_TraesCS7D02G212900.1
Tae_TraesCS7B02G118200.1
Tae_TraesCS7A02G211200.1
0
0
1
0
0
1
0
0
1
−1000
0
1000
2000
3000
4000

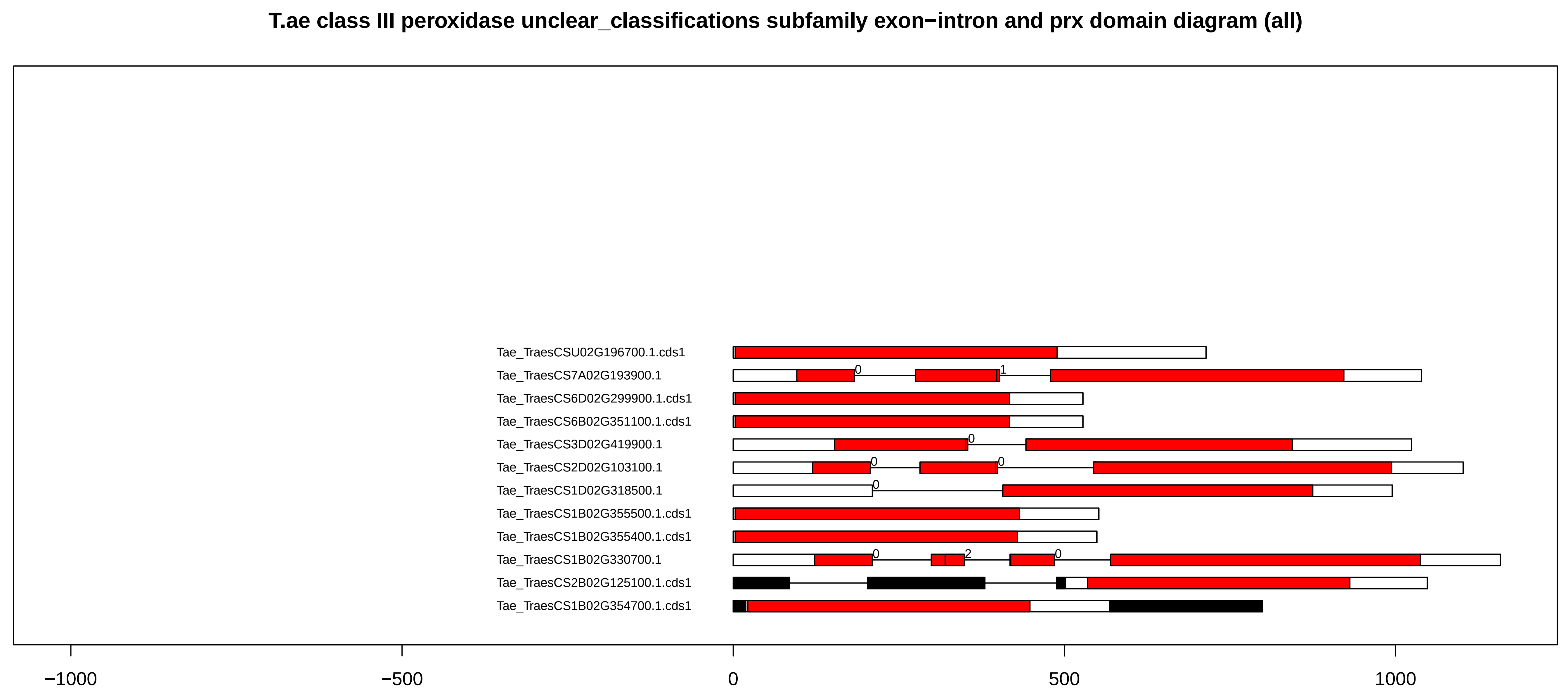

T.ae class III peroxidase unclear_classifications subfamily exon−intron and prx domain diagram (all)
Tae_TraesCSU02G196700.1.cds1
Tae_TraesCS7A02G193900.1
Tae_TraesCS6D02G299900.1.cds1
Tae_TraesCS6B02G351100.1.cds1
Tae_TraesCS3D02G419900.1
Tae_TraesCS2D02G103100.1
Tae_TraesCS1D02G318500.1
Tae_TraesCS1B02G355500.1.cds1
Tae_TraesCS1B02G355400.1.cds1
Tae_TraesCS1B02G330700.1
Tae_TraesCS2B02G125100.1.cds1
Tae_TraesCS1B02G354700.1.cds1
−1000
−500
0
500
1000

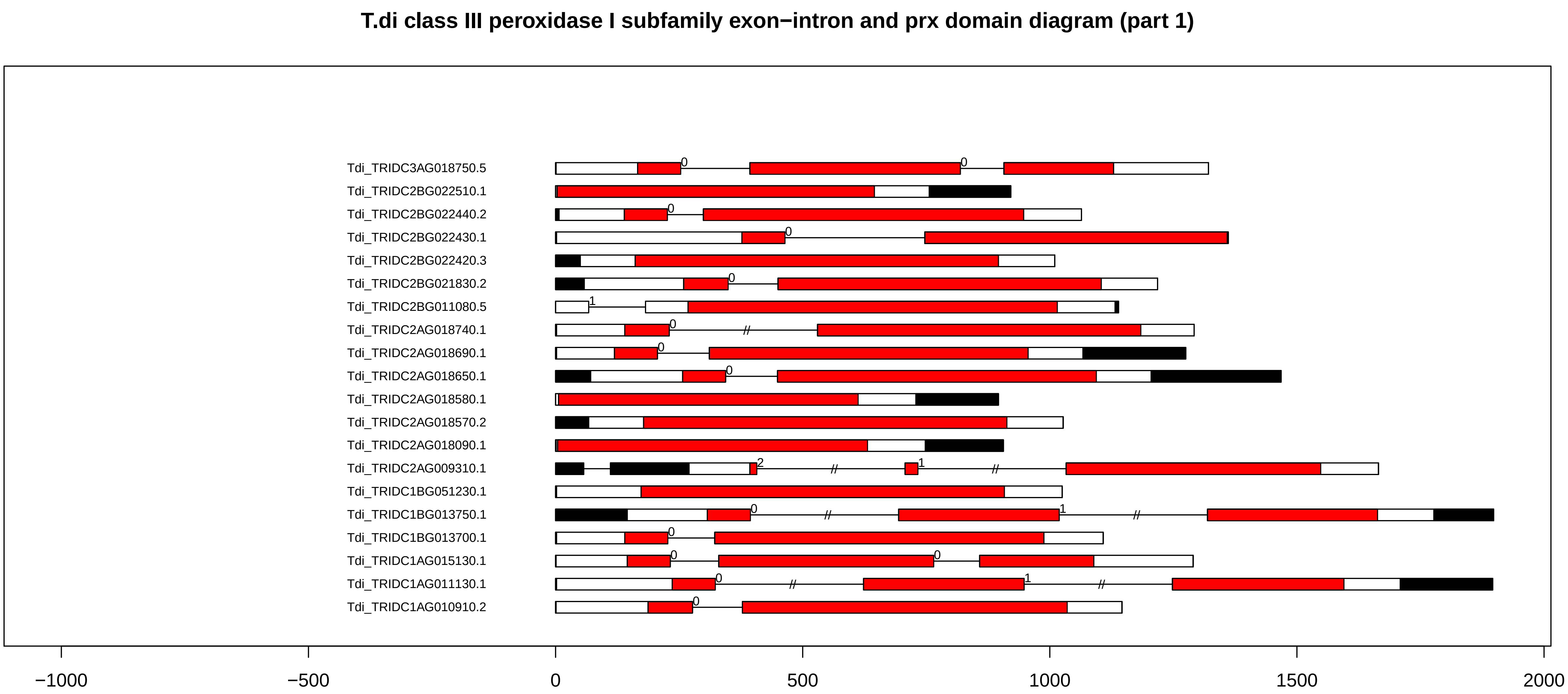
T.di class III peroxidase I subfamily exon−intron and prx domain diagram (part 1)
Tdi_TRIDC3AG018750.5
Tdi_TRIDC2BG022510.1
Tdi_TRIDC2BG022440.2
Tdi_TRIDC2BG022430.1
Tdi_TRIDC2BG022420.3
Tdi_TRIDC2BG021830.2
Tdi_TRIDC2BG011080.5
Tdi_TRIDC2AG018740.1
Tdi_TRIDC2AG018690.1
Tdi_TRIDC2AG018650.1
Tdi_TRIDC2AG018580.1
Tdi_TRIDC2AG018570.2
Tdi_TRIDC2AG018090.1
Tdi_TRIDC2AG009310.1
Tdi_TRIDC1BG051230.1
Tdi_TRIDC1BG013750.1
Tdi_TRIDC1BG013700.1
Tdi_TRIDC1AG015130.1
Tdi_TRIDC1AG011130.1
Tdi_TRIDC1AG010910.2
−1000
−500
0
500
1000
1500
2000

T.di class III peroxidase I subfamily exon−intron and prx domain diagram (part 2)

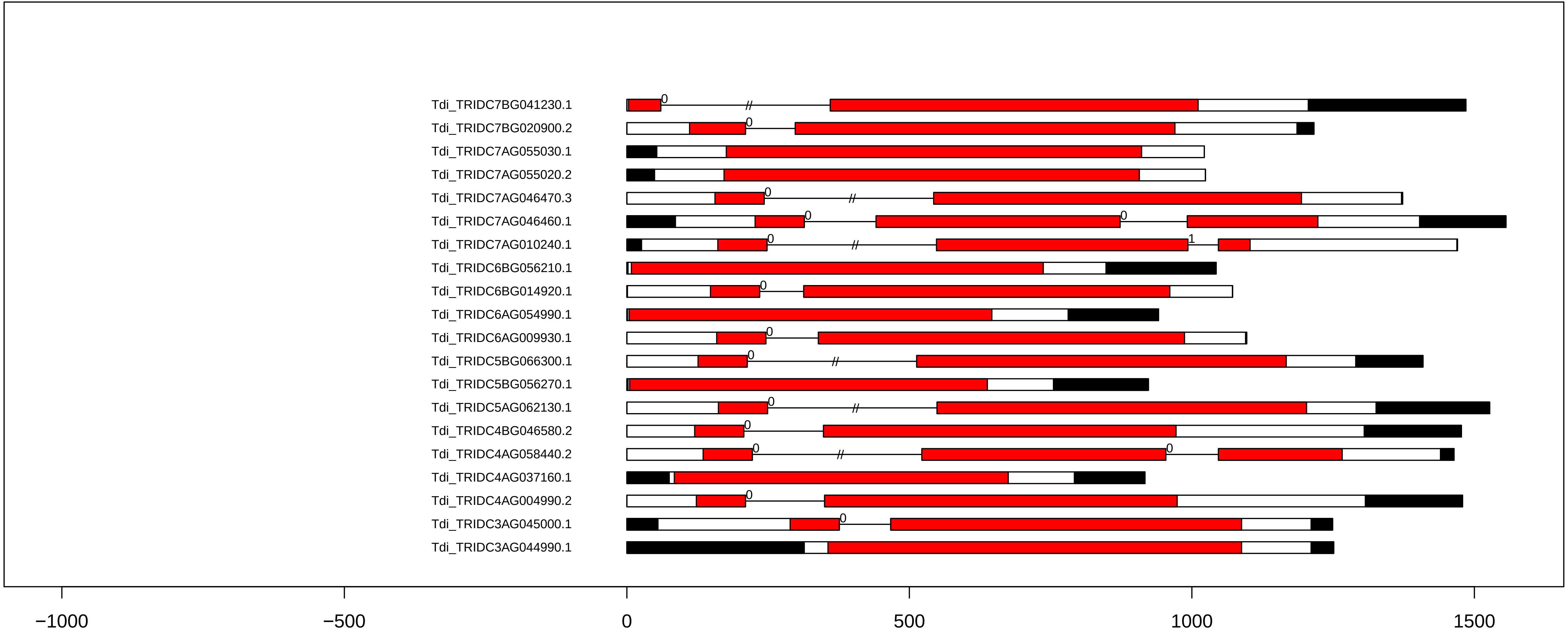

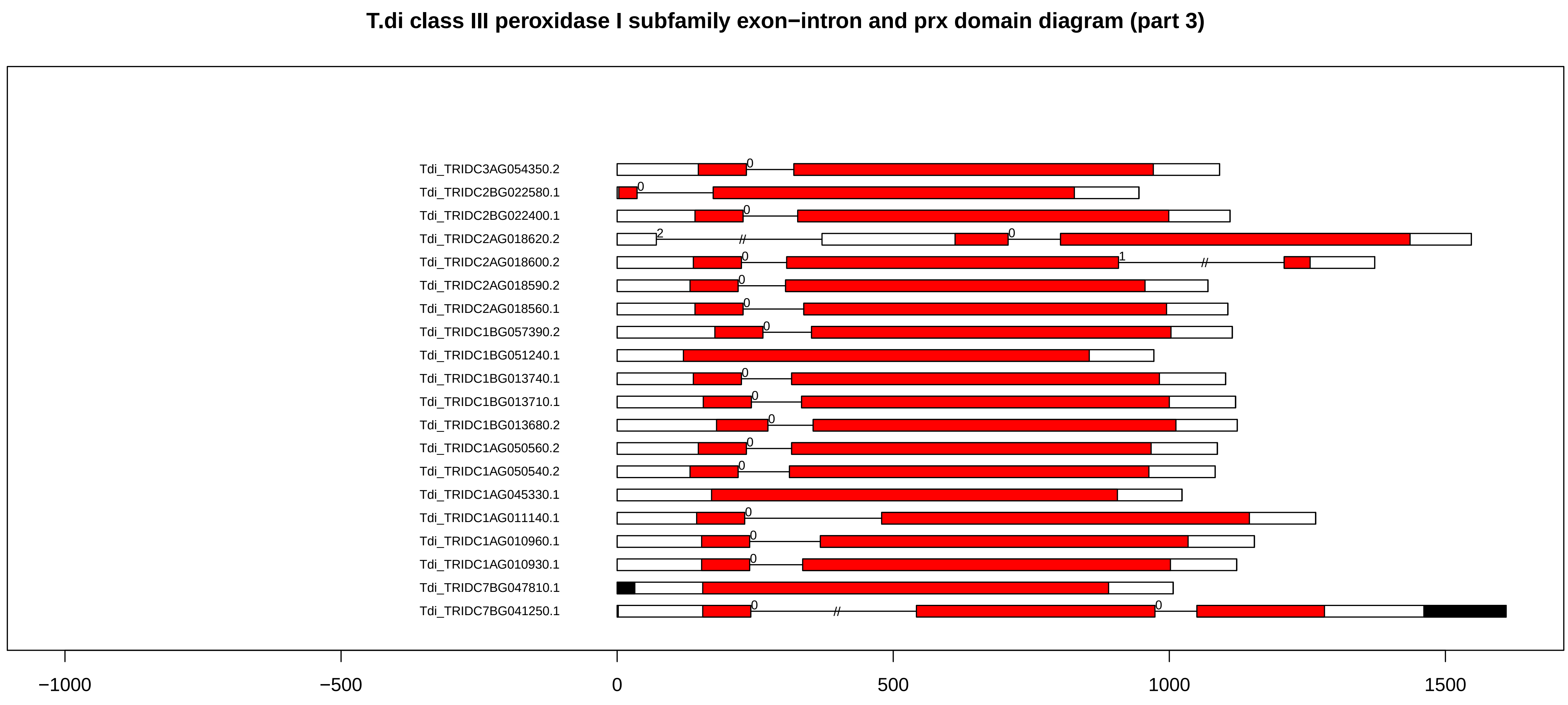
T.di class III peroxidase I subfamily exon−intron and prx domain diagram (part 3)
Tdi_TRIDC3AG054350.2
Tdi_TRIDC2BG022580.1
Tdi_TRIDC2BG022400.1
Tdi_TRIDC2AG018620.2
Tdi_TRIDC2AG018600.2
Tdi_TRIDC2AG018590.2
Tdi_TRIDC2AG018560.1
Tdi_TRIDC1BG057390.2
Tdi_TRIDC1BG051240.1
Tdi_TRIDC1BG013740.1
Tdi_TRIDC1BG013710.1
Tdi_TRIDC1BG013680.2
Tdi_TRIDC1AG050560.2
Tdi_TRIDC1AG050540.2
Tdi_TRIDC1AG045330.1
Tdi_TRIDC1AG011140.1
Tdi_TRIDC1AG010960.1
Tdi_TRIDC1AG010930.1
Tdi_TRIDC7BG047810.1
Tdi_TRIDC7BG041250.1
−1000
−500
0
500
1000
1500

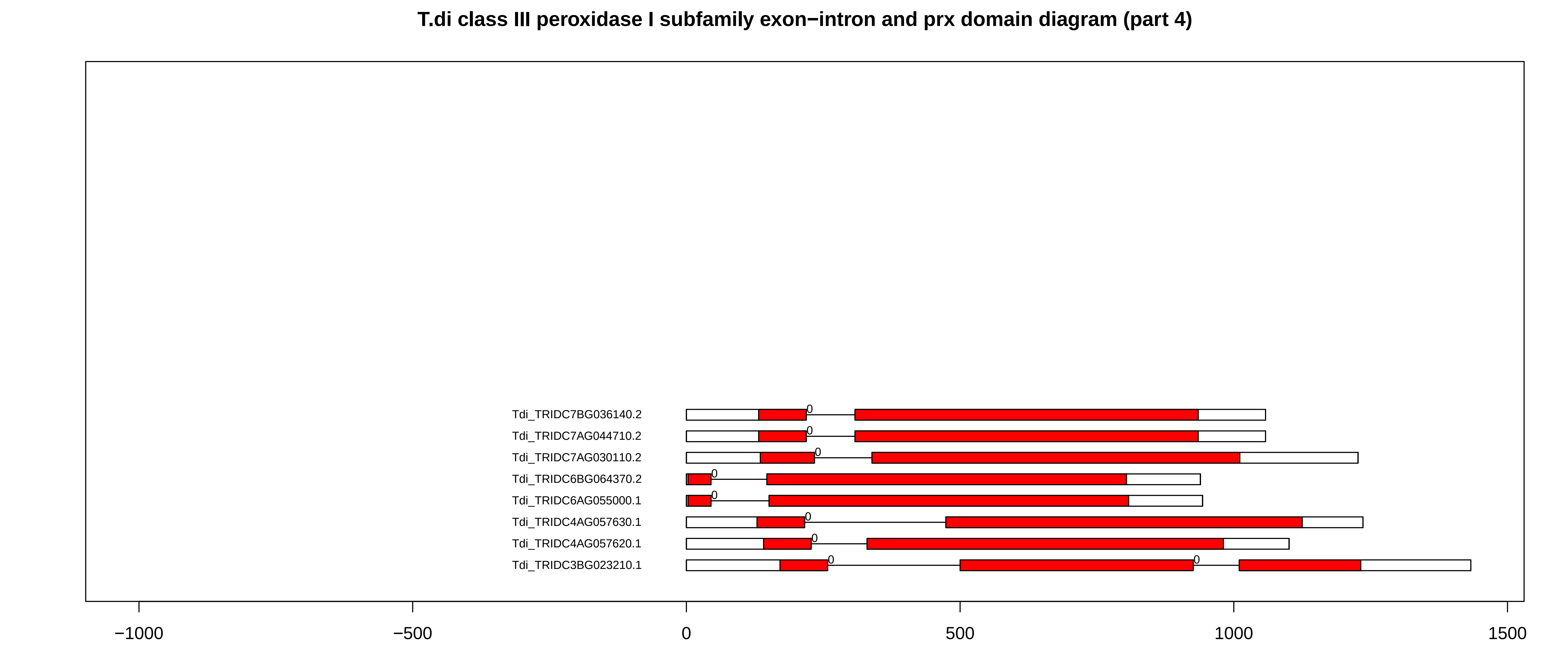

T.di class III peroxidase I subfamily exon−intron and prx domain diagram (part 4)
Tdi_TRIDC7BG036140.2
Tdi_TRIDC7AG044710.2
Tdi_TRIDC7AG030110.2
Tdi_TRIDC6BG064370.2
Tdi_TRIDC6AG055000.1
Tdi_TRIDC4AG057630.1
Tdi_TRIDC4AG057620.1
Tdi_TRIDC3BG023210.1
−1000
−500
0
500
1000
1500

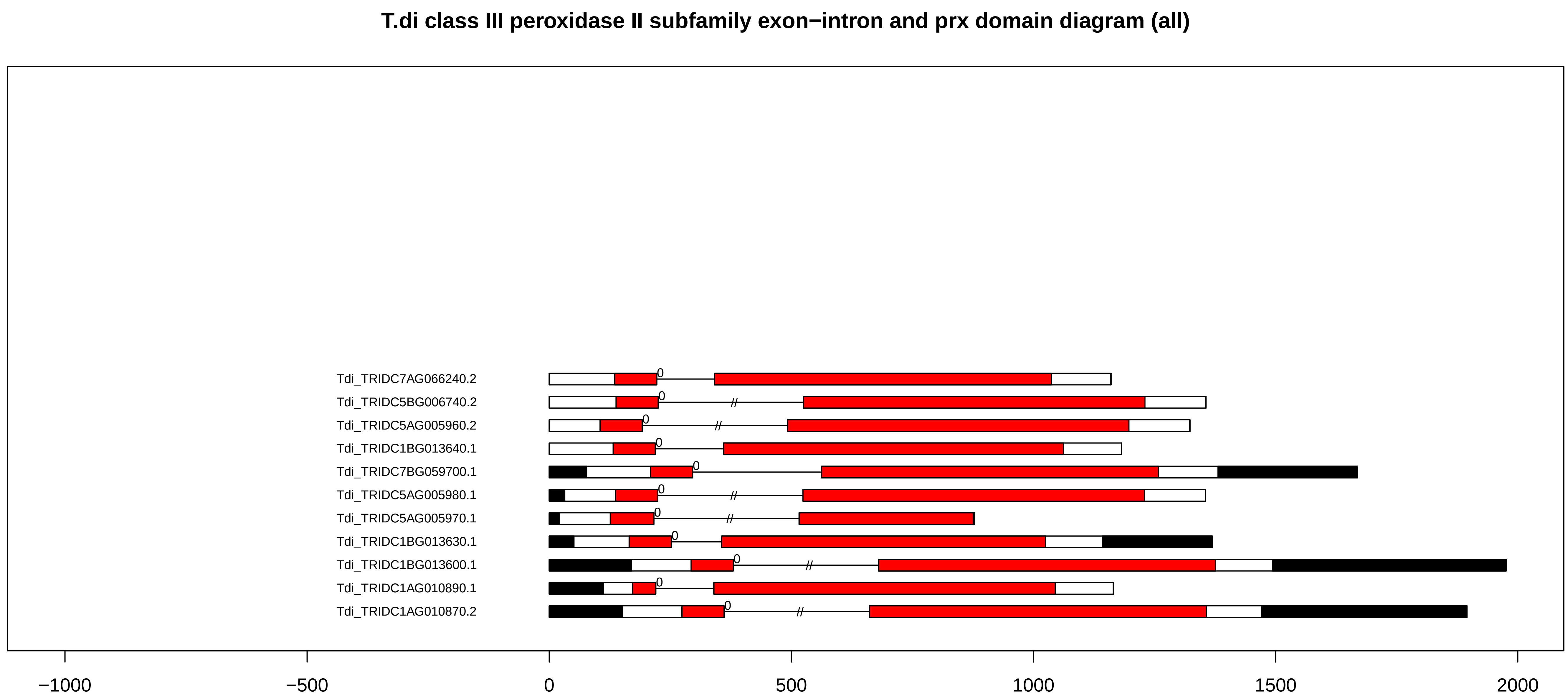
T.di class III peroxidase II subfamily exon−intron and prx domain diagram (all)
Tdi_TRIDC7AG066240.2
Tdi_TRIDC5BG006740.2
Tdi_TRIDC5AG005960.2
Tdi_TRIDC1BG013640.1
Tdi_TRIDC7BG059700.1
Tdi_TRIDC5AG005980.1
Tdi_TRIDC5AG005970.1
Tdi_TRIDC1BG013630.1
Tdi_TRIDC1BG013600.1
Tdi_TRIDC1AG010890.1
Tdi_TRIDC1AG010870.2
−1000
−500
0
500
1000
1500
2000

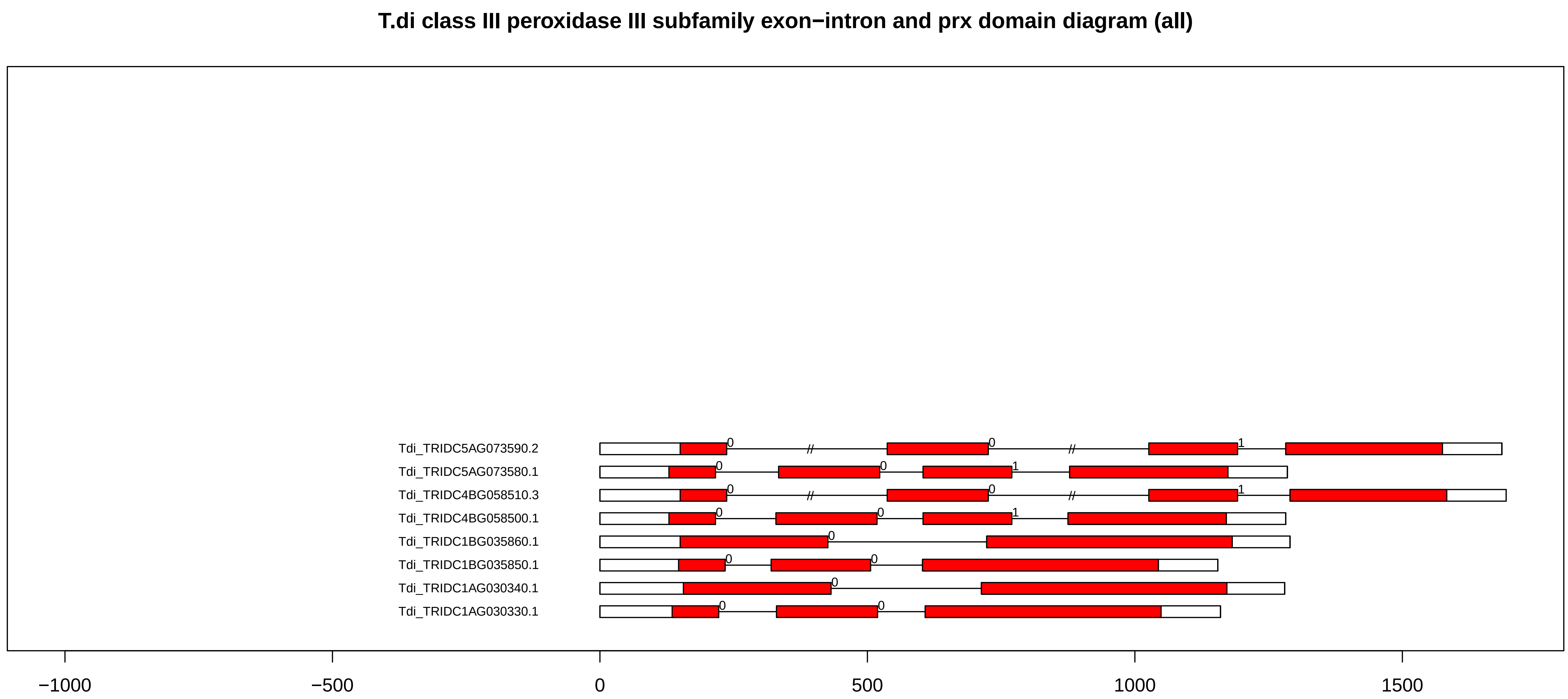

T.di class III peroxidase III subfamily exon−intron and prx domain diagram (all)
Tdi_TRIDC5AG073590.2
Tdi_TRIDC5AG073580.1
Tdi_TRIDC4BG058510.3
Tdi_TRIDC4BG058500.1
Tdi_TRIDC1BG035860.1
Tdi_TRIDC1BG035850.1
Tdi_TRIDC1AG030340.1
Tdi_TRIDC1AG030330.1
−1000
−500
0
500
1000
1500

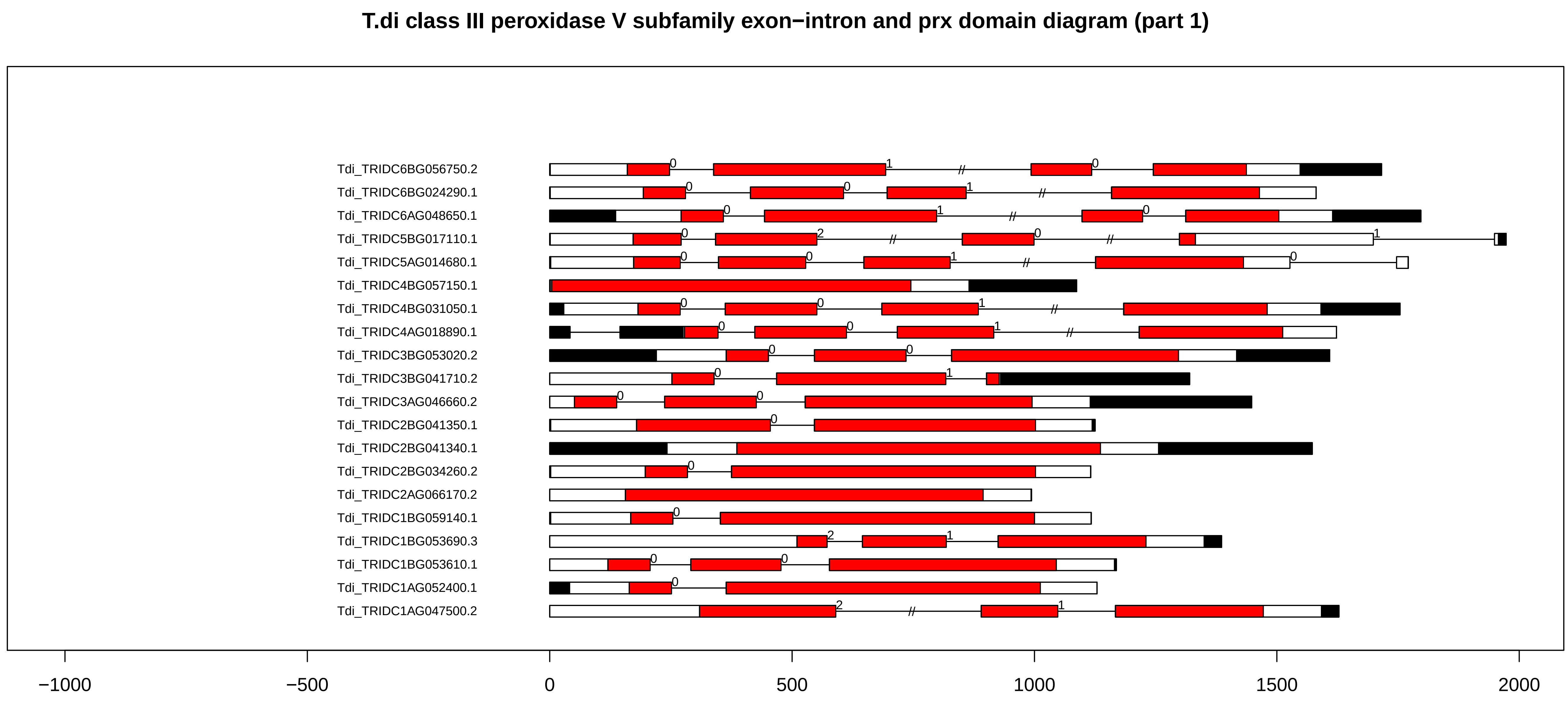

T.di class III peroxidase V subfamily exon−intron and prx domain diagram (part 1)
Tdi_TRIDC6BG056750.2
Tdi_TRIDC6BG024290.1
Tdi_TRIDC6AG048650.1
Tdi_TRIDC5BG017110.1
Tdi_TRIDC5AG014680.1
Tdi_TRIDC4BG057150.1
Tdi_TRIDC4BG031050.1
Tdi_TRIDC4AG018890.1
Tdi_TRIDC3BG053020.2
Tdi_TRIDC3BG041710.2
Tdi_TRIDC3AG046660.2
Tdi_TRIDC2BG041350.1
Tdi_TRIDC2BG041340.1
Tdi_TRIDC2BG034260.2
Tdi_TRIDC2AG066170.2
Tdi_TRIDC1BG059140.1
Tdi_TRIDC1BG053690.3
Tdi_TRIDC1BG053610.1
Tdi_TRIDC1AG052400.1
Tdi_TRIDC1AG047500.2
−1000
−500
0
500
1000
1500
2000

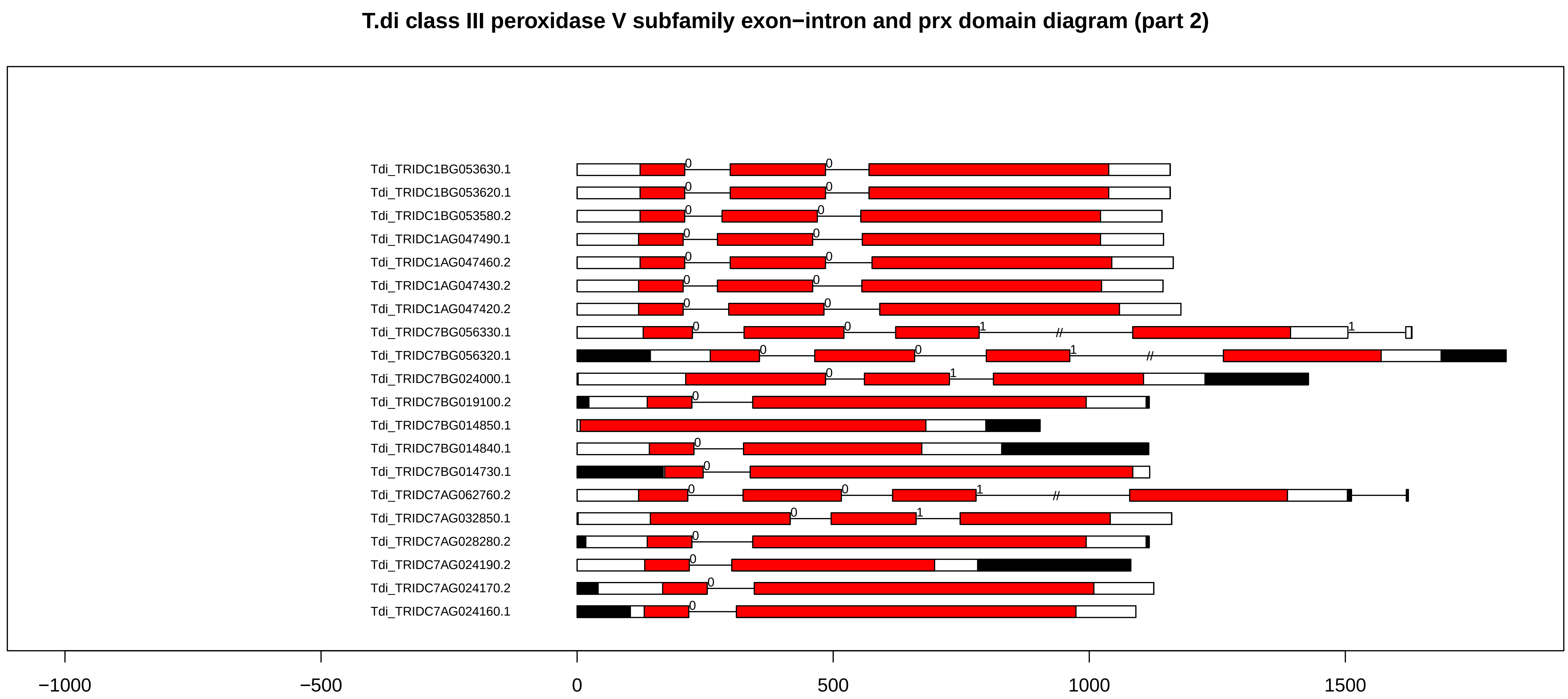
T.di class III peroxidase V subfamily exon−intron and prx domain diagram (part 2)
Tdi_TRIDC1BG053630.1
Tdi_TRIDC1BG053620.1
Tdi_TRIDC1BG053580.2
Tdi_TRIDC1AG047490.1
Tdi_TRIDC1AG047460.2
Tdi_TRIDC1AG047430.2
Tdi_TRIDC1AG047420.2
Tdi_TRIDC7BG056330.1
Tdi_TRIDC7BG056320.1
Tdi_TRIDC7BG024000.1
Tdi_TRIDC7BG019100.2
Tdi_TRIDC7BG014850.1
Tdi_TRIDC7BG014840.1
Tdi_TRIDC7BG014730.1
Tdi_TRIDC7AG062760.2
Tdi_TRIDC7AG032850.1
Tdi_TRIDC7AG028280.2
Tdi_TRIDC7AG024190.2
Tdi_TRIDC7AG024170.2
Tdi_TRIDC7AG024160.1
−1000
−500
0
500
1000
1500

T.di class III peroxidase V subfamily exon−intron and prx domain diagram (part 3)

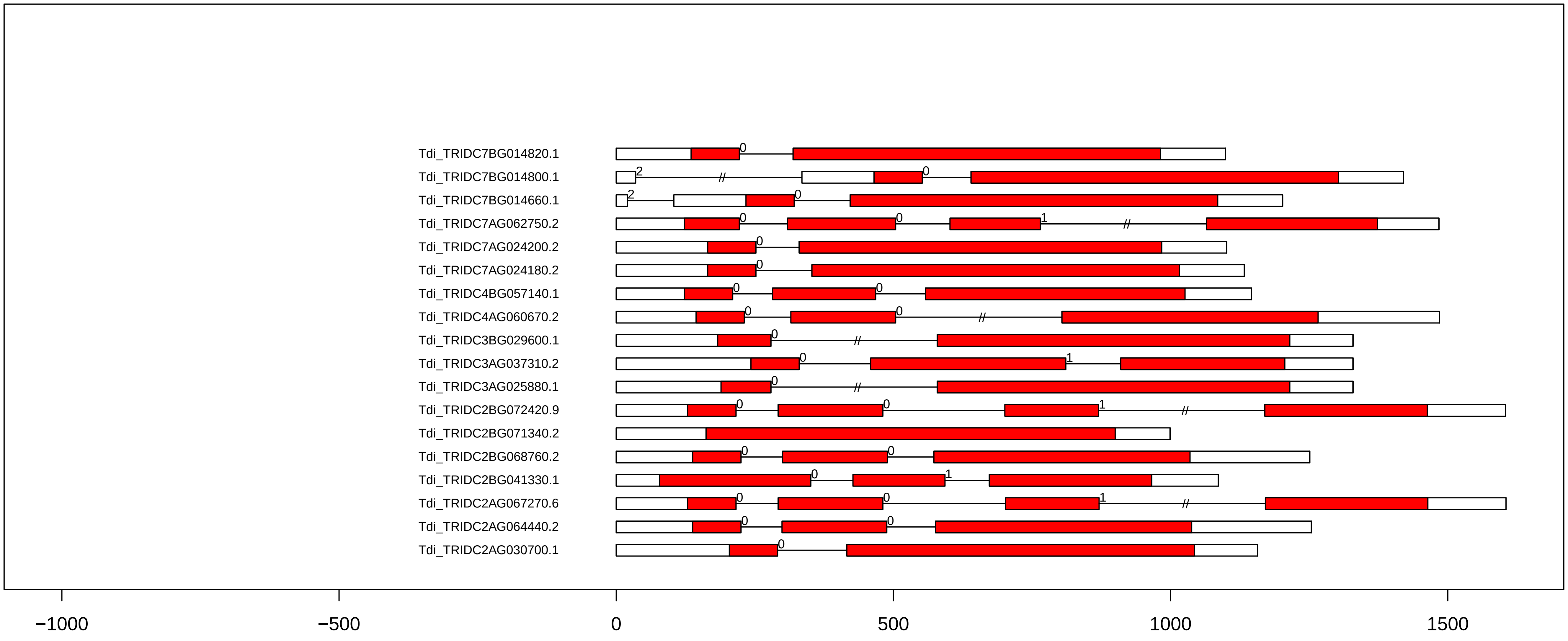

T.di class III peroxidase VI subfamily exon−intron and prx domain diagram (part 1)

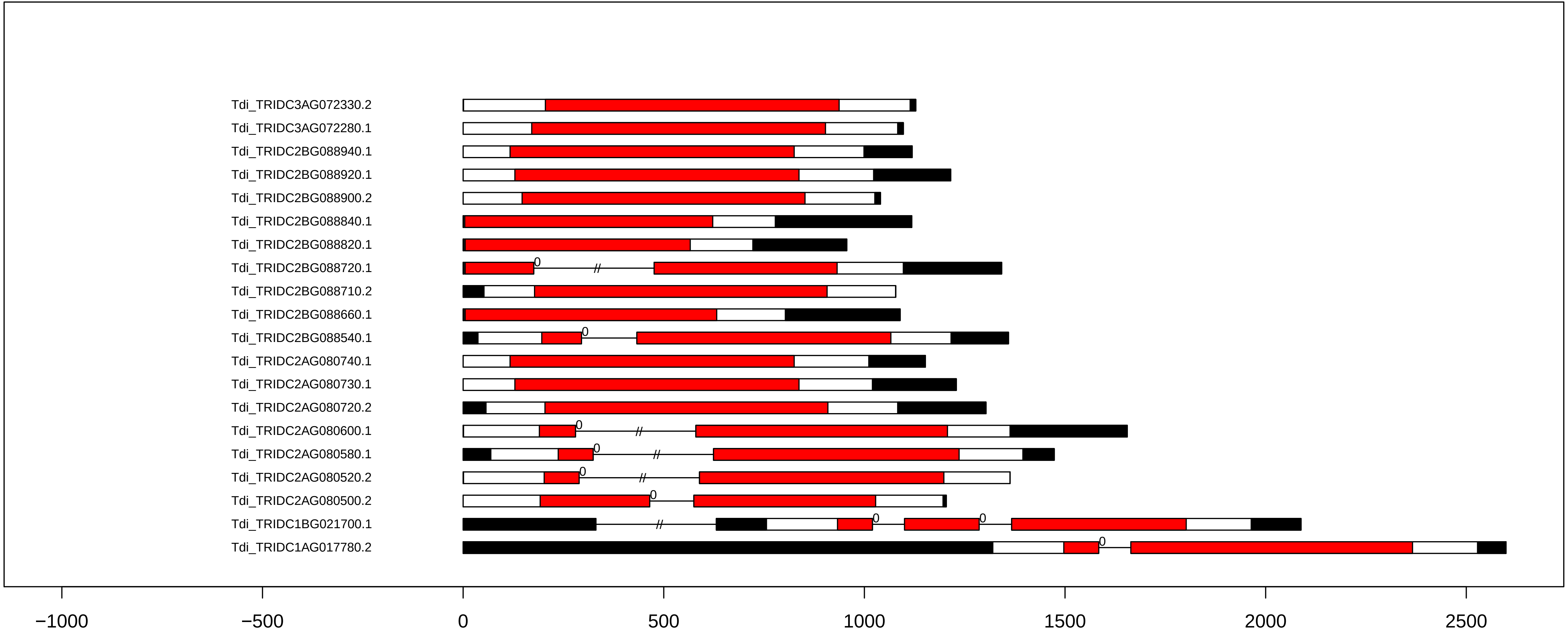

T.di class III peroxidase VI subfamily exon−intron and prx domain diagram (part 2)

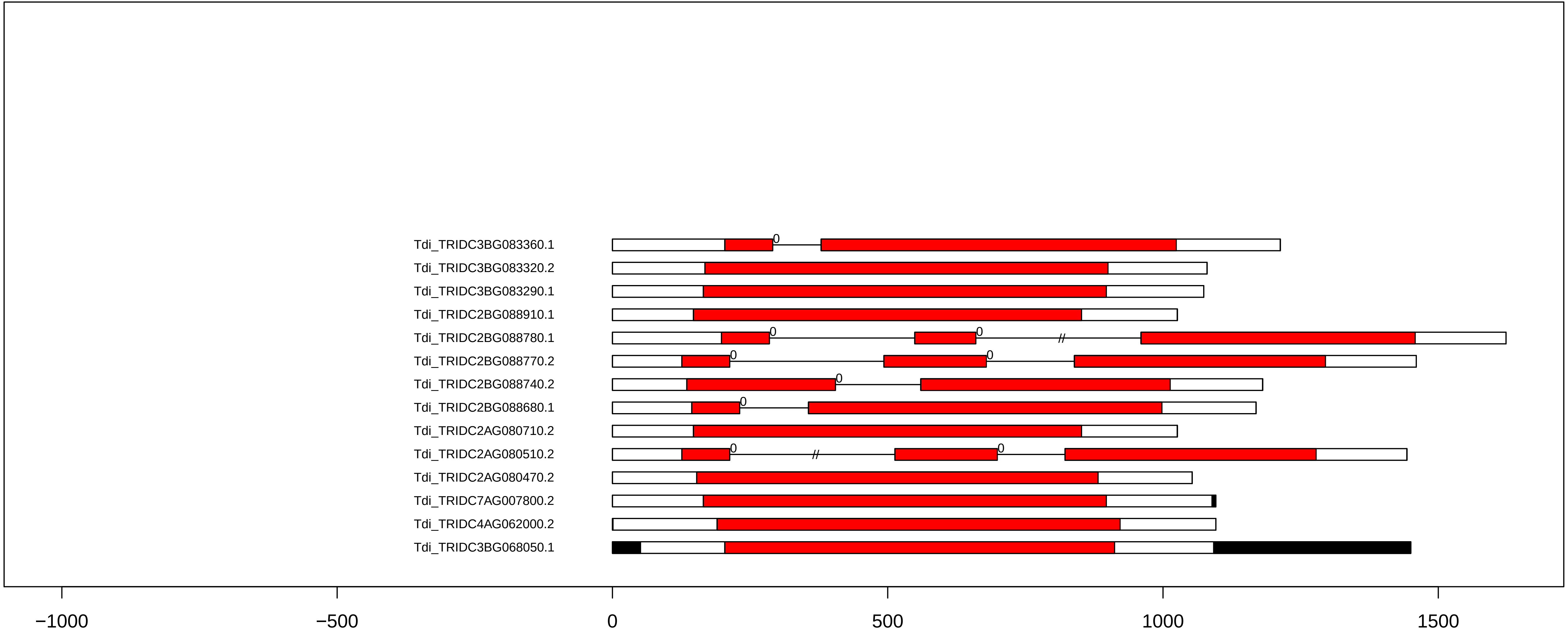

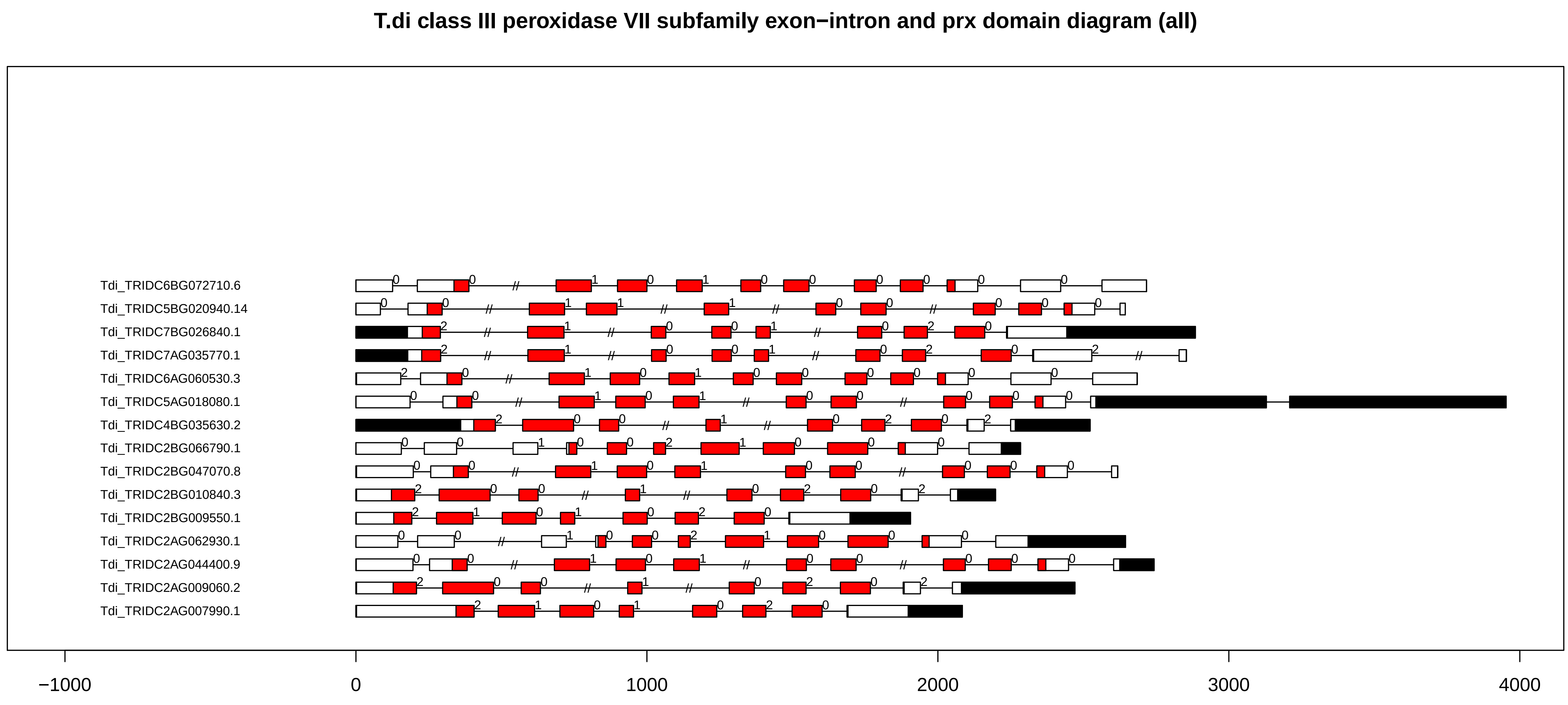
T.di class III peroxidase VII subfamily exon−intron and prx domain diagram (all)
Tdi_TRIDC6BG072710.6
Tdi_TRIDC5BG020940.14
Tdi_TRIDC7BG026840.1
Tdi_TRIDC7AG035770.1
Tdi_TRIDC6AG060530.3
Tdi_TRIDC5AG018080.1
Tdi_TRIDC4BG035630.2
Tdi_TRIDC2BG066790.1
Tdi_TRIDC2BG047070.8
Tdi_TRIDC2BG010840.3
Tdi_TRIDC2BG009550.1
Tdi_TRIDC2AG062930.1
Tdi_TRIDC2AG044400.9
Tdi_TRIDC2AG009060.2
Tdi_TRIDC2AG007990.1
−1000
0
1000
2000
3000
4000

T.di class III peroxidase IX subfamily exon−intron and prx domain diagram (part 1)

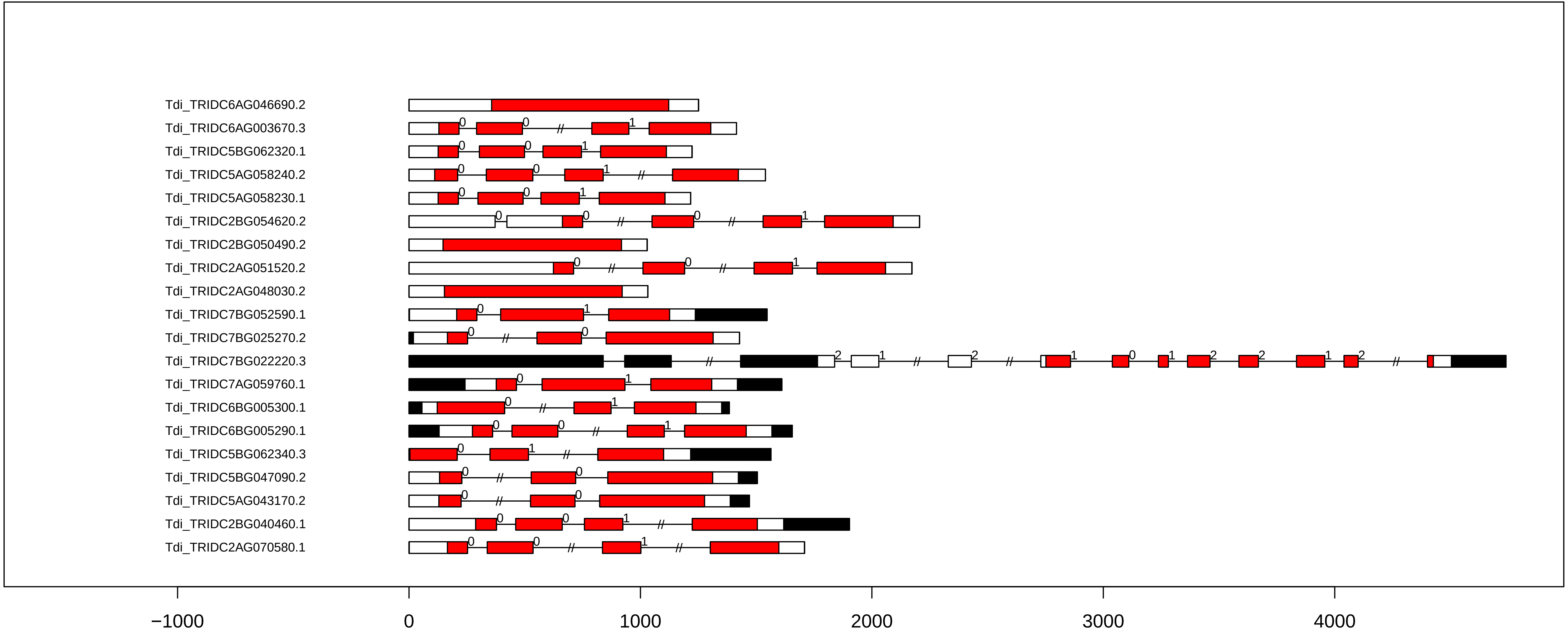

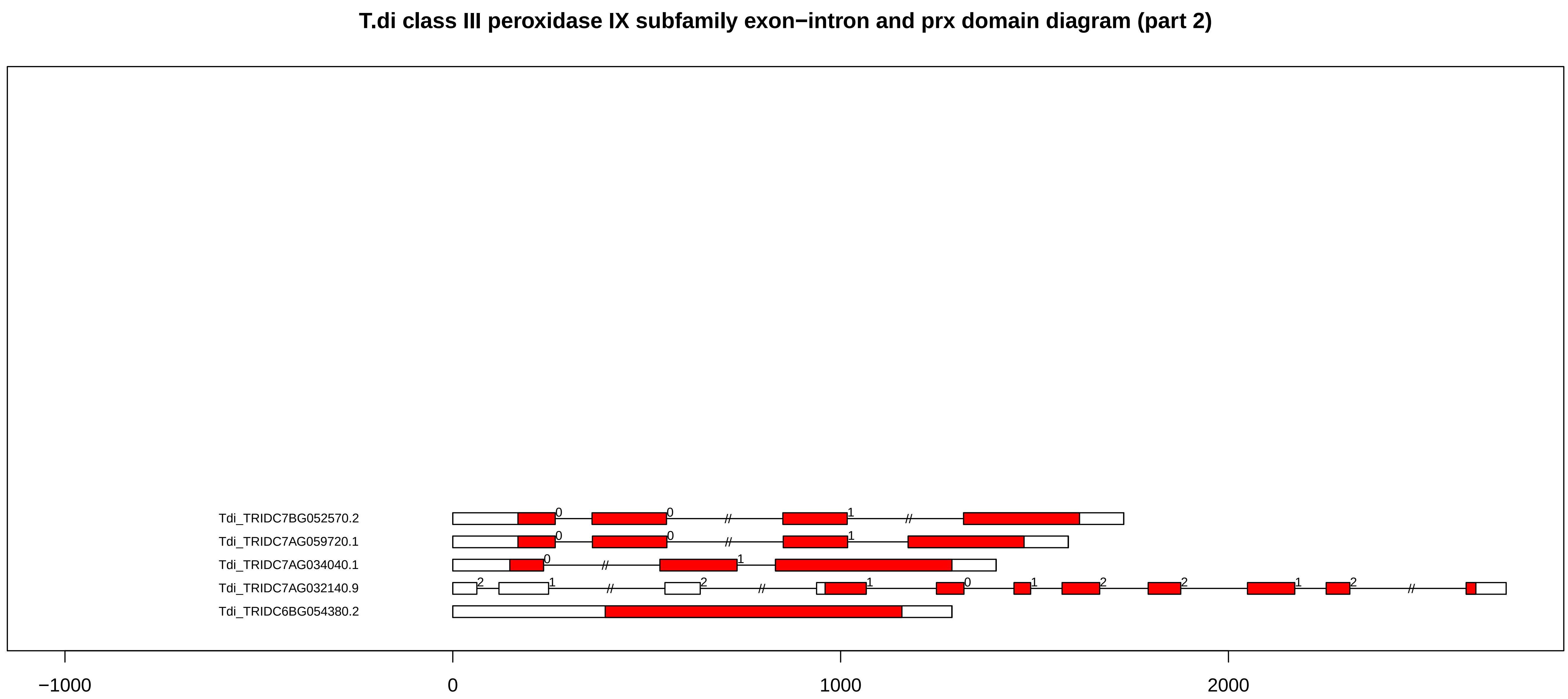

T.di class III peroxidase IX subfamily exon−intron and prx domain diagram (part 2)
Tdi_TRIDC7BG052570.2
Tdi_TRIDC7AG059720.1
Tdi_TRIDC7AG034040.1
Tdi_TRIDC7AG032140.9
Tdi_TRIDC6BG054380.2
−1000
0
1000
2000

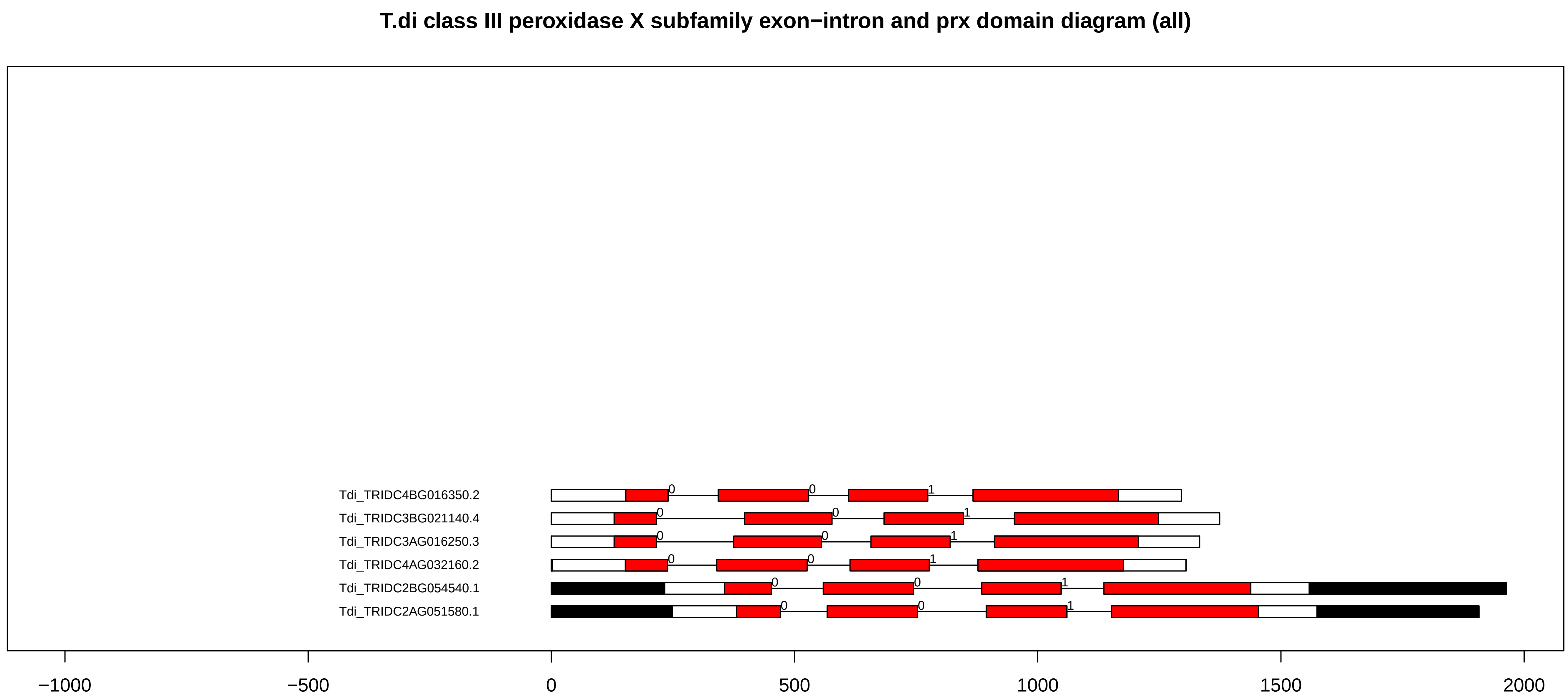

T.di class III peroxidase X subfamily exon−intron and prx domain diagram (all)
Tdi_TRIDC4BG016350.2
Tdi_TRIDC3BG021140.4
Tdi_TRIDC3AG016250.3
Tdi_TRIDC4AG032160.2
Tdi_TRIDC2BG054540.1
Tdi_TRIDC2AG051580.1
−1000
−500
0
500
1000
1500
2000

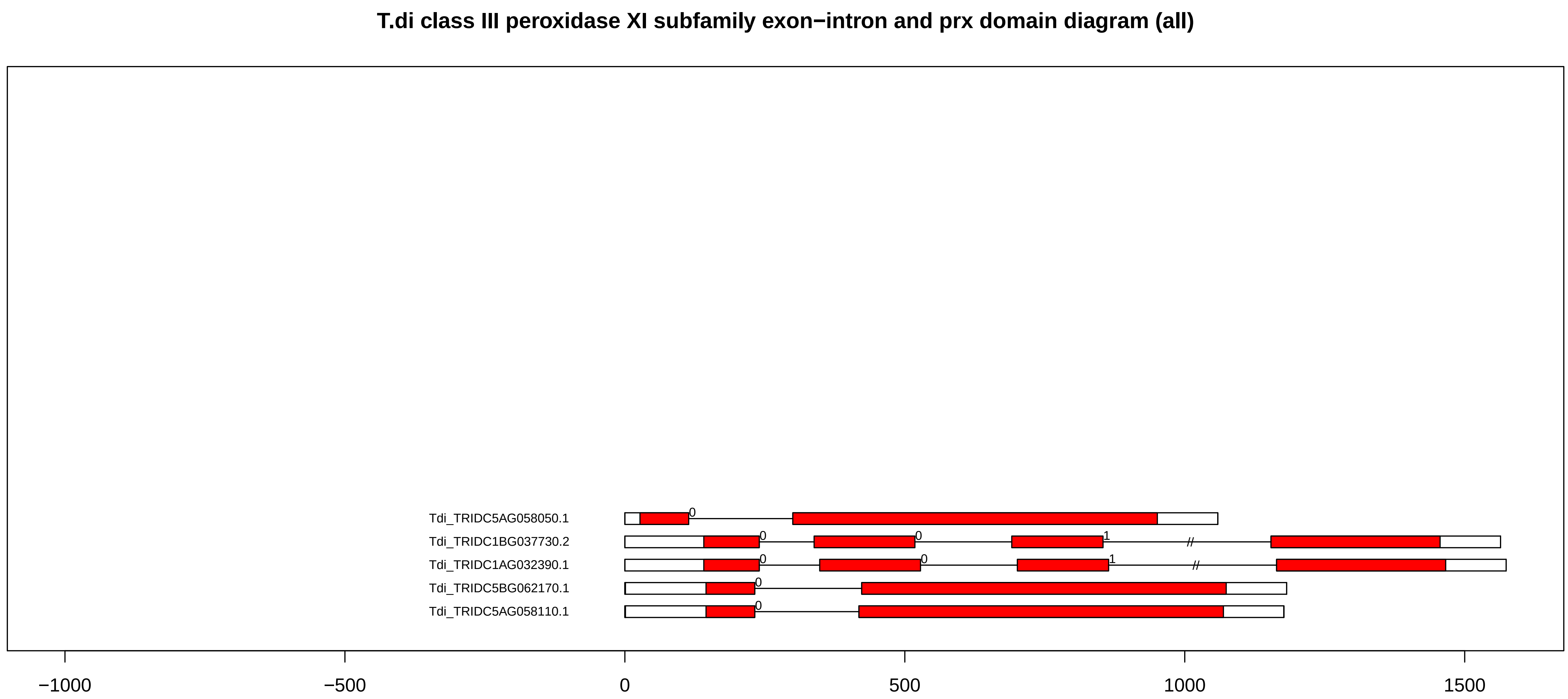

T.di class III peroxidase XI subfamily exon−intron and prx domain diagram (all)
Tdi_TRIDC5AG058050.1
Tdi_TRIDC1BG037730.2
Tdi_TRIDC1AG032390.1
Tdi_TRIDC5BG062170.1
Tdi_TRIDC5AG058110.1
0
0
0
1
0
0
1
0
0
//
//
−1000
−500
0
500
1000
1500

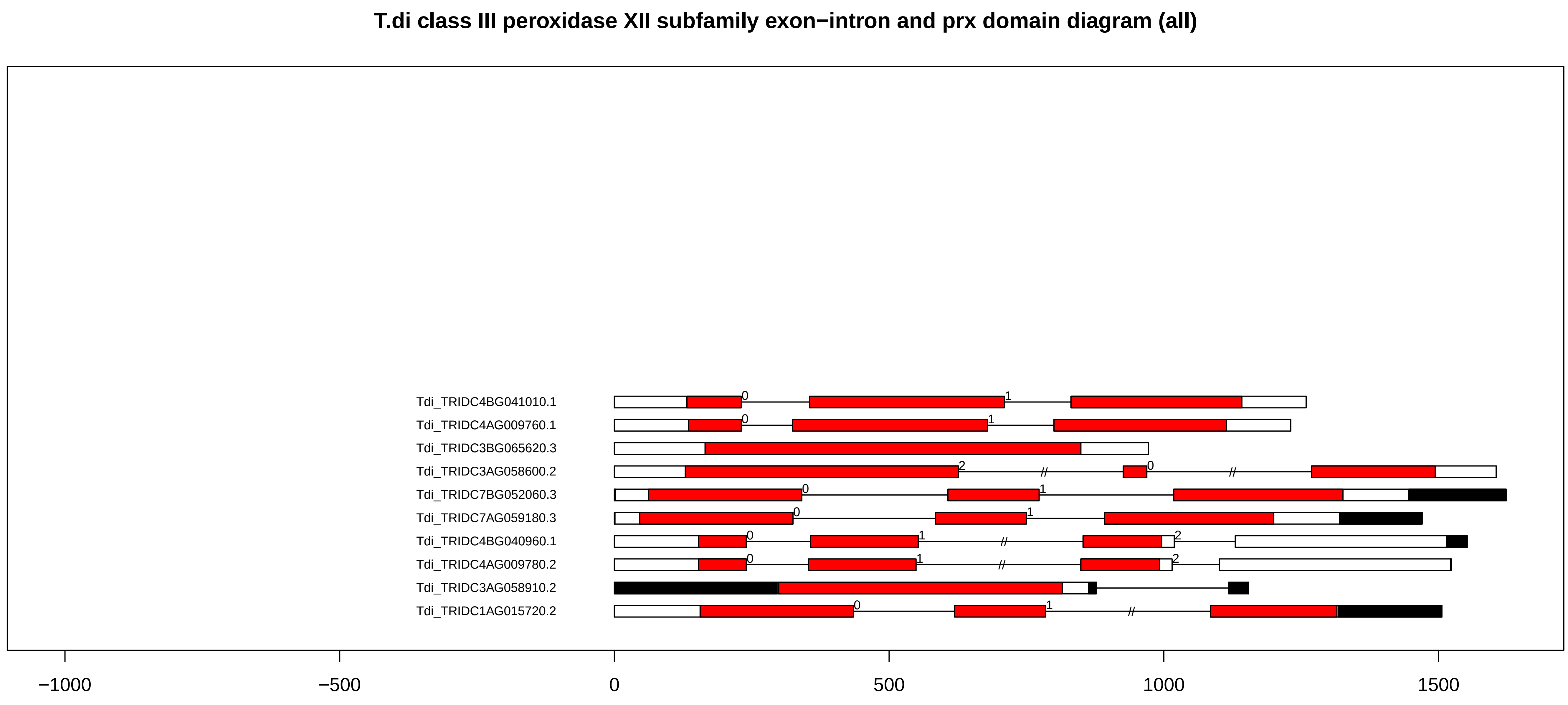
T.di class III peroxidase XII subfamily exon−intron and prx domain diagram (all)
Tdi_TRIDC4BG041010.1
Tdi_TRIDC4AG009760.1
Tdi_TRIDC3BG065620.3
Tdi_TRIDC3AG058600.2
Tdi_TRIDC7BG052060.3
Tdi_TRIDC7AG059180.3
Tdi_TRIDC4BG040960.1
Tdi_TRIDC4AG009780.2
Tdi_TRIDC3AG058910.2
Tdi_TRIDC1AG015720.2
−1000
−500
0
500
1000
1500

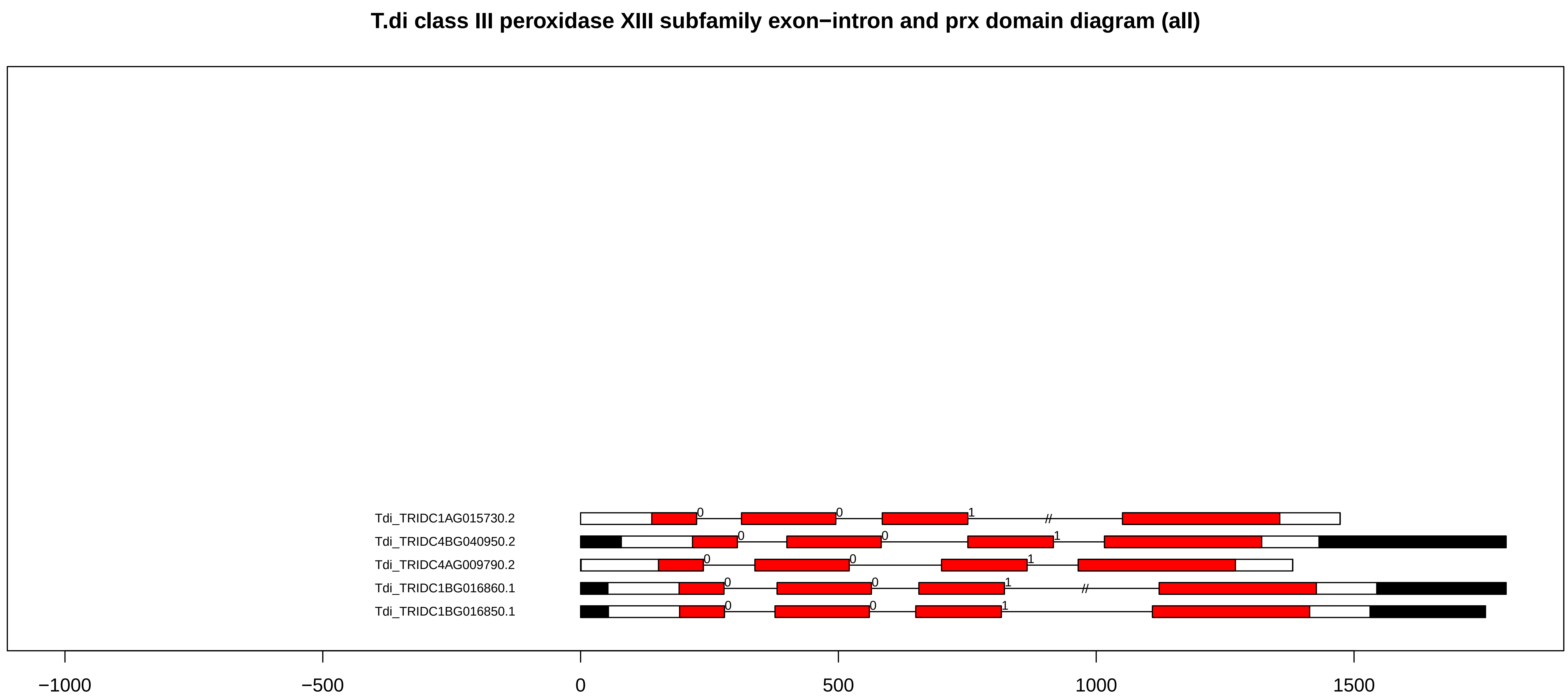
T.di class III peroxidase XIII subfamily exon−intron and prx domain diagram (all)
Tdi_TRIDC1AG015730.2
Tdi_TRIDC4BG040950.2
Tdi_TRIDC4AG009790.2
Tdi_TRIDC1BG016860.1
Tdi_TRIDC1BG016850.1
−1000
−500
0
500
1000
1500

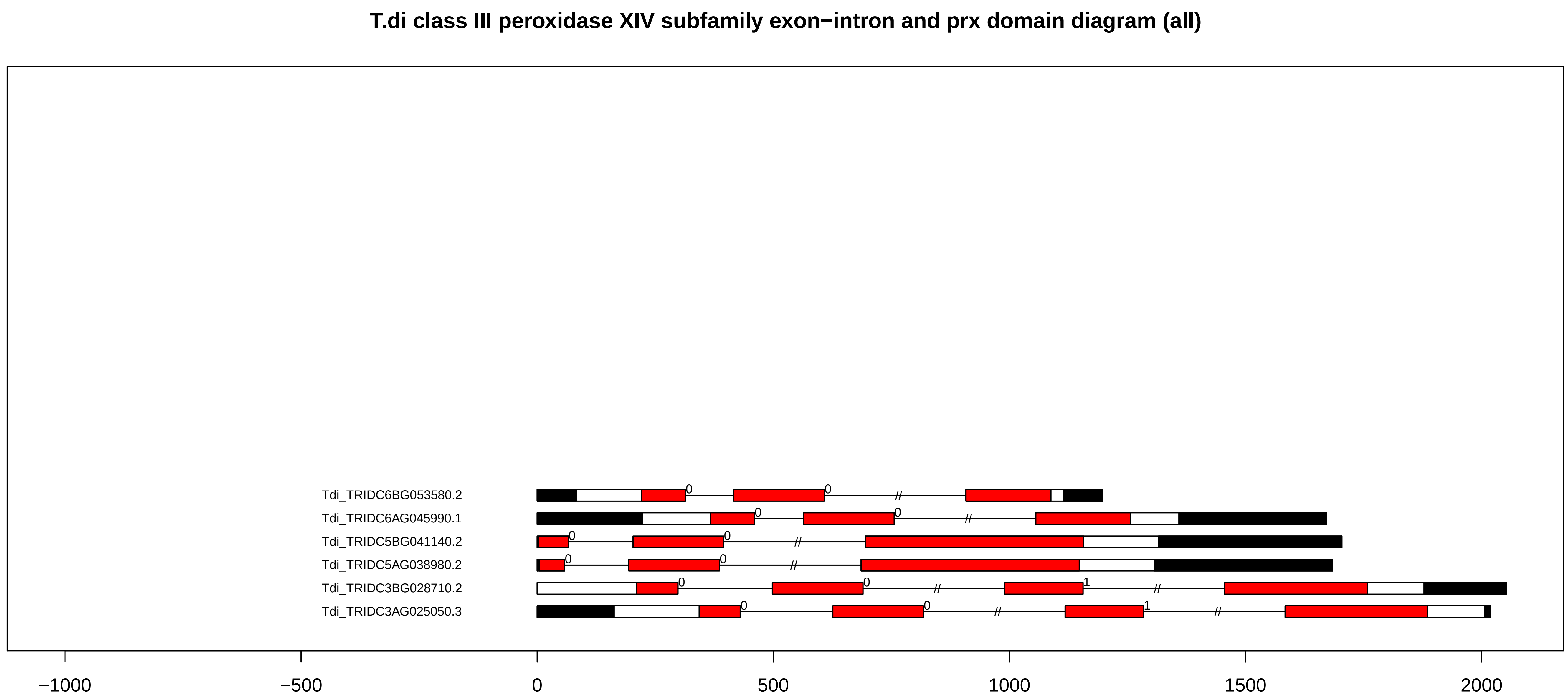

T.di class III peroxidase XIV subfamily exon−intron and prx domain diagram (all)
Tdi_TRIDC6BG053580.2
Tdi_TRIDC6AG045990.1
Tdi_TRIDC5BG041140.2
Tdi_TRIDC5AG038980.2
Tdi_TRIDC3BG028710.2
Tdi_TRIDC3AG025050.3
−1000
−500
0
500
1000
1500
2000

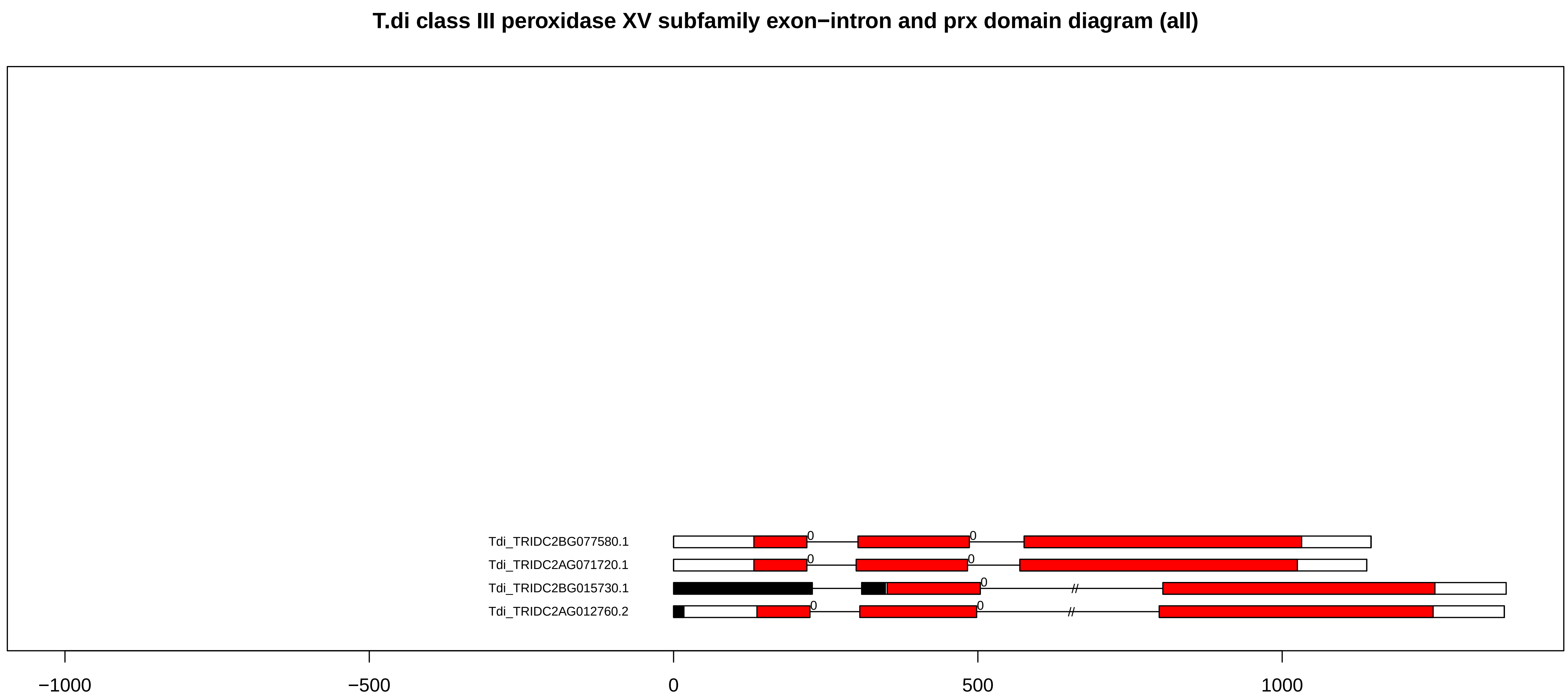

T.di class III peroxidase XV subfamily exon−intron and prx domain diagram (all)
Tdi_TRIDC2BG077580.1
Tdi_TRIDC2AG071720.1
Tdi_TRIDC2BG015730.1
Tdi_TRIDC2AG012760.2
−1000
−500
0
500
1000

T.di class III peroxidase XVI subfamily exon−intron and prx domain diagram (part 1)

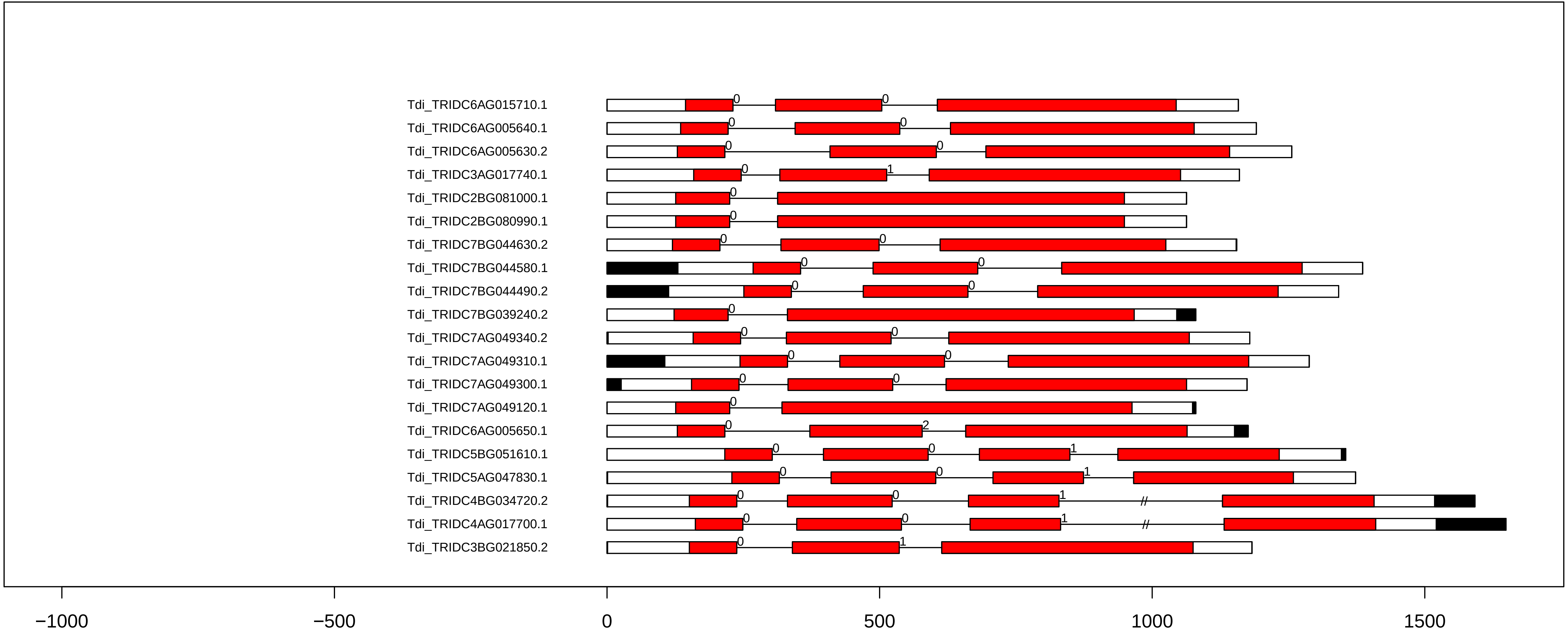

T.di class III peroxidase XVI subfamily exon−intron and prx domain diagram (part 2)

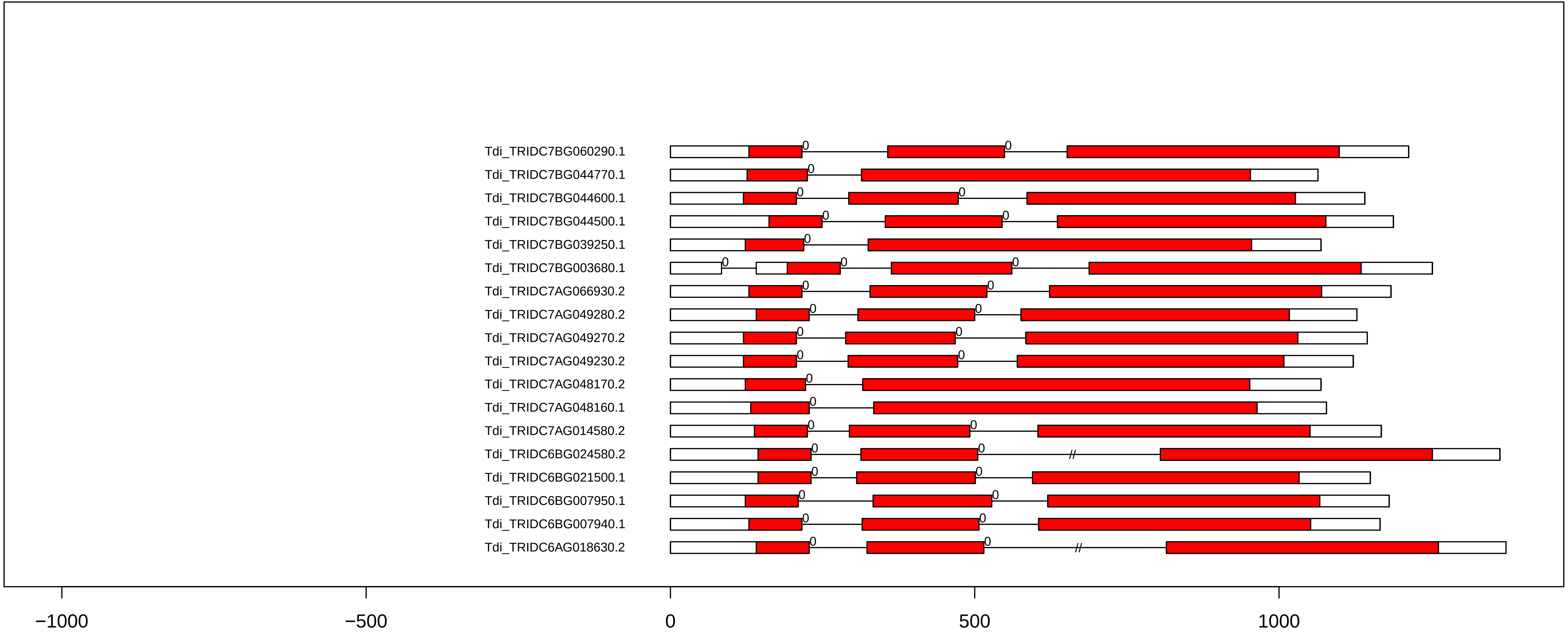

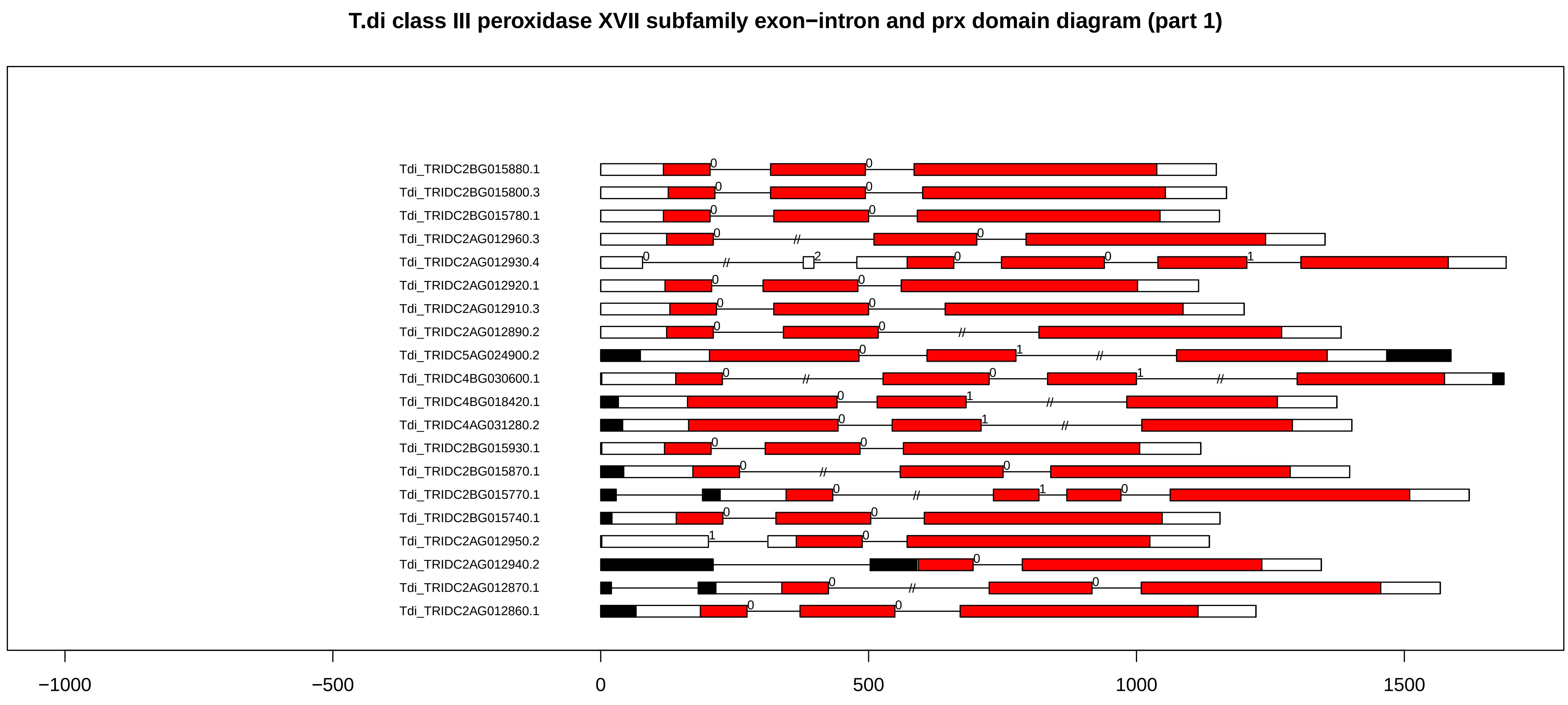
T.di class III peroxidase XVII subfamily exon−intron and prx domain diagram (part 1)
Tdi_TRIDC2BG015880.1
Tdi_TRIDC2BG015800.3
Tdi_TRIDC2BG015780.1
Tdi_TRIDC2AG012960.3
Tdi_TRIDC2AG012930.4
Tdi_TRIDC2AG012920.1
Tdi_TRIDC2AG012910.3
Tdi_TRIDC2AG012890.2
Tdi_TRIDC5AG024900.2
Tdi_TRIDC4BG030600.1
Tdi_TRIDC4BG018420.1
Tdi_TRIDC4AG031280.2
Tdi_TRIDC2BG015930.1
Tdi_TRIDC2BG015870.1
Tdi_TRIDC2BG015770.1
Tdi_TRIDC2BG015740.1
Tdi_TRIDC2AG012950.2
Tdi_TRIDC2AG012940.2
Tdi_TRIDC2AG012870.1
Tdi_TRIDC2AG012860.1
−1000
−500
0
500
1000
1500

T.di class III peroxidase XVII subfamily exon−intron and prx domain diagram (part 2)

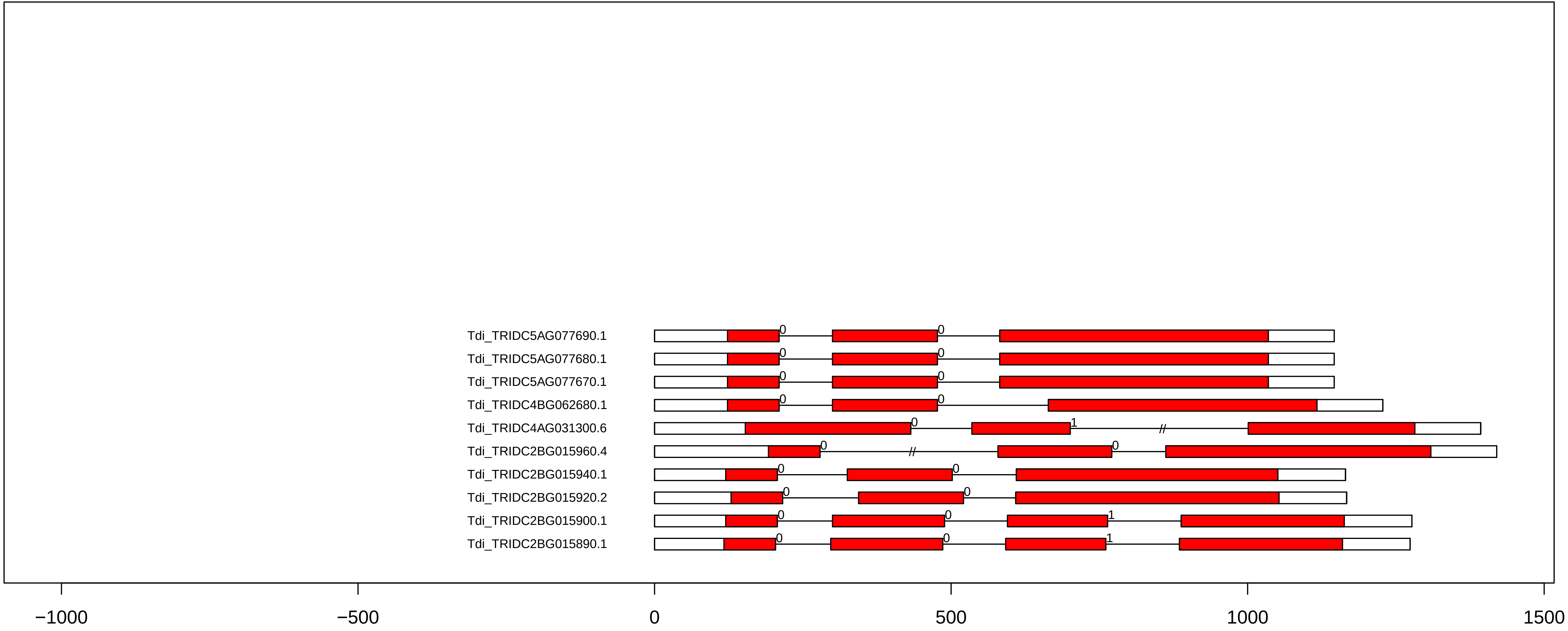

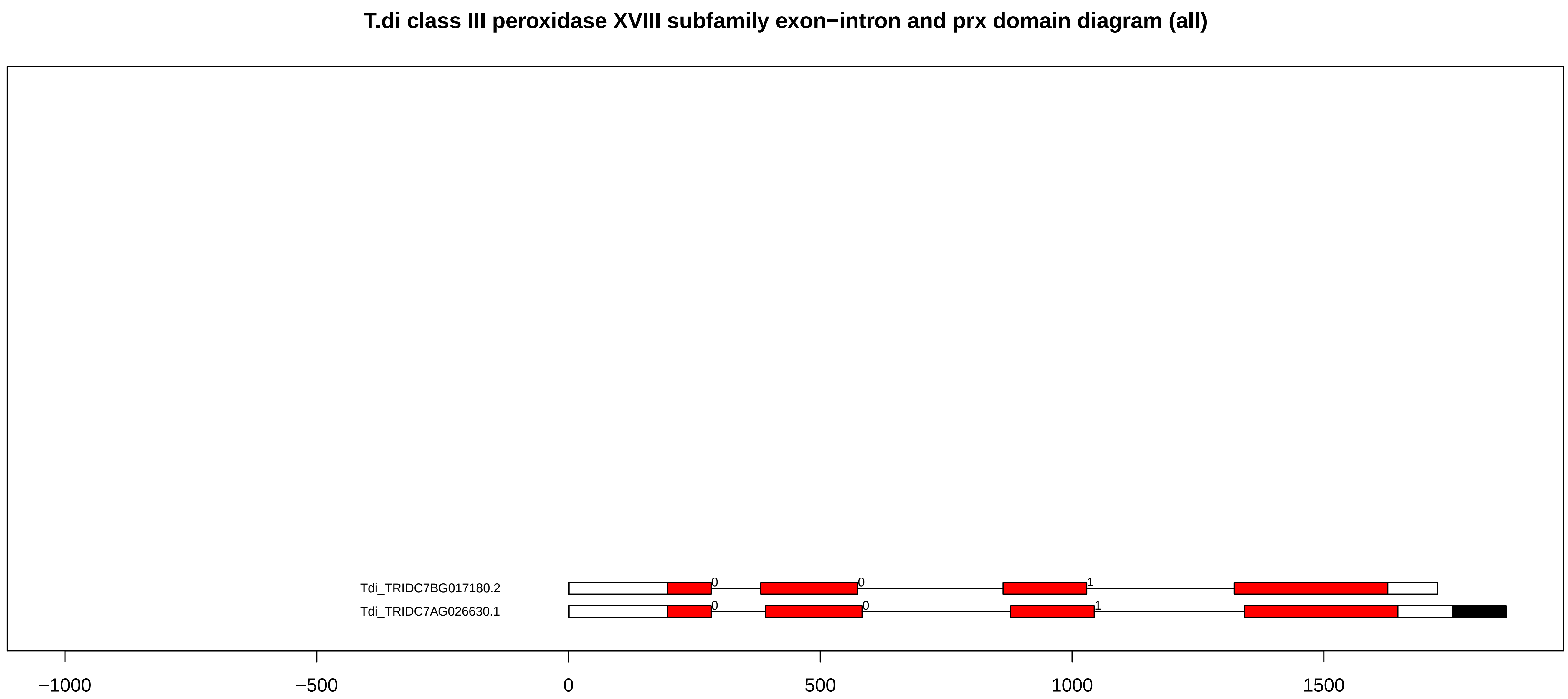

T.di class III peroxidase XVIII subfamily exon−intron and prx domain diagram (all)
Tdi_TRIDC7BG017180.2
Tdi_TRIDC7AG026630.1
0
0
1
0
0
1
−1000
−500
0
500
1000
1500

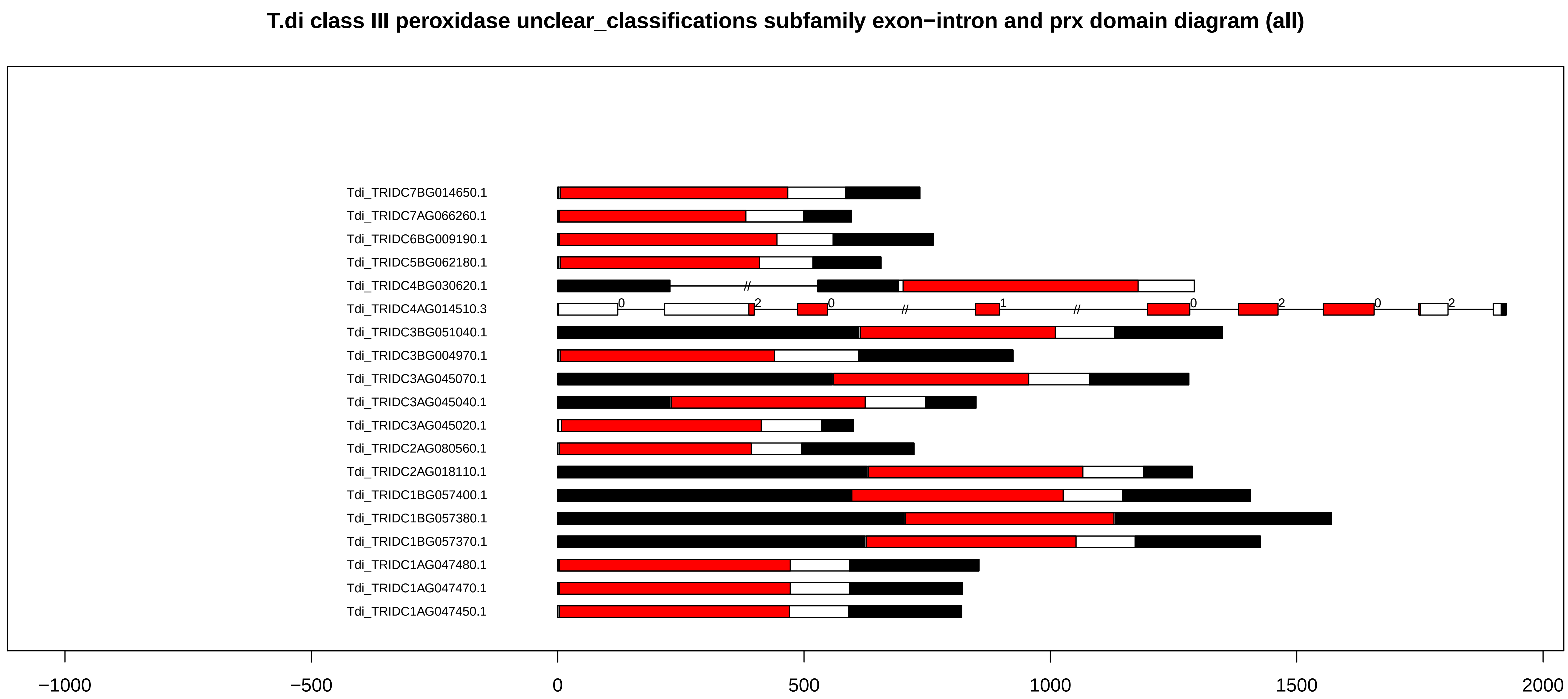

T.di class III peroxidase unclear_classifications subfamily exon−intron and prx domain diagram (all)
Tdi_TRIDC7BG014650.1
Tdi_TRIDC7AG066260.1
Tdi_TRIDC6BG009190.1
Tdi_TRIDC5BG062180.1
Tdi_TRIDC4BG030620.1
Tdi_TRIDC4AG014510.3
Tdi_TRIDC3BG051040.1
Tdi_TRIDC3BG004970.1
Tdi_TRIDC3AG045070.1
Tdi_TRIDC3AG045040.1
Tdi_TRIDC3AG045020.1
Tdi_TRIDC2AG080560.1
Tdi_TRIDC2AG018110.1
Tdi_TRIDC1BG057400.1
Tdi_TRIDC1BG057380.1
Tdi_TRIDC1BG057370.1
Tdi_TRIDC1AG047480.1
Tdi_TRIDC1AG047470.1
Tdi_TRIDC1AG047450.1
−1000
−500
0
500
1000
1500
2000

T.ur class III peroxidase I subfamily exon−intron and prx domain diagram (part 1)

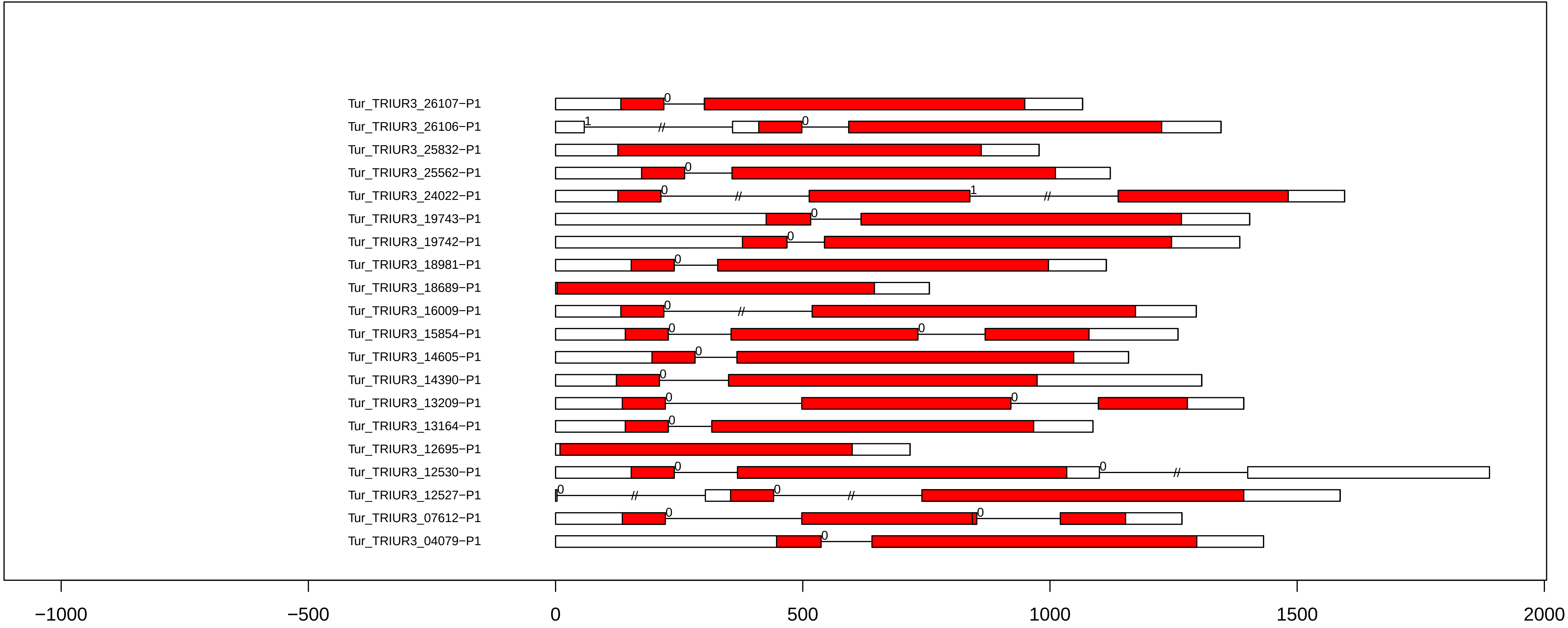

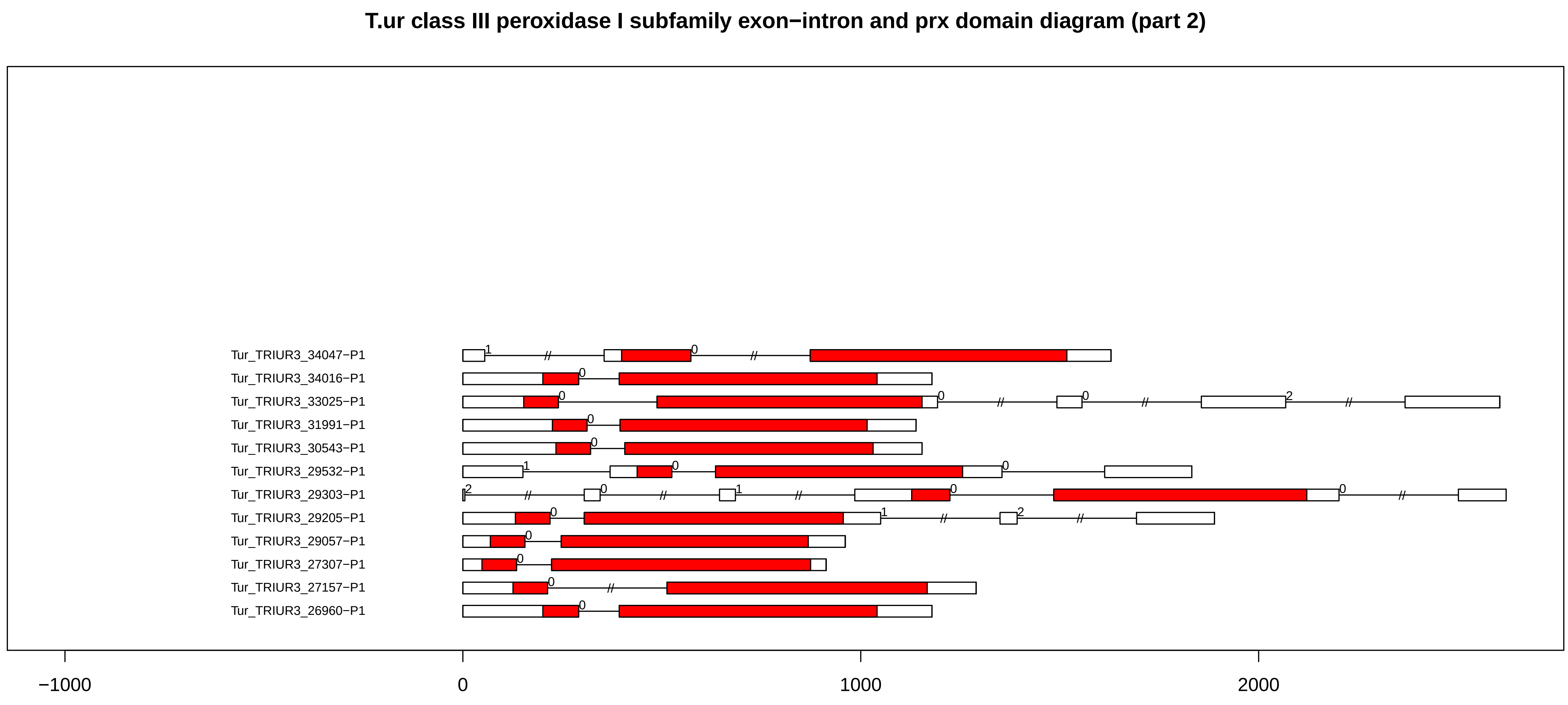

T.ur class III peroxidase I subfamily exon−intron and prx domain diagram (part 2)
Tur_TRIUR3_34047−P1
Tur_TRIUR3_34016−P1
Tur_TRIUR3_33025−P1
Tur_TRIUR3_31991−P1
Tur_TRIUR3_30543−P1
Tur_TRIUR3_29532−P1
Tur_TRIUR3_29303−P1
Tur_TRIUR3_29205−P1
Tur_TRIUR3_29057−P1
Tur_TRIUR3_27307−P1
Tur_TRIUR3_27157−P1
Tur_TRIUR3_26960−P1
−1000
0
1000
2000

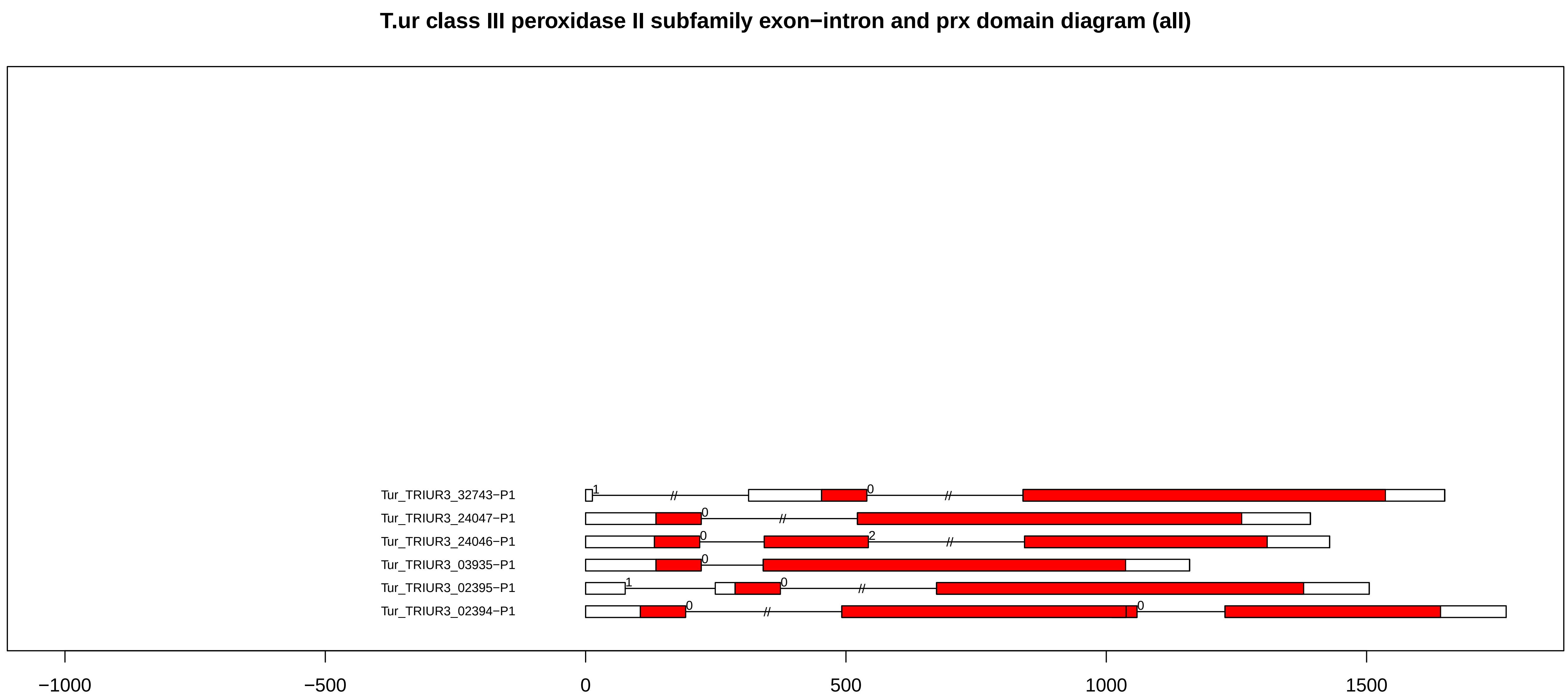
T.ur class III peroxidase II subfamily exon−intron and prx domain diagram (all)
Tur_TRIUR3_32743−P1
Tur_TRIUR3_24047−P1
Tur_TRIUR3_24046−P1
Tur_TRIUR3_03935−P1
Tur_TRIUR3_02395−P1
Tur_TRIUR3_02394−P1
−1000
−500
0
500
1000
1500

T.ur class III peroxidase III subfamily exon−intron and prx domain diagram (all)

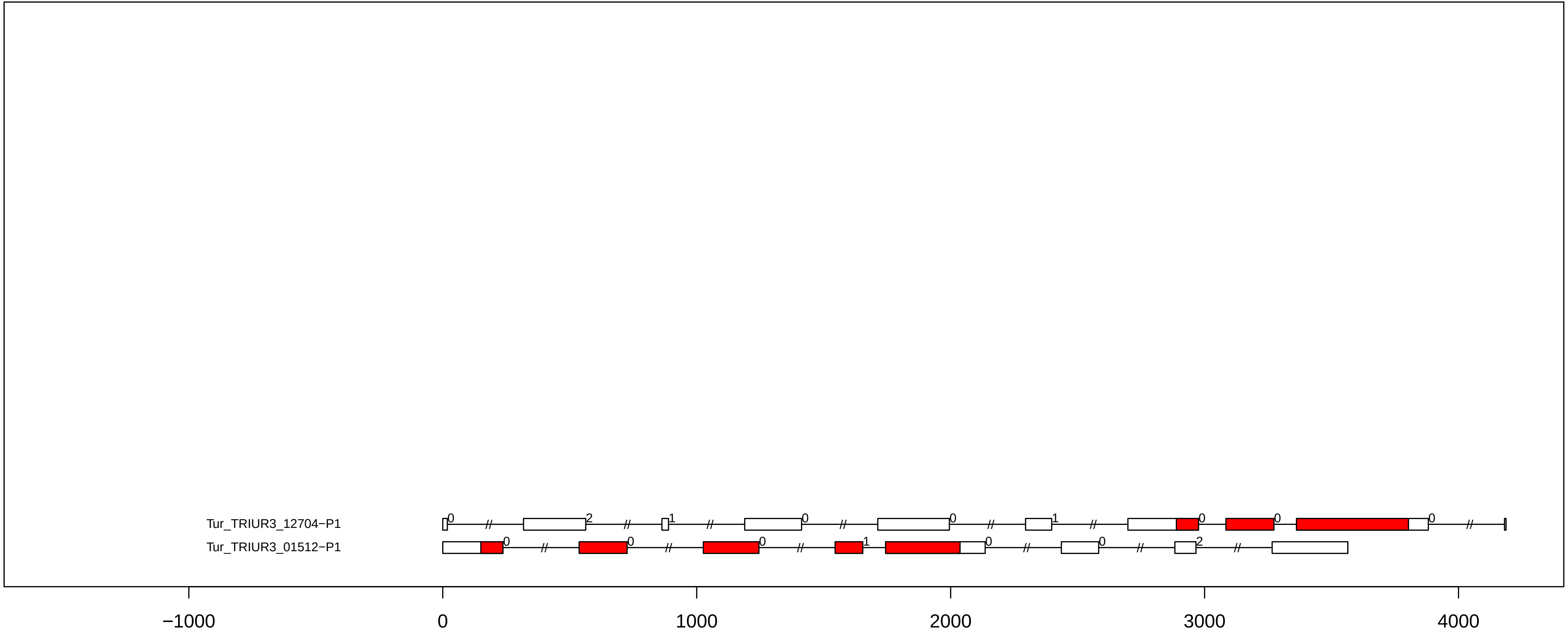

T.ur class III peroxidase V subfamily exon−intron and prx domain diagram (all)

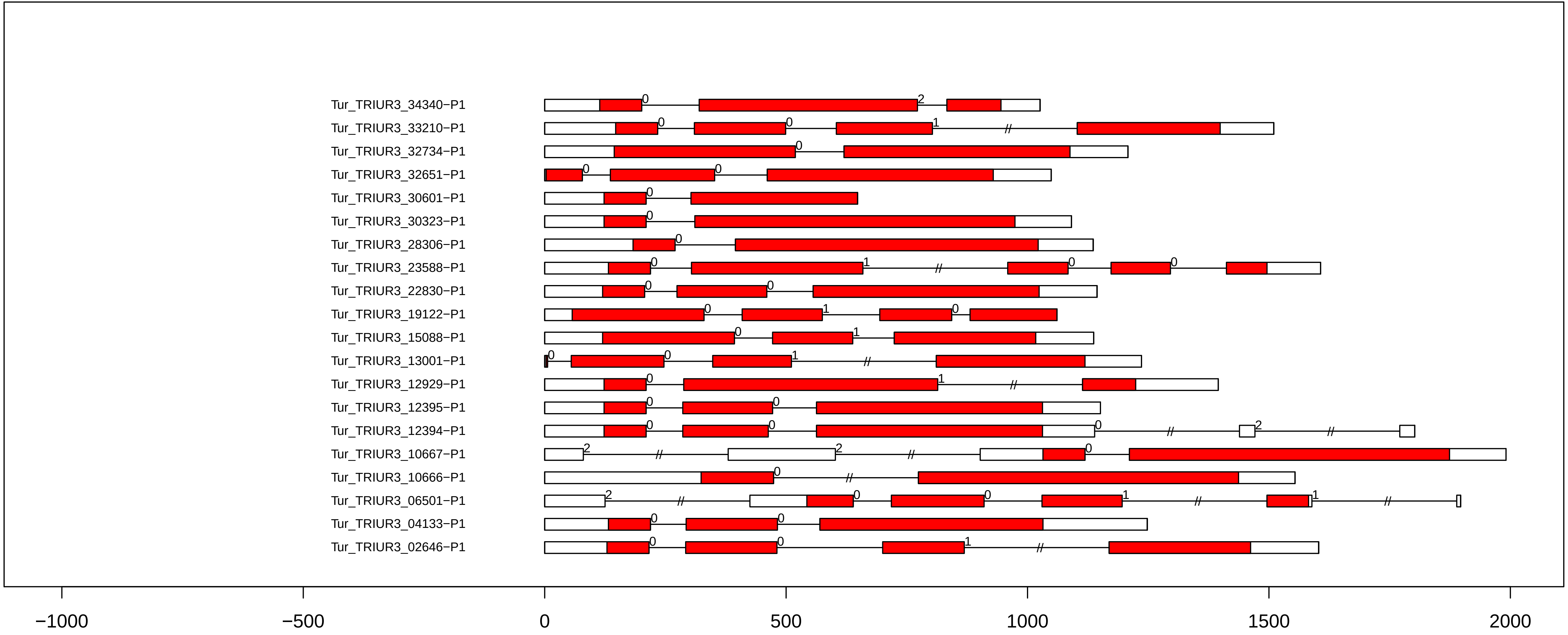

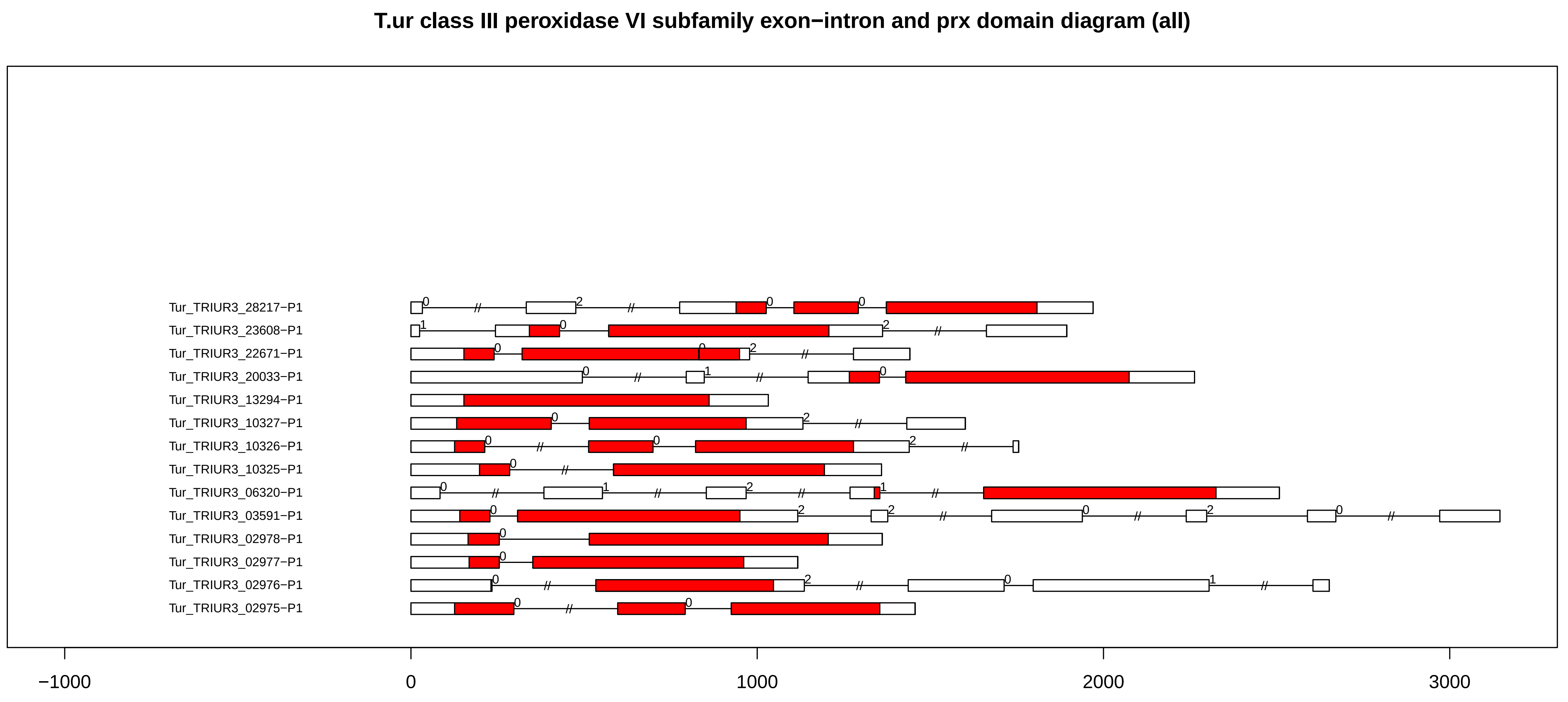

T.ur class III peroxidase VI subfamily exon−intron and prx domain diagram (all)
Tur_TRIUR3_28217−P1
Tur_TRIUR3_23608−P1
Tur_TRIUR3_22671−P1
Tur_TRIUR3_20033−P1
Tur_TRIUR3_13294−P1
Tur_TRIUR3_10327−P1
Tur_TRIUR3_10326−P1
Tur_TRIUR3_10325−P1
Tur_TRIUR3_06320−P1
Tur_TRIUR3_03591−P1
Tur_TRIUR3_02978−P1
Tur_TRIUR3_02977−P1
Tur_TRIUR3_02976−P1
Tur_TRIUR3_02975−P1
−1000
0
1000
2000
3000

T.ur class III peroxidase VII subfamily exon−intron and prx domain diagram (all)

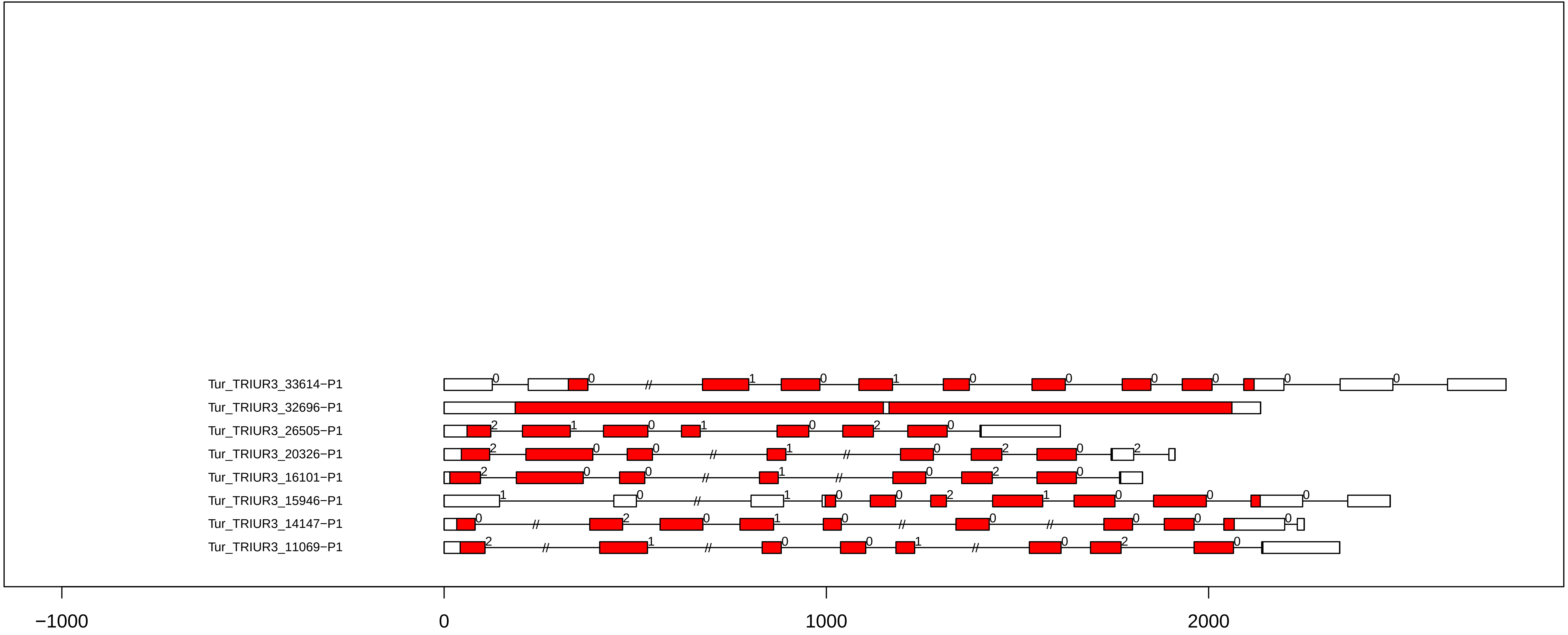

T.ur class III peroxidase IX subfamily exon−intron and prx domain diagram (all)

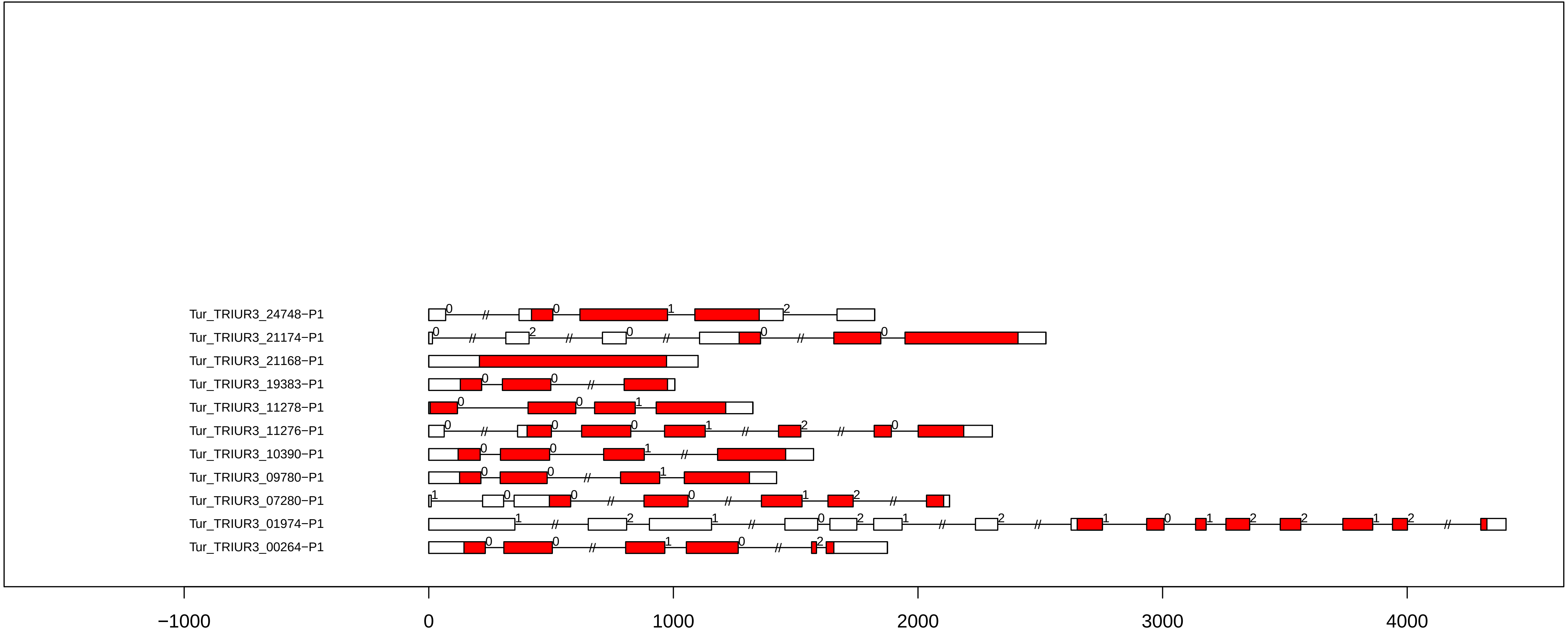

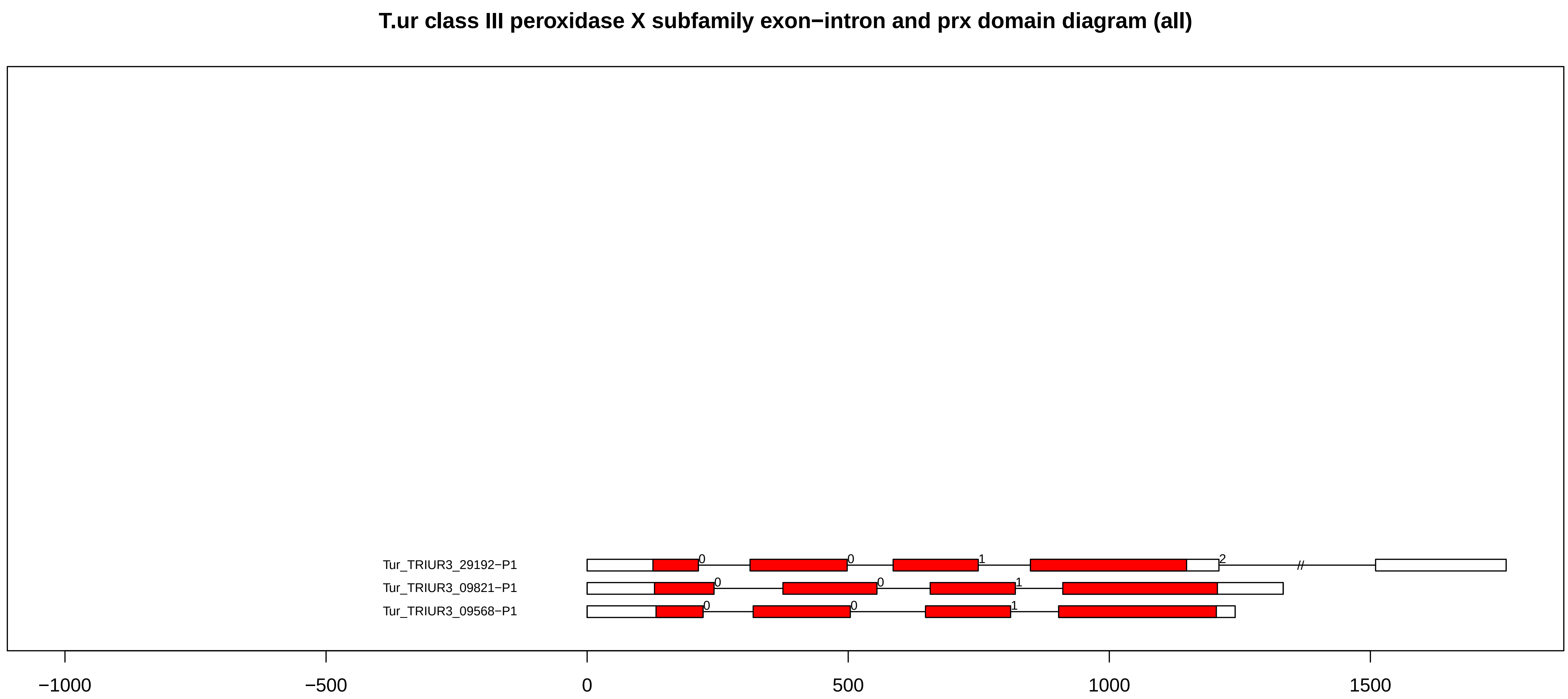
T.ur class III peroxidase X subfamily exon−intron and prx domain diagram (all)
Tur_TRIUR3_29192−P1
Tur_TRIUR3_09821−P1
Tur_TRIUR3_09568−P1
0
0
1
2
0
0
1
0
0
1
−1000
−500
0
500
1000
1500

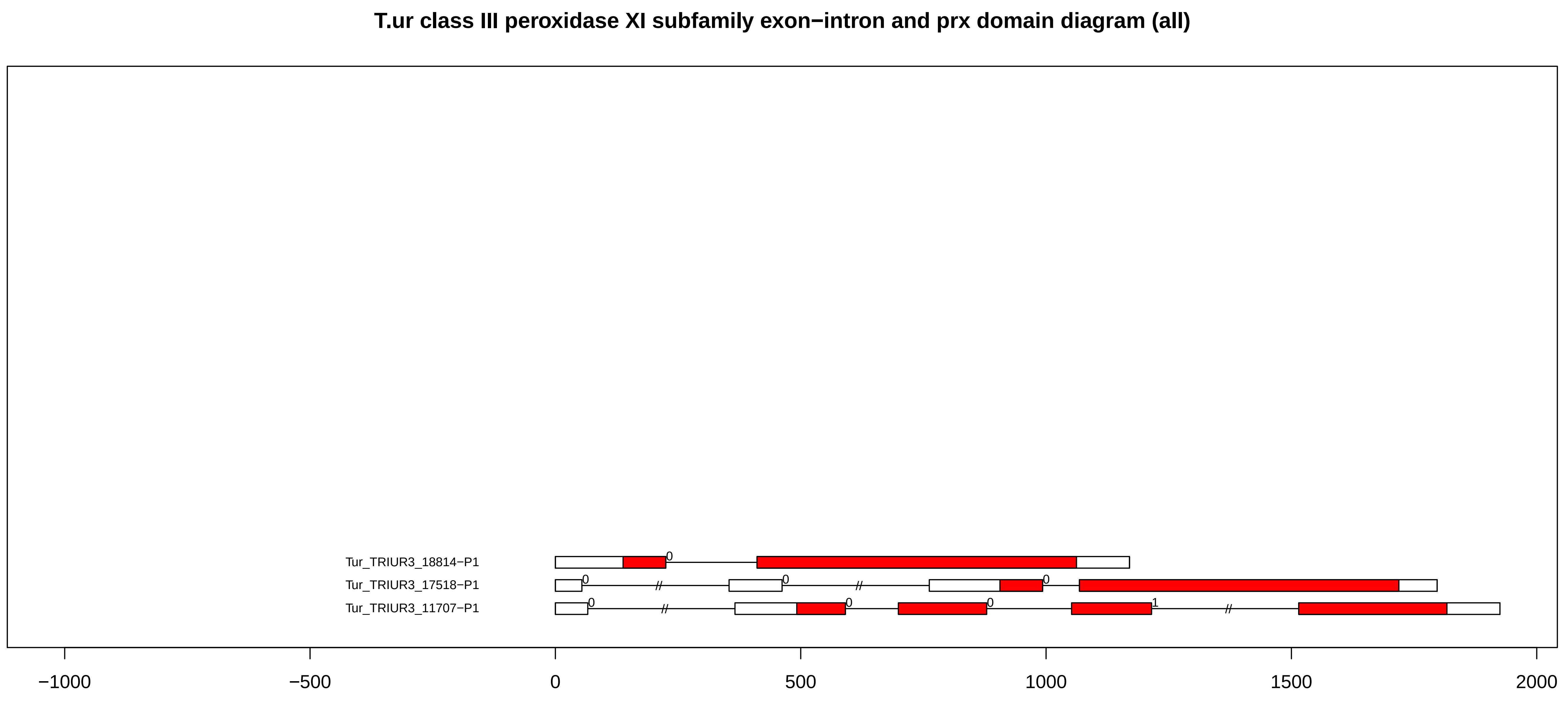
T.ur class III peroxidase XI subfamily exon−intron and prx domain diagram (all)
Tur_TRIUR3_18814−P1
Tur_TRIUR3_17518−P1
Tur_TRIUR3_11707−P1
0
0
0
0
0
0
0
1
−1000
−500
0
500
1000
1500
2000

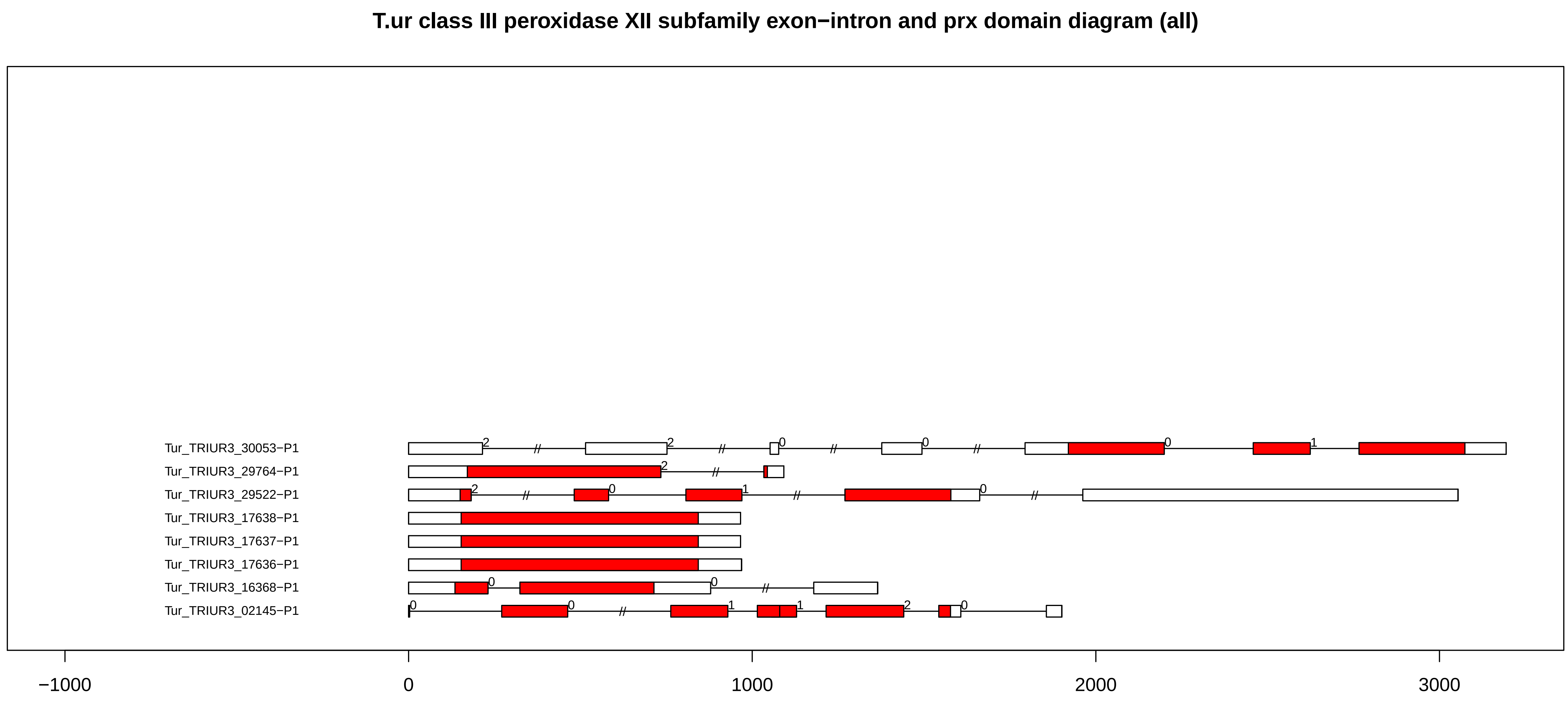

T.ur class III peroxidase XII subfamily exon−intron and prx domain diagram (all)
Tur_TRIUR3_30053−P1
Tur_TRIUR3_29764−P1
Tur_TRIUR3_29522−P1
Tur_TRIUR3_17638−P1
Tur_TRIUR3_17637−P1
Tur_TRIUR3_17636−P1
Tur_TRIUR3_16368−P1
Tur_TRIUR3_02145−P1
−1000
0
1000
2000
3000

T.ur class III peroxidase XIII subfamily exon−intron and prx domain diagram (all)

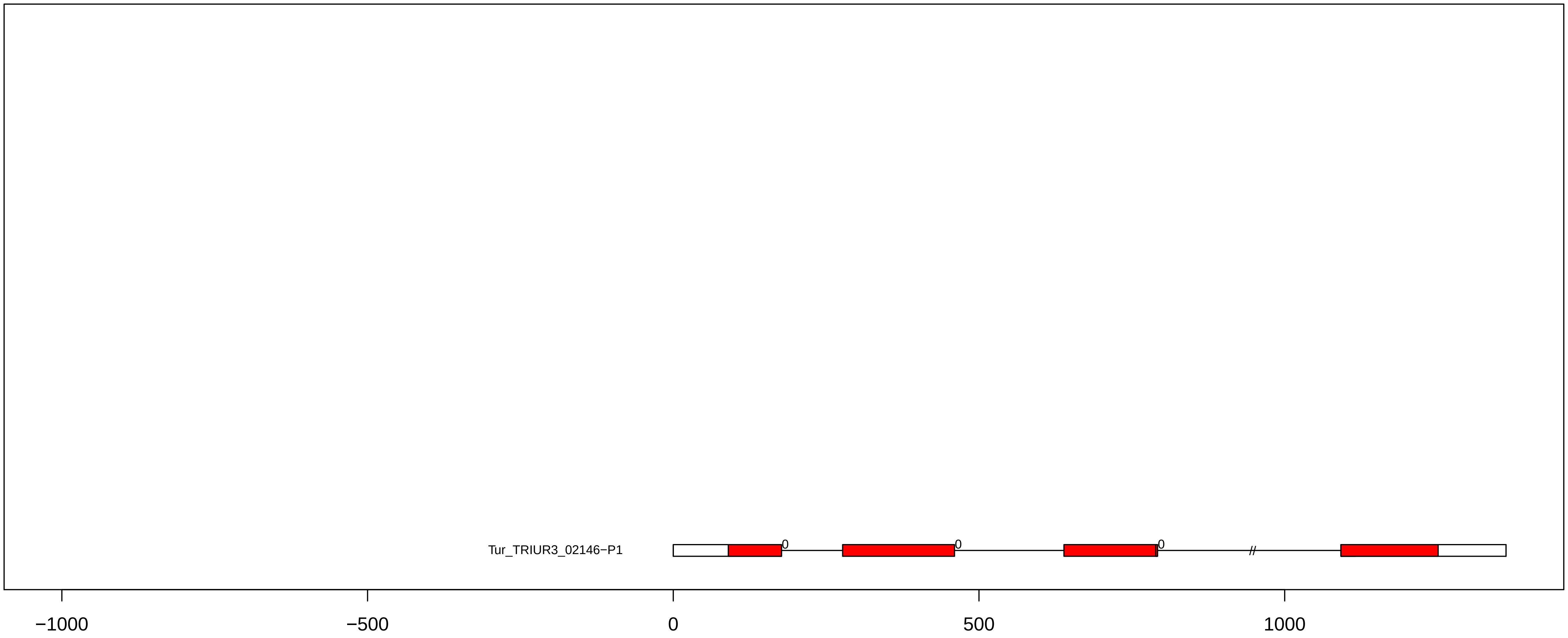

T.ur class III peroxidase XIV subfamily exon−intron and prx domain diagram (all)

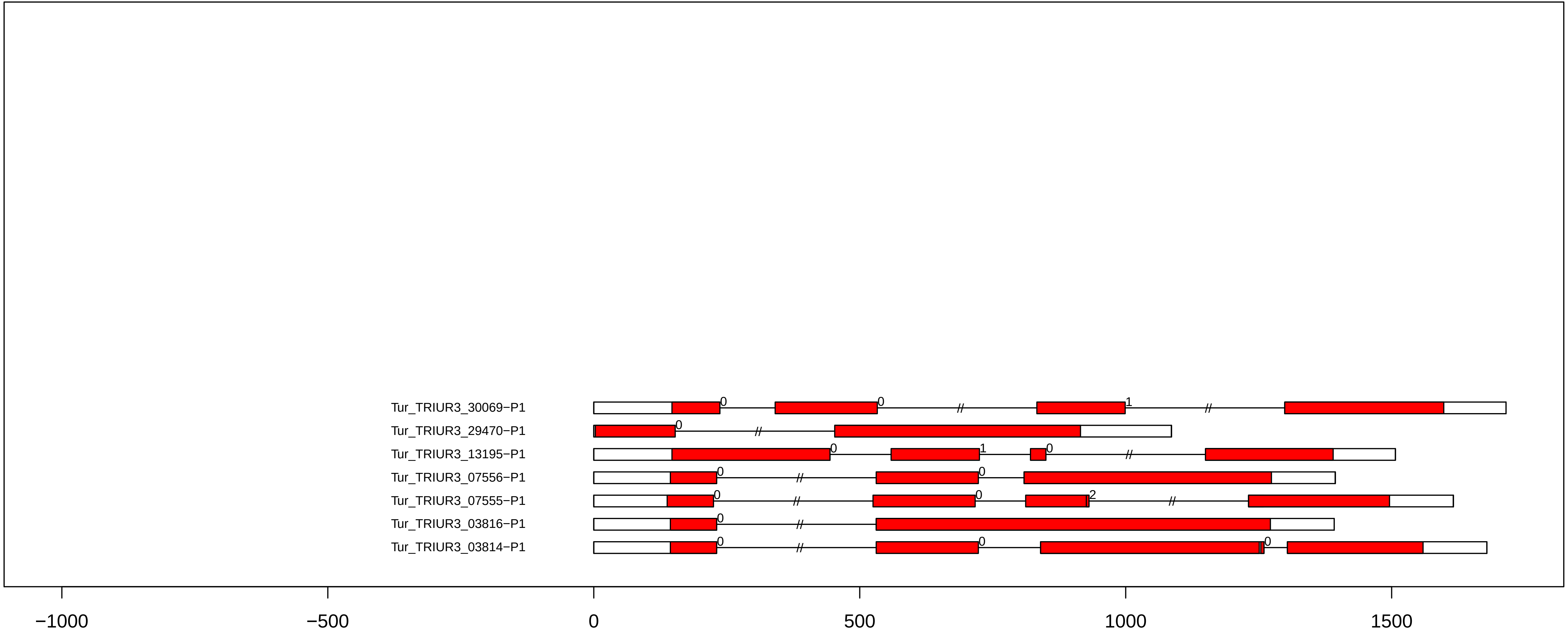

T.ur class III peroxidase XV subfamily exon−intron and prx domain diagram (all)

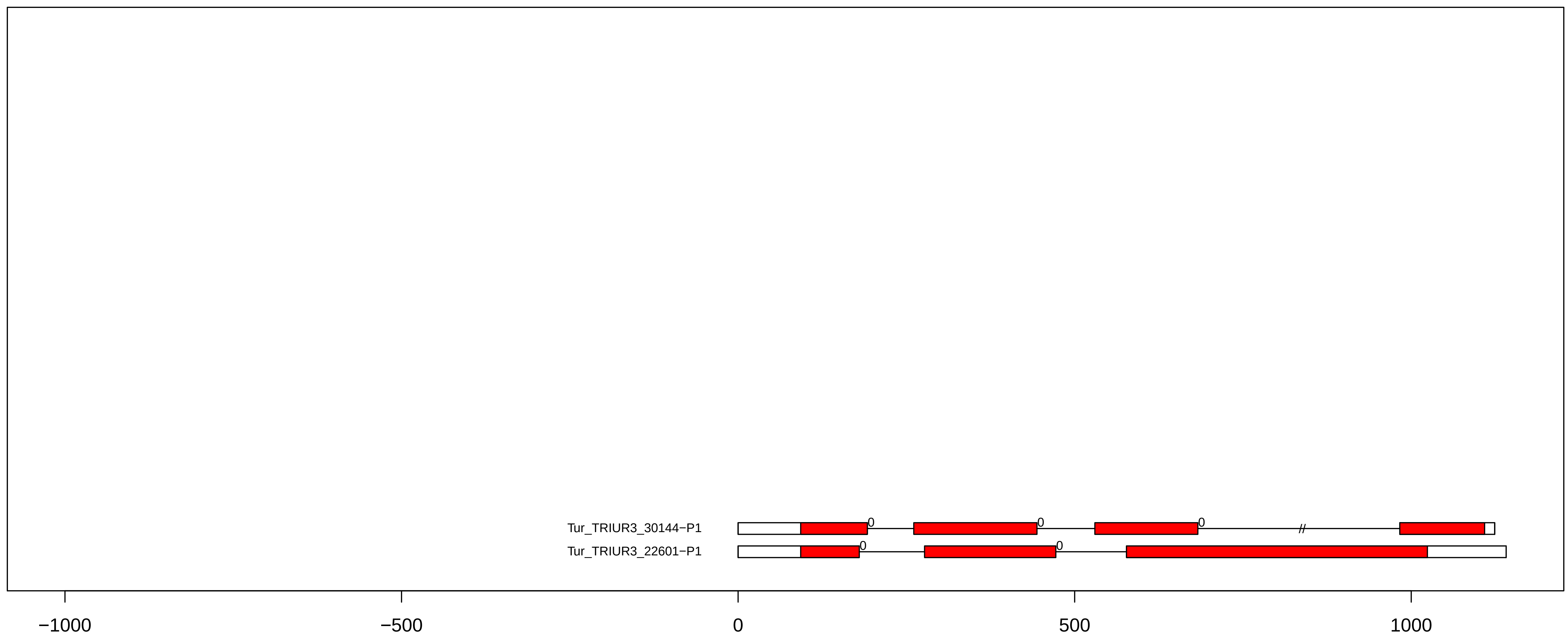

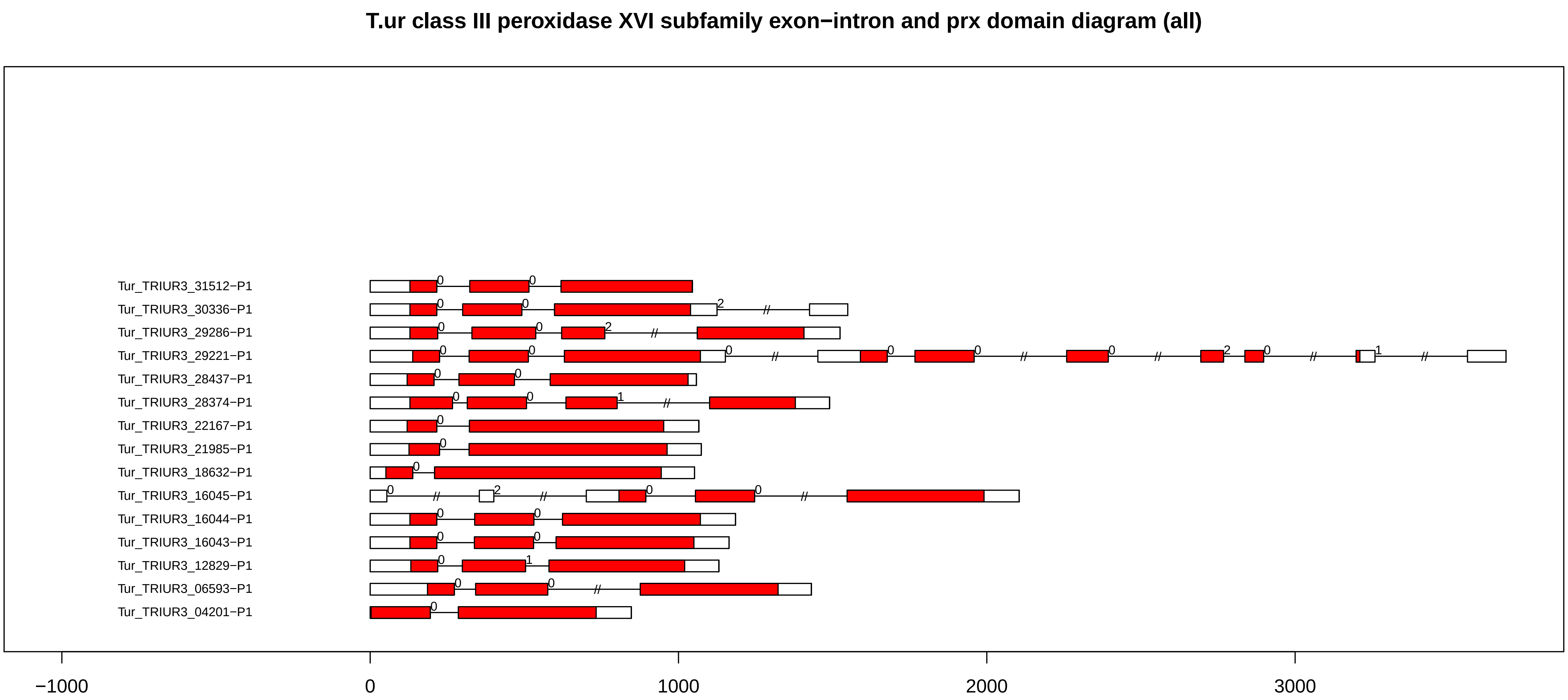
T.ur class III peroxidase XVI subfamily exon−intron and prx domain diagram (all)
Tur_TRIUR3_31512−P1
Tur_TRIUR3_30336−P1
Tur_TRIUR3_29286−P1
Tur_TRIUR3_29221−P1
Tur_TRIUR3_28437−P1
Tur_TRIUR3_28374−P1
Tur_TRIUR3_22167−P1
Tur_TRIUR3_21985−P1
Tur_TRIUR3_18632−P1
Tur_TRIUR3_16045−P1
Tur_TRIUR3_16044−P1
Tur_TRIUR3_16043−P1
Tur_TRIUR3_12829−P1
Tur_TRIUR3_06593−P1
Tur_TRIUR3_04201−P1
−1000
0
1000
2000
3000

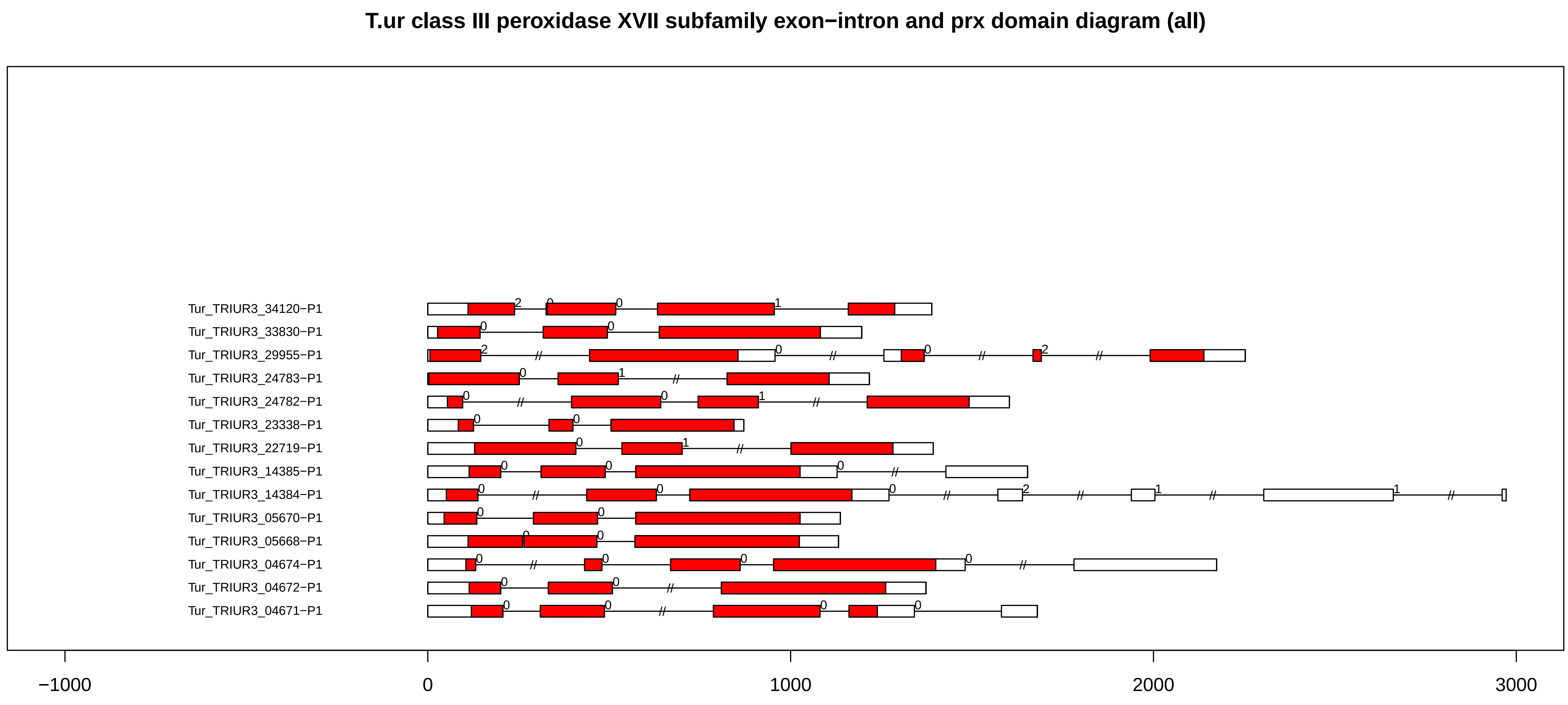
T.ur class III peroxidase XVII subfamily exon−intron and prx domain diagram (all)
Tur_TRIUR3_34120−P1
Tur_TRIUR3_33830−P1
Tur_TRIUR3_29955−P1
Tur_TRIUR3_24783−P1
Tur_TRIUR3_24782−P1
Tur_TRIUR3_23338−P1
Tur_TRIUR3_22719−P1
Tur_TRIUR3_14385−P1
Tur_TRIUR3_14384−P1
Tur_TRIUR3_05670−P1
Tur_TRIUR3_05668−P1
Tur_TRIUR3_04674−P1
Tur_TRIUR3_04672−P1
Tur_TRIUR3_04671−P1
−1000
0
1000
2000
3000

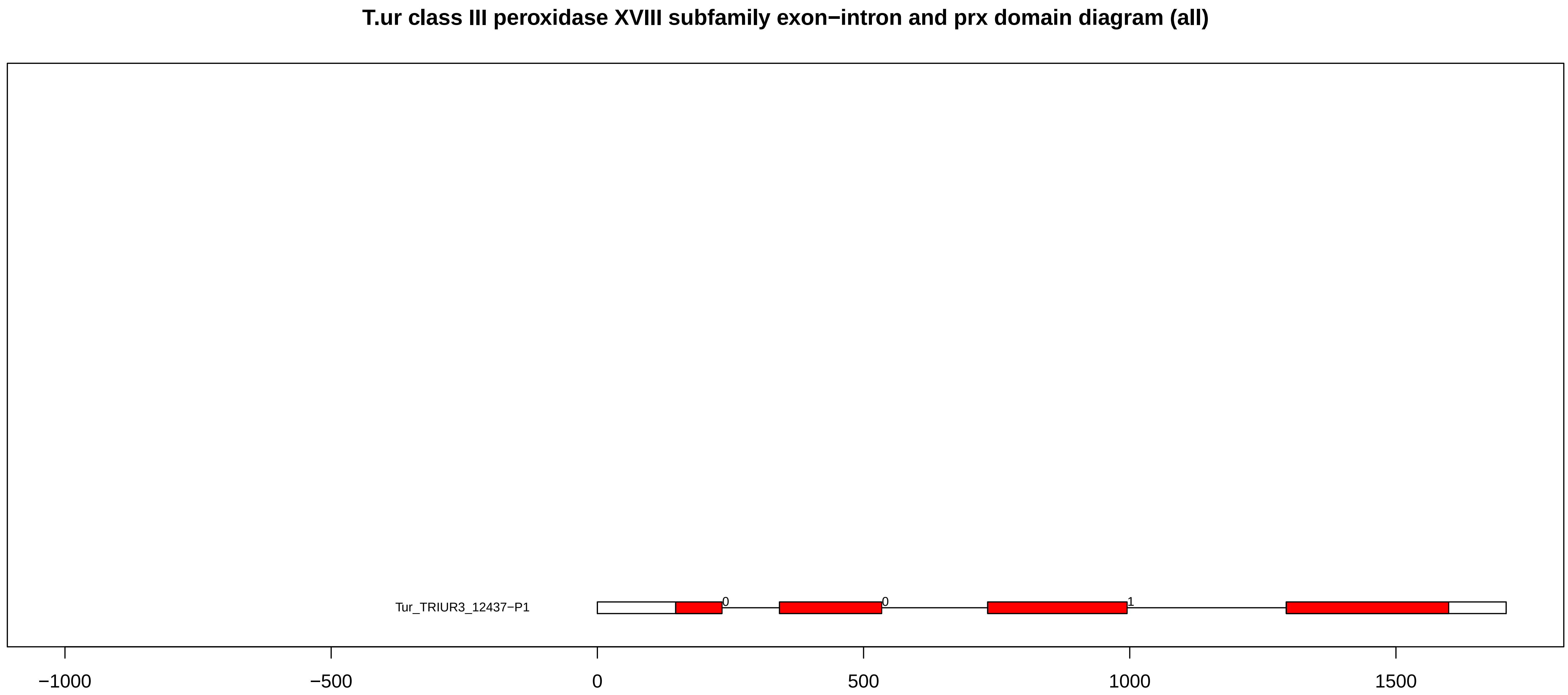

T.ur class III peroxidase XVIII subfamily exon−intron and prx domain diagram (all)
Tur_TRIUR3_12437−P1
0
0
1
−1000
−500
0
500
1000
1500

T.ur class III peroxidase unclear_classifications subfamily exon−intron and prx domain diagram (all)

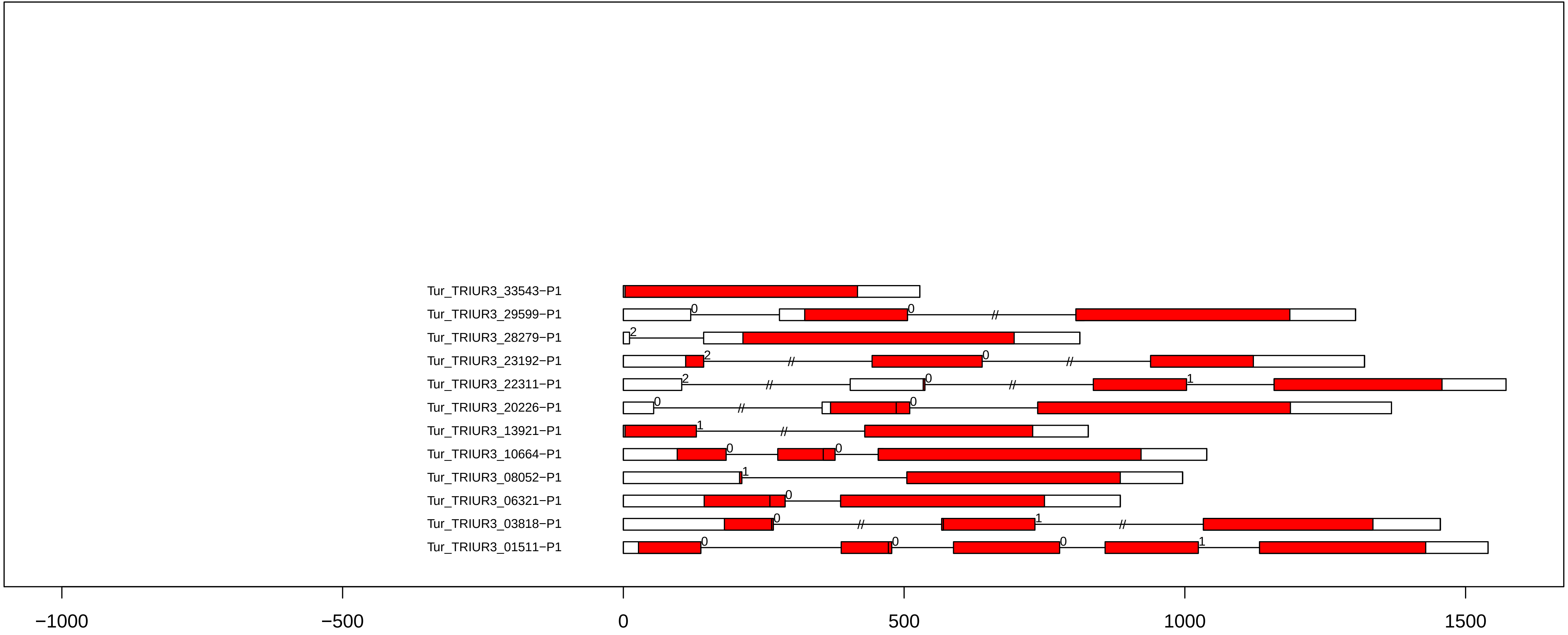

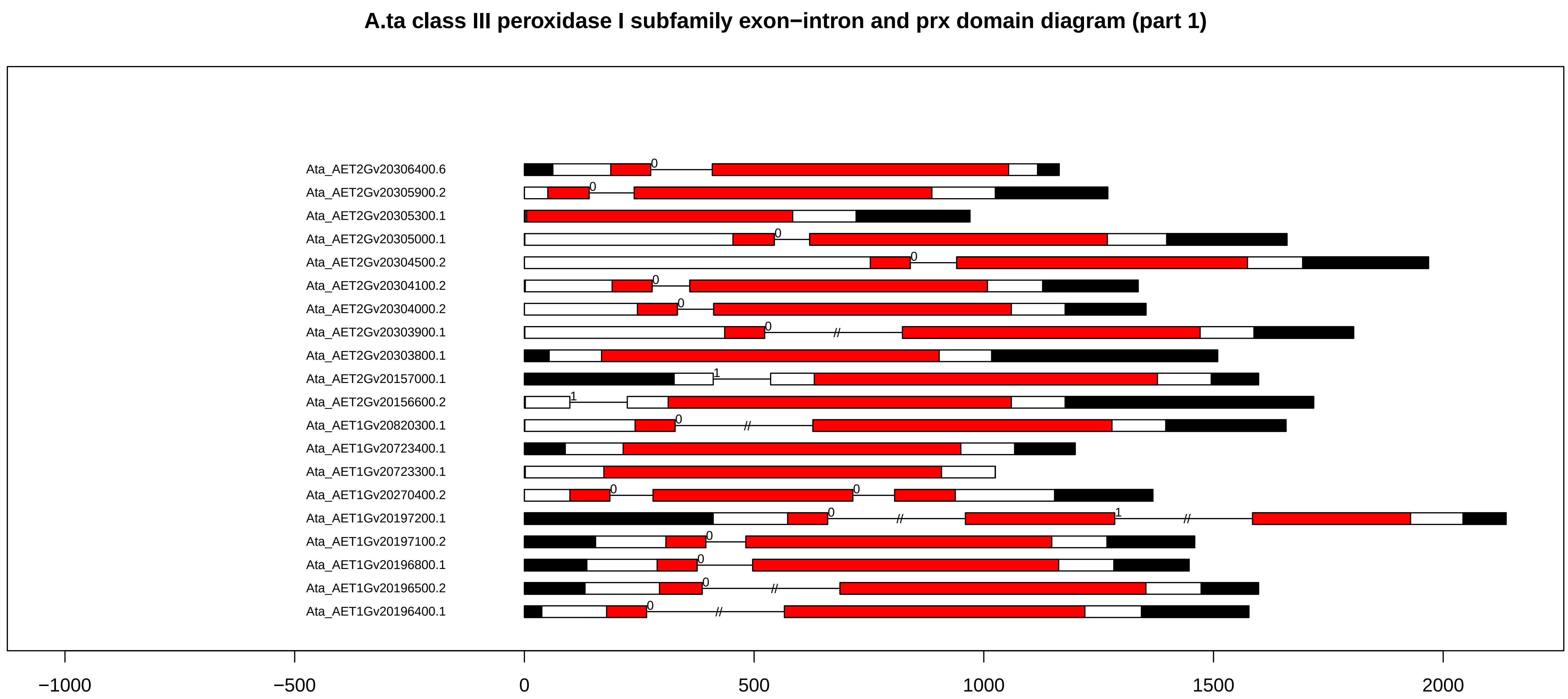
A.ta class III peroxidase I subfamily exon−intron and prx domain diagram (part 1)
Ata_AET2Gv20306400.6
Ata_AET2Gv20305900.2
Ata_AET2Gv20305300.1
Ata_AET2Gv20305000.1
Ata_AET2Gv20304500.2
Ata_AET2Gv20304100.2
Ata_AET2Gv20304000.2
Ata_AET2Gv20303900.1
Ata_AET2Gv20303800.1
Ata_AET2Gv20157000.1
Ata_AET2Gv20156600.2
Ata_AET1Gv20820300.1
Ata_AET1Gv20723400.1
Ata_AET1Gv20723300.1
Ata_AET1Gv20270400.2
Ata_AET1Gv20197200.1
Ata_AET1Gv20197100.2
Ata_AET1Gv20196800.1
Ata_AET1Gv20196500.2
Ata_AET1Gv20196400.1
−1000
−500
0
500
1000
1500
2000

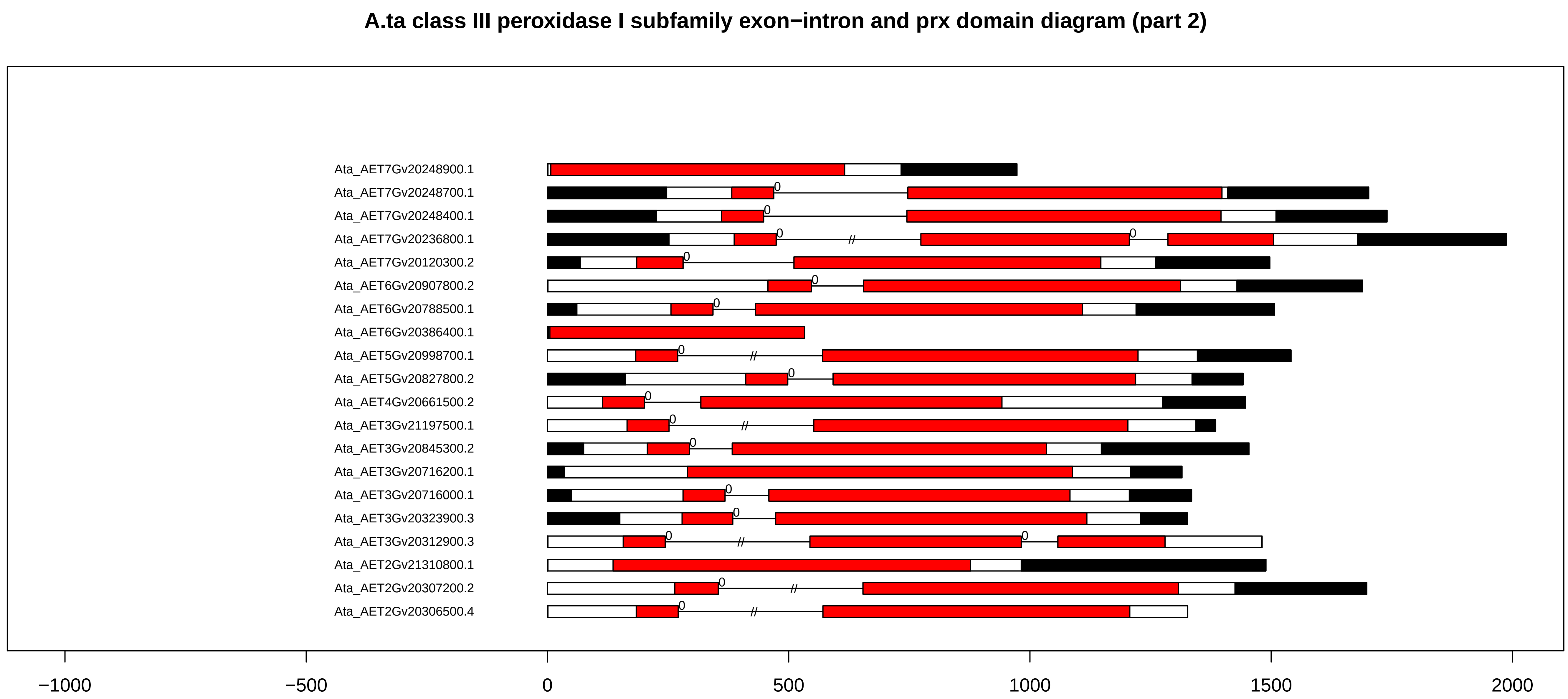

A.ta class III peroxidase I subfamily exon−intron and prx domain diagram (part 2)
Ata_AET7Gv20248900.1
Ata_AET7Gv20248700.1
Ata_AET7Gv20248400.1
Ata_AET7Gv20236800.1
Ata_AET7Gv20120300.2
Ata_AET6Gv20907800.2
Ata_AET6Gv20788500.1
Ata_AET6Gv20386400.1
Ata_AET5Gv20998700.1
Ata_AET5Gv20827800.2
Ata_AET4Gv20661500.2
Ata_AET3Gv21197500.1
Ata_AET3Gv20845300.2
Ata_AET3Gv20716200.1
Ata_AET3Gv20716000.1
Ata_AET3Gv20323900.3
Ata_AET3Gv20312900.3
Ata_AET2Gv21310800.1
Ata_AET2Gv20307200.2
Ata_AET2Gv20306500.4
−1000
−500
0
500
1000
1500
2000

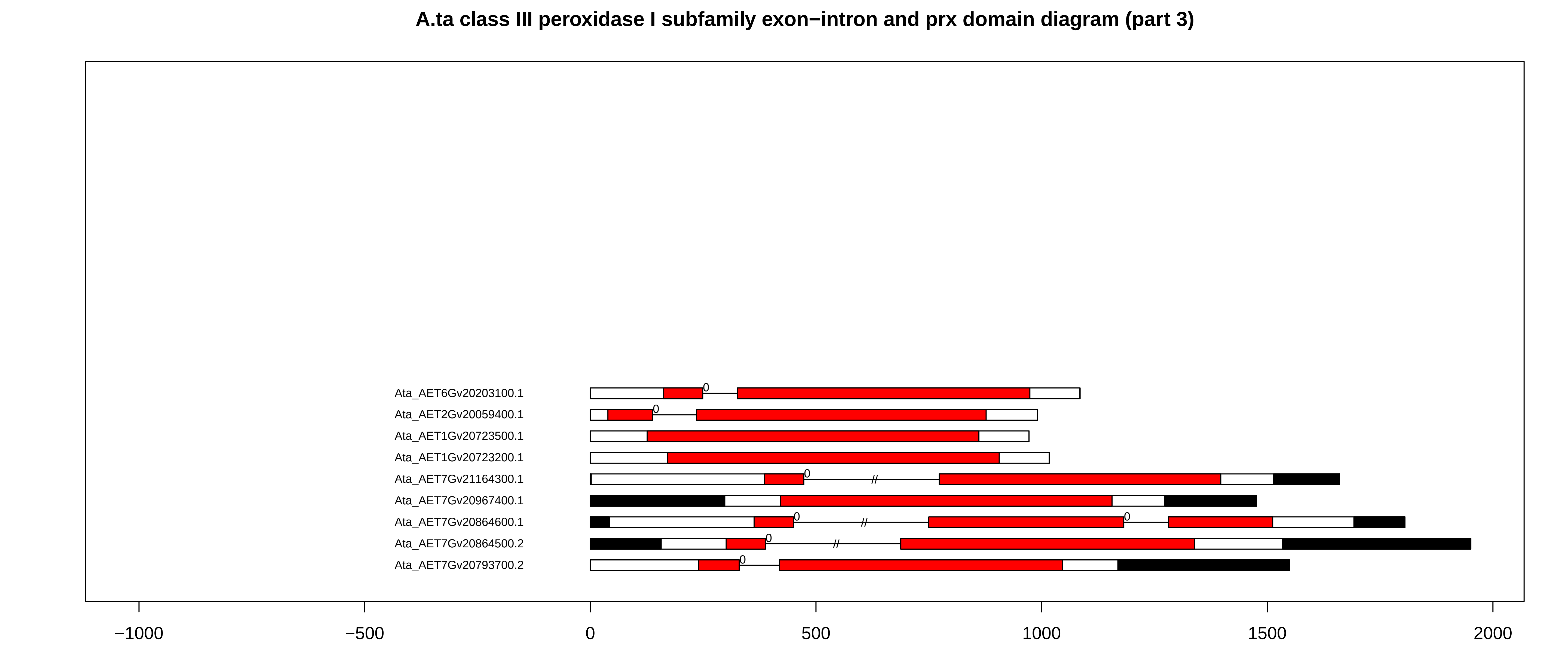
A.ta class III peroxidase I subfamily exon−intron and prx domain diagram (part 3)
Ata_AET6Gv20203100.1
Ata_AET2Gv20059400.1
Ata_AET1Gv20723500.1
Ata_AET1Gv20723200.1
Ata_AET7Gv21164300.1
Ata_AET7Gv20967400.1
Ata_AET7Gv20864600.1
Ata_AET7Gv20864500.2
Ata_AET7Gv20793700.2
−1000
−500
0
500
1000
1500
2000

A.ta class III peroxidase II subfamily exon−intron and prx domain diagram (all)

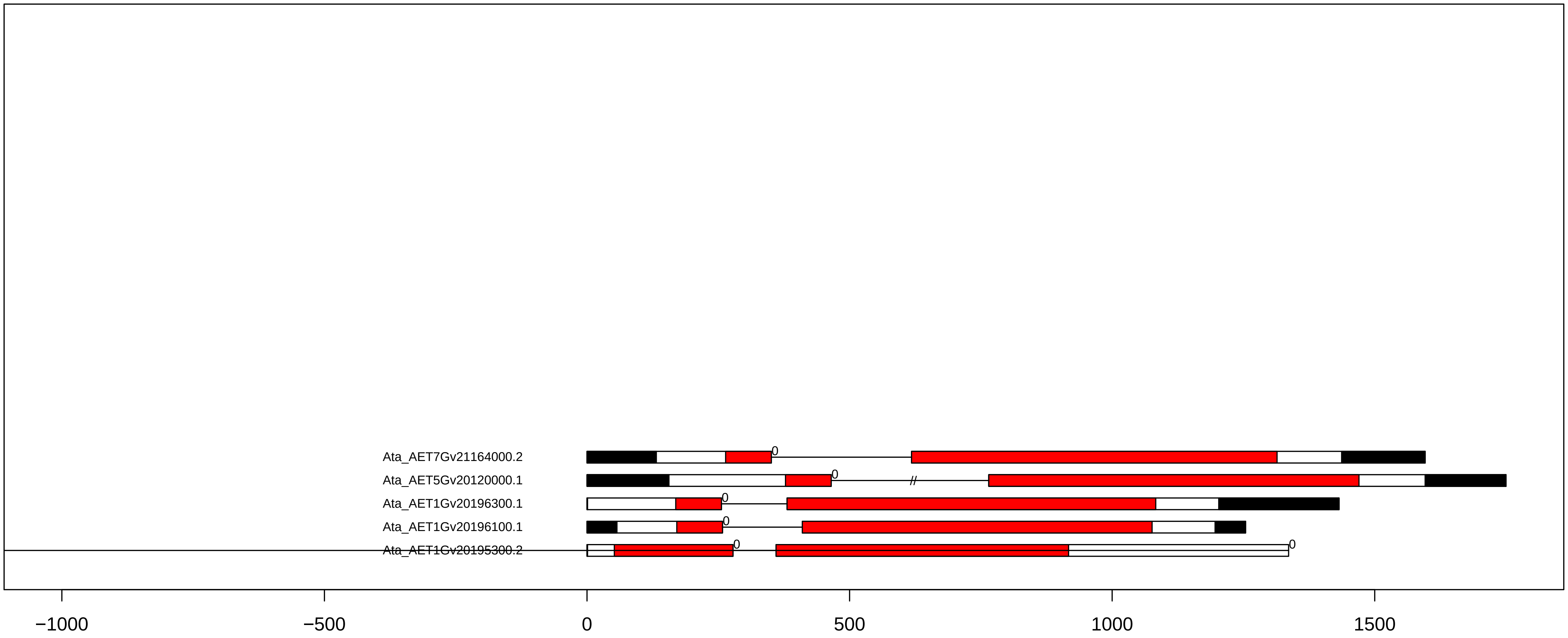

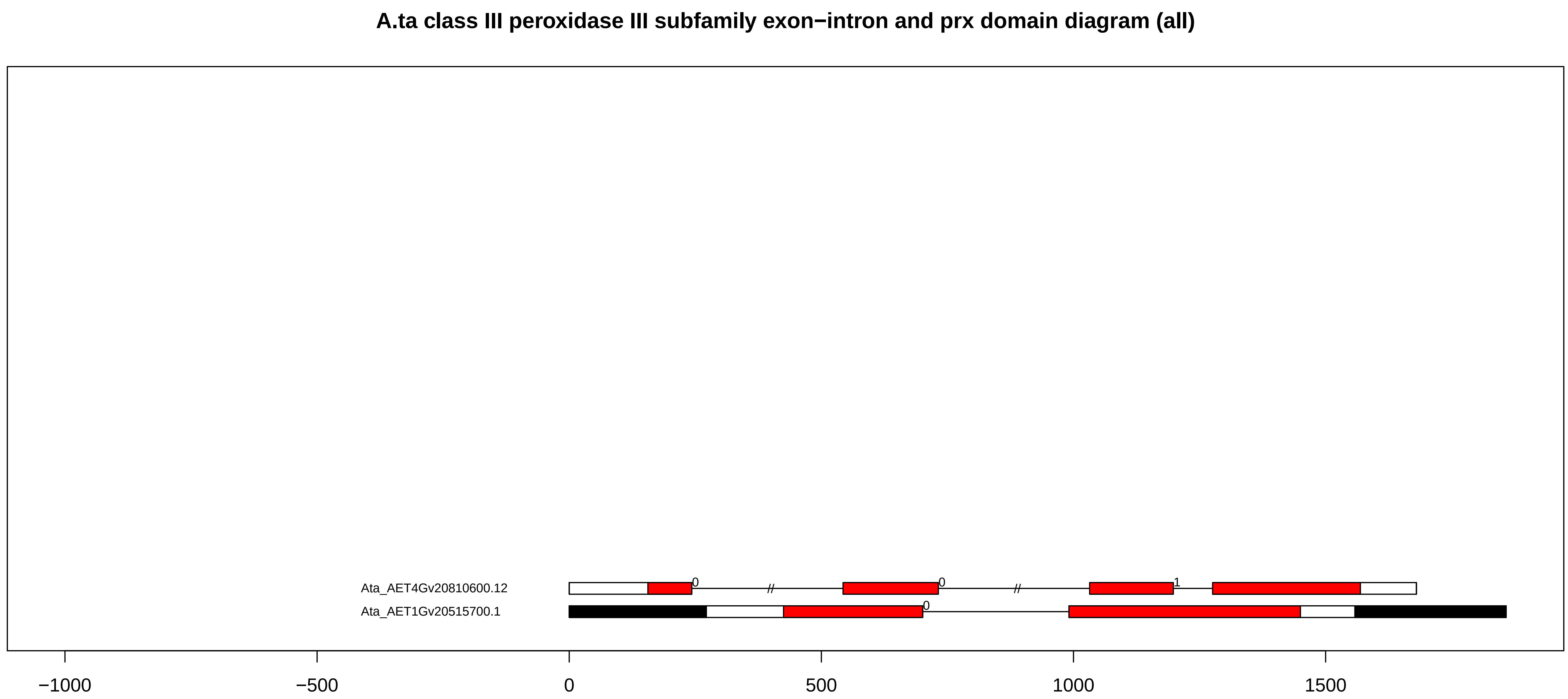
A.ta class III peroxidase III subfamily exon−intron and prx domain diagram (all)
Ata_AET4Gv20810600.12
Ata_AET1Gv20515700.1
0
0
1
0
−1000
−500
0
500
1000
1500

A.ta class III peroxidase V subfamily exon−intron and prx domain diagram (part 1)

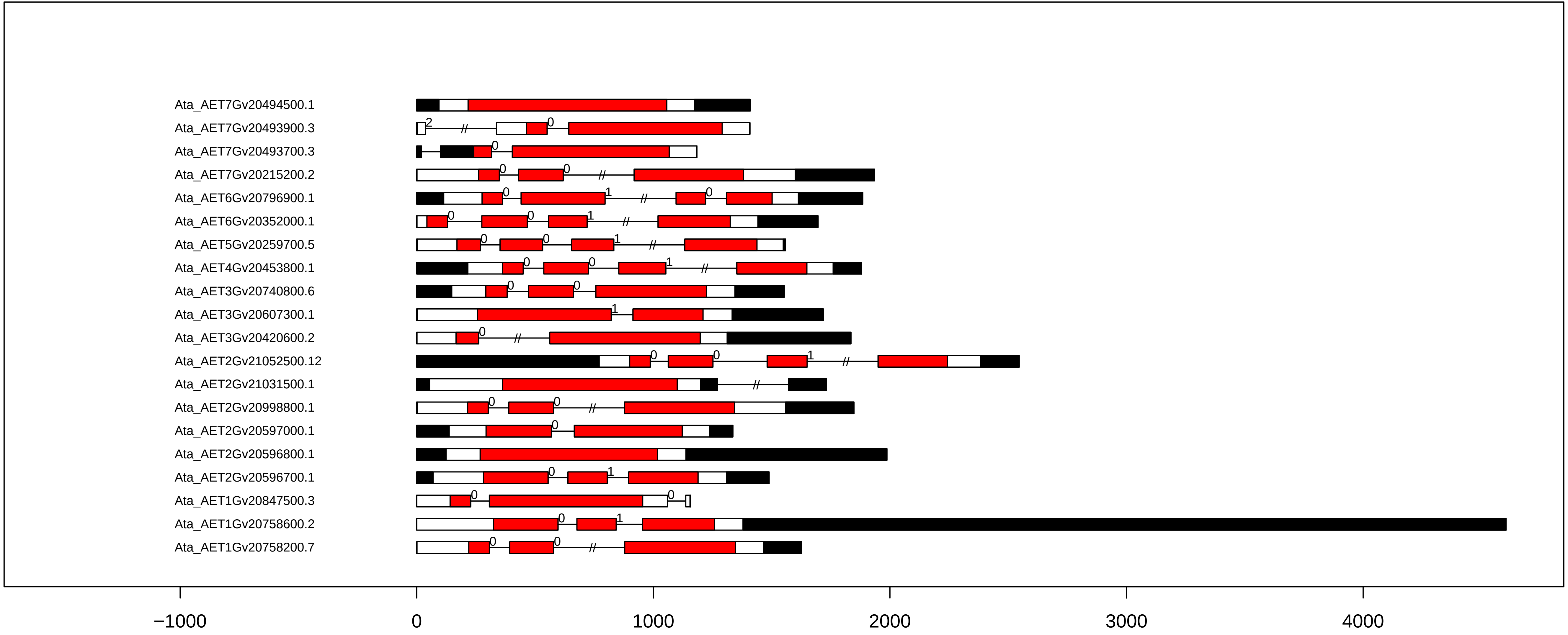

A.ta class III peroxidase V subfamily exon−intron and prx domain diagram (part 2)

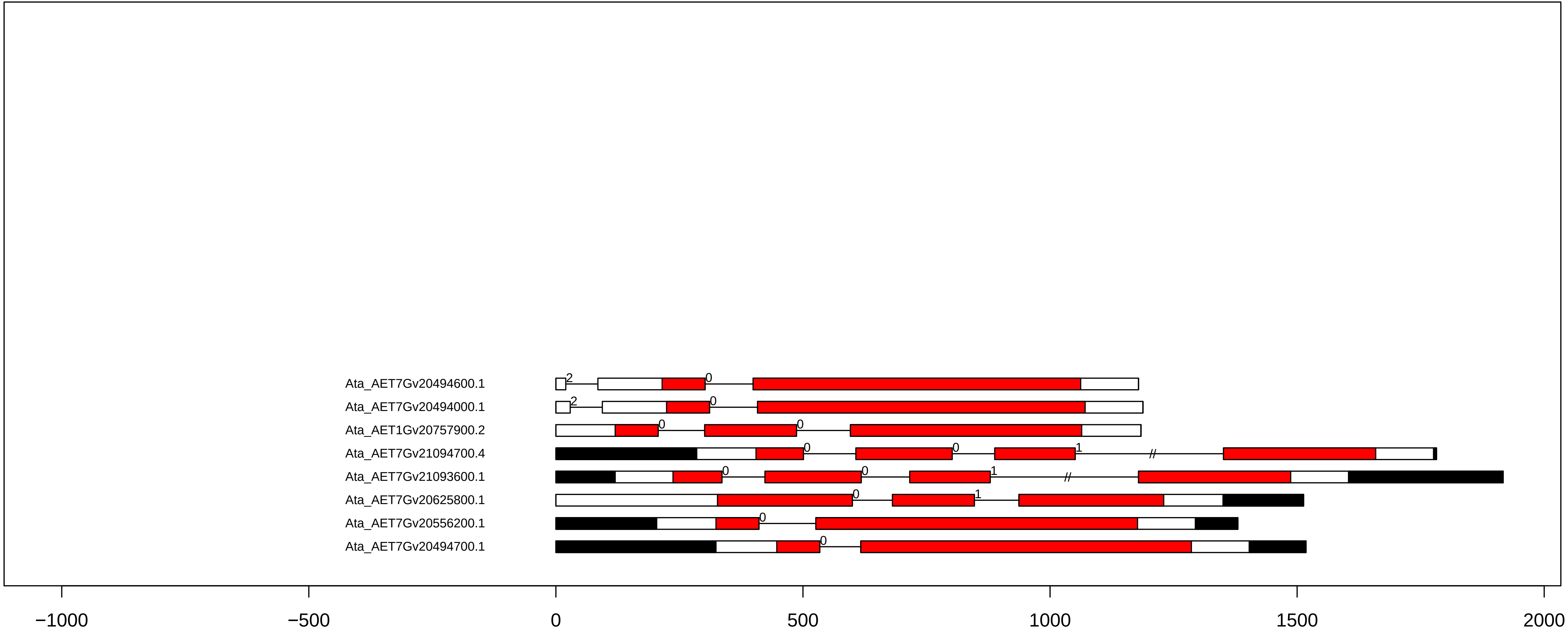

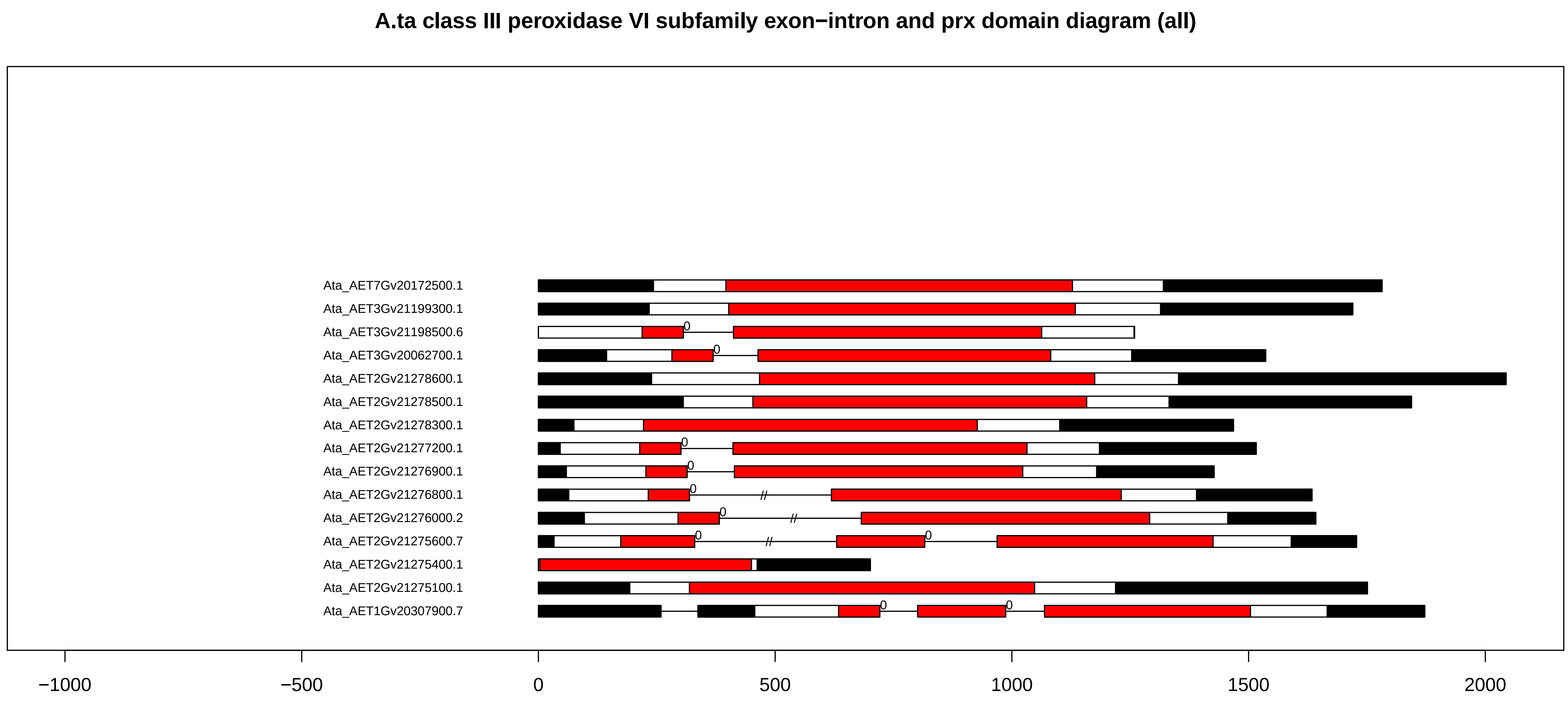
A.ta class III peroxidase VI subfamily exon−intron and prx domain diagram (all)
Ata_AET7Gv20172500.1
Ata_AET3Gv21199300.1
Ata_AET3Gv21198500.6
Ata_AET3Gv20062700.1
Ata_AET2Gv21278600.1
Ata_AET2Gv21278500.1
Ata_AET2Gv21278300.1
Ata_AET2Gv21277200.1
Ata_AET2Gv21276900.1
Ata_AET2Gv21276800.1
Ata_AET2Gv21276000.2
Ata_AET2Gv21275600.7
Ata_AET2Gv21275400.1
Ata_AET2Gv21275100.1
Ata_AET1Gv20307900.7
−1000
−500
0
500
1000
1500
2000

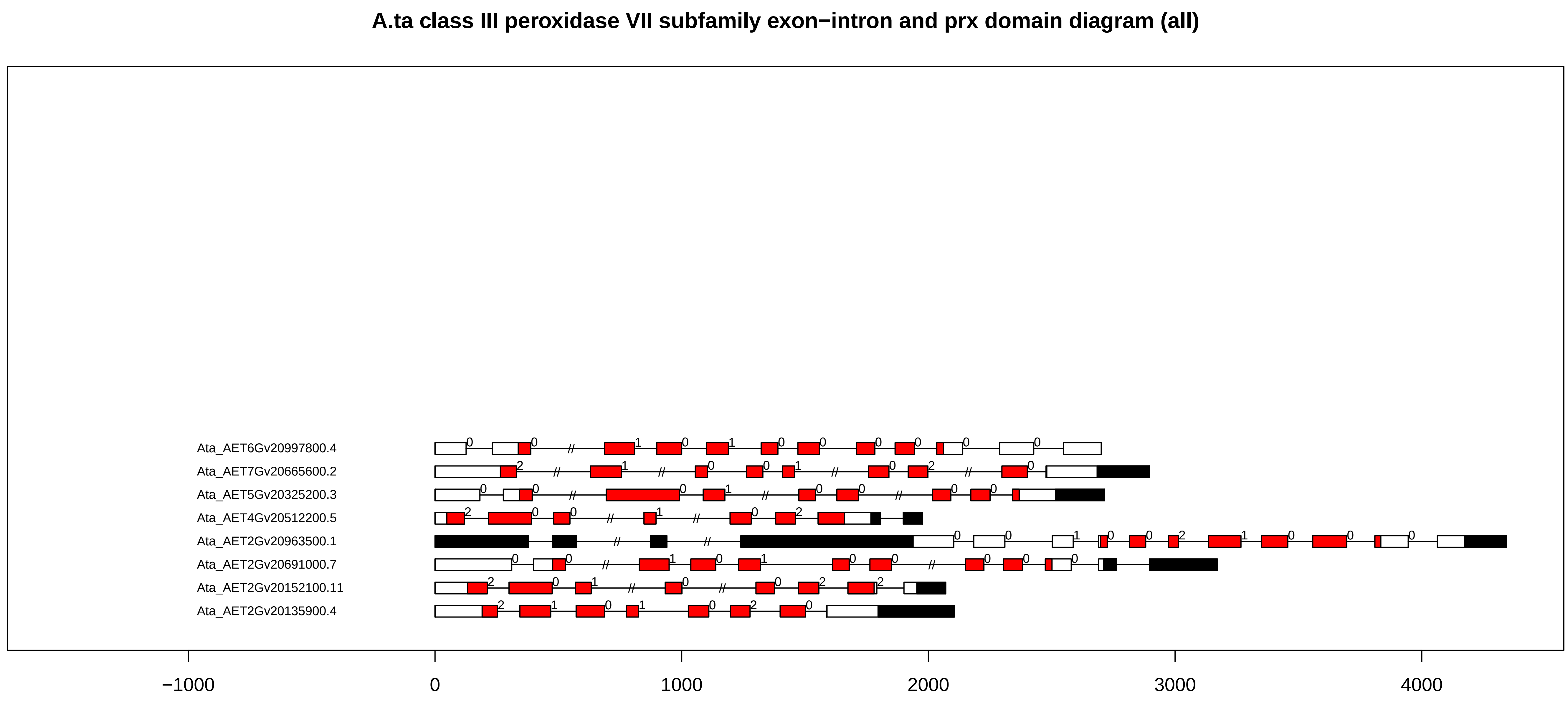

A.ta class III peroxidase VII subfamily exon−intron and prx domain diagram (all)
Ata_AET6Gv20997800.4
Ata_AET7Gv20665600.2
Ata_AET5Gv20325200.3
Ata_AET4Gv20512200.5
Ata_AET2Gv20963500.1
Ata_AET2Gv20691000.7
Ata_AET2Gv20152100.11
Ata_AET2Gv20135900.4
−1000
0
1000
2000
3000
4000

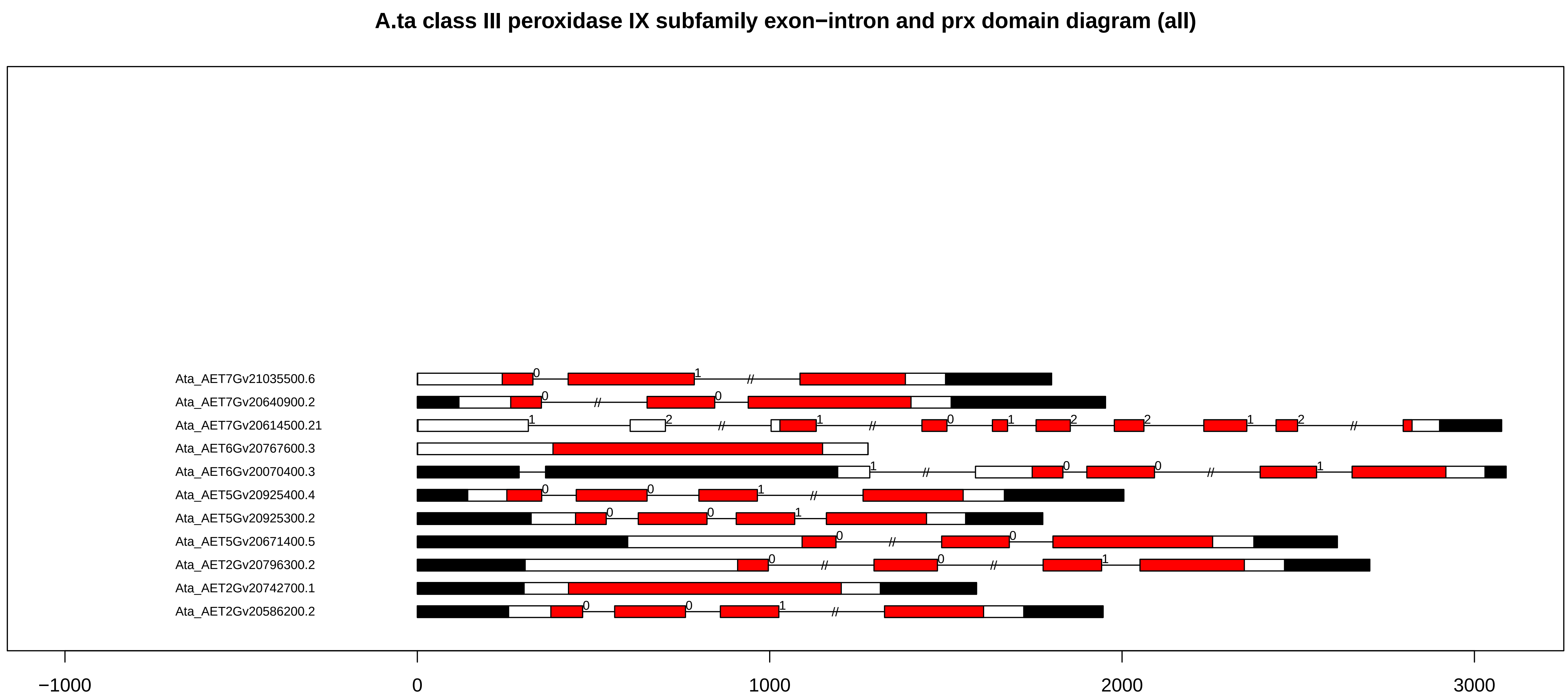

A.ta class III peroxidase IX subfamily exon−intron and prx domain diagram (all)
Ata_AET7Gv21035500.6
Ata_AET7Gv20640900.2
Ata_AET7Gv20614500.21
Ata_AET6Gv20767600.3
Ata_AET6Gv20070400.3
Ata_AET5Gv20925400.4
Ata_AET5Gv20925300.2
Ata_AET5Gv20671400.5
Ata_AET2Gv20796300.2
Ata_AET2Gv20742700.1
Ata_AET2Gv20586200.2
−1000
0
1000
2000
3000

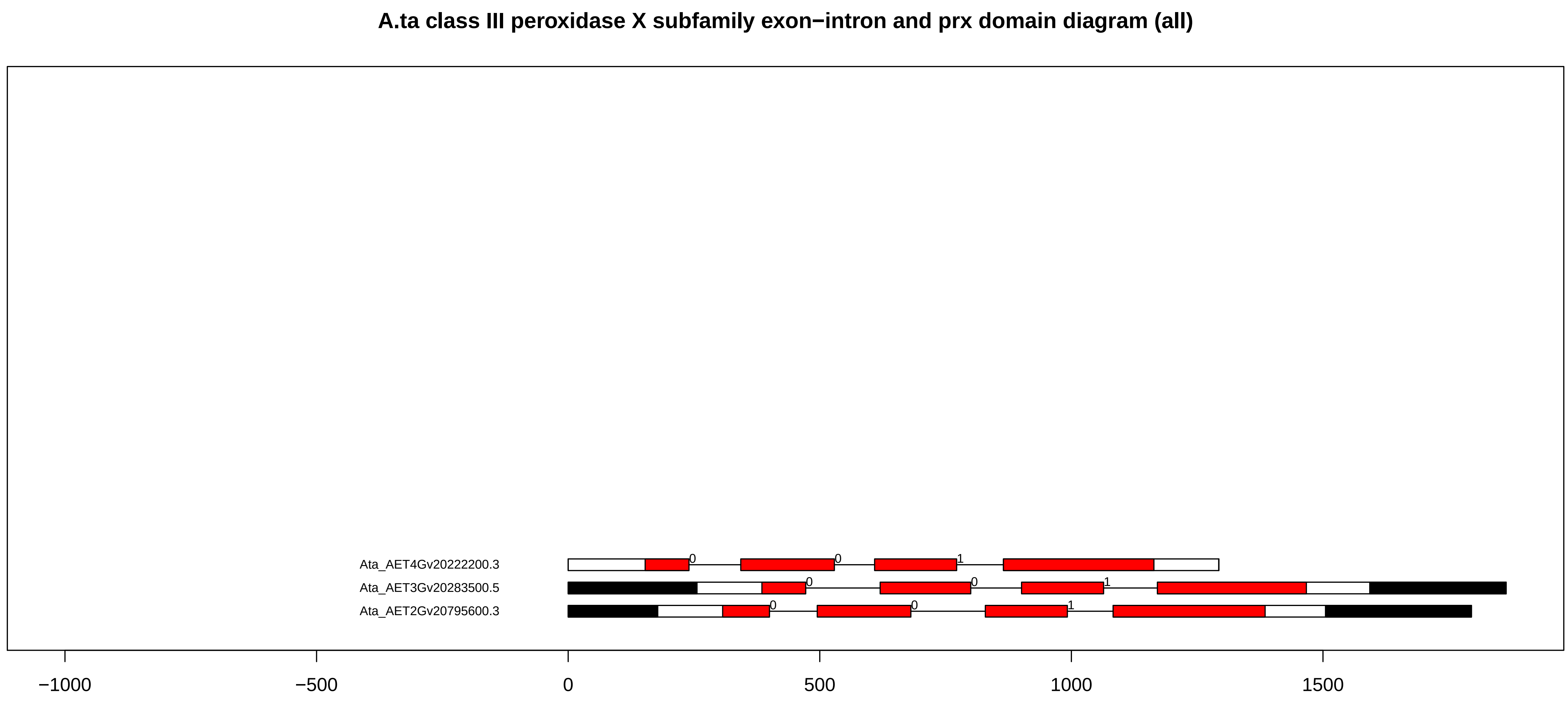
A.ta class III peroxidase X subfamily exon−intron and prx domain diagram (all)
Ata_AET4Gv20222200.3
Ata_AET3Gv20283500.5
Ata_AET2Gv20795600.3
0
0
1
0
0
1
0
0
1
−1000
−500
0
500
1000
1500

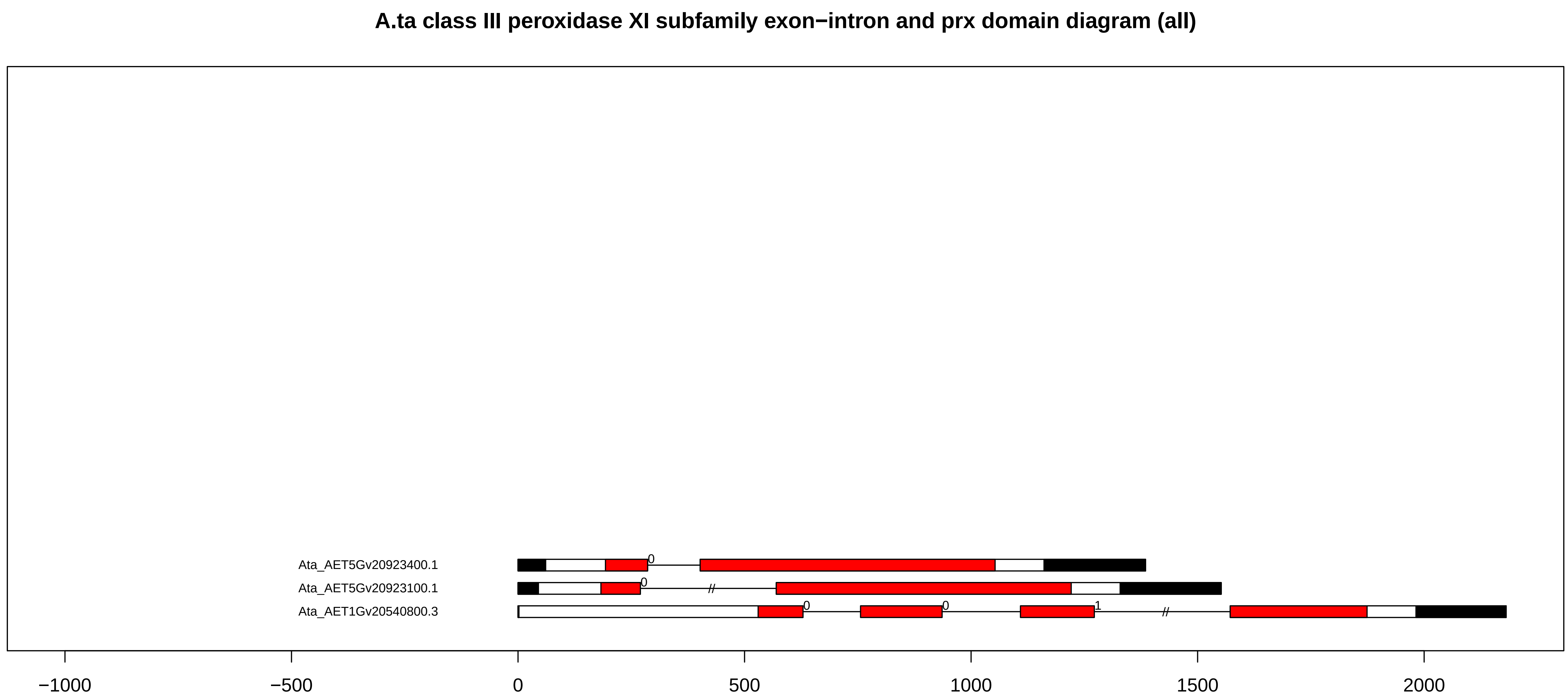
A.ta class III peroxidase XI subfamily exon−intron and prx domain diagram (all)
Ata_AET5Gv20923400.1
Ata_AET5Gv20923100.1
Ata_AET1Gv20540800.3
0
0
0
0
1
−1000
−500
0
500
1000
1500
2000

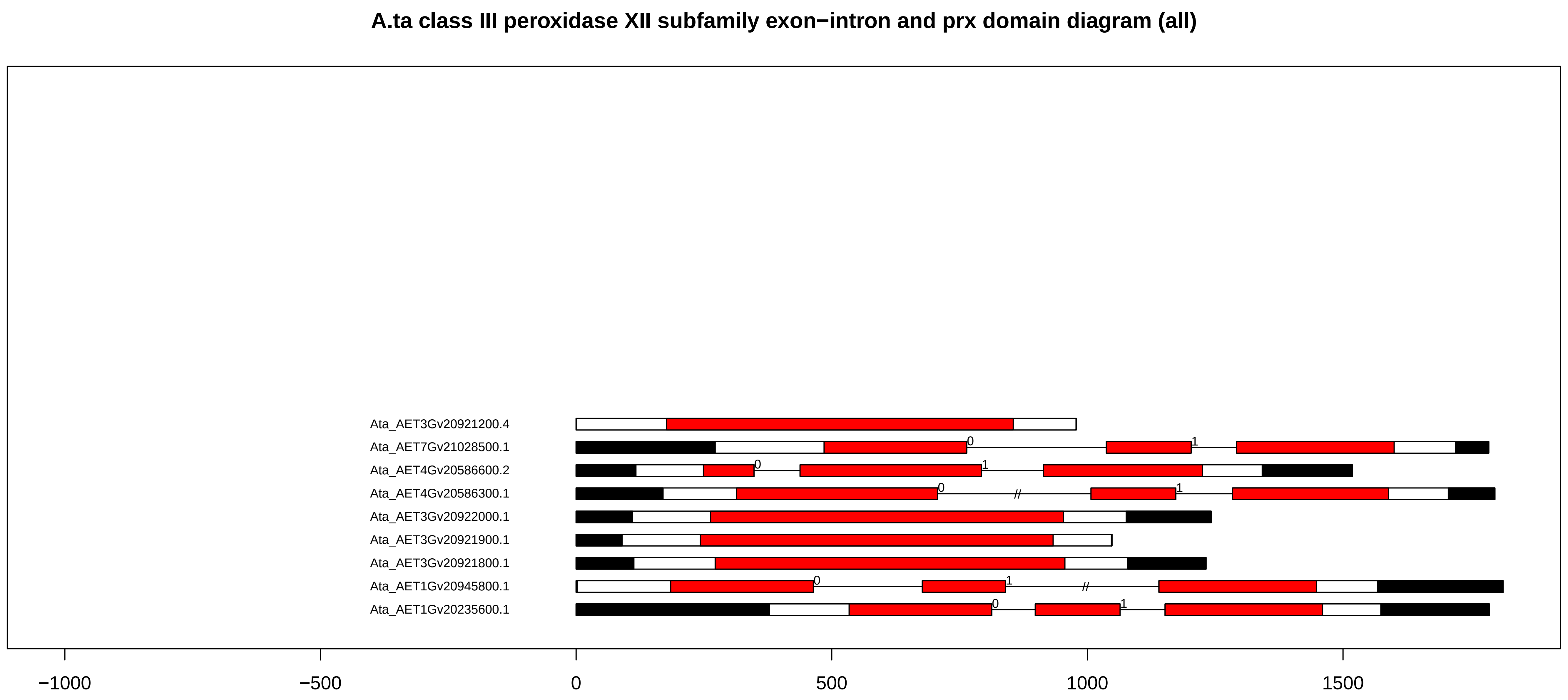

A.ta class III peroxidase XII subfamily exon−intron and prx domain diagram (all)
Ata_AET3Gv20921200.4
Ata_AET7Gv21028500.1
Ata_AET4Gv20586600.2
Ata_AET4Gv20586300.1
Ata_AET3Gv20922000.1
Ata_AET3Gv20921900.1
Ata_AET3Gv20921800.1
Ata_AET1Gv20945800.1
Ata_AET1Gv20235600.1
−1000
−500
0
500
1000
1500

A.ta class III peroxidase XIII subfamily exon−intron and prx domain diagram (all)

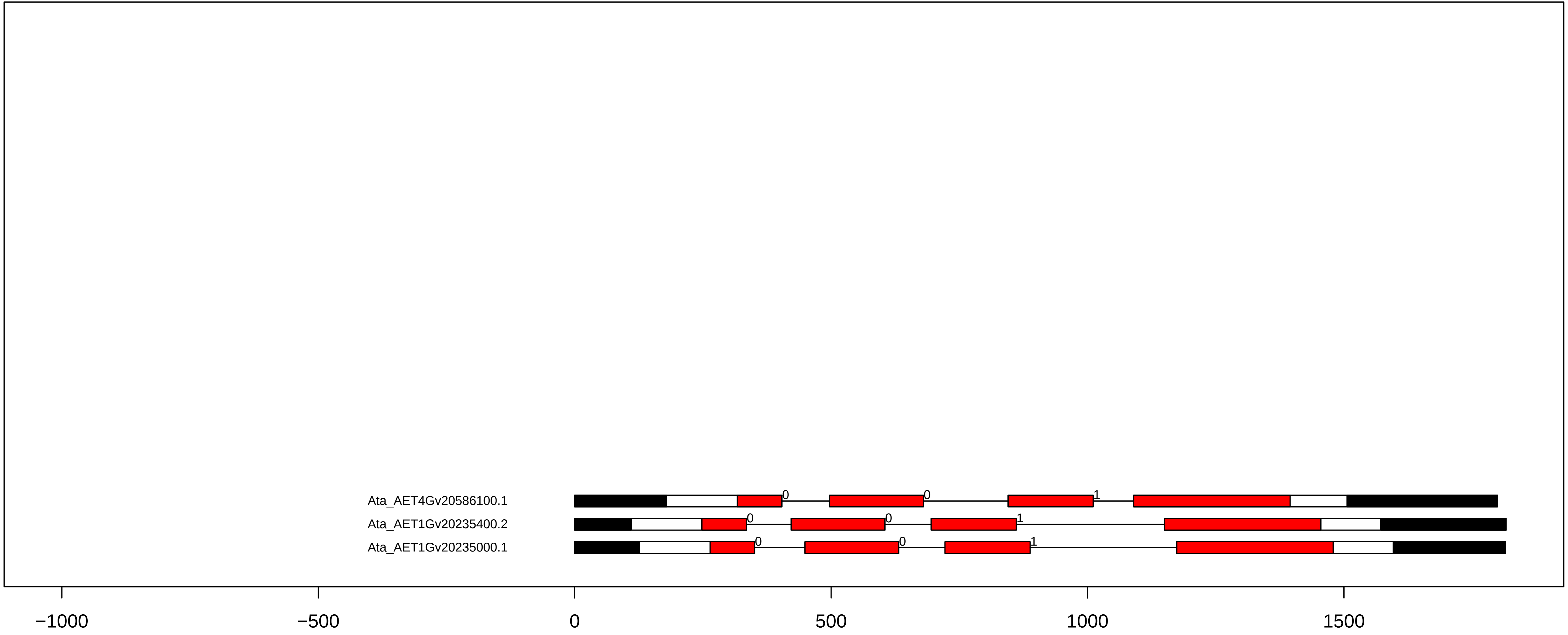

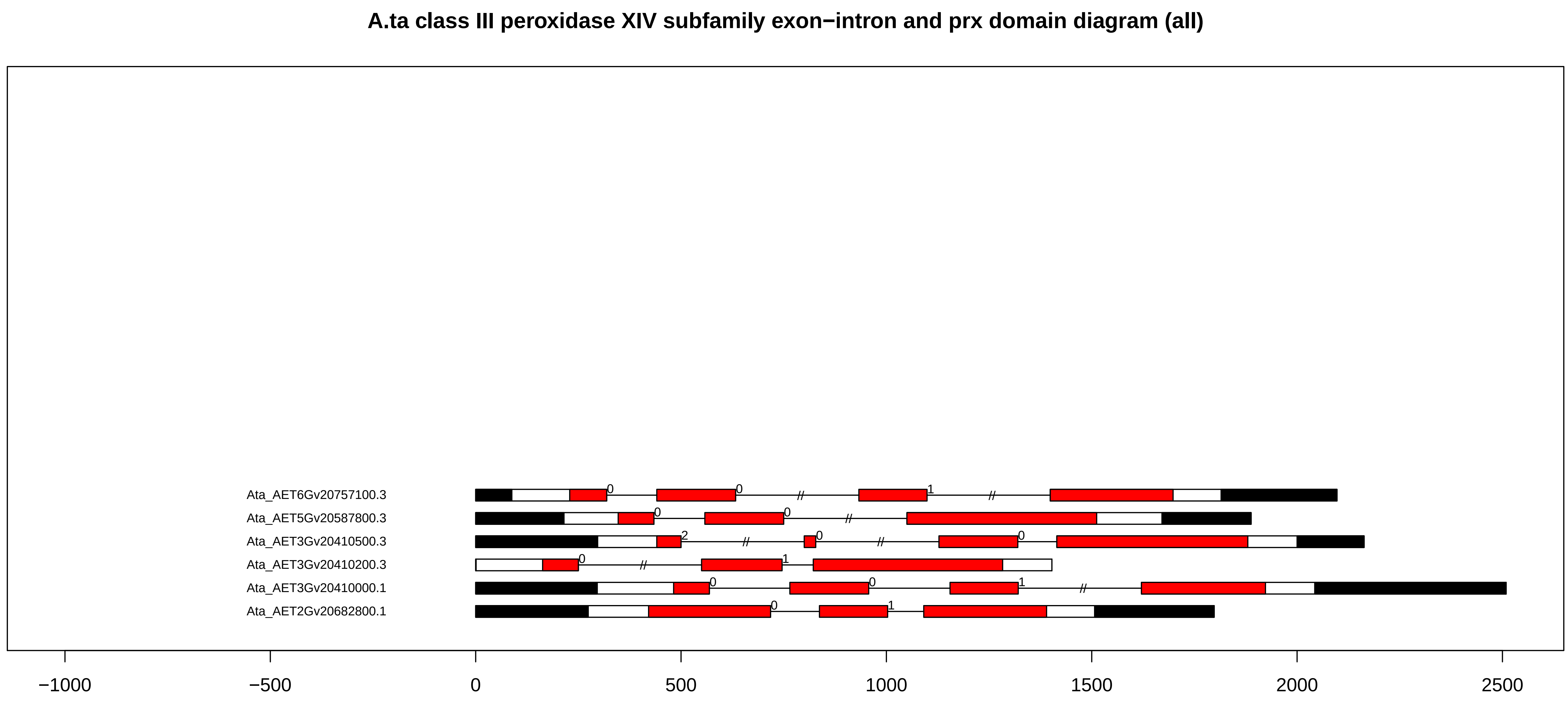
A.ta class III peroxidase XIV subfamily exon−intron and prx domain diagram (all)
Ata_AET6Gv20757100.3
Ata_AET5Gv20587800.3
Ata_AET3Gv20410500.3
Ata_AET3Gv20410200.3
Ata_AET3Gv20410000.1
Ata_AET2Gv20682800.1
−1000
−500
0
500
1000
1500
2000
2500

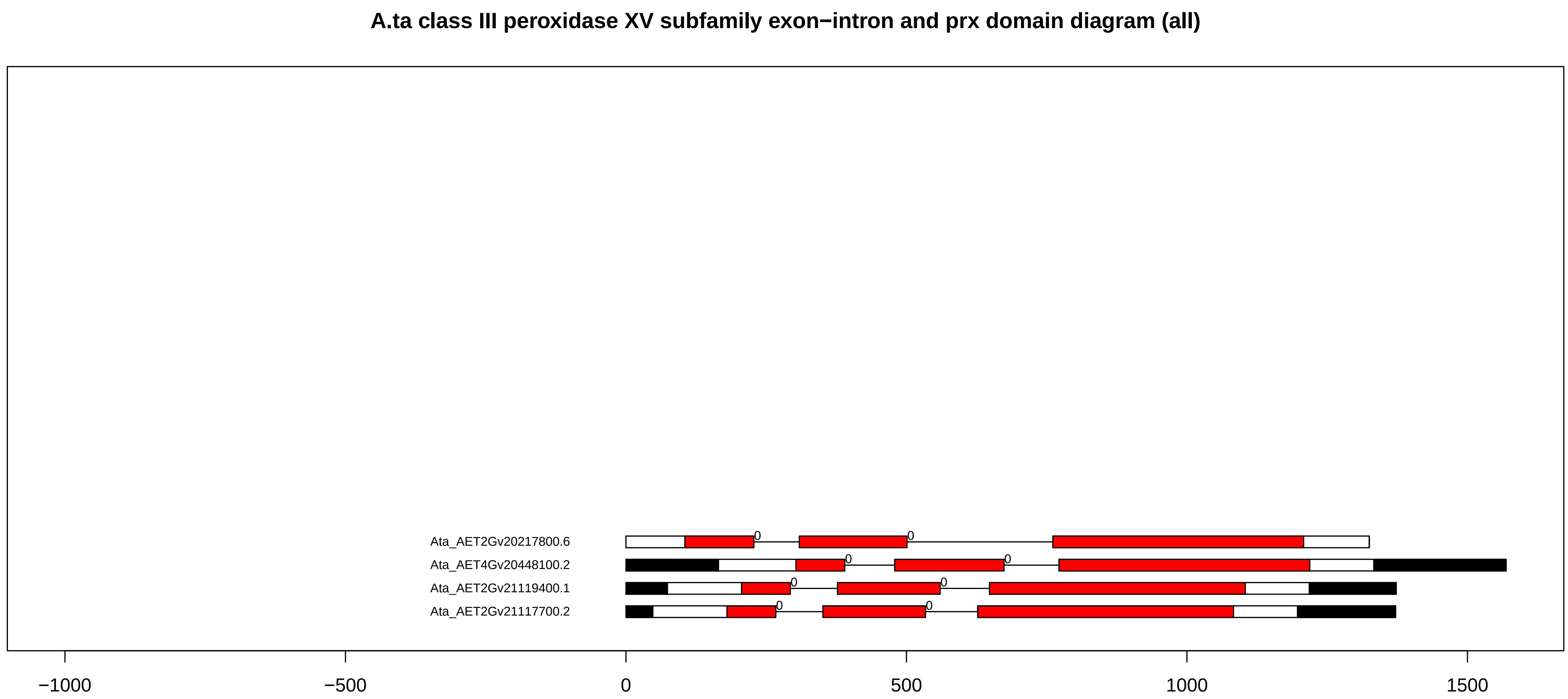
A.ta class III peroxidase XV subfamily exon−intron and prx domain diagram (all)
Ata_AET2Gv20217800.6
Ata_AET4Gv20448100.2
Ata_AET2Gv21119400.1
Ata_AET2Gv21117700.2
−1000
−500
0
500
1000
1500

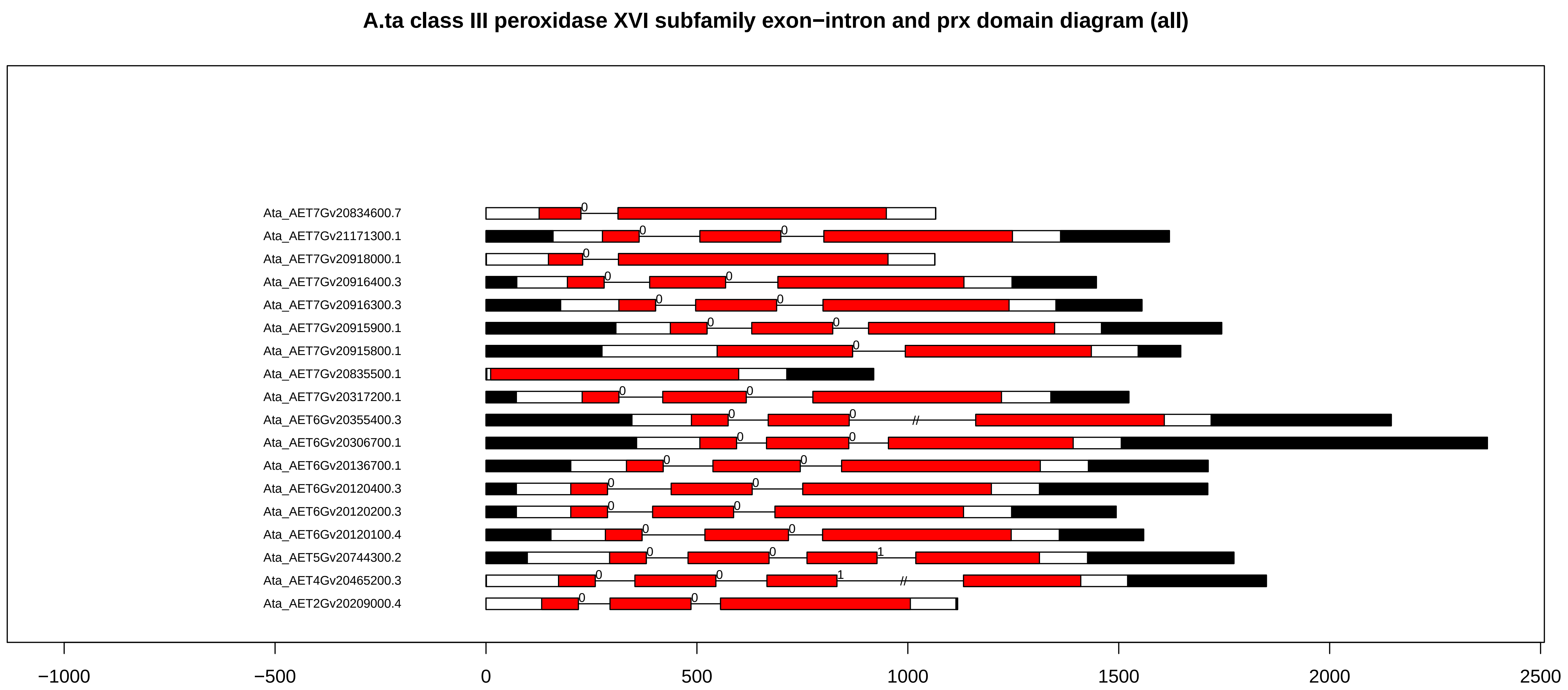
A.ta class III peroxidase XVI subfamily exon−intron and prx domain diagram (all)
Ata_AET7Gv20834600.7
Ata_AET7Gv21171300.1
Ata_AET7Gv20918000.1
Ata_AET7Gv20916400.3
Ata_AET7Gv20916300.3
Ata_AET7Gv20915900.1
Ata_AET7Gv20915800.1
Ata_AET7Gv20835500.1
Ata_AET7Gv20317200.1
Ata_AET6Gv20355400.3
Ata_AET6Gv20306700.1
Ata_AET6Gv20136700.1
Ata_AET6Gv20120400.3
Ata_AET6Gv20120200.3
Ata_AET6Gv20120100.4
Ata_AET5Gv20744300.2
Ata_AET4Gv20465200.3
Ata_AET2Gv20209000.4
−1000
−500
0
500
1000
1500
2000
2500

A.ta class III peroxidase XVII subfamily exon−intron and prx domain diagram (all)

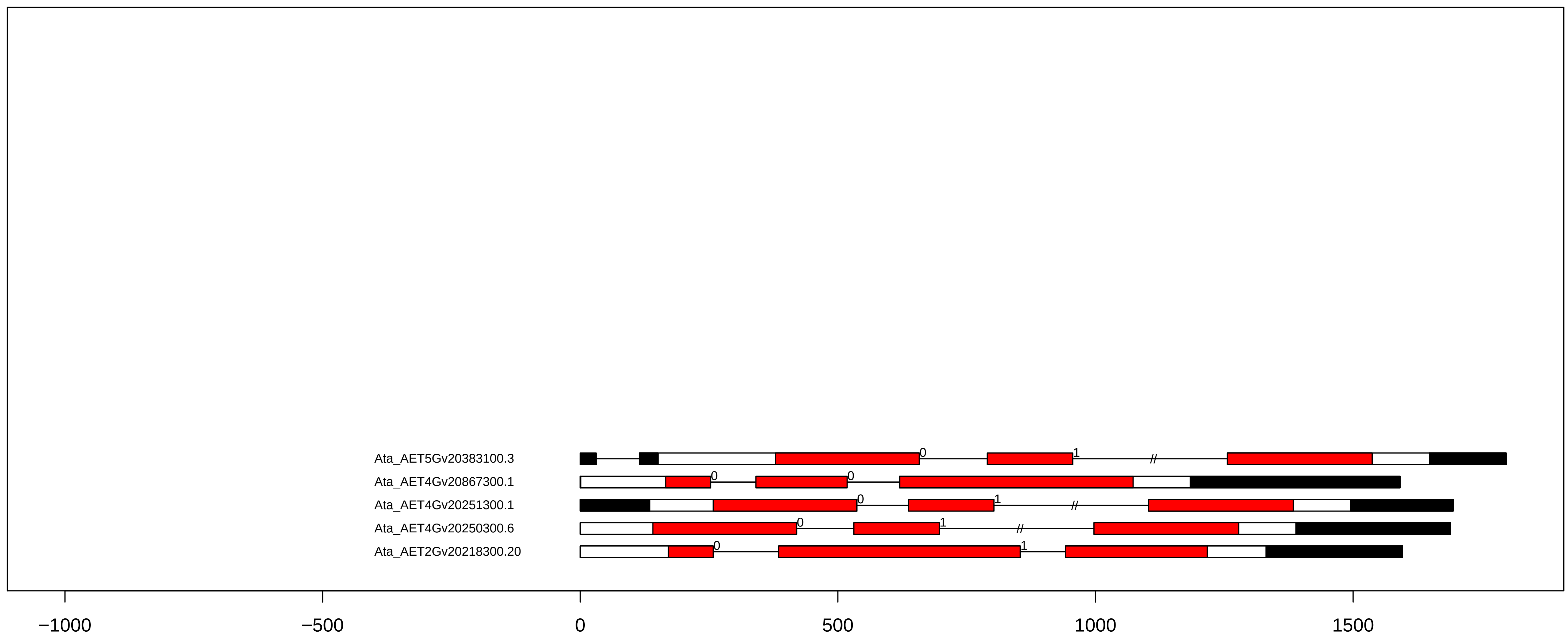

A.ta class III peroxidase XVIII subfamily exon−intron and prx domain diagram (all)

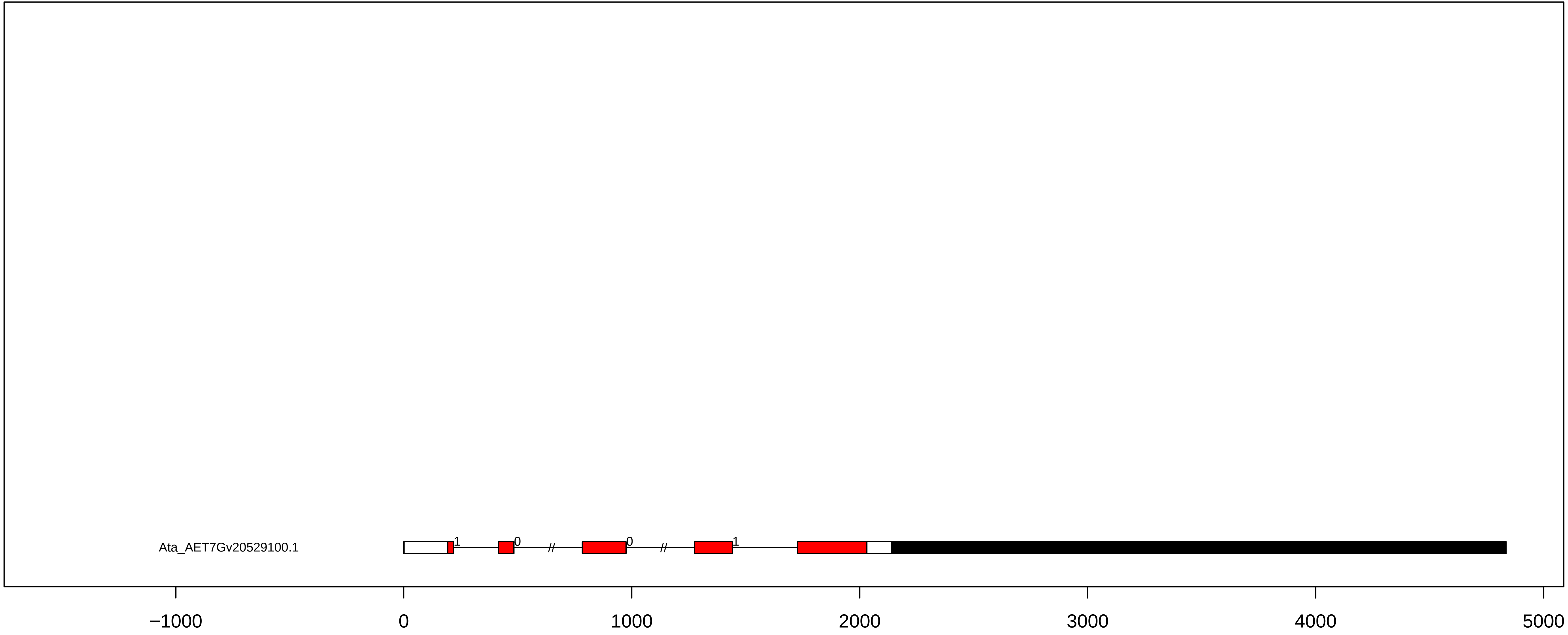

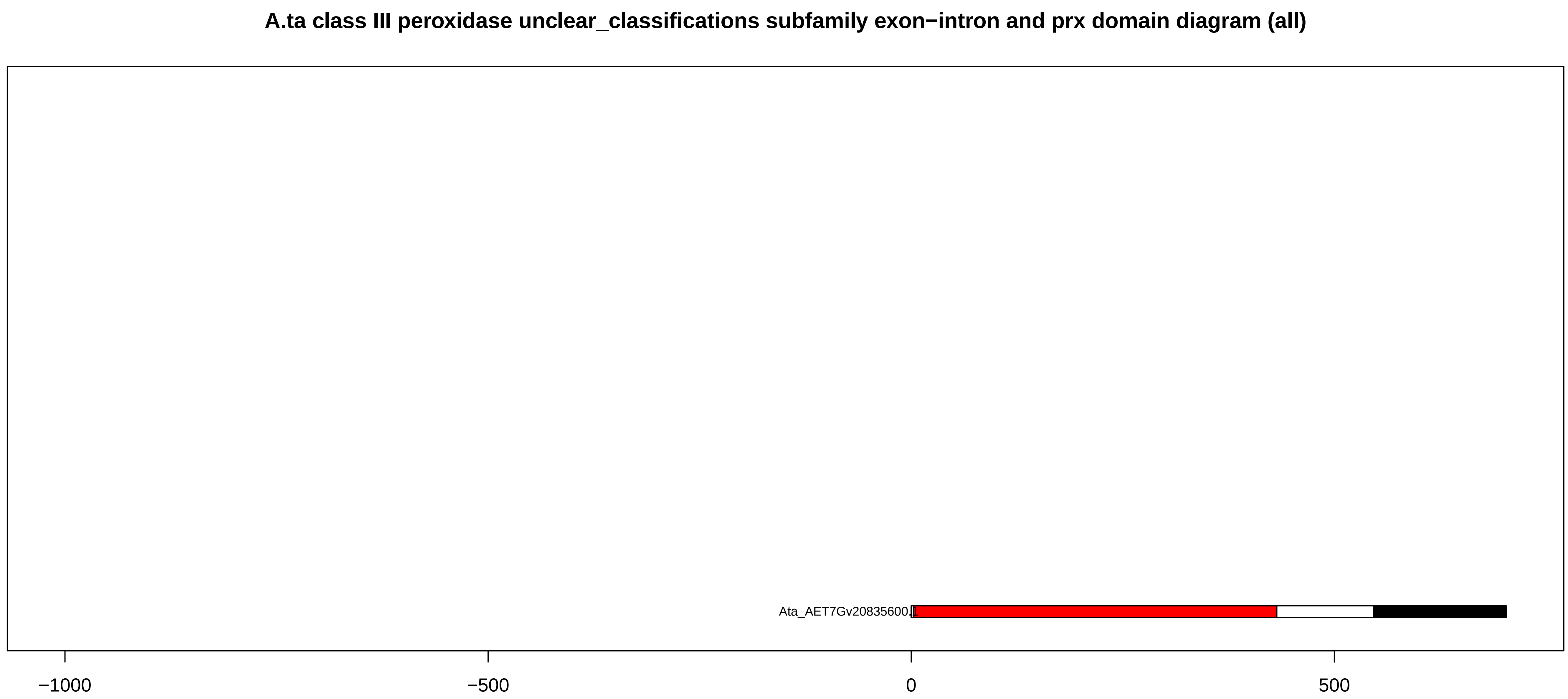
A.ta class III peroxidase unclear_classifications subfamily exon−intron and prx domain diagram (all)
Ata_AET7Gv20835600.1
−1000
−500
0
500

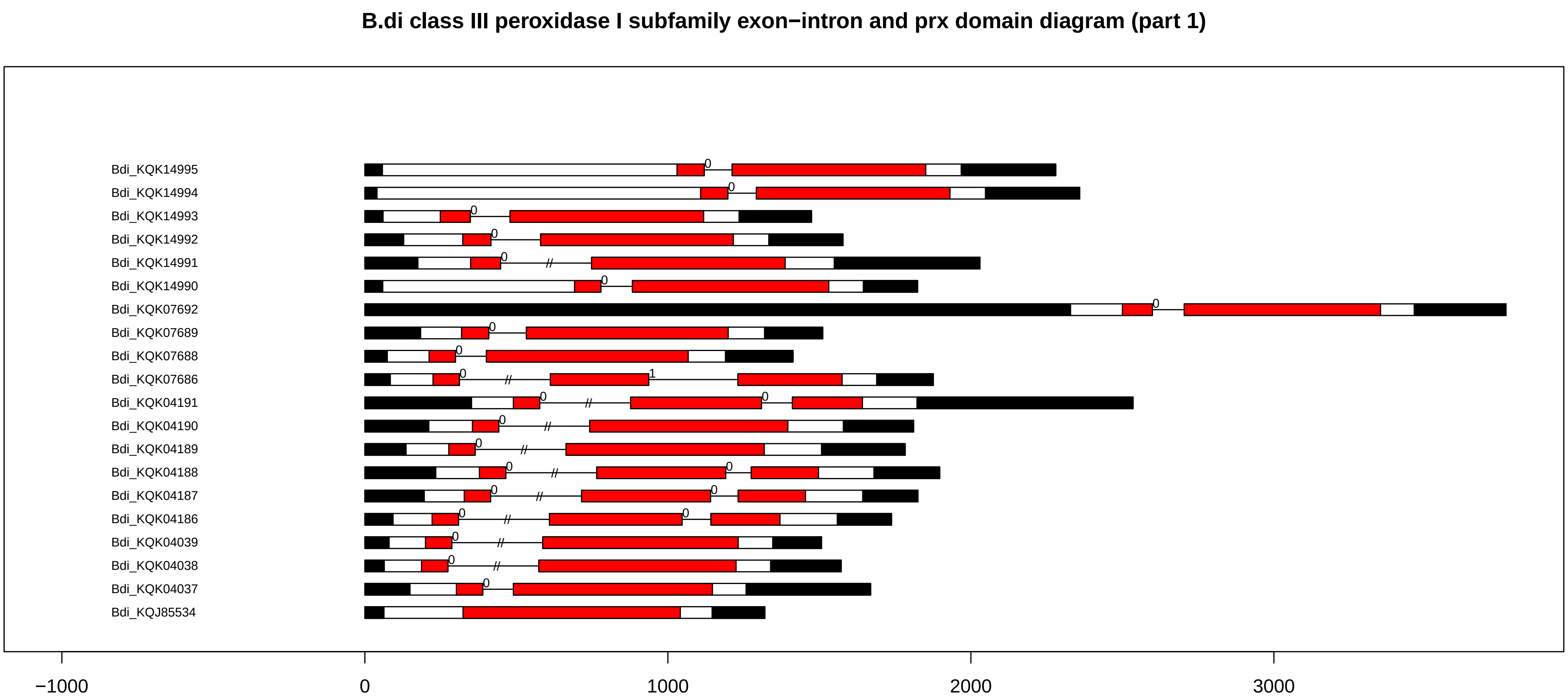
B.di class III peroxidase I subfamily exon−intron and prx domain diagram (part 1)
Bdi_KQK14995
Bdi_KQK14994
Bdi_KQK14993
Bdi_KQK14992
Bdi_KQK14991
Bdi_KQK14990
Bdi_KQK07692
Bdi_KQK07689
Bdi_KQK07688
Bdi_KQK07686
Bdi_KQK04191
Bdi_KQK04190
Bdi_KQK04189
Bdi_KQK04188
Bdi_KQK04187
Bdi_KQK04186
Bdi_KQK04039
Bdi_KQK04038
Bdi_KQK04037
Bdi_KQJ85534
−1000
0
1000
2000
3000

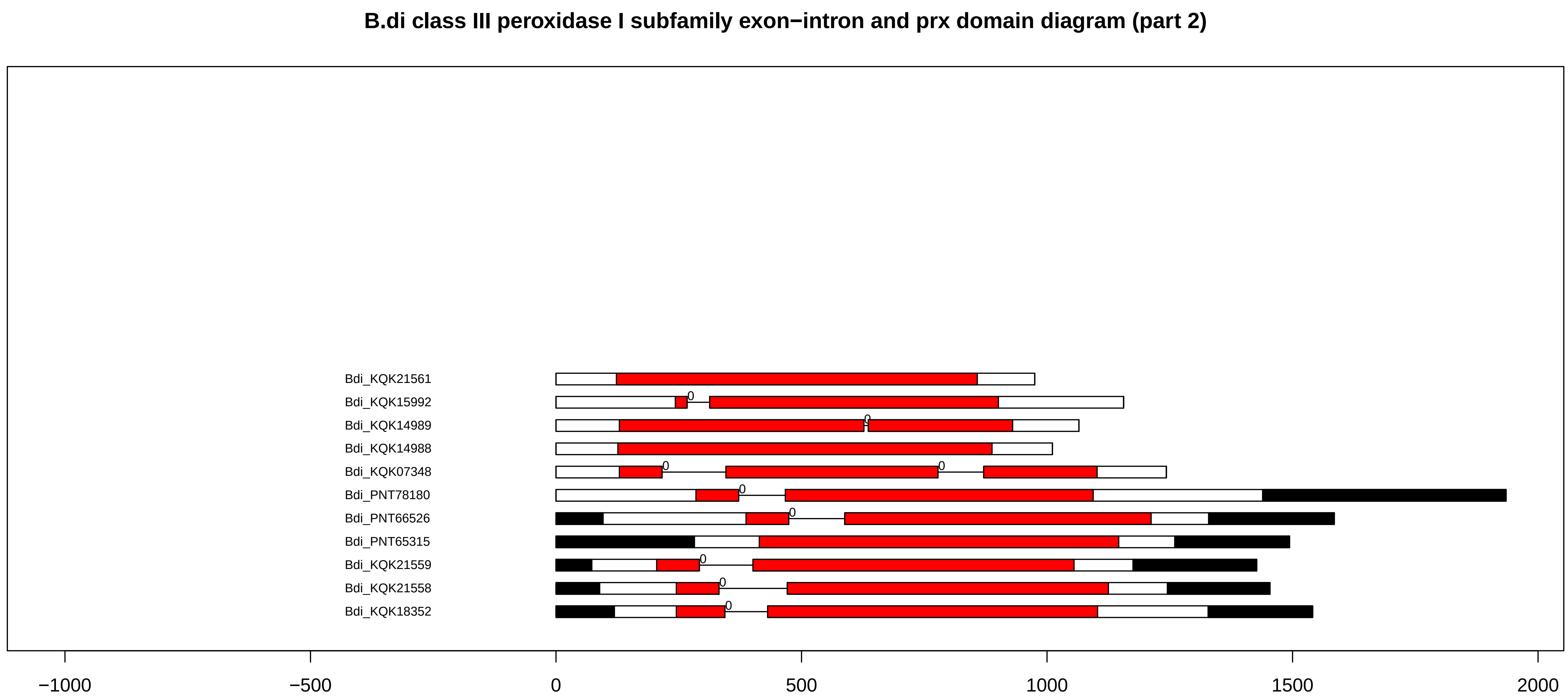

B.di class III peroxidase I subfamily exon−intron and prx domain diagram (part 2)
Bdi_KQK21561
Bdi_KQK15992
Bdi_KQK14989
Bdi_KQK14988
Bdi_KQK07348
Bdi_PNT78180
Bdi_PNT66526
Bdi_PNT65315
Bdi_KQK21559
Bdi_KQK21558
Bdi_KQK18352
−1000
−500
0
500
1000
1500
2000

B.di class III peroxidase II subfamily exon−intron and prx domain diagram (all)

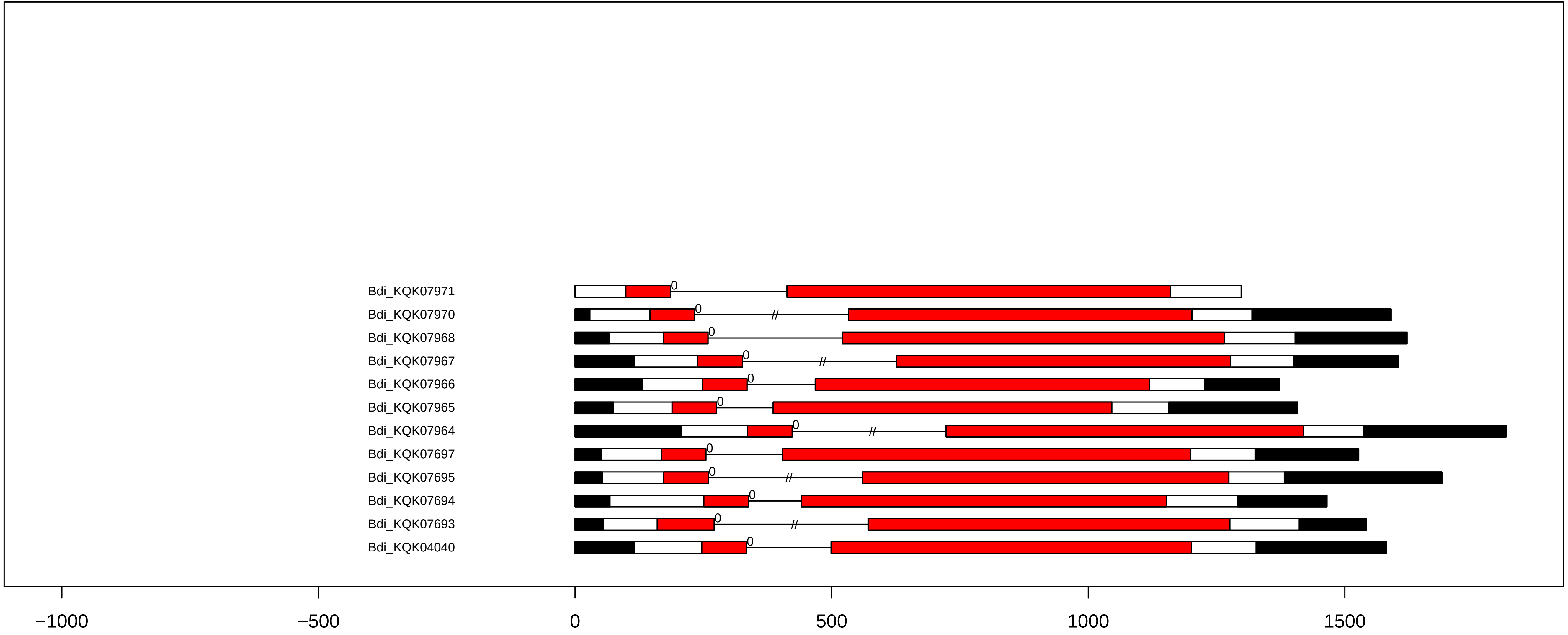

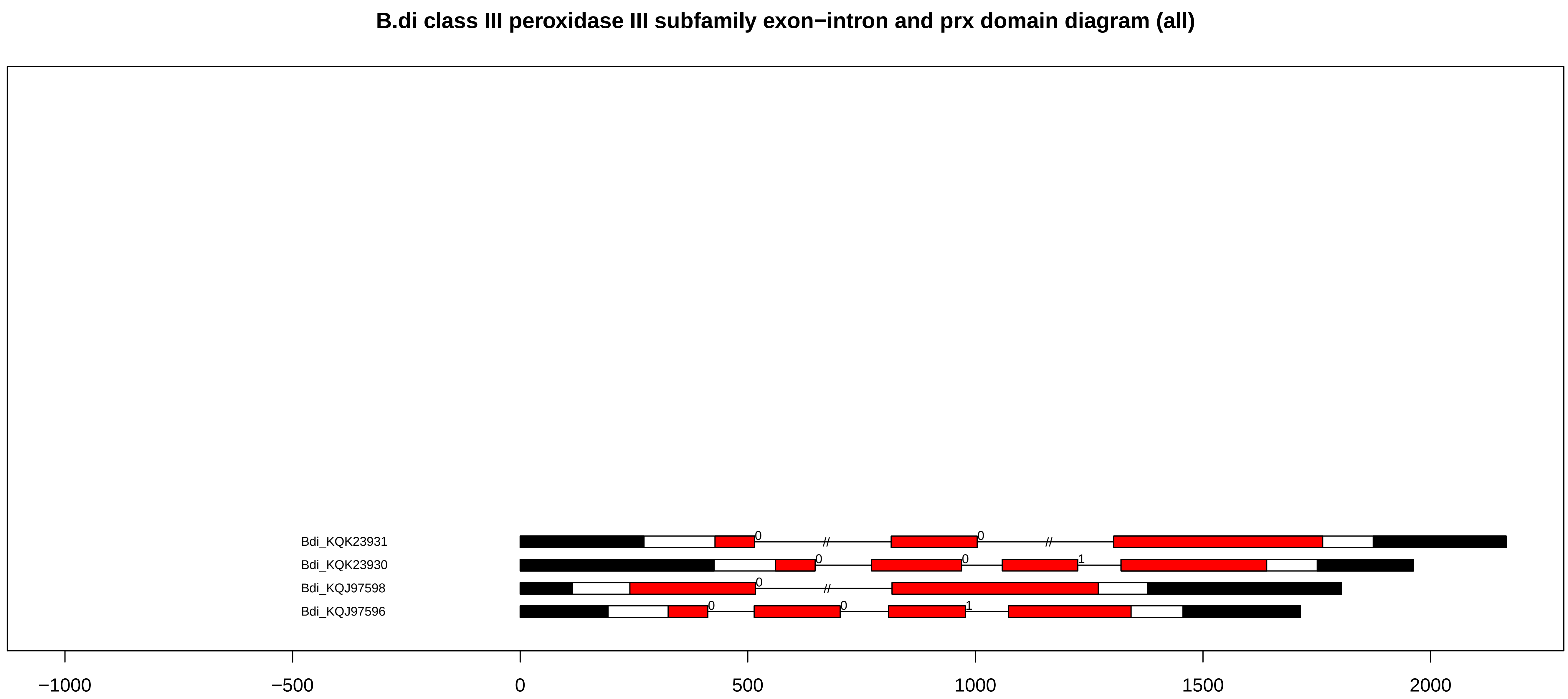

B.di class III peroxidase III subfamily exon−intron and prx domain diagram (all)
Bdi_KQK23931
Bdi_KQK23930
Bdi_KQJ97598
Bdi_KQJ97596
0
0
0
0
1
0
0
0
1
−1000
−500
0
500
1000
1500
2000

B.di class III peroxidase V subfamily exon−intron and prx domain diagram (part 1)

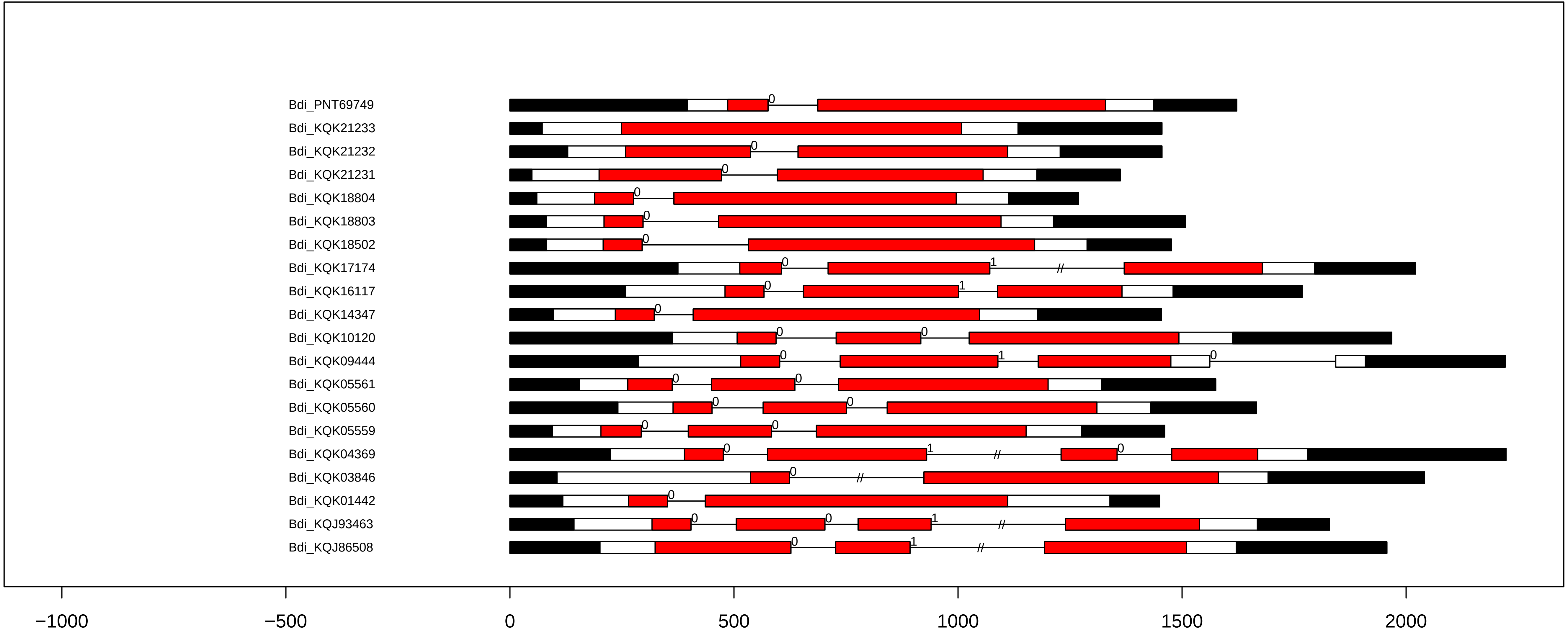

B.di class III peroxidase V subfamily exon−intron and prx domain diagram (part 2)

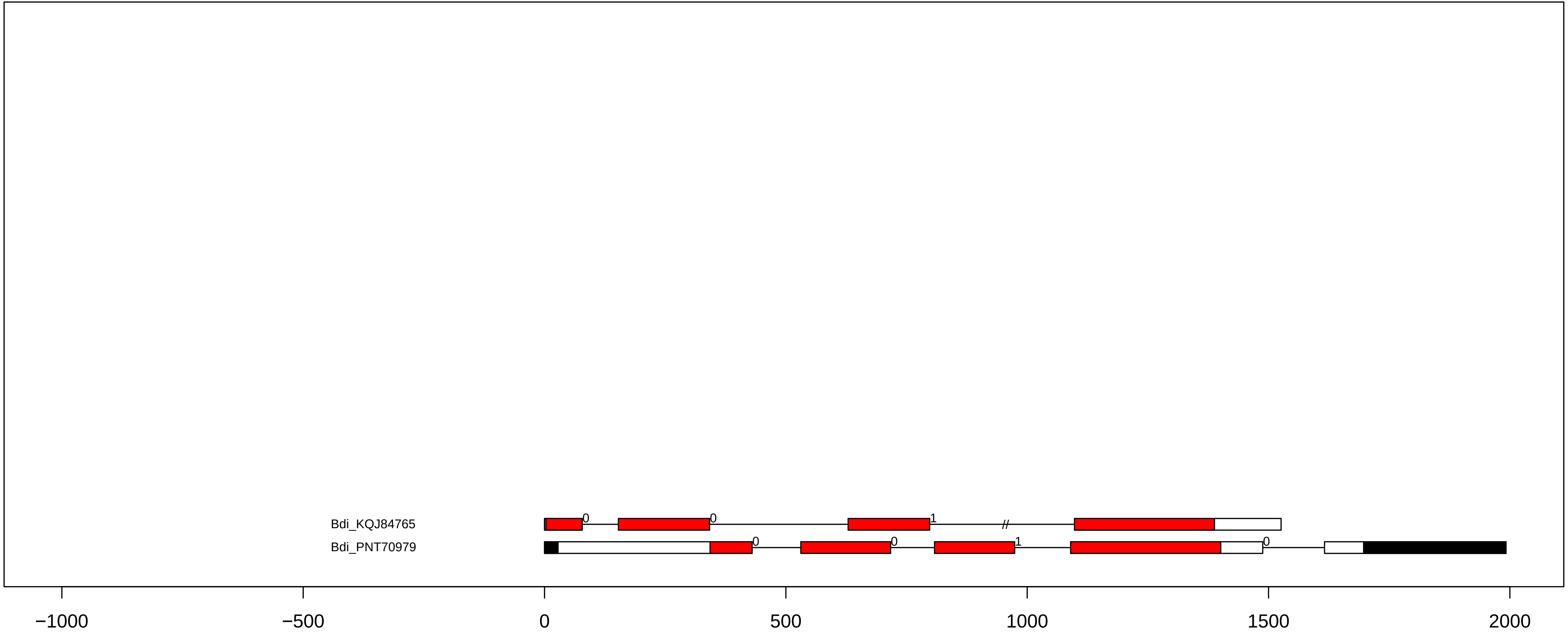

B.di class III peroxidase VI subfamily exon−intron and prx domain diagram (all)

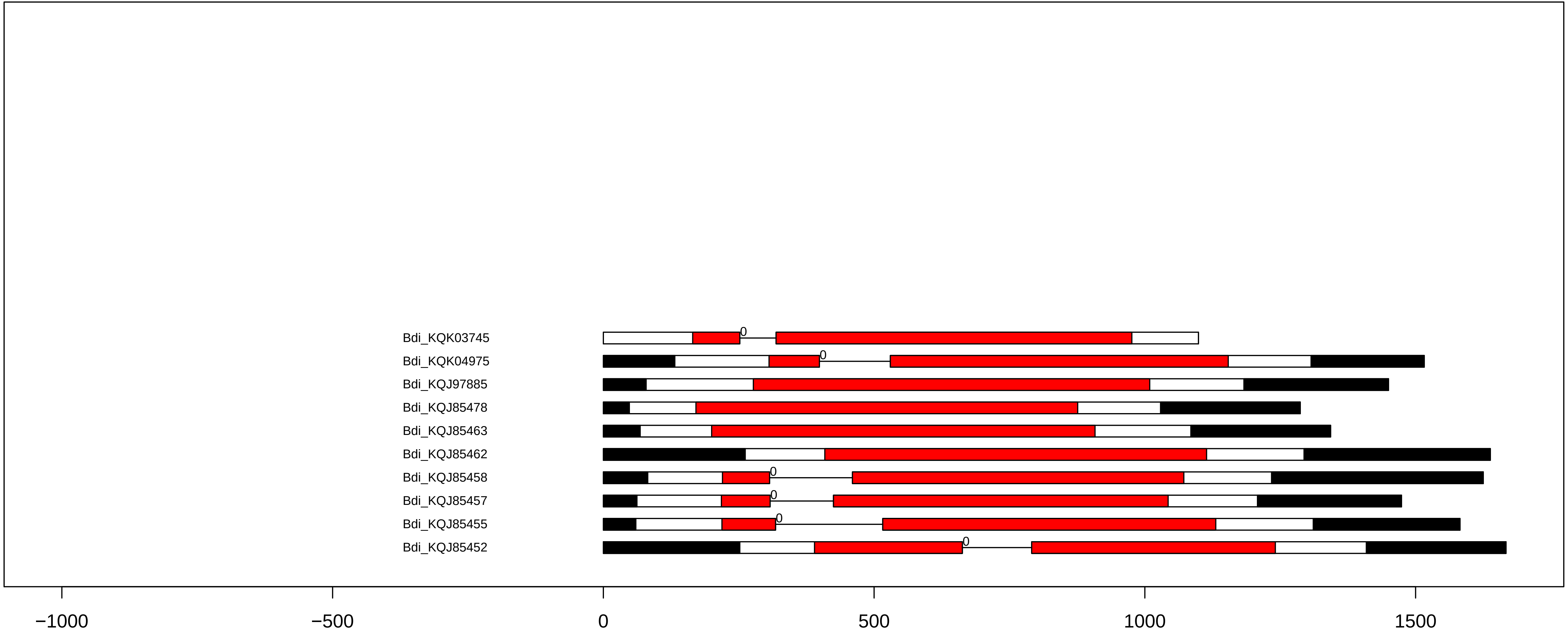

B.di class III peroxidase VII subfamily exon−intron and prx domain diagram (all)

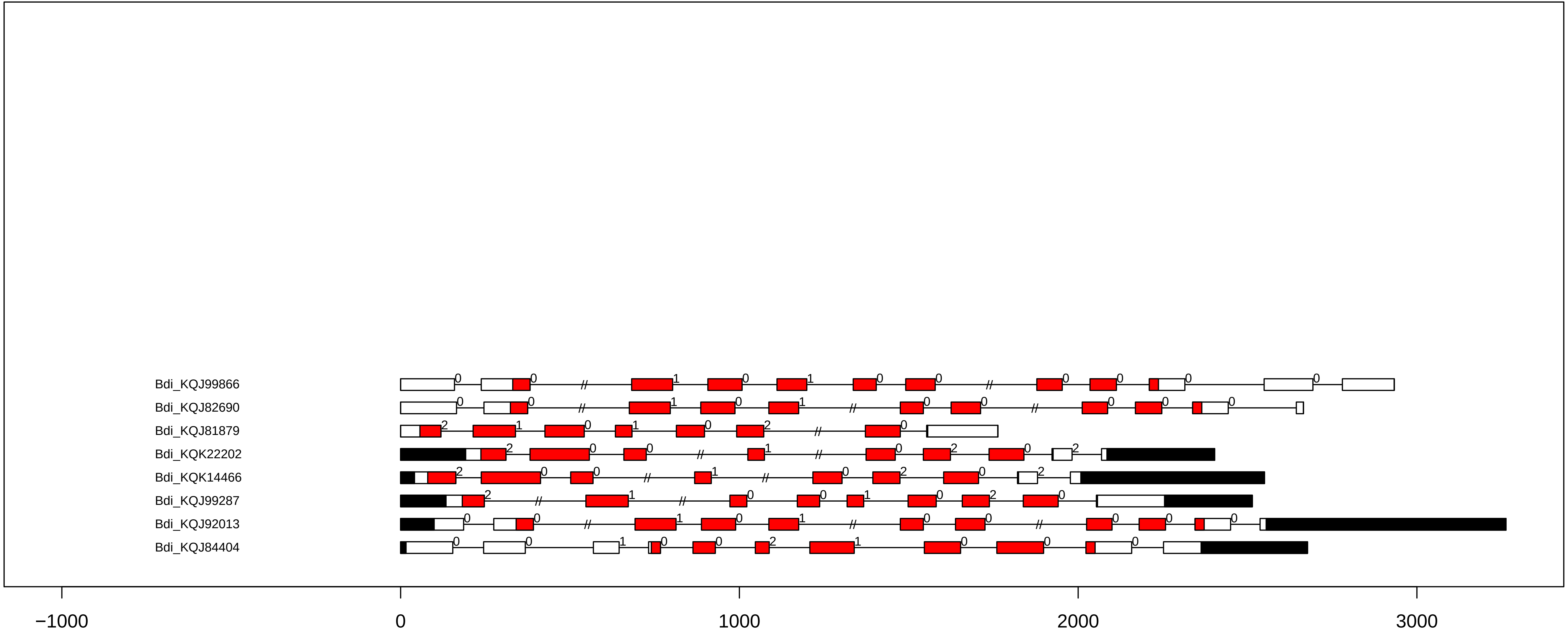

B.di class III peroxidase IX subfamily exon−intron and prx domain diagram (all)

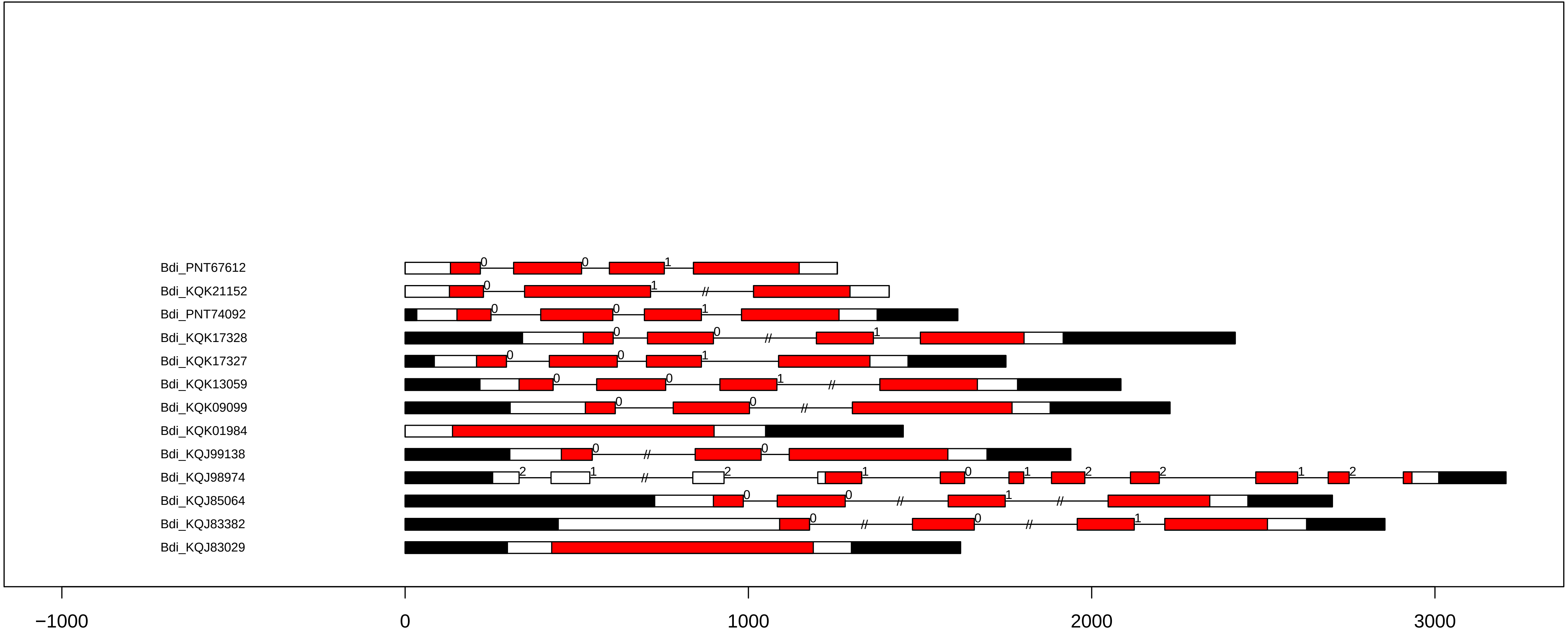

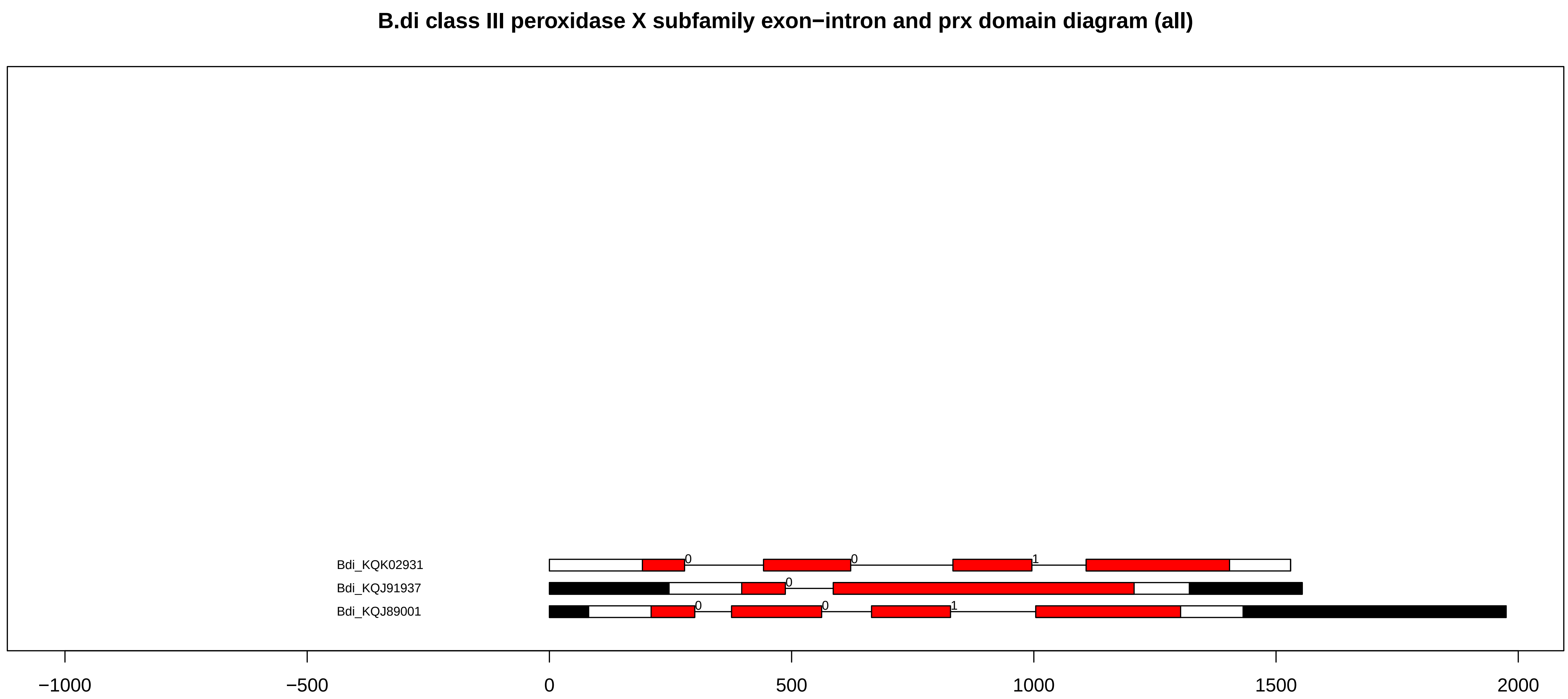

B.di class III peroxidase X subfamily exon−intron and prx domain diagram (all)
Bdi_KQK02931
Bdi_KQJ91937
Bdi_KQJ89001
0
0
1
0
0
0
1
−1000
−500
0
500
1000
1500
2000

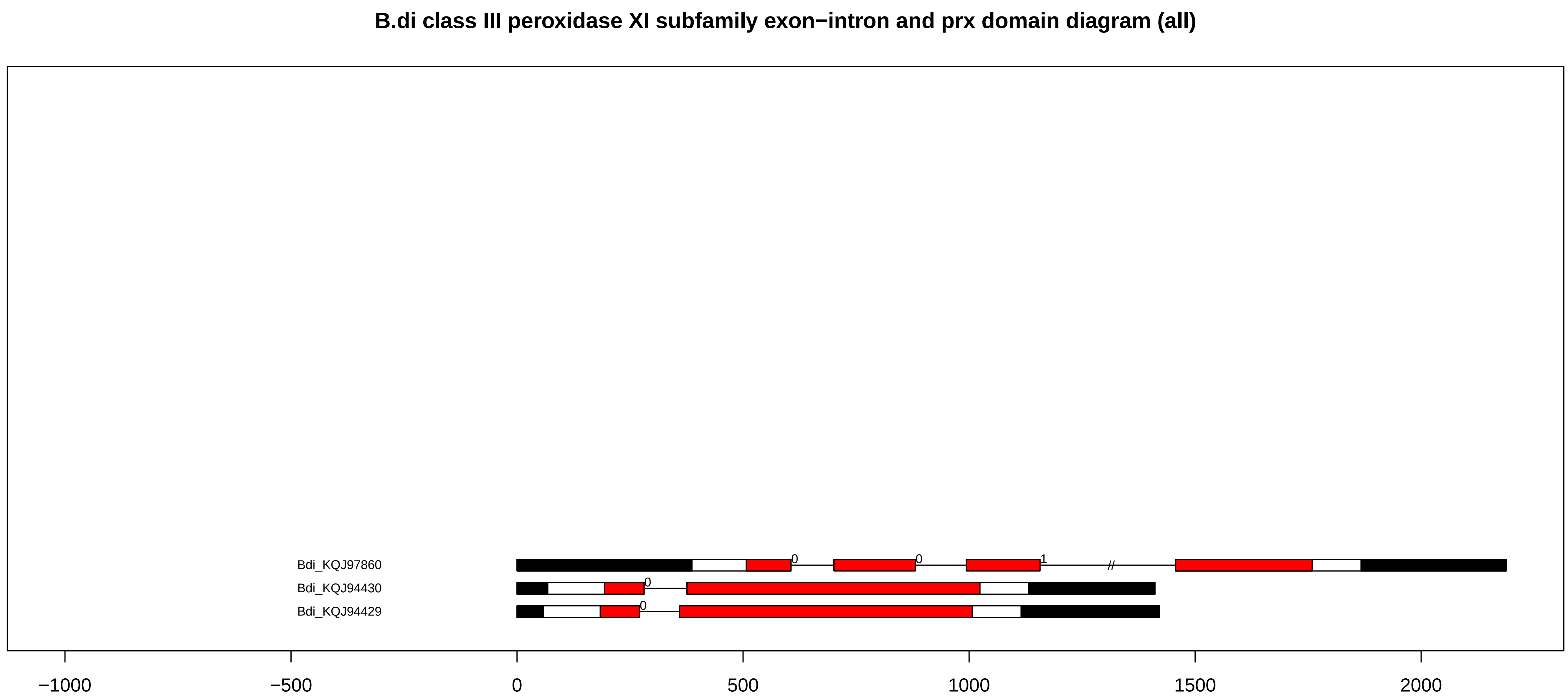

B.di class III peroxidase XI subfamily exon−intron and prx domain diagram (all)
Bdi_KQJ97860
Bdi_KQJ94430
Bdi_KQJ94429
0
0
1
//
0
0
−1000
−500
0
500
1000
1500
2000

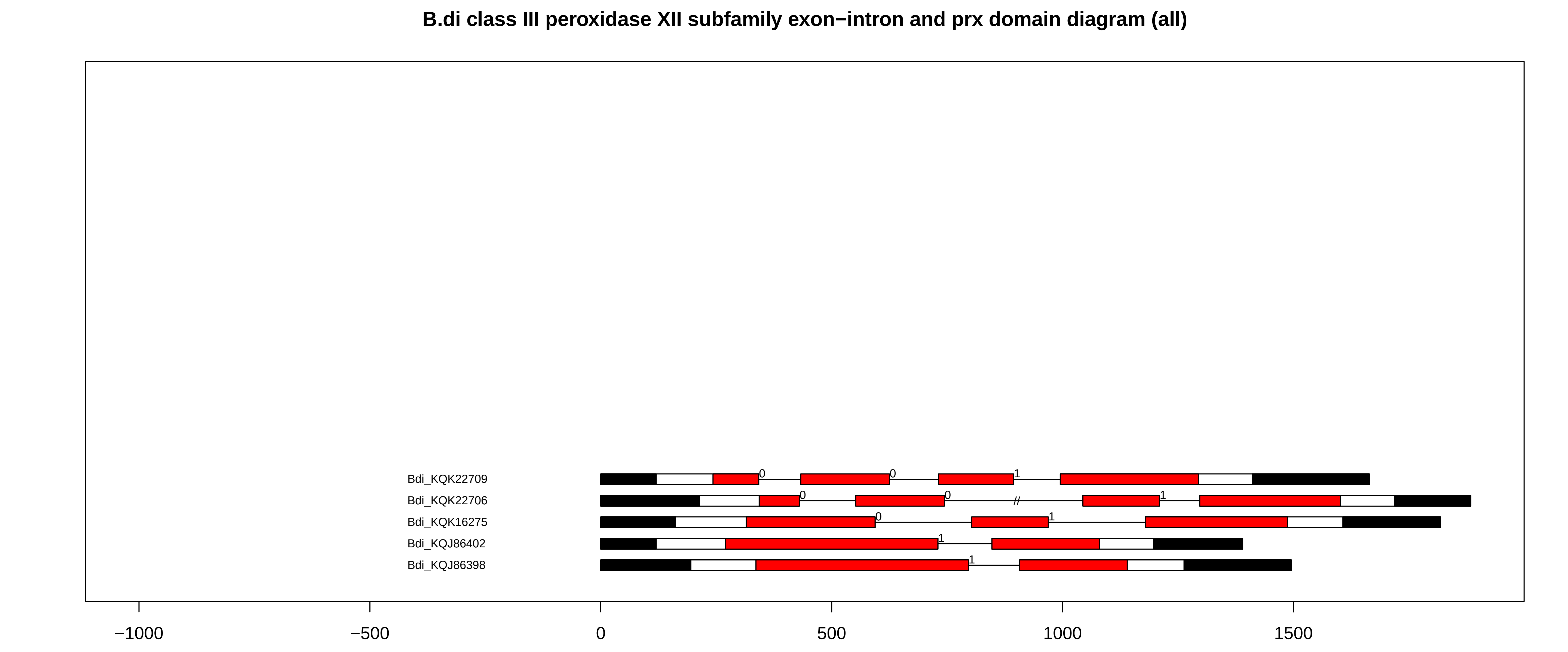
B.di class III peroxidase XII subfamily exon−intron and prx domain diagram (all)
Bdi_KQK22709
Bdi_KQK22706
Bdi_KQK16275
Bdi_KQJ86402
Bdi_KQJ86398
0
0
1
0
0
//
1
0
1
1
1
−1000
−500
0
500
1000
1500

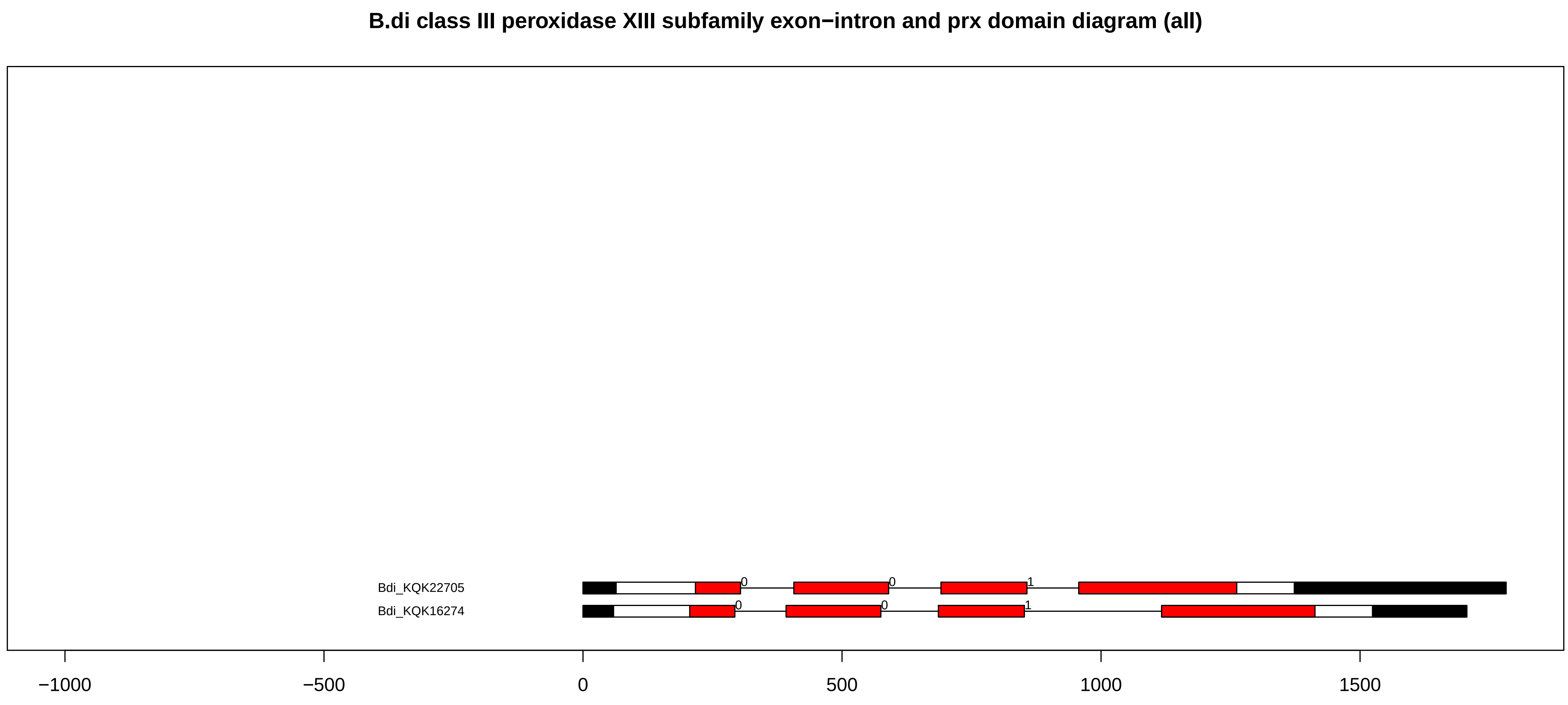

B.di class III peroxidase XIII subfamily exon−intron and prx domain diagram (all)
Bdi_KQK22705
Bdi_KQK16274
0
0
1
0
0
1
−1000
−500
0
500
1000
1500

B.di class III peroxidase XIV subfamily exon−intron and prx domain diagram (all)

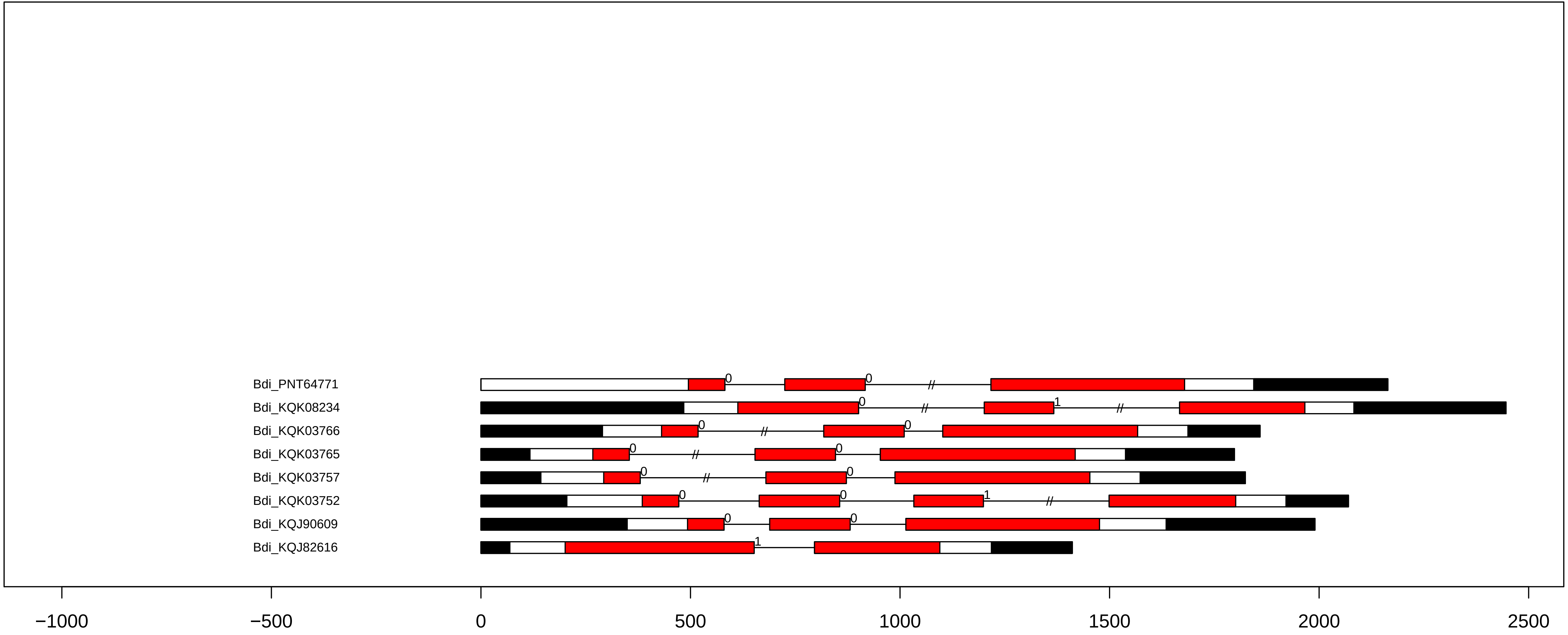

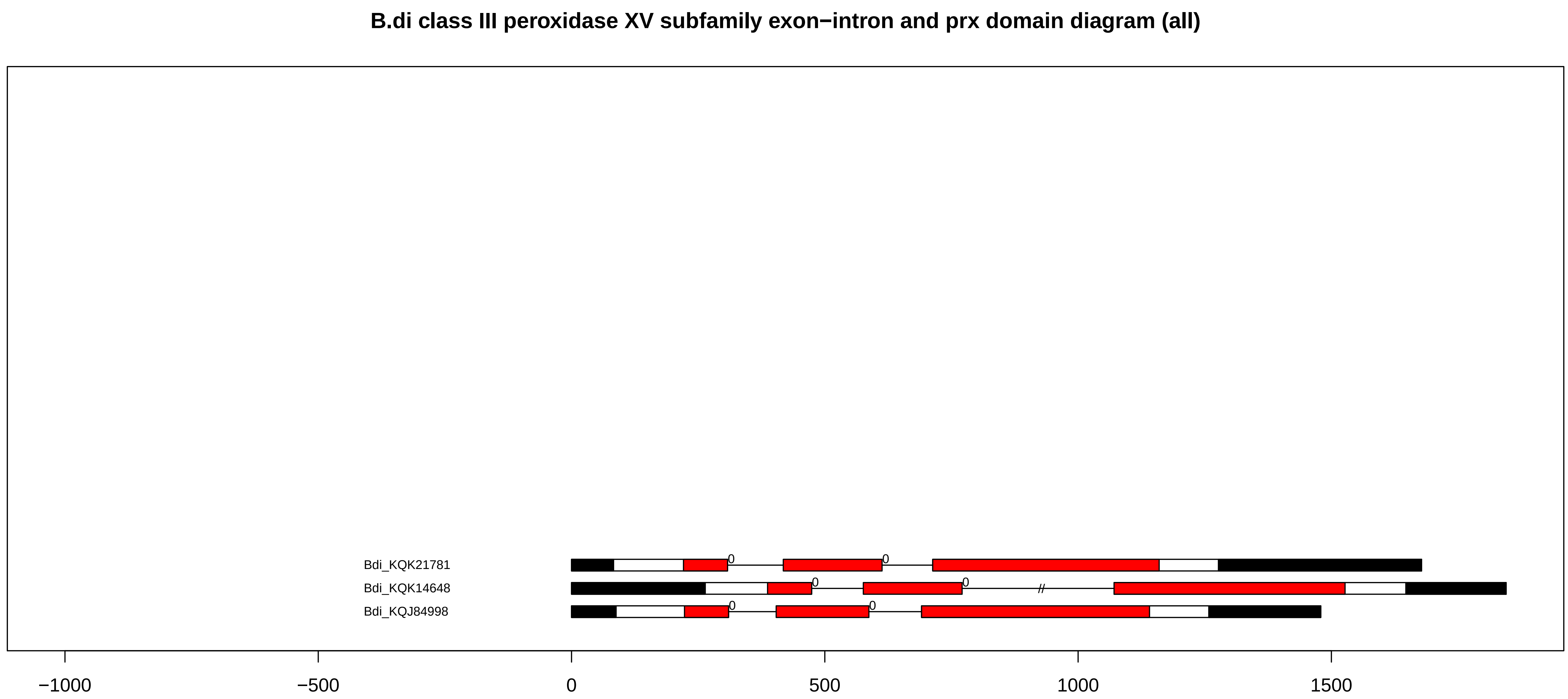
B.di class III peroxidase XV subfamily exon−intron and prx domain diagram (all)
Bdi_KQK21781
Bdi_KQK14648
Bdi_KQJ84998
0
0
0
0
0
0
//
−1000
−500
0
500
1000
1500

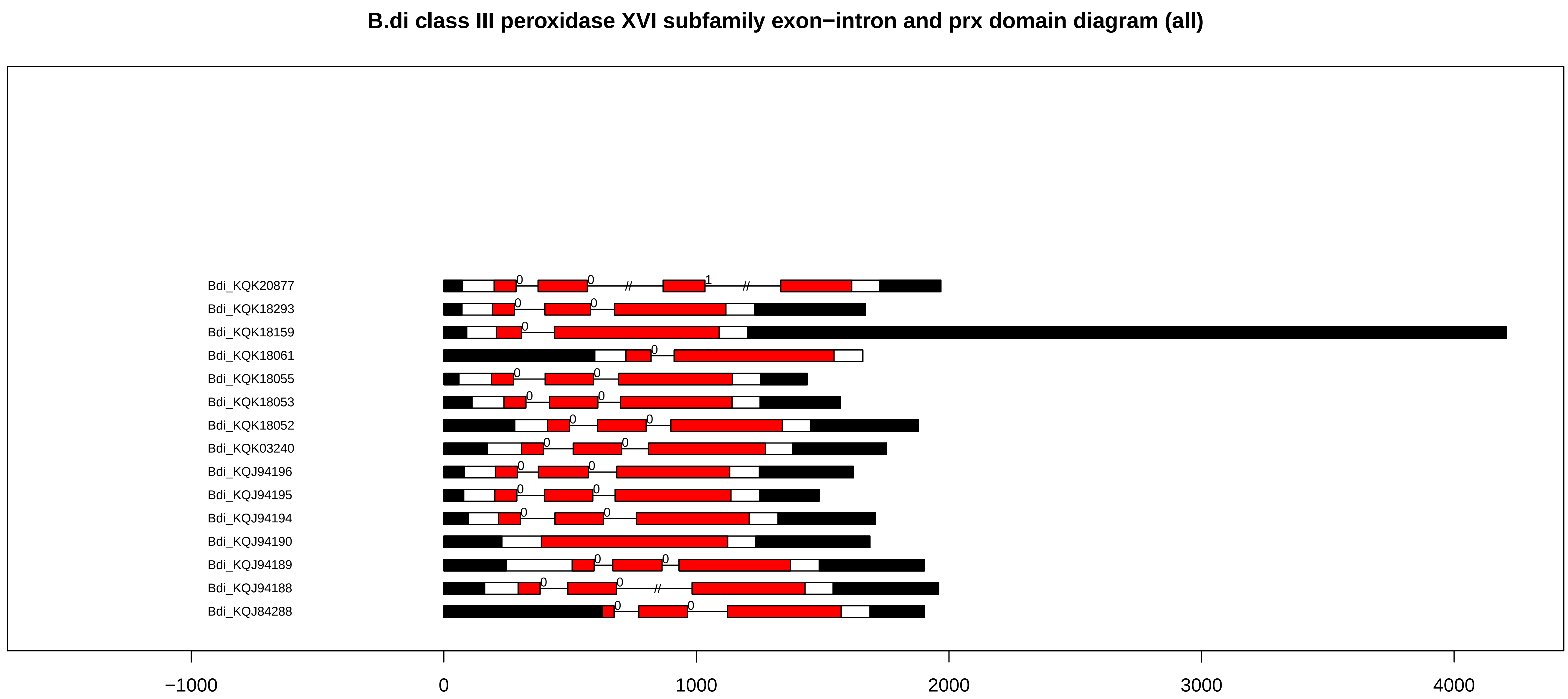

B.di class III peroxidase XVI subfamily exon−intron and prx domain diagram (all)
Bdi_KQK20877
Bdi_KQK18293
Bdi_KQK18159
Bdi_KQK18061
Bdi_KQK18055
Bdi_KQK18053
Bdi_KQK18052
Bdi_KQK03240
Bdi_KQJ94196
Bdi_KQJ94195
Bdi_KQJ94194
Bdi_KQJ94190
Bdi_KQJ94189
Bdi_KQJ94188
Bdi_KQJ84288
−1000
0
1000
2000
3000
4000

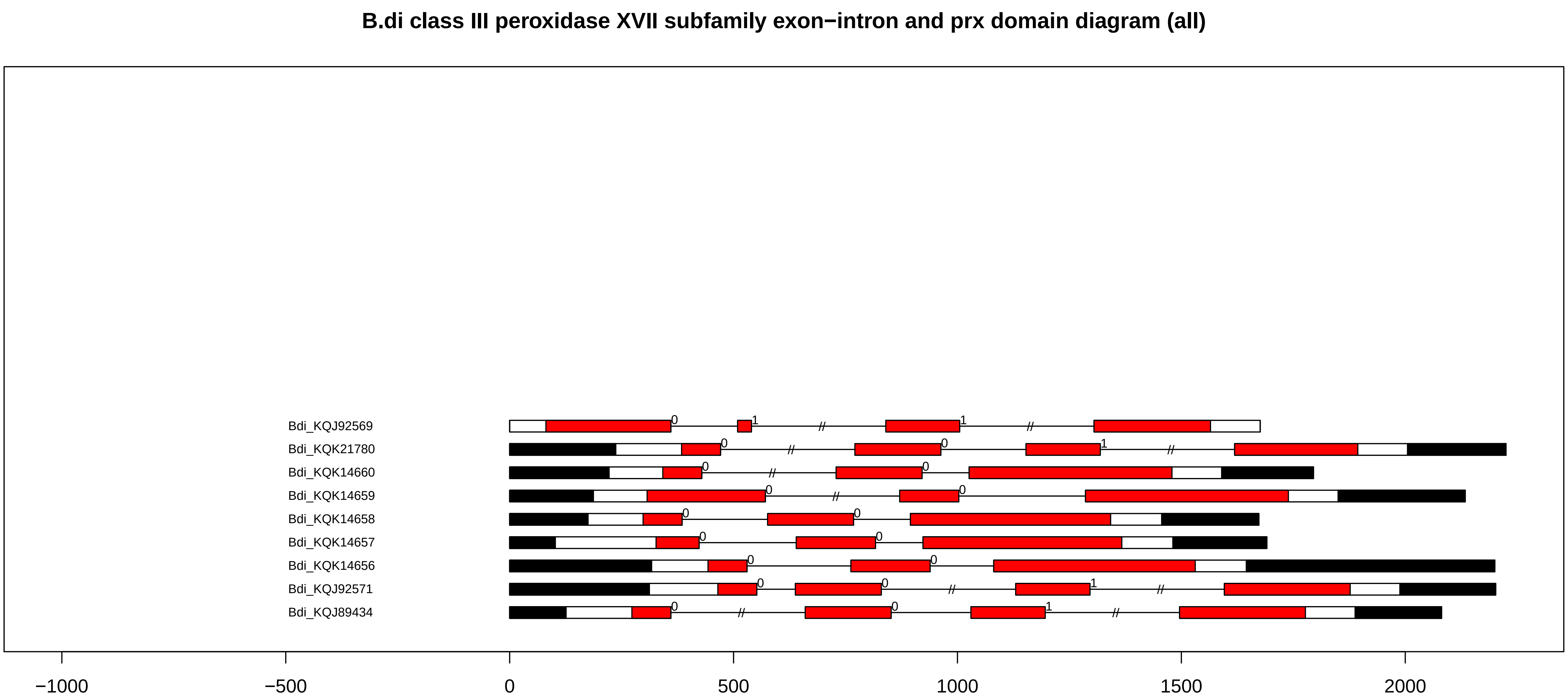
B.di class III peroxidase XVII subfamily exon−intron and prx domain diagram (all)
Bdi_KQJ92569
Bdi_KQK21780
Bdi_KQK14660
Bdi_KQK14659
Bdi_KQK14658
Bdi_KQK14657
Bdi_KQK14656
Bdi_KQJ92571
Bdi_KQJ89434
−1000
−500
0
500
1000
1500
2000

B.di class III peroxidase XVIII subfamily exon−intron and prx domain diagram (all)

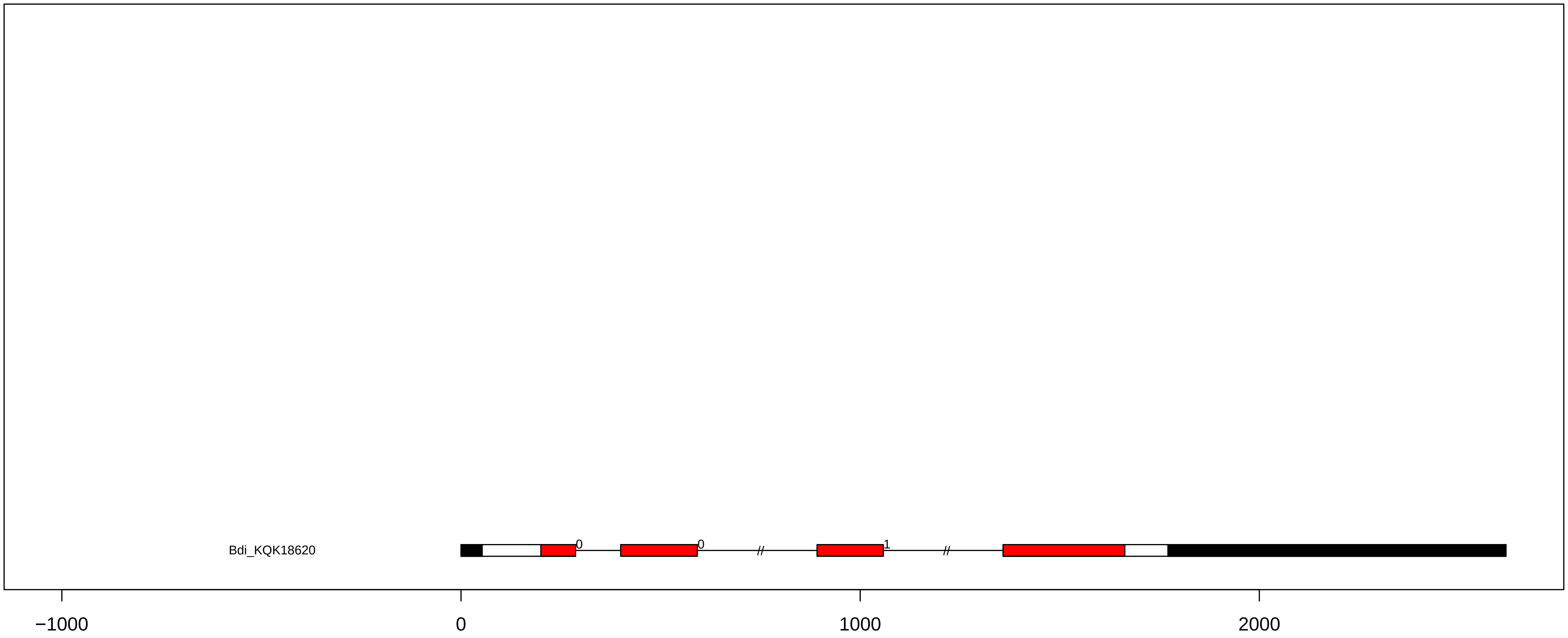

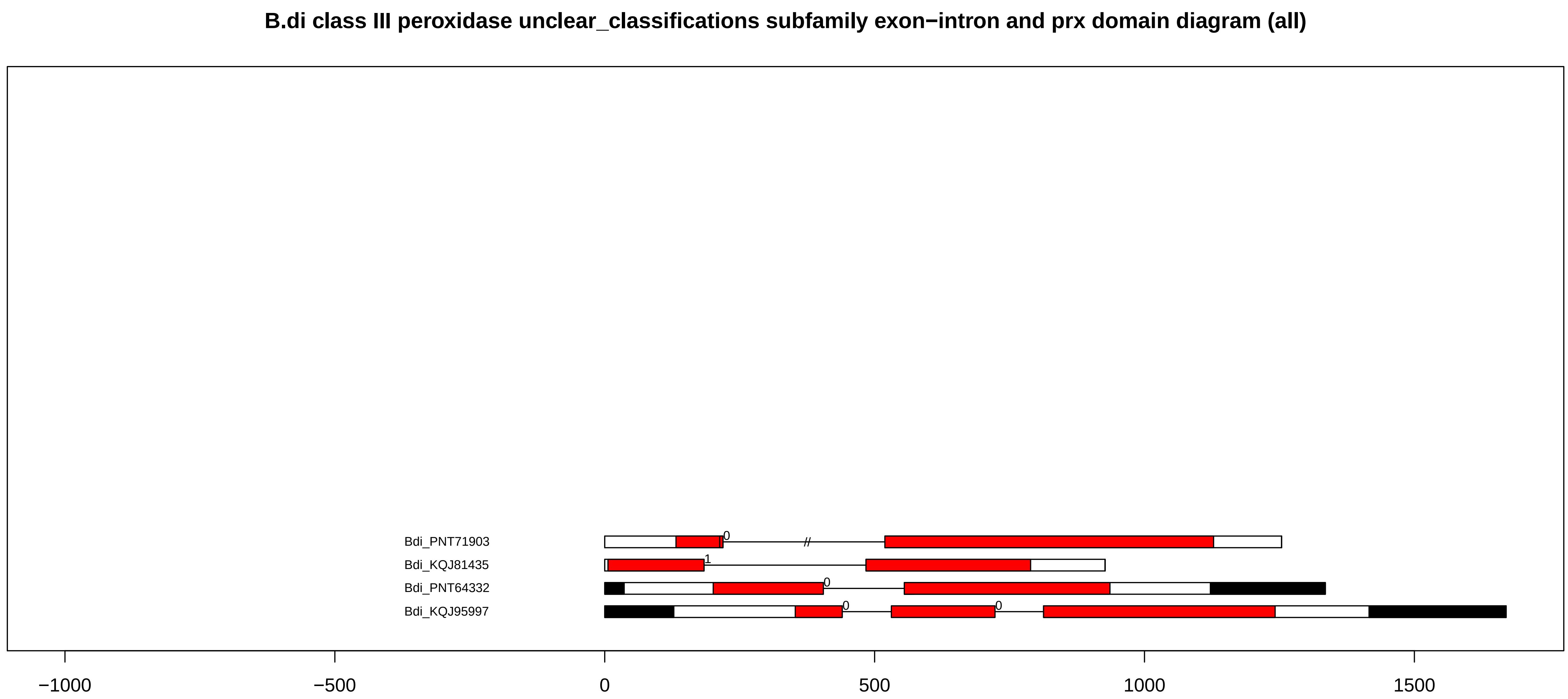
B.di class III peroxidase unclear_classifications subfamily exon−intron and prx domain diagram (all)
Bdi_PNT71903
Bdi_KQJ81435
Bdi_PNT64332
Bdi_KQJ95997
0
1
0
0
0
//
−1000
−500
0
500
1000
1500

O.sa class III peroxidase I subfamily exon−intron and prx domain diagram (part 1)

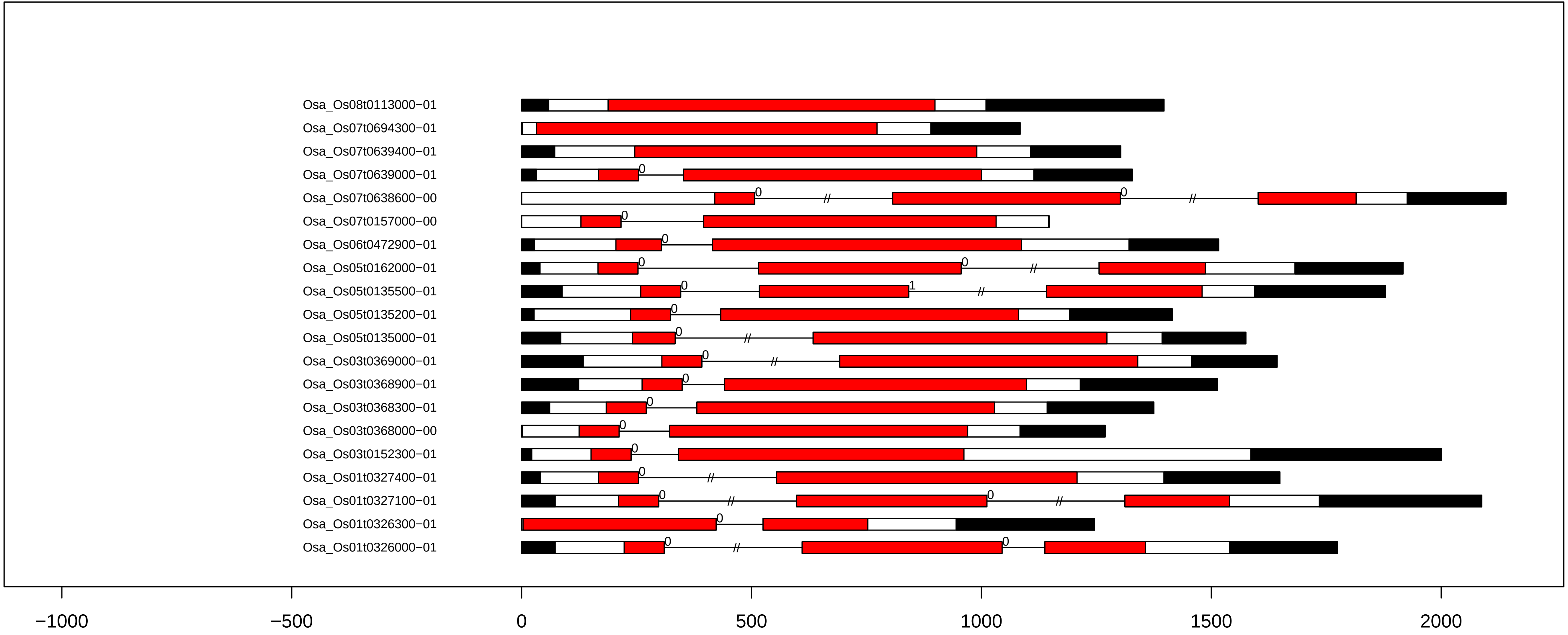

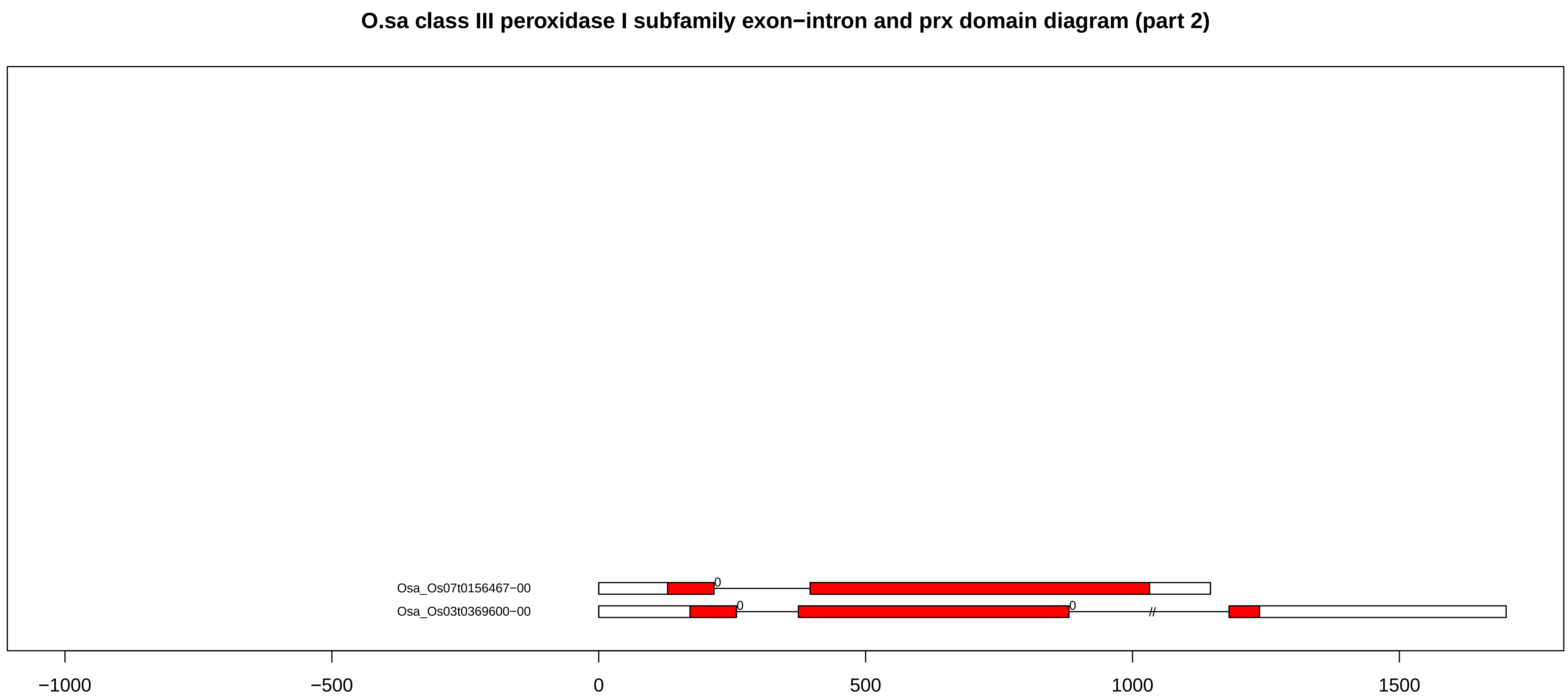

O.sa class III peroxidase I subfamily exon−intron and prx domain diagram (part 2)
Osa_Os07t0156467−00
Osa_Os03t0369600−00
0
0
0
//
−1000
−500
0
500
1000
1500

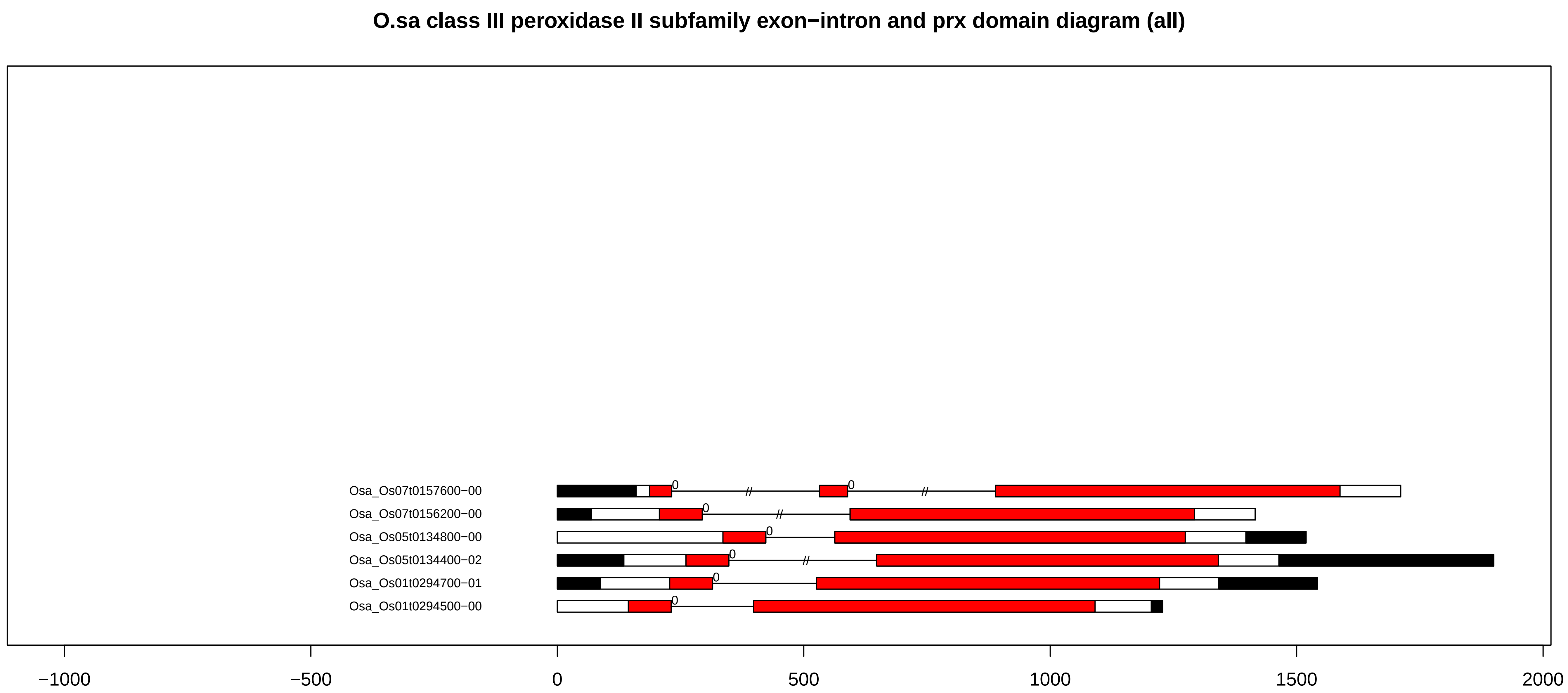

O.sa class III peroxidase II subfamily exon−intron and prx domain diagram (all)
Osa_Os07t0157600−00
Osa_Os07t0156200−00
Osa_Os05t0134800−00
Osa_Os05t0134400−02
Osa_Os01t0294700−01
Osa_Os01t0294500−00
−1000
−500
0
500
1000
1500
2000

O.sa class III peroxidase III subfamily exon−intron and prx domain diagram (all)

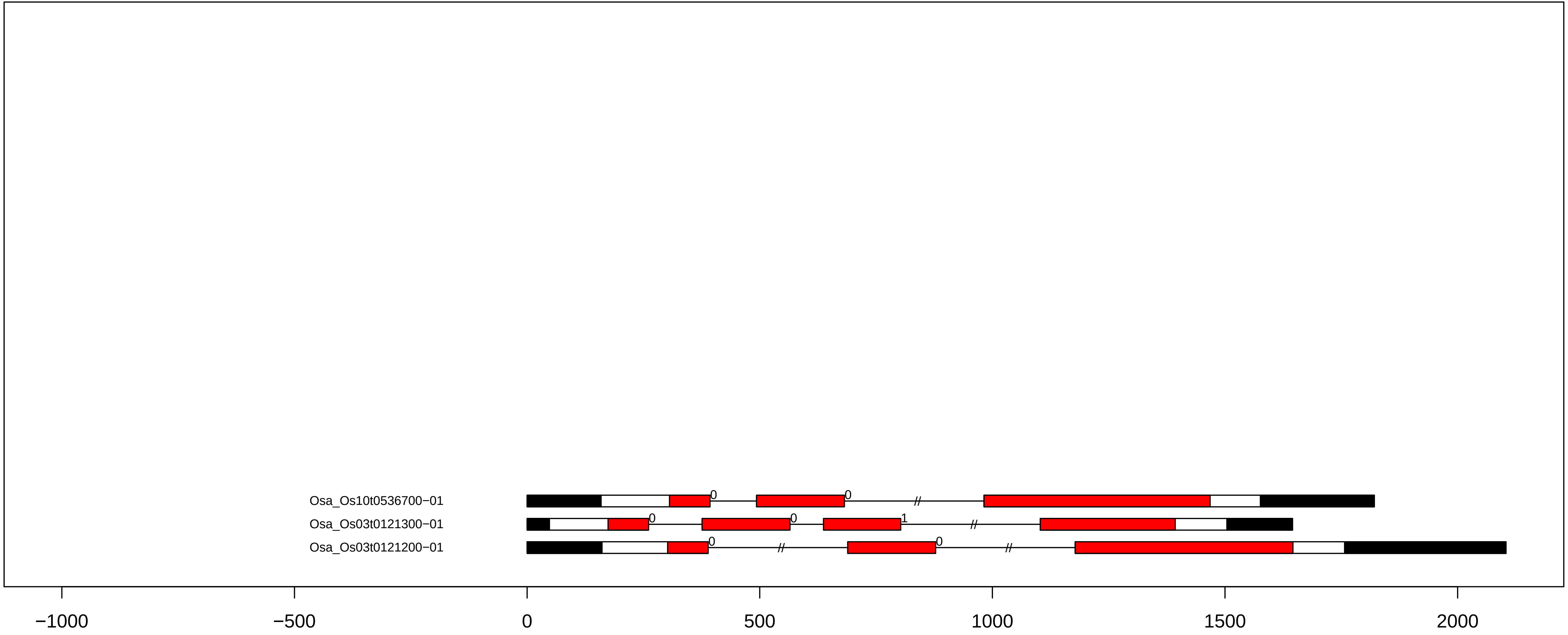

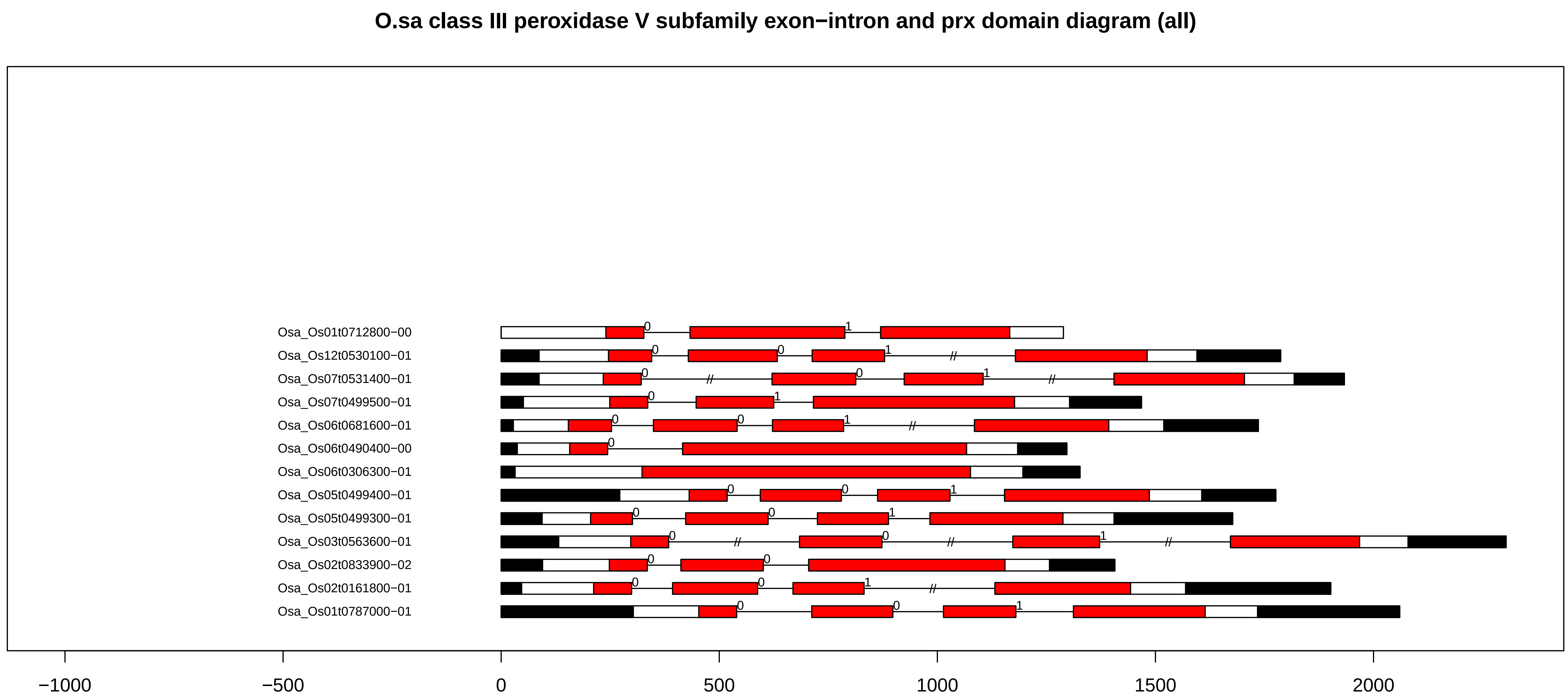
O.sa class III peroxidase V subfamily exon−intron and prx domain diagram (all)
Osa_Os01t0712800−00
Osa_Os12t0530100−01
Osa_Os07t0531400−01
Osa_Os07t0499500−01
Osa_Os06t0681600−01
Osa_Os06t0490400−00
Osa_Os06t0306300−01
Osa_Os05t0499400−01
Osa_Os05t0499300−01
Osa_Os03t0563600−01
Osa_Os02t0833900−02
Osa_Os02t0161800−01
Osa_Os01t0787000−01
−1000
−500
0
500
1000
1500
2000

O.sa class III peroxidase VI subfamily exon−intron and prx domain diagram (all)

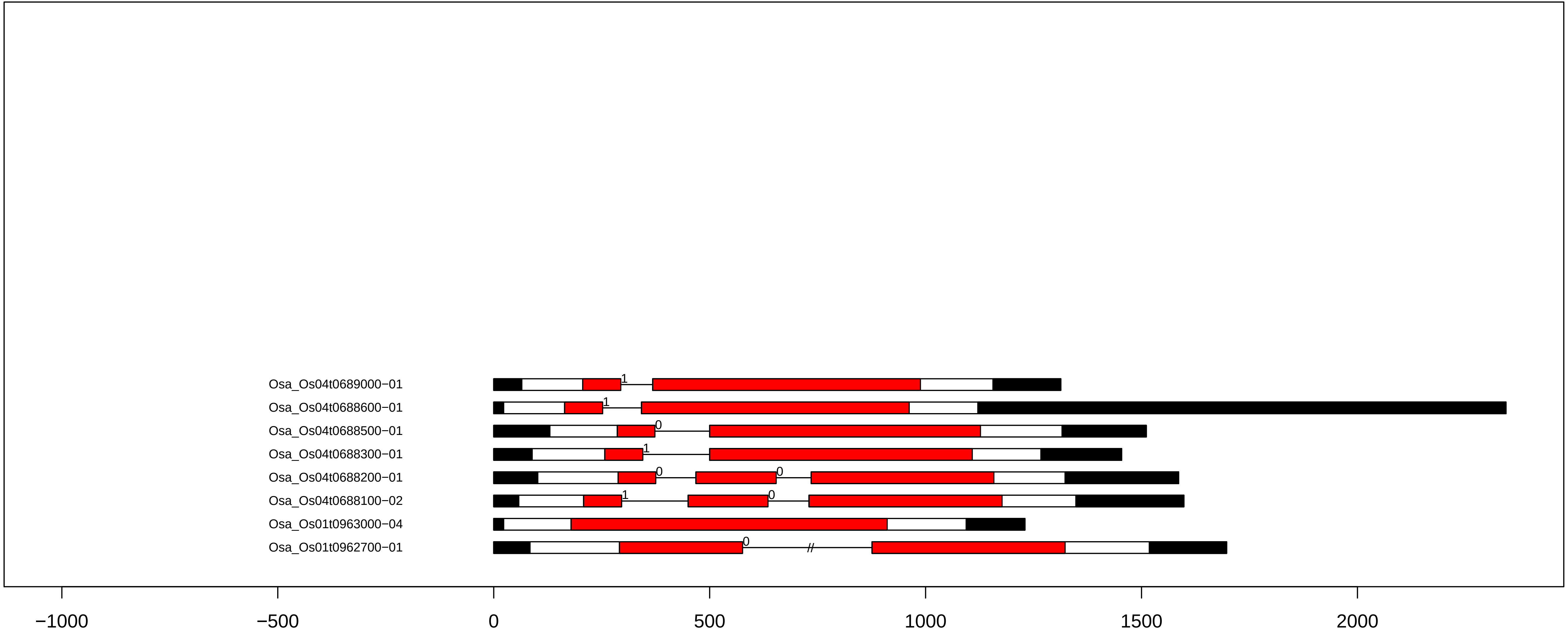

O.sa class III peroxidase VII subfamily exon−intron and prx domain diagram (all)

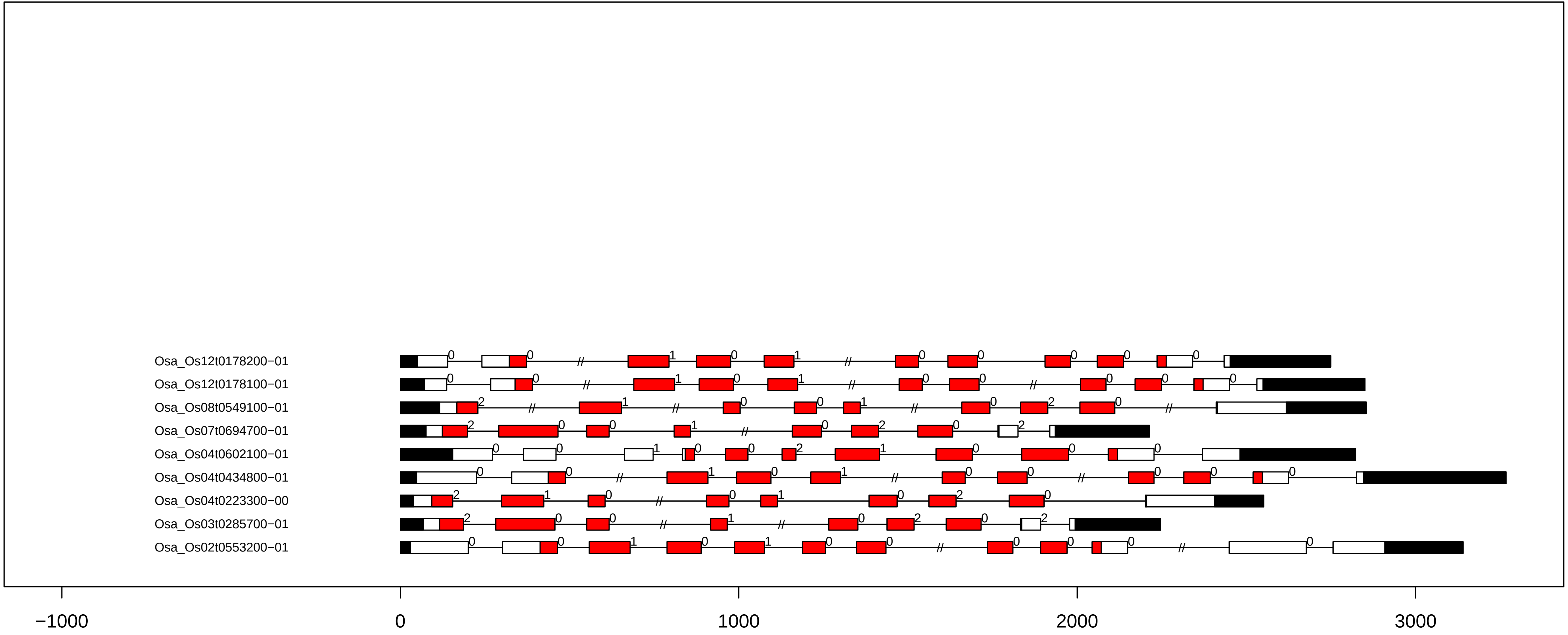

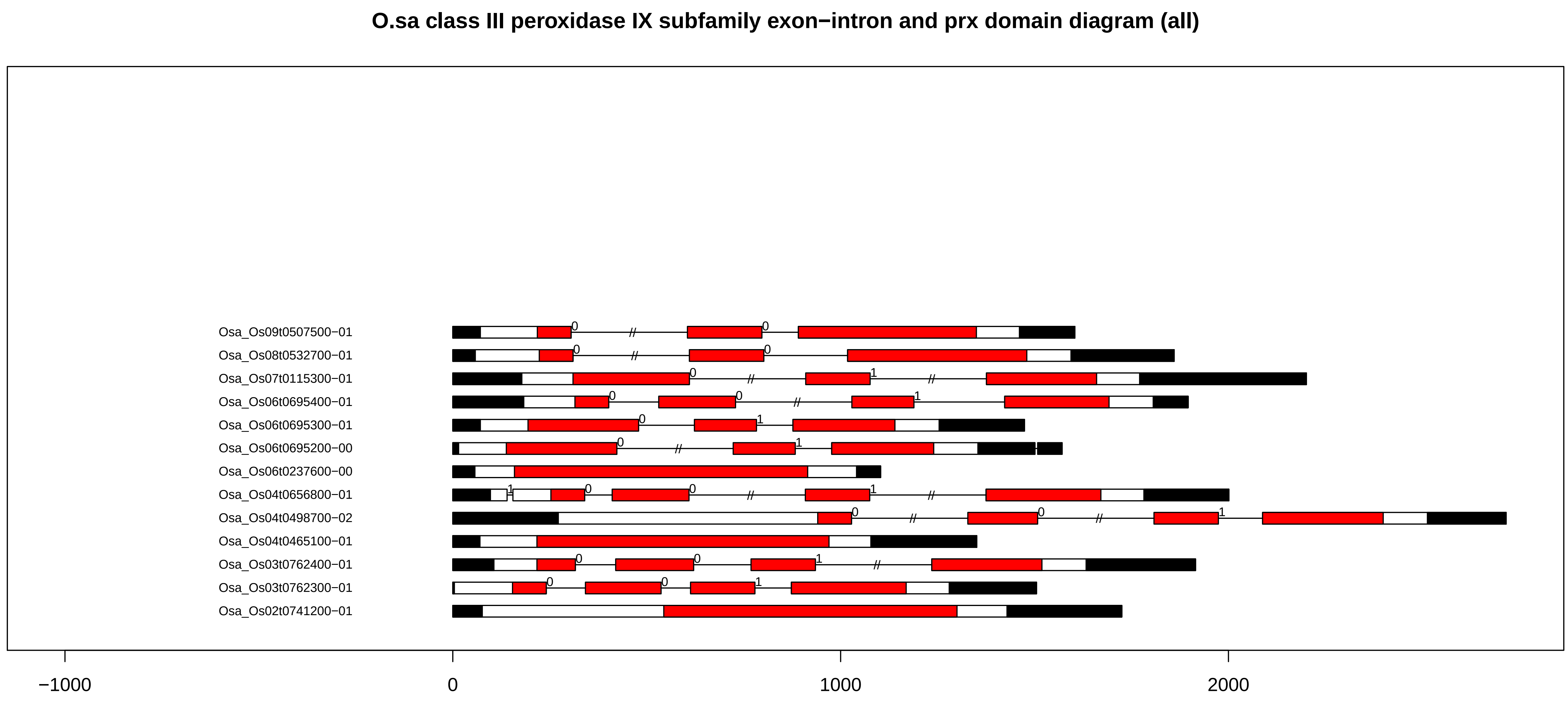

O.sa class III peroxidase IX subfamily exon−intron and prx domain diagram (all)
Osa_Os09t0507500−01
Osa_Os08t0532700−01
Osa_Os07t0115300−01
Osa_Os06t0695400−01
Osa_Os06t0695300−01
Osa_Os06t0695200−00
Osa_Os06t0237600−00
Osa_Os04t0656800−01
Osa_Os04t0498700−02
Osa_Os04t0465100−01
Osa_Os03t0762400−01
Osa_Os03t0762300−01
Osa_Os02t0741200−01
−1000
0
1000
2000

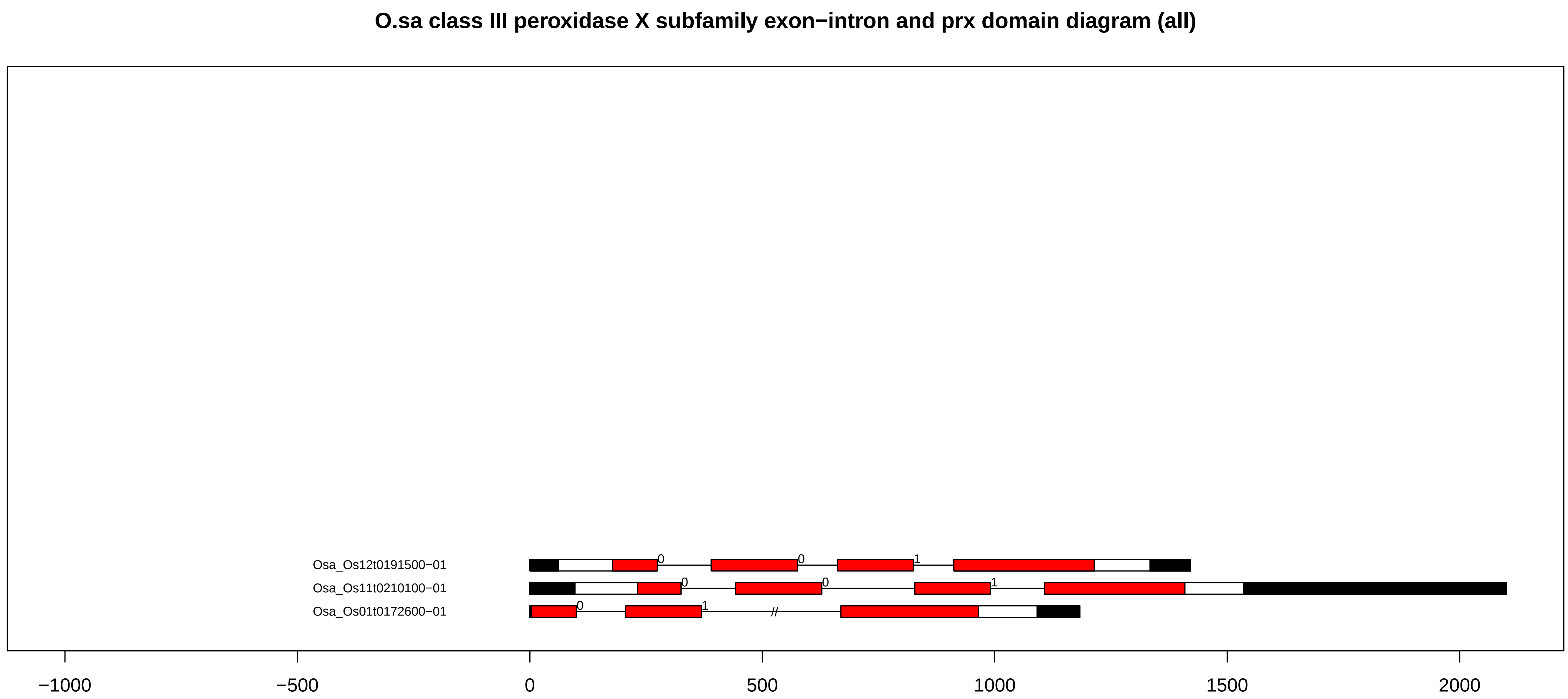
O.sa class III peroxidase X subfamily exon−intron and prx domain diagram (all)
Osa_Os12t0191500−01
Osa_Os11t0210100−01
Osa_Os01t0172600−01
0
0
1
0
0
1
0
1
//
−1000
−500
0
500
1000
1500
2000

O.sa class III peroxidase XI subfamily exon−intron and prx domain diagram (all)

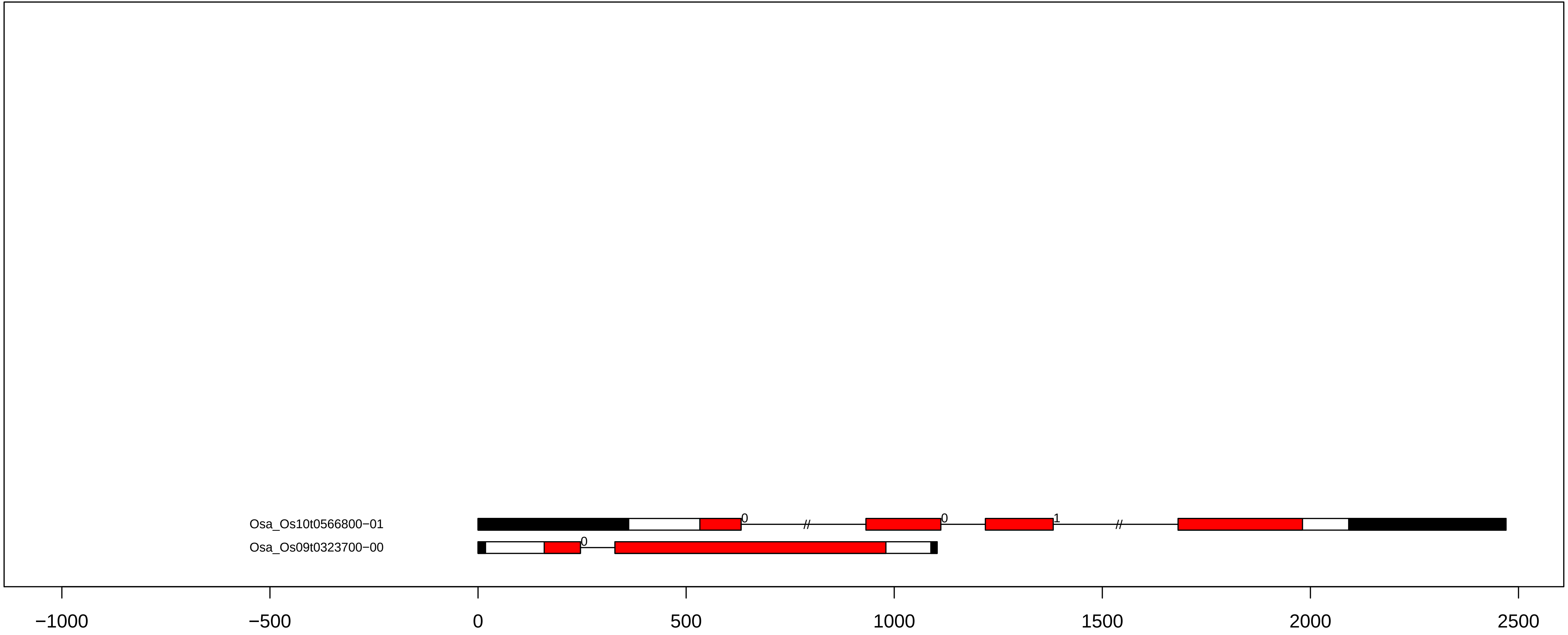

O.sa class III peroxidase XII subfamily exon−intron and prx domain diagram (all)

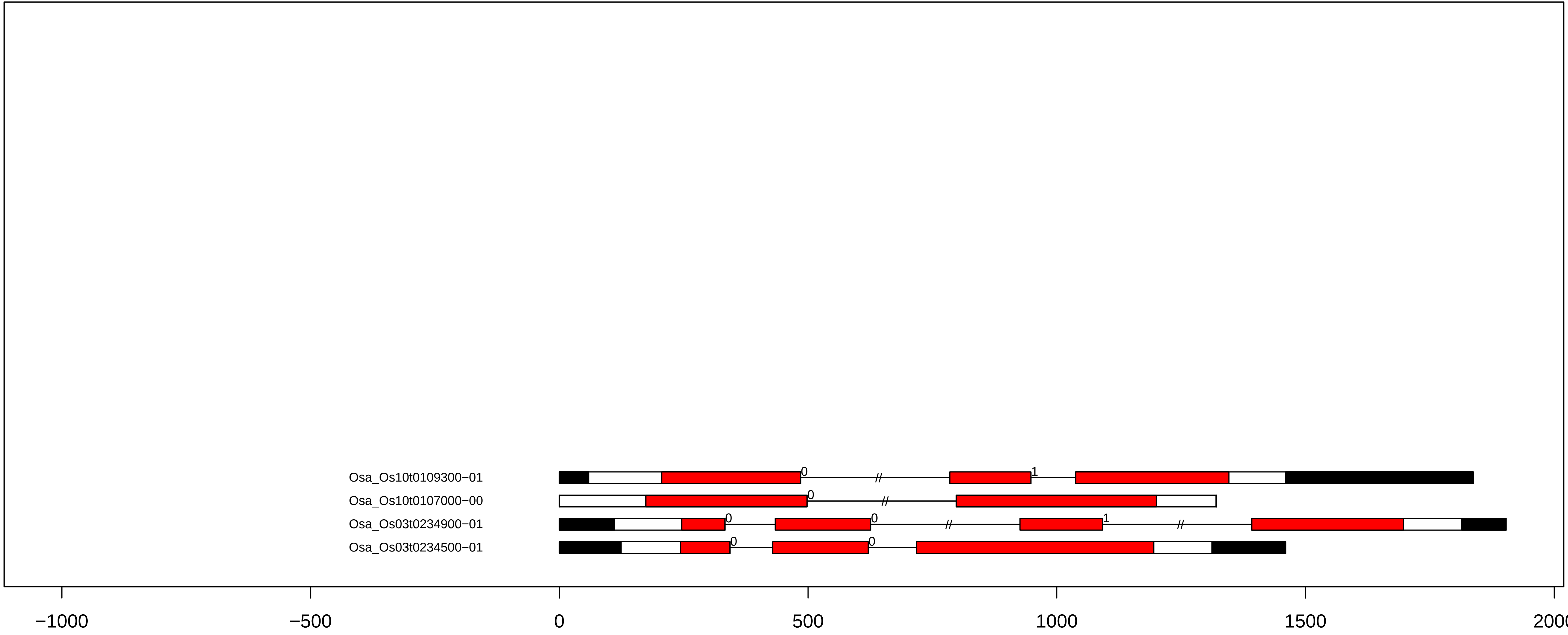

O.sa class III peroxidase XIII subfamily exon−intron and prx domain diagram (all)

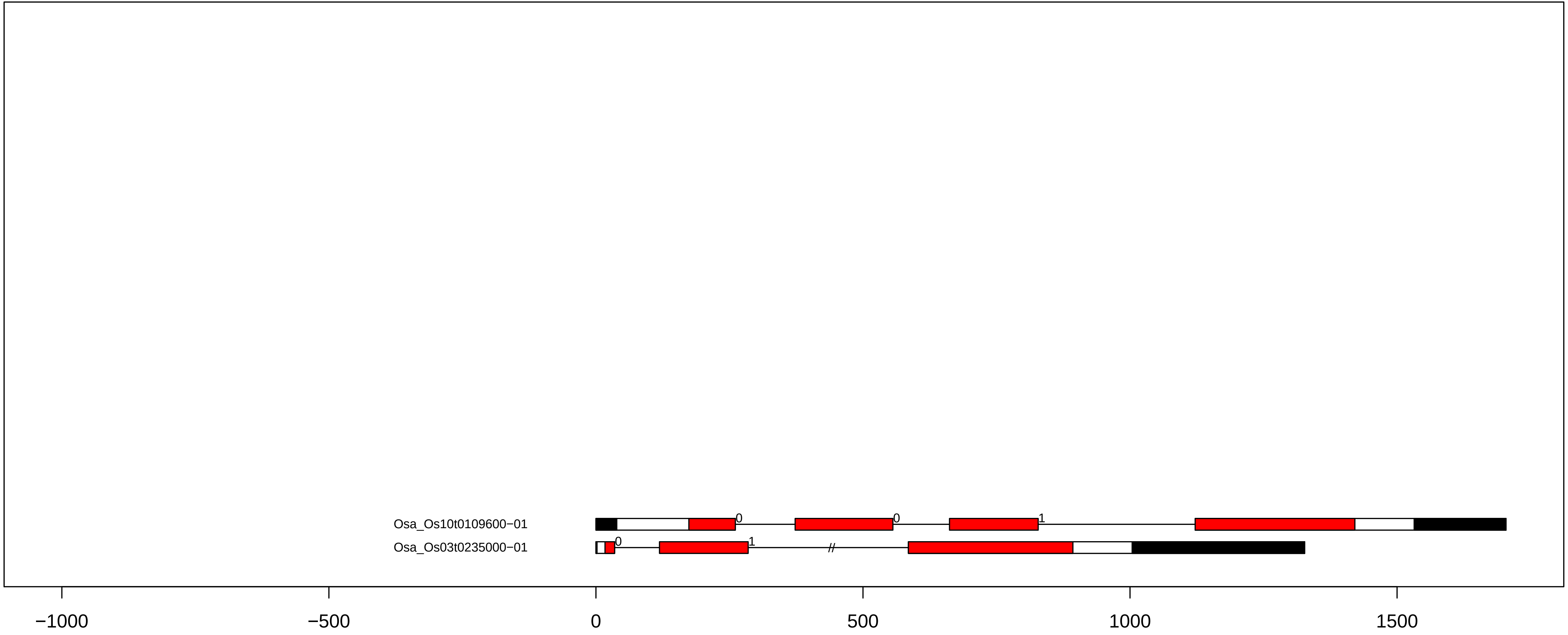

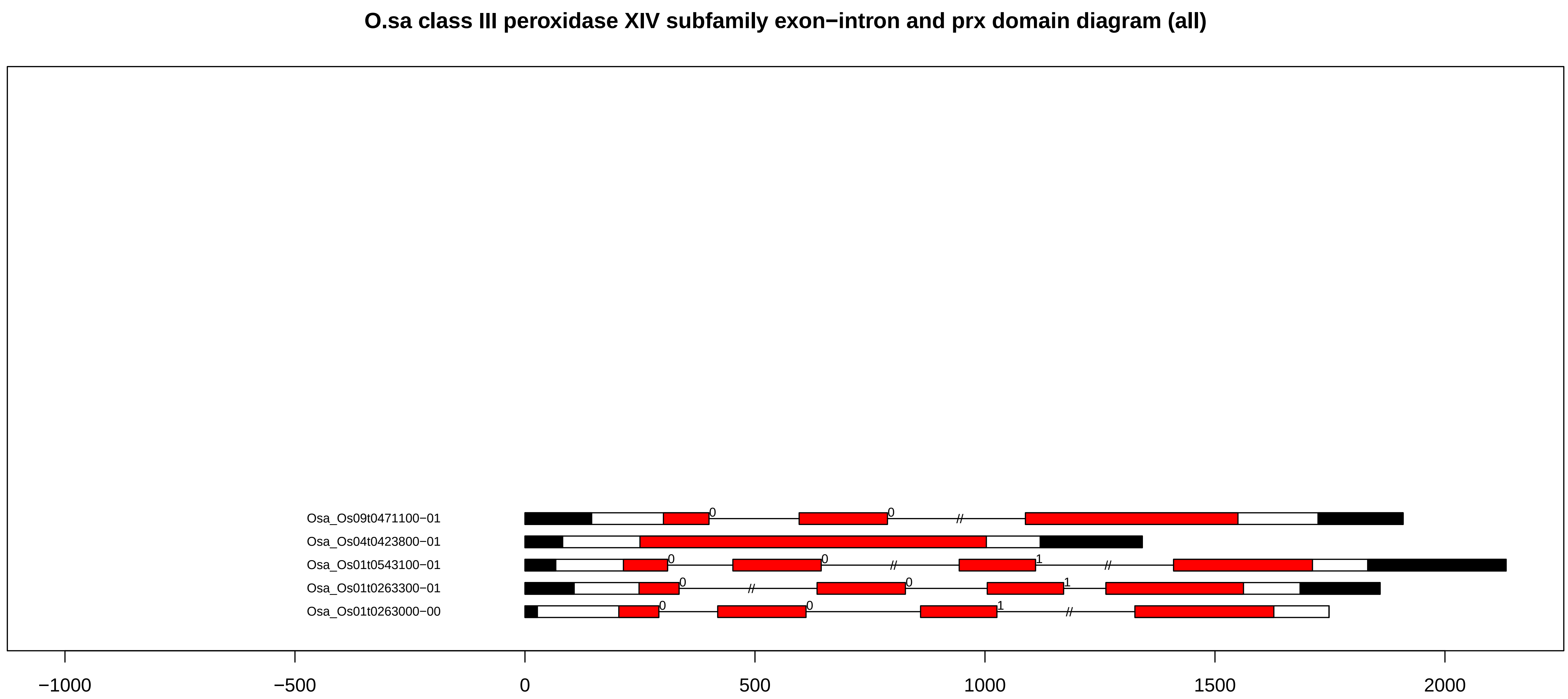
O.sa class III peroxidase XIV subfamily exon−intron and prx domain diagram (all)
Osa_Os09t0471100−01
Osa_Os04t0423800−01
Osa_Os01t0543100−01
Osa_Os01t0263300−01
Osa_Os01t0263000−00
−1000
−500
0
500
1000
1500
2000

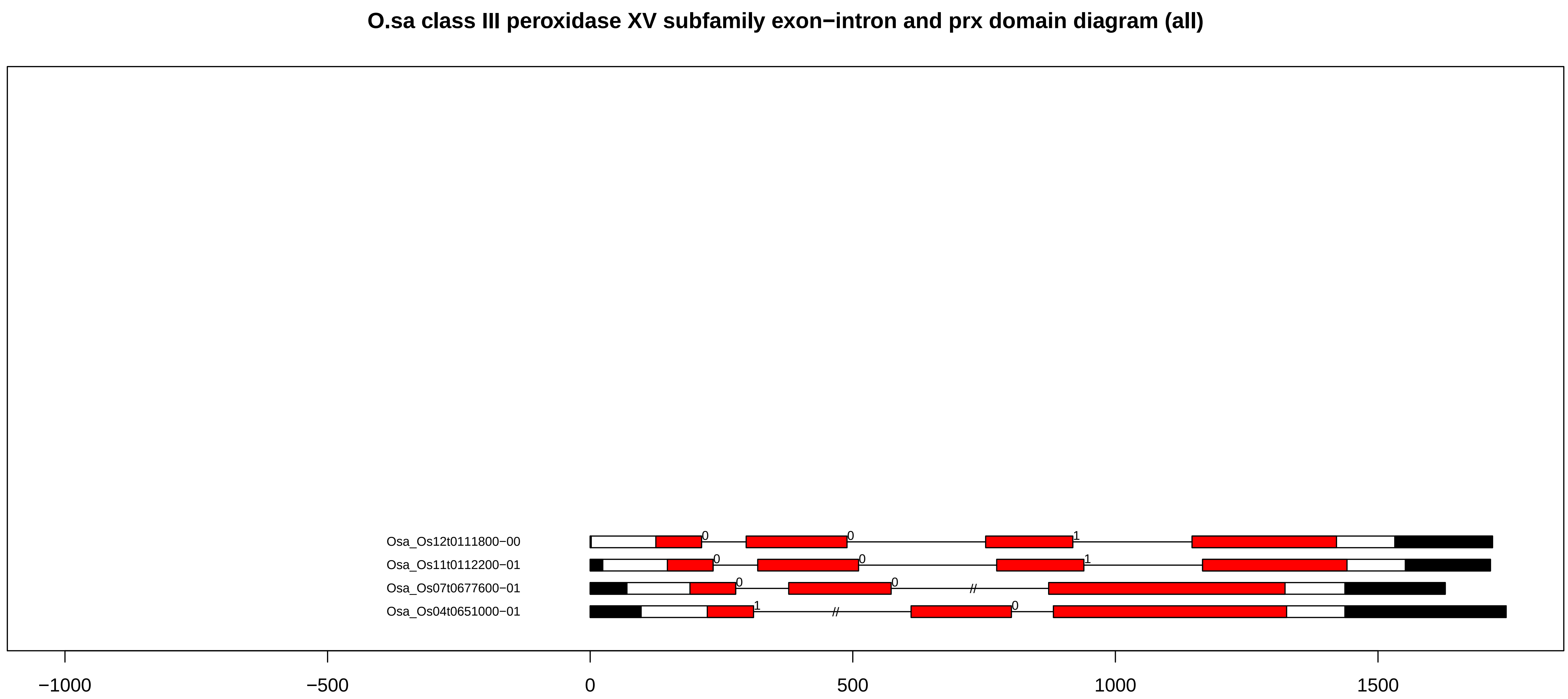
O.sa class III peroxidase XV subfamily exon−intron and prx domain diagram (all)
Osa_Os12t0111800−00
Osa_Os11t0112200−01
Osa_Os07t0677600−01
Osa_Os04t0651000−01
0
0
1
0
0
1
0
0
1
0
−1000
−500
0
500
1000
1500

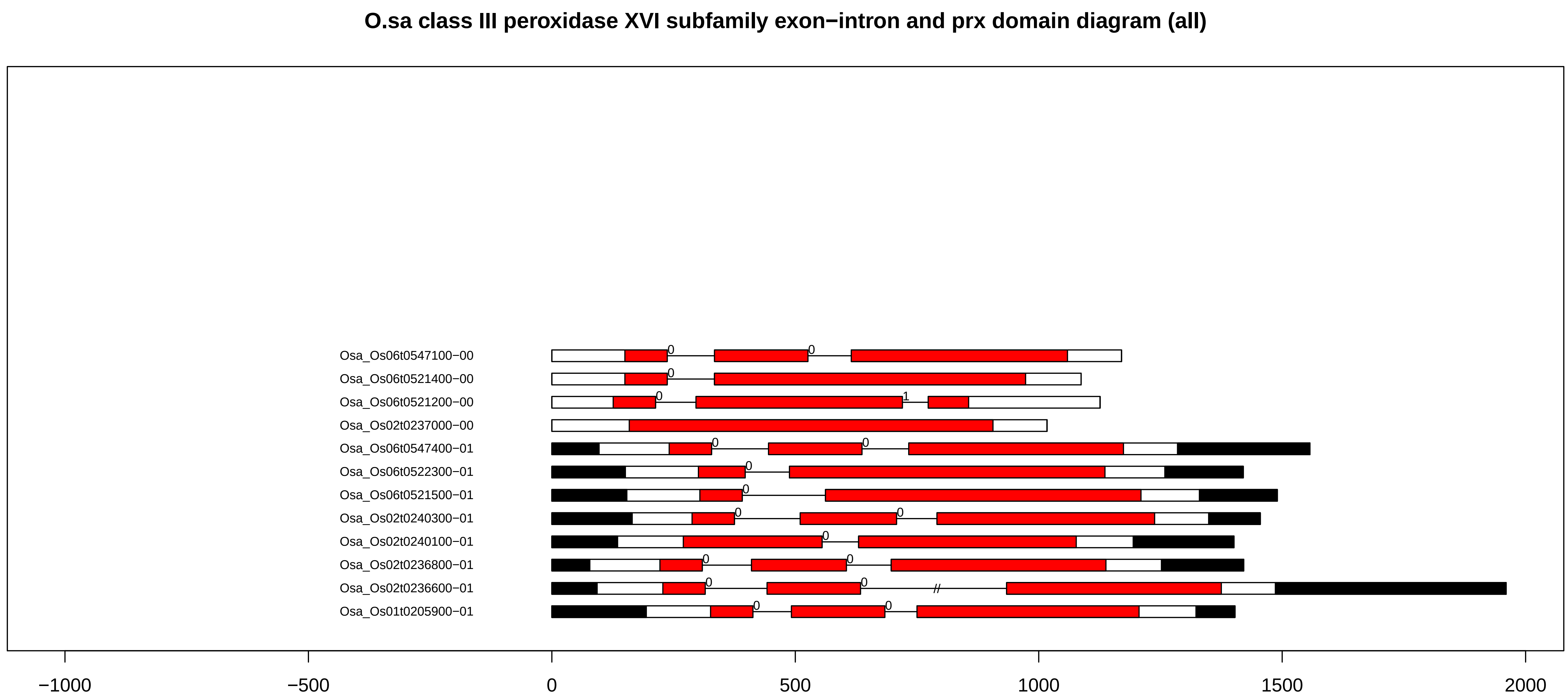
O.sa class III peroxidase XVI subfamily exon−intron and prx domain diagram (all)
Osa_Os06t0547100−00
Osa_Os06t0521400−00
Osa_Os06t0521200−00
Osa_Os02t0237000−00
Osa_Os06t0547400−01
Osa_Os06t0522300−01
Osa_Os06t0521500−01
Osa_Os02t0240300−01
Osa_Os02t0240100−01
Osa_Os02t0236800−01
Osa_Os02t0236600−01
Osa_Os01t0205900−01
−1000
−500
0
500
1000
1500
2000

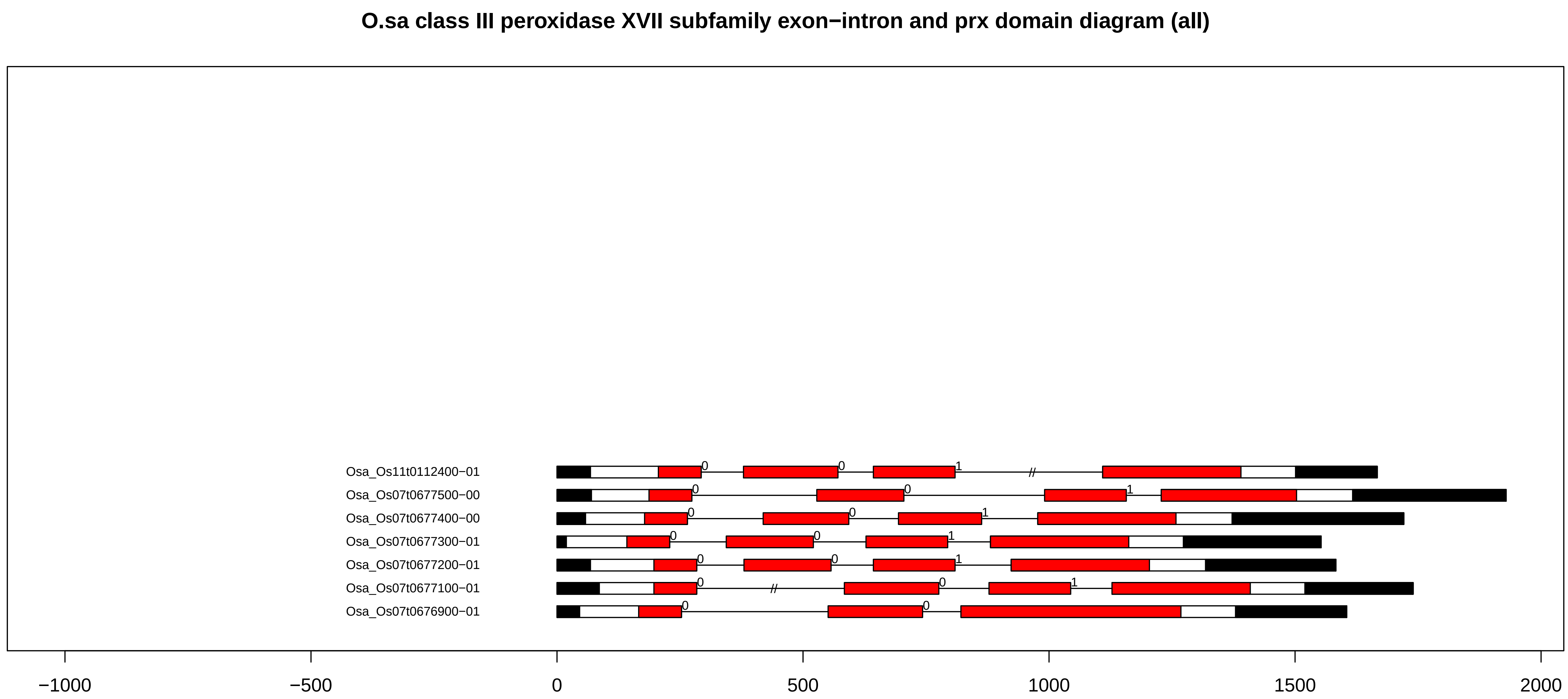

O.sa class III peroxidase XVII subfamily exon−intron and prx domain diagram (all)
Osa_Os11t0112400−01
Osa_Os07t0677500−00
Osa_Os07t0677400−00
Osa_Os07t0677300−01
Osa_Os07t0677200−01
Osa_Os07t0677100−01
Osa_Os07t0676900−01
−1000
−500
0
500
1000
1500
2000

O.sa class III peroxidase XVIII subfamily exon−intron and prx domain diagram (all)

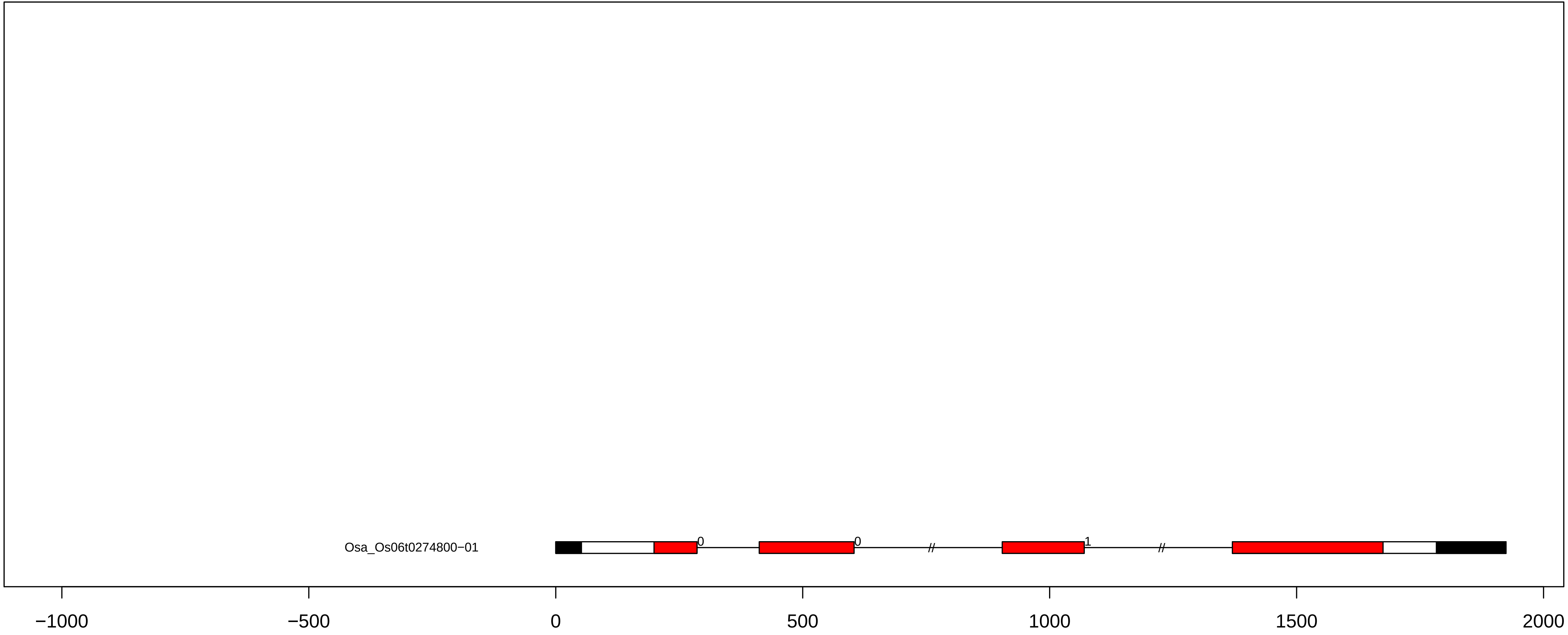

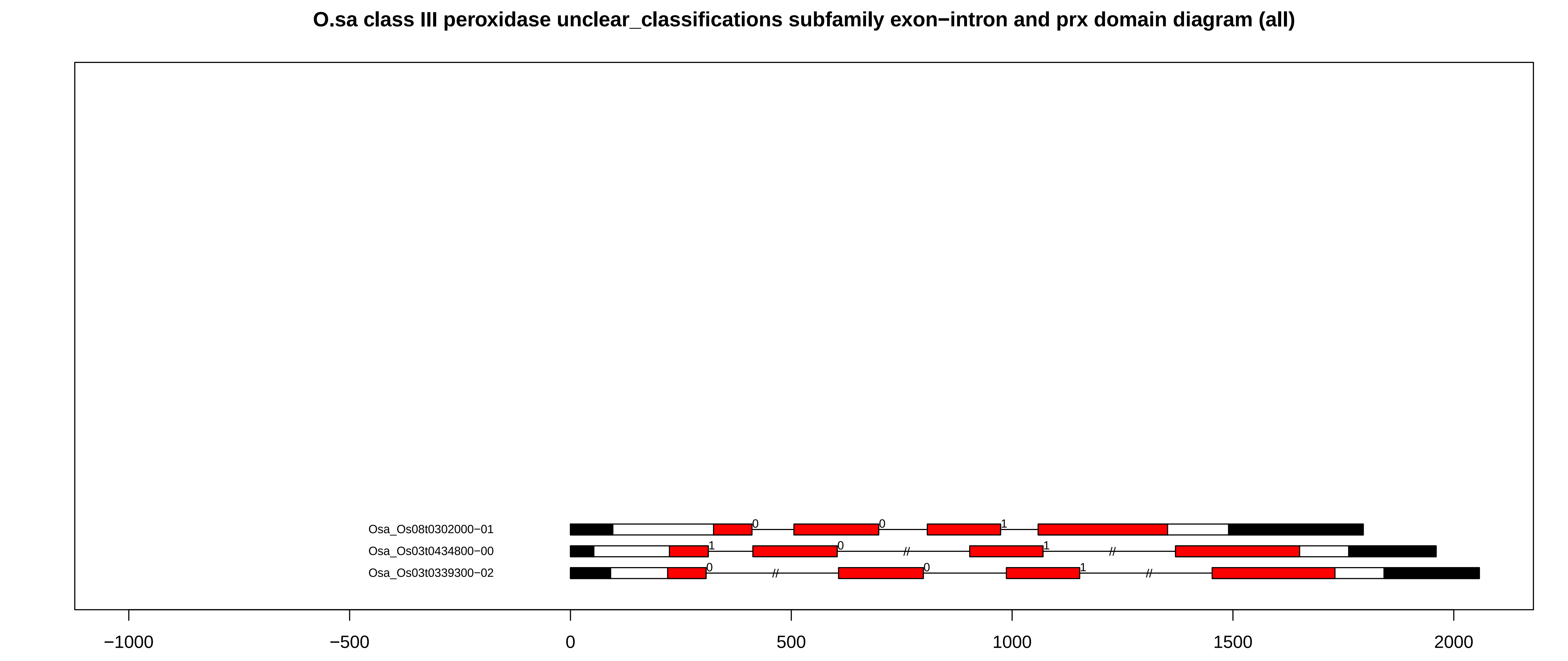

O.sa class III peroxidase unclear_classifications subfamily exon−intron and prx domain diagram (all)
Osa_Os08t0302000−01
Osa_Os03t0434800−00
Osa_Os03t0339300−02
0
0
1
1
0
1
0
0
1
−1000
−500
0
500
1000
1500
2000

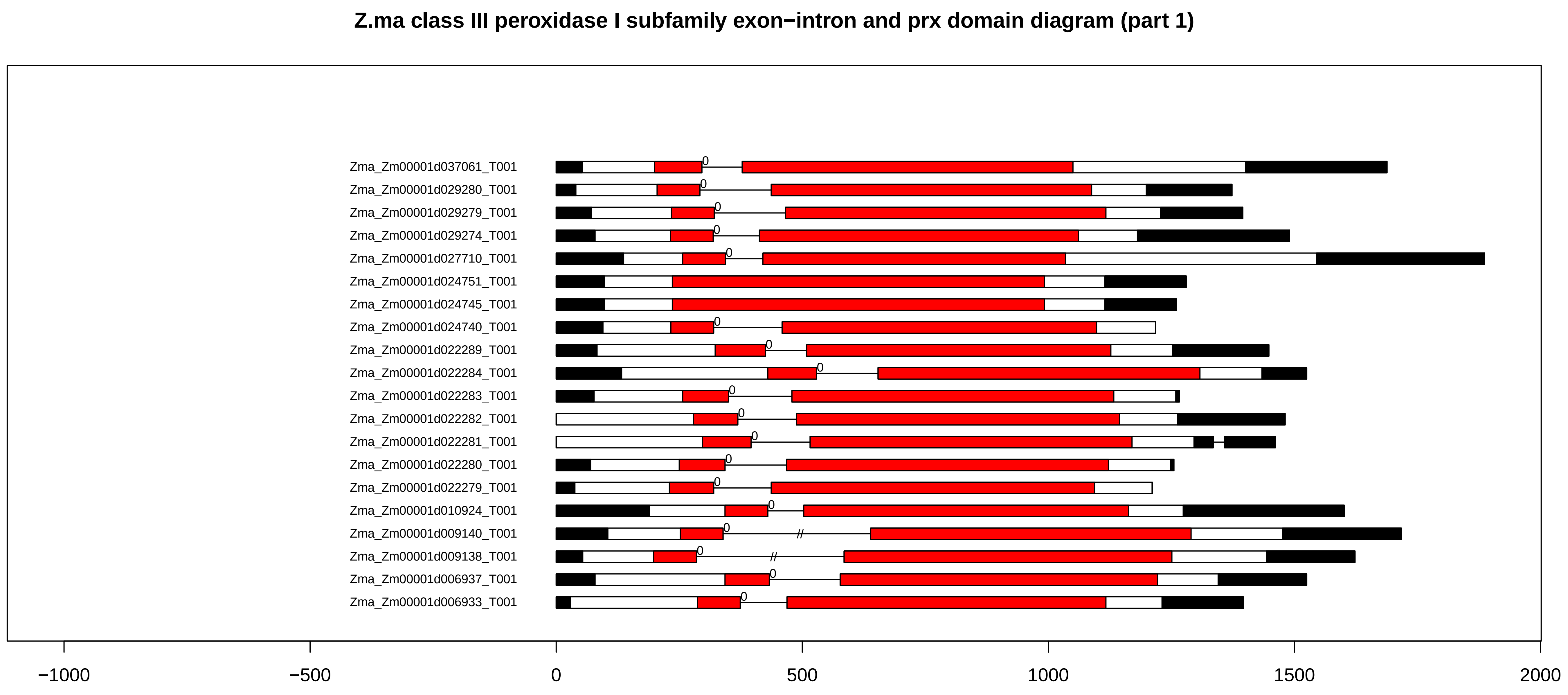

Z.ma class III peroxidase I subfamily exon−intron and prx domain diagram (part 1)
Zma_Zm00001d037061_T001
Zma_Zm00001d029280_T001
Zma_Zm00001d029279_T001
Zma_Zm00001d029274_T001
Zma_Zm00001d027710_T001
Zma_Zm00001d024751_T001
Zma_Zm00001d024745_T001
Zma_Zm00001d024740_T001
Zma_Zm00001d022289_T001
Zma_Zm00001d022284_T001
Zma_Zm00001d022283_T001
Zma_Zm00001d022282_T001
Zma_Zm00001d022281_T001
Zma_Zm00001d022280_T001
Zma_Zm00001d022279_T001
Zma_Zm00001d010924_T001
Zma_Zm00001d009140_T001
Zma_Zm00001d009138_T001
Zma_Zm00001d006937_T001
Zma_Zm00001d006933_T001
−1000
−500
0
500
1000
1500
2000

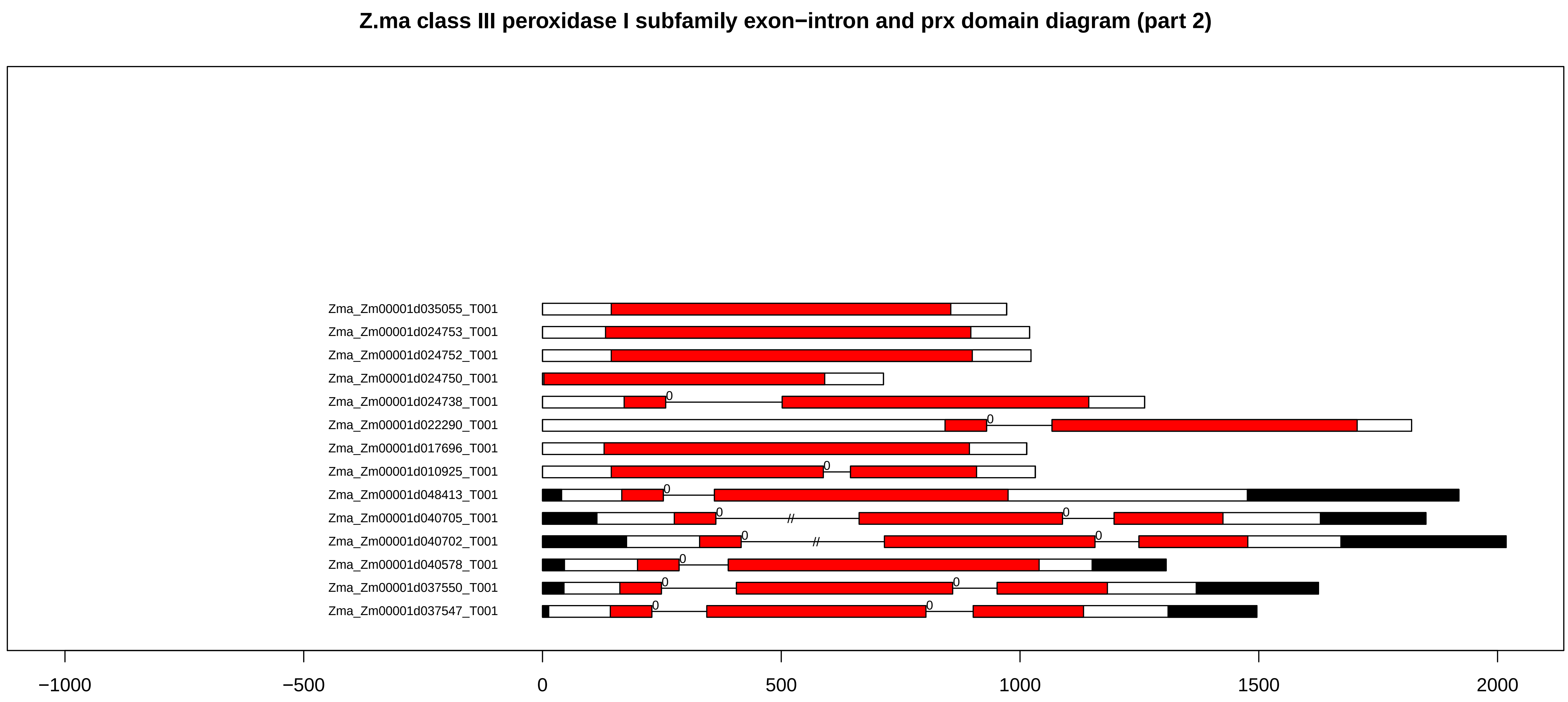
Z.ma class III peroxidase I subfamily exon−intron and prx domain diagram (part 2)
Zma_Zm00001d035055_T001
Zma_Zm00001d024753_T001
Zma_Zm00001d024752_T001
Zma_Zm00001d024750_T001
Zma_Zm00001d024738_T001
Zma_Zm00001d022290_T001
Zma_Zm00001d017696_T001
Zma_Zm00001d010925_T001
Zma_Zm00001d048413_T001
Zma_Zm00001d040705_T001
Zma_Zm00001d040702_T001
Zma_Zm00001d040578_T001
Zma_Zm00001d037550_T001
Zma_Zm00001d037547_T001
−1000
−500
0
500
1000
1500
2000

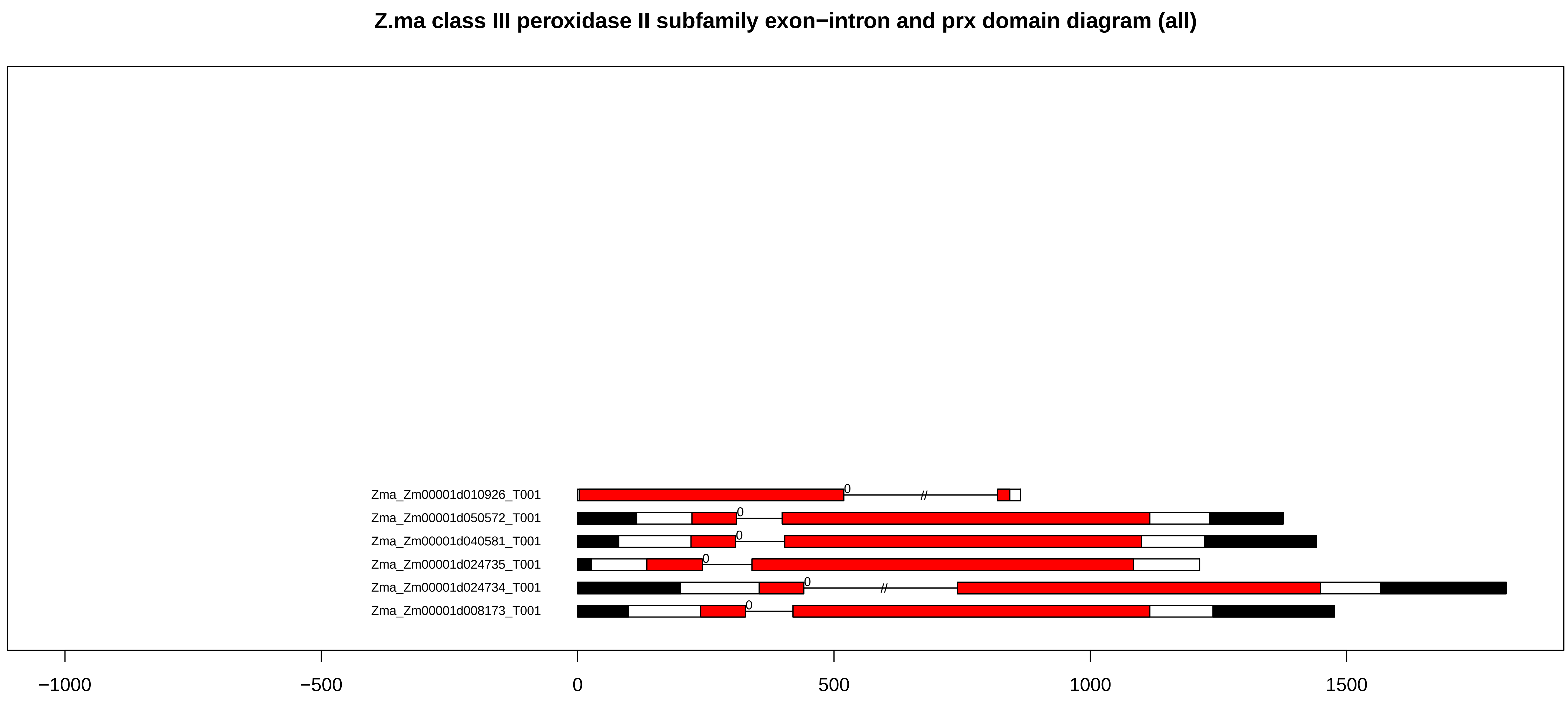
Z.ma class III peroxidase II subfamily exon−intron and prx domain diagram (all)
Zma_Zm00001d010926_T001
Zma_Zm00001d050572_T001
Zma_Zm00001d040581_T001
Zma_Zm00001d024735_T001
Zma_Zm00001d024734_T001
Zma_Zm00001d008173_T001
−1000
−500
0
500
1000
1500

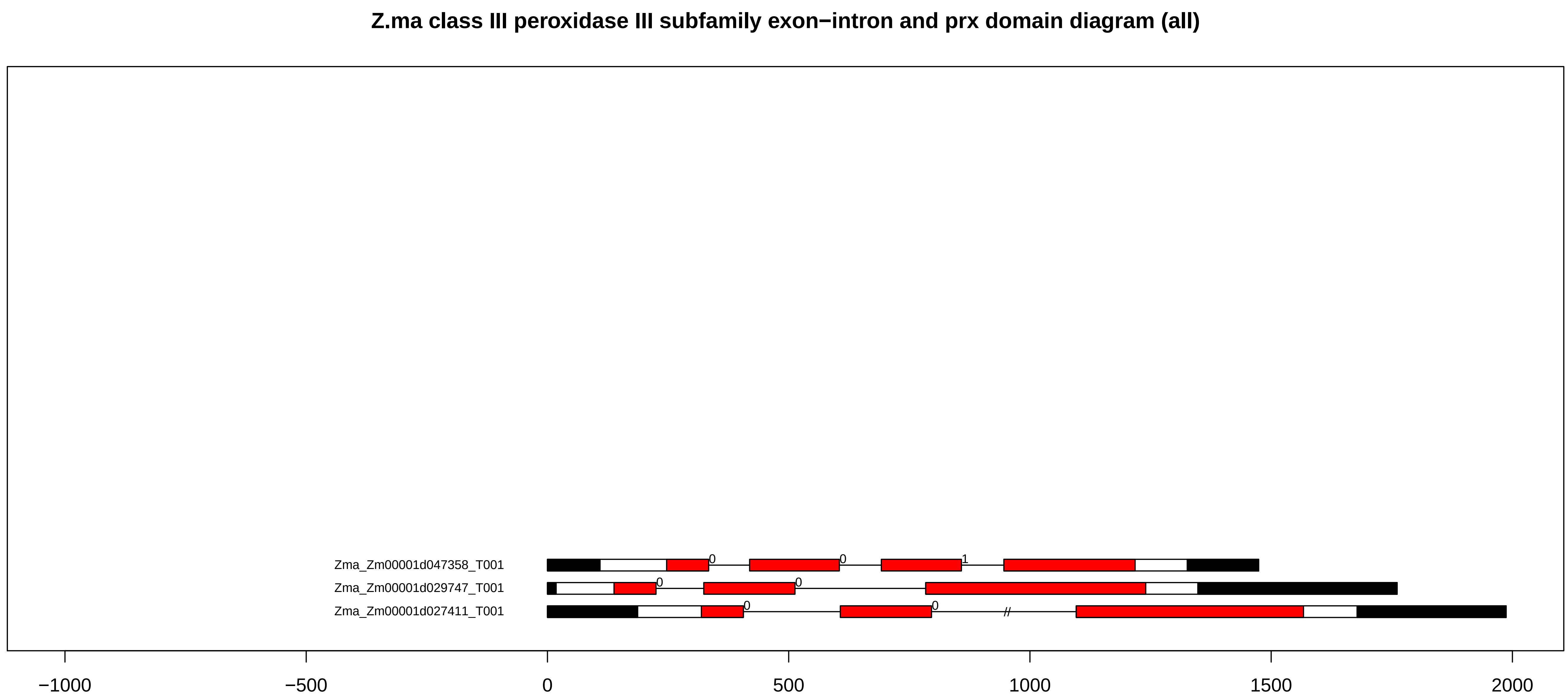
Z.ma class III peroxidase III subfamily exon−intron and prx domain diagram (all)
Zma_Zm00001d047358_T001
Zma_Zm00001d029747_T001
Zma_Zm00001d027411_T001
0
0
1
0
0
0
0
−1000
−500
0
500
1000
1500
2000

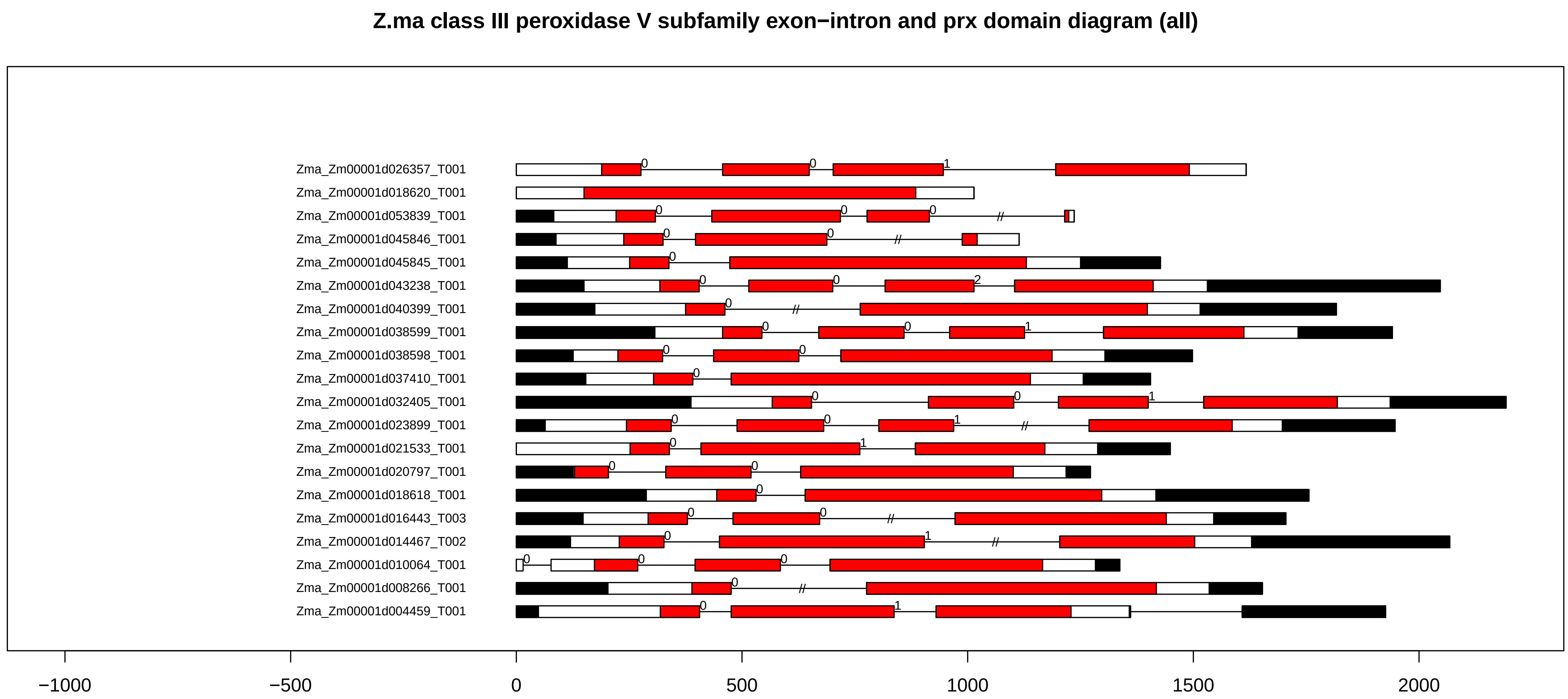

Z.ma class III peroxidase V subfamily exon−intron and prx domain diagram (all)
Zma_Zm00001d026357_T001
Zma_Zm00001d018620_T001
Zma_Zm00001d053839_T001
Zma_Zm00001d045846_T001
Zma_Zm00001d045845_T001
Zma_Zm00001d043238_T001
Zma_Zm00001d040399_T001
Zma_Zm00001d038599_T001
Zma_Zm00001d038598_T001
Zma_Zm00001d037410_T001
Zma_Zm00001d032405_T001
Zma_Zm00001d023899_T001
Zma_Zm00001d021533_T001
Zma_Zm00001d020797_T001
Zma_Zm00001d018618_T001
Zma_Zm00001d016443_T003
Zma_Zm00001d014467_T002
Zma_Zm00001d010064_T001
Zma_Zm00001d008266_T001
Zma_Zm00001d004459_T001
−1000
−500
0
500
1000
1500
2000

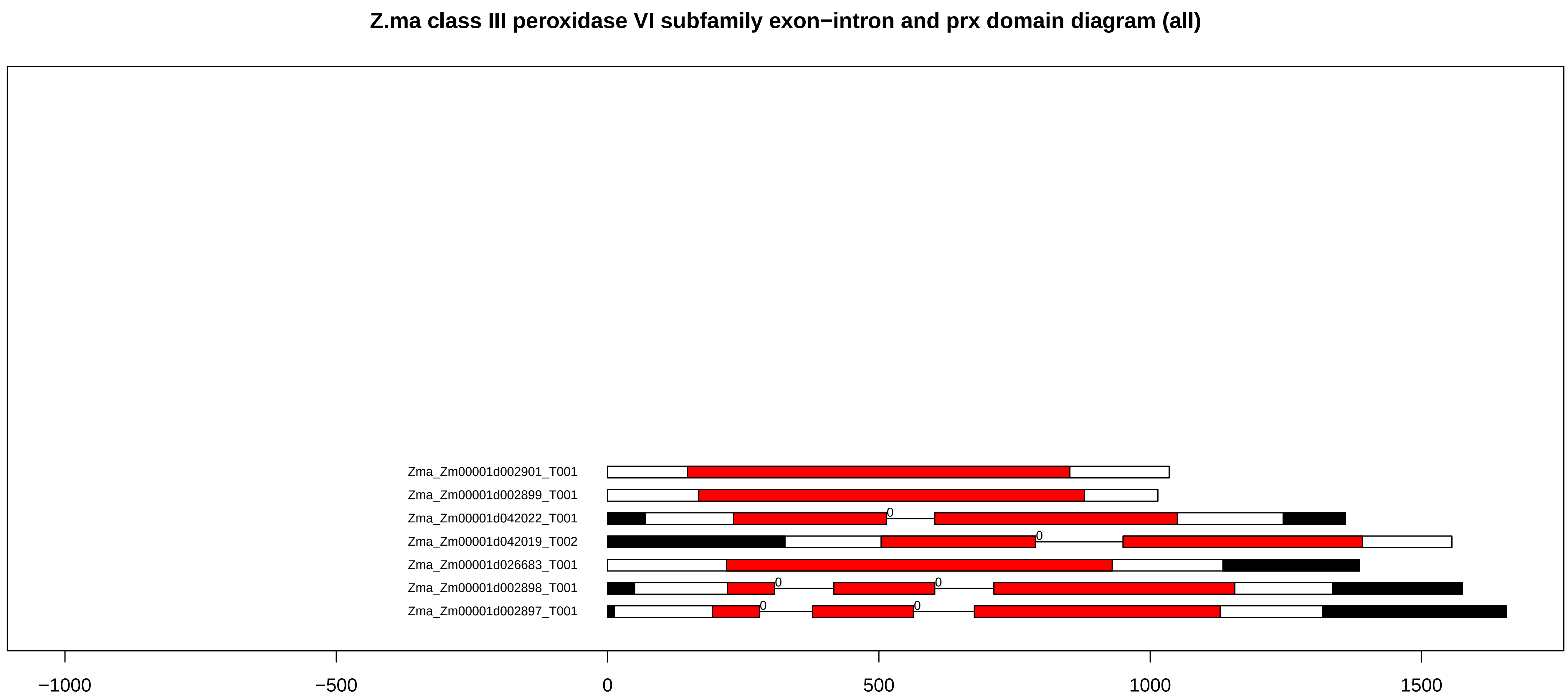

Z.ma class III peroxidase VI subfamily exon−intron and prx domain diagram (all)
Zma_Zm00001d002901_T001
Zma_Zm00001d002899_T001
Zma_Zm00001d042022_T001
Zma_Zm00001d042019_T002
Zma_Zm00001d026683_T001
Zma_Zm00001d002898_T001
Zma_Zm00001d002897_T001
0
0
0
0
0
0
−1000
−500
0
500
1000
1500

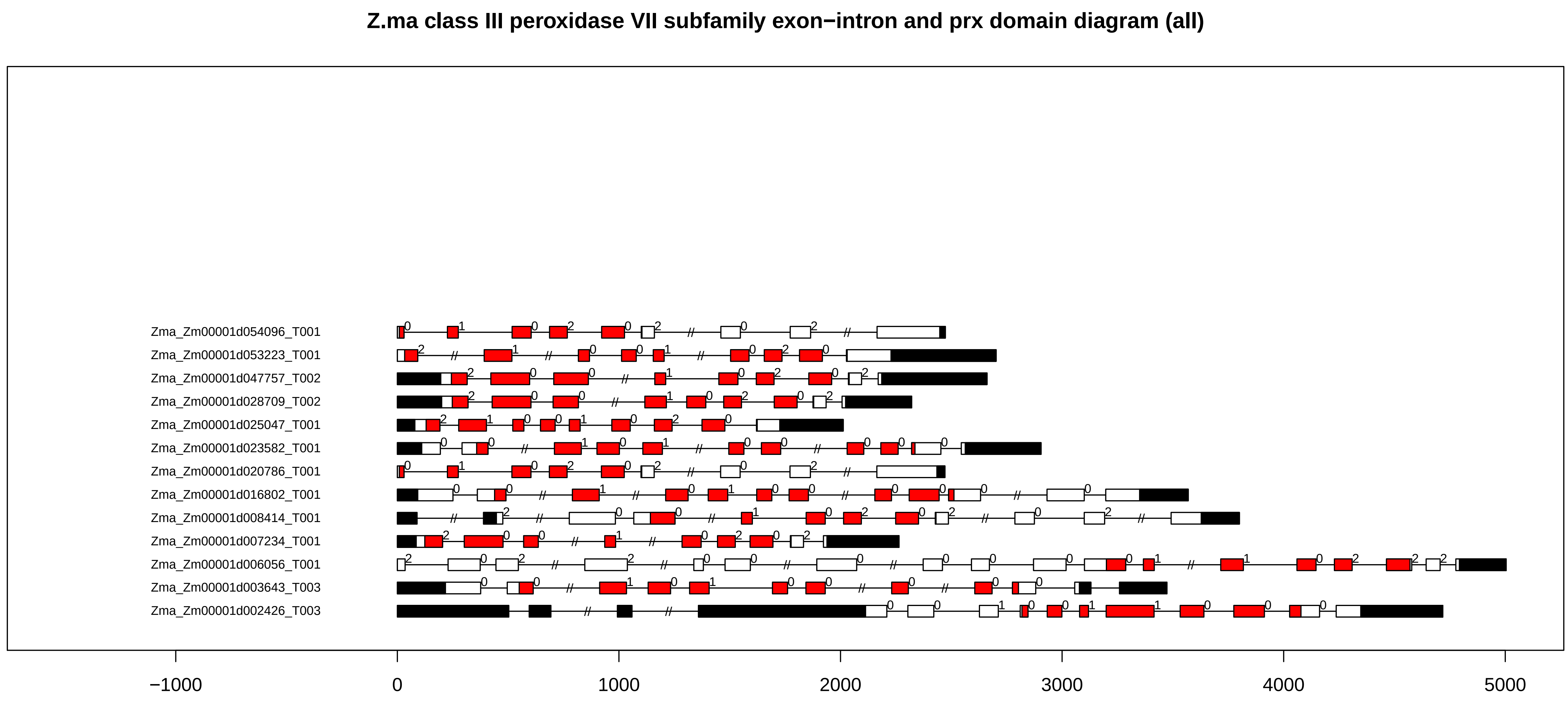

Z.ma class III peroxidase VII subfamily exon−intron and prx domain diagram (all)
Zma_Zm00001d054096_T001
Zma_Zm00001d053223_T001
Zma_Zm00001d047757_T002
Zma_Zm00001d028709_T002
Zma_Zm00001d025047_T001
Zma_Zm00001d023582_T001
Zma_Zm00001d020786_T001
Zma_Zm00001d016802_T001
Zma_Zm00001d008414_T001
Zma_Zm00001d007234_T001
Zma_Zm00001d006056_T001
Zma_Zm00001d003643_T003
Zma_Zm00001d002426_T003
−1000
0
1000
2000
3000
4000
5000

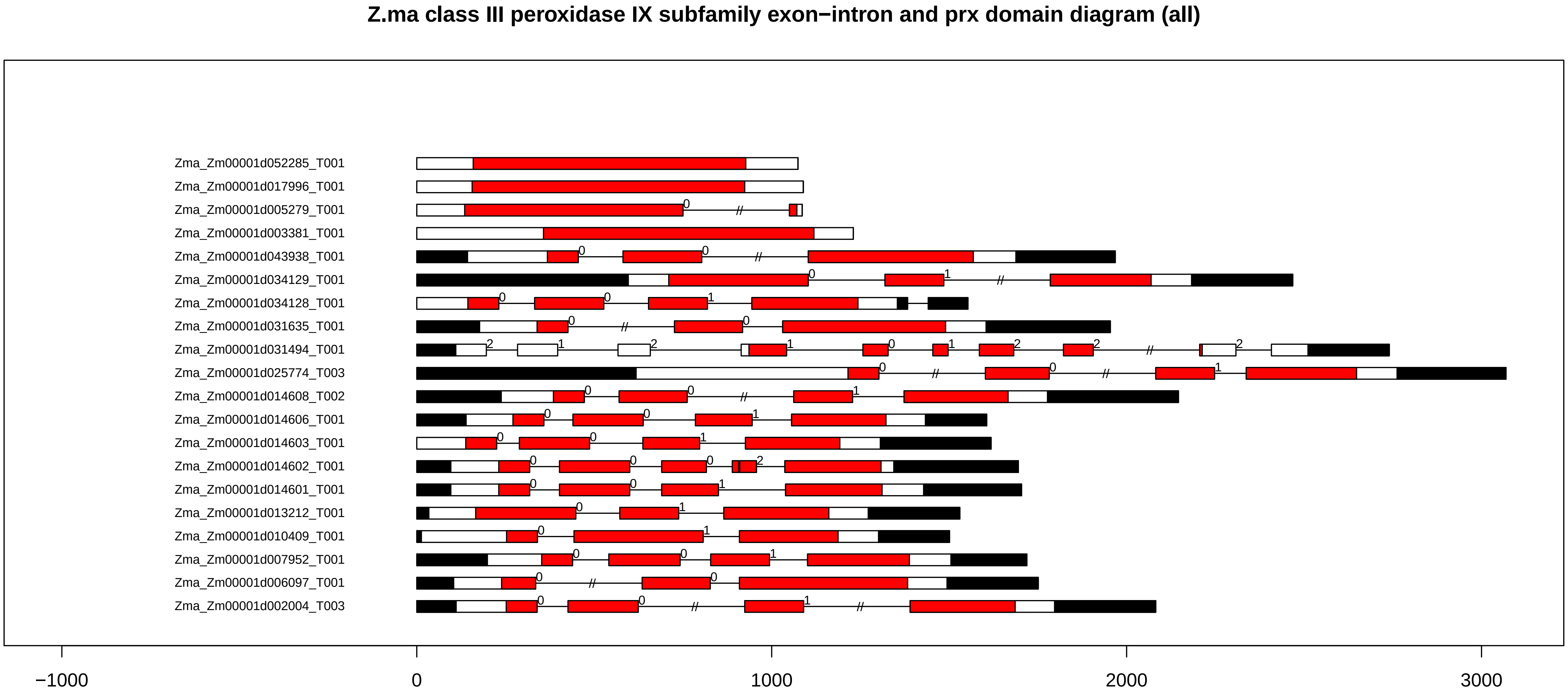
Z.ma class III peroxidase IX subfamily exon−intron and prx domain diagram (all)
Zma_Zm00001d052285_T001
Zma_Zm00001d017996_T001
Zma_Zm00001d005279_T001
Zma_Zm00001d003381_T001
Zma_Zm00001d043938_T001
Zma_Zm00001d034129_T001
Zma_Zm00001d034128_T001
Zma_Zm00001d031635_T001
Zma_Zm00001d031494_T001
Zma_Zm00001d025774_T003
Zma_Zm00001d014608_T002
Zma_Zm00001d014606_T001
Zma_Zm00001d014603_T001
Zma_Zm00001d014602_T001
Zma_Zm00001d014601_T001
Zma_Zm00001d013212_T001
Zma_Zm00001d010409_T001
Zma_Zm00001d007952_T001
Zma_Zm00001d006097_T001
Zma_Zm00001d002004_T003
−1000
0
1000
2000
3000

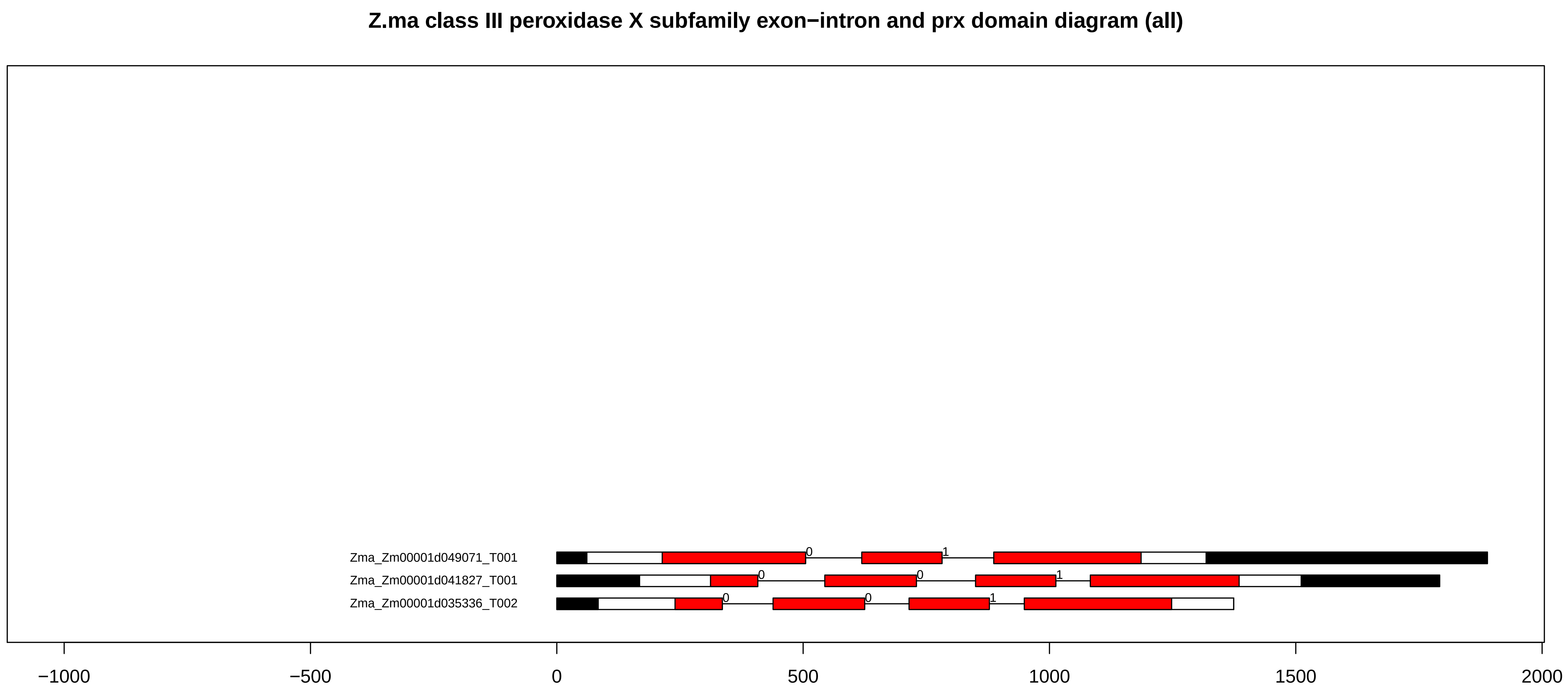

Z.ma class III peroxidase X subfamily exon−intron and prx domain diagram (all)
Zma_Zm00001d049071_T001
Zma_Zm00001d041827_T001
Zma_Zm00001d035336_T002
0
1
0
0
1
0
0
1
−1000
−500
0
500
1000
1500
2000

Z.ma class III peroxidase XI subfamily exon−intron and prx domain diagram (all)

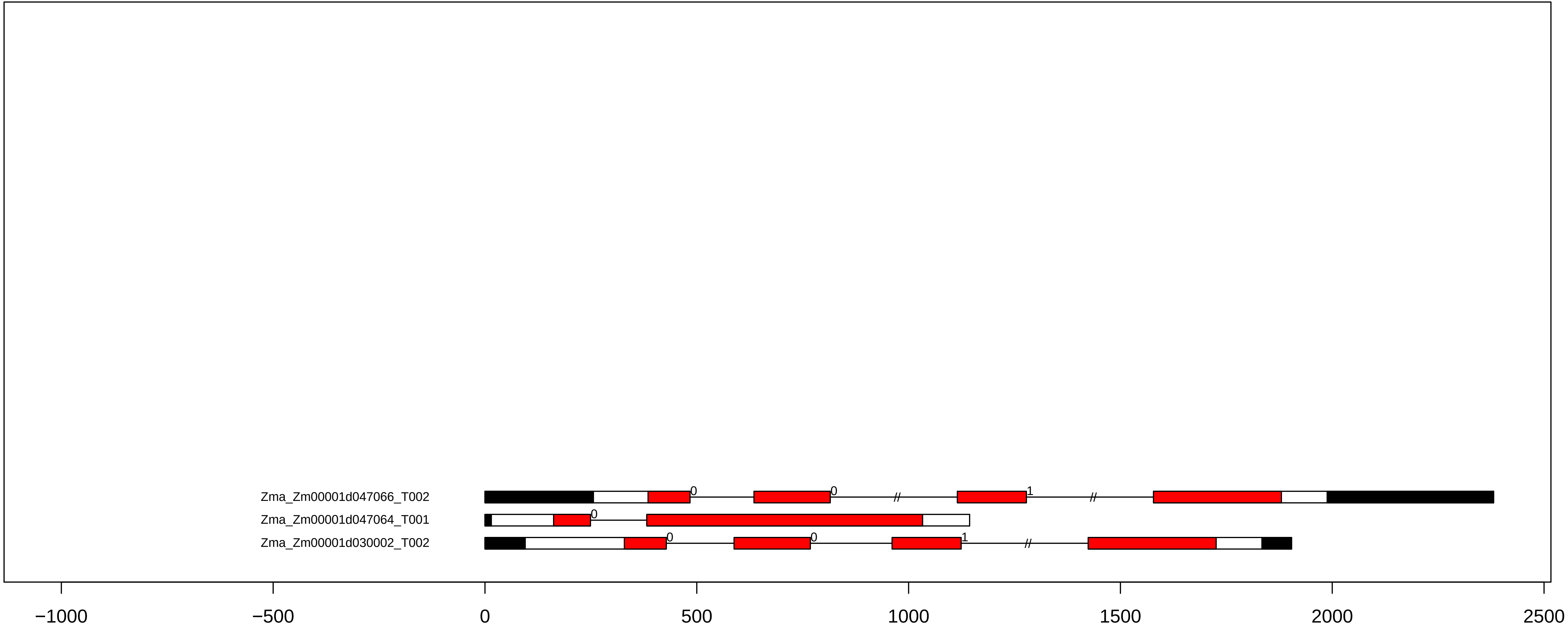

Z.ma class III peroxidase XII subfamily exon−intron and prx domain diagram (all)

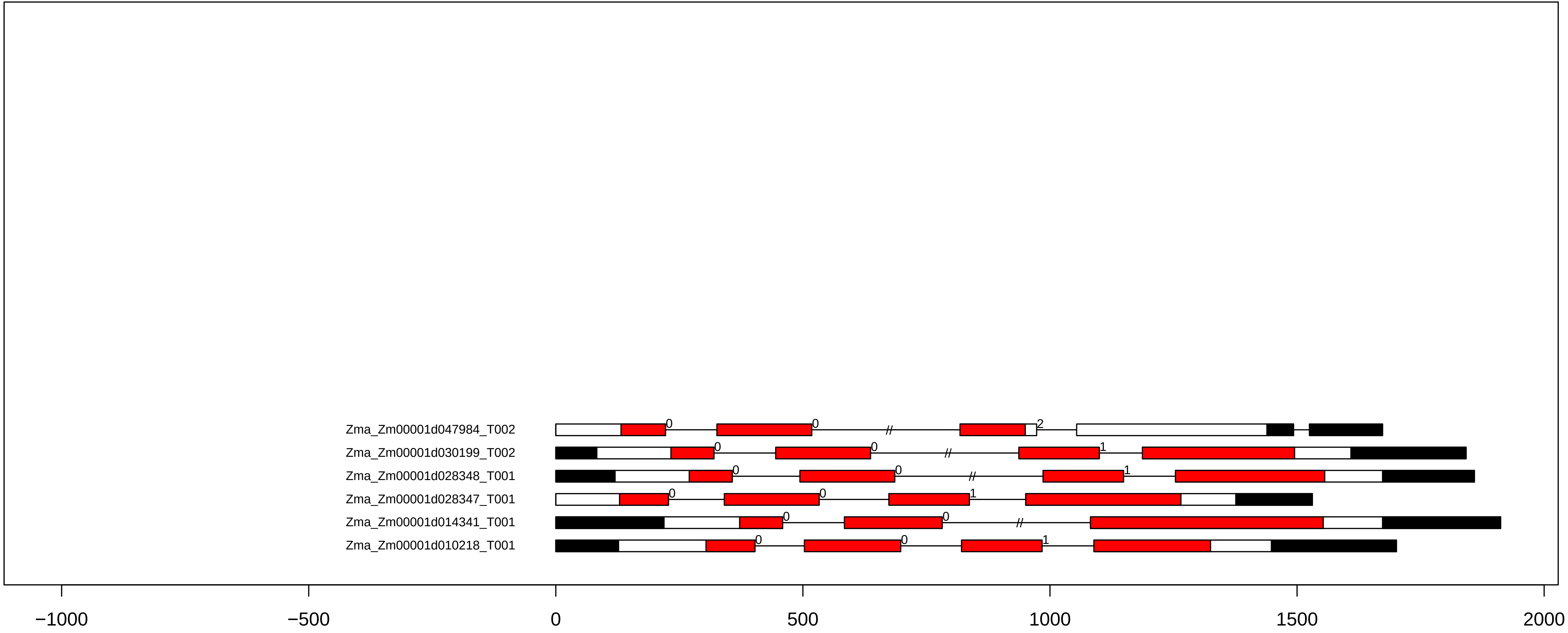

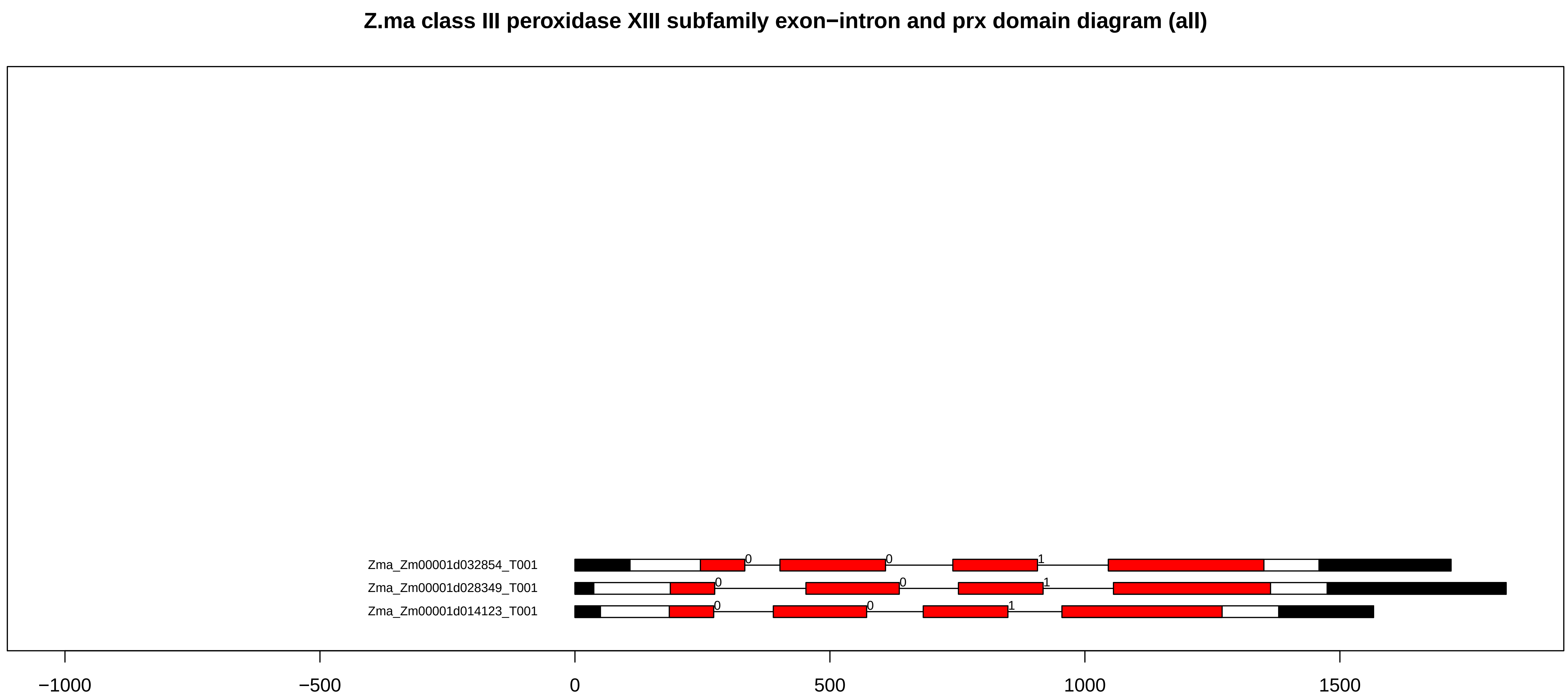

Z.ma class III peroxidase XIII subfamily exon−intron and prx domain diagram (all)
Zma_Zm00001d032854_T001
Zma_Zm00001d028349_T001
Zma_Zm00001d014123_T001
0
0
1
0
0
1
0
0
1
−1000
−500
0
500
1000
1500

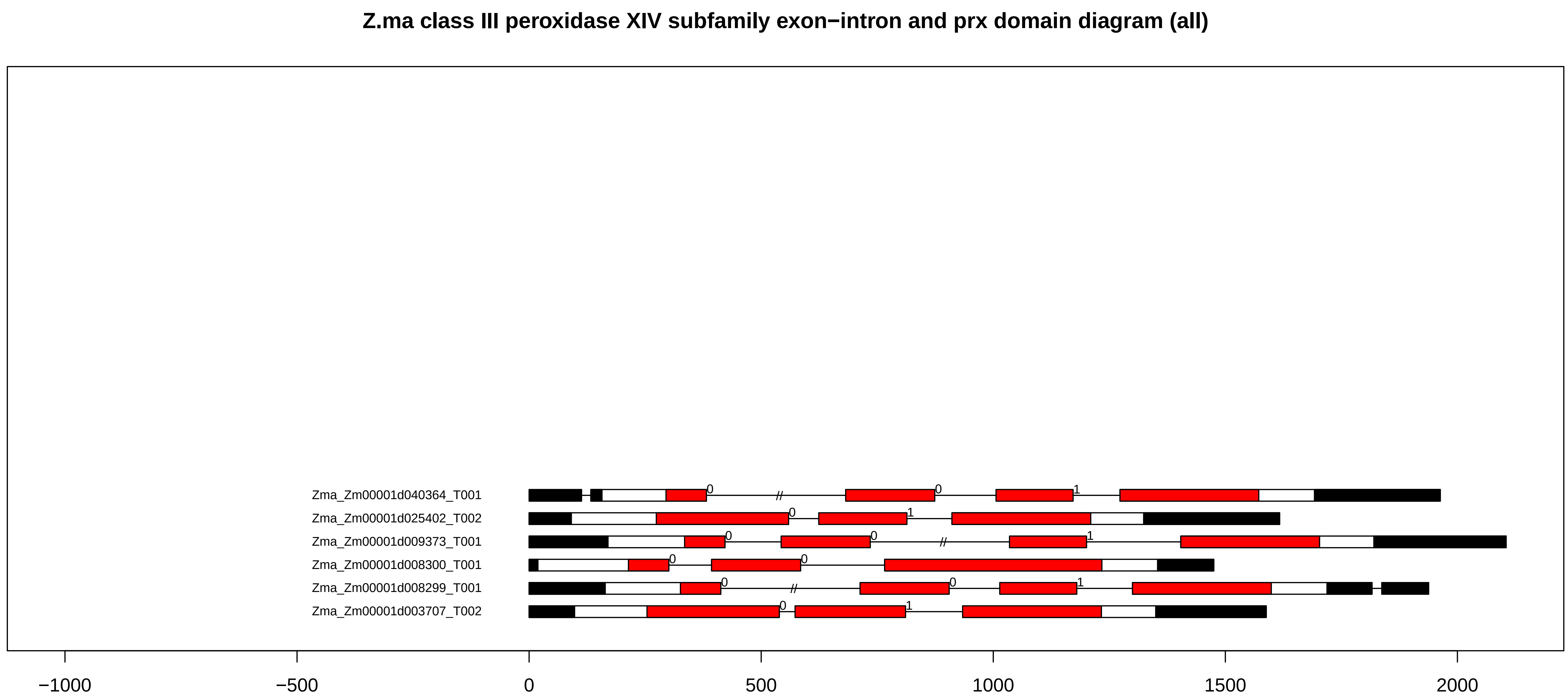
Z.ma class III peroxidase XIV subfamily exon−intron and prx domain diagram (all)
Zma_Zm00001d040364_T001
Zma_Zm00001d025402_T002
Zma_Zm00001d009373_T001
Zma_Zm00001d008300_T001
Zma_Zm00001d008299_T001
Zma_Zm00001d003707_T002
−1000
−500
0
500
1000
1500
2000

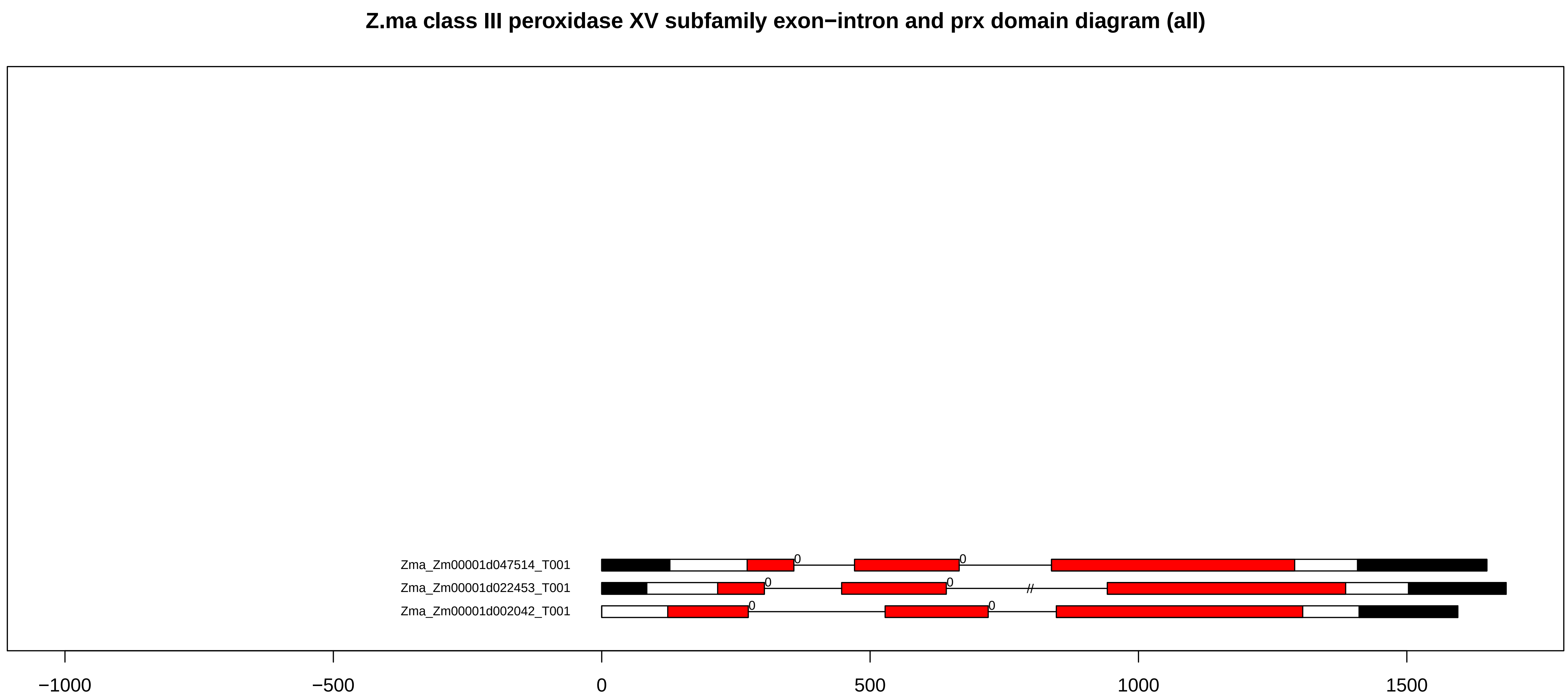
Z.ma class III peroxidase XV subfamily exon−intron and prx domain diagram (all)
Zma_Zm00001d047514_T001
Zma_Zm00001d022453_T001
Zma_Zm00001d002042_T001
0
0
0
0
0
0
−1000
−500
0
500
1000
1500

Z.ma class III peroxidase XVI subfamily exon−intron and prx domain diagram (all)

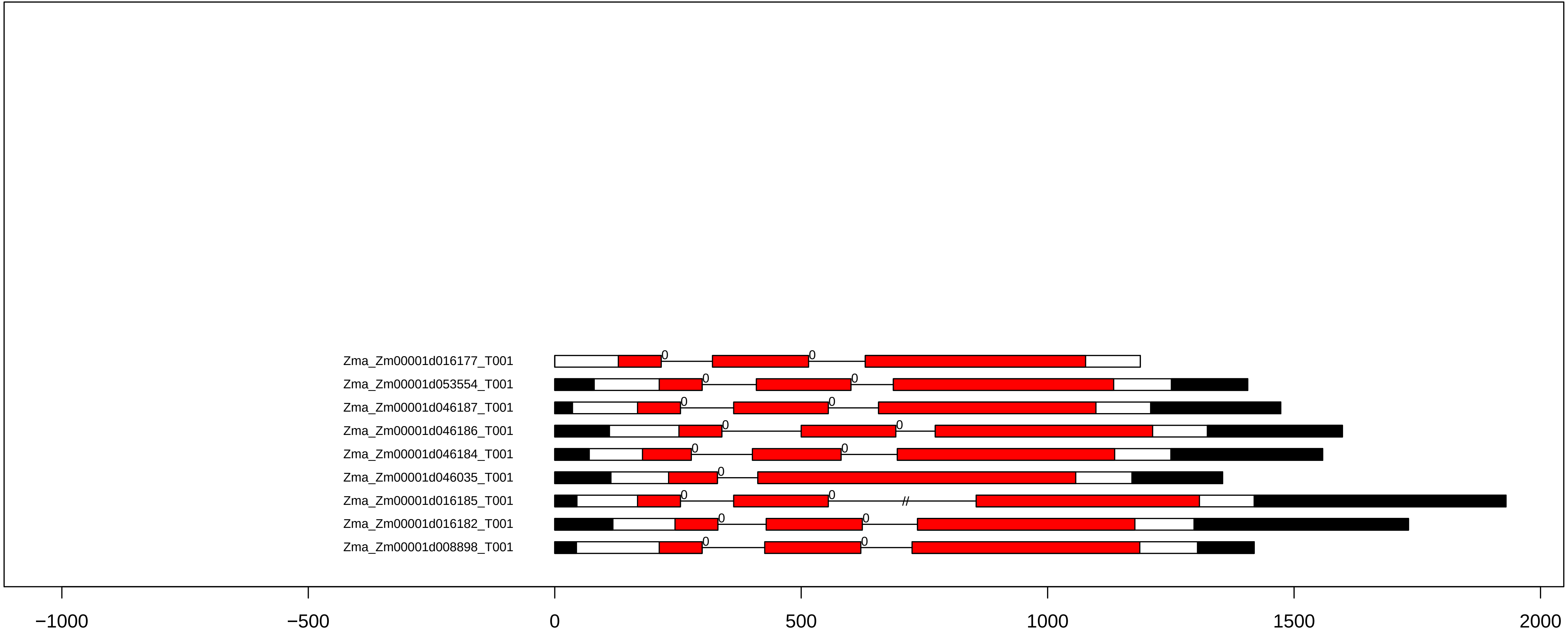

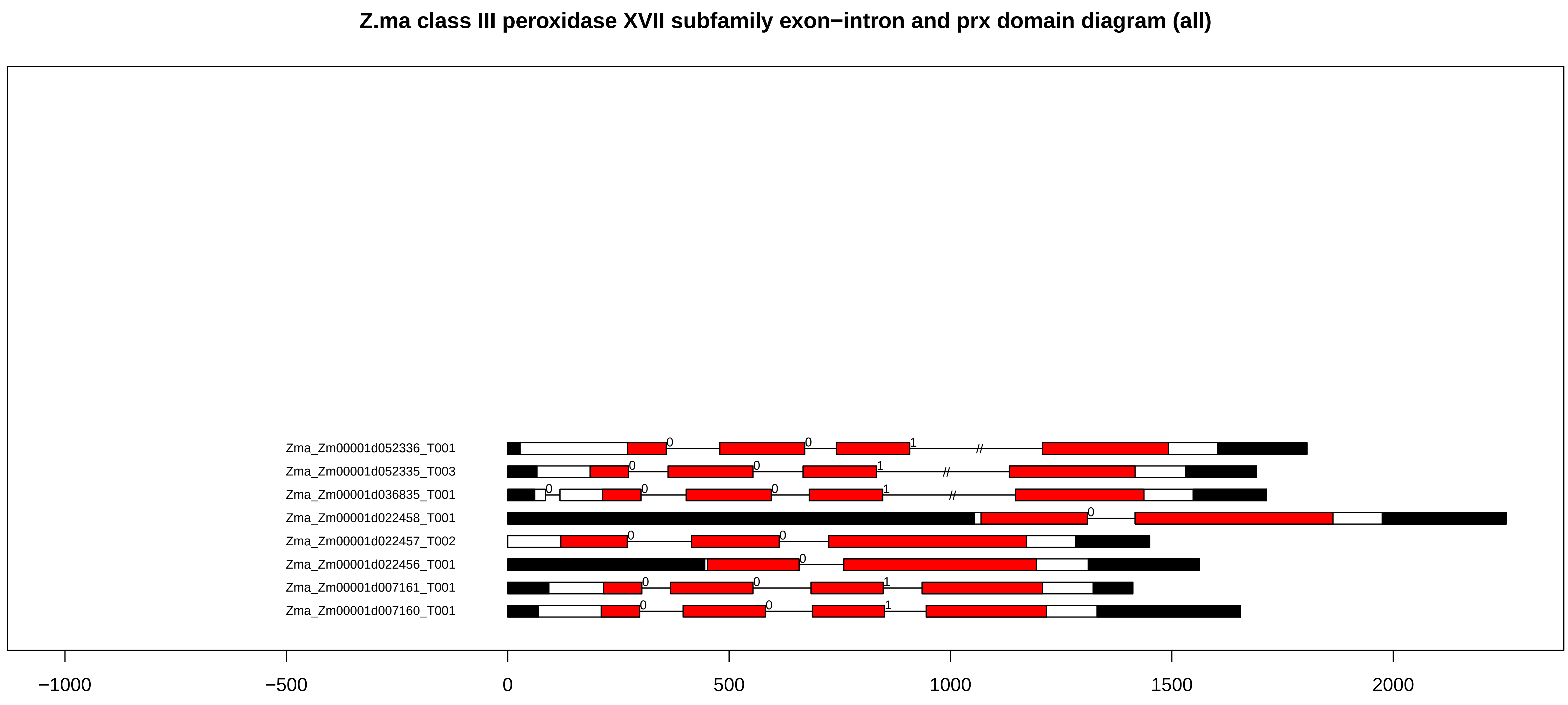

Z.ma class III peroxidase XVII subfamily exon−intron and prx domain diagram (all)
Zma_Zm00001d052336_T001
Zma_Zm00001d052335_T003
Zma_Zm00001d036835_T001
Zma_Zm00001d022458_T001
Zma_Zm00001d022457_T002
Zma_Zm00001d022456_T001
Zma_Zm00001d007161_T001
Zma_Zm00001d007160_T001
−1000
−500
0
500
1000
1500
2000

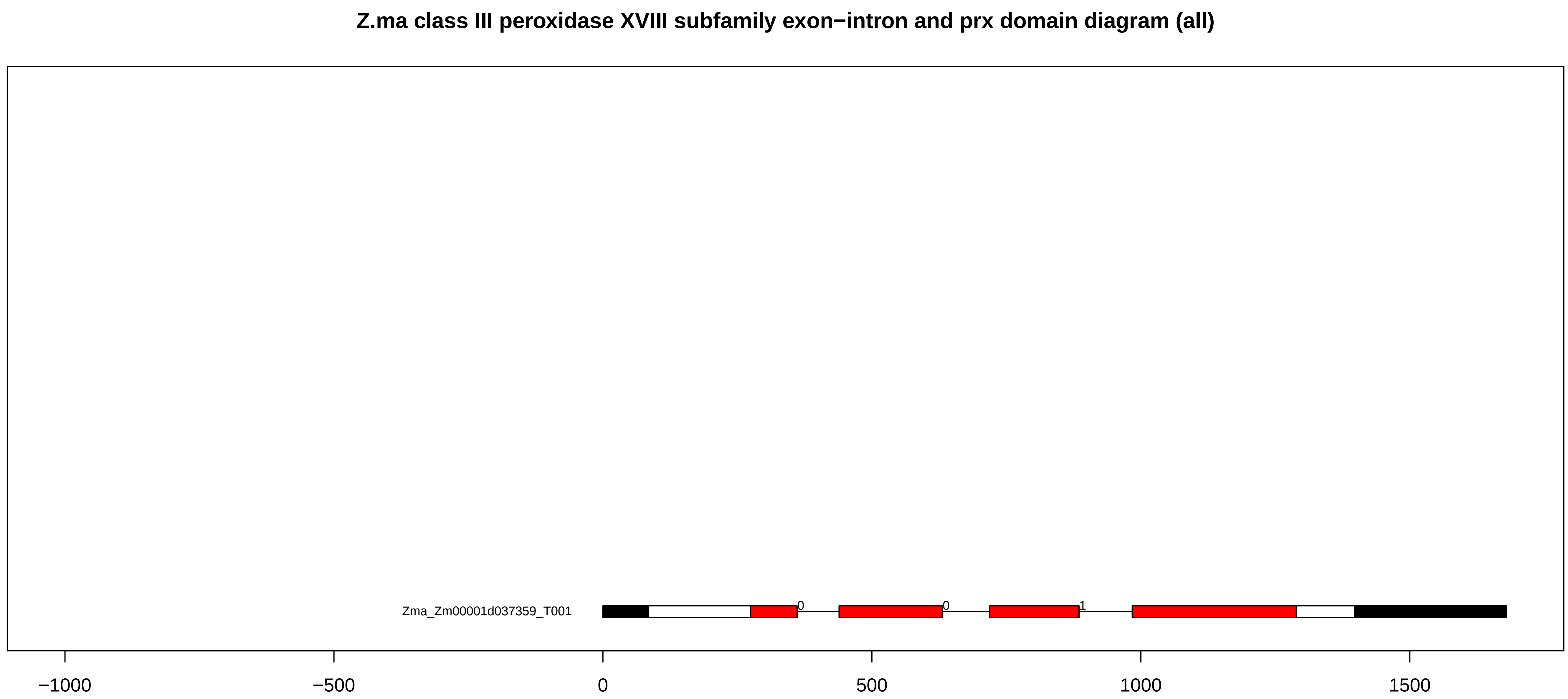

Z.ma class III peroxidase XVIII subfamily exon−intron and prx domain diagram (all)
Zma_Zm00001d037359_T001
0
0
1
−1000
−500
0
500
1000
1500

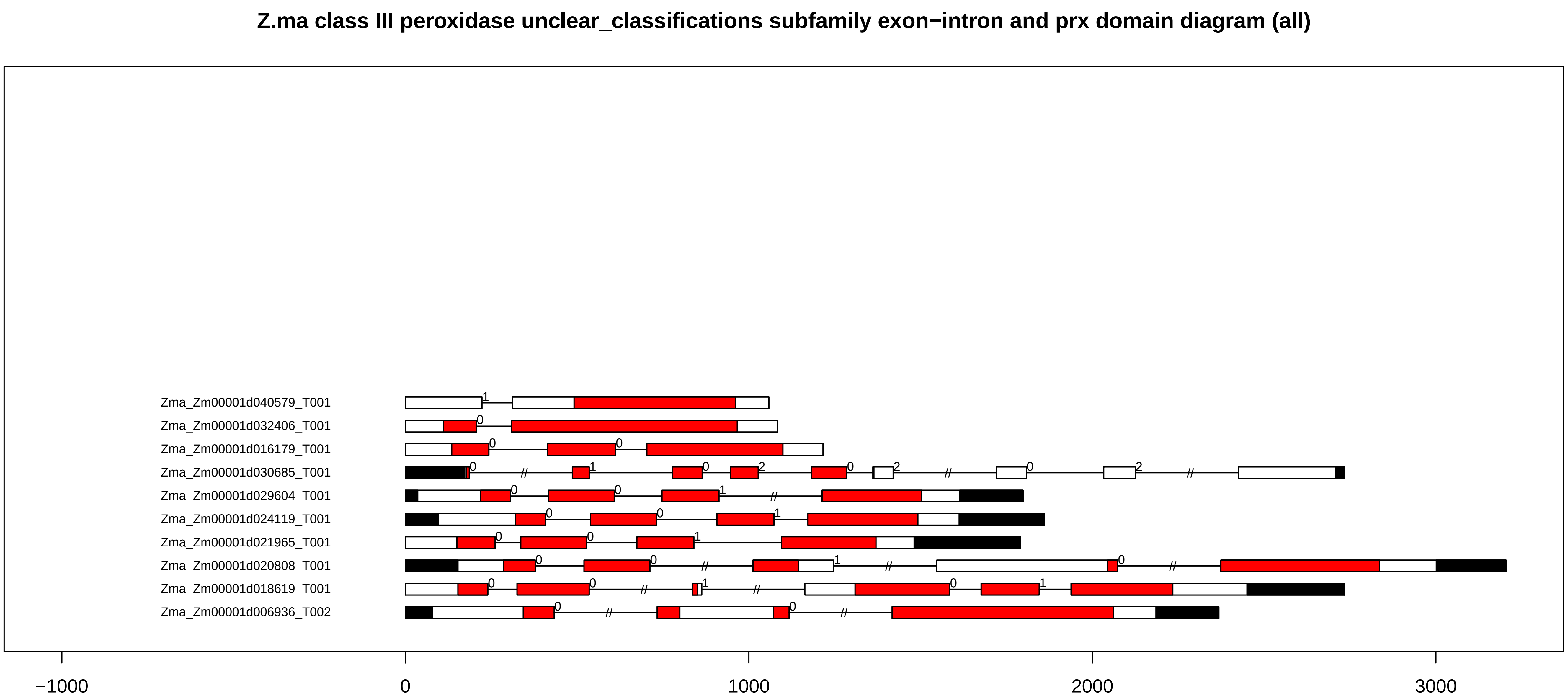

Z.ma class III peroxidase unclear_classifications subfamily exon−intron and prx domain diagram (all)
Zma_Zm00001d040579_T001
Zma_Zm00001d032406_T001
Zma_Zm00001d016179_T001
Zma_Zm00001d030685_T001
Zma_Zm00001d029604_T001
Zma_Zm00001d024119_T001
Zma_Zm00001d021965_T001
Zma_Zm00001d020808_T001
Zma_Zm00001d018619_T001
Zma_Zm00001d006936_T002
−1000
0
1000
2000
3000

A.th class III peroxidase I subfamily exon−intron and prx domain diagram (all)

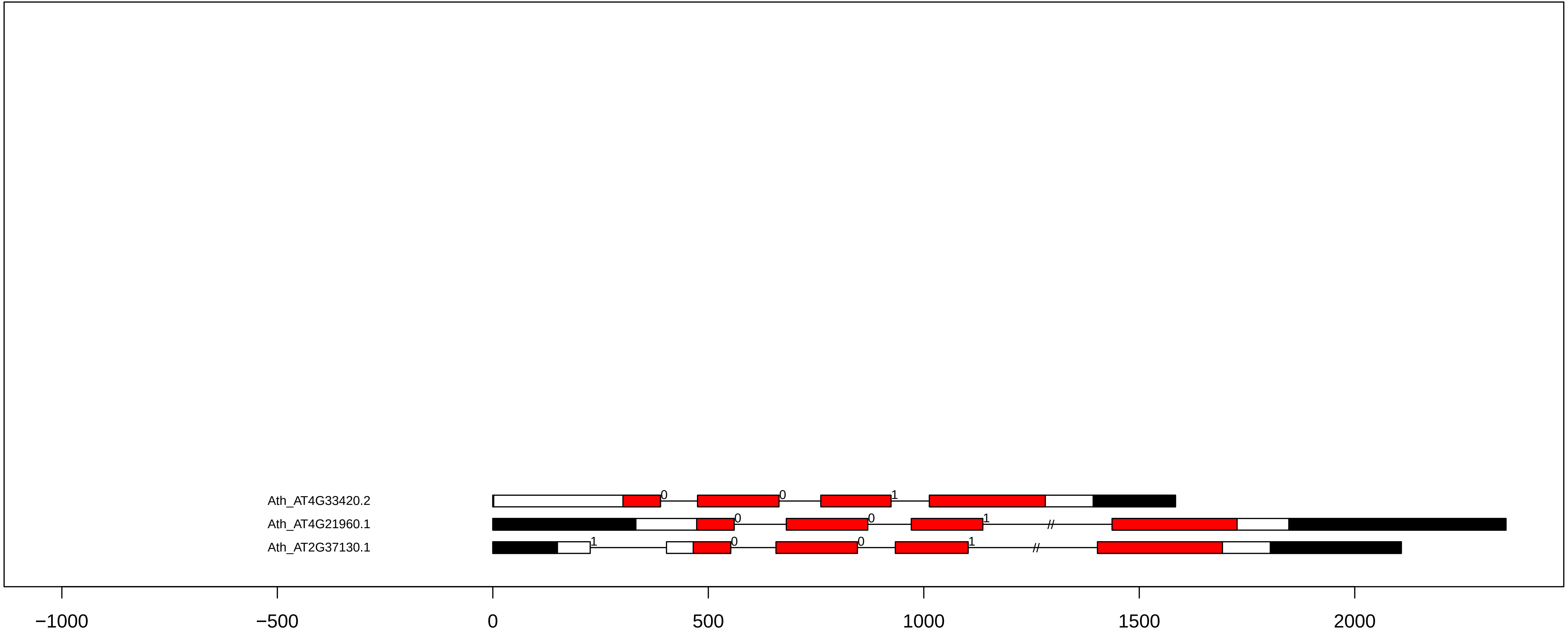

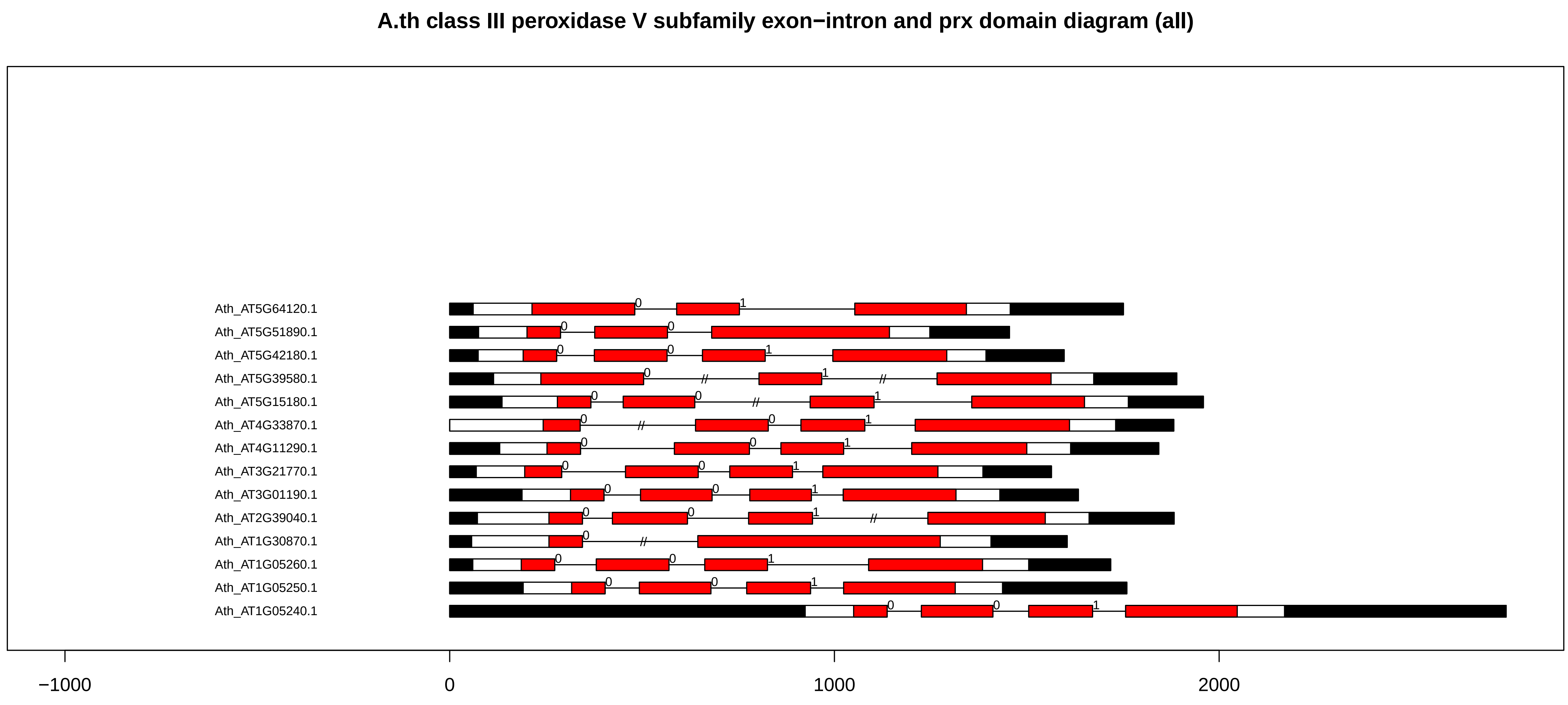
A.th class III peroxidase V subfamily exon−intron and prx domain diagram (all)
Ath_AT5G64120.1
Ath_AT5G51890.1
Ath_AT5G42180.1
Ath_AT5G39580.1
Ath_AT5G15180.1
Ath_AT4G33870.1
Ath_AT4G11290.1
Ath_AT3G21770.1
Ath_AT3G01190.1
Ath_AT2G39040.1
Ath_AT1G30870.1
Ath_AT1G05260.1
Ath_AT1G05250.1
Ath_AT1G05240.1
−1000
0
1000
2000

A.th class III peroxidase VI subfamily exon−intron and prx domain diagram (all)

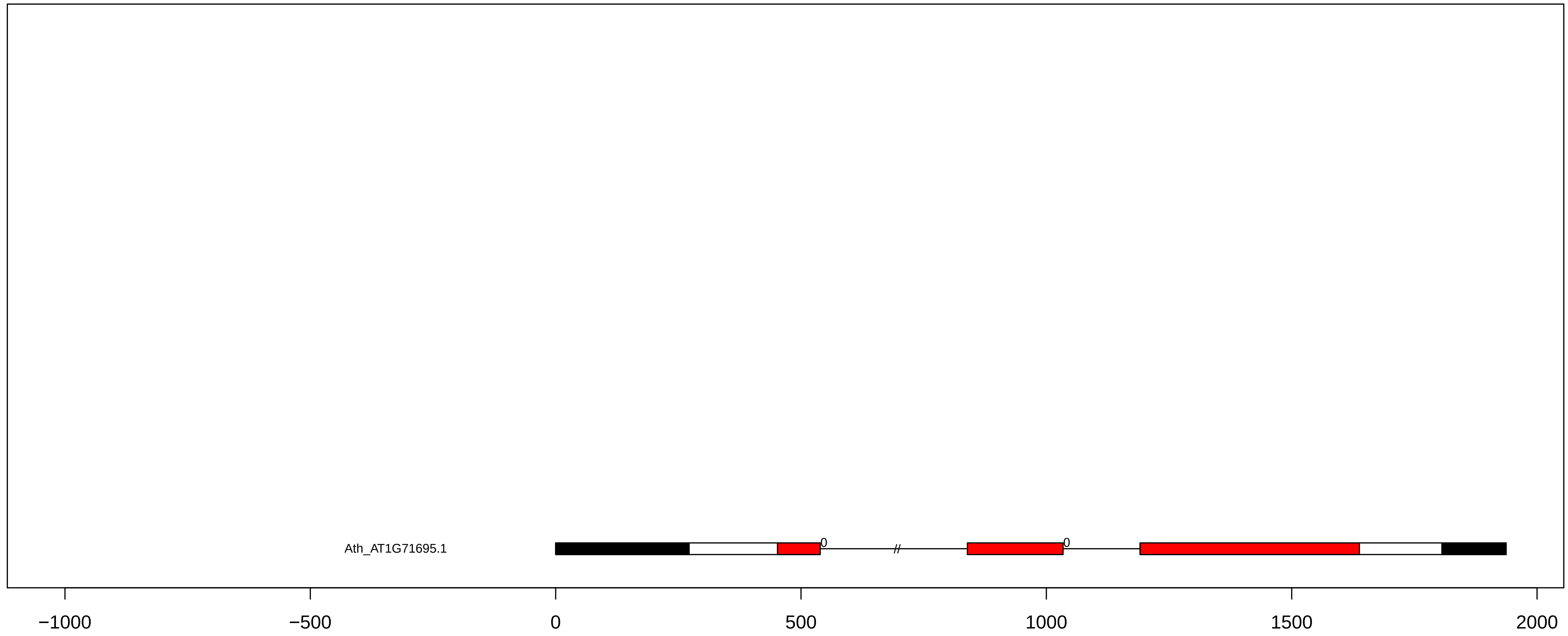

A.th class III peroxidase VII subfamily exon−intron and prx domain diagram (all)

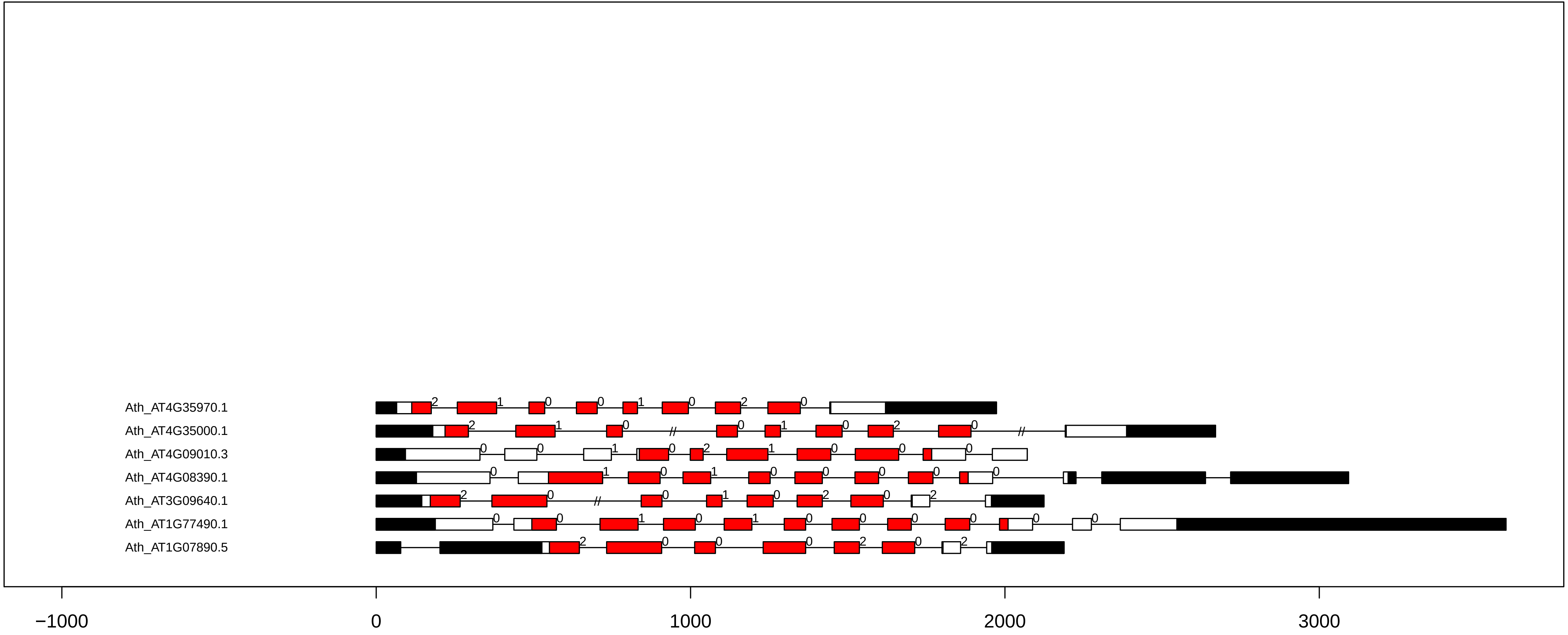

A.th class III peroxidase IX subfamily exon−intron and prx domain diagram (all)

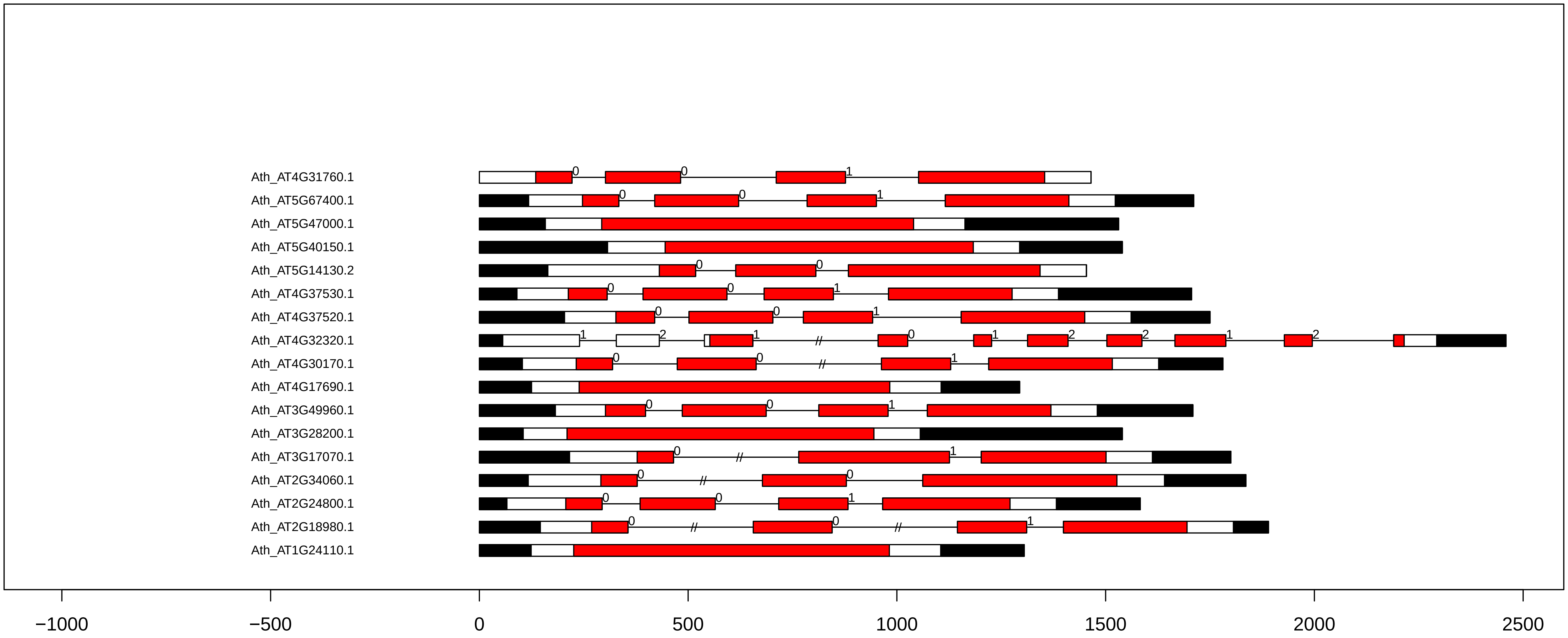

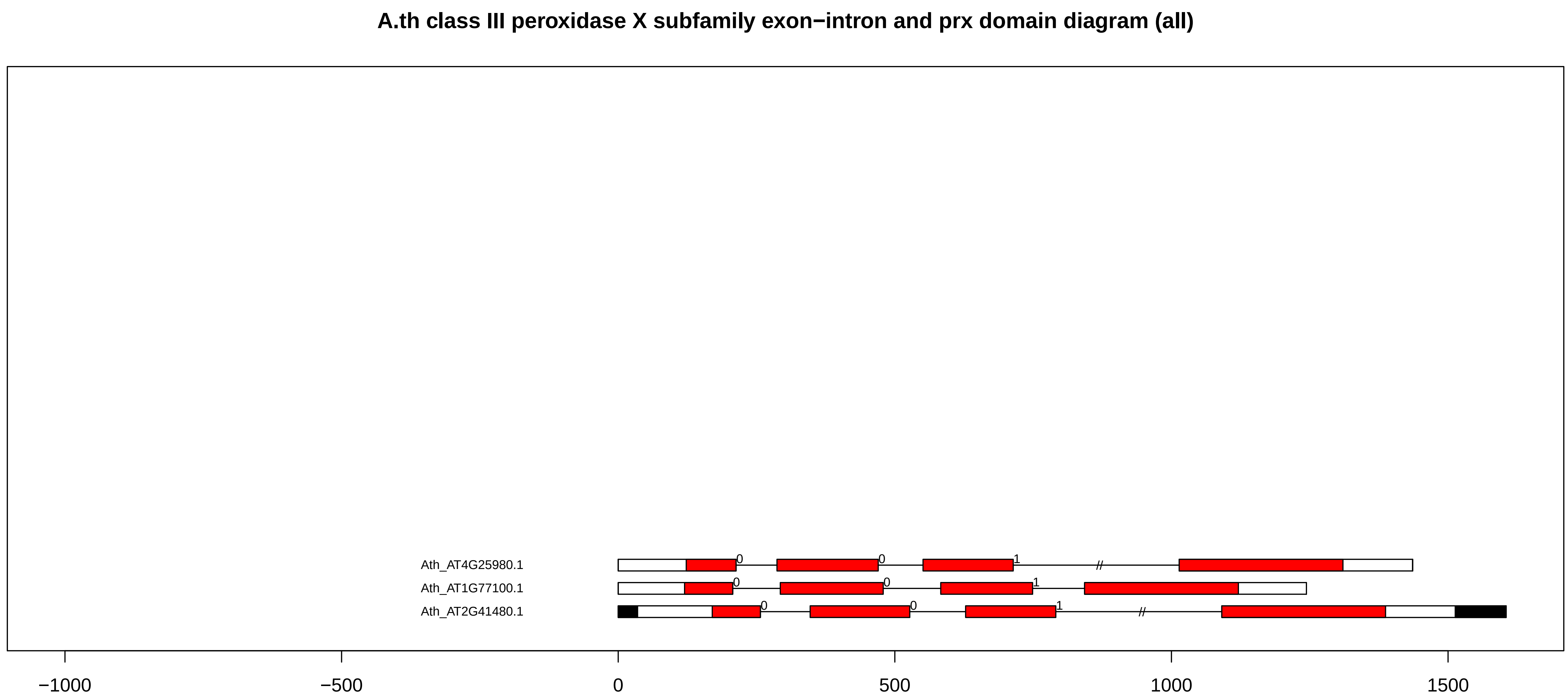
A.th class III peroxidase X subfamily exon−intron and prx domain diagram (all)
Ath_AT4G25980.1
Ath_AT1G77100.1
Ath_AT2G41480.1
0
0
1
//
−1000
−500
0
500
1000
1500

A.th class III peroxidase XI subfamily exon−intron and prx domain diagram (all)

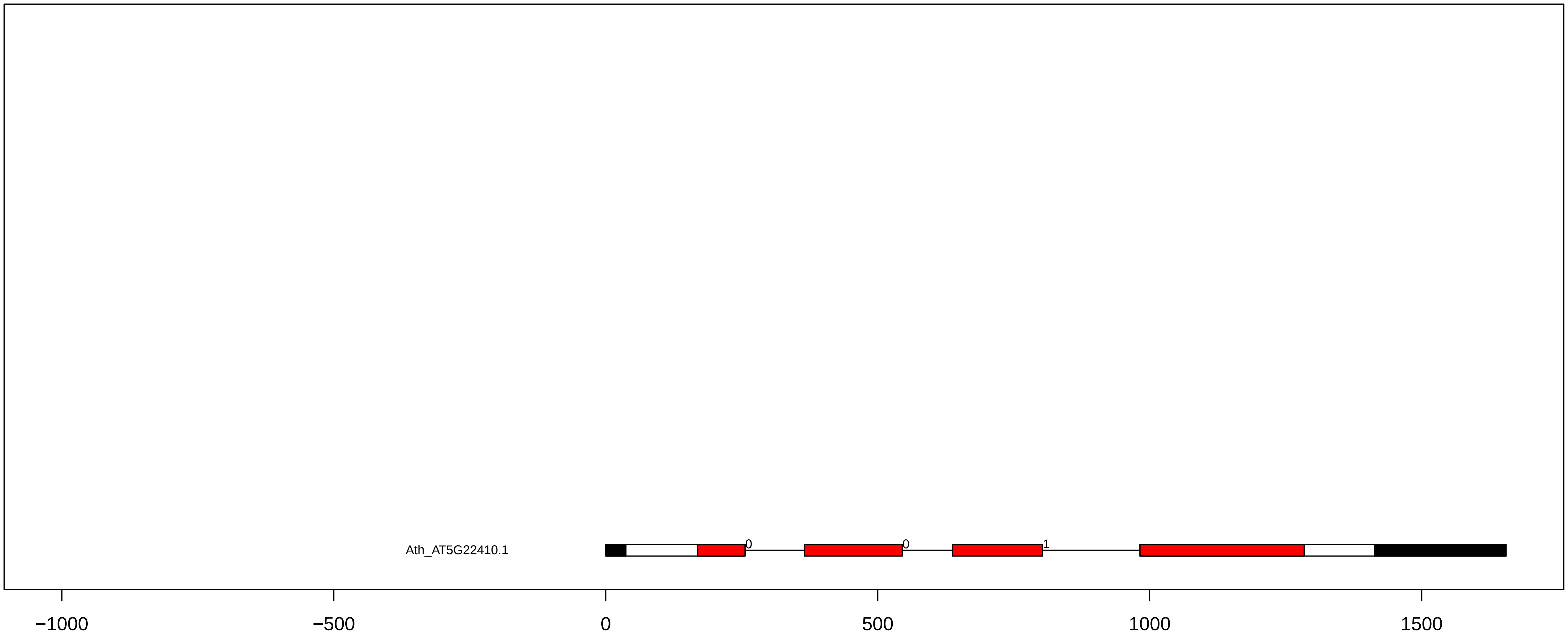

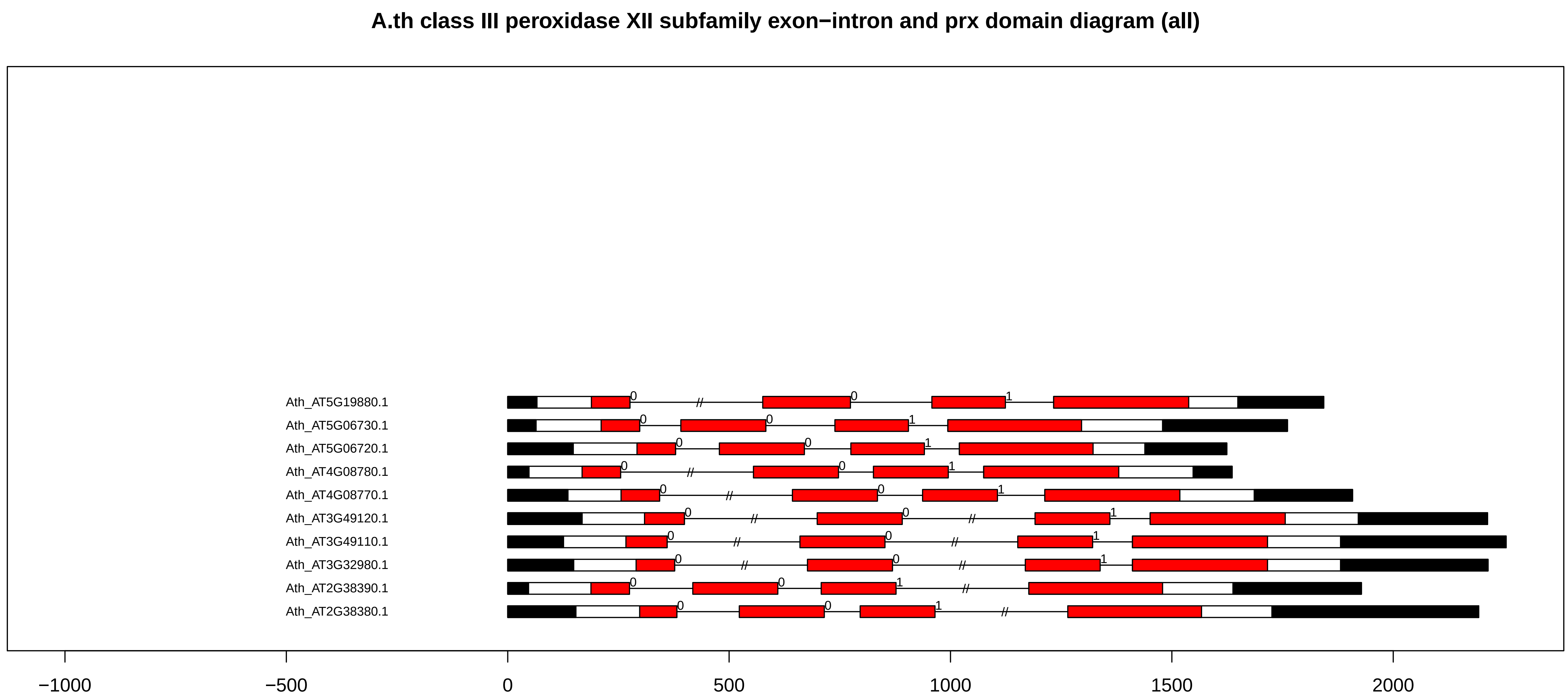
A.th class III peroxidase XII subfamily exon−intron and prx domain diagram (all)
Ath_AT5G19880.1
Ath_AT5G06730.1
Ath_AT5G06720.1
Ath_AT4G08780.1
Ath_AT4G08770.1
Ath_AT3G49120.1
Ath_AT3G49110.1
Ath_AT3G32980.1
Ath_AT2G38390.1
Ath_AT2G38380.1
−1000
−500
0
500
1000
1500
2000

A.th class III peroxidase XIII subfamily exon−intron and prx domain diagram (all)

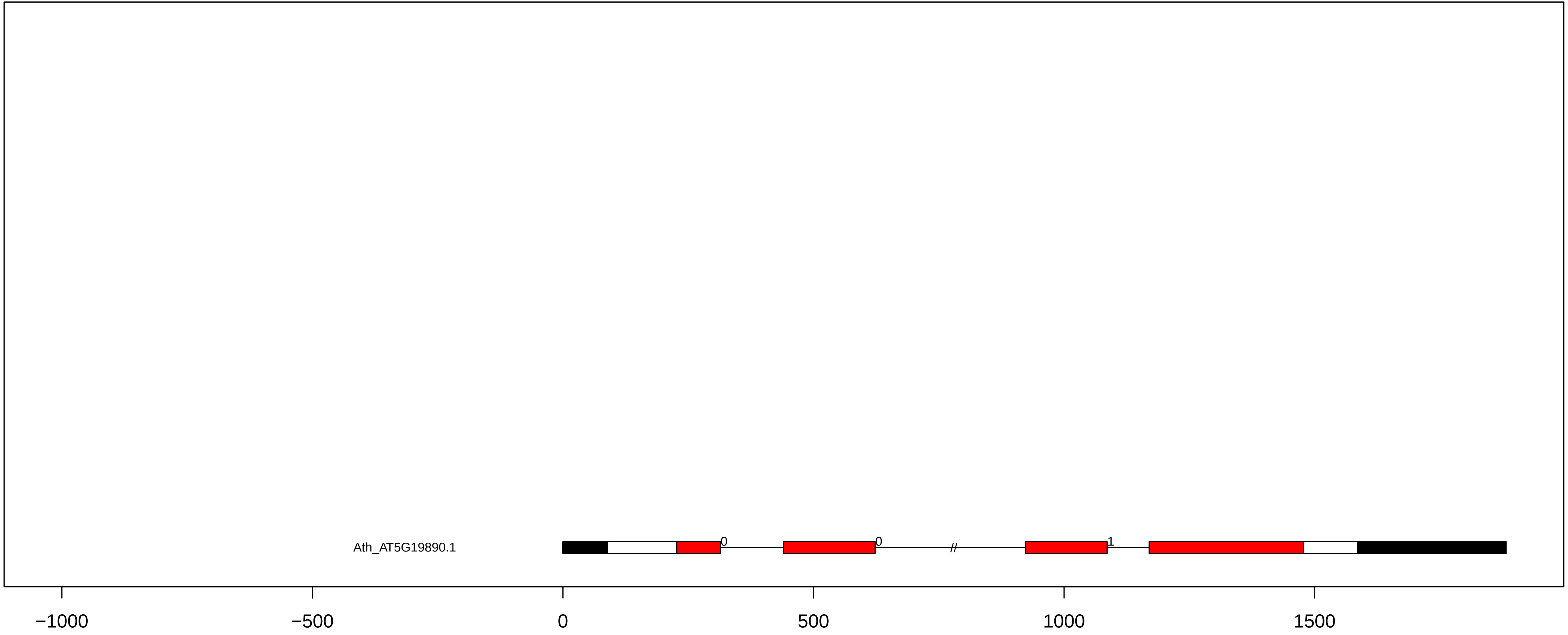

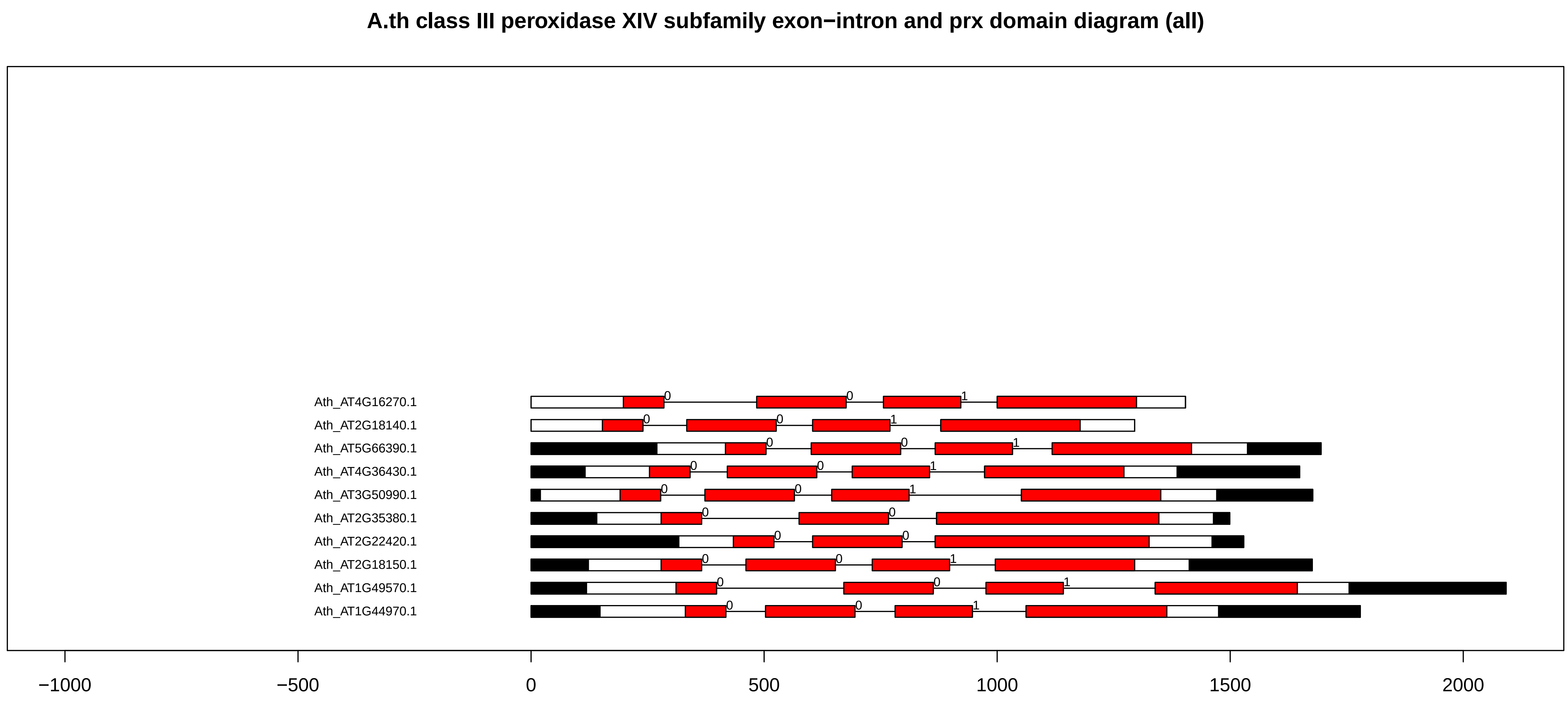
A.th class III peroxidase XIV subfamily exon−intron and prx domain diagram (all)
Ath_AT4G16270.1
Ath_AT2G18140.1
Ath_AT5G66390.1
Ath_AT4G36430.1
Ath_AT3G50990.1
Ath_AT2G35380.1
Ath_AT2G22420.1
Ath_AT2G18150.1
Ath_AT1G49570.1
Ath_AT1G44970.1
−1000
−500
0
500
1000
1500
2000

A.th class III peroxidase XVII subfamily exon−intron and prx domain diagram (all)

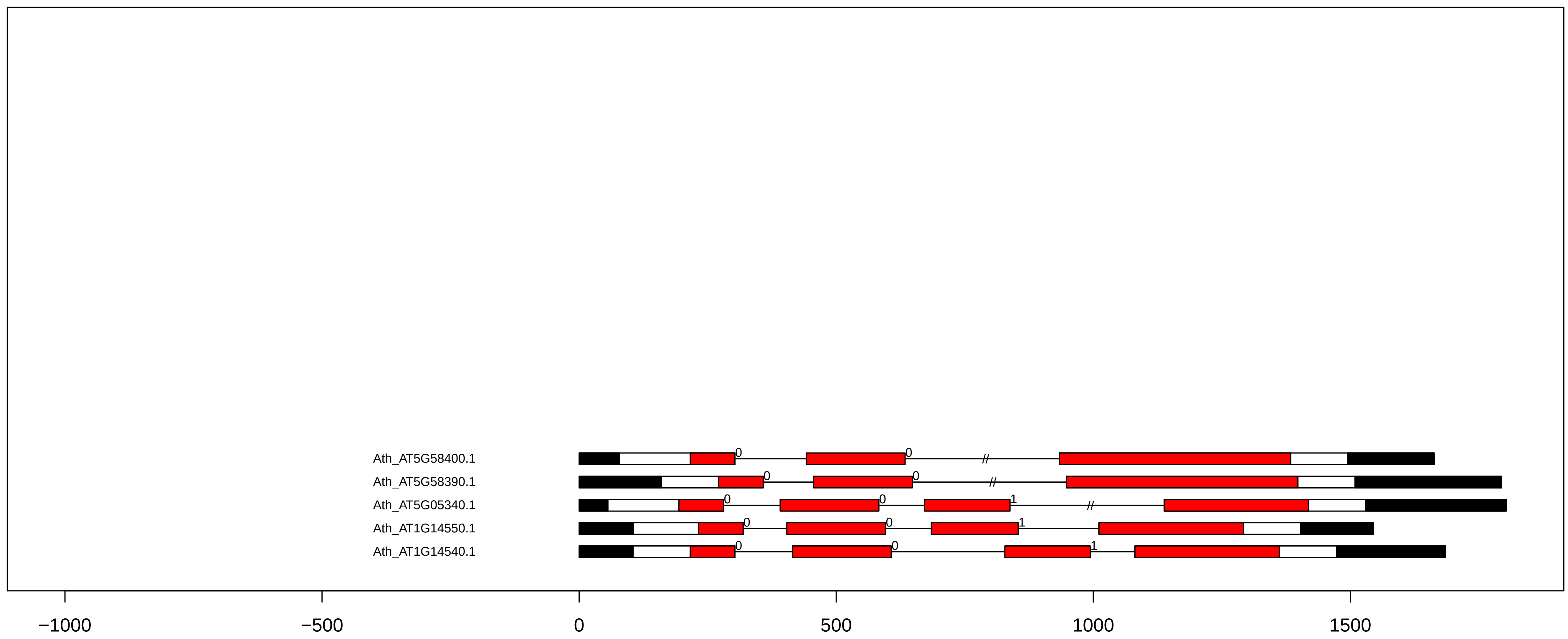

A.th class III peroxidase XVIII subfamily exon−intron and prx domain diagram (all)

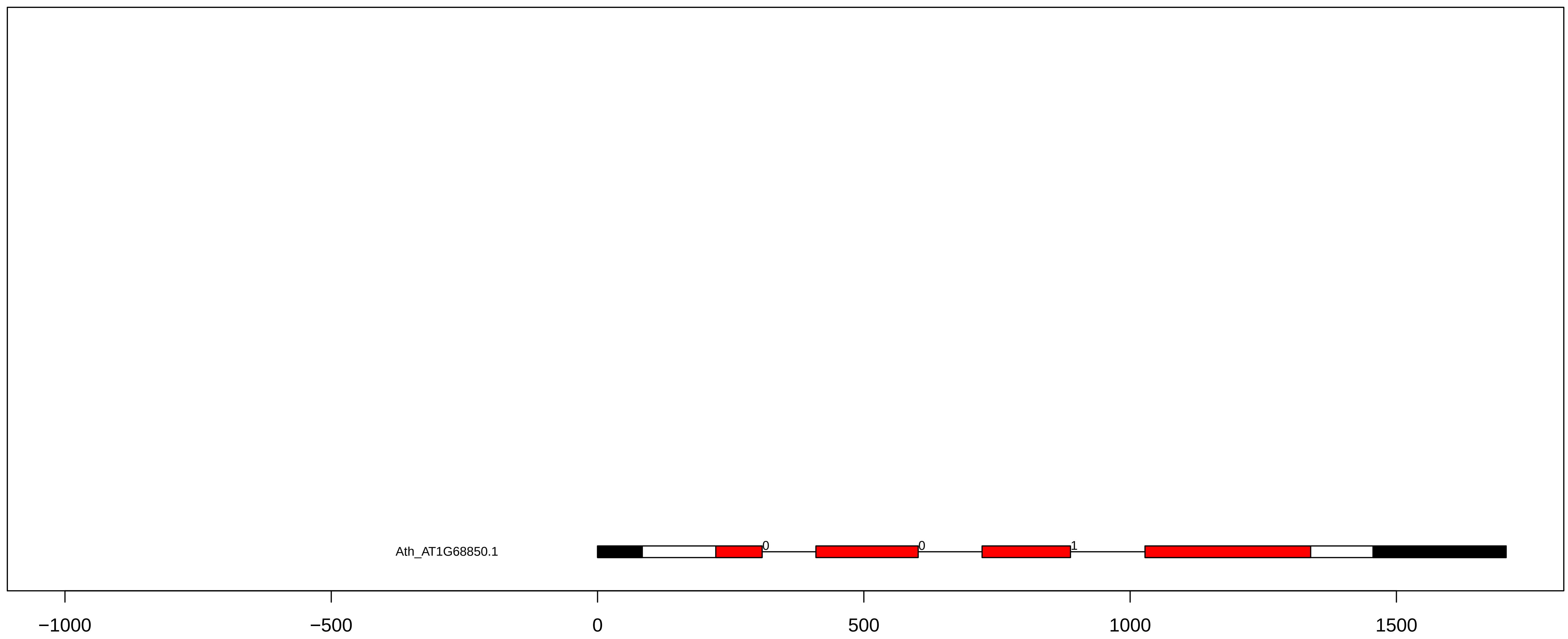

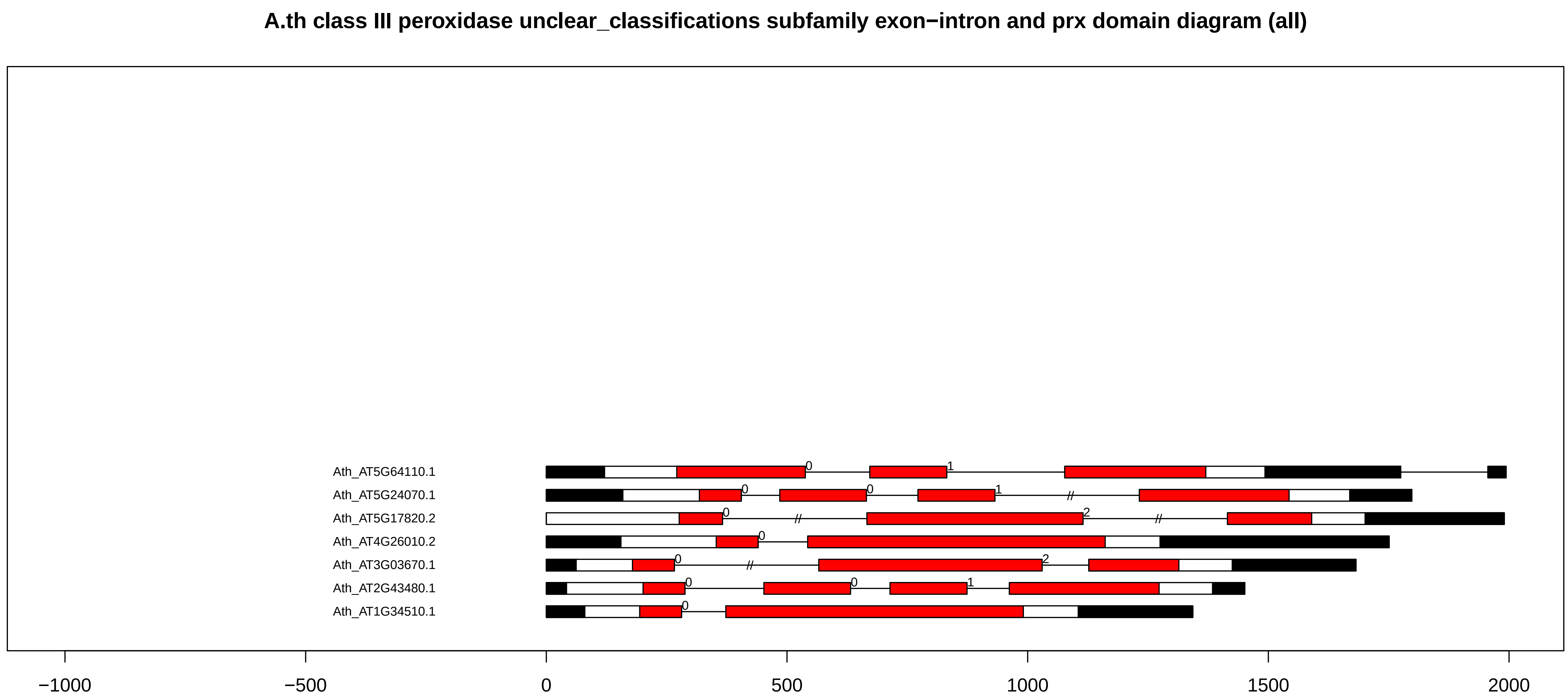
A.th class III peroxidase unclear_classifications subfamily exon−intron and prx domain diagram (all)
Ath_AT5G64110.1
Ath_AT5G24070.1
Ath_AT5G17820.2
Ath_AT4G26010.2
Ath_AT3G03670.1
Ath_AT2G43480.1
Ath_AT1G34510.1
−1000
−500
0
500
1000
1500
2000

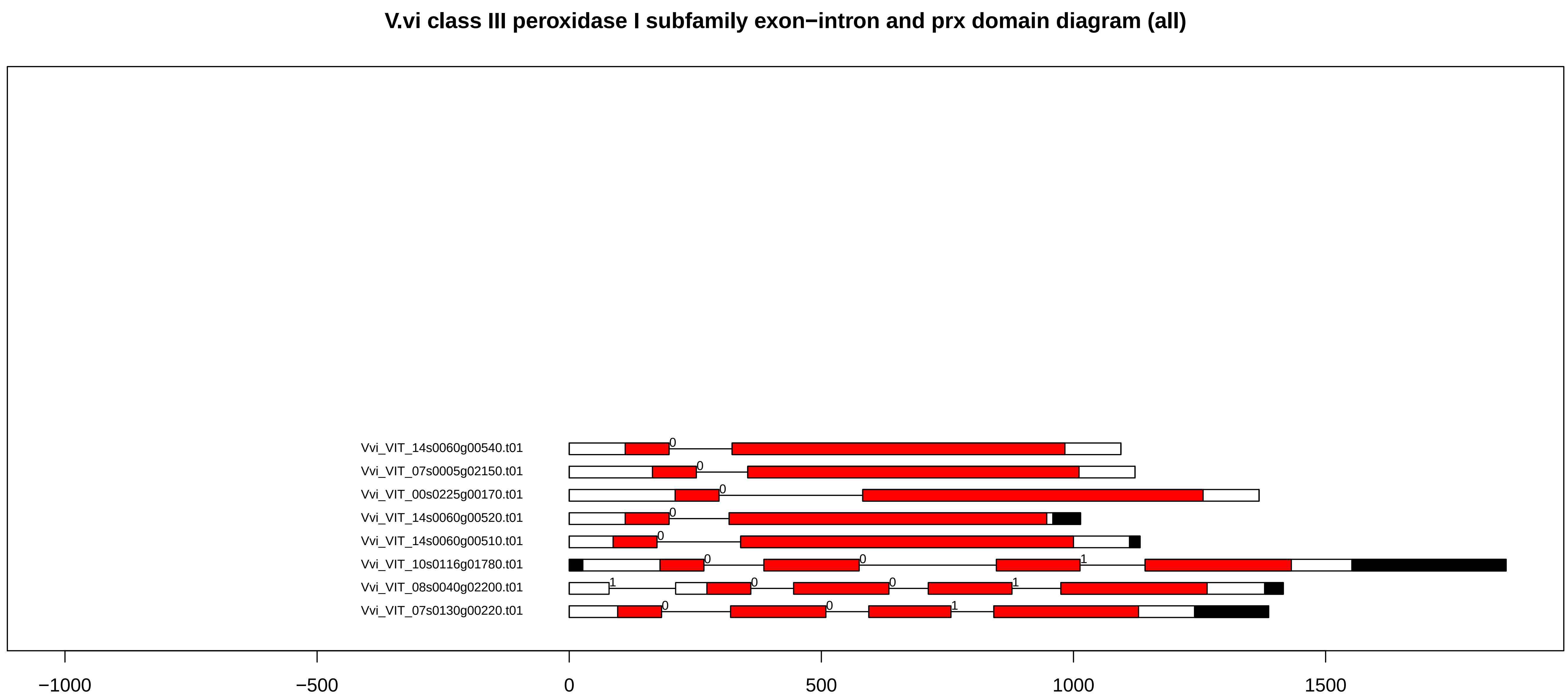
V.vi class III peroxidase I subfamily exon−intron and prx domain diagram (all)
Vvi_VIT_14s0060g00540.t01
Vvi_VIT_07s0005g02150.t01
Vvi_VIT_00s0225g00170.t01
Vvi_VIT_14s0060g00520.t01
Vvi_VIT_14s0060g00510.t01
Vvi_VIT_10s0116g01780.t01
Vvi_VIT_08s0040g02200.t01
Vvi_VIT_07s0130g00220.t01
−1000
−500
0
500
1000
1500

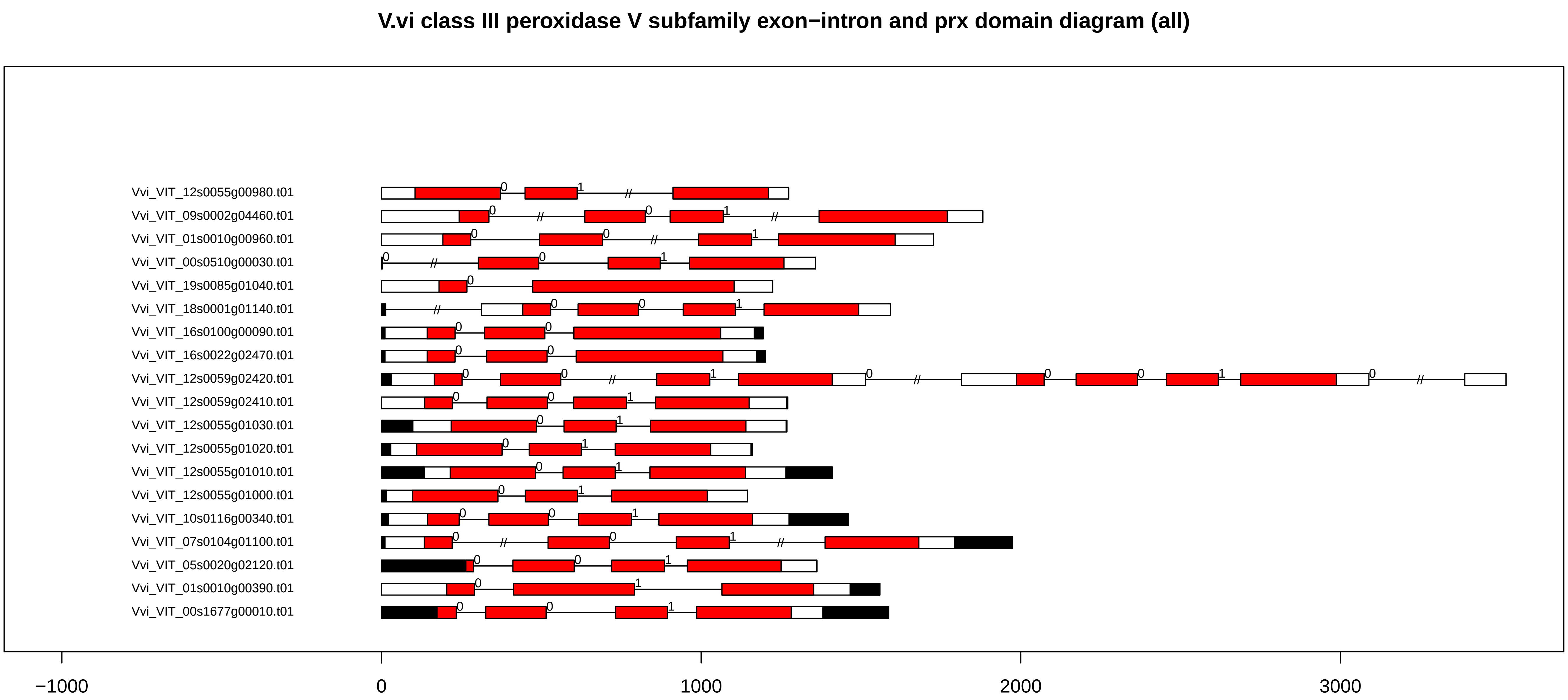
V.vi class III peroxidase V subfamily exon−intron and prx domain diagram (all)
Vvi_VIT_12s0055g00980.t01
Vvi_VIT_09s0002g04460.t01
Vvi_VIT_01s0010g00960.t01
Vvi_VIT_00s0510g00030.t01
Vvi_VIT_19s0085g01040.t01
Vvi_VIT_18s0001g01140.t01
Vvi_VIT_16s0100g00090.t01
Vvi_VIT_16s0022g02470.t01
Vvi_VIT_12s0059g02420.t01
Vvi_VIT_12s0059g02410.t01
Vvi_VIT_12s0055g01030.t01
Vvi_VIT_12s0055g01020.t01
Vvi_VIT_12s0055g01010.t01
Vvi_VIT_12s0055g01000.t01
Vvi_VIT_10s0116g00340.t01
Vvi_VIT_07s0104g01100.t01
Vvi_VIT_05s0020g02120.t01
Vvi_VIT_01s0010g00390.t01
Vvi_VIT_00s1677g00010.t01
−1000
0
1000
2000
3000

V.vi class III peroxidase VI subfamily exon−intron and prx domain diagram (all)

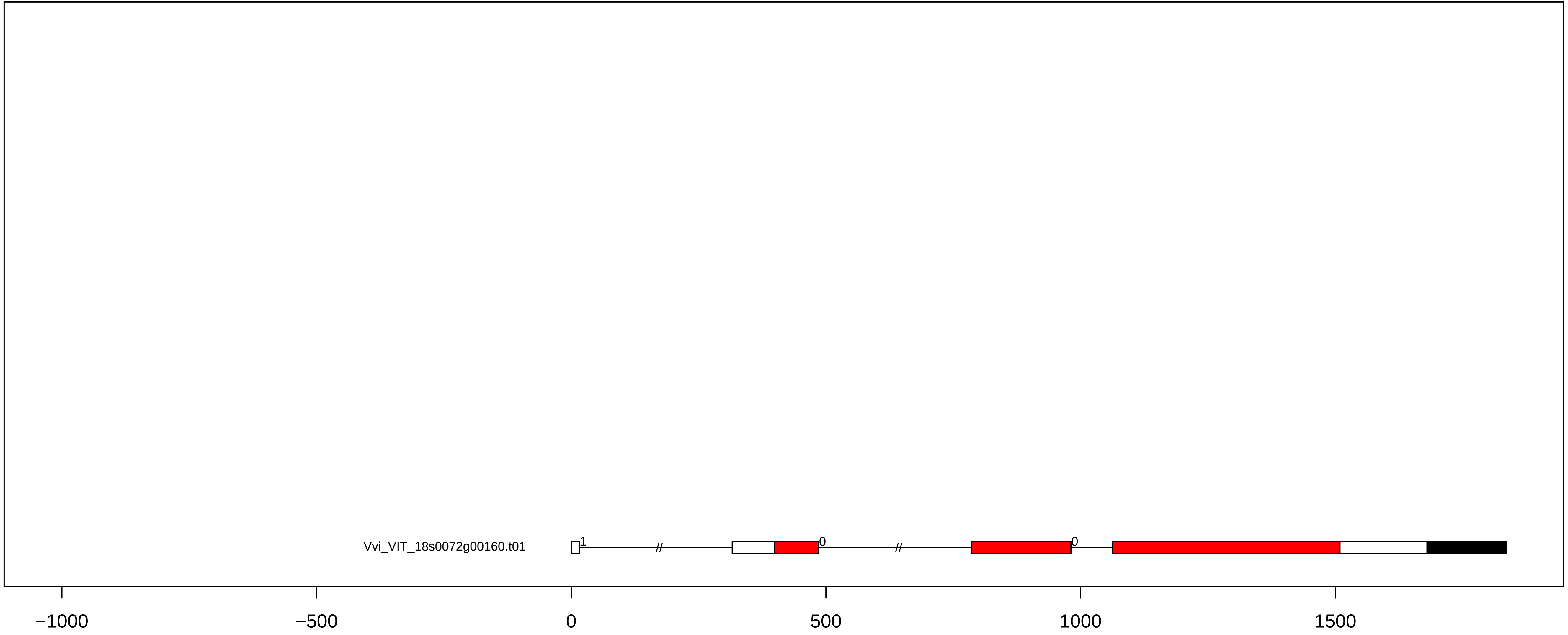

V.vi class III peroxidase VII subfamily exon−intron and prx domain diagram (all)

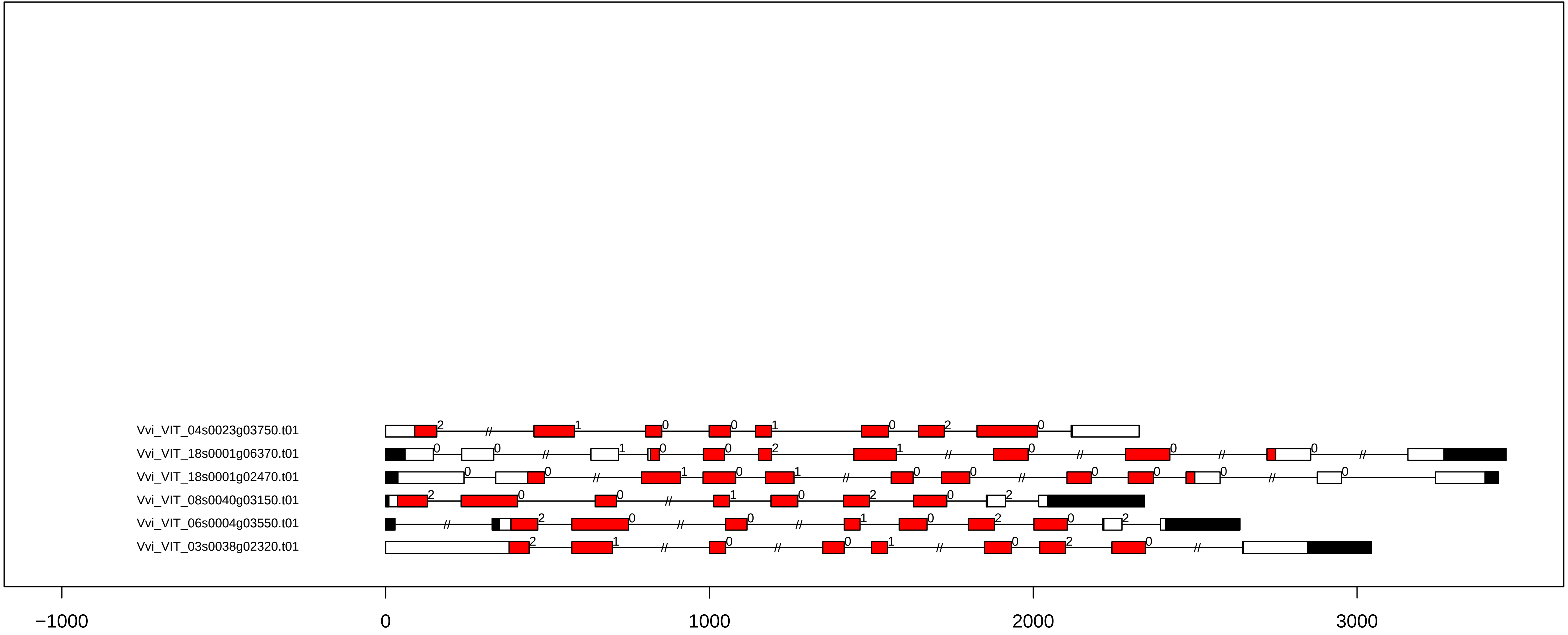

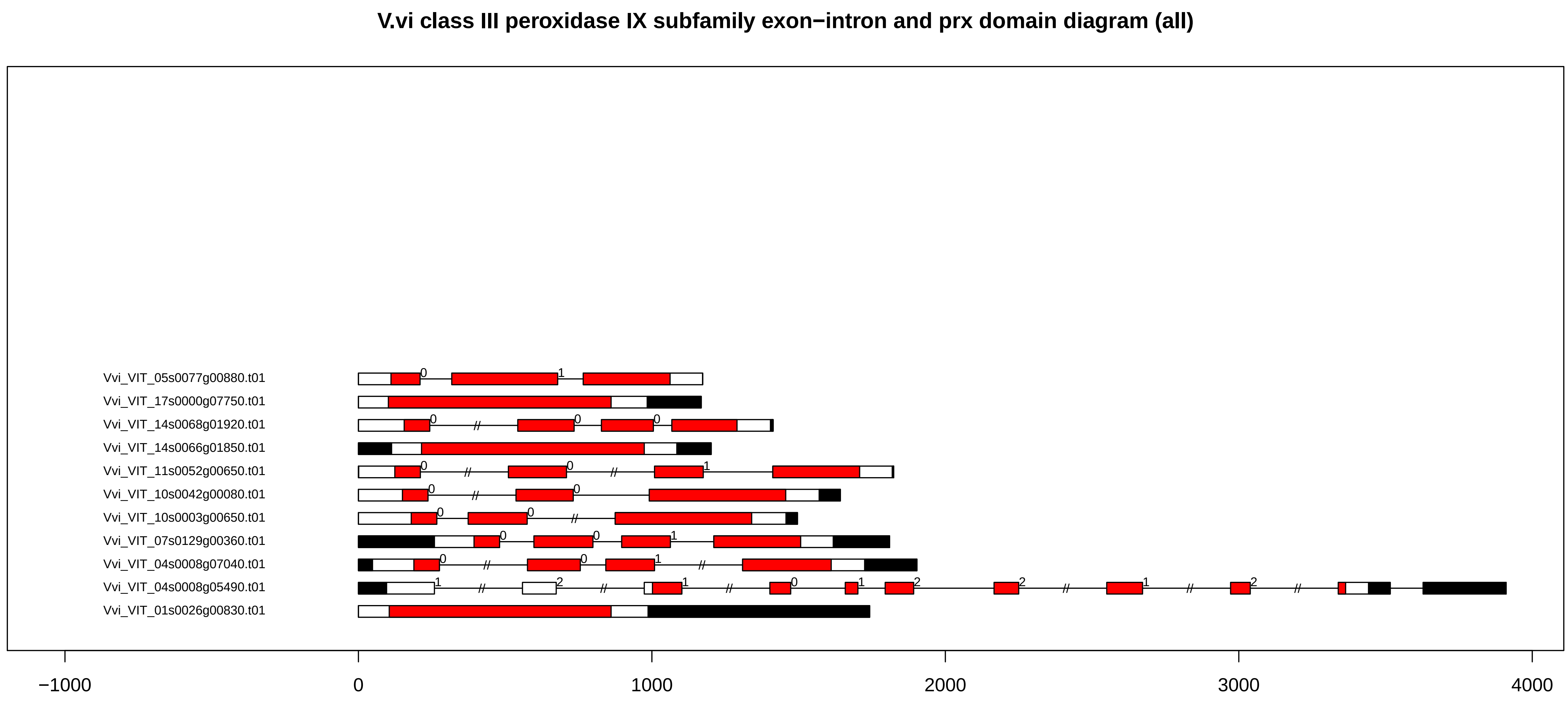
V.vi class III peroxidase IX subfamily exon−intron and prx domain diagram (all)
Vvi_VIT_05s0077g00880.t01
Vvi_VIT_17s0000g07750.t01
Vvi_VIT_14s0068g01920.t01
Vvi_VIT_14s0066g01850.t01
Vvi_VIT_11s0052g00650.t01
Vvi_VIT_10s0042g00080.t01
Vvi_VIT_10s0003g00650.t01
Vvi_VIT_07s0129g00360.t01
Vvi_VIT_04s0008g07040.t01
Vvi_VIT_04s0008g05490.t01
Vvi_VIT_01s0026g00830.t01
−1000
0
1000
2000
3000
4000

V.vi class III peroxidase X subfamily exon−intron and prx domain diagram (all)

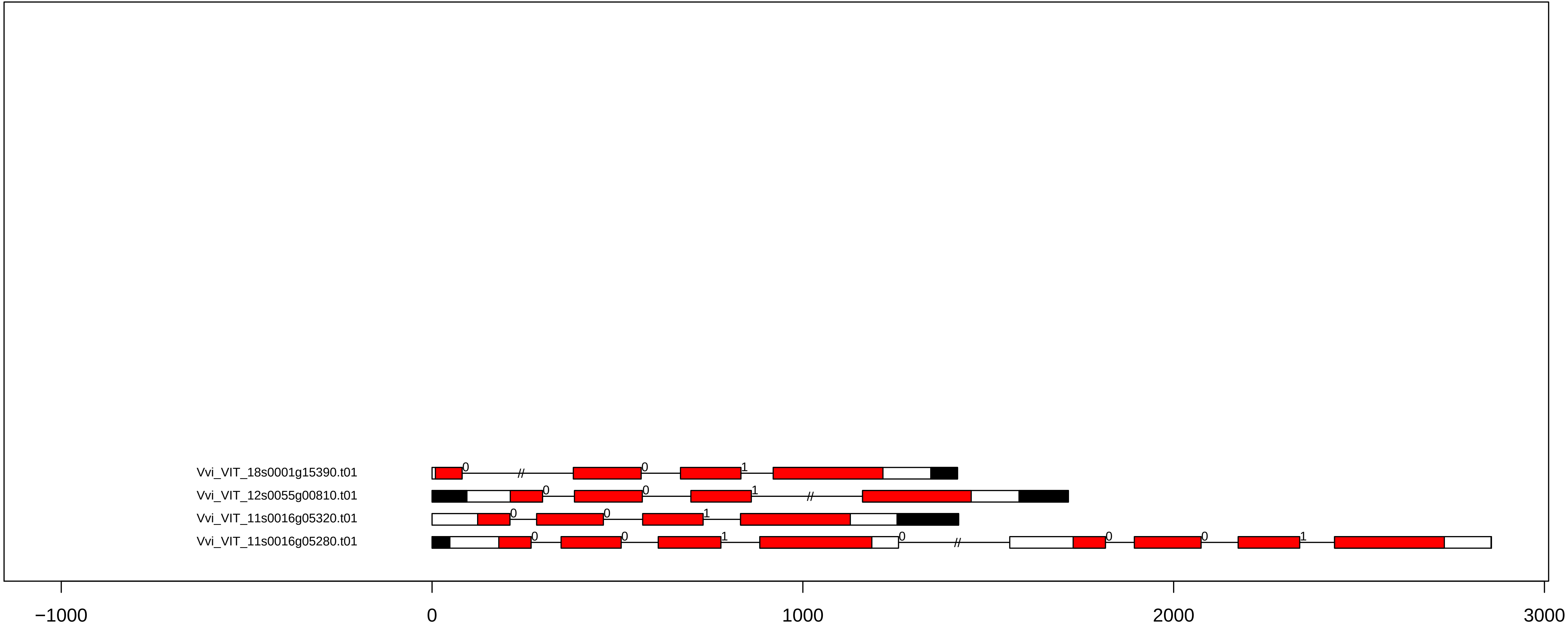

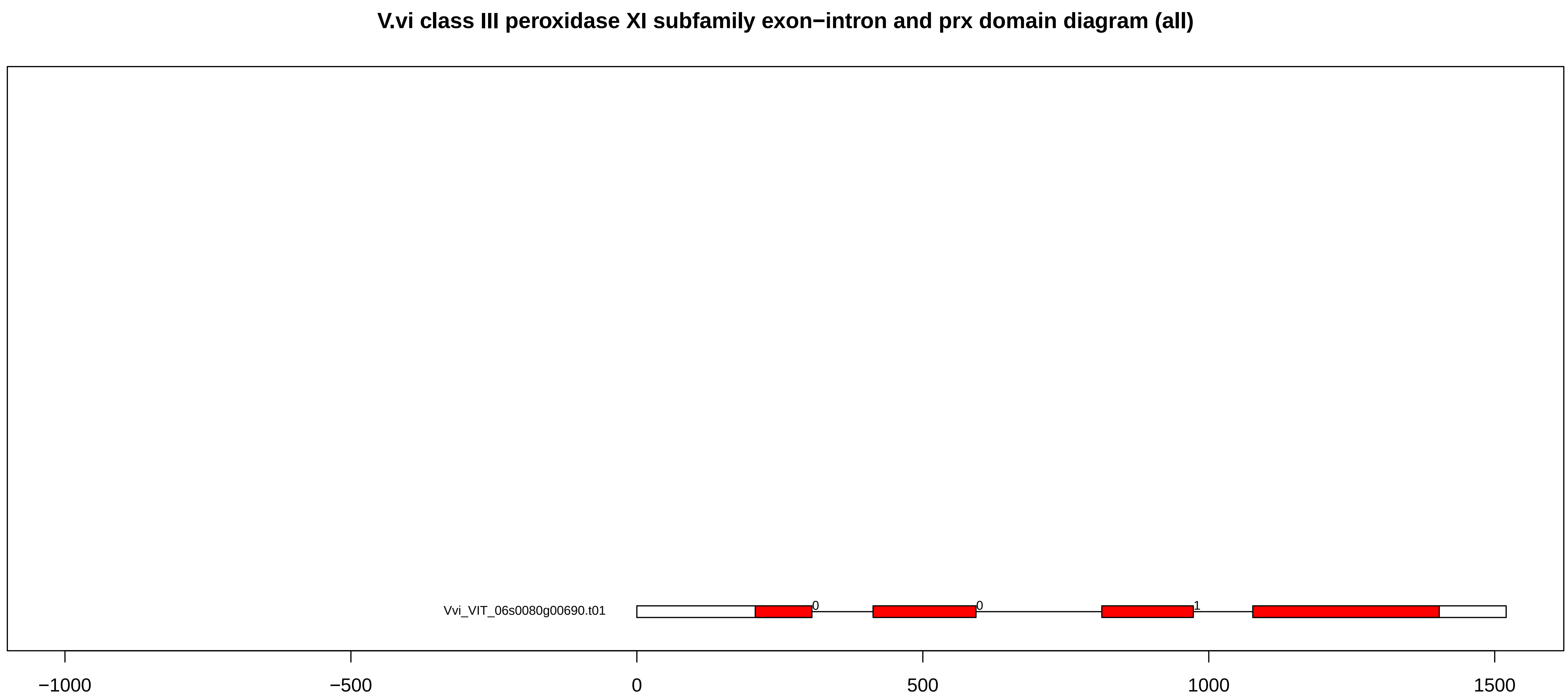
V.vi class III peroxidase XI subfamily exon−intron and prx domain diagram (all)
Vvi_VIT_06s0080g00690.t01
0
0
1
−1000
−500
0
500
1000
1500

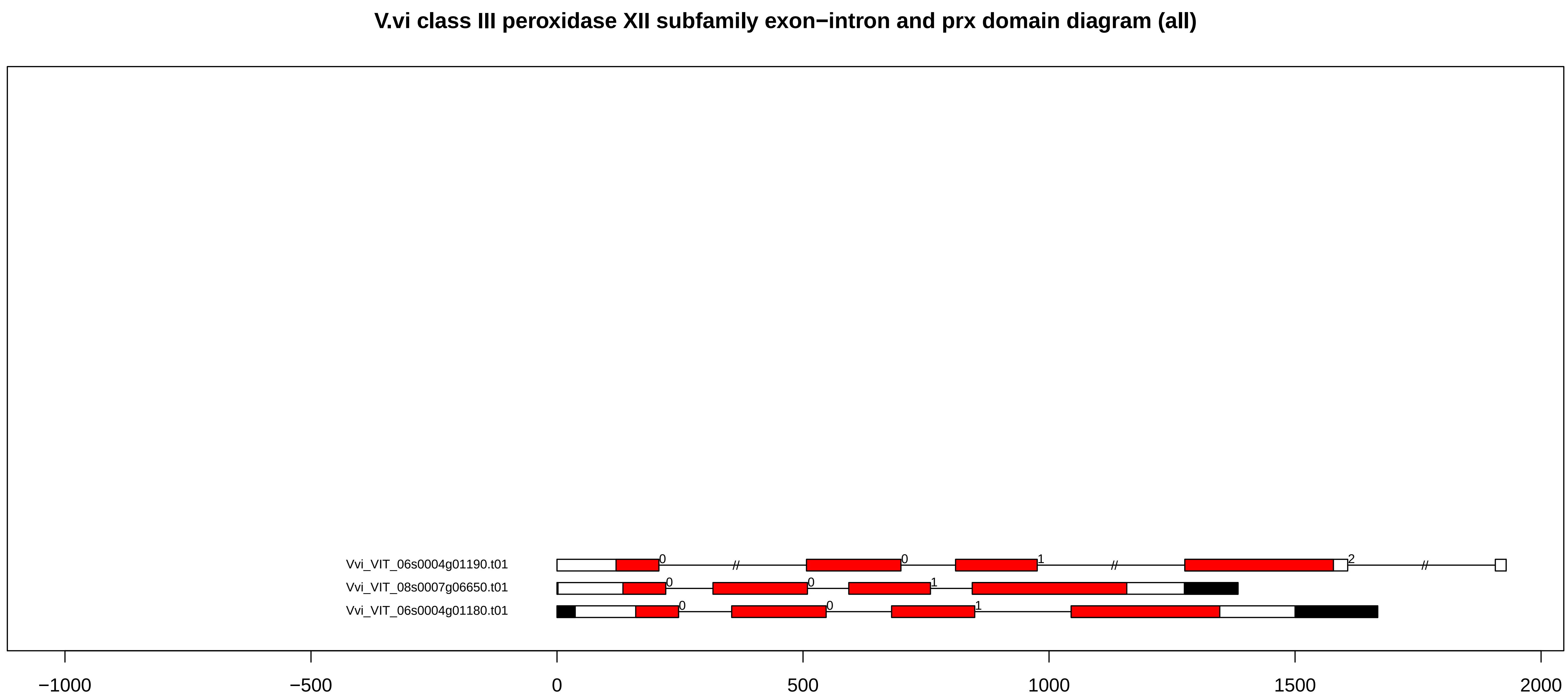
V.vi class III peroxidase XII subfamily exon−intron and prx domain diagram (all)
Vvi_VIT_06s0004g01190.t01
Vvi_VIT_08s0007g06650.t01
Vvi_VIT_06s0004g01180.t01
0
0
1
2
0
0
1
0
0
1
−1000
−500
0
500
1000
1500
2000

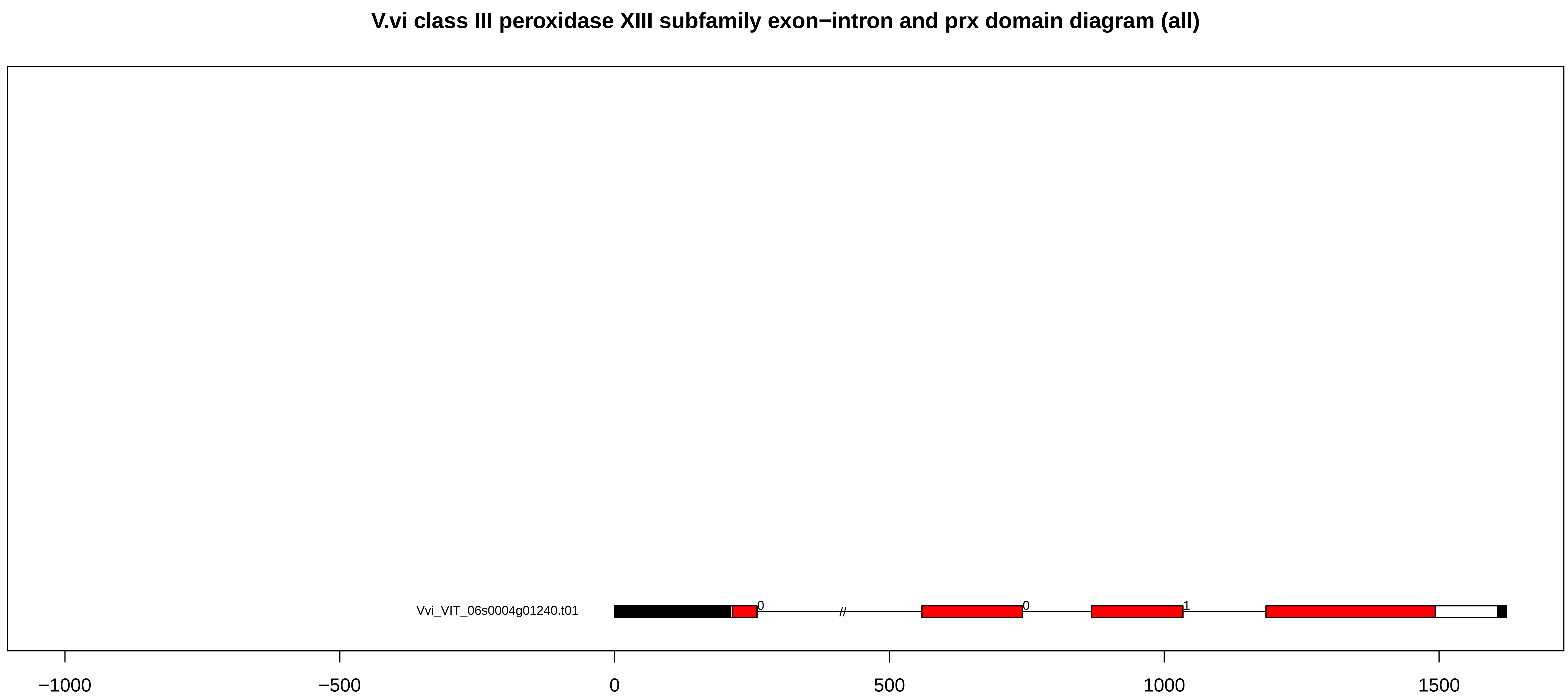
V.vi class III peroxidase XIII subfamily exon−intron and prx domain diagram (all)
Vvi_VIT_06s0004g01240.t01
0
0
1
−1000
−500
0
500
1000
1500

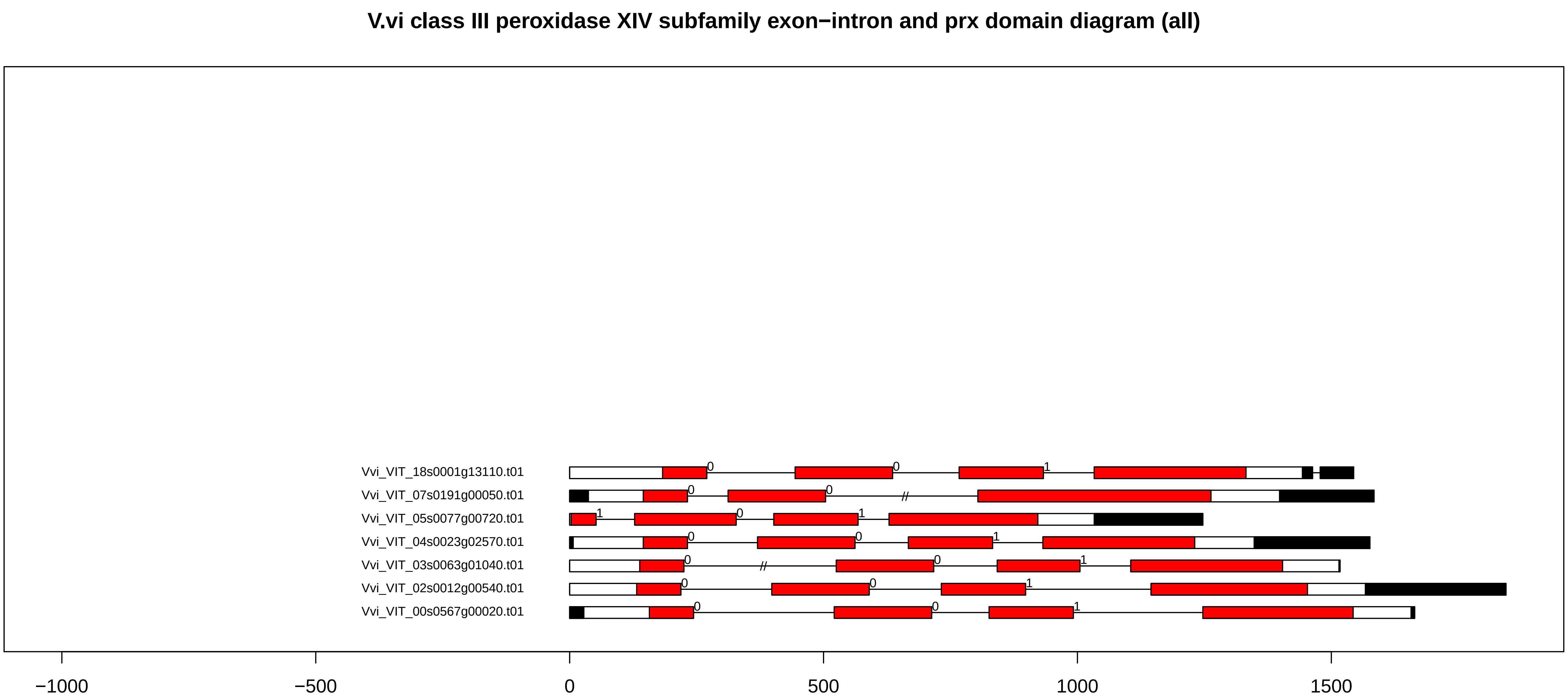
V.vi class III peroxidase XIV subfamily exon−intron and prx domain diagram (all)
Vvi_VIT_18s0001g13110.t01
Vvi_VIT_07s0191g00050.t01
Vvi_VIT_05s0077g00720.t01
Vvi_VIT_04s0023g02570.t01
Vvi_VIT_03s0063g01040.t01
Vvi_VIT_02s0012g00540.t01
Vvi_VIT_00s0567g00020.t01
−1000
−500
0
500
1000
1500

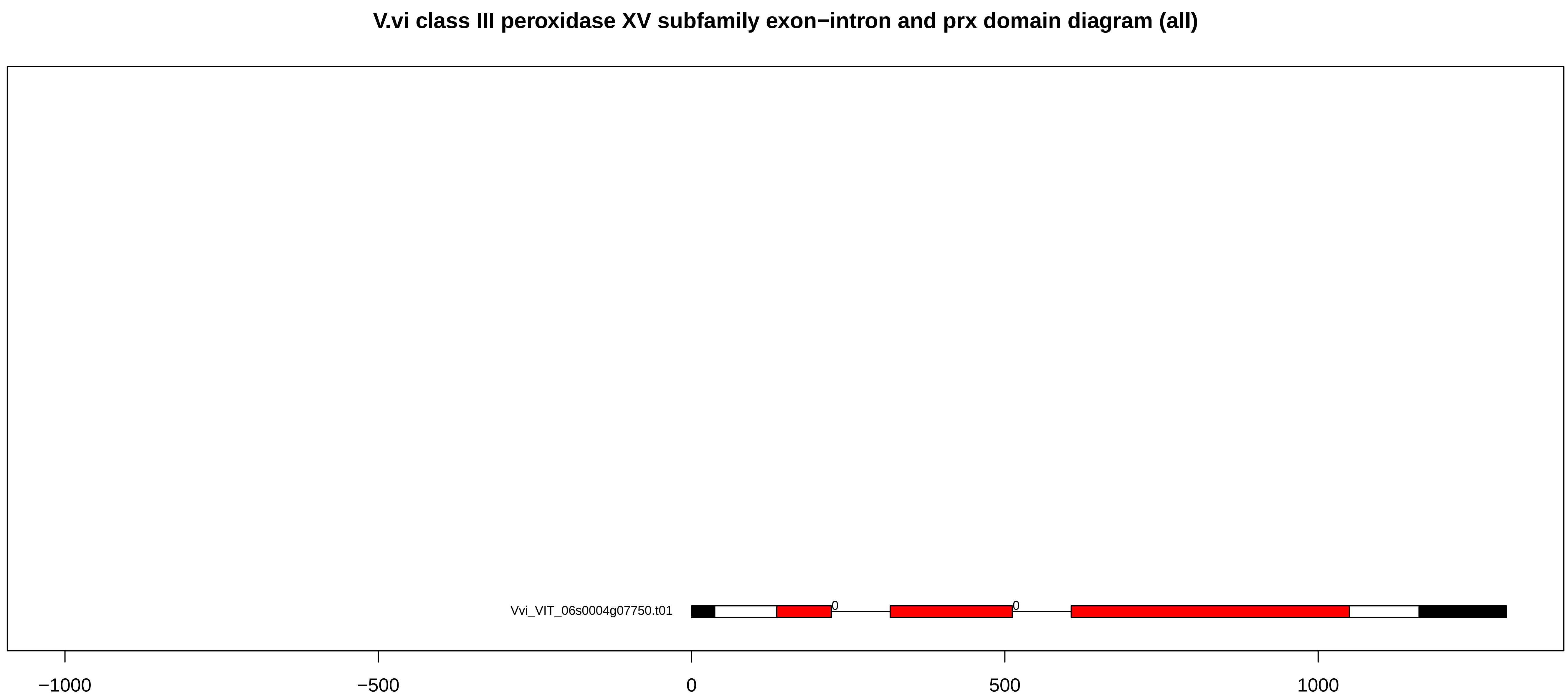
V.vi class III peroxidase XV subfamily exon−intron and prx domain diagram (all)
Vvi_VIT_06s0004g07750.t01
0
0
−1000
−500
0
500
1000

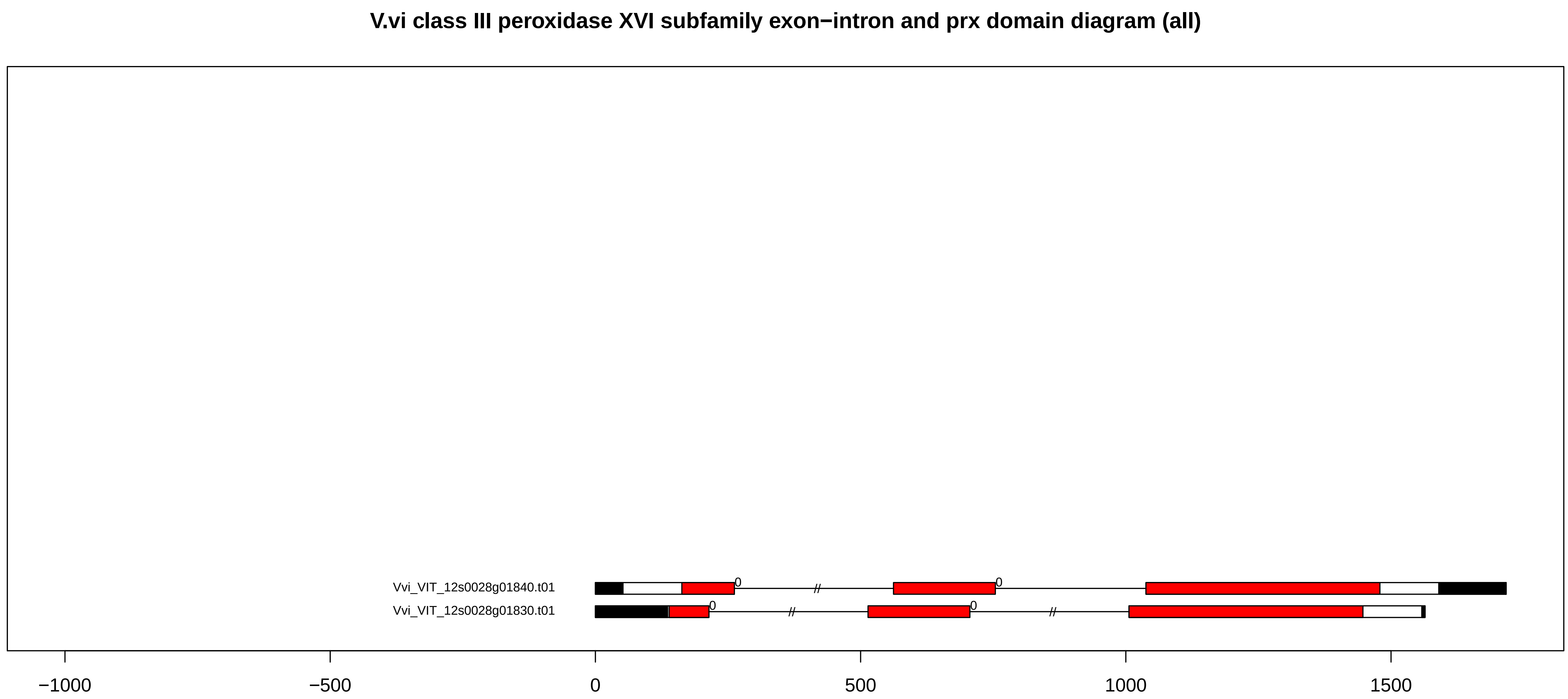

V.vi class III peroxidase XVI subfamily exon−intron and prx domain diagram (all)
Vvi_VIT_12s0028g01840.t01
Vvi_VIT_12s0028g01830.t01
0
0
0
0
−1000
−500
0
500
1000
1500

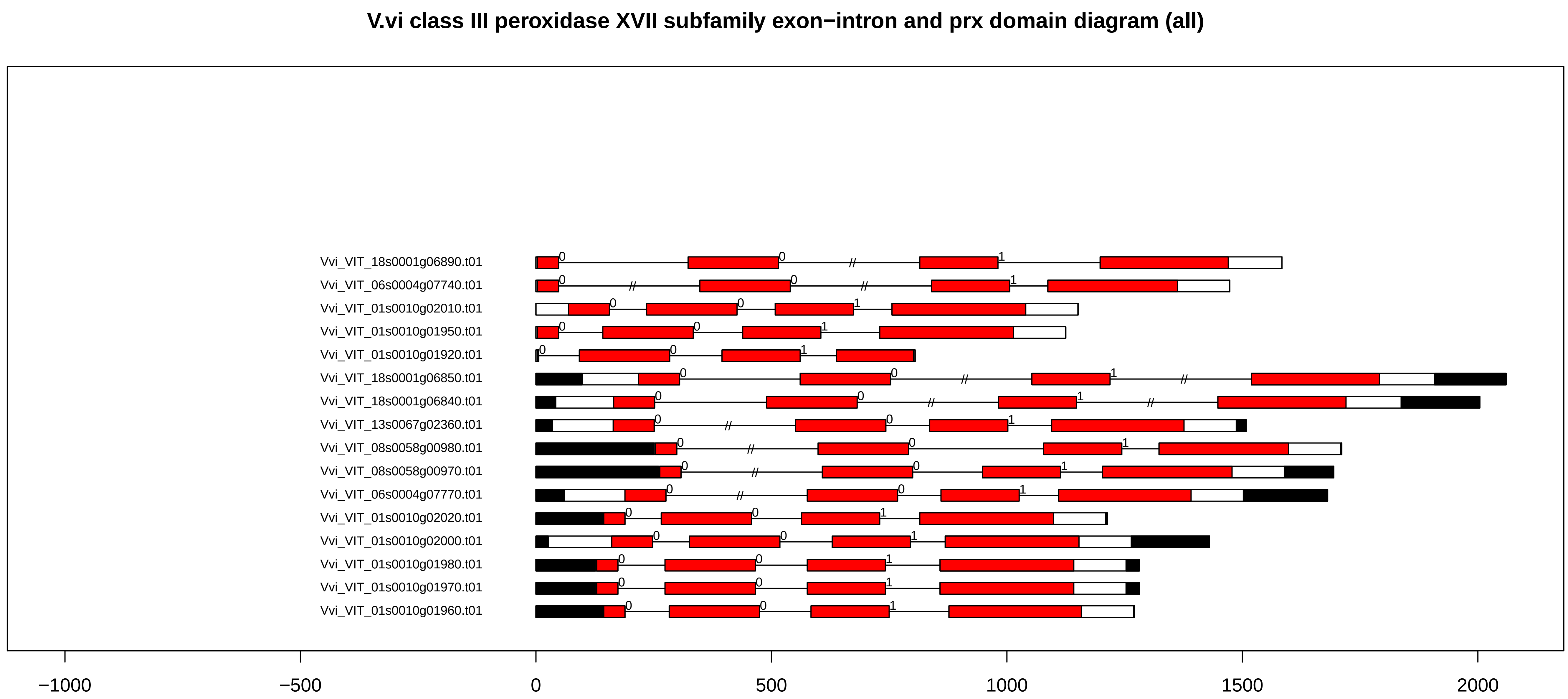

V.vi class III peroxidase XVII subfamily exon−intron and prx domain diagram (all)
Vvi_VIT_18s0001g06890.t01
Vvi_VIT_06s0004g07740.t01
Vvi_VIT_01s0010g02010.t01
Vvi_VIT_01s0010g01950.t01
Vvi_VIT_01s0010g01920.t01
Vvi_VIT_18s0001g06850.t01
Vvi_VIT_18s0001g06840.t01
Vvi_VIT_13s0067g02360.t01
Vvi_VIT_08s0058g00980.t01
Vvi_VIT_08s0058g00970.t01
Vvi_VIT_06s0004g07770.t01
Vvi_VIT_01s0010g02020.t01
Vvi_VIT_01s0010g02000.t01
Vvi_VIT_01s0010g01980.t01
Vvi_VIT_01s0010g01970.t01
Vvi_VIT_01s0010g01960.t01
−1000
−500
0
500
1000
1500
2000

V.vi class III peroxidase XVIII subfamily exon−intron and prx domain diagram (all)

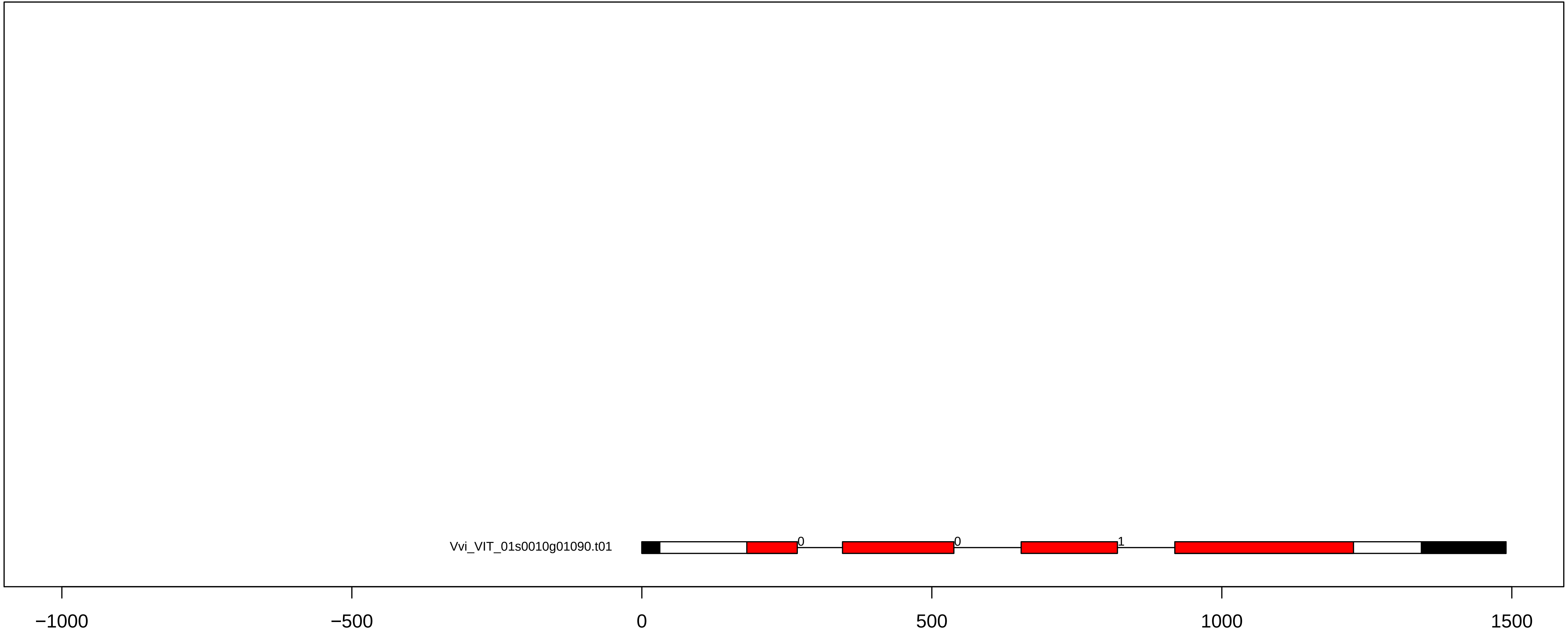

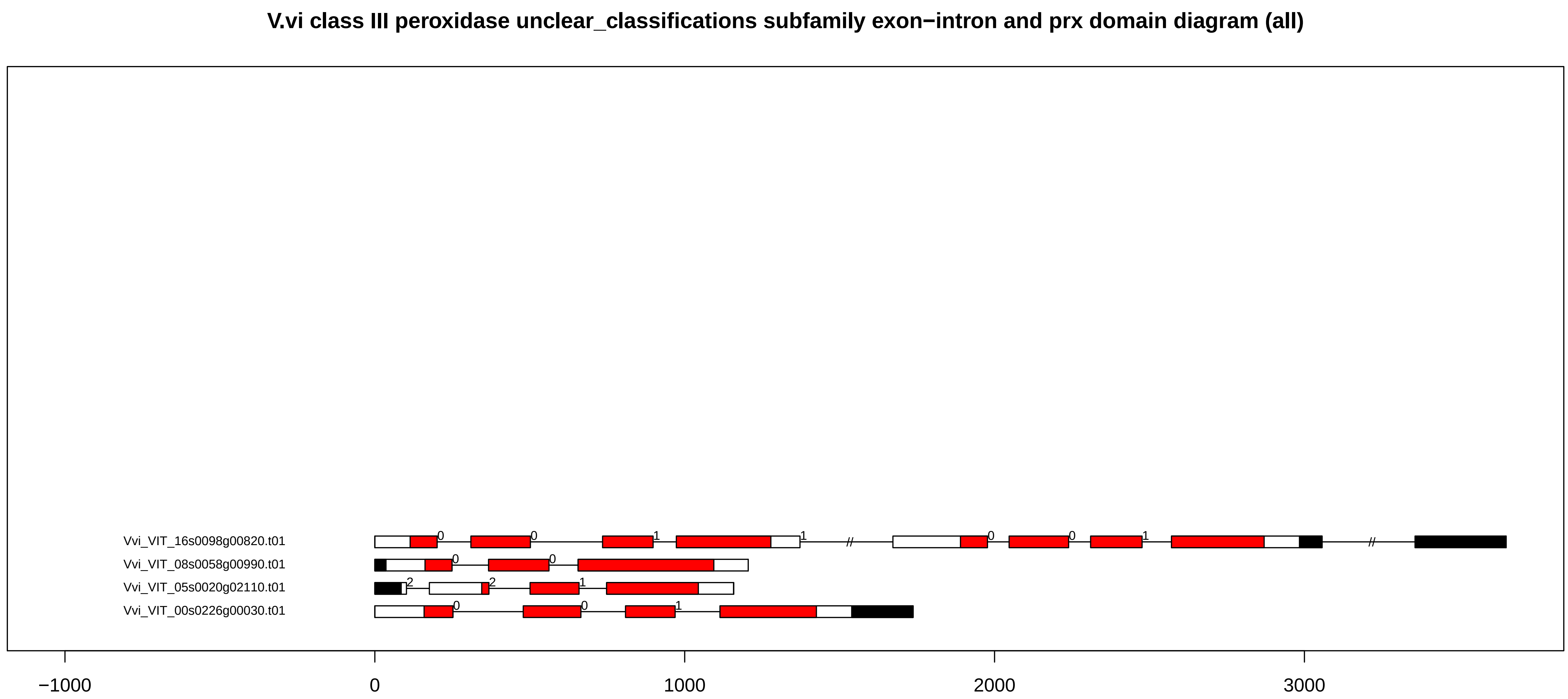
V.vi class III peroxidase unclear_classifications subfamily exon−intron and prx domain diagram (all)
Vvi_VIT_16s0098g00820.t01
Vvi_VIT_08s0058g00990.t01
Vvi_VIT_05s0020g02110.t01
Vvi_VIT_00s0226g00030.t01
−1000
0
1000
2000
3000

S.mo class III peroxidase I subfamily exon−intron and prx domain diagram (part 1)

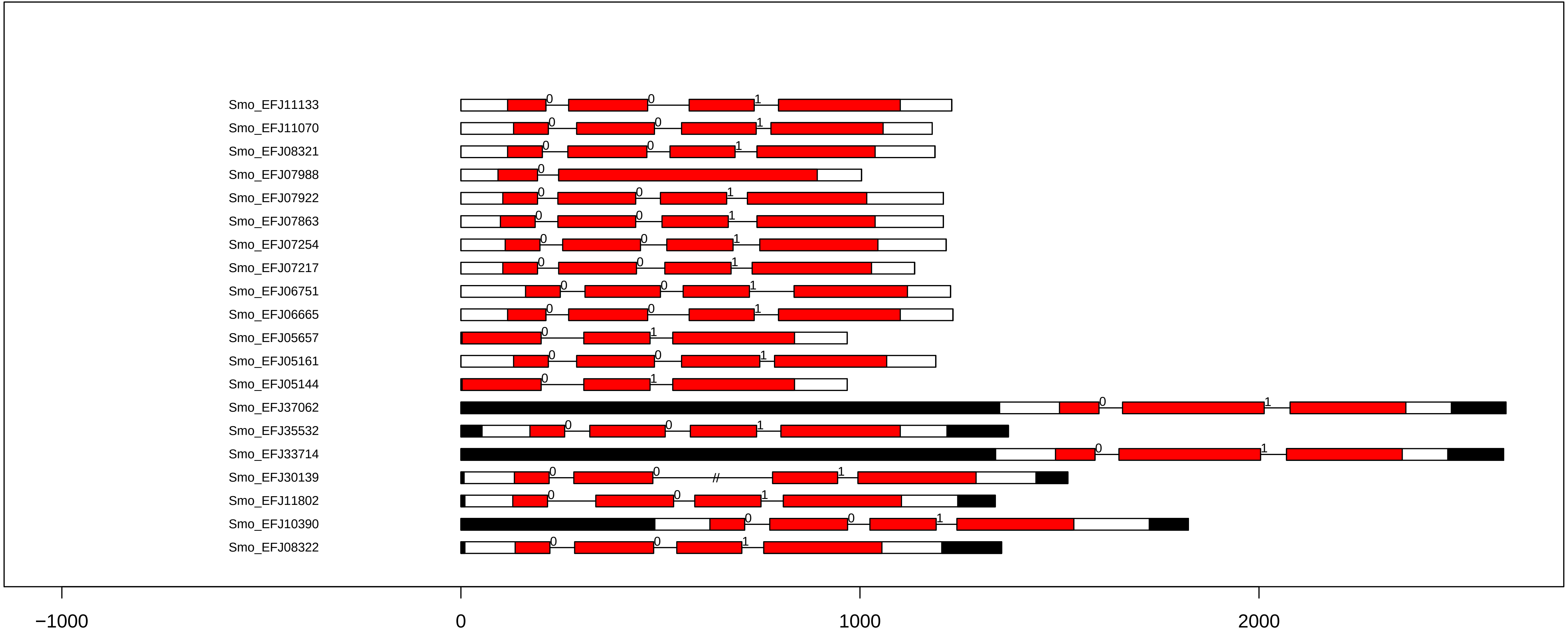

S.mo class III peroxidase I subfamily exon−intron and prx domain diagram (part 2)

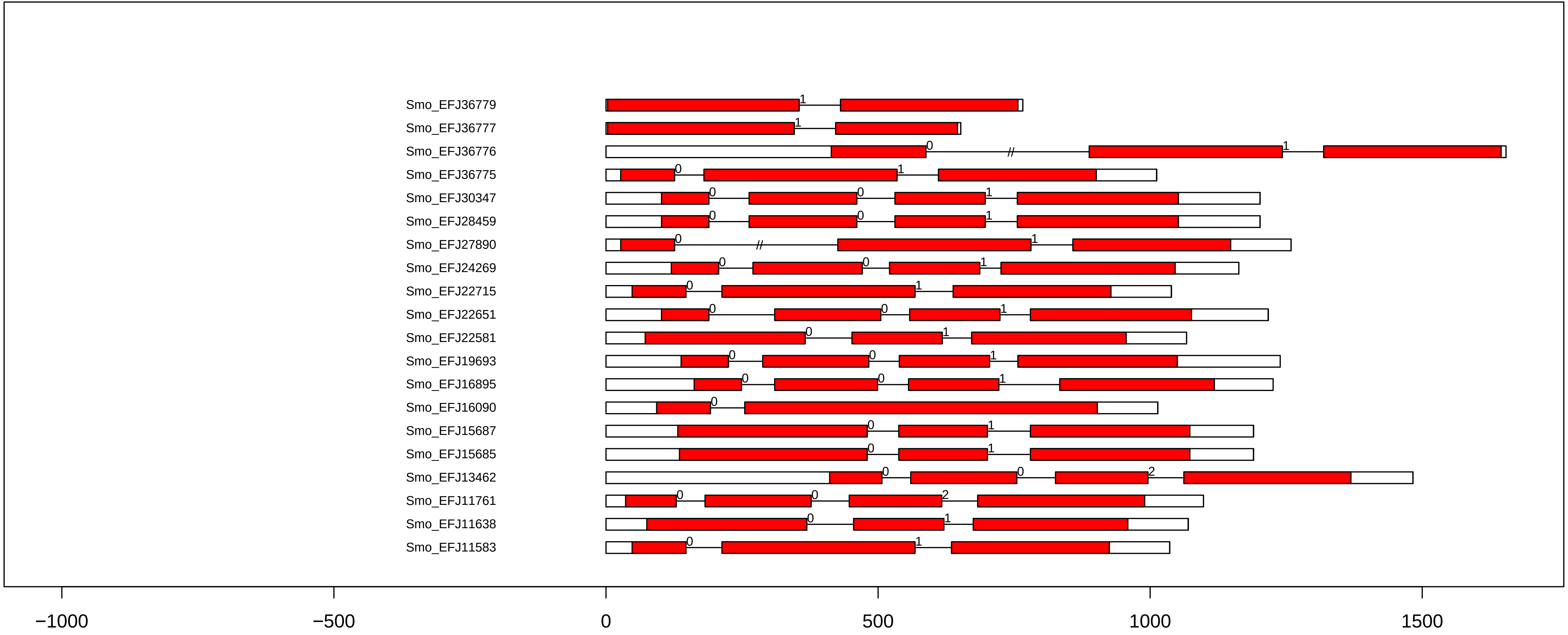

S.mo class III peroxidase I subfamily exon−intron and prx domain diagram (part 3)

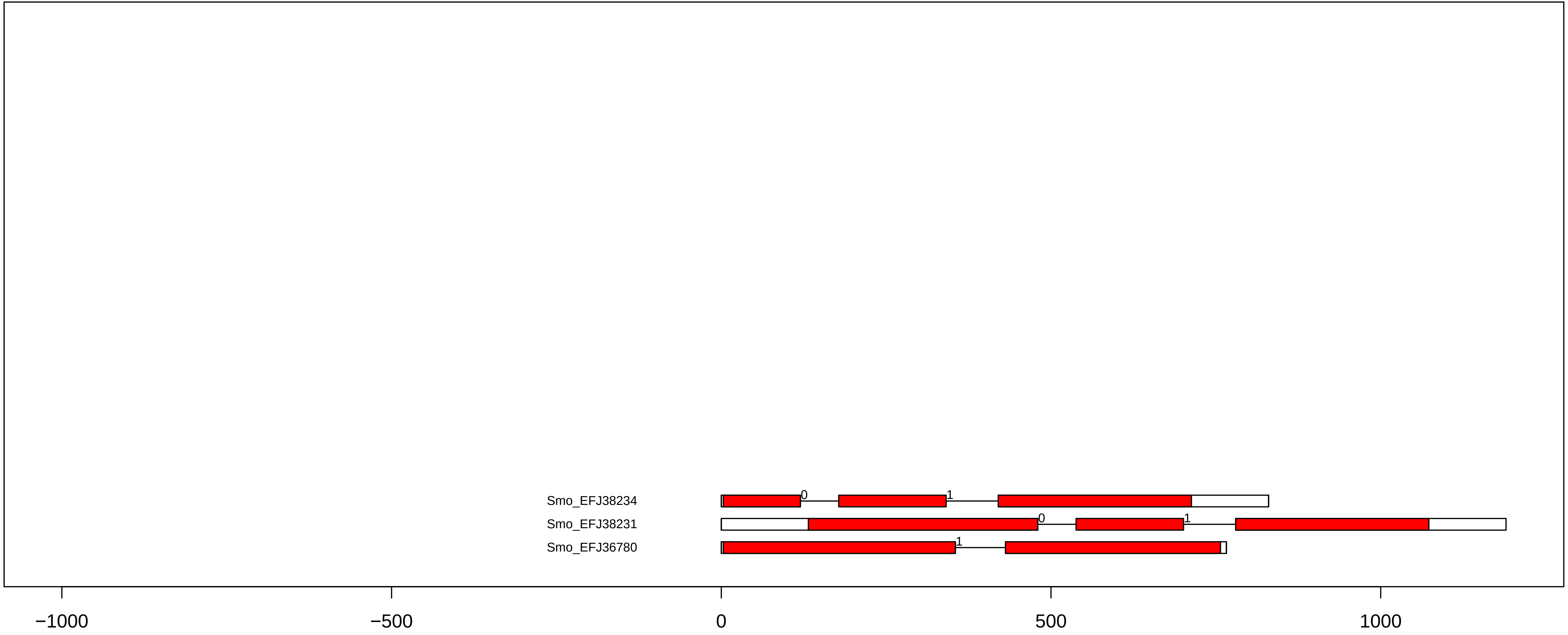

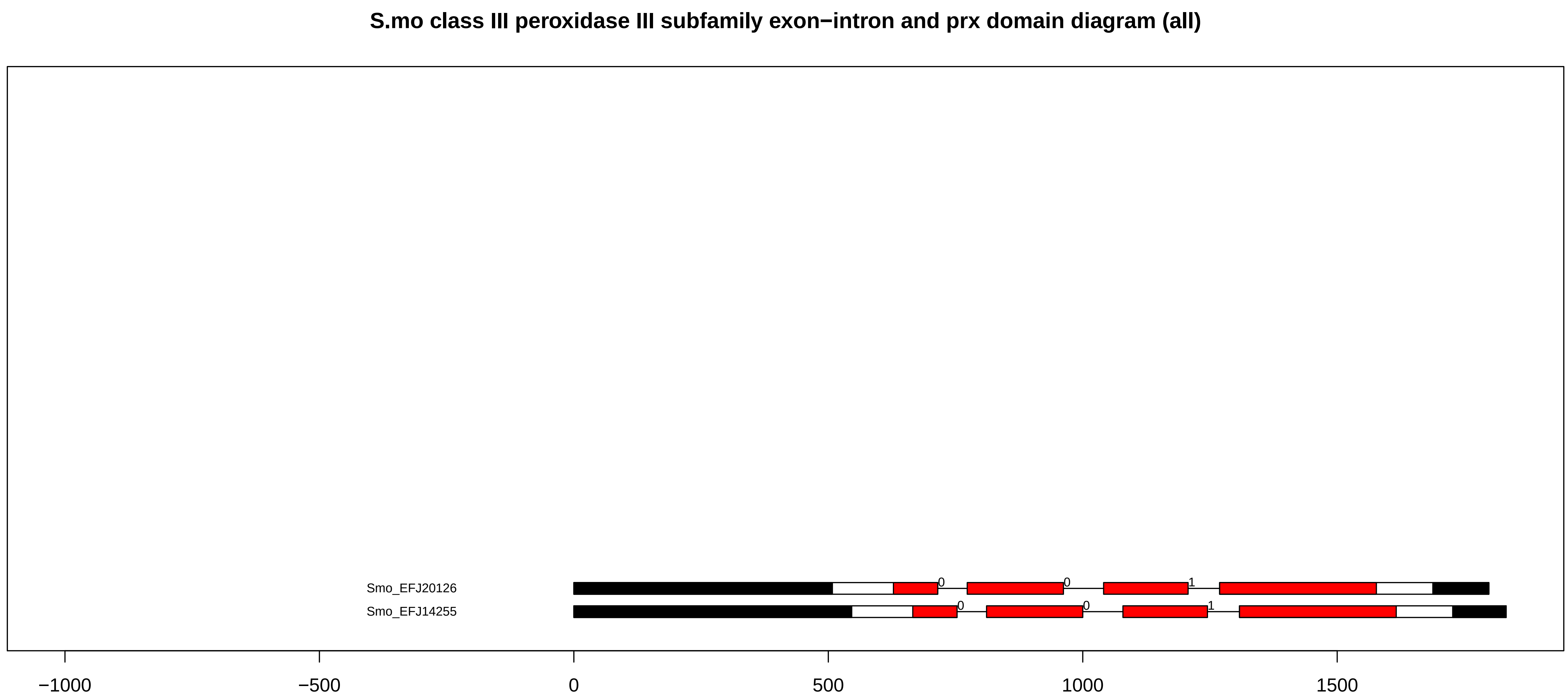

S.mo class III peroxidase III subfamily exon−intron and prx domain diagram (all)
Smo_EFJ20126
Smo_EFJ14255
0
0
1
0
0
1
−1000
−500
0
500
1000
1500

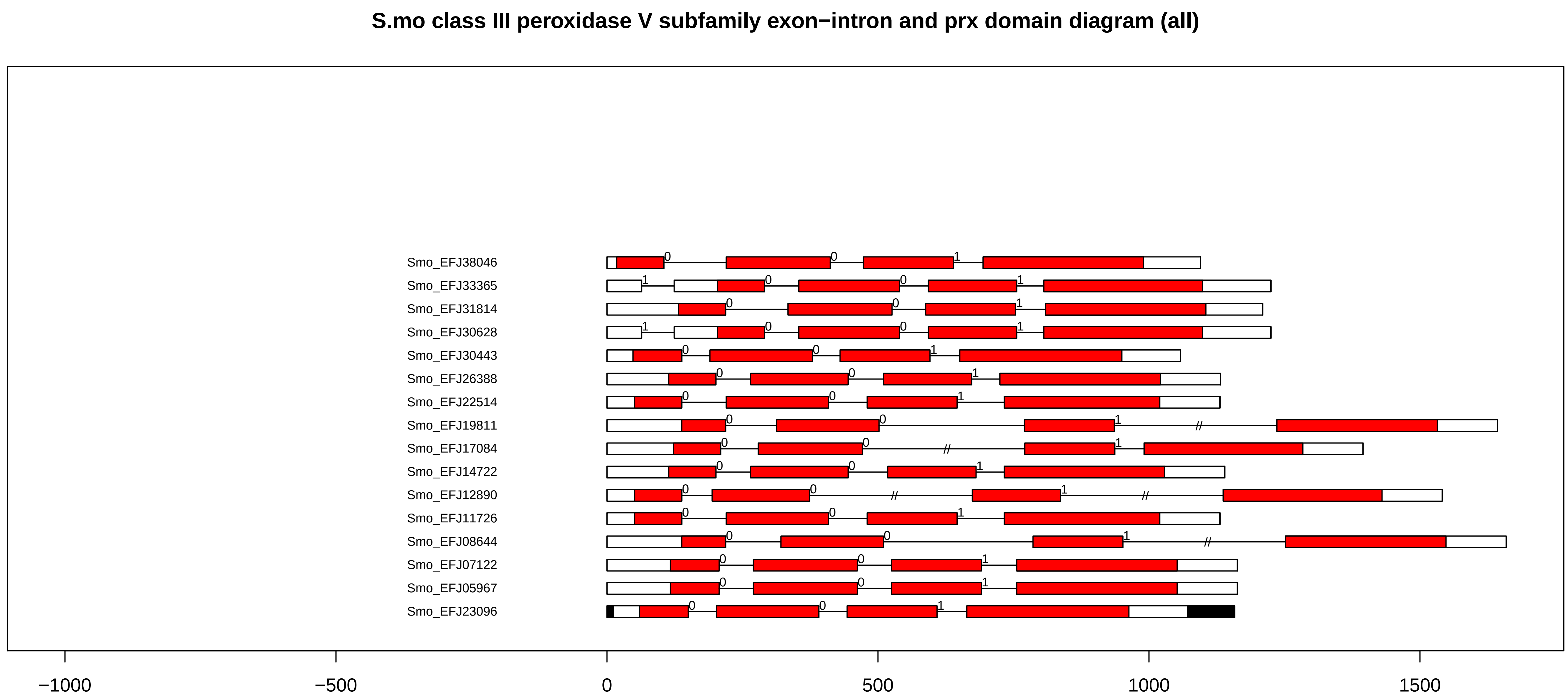

S.mo class III peroxidase V subfamily exon−intron and prx domain diagram (all)
Smo_EFJ38046
Smo_EFJ33365
Smo_EFJ31814
Smo_EFJ30628
Smo_EFJ30443
Smo_EFJ26388
Smo_EFJ22514
Smo_EFJ19811
Smo_EFJ17084
Smo_EFJ14722
Smo_EFJ12890
Smo_EFJ11726
Smo_EFJ08644
Smo_EFJ07122
Smo_EFJ05967
Smo_EFJ23096
−1000
−500
0
500
1000
1500

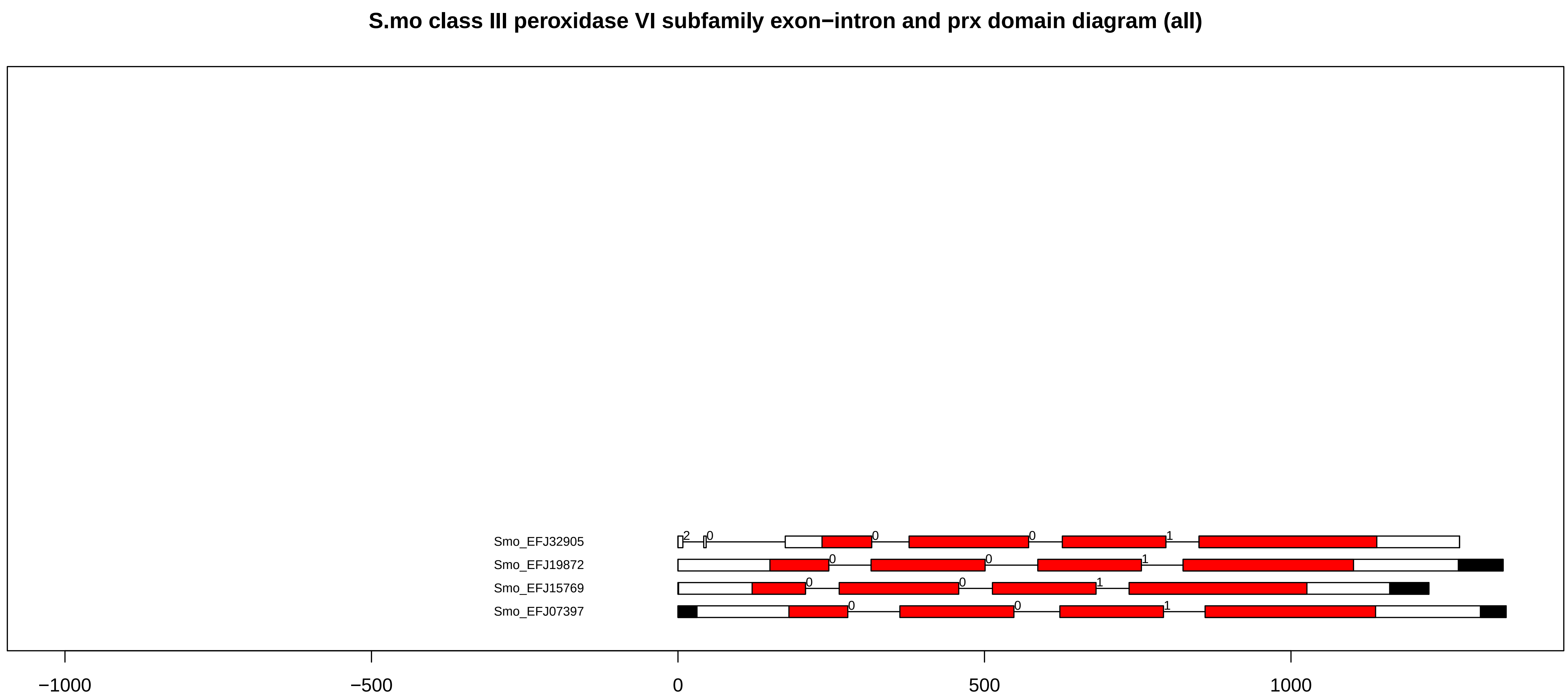
S.mo class III peroxidase VI subfamily exon−intron and prx domain diagram (all)
Smo_EFJ32905
Smo_EFJ19872
Smo_EFJ15769
Smo_EFJ07397
−1000
−500
0
500
1000

S.mo class III peroxidase VII subfamily exon−intron and prx domain diagram (all)

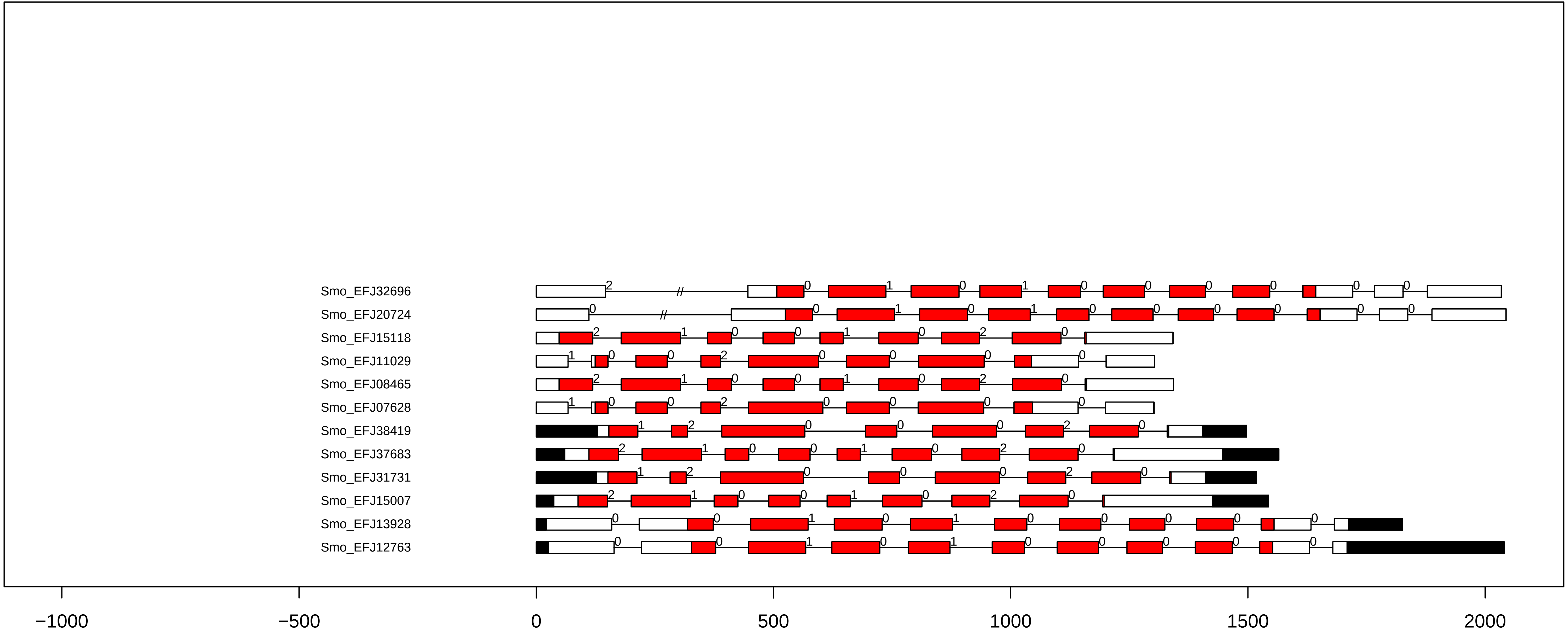

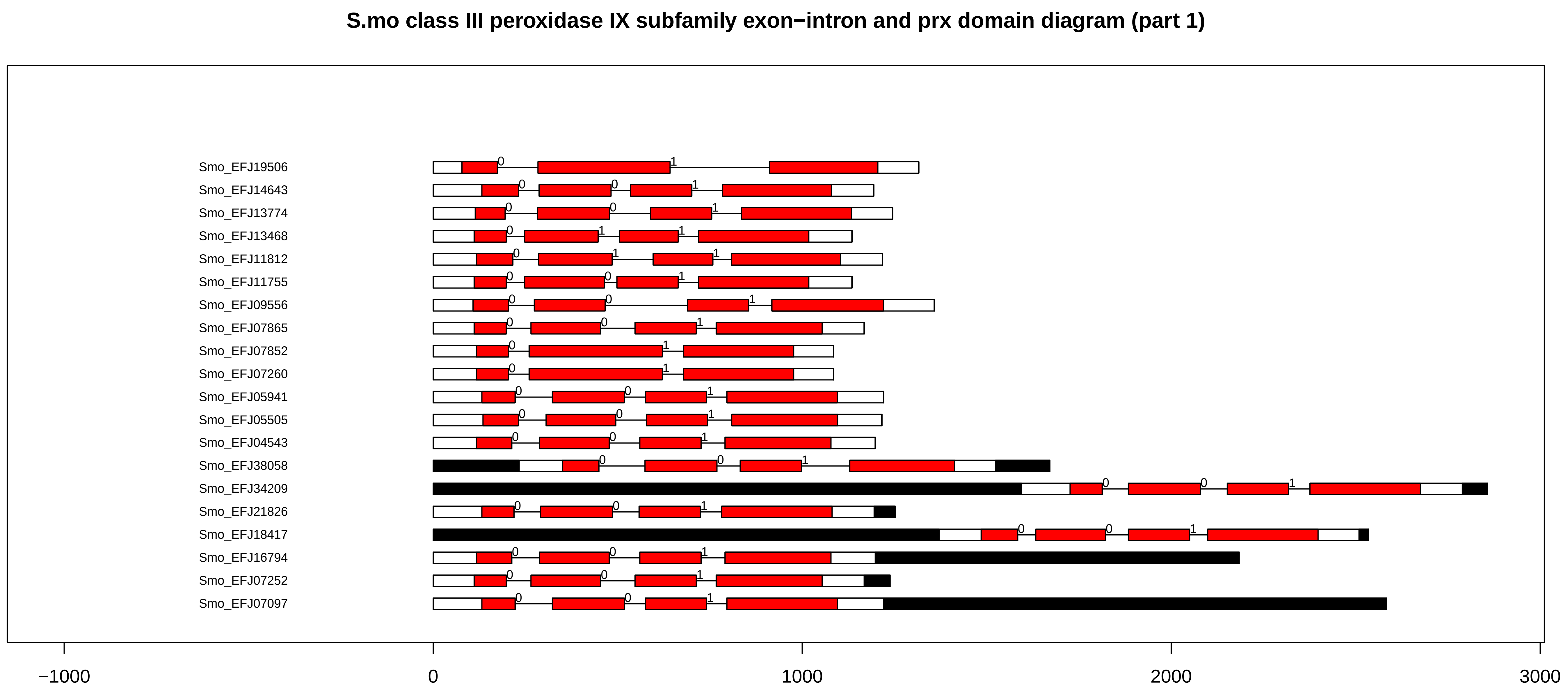
S.mo class III peroxidase IX subfamily exon−intron and prx domain diagram (part 1)
Smo_EFJ19506
Smo_EFJ14643
Smo_EFJ13774
Smo_EFJ13468
Smo_EFJ11812
Smo_EFJ11755
Smo_EFJ09556
Smo_EFJ07865
Smo_EFJ07852
Smo_EFJ07260
Smo_EFJ05941
Smo_EFJ05505
Smo_EFJ04543
Smo_EFJ38058
Smo_EFJ34209
Smo_EFJ21826
Smo_EFJ18417
Smo_EFJ16794
Smo_EFJ07252
Smo_EFJ07097
−1000
0
1000
2000
3000

S.mo class III peroxidase IX subfamily exon−intron and prx domain diagram (part 2)

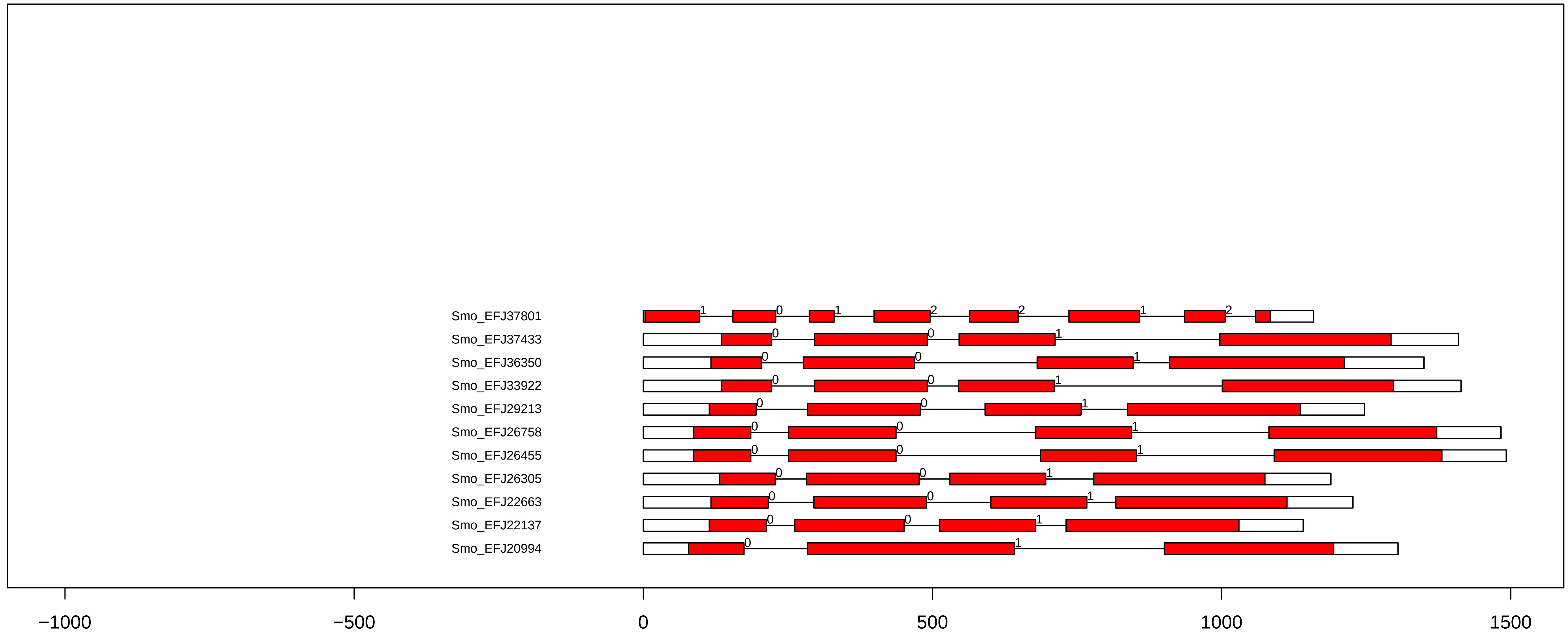

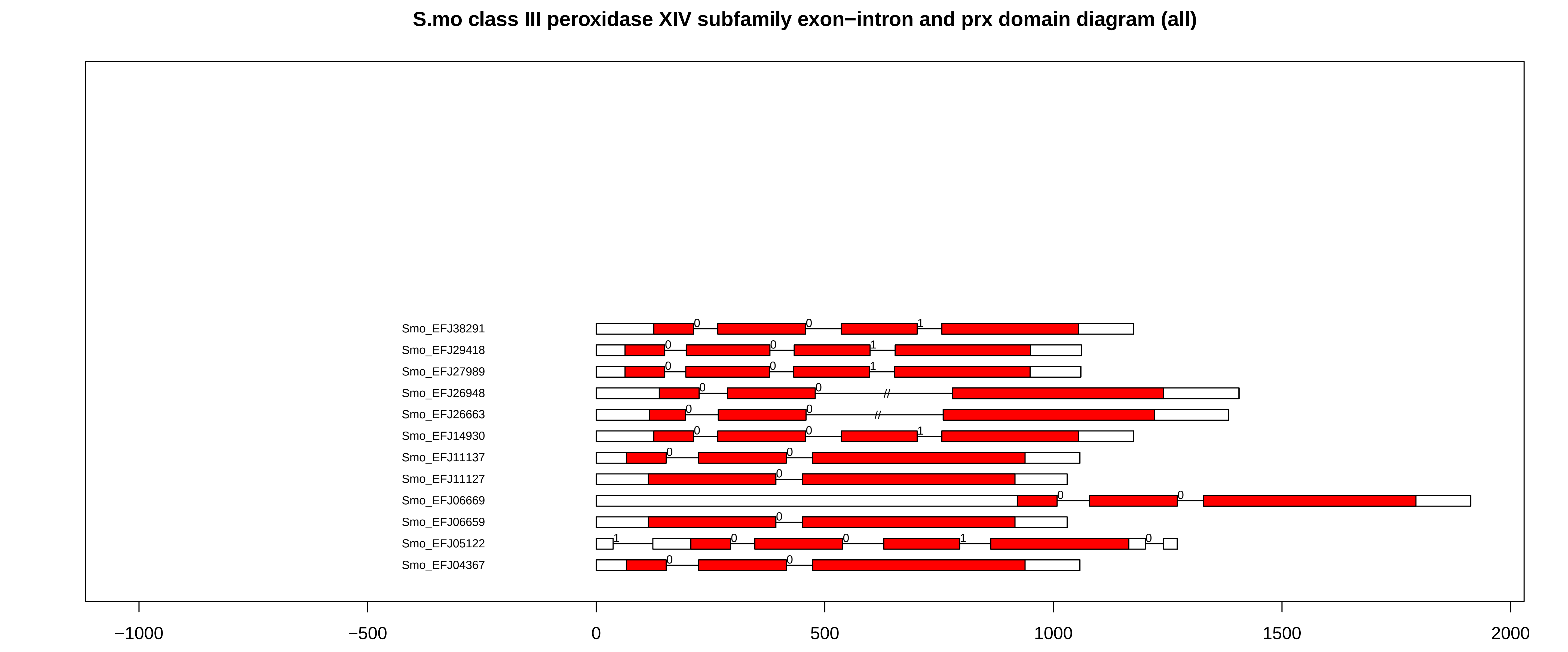
S.mo class III peroxidase XIV subfamily exon−intron and prx domain diagram (all)
Smo_EFJ38291
Smo_EFJ29418
Smo_EFJ27989
Smo_EFJ26948
Smo_EFJ26663
Smo_EFJ14930
Smo_EFJ11137
Smo_EFJ11127
Smo_EFJ06669
Smo_EFJ06659
Smo_EFJ05122
Smo_EFJ04367
−1000
−500
0
500
1000
1500
2000

S.mo class III peroxidase XV subfamily exon−intron and prx domain diagram (all)

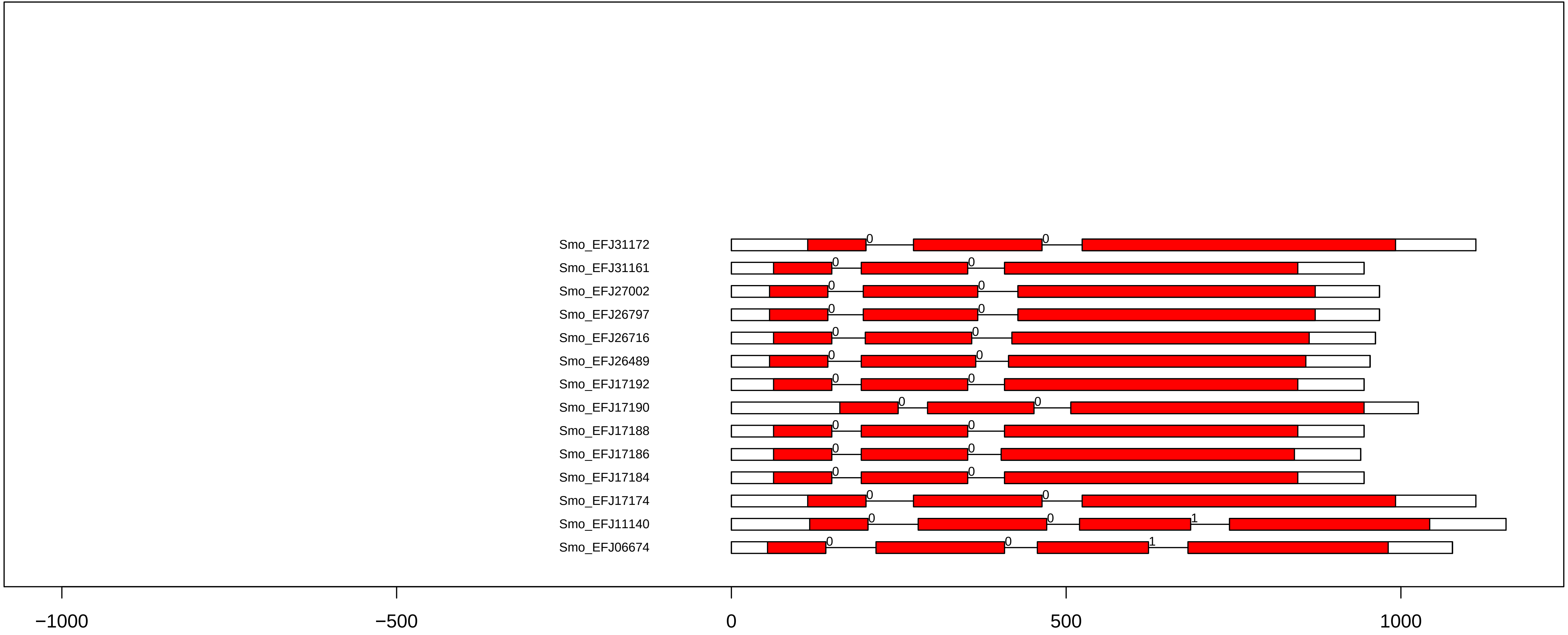

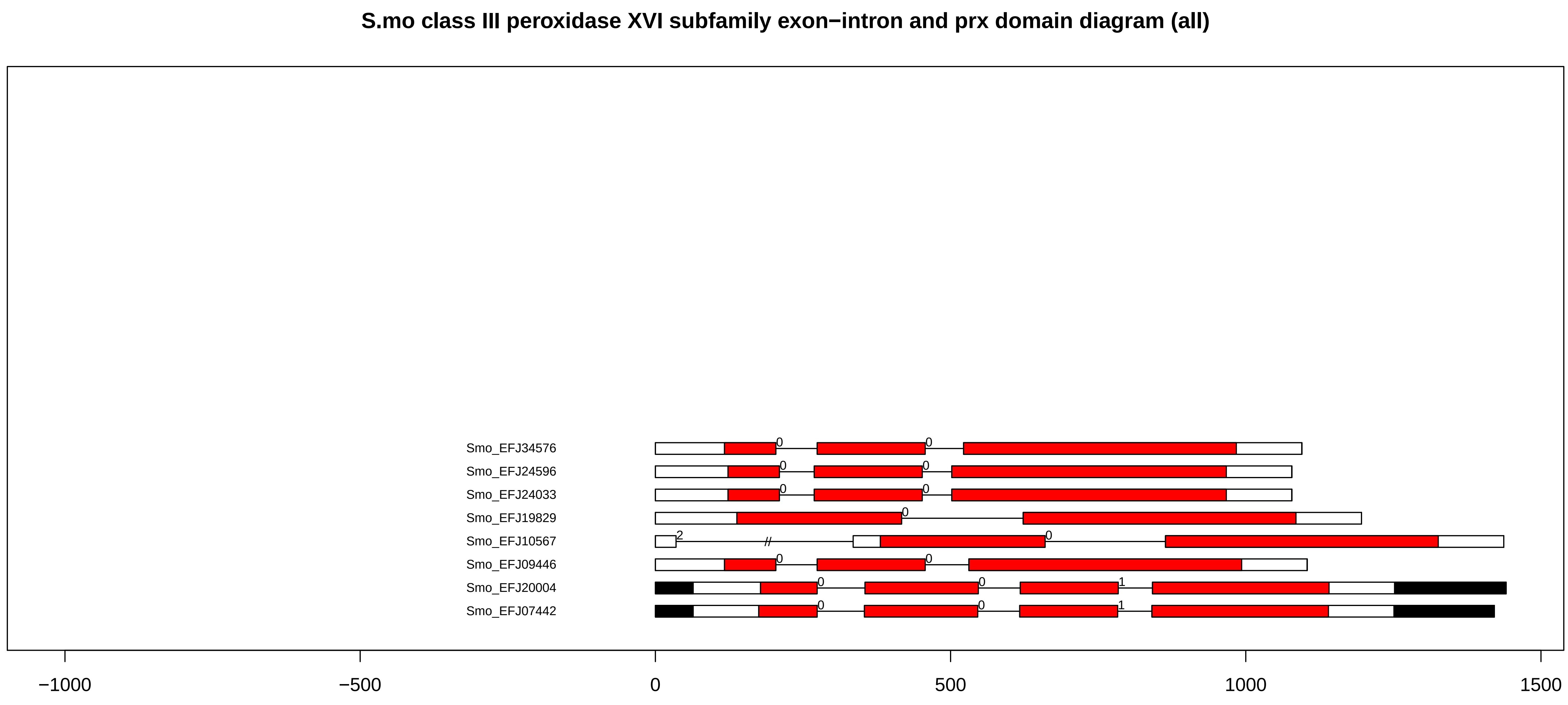
S.mo class III peroxidase XVI subfamily exon−intron and prx domain diagram (all)
Smo_EFJ34576
Smo_EFJ24596
Smo_EFJ24033
Smo_EFJ19829
Smo_EFJ10567
Smo_EFJ09446
Smo_EFJ20004
Smo_EFJ07442
−1000
−500
0
500
1000
1500

S.mo class III peroxidase XVII subfamily exon−intron and prx domain diagram (all)

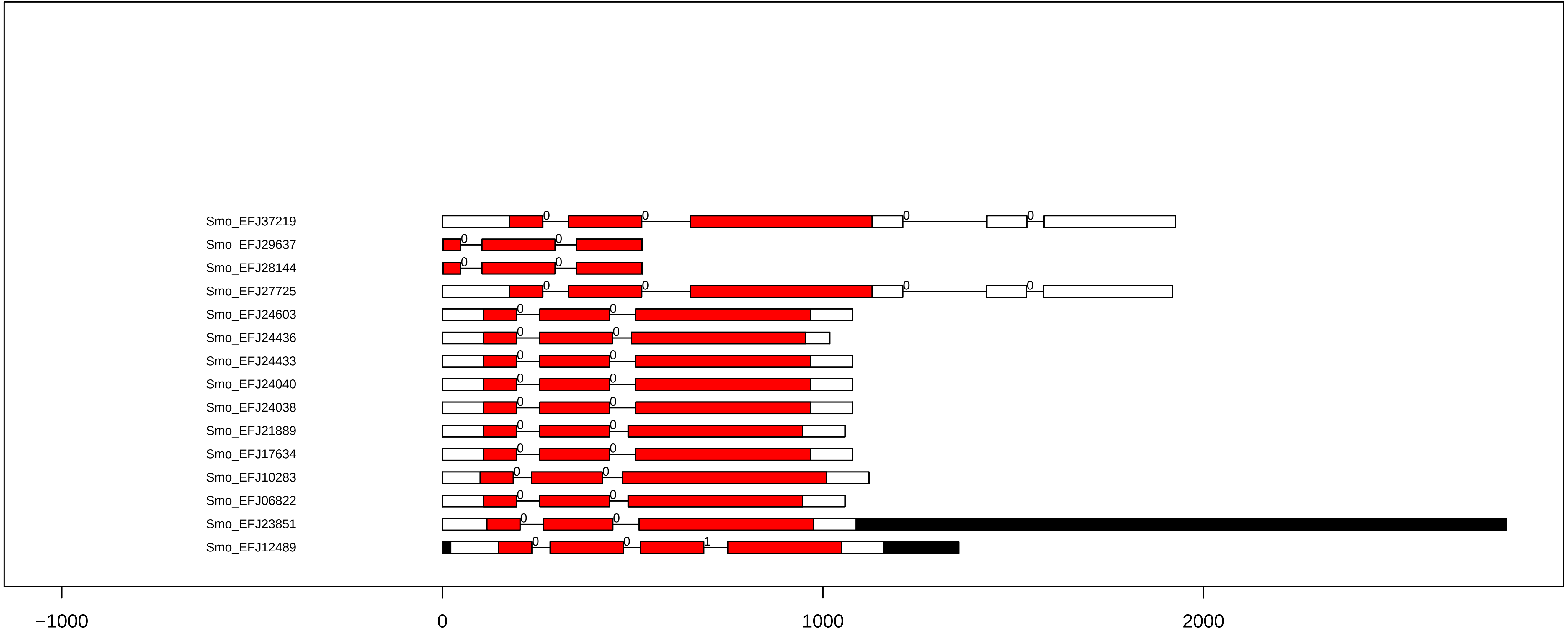

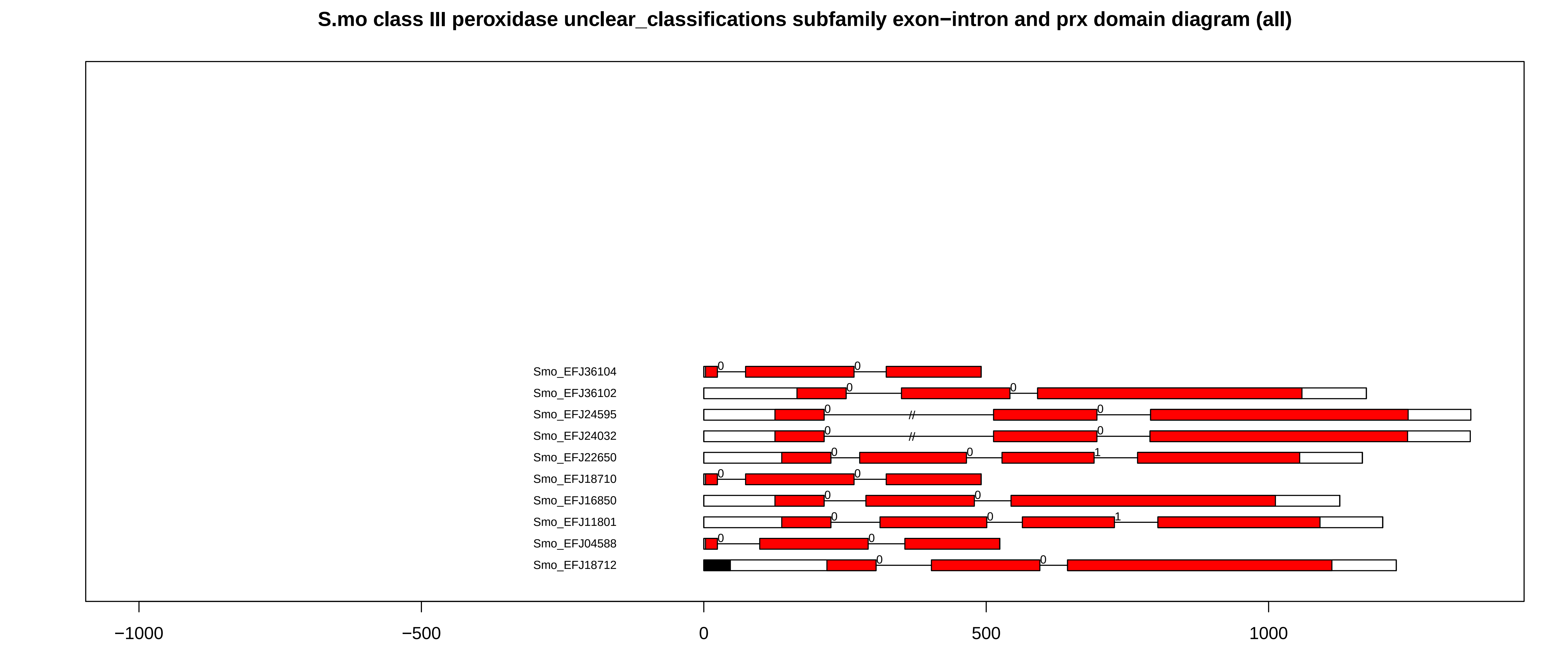
S.mo class III peroxidase unclear_classifications subfamily exon−intron and prx domain diagram (all)
Smo_EFJ36104
Smo_EFJ36102
Smo_EFJ24595
Smo_EFJ24032
Smo_EFJ22650
Smo_EFJ18710
Smo_EFJ16850
Smo_EFJ11801
Smo_EFJ04588
Smo_EFJ18712
−1000
−500
0
500
1000

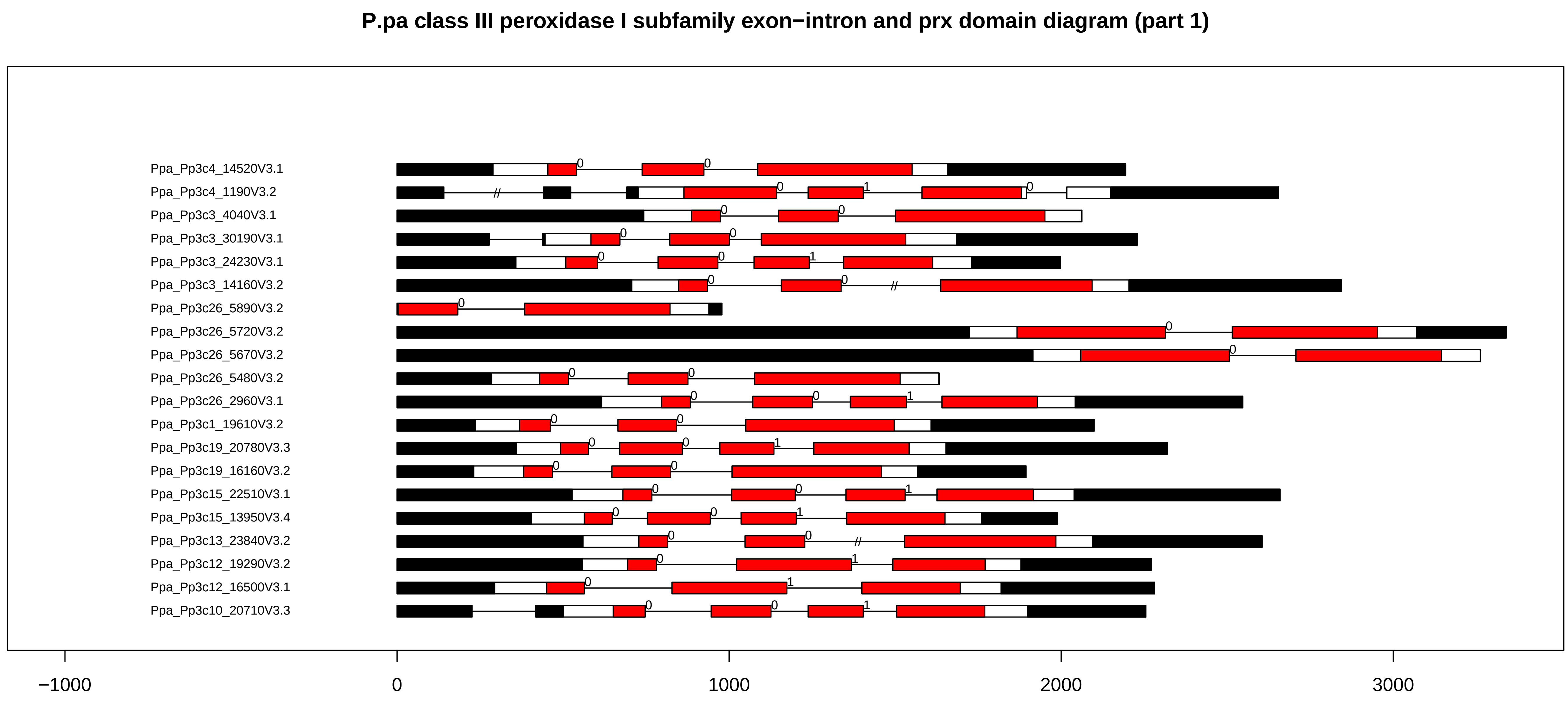

P.pa class III peroxidase I subfamily exon−intron and prx domain diagram (part 1)
Ppa_Pp3c4_14520V3.1
Ppa_Pp3c4_1190V3.2
Ppa_Pp3c3_4040V3.1
Ppa_Pp3c3_30190V3.1
Ppa_Pp3c3_24230V3.1
Ppa_Pp3c3_14160V3.2
Ppa_Pp3c26_5890V3.2
Ppa_Pp3c26_5720V3.2
Ppa_Pp3c26_5670V3.2
Ppa_Pp3c26_5480V3.2
Ppa_Pp3c26_2960V3.1
Ppa_Pp3c1_19610V3.2
Ppa_Pp3c19_20780V3.3
Ppa_Pp3c19_16160V3.2
Ppa_Pp3c15_22510V3.1
Ppa_Pp3c15_13950V3.4
Ppa_Pp3c13_23840V3.2
Ppa_Pp3c12_19290V3.2
Ppa_Pp3c12_16500V3.1
Ppa_Pp3c10_20710V3.3
−1000
0
1000
2000
3000

P.pa class III peroxidase I subfamily exon−intron and prx domain diagram (part 2)

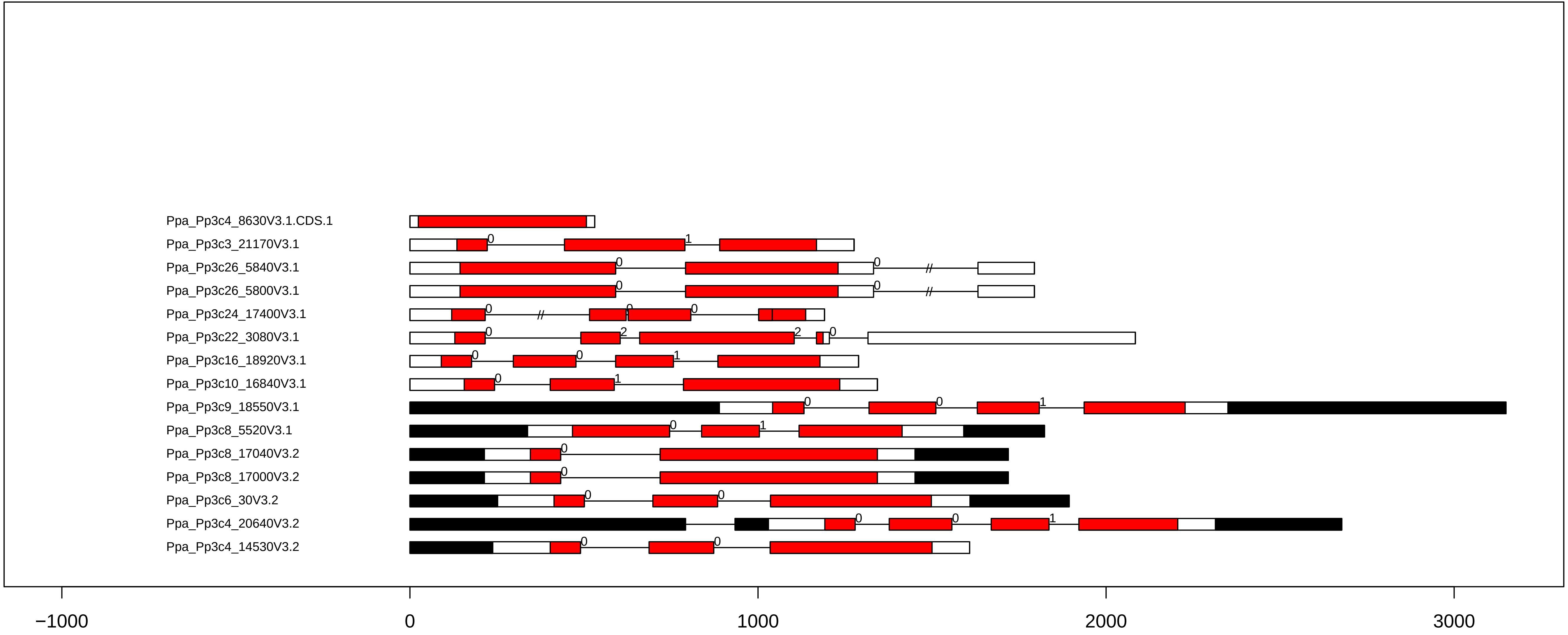

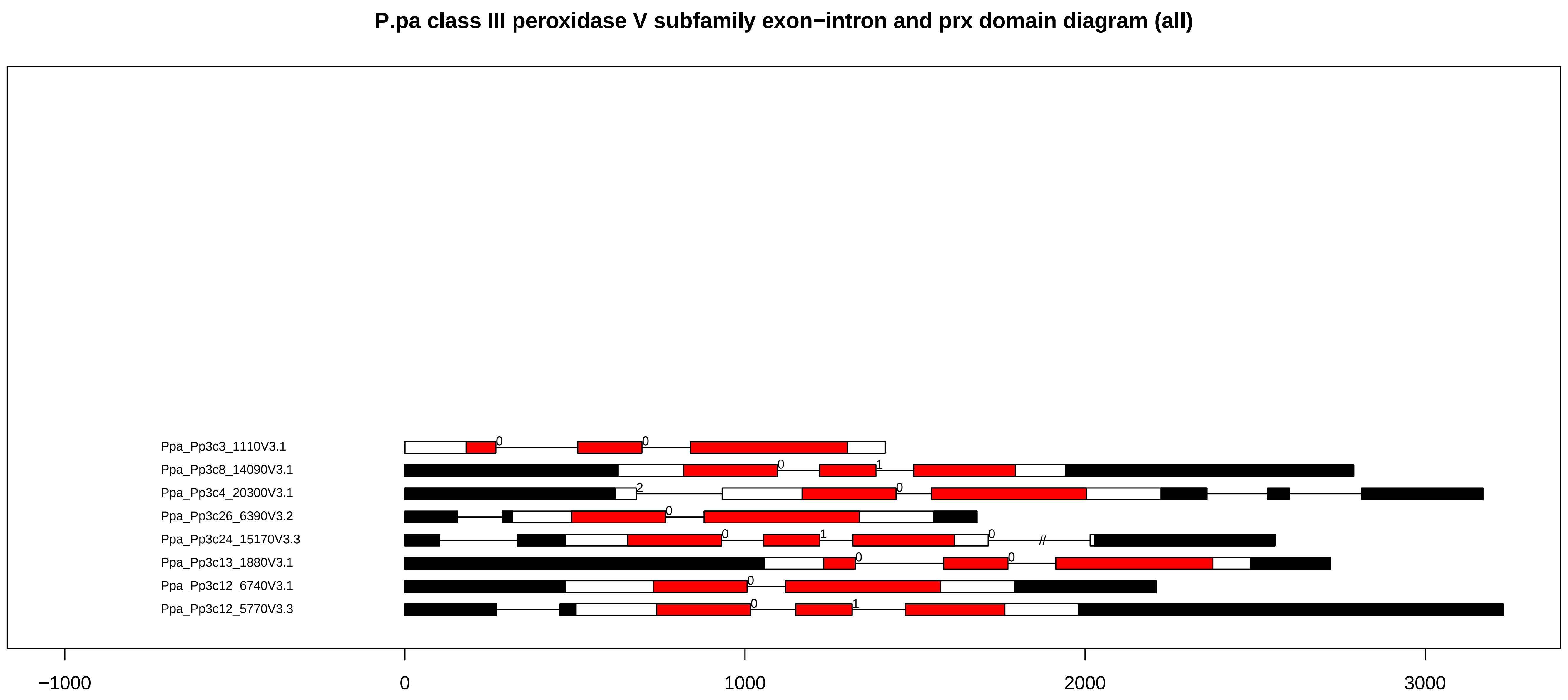

P.pa class III peroxidase V subfamily exon−intron and prx domain diagram (all)
Ppa_Pp3c3_1110V3.1
Ppa_Pp3c8_14090V3.1
Ppa_Pp3c4_20300V3.1
Ppa_Pp3c26_6390V3.2
Ppa_Pp3c24_15170V3.3
Ppa_Pp3c13_1880V3.1
Ppa_Pp3c12_6740V3.1
Ppa_Pp3c12_5770V3.3
−1000
0
1000
2000
3000

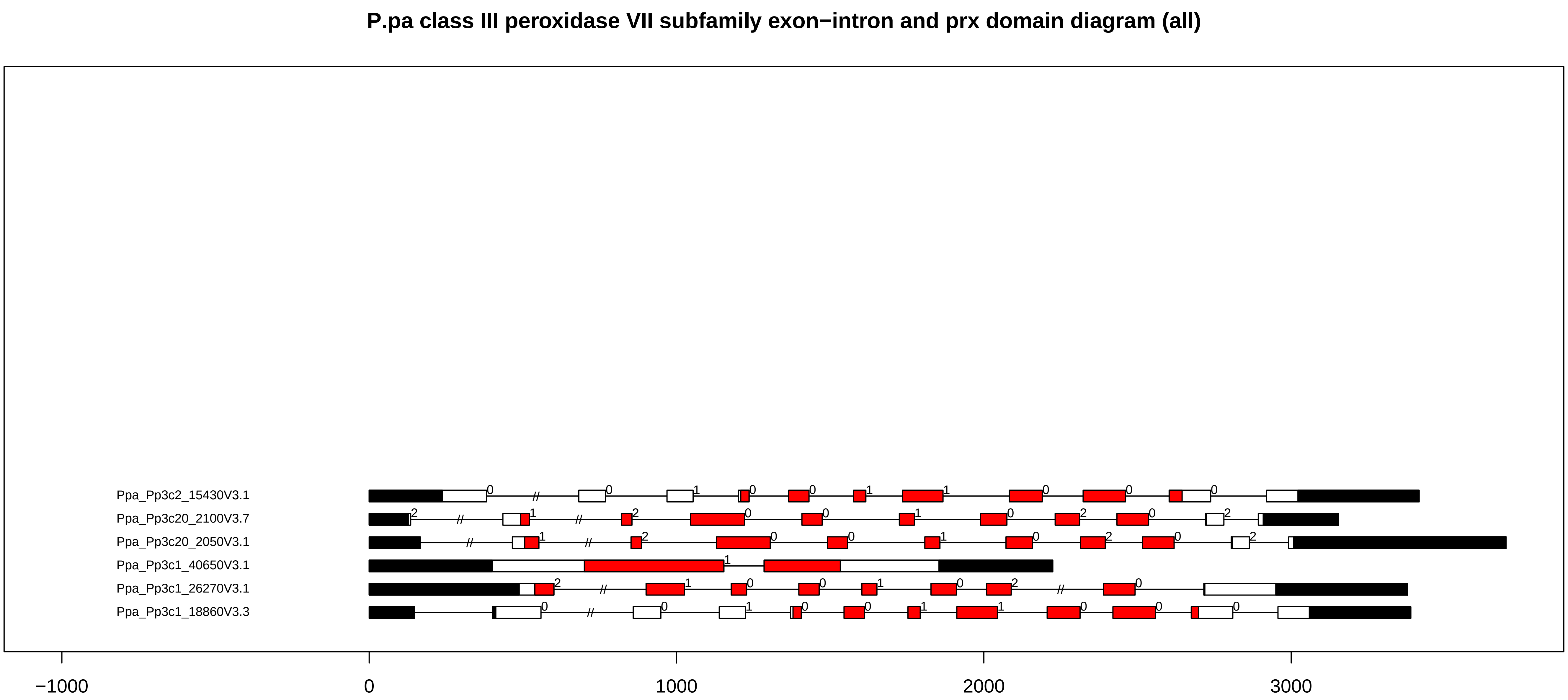
P.pa class III peroxidase VII subfamily exon−intron and prx domain diagram (all)
Ppa_Pp3c2_15430V3.1
Ppa_Pp3c20_2100V3.7
Ppa_Pp3c20_2050V3.1
Ppa_Pp3c1_40650V3.1
Ppa_Pp3c1_26270V3.1
Ppa_Pp3c1_18860V3.3
−1000
0
1000
2000
3000

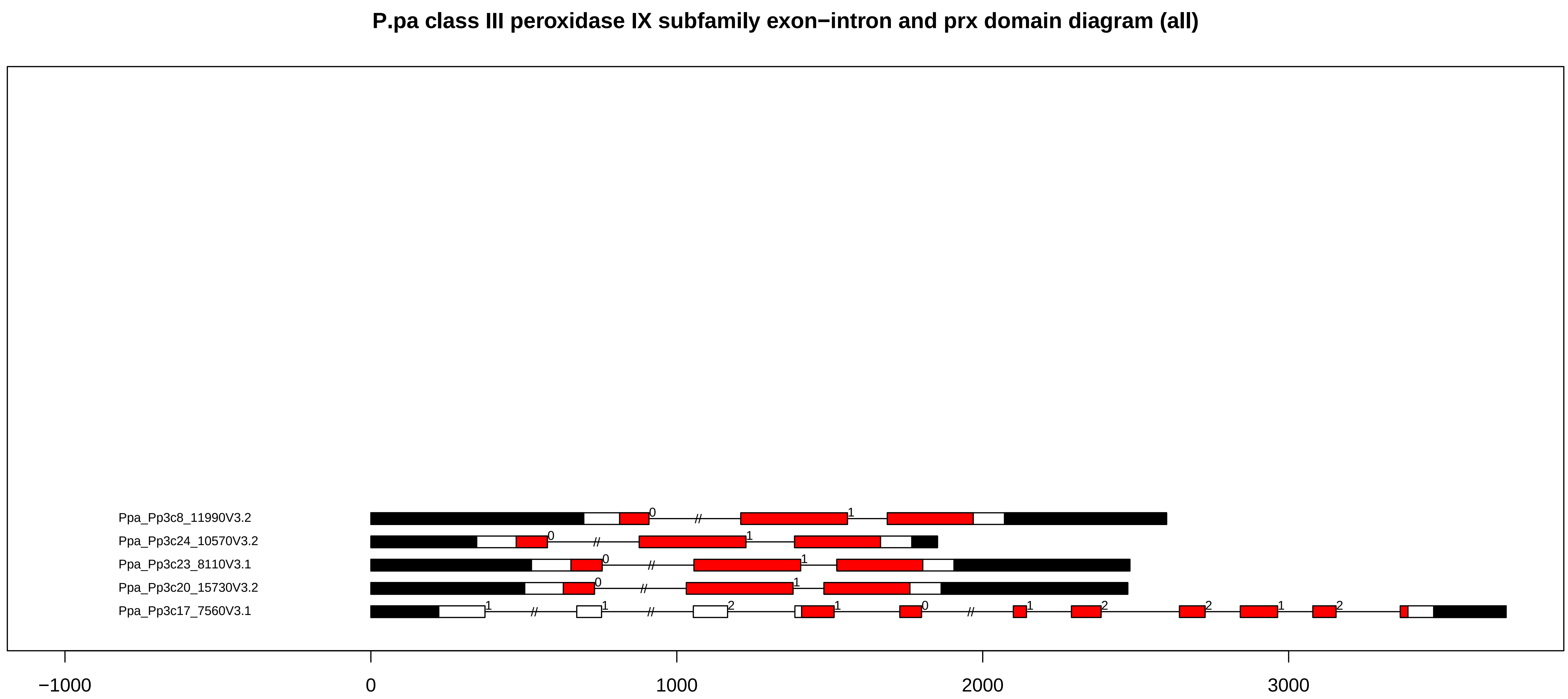
P.pa class III peroxidase IX subfamily exon−intron and prx domain diagram (all)
Ppa_Pp3c8_11990V3.2
Ppa_Pp3c24_10570V3.2
Ppa_Pp3c23_8110V3.1
Ppa_Pp3c20_15730V3.2
Ppa_Pp3c17_7560V3.1
−1000
0
1000
2000
3000

P.pa class III peroxidase XIV subfamily exon−intron and prx domain diagram (all)

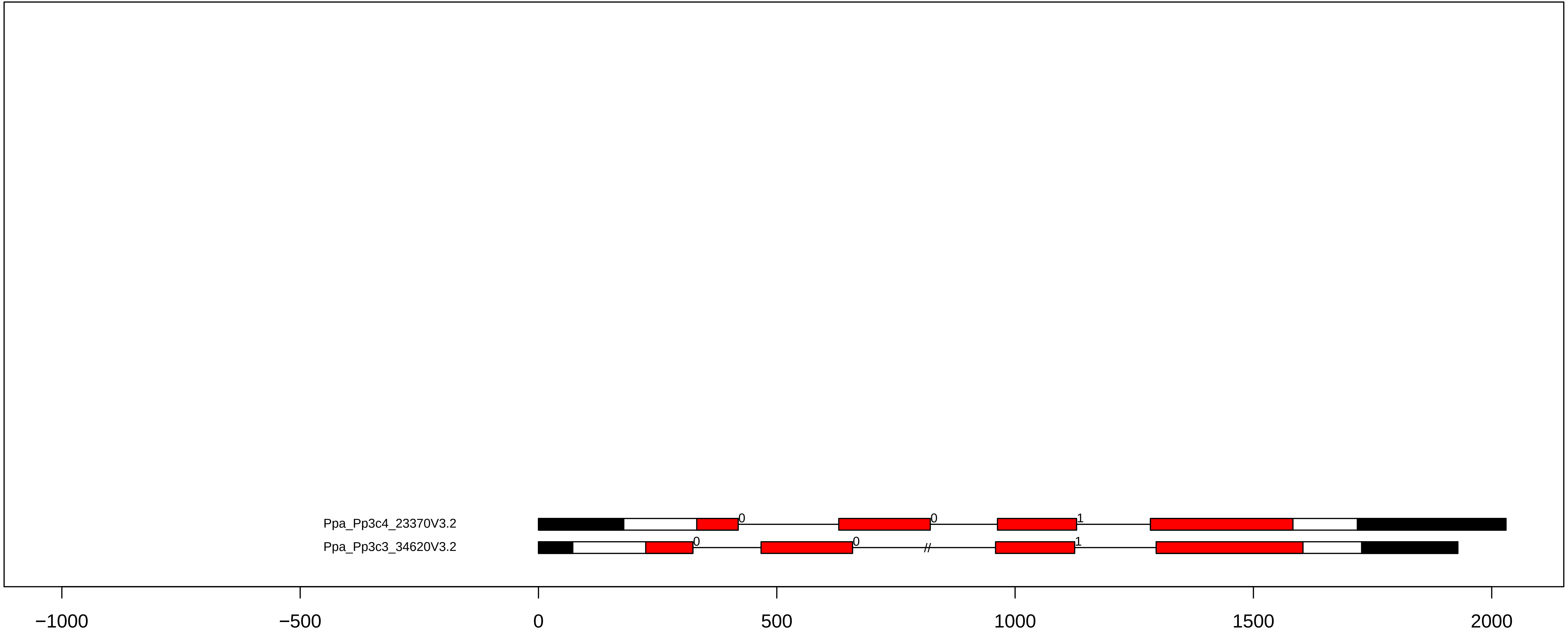

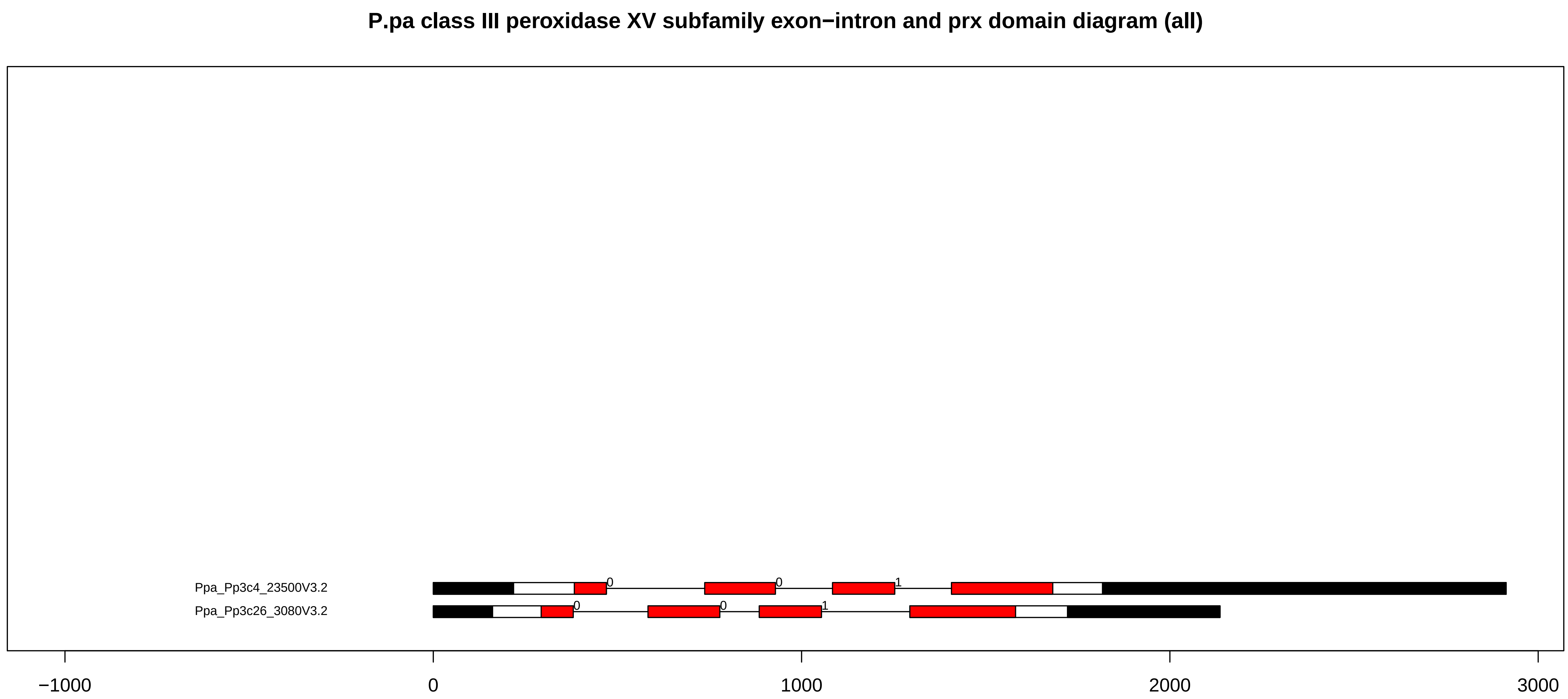

P.pa class III peroxidase XV subfamily exon−intron and prx domain diagram (all)
Ppa_Pp3c4_23500V3.2
Ppa_Pp3c26_3080V3.2
0
0
1
0
0
1
−1000
0
1000
2000
3000

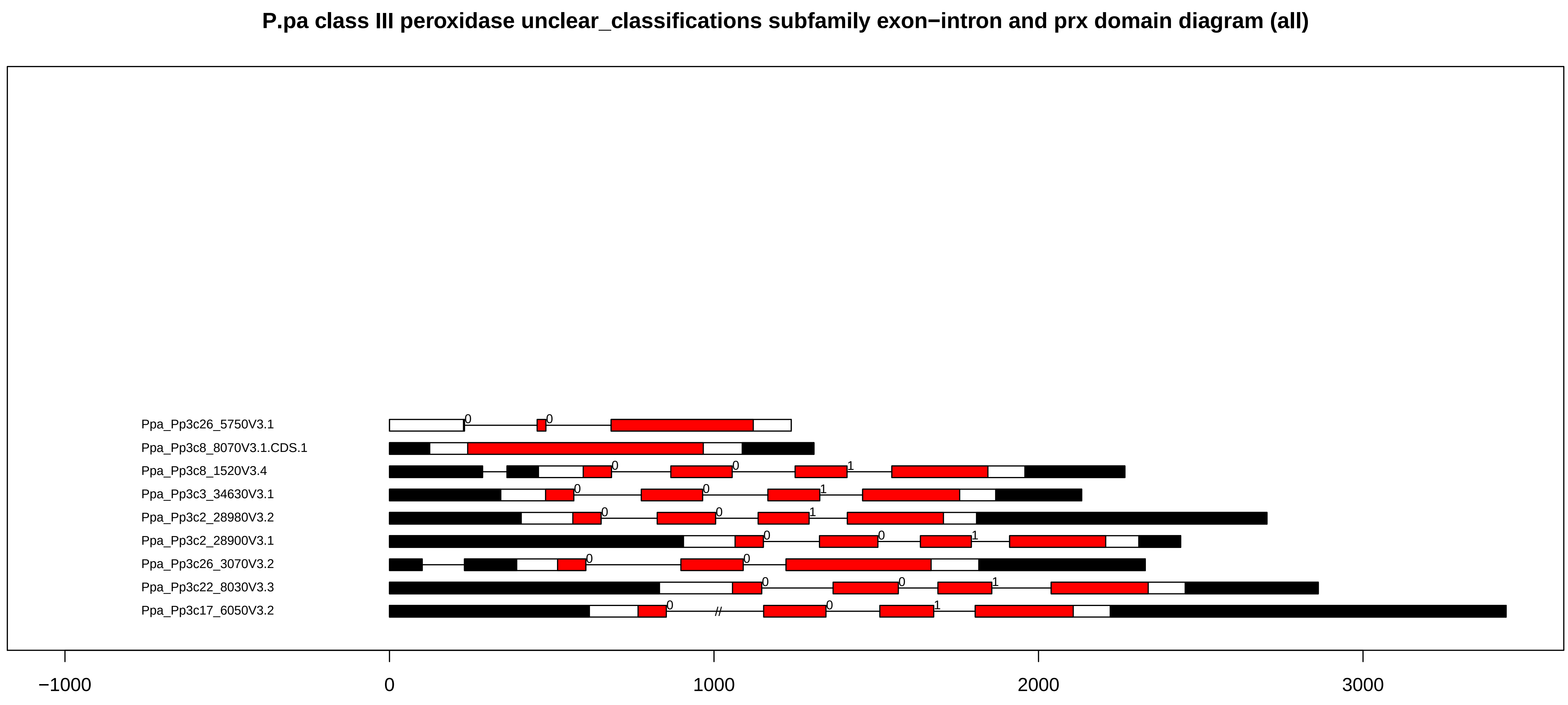
P.pa class III peroxidase unclear_classifications subfamily exon−intron and prx domain diagram (all)
Ppa_Pp3c26_5750V3.1
Ppa_Pp3c8_8070V3.1.CDS.1
Ppa_Pp3c8_1520V3.4
Ppa_Pp3c3_34630V3.1
Ppa_Pp3c2_28980V3.2
Ppa_Pp3c2_28900V3.1
Ppa_Pp3c26_3070V3.2
Ppa_Pp3c22_8030V3.3
Ppa_Pp3c17_6050V3.2
−1000
0
1000
2000
3000

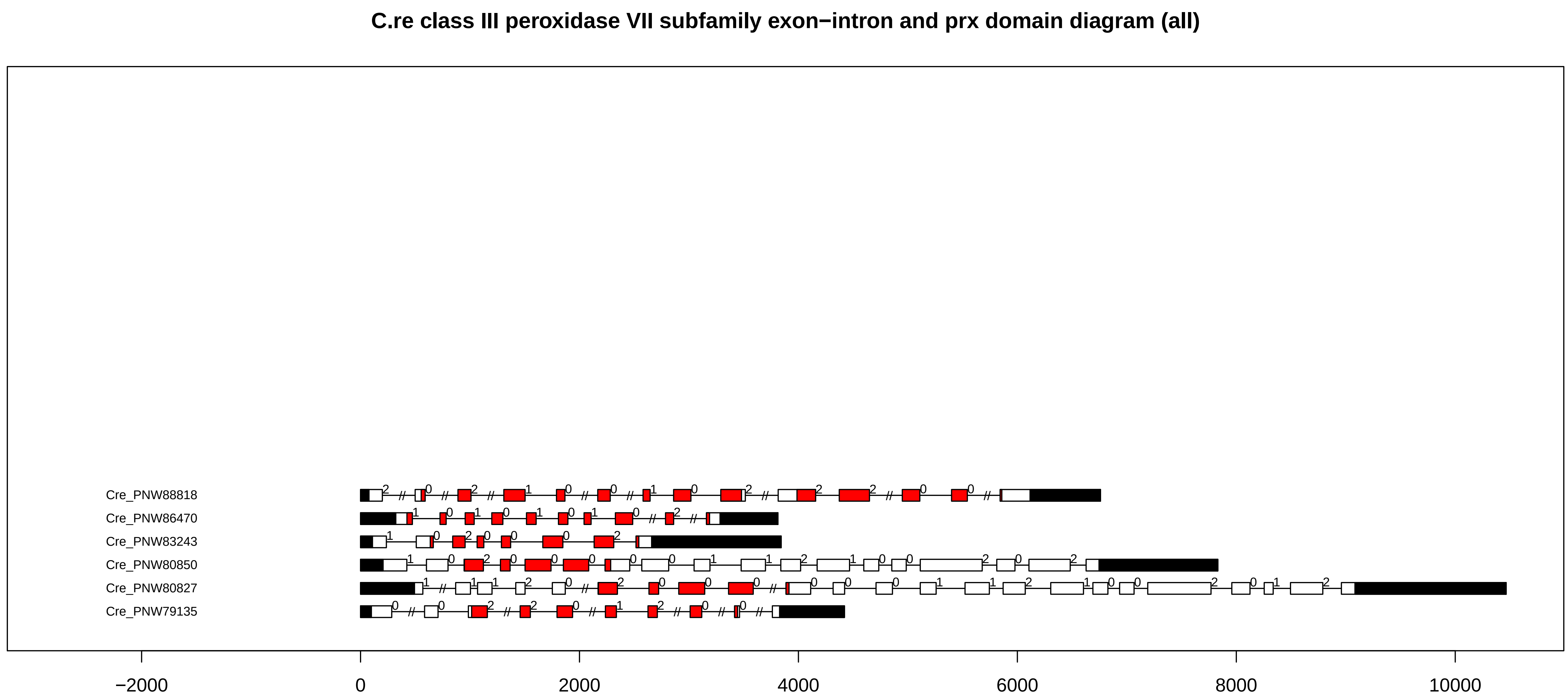

C.re class III peroxidase VII subfamily exon−intron and prx domain diagram (all)
Cre_PNW88818
Cre_PNW86470
Cre_PNW83243
Cre_PNW80850
Cre_PNW80827
Cre_PNW79135
−2000
0
2000
4000
6000
8000
10000

C.re class III peroxidase IX subfamily exon−intron and prx domain diagram (all)

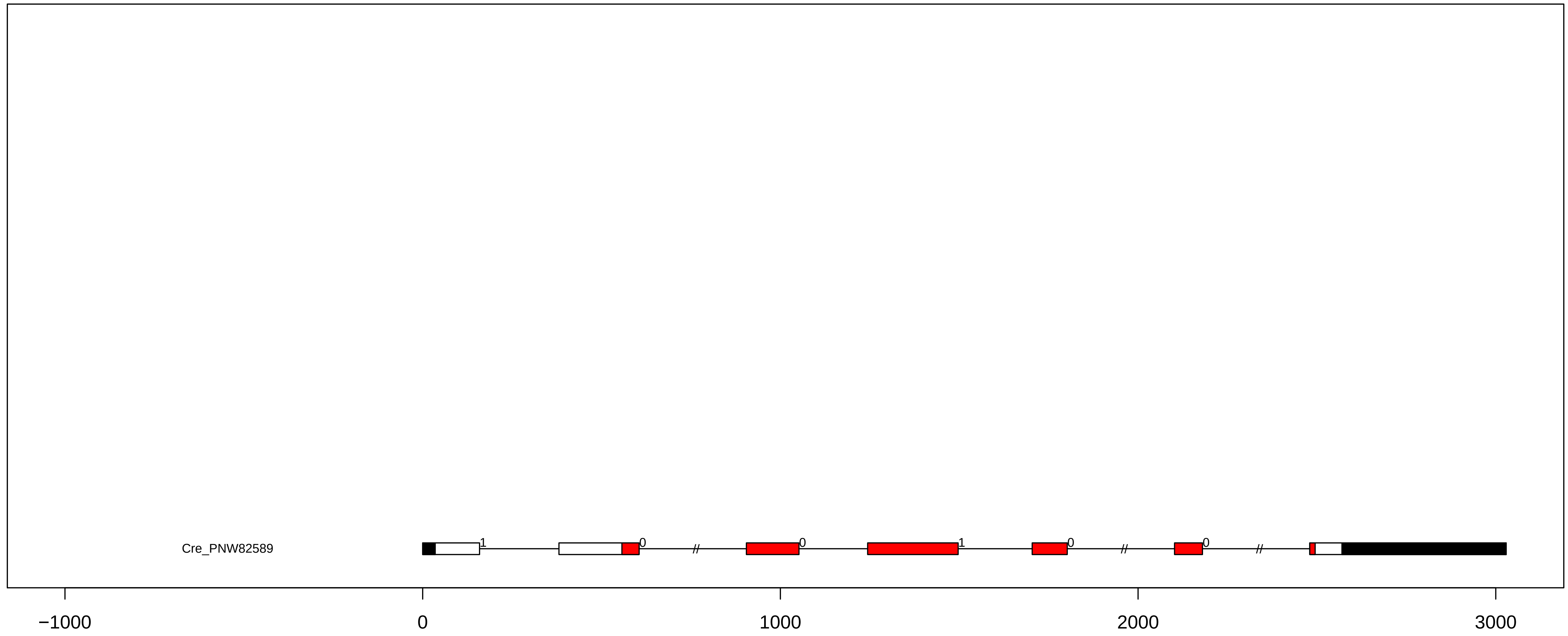

Supplement: Supplementary file 5 — Additional file 5: Figure S2. Domain and exon-intron structure diagrams of class III peroxidase in A. thaliana, V. vinifera, T. aestivum, P. patens, T. dicoccoides, T. urartu, Ae. tauschii, B. distachyon, C. reinhardtii, Z. mays, O. sativa and S. moellendorffii. Filled boxes: red represents the PRX domain; white boxes represent the other exon regions; black boxes represent the untranslated regions (UTRs); lines represent the PRX introns; numbers 0, 1, and 2 represent the exon phases. The long introns are shortened by “//”. [file 12870_2020_2602_MOESM5_ESM.pdf]
