# Supplementary material for: A member of wheat class III peroxidase gene family, TaPRX-2A, enhanced the tolerance of salt stress
Source: BMC Plant Biol. 2020 Aug 26;20:392. doi: 10.1186/s12870-020-02602-1 (PMC7449071; doi:10.1186/s12870-020-02602-1)

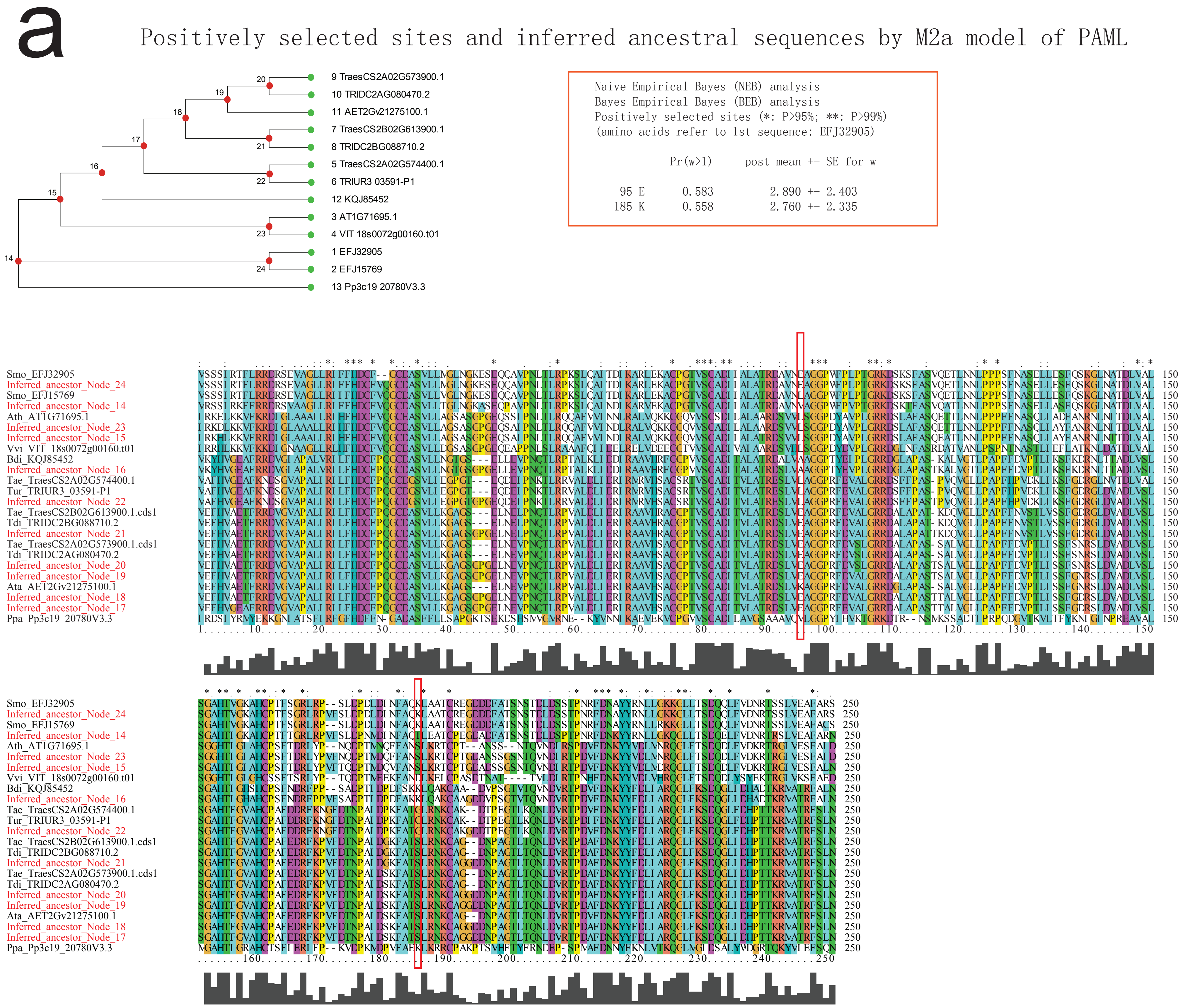
a
Positively selected sites and inferred ancestral sequences by M2a model of PAML
9 TraesCS2A02G573900.1
10 TRIDC2AG080470.2
11 AET2Gv21275100.1
7 TraesCS2B02G613900.1
8 TRIDC2BG088710.2
5 TraesCS2A02G574400.1
6 TRIUR3 03591-P1
12 KQJ85452
3 AT1G71695.1
4 VIT 18s0072g00160.t01
1 EFJ32905
2 EFJ15769
13 Pp3c19 20780V3.3
Naive Empirical Bayes (NEB) analysis
Bayes Empirical Bayes (BEB) analysis
Positively selected sites (*: P>95%; **: P>99%)
(amino acids refer to 1st sequence: EFJ32905)
Pr(w>1) post mean +- SE for w
95 E 0.583 2.890 +- 2.403
185 K 0.558 2.760 +- 2.335
Smo_EFJ32905
Inferred_ancestor_Node_24
Smo_EFJ15769
Inferred_ancestor_Node_14
Ath_AT1G71695.1
Inferred_ancestor_Node_23
Inferred_ancestor_Node_15
Vvi_VIT_18s0072g00160.t01
Bdi_KQJ85452
Inferred_ancestor_Node_16
Tae_TraesCS2A02G574400.1
Tur_TRIUR3_03591-P1
Inferred_ancestor_Node_22
Tae_TraesCS2B02G613900.1.cds1
Tdi_TRIDC2BG088710.2
Inferred_ancestor_Node_21
Tae_TraesCS2A02G573900.1.cds1
Tdi_TRIDC2AG080470.2
Inferred_ancestor_Node_20
Inferred_ancestor_Node_19
Ata_AET2Gv21275100.1
Inferred_ancestor_Node_18
Inferred_ancestor_Node_17
Ppa_Pp3c19_20780V3.3

b

Positively selected sites and inferred ancestral sequences by M8 model of PAML

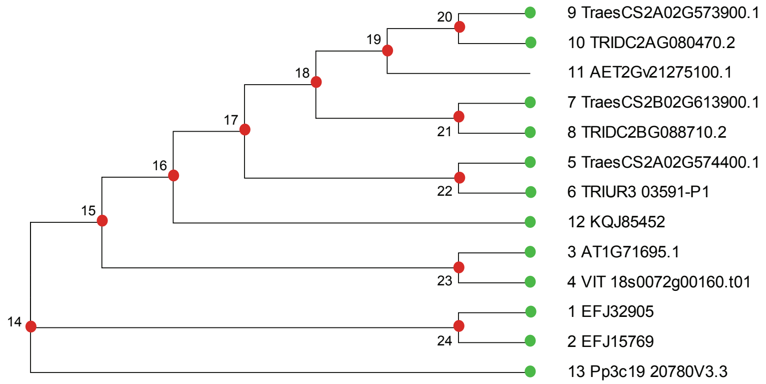

Naive Empirical Bayes (NEB) analysis
Bayes Empirical Bayes (BEB) analysis
Positively selected sites (*: P>95%; **: P>99%)
(amino acids refer to 1st sequence: EFJ32905)

		Pr(w>1)	post mean +- SE for w
95	E	0.843	2.107 +- 1.258
110	S	0.531	1.427 +- 1.021
117	Q	0.665	1.798 +- 1.321
135	E	0.608	1.538 +- 1.007
185	K	0.867	2.131 +- 1.237
212	R	0.704	1.842 +- 1.248

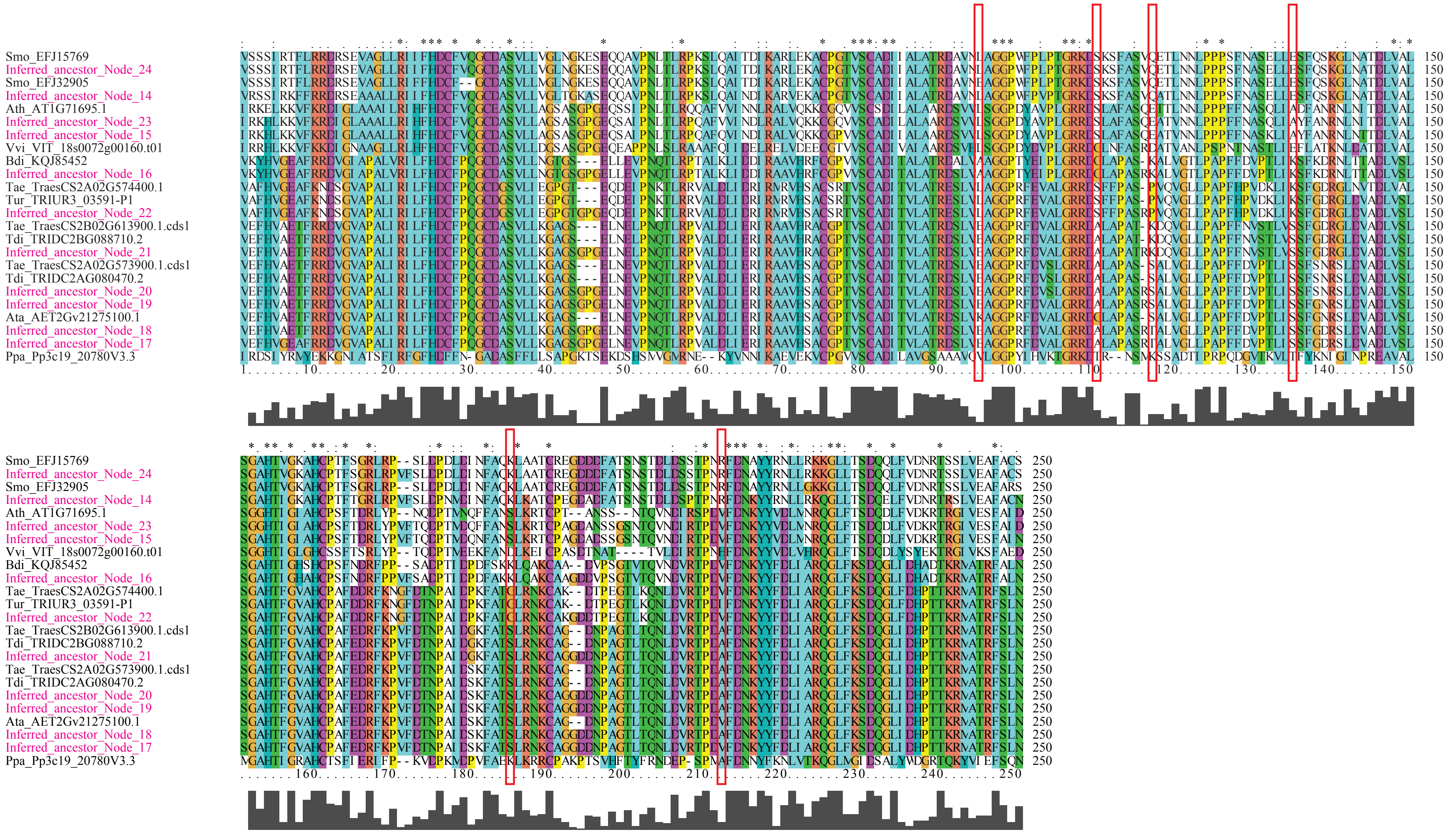

C

Inferred ancestral sequences by MEGAX

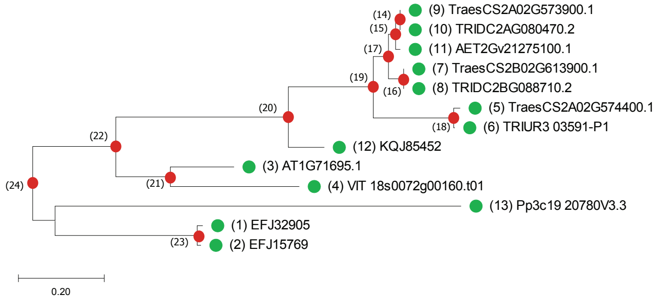

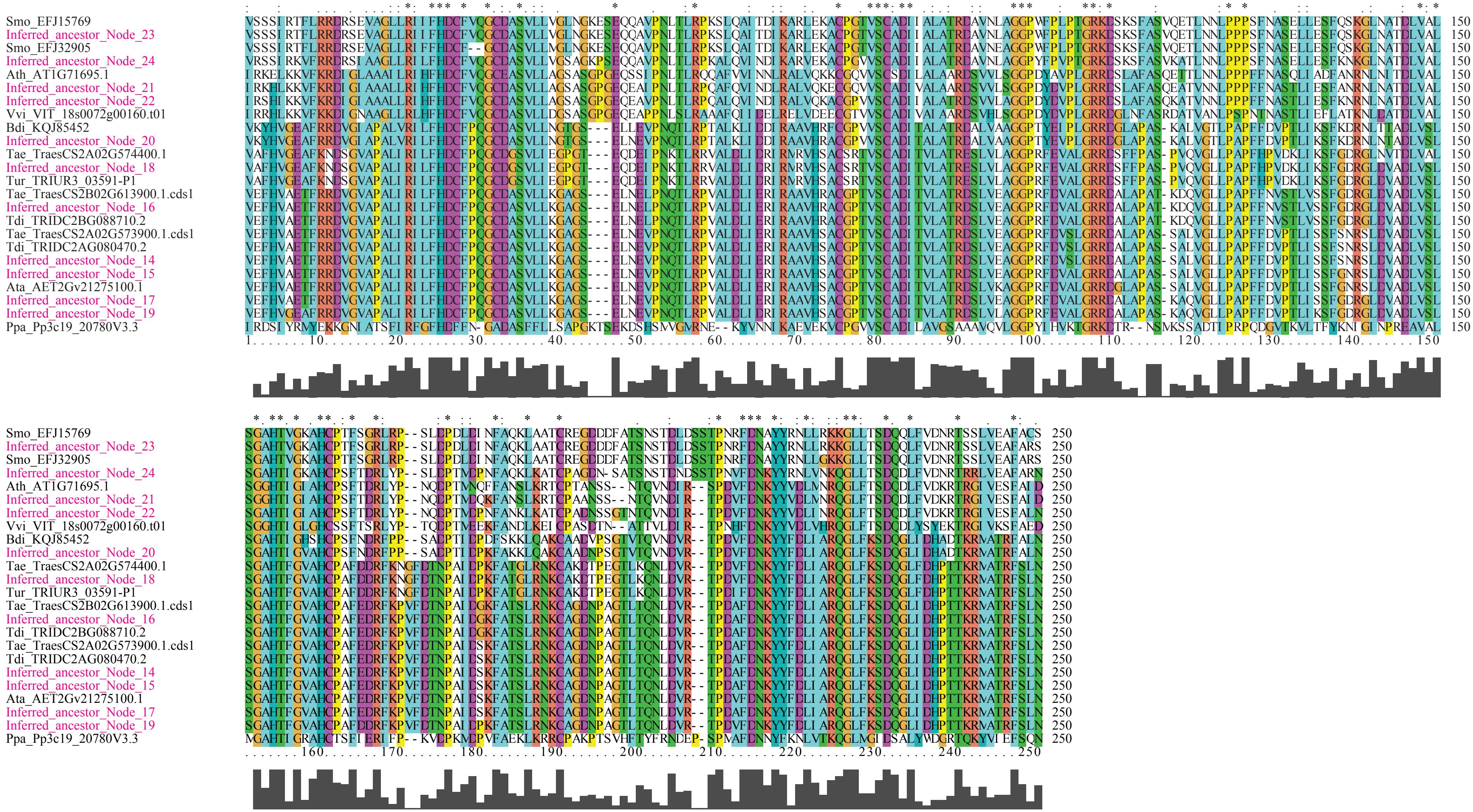

Supplement: Supplementary file 7 — Additional file 7: Figure S3. Positively selected sites and inferred ancestral sequences. (a) by using M2a model of PAML. (b) by using M8 model of PAML. (c) by using MEGAX. Inferred positively selected sites were circled by red boxes in the alignment of 13 PRXs and 11 inferred ancestral sequences. [file 12870_2020_2602_MOESM7_ESM.pdf]
