# Supplementary material for: A member of wheat class III peroxidase gene family, TaPRX-2A, enhanced the tolerance of salt stress
Source: BMC Plant Biol. 2020 Aug 26;20:392. doi: 10.1186/s12870-020-02602-1 (PMC7449071; doi:10.1186/s12870-020-02602-1)
Supplement: Supplementary file 9 — Additional file 9: Figure S5. The prediction of nuclear localization signals in TaPRX-2A. [file 12870_2020_2602_MOESM9_ESM.pdf]

cNLS Mapper Result

Predicted NLSs in query sequence	
MASRAAAAIAVLALVCAAVHSSEGQLSPNFHAATCPDLEHIVEFHVAETF	50
RRDVGVAPALIRILFHDCFPQGCDASVLLKGAGSELNEVPNQTLRPVALD	100
LIERIRAAVHSACGPTVSCADITVLATRDSLVEAGGPRFDVSLGRRDALA	150
PASSALVGLLPAPFFDVPTLISSFSNRSLDVADLVSLSGAHTFGVAHCPA	200
FEDRFKPVFDTNPAIDSKFATSLRNKCAGDNPAGTLTQNLDVRTPDAFDN	250
KYYFDLIARQGLFKSDQGLIDHPTTKRMATRFSLNQGAFFEQFARSMTKM	300
SNMDLLTGNKGEIRNNCAAPNRRVQDIETATTGDEGIAADM	341

Predicted monopartite NLS		
Pos.	Sequence	Score

Predicted bipartite NLS		
Pos.	Sequence	Score
221	TSLRNKCAGDNPAGTLTQNLDVRTPDAFDNK	2.1
271	DHPTTKRMATRFSLNQGAFFEQFARSMTKMS	2.1
271	DHPTTKRMATRFSLNQGAFFEQFARSMTKMSN	5
274	TTKRMATRFSLNQGAFFEQFARSMTKMSNMD	2.9
295	RSMTKMSNMDLLTGNKGEIRNNCAAPNRRVQD	2.1
